# From the Glovebox to the Benchtop: Air-Stable High Performance Molybdenum Alkylidyne Catalysts for Alkyne Metathesis

**DOI:** 10.1021/jacs.3c10430

**Published:** 2023-11-30

**Authors:** J. Nepomuk Korber, Christian Wille, Markus Leutzsch, Alois Fürstner

**Affiliations:** Max-Planck-Institut für Kohlenforschung, D-45470 Mülheim/Ruhr, Germany

## Abstract

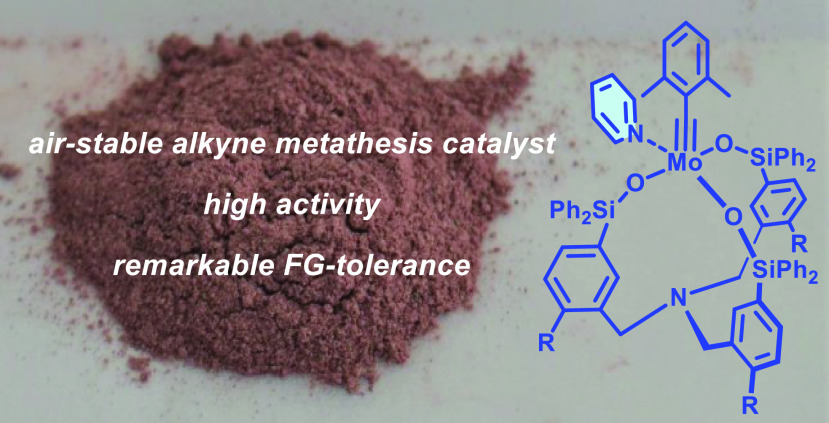

Molybdenum alkylidynes endowed with tripodal silanolate ligands belong to the most active and selective catalysts for alkyne metathesis known to date. This paper describes a new generation that is distinguished by an unprecedented level of stability and practicality without sacrificing the chemical virtues of their predecessors. Specifically, pyridine adducts of type **16** are easy to make on gram scale, can be routinely weighed and handled in air, and stay intact for many months outside the glovebox. When dissolved in toluene, however, spontaneous dissociation of the stabilizing pyridine ligand releases an active species of excellent performance and functional group tolerance. Specifically, a host of polar and apolar groups, various protic sites, and numerous basic functionalities proved compatible. The catalysts are characterized by crystallographic and spectroscopic means, including ^95^Mo NMR; their activity and stability are benchmarked in detail, and the enabling properties are illustrated by advanced applications to natural product synthesis. For the favorable overall application profile and ease of handling, complexes of this new series are expected to replace earlier catalyst generations and help encourage a more regular use of alkyne metathesis in general.

## Introduction

During the past two decades, alkyne metathesis has arguably evolved from a curiosity to a method of strategy level status in material science and small molecule synthesis alike.^1−8^ The progress is innately linked to advances in catalyst design;^9^ to this end, the focus increasingly shifted away from tungsten alkylidynes as the historic lead compounds^10−13^ to molybdenum alkylidynes endowed with ever more adequate ancillary ligands.^1−9,14,15^ These catalysts combine high activity with enabling functional group tolerance. The recent advent of non-d^0^ rhenium alkylidynes (e.g., **5**) with a somewhat complementary chemoselectivity profile constitutes another promising starting point.^16^

**Figure 1:**
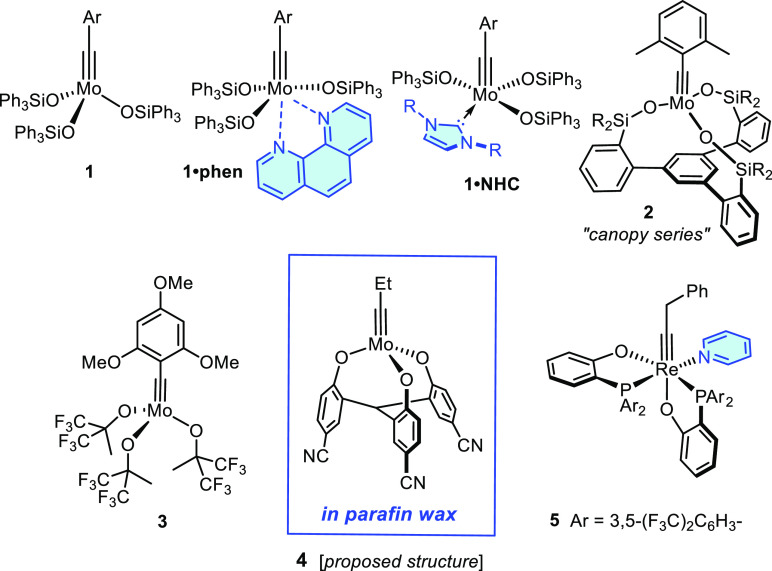
Selection of contemporary alkyne metathesis catalysts and (semi)stable versions thereof; the stabilizing ligand/matrix is shown in blue

Notwithstanding the high level of sophistication in the field, improved user-friendliness is desirable; only if the best catalysts are easy to handle are they going to be routinely used. Some milestones along this line have been reached in the past (Figure 1). While molybdenum alkylidynes such as **1** and **3**(17) themselves degrade within hours when kept in air, our laboratory showed that **1** forms adducts with phenanthroline or 2,2′-bipyridine that can be stored at the bench for extended periods of time.^18,19^ Although **1·phen** itself is catalytically inert, the stabilizing chelate ligand can be removed and the active species be released on treatment with a Lewis acidic additive such as ZnCl_2_ or MnCl_2_. This preactivation step is best carried out in toluene at ∼80 °C over the course of ca. 30 min (extended heating entails partial decomposition). The more Lewis acidic ZnCl_2_ is quite effective but has to be thoroughly dried prior to use; nonhygroscopic MnCl_2_, on the other hand, is only sparingly soluble in this solvent and does not lead to quantitative decomplexation. This shortcoming usually needs to be counterbalanced by higher loadings of **1**·**phen**/MnCl_2_ at the outset.^18−20^

The underlying concept was recently extended by the group of Buchmeiser, using N-heterocyclic carbenes (NHCs) instead of phenanthroline to form meta-stable adducts **1**·**NHC**.^21,22^ A particular complex of this type (Ar = 2,4,6-trimethylphenyl; NHC = 1,3-diisopropylimidazol-2-ylidene) was reported to show a lifetime in the solid state of more than a week on the bench; therefore, it can be weighed and transferred in air. The alkyne metathesis reaction itself then needs to be carried out in 1,2-dichloroethane under a nitrogen atmosphere at 80 °C to enforce (partial) decomplexation of the stabilizing NHC ligand (no activity was observed at room temperature). Under these conditions, good turnover numbers were secured at low catalyst loadings, at least for barely functionalized substrates.^21^

Other authors pursued different approaches toward user-friendly setups. A notable report by Zhang and co-workers showed that the (presumed) tripodal catalyst **4** generated in situ allowed the subsequent alkyne metathesis reactions to be carried out in air at 70 °C; however, hepatotoxic and potentially carcinogenic CCl_4_ is needed as the solvent to ensure optimal results.^23−25^ Attempts to protect the active species for benchtop storage by formulation in paraffin were only partly successful: although the activity of the catalyst wax was largely unchanged when kept on the bench for one day, a simple homometathesis reaction did not go to completion at 5 mol% loading after storage for one month.^23^

Collectively, these reports are encouraging but also show that there is much room for improvement. The next step forward needs to target a structurally well-defined catalyst system that (i) is air-stable for extended periods of time, (ii) can be stored and handled without any particular precautions, (iii) does not require a separate preactivation step, and (iv) operates in a benign solvent over a wide temperature range, ideally even at ambient conditions. At the same time, its activity and chemoselectivity profile should reach or even rival those of the best catalysts known to date. Outlined below is a significant step in this direction.

## Results and Discussion

### Design Concept

**Figure 2:**
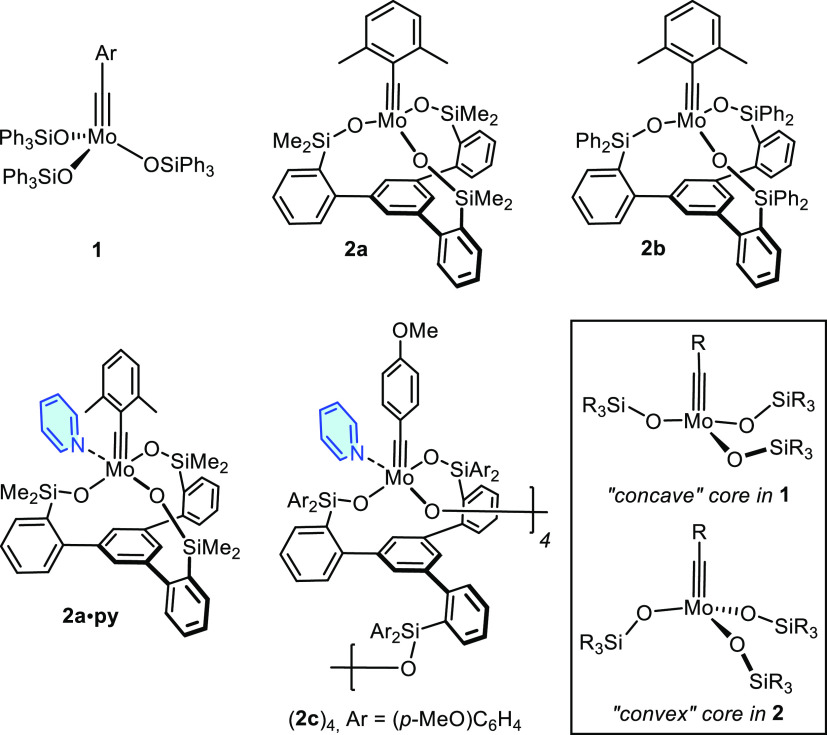
Prototype molybdenum alkylidyne tris-silanolate catalysts; attempted stabilization by ligation to pyridine. The parent complexes **1** differ from the “canopy” variant **2** in the curvature of the first coordination sphere about the metal center (see the Inset and the Text).

Molybdenum alkylidynes synergize exceedingly well with silanolates as ancillary ligands.^26,27^ The effect was originally discovered with the parent complexes **1**,^18,19,28,29^ but extends to more elaborate variants,^30^ including the catalysts of the “canopy” series featuring a tripodal silanolate ligand framework (Figure 2):^31−36^ complexes of type **2** show higher robustness against protic substituents by virtue of the chelate effect and an excellent compatibility with polar and apolar substiuents including Lewis basic functionality, which is noteworthy given the presence of a high-valent Mo(+6) center.^37,38^

The activity of the “canopy” catalysts strongly depends on the size and nature of the lateral groups R on the silicon atoms (e. g., **2a** ≫ **2b**). Actually, complex **2a** is (one of) the most active and selective alkyne metathesis catalysts known to date.^32^ However, neither **2a** itself nor the derived pyridine adduct **2a**·**py** are bench-stable: when kept in air, lilac/purple samples of **2a**·**py** turn brown within ≤1 day, and the active species is completely decomposed after ∼1 week. Moreover, attempted stabilization with pyridine may result in partial opening of the ligand framework as manifested in the cyclo-tetrameric adduct (**2c**)_4_.^34^ This structural reorganization is likely driven by strain release, in that the “convex” curvature of the “canopy” framework in **2** relaxes to a more favorable “concave” ligand environment about the Mo-centers in (**2c**)_4_, which is similar to what is observed for the parent complex **1** (see Inset).

**Scheme 1:**
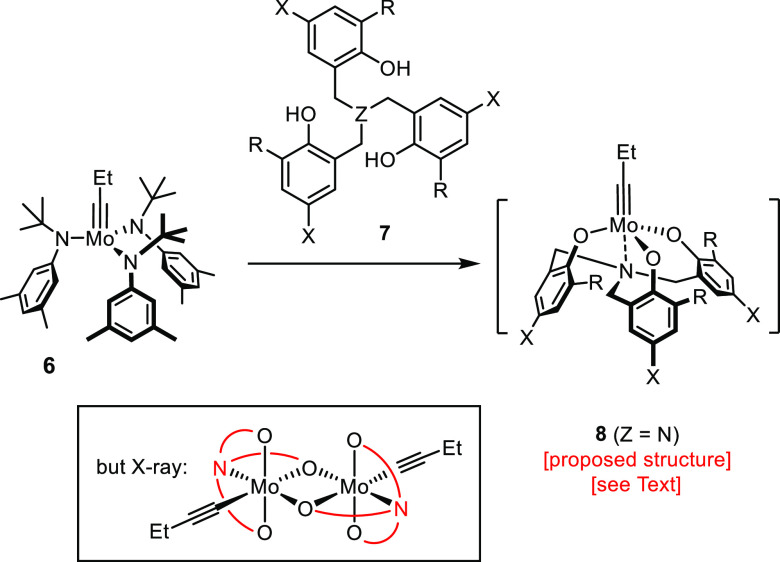
Alternative Catalyst Design Based on a Tethered Triphenol Ligand Scaffold, Exemplified by an N-Tethered Variant (Z = N, R = H)

An alternative ligand design was pursued by the Zhang group, in which three phenol (rather than silanol) units are connected via a central C-, N- or Si-atom (**7**, Scheme 1).^39−42^ The derived catalysts are usually drawn as tripodal *C*_3_-symmetric complexes **8**, but rigorous proof is missing; actually, rather complex NMR spectra are recorded upon mixing **6** and **7** (for an example showing the presence of more than one species of unknown constitution, see the Supporting Information). Moreover, the structures of **8a** (X = H, Z = N)^39^ and **8b** (X = NO_2_, Z = N)^43^ in the solid state are phenoxide-bridged dimers, with the N-atoms ligated *cis* rather than *trans* to each of the Mo-centers (see the Inset in Scheme 1). **8a** was described as bench-stable yet hardly reactive; reasonable activity was observed only after addition of a protic solvent that is supposed to disassemble the aggregate; the exact structure of the resulting species in solution remains unknown.^43^

**Scheme 2:**
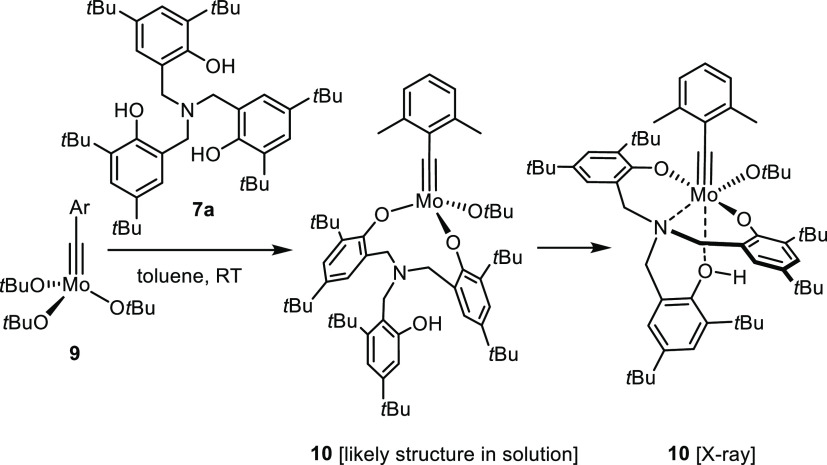
Sterically Demanding N-Tethered Trisphenol Ligand **7a** Fails To Afford a Tripodal Catalyst

**Figure 3:**
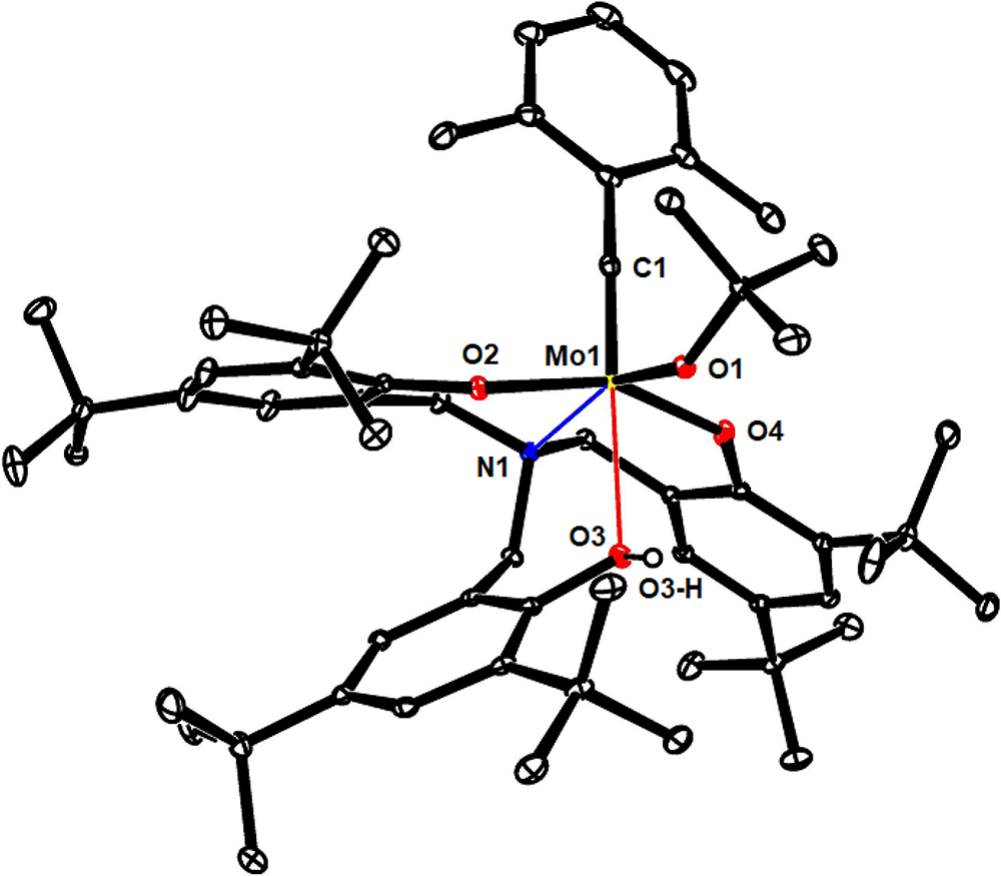
Structure of complex **10** in the solid state; all H-atoms, except the phenolic −OH, and solute benzene in the unit cell were removed for clarity. The full structure is contained in the Supporting Information.

Since the claim that the active species derived from ligands of type **7** are tripodal molybdenum alkylidynes had not been convincingly backed-up by experimental data, we were seeking additional information. To this end, the amine-tethered trisphenol **7a** carrying *tert*-butyl groups next to the phenolic −OH was prepared; it was expected that these bulky substituents would render dimerization of any derived complex less favorable (Scheme 2). The product generated upon reaction of **7a** with the more convenient precatalyst **9**(31,32,44,45) in toluene is indeed monomeric but retains a *tert*-butoxide moiety, which implies that a tripodal ligand framework cannot have formed. The structure of the heteroleptic complex in the solid state confirms this notion (Figure 3). In addition to the remaining *tert*-butoxide, two phenolates are covalently bound to the Mo center, whereas the third phenolic −OH group is intact; it occupies the axial site opposed to the alkylidyne unit. As the latter exerts a strong *trans*-influence, the Mo···OH interaction [2.474(1) Å] is weak. Indeed, the NMR spectra of **10** recorded in C_6_D_6_ are more in line with a species in which the phenol is freely dangling; moreover, the N-atom of the linker is also likely off the metal center in solution,^46^ whereas it is tightly bound *cis* to the alkylidyne in the solid state (Mo1···N1 2.296(1) Å). Taken together, these data suggest that the coordination chemistry of Zhang-type catalysts is far more intricate than what the literature suggests.

**Figure 4:**
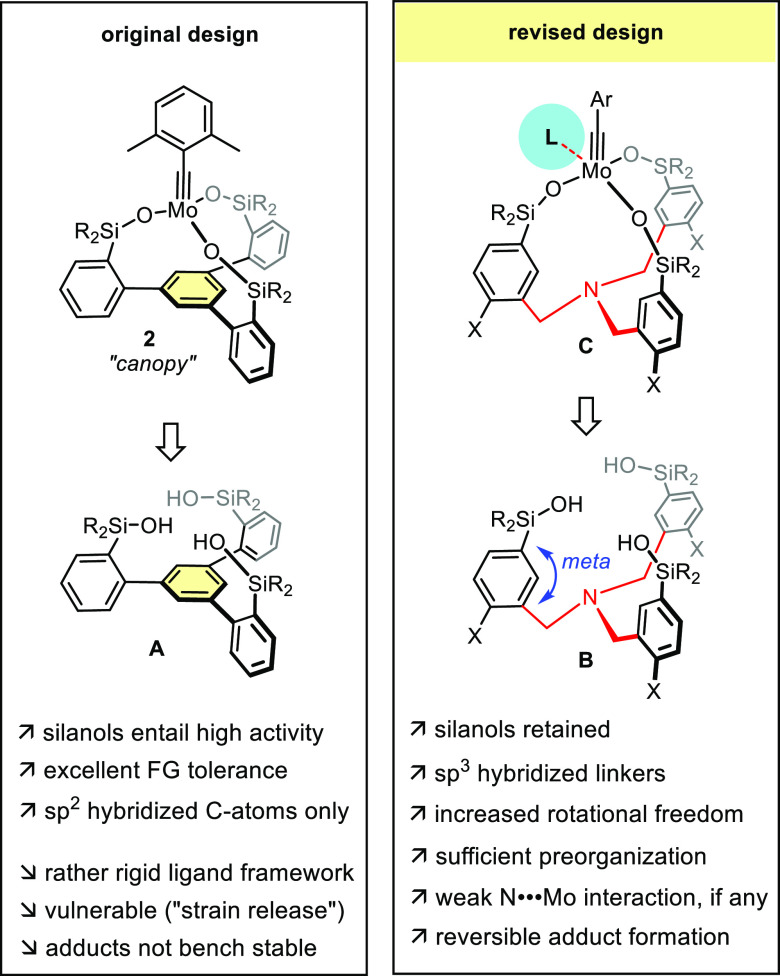
Design concept revisited.

In consideration of the prior art, we clearly favored a silanolate-based (rather than phenolate) ligand design en route to potentially bench-stable high-performance catalysts (Figure 4). To ensure formation of structurally well-defined tripodal complexes, the free ligand must be sufficiently preorganized to preclude competing oligomerization^30,34^ on reaction with **9**. Ideally, the resulting chelate complexes should be somewhat less rigid than those of the “canopy” series in order to accommodate all changes of the coordination geometry (tetrahedral, square-pyramidal, and trigonal-bipyramidal) that the complex has to adopt along the catalytic cycle, which will potentially benefit the activity. At the same time, a certain floppiness should prevent undesirable ring opening reactions driven by strain-release from occurring and hence improve stability.^34^

To achieve these critical objectives, the rigid benzene ring forming the basal plane of canopy ligand framework **A** was formally replaced by a central tris-benzylic amine in **B**, in the hope that the additional degrees of rotational freedom about the N–CH_2_–Ar bonds would provide the desirable level of flexibility without compromising the ligand preorganization altogether. A nitrogen-linker was chosen solely for the ease of synthesis in the expectation that the internal donor site will not quench the Lewis acidity necessary for a molybdenum alkylidyne to be catalytically competent.^26,27,33,47^

While the implementation of the N-tether into **B** obviously borrows from the ligands used by Zhang and co-workers (Scheme 1), it is important to note that the positioning of the −OH groups differs—on purpose—from this literature precedent.^39^ We conjectured that placement at the *ortho* position of the phenyl ring would actually be unfavorable on geometric grounds; rather, it was planned to install the R_2_Si-OH groups *meta* to the benzylamine linker, once again in the hope of relaxing the ligand backbone in a gentle but nondisruptive manner. As silanolates are less good bridging ligands than phenolates,^48^ it was also expected that unreactive dimers analogous to what is found for **8**(39,43) would not be formed at all or any such aggregate be easily broken upon addition of an external stabilizing ligand L.

The proper choice of “L” is arguably a critical parameter: on one hand, this ligand must bind tightly enough to the molybdenum alkylidyne in **C** in order to protect the catalyst and render the resulting adduct as bench-stable and storable as possible. On the other hand, ligation must be reversible, ideally without any extra chemical or physical stimulus to obviate the need for a separate preactivation step. Therefore, phenanthroline or bipyridine were sorted out,^18,19,49^ and monodentate donors less “sticky” than the NHCs used by the Buchmeiser group were sought.^21^ Since (canopy) molybdenum silanolate catalysts tolerate many different Lewis basic groups likely because their binding to the Mo(+6) center is reversible,^32^ we saw a window of opportunity to find the right match. Among the possible candidates, pyridine (or its commercial derivatives) was deemed a good starting point.

### Ligand and Catalyst Synthesis

**Scheme 3:**
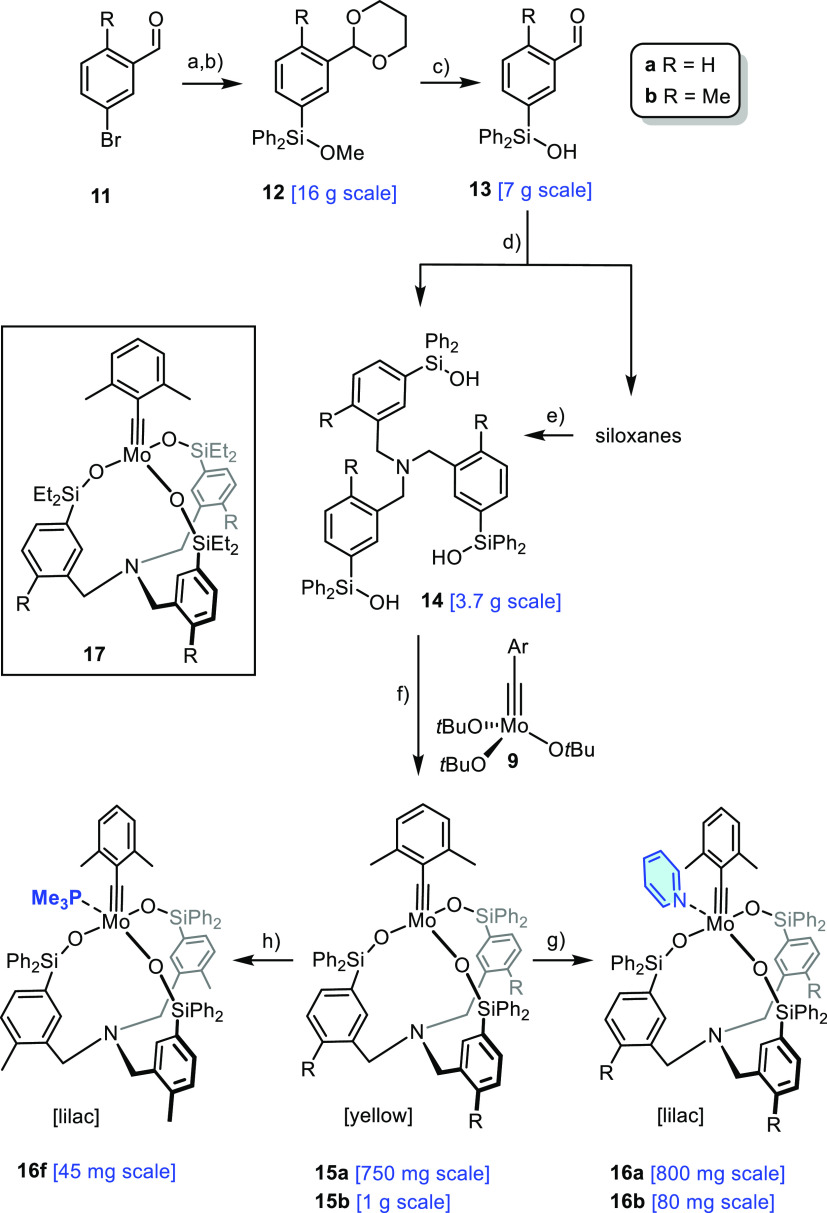
Preparation of Ligands and Catalysts Reagents and conditions: (a) 1,3-propanediol, *p*TsOH (3 mol%), toluene, reflux, 81% (R = H), 72% (R = Me); (b) (i) *i*-PrMgBr (0.4 equiv.), *n*-BuLi (0.8 equiv.), THF, 0°C; (ii) Ph_2_Si(OMe)_2_, 0°C → RT, 85% (R = H), 70% (R = Me); (c) HCl (6 M), THF, 0°C, 92% (R = H), 94% (R = Me); (d) NH_4_OAc, NaBH(OAc)_3_, THF; (e) NaOH (2 M), THF, 60% (over two steps, R = H), 65% (over two steps, R = Me); (f) **9**, toluene, 94% (R = H), 98% (R = Me); (g) pyridine, CH_2_Cl_2_, 88% (R = H), 77% (R = Me); (h) PMe_3_, CH_2_Cl_2_, 92%; the indicated scales refer to the single largest batch for R = Me (unless otherwise specified).

The preparation of the envisaged ligands of type **B** proved straightforward on a multigram scale (Scheme 3). The commercial bromobenzaldehydes **11** (R = H, Me) were transformed into the corresponding acetals prior to metal/halogen exchange on treatment with *i-*PrMgBr/*n-*BuLi.^50^ The resulting organometallic species were quenched with Ph_2_Si(OMe)_2_, and the siloxanes **12** thus formed treated with aq. HCl to concurrently unveil the Si-OH group and the aldehyde. Reductive amination of **13** furnished the targeted tris-silanols **14**; however, this step is accompanied by variable degrees of inter- and/or intramolecular siloxane formation. Stirring of the oligomeric fraction with aq. NaOH in THF rectified the issue and regenerated the desired monomer, thus making targeted ligand **14** available on scale in good overall yield from cheap starting materials.

**Figure 5:**
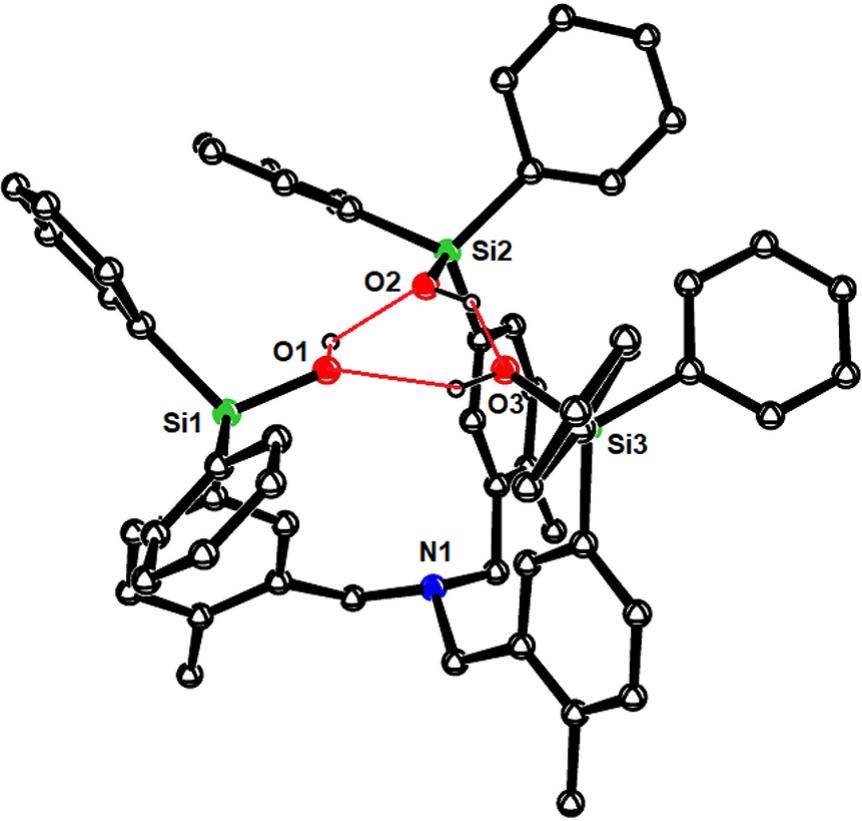
Structure of ligand **14b** in the solid state; all H-atoms were removed for clarity, except those of the Si–OH groups, which mutually engage in hydrogen bonding interactions (2.03 Å) and stabilize the favorable “upward/inward” conformation.

In the solid state, compound **14b** orients all three silanol groups “upward/inward” likely as the result of mutual intramolecular hydrogen bonding between the −SiOH groups (Figure 5). This preorganization of the ligand scaffold persists in an aprotic medium, as manifested in the NMR spectra that fit a *C*_3_-symmetric compound; chelate complex formation should hence be favorable. In fact, stirring a solution of **14** and **9** in toluene furnished the desired complexes **15** in excellent yields as yellow solid materials. The formation of oligomeric complexes^30,34^ does not interfere to any noticeable degree, and washing of the crude product with pentane suffices to obtain **15** in analytically pure form. A procedural adjustment also brought the ethyl variant **17** into reach, although the reductive amination during ligand synthesis was less efficient in this case (for details, see the Supporting Information). As the ethyl variant **17** proved to be less amenable to stabilization by ligation to external pyridine, we refrained from optimizing this step.

**Figure 6:**
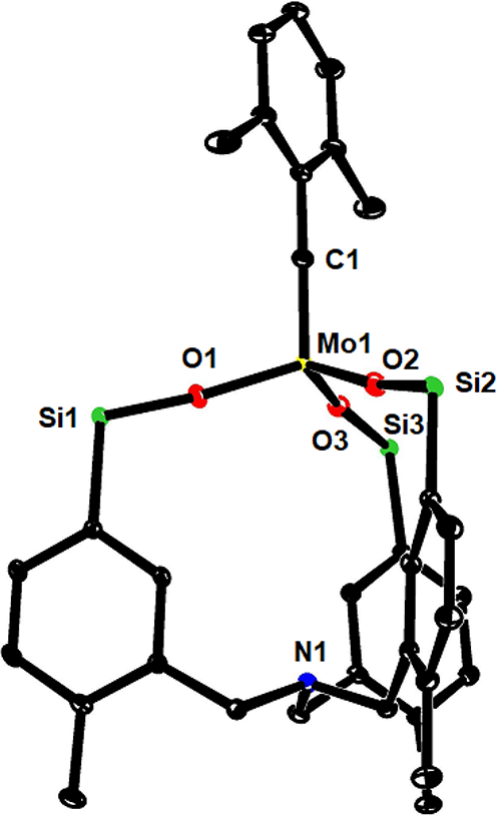
Truncated structure of complex **15b** in the solid state; the two phenyl rings on each of the Si-atoms were removed for clarity, and solute solvents and the second independent molecule in the unit cell are not shown either. The full structure is contained in the Supporting Information.

The recorded spectral data in [D_8_]-toluene are perfectly in line with the proposed tripodal constitution of these complexes. The fact that the ^15^N NMR shifts of the tethering N-atom in the free ligand **14b** (δ_N_ = −335 ppm) and the derived complex **15b** (δ_N_ = −333 ppm) are almost identical suggests that the tertiary amine does not interact with the Lewis acidic Mo(+6) center in vicinity. This notion is confirmed by the structure of **15b** in the solid state, which shows a quasi *C*_3_-symmetrical arrangement of the podand cap about the central metal (Figure 6) (the structure of **15a** is very similar, see the Supporting Information). With a Mo1/N1 distance of no less than 5.30 Å, a bonding interaction can definitely be excluded, even though the nitrogen lone-pair points inside the cage; it is the *meta*-substitution pattern of the phenyl rings that precludes transannular binding from occurring and sets complexes **15** apart from the phenolate-based Zhang-type catalysts alluded to above.^39,43^ For the lack of any significant N→Mo electron donation, the favorable Lewis acidic properties imparted onto the molybdenum alkylidyne by the silanolate units should neither be mitigated nor quenched; good catalytic performance can therefore be anticipated. This expectation is substantiated by the recorded ^13^C and ^95^Mo NMR shifts (**15b**: δ_C_ = 309.6 ppm; δ_Mo_ = 495 ppm) of the alkylidyne unit, both of which fall into the range recently defined as particularly pertinent for active alkyne metathesis catalysts (δ_C_ ≈ 300–317 ppm; δ_Mo(iso)_ > 350 ppm).^27^ If these criteria are applied, complex **10** bearing the Zhang-type phenolate-based ligand (δ_C_ = 292.7 ppm; δ_Mo_ = 349 ppm) should be a borderline case; indeed, it showed only modest catalytic activity even at elevated temperatures (see below).

A comparison of **15** with the “canopy catalysts” **2** is informative too. As mentioned in the Introduction, the ligand sphere of **2** comprising only sp^2^-hydridized C-atoms is somewhat strained, as manifested in its “convex” curvature (see Insert in Figure 2).^34^ In contrast, the new ligand architecture of **15** accommodates a more relaxed and largely “concave” environment, which should make the complexes less vulnerable on treatment with external donors that may serve their stabilization.

### Reversible Adduct Formation

**Figure 7:**
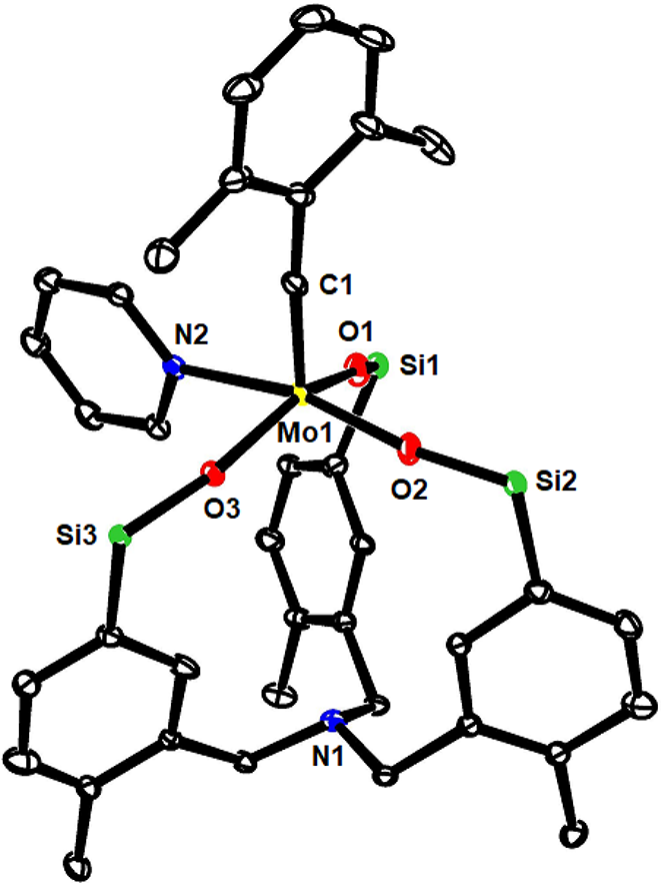
Truncated structure of pyridine adduct **16b** in the solid state; the two phenyl rings on each of the Si-atoms were removed for clarity. The full structure is contained in the Supporting Information.

Addition of a slight excess of pyridine to a yellow solution of **15b** in CH_2_Cl_2_ at ambient temperature causes a color change to deep lilac. Evaporation of the solvent and washing of the residue with pentane to remove excess pyridine afforded the desired adduct **16b** as a lilac powder that can be recrystallized from CH_2_Cl_2_/pentane to give purple single crystals suitable for X-ray diffraction. The structure in the solid state (Figure 7) shows that the external pyridine ligand is tightly bound (Mo1-N2 2.255(1) Å), whereas the N-atom of the tris-benzylic linker is off the metal center (Mo1/N1 5.38 Å); it has solely a geometric function but exerts no electronic effect.

Interestingly, a lilac solution of **16** in [D_8_]-toluene turns yellow/brown again upon gentle warming from 298 to 323 K. The effect is reversible, which suggests that decomplexation/recomplexation of pyridine is facile and nondestructive. Variable temperature (VT) ^1^H NMR spectra confirm this arguably favorable property (for details, see the Supporting Information): the fact that all signals are broad at 298 K likely indicates that **15** and **16** are in equilibrium even at ambient temperature. Warming shifts the equilibrium and entails an essentially quantitative release of *C*_3_-symmetric yellow catalyst **15**, in line with the observed color change. Appreciable catalytic activity can hence be expected at/close to room temperature. In contrast, sharp NMR signals are recorded at 233 K; the observed lower symmetry indicates that adduct **16** is present, and the binding of the pyridine is tight at this low temperature.

A number of pyridine derivatives differing in steric demand and donor ability was also screened (for the resulting adducts **16c**-**f**, see the Supporting Information).^51^ Whereas 2,6-dimethylpyridine does not bind at all to **15**, the adducts derived from 3-bromopyridine and 3,5-dibromopyridine proved (too) labile.^52^ The more electron rich 4-pyrrolidinopyridine led to a stable adduct that is hardly prone to decomplexation at room temperature. Moreover, adduct formation with PMe_3_ is possible and reversible; as the resulting complex **16f** does not provide a significant advantage over the pyridine adducts, it was not investigated in detail. Overall, this brief survey suggests that cheap pyridine itself—though definitely not the only option—is actually a good comprise: the resulting adduct **16** is storable at the benchtop or in a freezer for extended periods of time (see below) and can be weighed in air, while solutions in toluene show excellent catalytic activity and allow a multitude of challenging alkyne metathesis reactions to be carried out at or slightly above room temperature.

### Stability in Air

Even the pyridine-free complex **15b** has a half-lifetime on the order of days when kept in air as a microcrystalline powder (for details, see the Supporting Information); it can be weighed and handled without particular precautions, although long-term storage mandates inert conditions.

**Figure 8:**
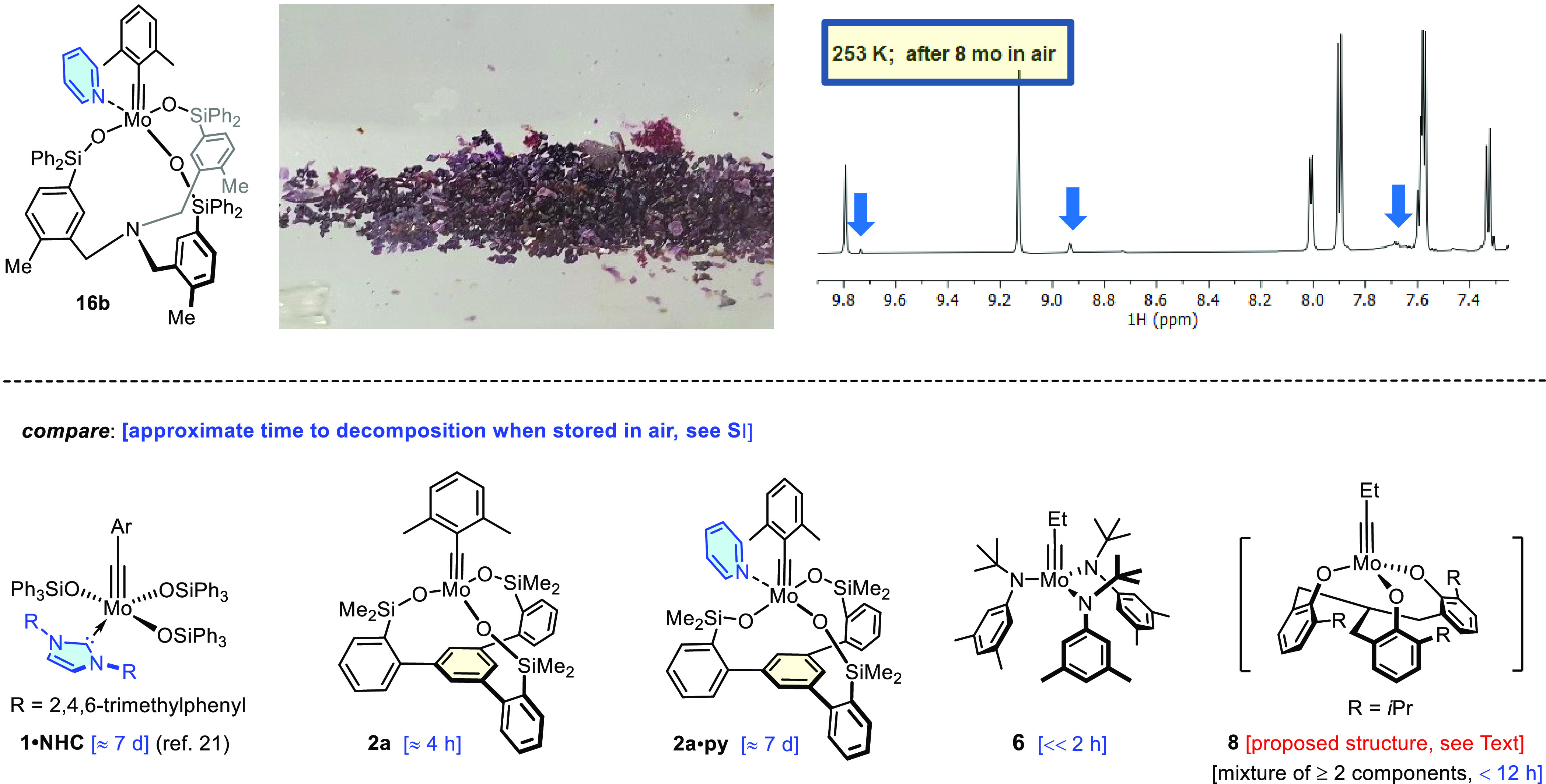
Top: Photograph of a sample of the crystalline pyridine adduct **16b**; aromatic region of the ^1^H NMR spectrum ([D_8_]-toluene, 253 K) of this sample after storage in air at ambient temperature for 8 months, showing only trace impurities caused by hydrolysis (for the full spectra, see the Supporting Information). Bottom: approximate lifetimes of other molybdenum alkylidyne catalysts when stored in air.

As expected, complexation to pyridine increases the stability to a significant extent. Hardly any signs of degradation were detected by ^1^H NMR when adduct **16b** in crystalline form was stored in air at ambient temperature for up to eight months (Figure 8, top). When the product is kept in air as a powder at ambient temperature, hydrolysis becomes more prominent with time. However, the integrity of such powder samples is easy to ensure simply upon storage in a desiccator or, alternatively, in a screw-capped vial in a freezer. Under these conditions, powder samples of **16b** remain intact for many months as proven by NMR as well as elemental analysis; importantly, all catalytic test reactions occurred with unchanged rate and efficiency (see the Supporting Information). Although the pyridine adducts **16** are hence not fully inert toward moisture and will eventually hydrolyze, long-term storage is possible outside a glovebox under conditions that are easy to realize in any chemical laboratory. To the best of our knowledge, lifetimes on the shelf of this order of magnitude exceed—by far—everything reported in the literature for the molybdenum alkylidyne series (Figure 8, bottom). Adducts **16** are hence deemed enabling and practical tools adequate for use even by nonexperts.

### Chemical Stability

**Scheme 4:**
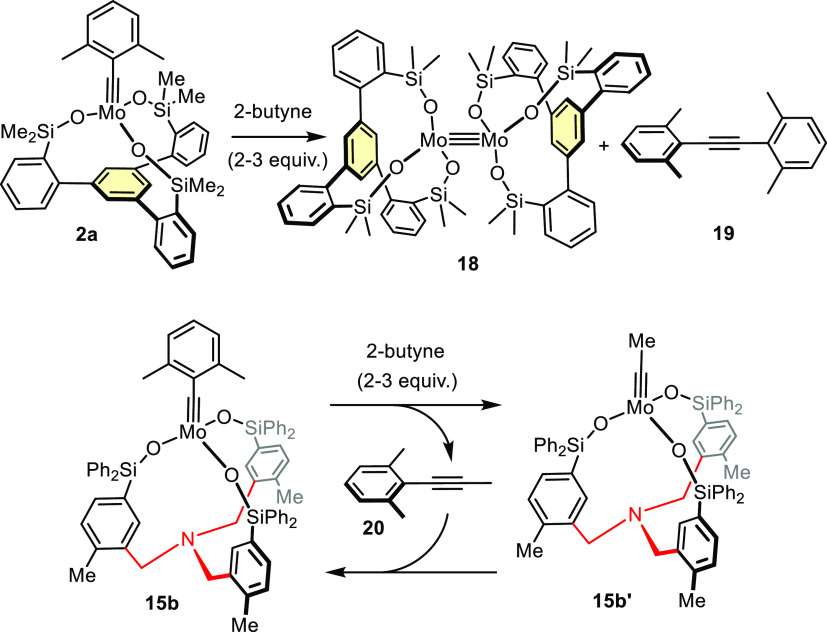
Distinct Behavior of Tripodal Molybdenum Alkylidyne Silanolate Complexes Vis-à-Vis 2-Butyne

Complex **2a** as the most active of the “canopy” series is prone to bimolecular decomposition. When exposed to 2-butyne in toluene at room temperature, it converts within ∼60 min into a mixture of the homobimetallic complex **18** and tolane **19** (Scheme 4);^34^ this surprisingly facile transformation is supposedly one of the reasons why fairly high loadings of **2a** are needed in many applications, despite the high inherent activity of this catalyst, particularly if the reactions have to be performed at elevated temperatures.

The new complexes are not nearly as vulnerable as **2a**: although ^1^H NMR showed that **15b** rapidly reacts with 2-butyne, the characteristic signals of the original 2,6-dimethylbenzylidyne group reappeared after stirring of the solution for ≈1.5 h at 50 °C; during that time, the 2-butyne was polymerized without damaging the active species;^53^ once the butyne is depleted, the latter traps remaining **20** to regenerate complex **15b**. Other aliphatic alkynes will eventually polymerize too, but this undesirable side reaction is (much) slower than productive metathesis, as exemplified for 2-octyne (see the Supporting Information); only in case of product **31** (see below) did polymerization interfere to a noticeable extent.^54^

### Benchmarking

**Scheme 5:**
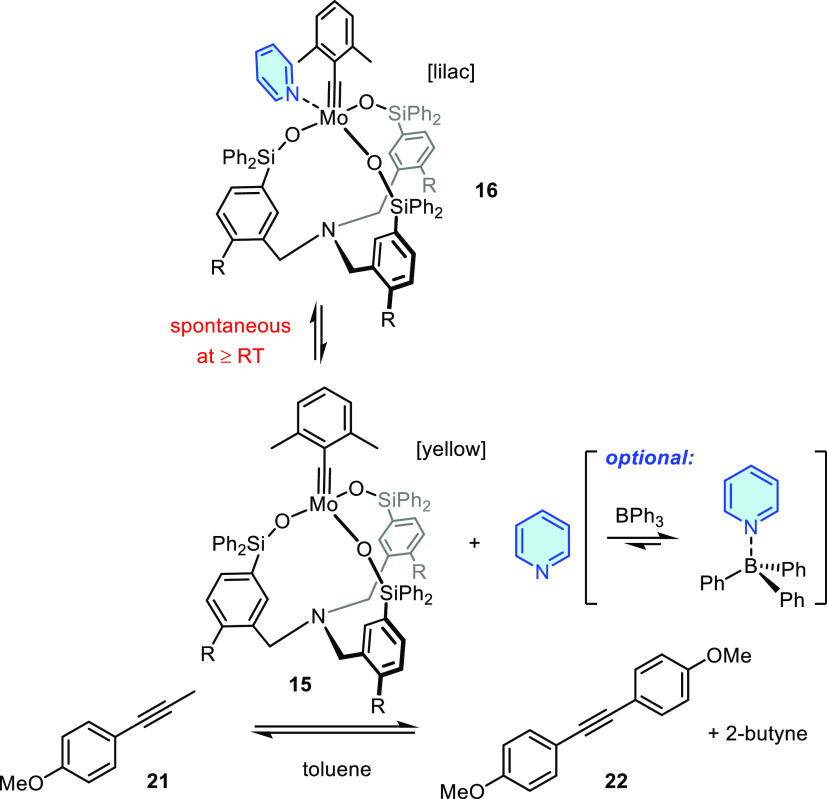
Model Alkyne Metathesis Reaction Used for Benchmarking Purposes

The homometathesis of alkyne **21** to the tolane derivative **22** allowed the activity of the new catalysts and the derived pyridine adducts to be assessed (Scheme 5). The reactions were performed in [D_8_]-toluene in the absence of molecular sieves as butyne-sequestering agent;^18,19^ they were monitored by ^1^H NMR spectroscopy until the equilibrium was reached.

**Figure 9:**
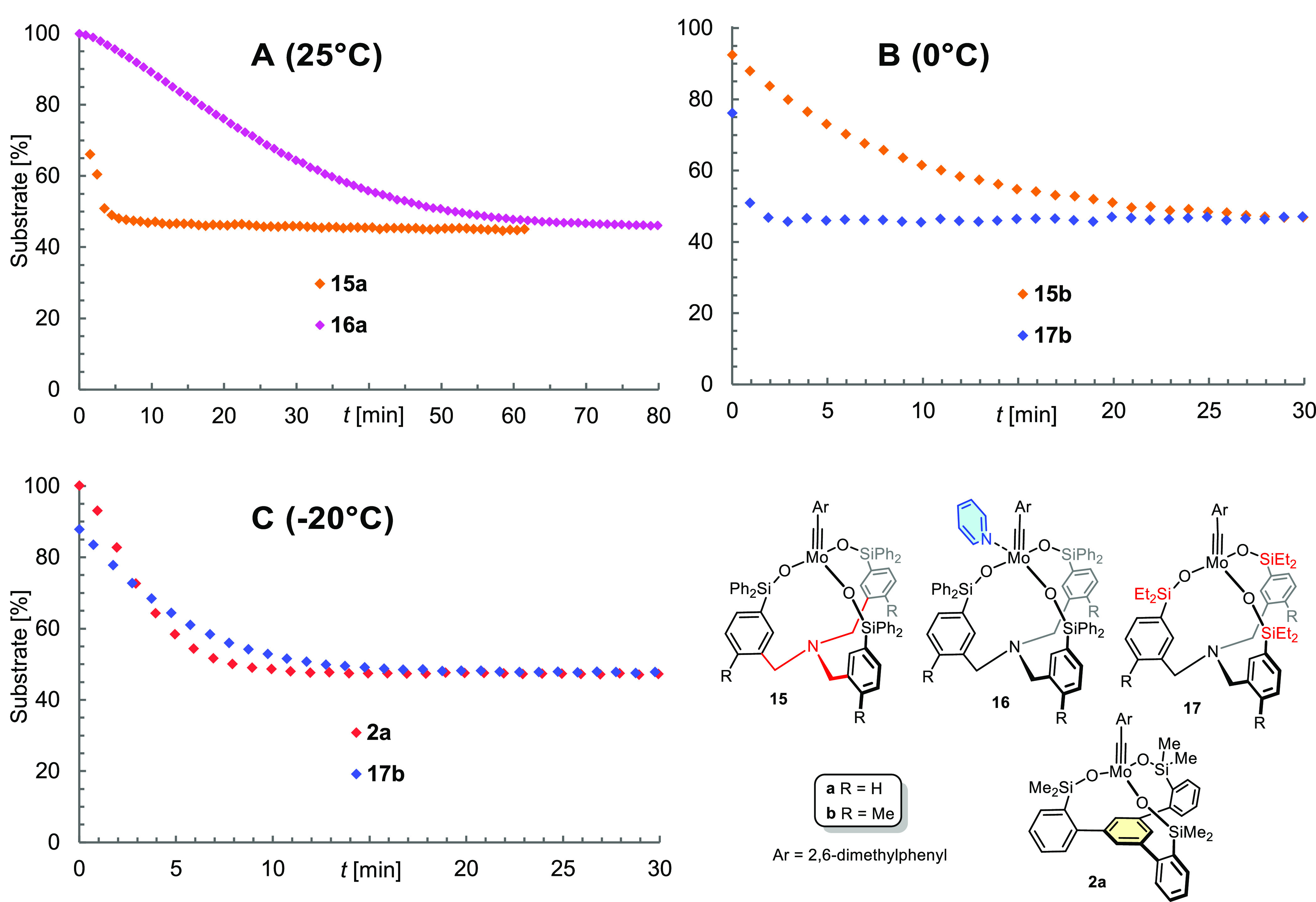
Consumption of alkyne **21** with time as monitored by ^1^H NMR spectroscopy in different benchmarking experiments using 5 mol% of the respective catalyst in [D_8_]-toluene. (A) Comparison of the tripodal complex **15a** and the derived pyridine adduct **16a** at 25 °C; (B) comparsion of complexes **15b** and **17**, differing only in the substituents at the silicon linkers at 0 °C; (C) comparison of the canopy catalyst **2a** and complex **17b** at −20 °C.

Several conclusions can be drawn from the recorded data. First, the activity of the new tripodal complexes **15** is excellent in that it takes ≤ 5 min to reach equilibrium at 25 °C (Figure 9A). For comparison: complex **10** bearing the modified phenolate-based Zhang-type ligand is much less active under these conditions; even when heated to ≥ 70 °C it requires ca. 120 min to entail equilibration (see the Supporting Information).^55^

The test reaction had to be performed at 0 °C in order to see differences between **15b** and ethyl variant **17b**, with the latter being more active (Figure 9B). The rather close margin came as a surprise since the nature of the substituents on silicon has a pronounced influence on the catalytic activity in the original “canopy” series (Me > Et ≫ Ph).^32,34^ Actually, the run at −20 °C shows that the performance of **17b** comes fairly close to that of the most active “canopy” catalyst **2a** known to date (Figure 9C).^32,56^ The ability to carry alkyne metathesis reactions out at such low temperature is *per se* a remarkable finding and, to the best of our knowledge, unprecedented in the literature (see also below).^57^ We suppose that these observation reflect the good balance between flexibility and stiffness of the new ligand framework: it is sufficiently floppy to accommodate the different coordination geometries that the reactive intermediates pass through during a productive catalytic cycle (tetrahedral, square-pyramidal, trigonal-bipyramidal).^26,33^ In the solid state, however, the backbone is sufficiently rigid that the sensitive molybdenum alkylidyne is protected by the encircling fence formed by the lateral −SiR_2_ substituents, provided they are sufficiently large (Ph > Et); this feature translates into the high stability on the bench, especially of the pyridine adducts.

As forecasted by the NMR data, the pyridine adducts **16** also exhibit appreciable catalytic activity at ambient temperature by virtue of the spontaneous release of **15** in toluene solution; the equilibrium is reached in ≈60 min at 25 °C (Figure 9A). As expected, raising the temperature results in a massive rate acceleration.

### Promotion by BPh_3_

Although these results prove that the air-persistent pyridine adducts **16** are fully competent catalysts that do not need any preactivation other than dissolution in an inert solvent, it is possible to promote the reaction, if one so desires. Added BPh_3_ (1 equiv. relative to **16**) scavenges the pyridine and hence largely shifts the equilibrium between **15** and **16** to the side of the free catalyst (Scheme 5); once again, this comes along with a characteristic color change of the solution from lilac to yellow. Under these conditions, the test reaction proceeded within minutes at ambient temperature (for details, see the Supporting Information). As the stability of the pyridine adducts **16** increases upon cooling, addition of BPh_3_ is necessary for reactions carried out below room temperature (for the homometathesis of **21** at 0 °C catalyzed by **16a**/BPh_3_, see the Supporting Information). While promotors other than BPh_3_ can be envisaged, this crystalline compound was chosen for the ease of handling and its benign character, which likely poses little risk in advanced applications.

**Scope**. Based on our previous experiences with molybdenum alkylidynes endowed with silanolate ligands,^1,4^ we anticipated a favorable application profile for the chelate catalysts of the new series. This expectation proved correct. Most reactions were performed with 2 mol% of adduct **16a** as the catalyst in toluene at 50 °C in the presence of MS 5Å as scavenger for the released 2-butyne;^18,19^ however, higher catalyst loadings (usually 5 mol%) were employed for substrates carrying protic substituents.

**Scheme 6:**
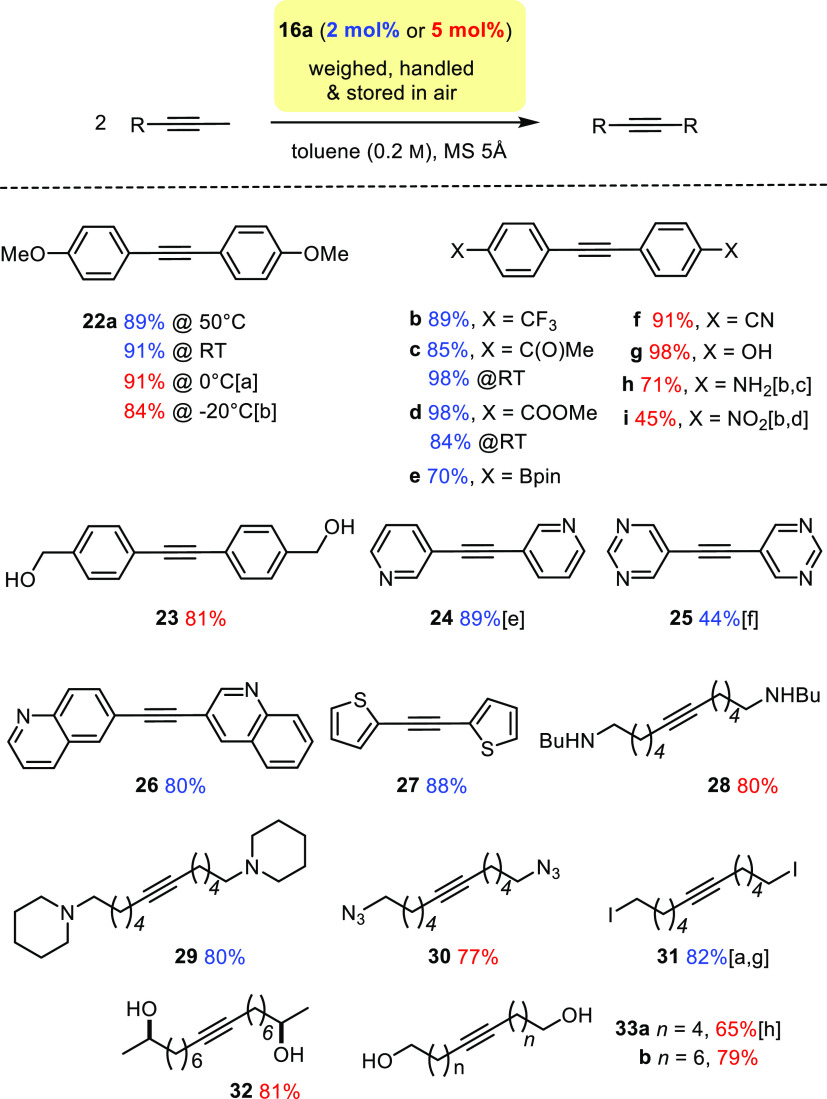
Homo-metathesis Reactions of Functionalized Substrates Using Complex **16a** as the Catalyst at 50°C, unless Stated Otherwise; the Chosen Catalyst Loading Is Color-Coded (Blue = 2 mol%; Red = 5 mol%) With added BPh_3_ (5 mol%). Using the free complex **17** instead of adduct **16a**. At 110°C. With 7 mol% of catalyst at 110 °C. At 90°C. At 100°C. At RT. With MS 4Å (instead of 5Å).

The first round of evaluation focusing on homometathesis reactions quickly confirmed the broad functional group tolerance of **16** (Scheme 6). As proven by the formation of **22a**, advantage can be taken from the broad temperature range at which the catalysts are active, although the combination **16**/BPh_3_ or the unstabilized complex **17** are needed when the reactions are performed at 0 °C or even −20 °C. Substrates comprising protic groups may eventually react with the complex, replace the silanolates and, in doing so, spoil catalytic activity; therefore, they had marked an important limitation for alkyne metathesis in the past and were one of the main reasons for the development of catalysts harnessing the chelate effect.^30−32^ The new user-friendly catalysts **16** are operative in the presence of primary and secondary aliphatic, benzylic and propargylic alcohols, a phenolic −OH, the −NH_2_ substituent of an aniline, and the −NH group of a secondary amine. As mentioned above, however, a loading of 5 mol% was used in these cases to ensure full conversion.

The fact that binding of external pyridine to the active molybdenum alkylidyne is reversible even at ambient temperature forecasts excellent compatibility of **16** with various donor sites. This virtue is perhaps best appreciated if one considers that even the venerable Grubbs-type catalysts for olefin metathesis are quite sensitive toward basic functionality.^58−60^ Specifically, free amines, pyridines and related basic heterocycles are usually not tolerated unless being protonated or properly protected otherwise;^61^ likewise, nitrile-containing substrates tend to be challenging for ruthenium carbenes. When seen against this backdrop, the compatibility of complexes **16** comprising an early transition metal center in the highest possible oxidation state with pyridine, pyrimidine, quinoline, thiophene, thiazole, N-alkyl piperidine, an aliphatic secondary amine, aniline, and benzonitrile is noteworthy; higher reaction temperatures were necessary in those cases in which the functional group renders the triple bond electron-deficient. This favorable profile also distinguishes **16** from [(*t*BuO)_3_W≡CCMe_3_] as the prototype of a catalytically active alkylidyne complex, which had previously failed to convert substrates comprising a thiazole or thiazolidinone ring^62,63^ or even a pyridine of strongly reduced basicity.^64^

Classical Grubbs-type catalysts are also incompatible with compounds carrying strong alkylating agents and azides. An unhindered primary alkyl iodide will eventually react with the PCy_3_ ligand once it decoordinates from the Ru center. The same is true for azides, which engage the phosphine in a Staudinger reaction; either process will ultimately destroy the catalyst and substrate alike. Although such damage can be circumvented by choosing phosphine-free ruthenium complexes,^58^ it is worth mentioning that no such complications arise when working with **16** as illustrated by the high-yielding formation of products **30** and **31**.^65^

**Scheme 7:**
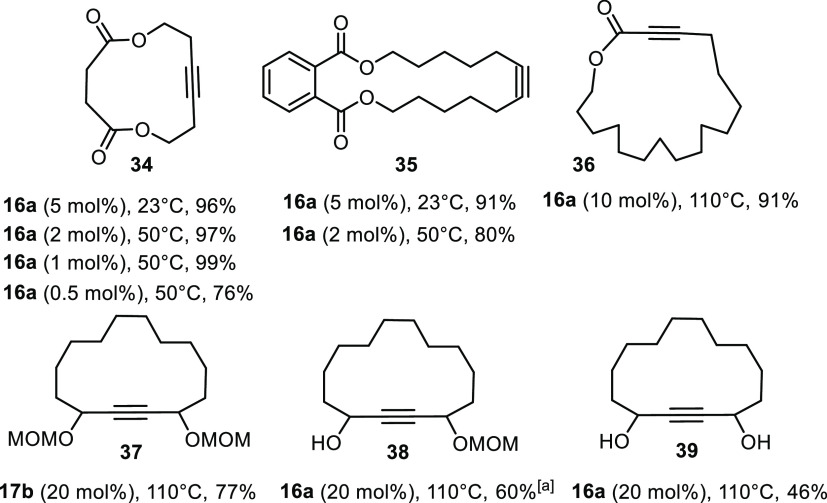
Macrocycles by Ring Closing Alkyne Metathesis All reactions were performed in toluene (2 mM) in the presence of MS 5Å using the indicated catalyst; note that complex **16a** was weighed, handled and stored in air. All substrates were carrying methyl caps on the triple bonds. A modified “expedited” workup was necessary to obtain pure samples; see the Text.

A number of ring closing alkyne metathesis (RCAM)^66^ reactions reinforce the favorable impression (Scheme 7). As expected, the simple macrocycles **34** and **35** were obtained in excellent yields; the reactions can be performed at room temperature or above, as deemed desirable; the comparison also shows that there is room for optimizing the catalyst loading as the formation of **34** proceeded well even with 1 mol% or less of adduct **16a**. Product **36** constitutes a more stringent test: for their low reactivity, ynoates and related substrates had been beyond the scope of [(*t*BuO)_3_W≡CCMe_3_] as the historic landmark in the field.^64,67^ As far as we know, only molybdenum alkylidynes with silanolates have so far been shown to be capable of forming macrocyclic ynoates by RCAM;^28,68^ the new user-friendly variant **16** retains this ability.

A short survey was conducted to study how different substituents on the reacting triple bonds impact the outcome of the RCAM reaction (Table 1). As expected, cyclization of **40a** bearing methyl caps was high yielding, but the reaction of **40b** carrying propyl groups instead was equally productive. This result reflects the ability of MS 5Å to sequester not only 2-butyne but also other short unbranched aliphatic alkynes such as 4-octyne.^69^ Even diyne **40c** with a silyl terminus could be ring-closed in high yield, although more forcing conditions and a longer reaction time were necessary. This observation confirms previous findings that silylated alkynes are generally less reactive than “ordinary” alkynes.^70,71^ While the reaction rates and temperatures differ, the efficiency of ring closure is largely unaffected by the choice of end caps. This fact is arguably of preparative significance, as it provides flexibility when planning a (target-oriented) synthesis based on RCAM.

**Table 1:**
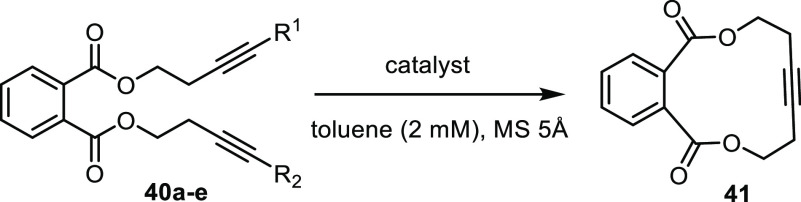
Survey of the Effect Exerted by the Alkyne Caps on RCAM

Entry	Substrate	R^1^	R^2^	Catalyst	Additive	*T* (°C)	Yield
1	**40a**	Me	Me	**16a** (5 mol%)	—	23	90%
2	**40b**	C_3_H_7_	C_3_H_7_	**16a** (5 mol%)	—	50	90%
3	**40c**	Me	SiMe_3_	**16a** (5 mol%)	—	100	86%^a^^,^^b^
4	**40d**	Me	H	**16a** (5 mol%)	—	23/50	—c
5				**15a** (5 mol%)	—	23	66%
6				**16a** (10 mol%)	BPh_3_	23	92%^d^
7	**40e**	H	H	**16a** (10 mol%)	BPh_3_	23	16%^d^

a The reaction was performed in the absence of molecular sieves.

b Diyne **40** with R^1^ = R^2^ = SiMe_3_ was formed as a byproduct (ca. 7%).

c No reaction was observed at RT, whereas the substrate was polymerized when the mixture was heated to 50 °C.

d In presence of MS 4Å and MS 5Å.

Not unexpectedly, the challenge increases if one uses substrates comprising a terminal alkyne: such compounds are prone to polymerization on exposure to metal alkylidynes and have long been elusive until certain molybdenum alkylidynes proved competent.^28,70,72,73^ The new complexes fall into this category even though the window of opportunity is narrower: while the air-persistent pyridine adduct **16** failed,^74^ the unbound catalyst **15a** and the combination **16a**/BPh_3_ proved effective. Polymerization prevailed with substrate **40e** comprising two terminal alkynes and only a poor yield of product **41** was obtained; a few exceptions notwithstanding, such substrates continue to mark a limitation.

### Expedited Workup

The formation of cycloalkynes such as **37**-**39** carrying −OR substituents on both propargylic positions is also known to be highly demanding (Scheme 7). Only few such examples are known in the literature, again relying on molybdenum alkylidynes ligated to silanolates.^30,32^ It is therefore noteworthy that **16** and **17** fall into the elite class of catalysts able to form such compounds, although it was necessary to increase the loading to 20 mol% for full conversion. However, product **38** and the tris-silanolate ligand hydrolyzed off the catalyst upon workup were found to coelute during flash chromatography, thus making product purification challenging; this phenomenon is not uncommon when working with silanol derivatives and silica as the stationary phase. The N-atom in the ligand backbone proved handy to address the issue. The reaction mixture was simply washed with aqueous HCl prior to the chromatographic purification of the crude material; under these conditions, the ligand was quantitatively removed. This example illustrates how advantage can be taken of the basic N-atom in the ligand backbone in case of sufficiently acid-stable products.

### Limitations

**Figure 10:**
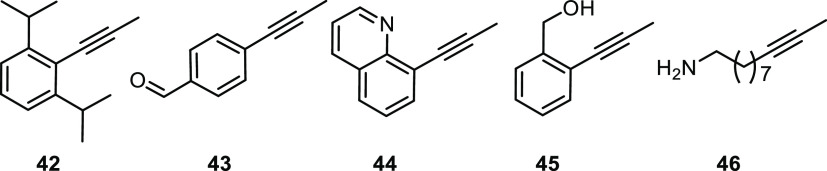
Unreactive substrates.

Because of the steric demand and a certain rigidity of the podand ligand framework, it is reasonable to expect that very bulky substrates will denote a limitation for the “canopy” catalysts **2** and the members of the new series (**15**-**17**) alike. In line with this notion, the inert nature of **42**, a pure hydrocarbon, suggests that binding and activation of the triple bond are precluded on steric grounds (Figure 10). Aldehydes such as **43** proved incompatible;^75^ the same is true for substrates with a heteroatom facing the alkyne (**44**) or poised to react with the ancillary ligands of the incoming catalyst (**45**), as well as for an alkyne carrying an unhindered primary amine (**46**).^76^

### Advanced Applications

**Scheme 8:**
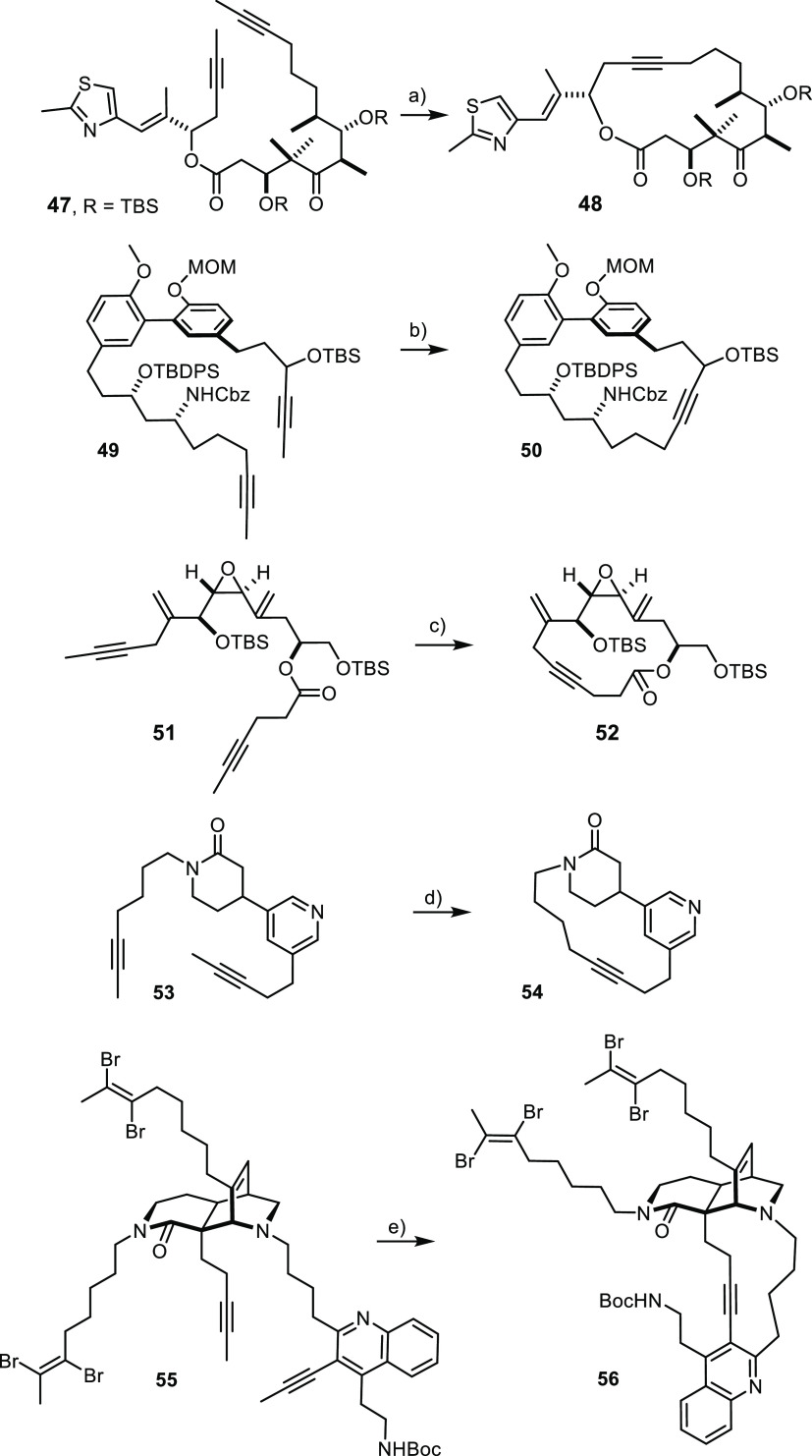
Applications to Advanced Intermediates of Previous Natural Product Syntheses; Note That Complex **16a** Was Weighed, Handled and Stored in Air **16a** (5 mol%), 60°C, toluene, MS 5Å, 81%. **16a** (5 mol%), 50°C, toluene, MS 5Å, 65%. **16a** (10 mol%), 60°C, toluene, MS 5Å, 94%. **16a** (30 mol%), 110°C, toluene, MS 5Å, 71%. **16a** (30 mol%), 110°C, toluene, MS 5Å, 81%.

For the final round of evaluation, we used a selection of advanced intermediates of previous natural product synthesis campaigns pursued in this laboratory (Scheme 8). It is pointed out that the catalyst loading was not optimized because the available amount of these precious substrates was limited. Anyway, cycloalkyne **48** as precursor for the anticancer agent epothilone C was readily formed,^62,77^ thus confirming the compatibility of the catalyst with a dense array of functional groups, including silyl ethers, an ester, a ketone and the aldol substructure associated with it, an olefin, and a thiazole nucleus. The list is further complemented by the acetal and carbamate present in **50**, which had previously served the total synthesis of the alkaloid lythranidine.^78^

Alkyne metathesis is orthogonal to alkene metathesis in that molybdenum alkylidynes leave all types of double bonds untouched. This favorable chemoselectivity already surfaced in the epothilone case (**48**), but is more prominently manifested in the high-yielding formation of **52**, which belongs to the amphidinolide V series:^79^ one of the two *exo*-methylene groups decorating the macrocycle is part of a vinyl-epoxide and hence of a quite reactive functional group, whereas the other one carries an allylic leaving group.

The notion that the compatibility of the catalysts with different Lewis basic groups is enabling from a synthesis perspective is arguably best illustrated by applications to alkaloids. Although **54**, the key precursor en route to the marine pyridinium alkaloid *epi*-tetradehydro-halicyclamine B, may not seem overly complex at first sight, its synthesis had borne witness for significant recent advances in chemoselectivity management beyond the range procurable by the classical repertoire.^80^ One of the critical aspects concerned the ability to effect macrocyclization in the presence of an unhindered pyridine, which was ultimately achieved by RCAM with the aid of a “canopy” catalyst **2**. The more user-friendly adduct **16a** has now been shown to be similarly effective.

The high-yielding formation of the polyfunctionalized product **56** comprising the basal macrocycle of nominal njaoamine I, a structurally complex cytotoxic marine alkaloid, is equally instructive.^81^ Although elevated temperatures were necessary for the reaction to proceed, the presence of a tertiary amine as well as a quinoline in the proximity of the reacting triple bonds arguably poses a challenge for any high-valent early transition metal reagent or catalyst. In terms of efficiency, the new air-stable adduct **16a** is on par with the molybdenum alkylidynes used in our original study.^81^ Equal performance combined with significantly improved user-friendliness marks an important advance in the field.

**Scheme 9:**
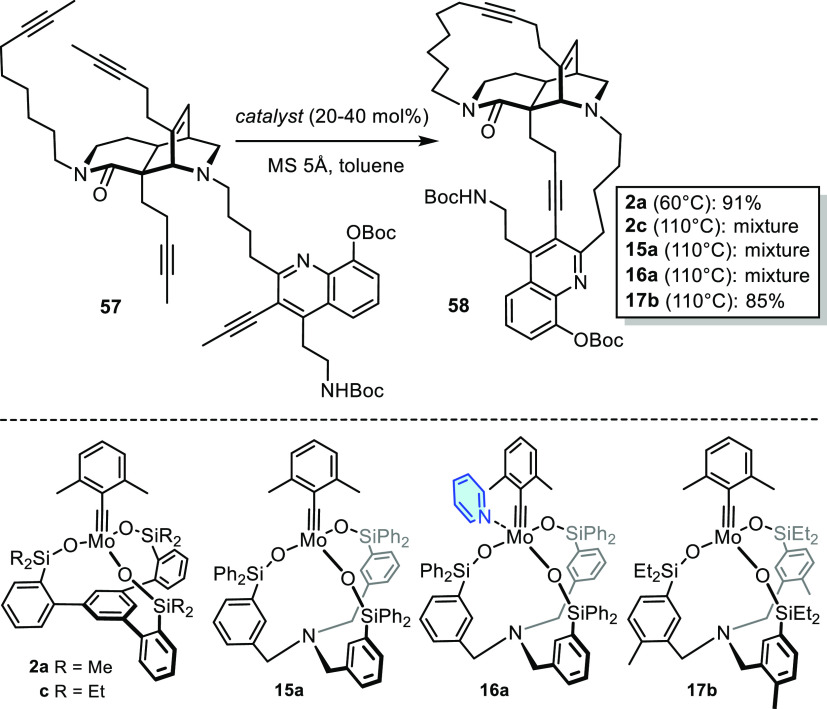
Concurrent Formation of Two Macrocycles

At first sight, the formation of product **58**, the key synthetic intermediate en route to njaoamine C as yet another member of this family of marine natural products, may seem to be a minor extrapolation (Scheme 9).^82^ Note, however, that both enveloping macrocycles are concurrently closed in this case; as the alkyne side arms branching off the core of tetra-yne **57** can, a priori, be connected in many different intra- and intermolecular ways, the formation of **58** in one pot is a particularly taxing transformation. When seen against this background, the result obtained with complex **2a** as the most active member of the “canopy series” is remarkable. In contrast, the only slightly more hindered sibling **2c** carrying ethyl- rather than methyl substituents on the silicon linkers furnished a complex mixture even under more forcing conditions.^81^ The new catalyst **15a** and the derived pyridine adduct **16a** led to the same disappointing outcome, whereas the somewhat more reactive ethyl variant **17b** (see Scheme 9) furnished product **58** in no less than 85% yield. These subtleties remind us that no single catalyst can serve all cases: although the coverage achieved with **15a** and the bench-stable adduct **16a** is very broad indeed, testing of structural variants is warranted when it comes to ensuring optimal results in highly advanced applications.

## Conclusions

The quest for alkyne metathesis catalysts truly apt for use in advanced organic synthesis has been ongoing in this and other laboratories for many years. In our case, the decisive milestones were the early deliberate departure from tungsten alkylidynes as the historic lead compounds and the discovery that molybdenum alkylidynes synergize very well with silanolates. For the sake of improved stability, the monodentate silanolates originally used were then evolved into a tripodal ligand set: the resulting “canopy” catalysts combine high activity with unparalleled selectivity and, therefore, arguably set the standard in the field.

The goal of the current project was to maintain these favorable attributes while improving the user-friendliness of the catalysts. Indeed, the new generation of molybdenum alkylidynes described herein arguably meets all critically important aspects: the pyridine adducts **16** are easy to make on scale, can be routinely weighed and handled in air, and can be stored for extended periods of time outside a glovebox. At the same time, they comply with the highest standards in terms of reactivity and selectivity; they embrace a host of polar and apolar functional groups and different protic sites as well as numerous basic functionalities. These chemical virtues are all the more noteworthy since the operative unit comprises an early transition metal in its highest oxidation state. For the favorable overall profile, we expect that adducts of type **16** will likely replace earlier catalyst generations in all but the most specialized cases; for their user-friendliness, they might well encourage even a nonexpert user to harness the power of alkyne metathesis, and, in doing so, foster more regular applications of this enabling transformation in organic synthesis and material science alike.

## Supporting Information Available

Experimental section including characterization data, VT-NMR studies, assessment of the air stability of the pyridine adducts, NMR spectra of new compounds (PDF)
Single crystal structure analysis (PDF)
