# Supplementary material for: From the Glovebox to the Benchtop: Air-Stable High Performance Molybdenum Alkylidyne Catalysts for Alkyne Metathesis
Source: J Am Chem Soc. 2023 Nov 30;145(49):26993–7009. doi: 10.1021/jacs.3c10430 (PMC10722517; doi:10.1021/jacs.3c10430)

SUPPORTING INFORMATION

From the Glovebox to the Benchtop: Air-Stable High Performance Molybdenum Alkylidyne Catalysts for Alkyne Metathesis

J. Nepomuk Korber, Christian Wille, Markus Leutzsch, and Alois Fürstner*

Max-Planck-Institut für Kohlenforschung, 45470 Mülheim an der Ruhr, Germany

*E-Mail: fuerstner@mpi-muelheim.mpg.de

Table of Contents

1 Experimental Part

General S1

Experimental Procedures S2
- Molybdenum Precursor Complexes S2
- The Ligands S5
- Complexes S12
- Adducts S15
- Benchmarking Experiments: Catalyst Activity S19
- Side Reaction: Polymerization S24
- Pyridine Scavenger Strategy S25
- Reactive Intermediates S26
- Stability S29
- VT-NMR Studies of Different Adducts S38
- Expedited Ligand Removal S41
- New Substrates S42
- Alkyne Metathesis Reactions S43

2 References **S55**

3 NMR Spectra of New Compounds
- Ligands S57
- Complexes S84
- Adducts S95
- New Substrates S107
- Byproduct S109

1 Experimental Part

General

Unless stated otherwise, all reactions were carried out under argon in flame-dried glassware following standard Schlenk techniques. The solvents were purified by distillation over the drying agents indicated and were transferred under argon: tetrahydrofuran (magnesium/anthracene), toluene ($NaAlEt_4$), benzene (CaH_2), dichloromethane (CaH_2), 1,2-dimethoxyethane (CaH_2), acetonitrile (CaH_2), diethyl ether (Na/K), and *n*-pentane (Na/K). Deuterated solvents were stored over molecular sieves (3 Å) for a minimum of five days prior to use.

The molecular sieves and Celite® used in this investigation were dried for 24 h at 150 °C (sand bath) under vacuum (10^{-3} mbar) prior to use and were stored and transferred under argon atmosphere.

Flash chromatography was carried out with Merck silica gel, type 9385 (230 – 400 mesh, 60 Å pore diameter).

All commercially available compounds (abcr, Lancaster, Aldrich, TCI) were used as received, unless stated otherwise. Ligand **7a** was prepared by Dr. L. Martínez-Rodríguez as described in the literature.[1] Substrates for alkyne metathesis reactions were prepared as described in the literature.[2-5] Starting materials for the intermediates of natural product syntheses were graciously provided by Dr. A. Dalling, Dr. C. Mathes, Dr. O. Larionov, Dr. K. Gebauer and Dr. S. Spohr.[6-10]

NMR: Spectra were acquired on Bruker AvanceIII 300, 400, 500 MHz or an Avance Neo 600 MHz NMR (equipped with a Bruker BBO CryoProbe) spectrometer in the solvents indicated; chemical shifts (δ) are given in ppm relative to tetramethylsilane (TMS), coupling constants (*J*) in Hz. The solvent signals were used as references and the chemical shifts converted to the TMS scale ($CDCl_3$: δ_C = 77.16 ppm; residual ^{1}H: δ_H = 7.26 ppm; CD_2Cl_2: δ_C = 53.84 ppm; residual ^{1}H: δ_H = 5.32 ppm; C_6D_6: δ_C = 128.06 ppm, residual ^{1}H: δ_H = 7.16 ppm; [D$_8$]-toluene: δ_C = 20.60 ppm; residual ^{1}H: δ_H = 2.09 ppm).[11] 1D ^{29}Si NMR spectra were acquired with a polarization transfer pulse sequence (refocussed INEPT) and broadband proton decoupling.

^{95}Mo NMR spectra were acquired with the aring pulse sequence to minimize acoustic ringing from the NMR probe. The π/2 pulse was calibrated for a 2 M Na_2MoO_4 in D_2O and had a typical length of 22.5 µs at a power of 85 W.

The ^{15}N chemical shifts of compounds **14b** and **15b** were determined with a 2D H(C)N multiple bond correlation experiment (Bruker sequence: hcnmbc)[12] acquired on a Bruker AVIII 600 MHz NMR equipped with a TCI cryoprobe.

^{29}Si, ^{15}N and ^{95}Mo chemical shifts were referenced indirectly to the ^{1}H chemical shift of the solvent according to IUPAC recommendations using the *xiref* macro in Bruker Topspin. ^{29}Si chemical shifts are reported relative to TMS (δ = 0 ppm; Ξ = 19.867187%), ^{15}N chemical shifts are reported relative to $MeNO_2$ (δ = 0 ppm; Ξ = 10.136767%), and ^{95}Mo chemical shifts relative to Na_2MO_4 (δ = 0 ppm; Ξ = 6.516926%).

IR: FT-IR spectrometer ALPHA (Bruker), wavenumbers ($\tilde{\nu}$) in cm^{-1}. Samples were prepared as a neat film or film by evaporation of a solution in $CDCl_3$ prior to direct application on the ATR unit.

MS (EI): Finnigan MAT 8200 (70 eV), **ESI-MS**: ESQ3000 (Bruker), accurate mass determinations: Bruker APEX III FT-MS (7 T magnet) or Mat 95 (Finnigan). All values are given in mass units per elementary charge (*m/z*).

Elemental analysis: H. Kolbe, Oberhausen.

Experimental Procedures

Molybdenum Precursor Complexes

Complex [Li·$(Et_2O)_{1/2}$][$Mo(CO)_5(COAr)$] (S1, Ar = 2,6-di(methyl)phenyl)

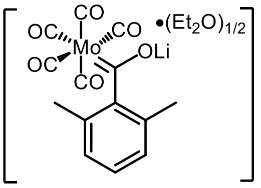

A 250 mL, three-necked, round bottom flask equipped with a 50 mL dropping funnel and connected to a vacuum/argon manifold was flame dried. The flask was charged with 2-bromo-1,3-dimethylbenzene (3.28 mL, 24.1 mmol) and diethyl ether (60 mL). The solution was cooled to −40 °C before a solution of *tert*-butyllithium (1.6 M in *n*-pentane, 30.1 mL, 48.3 mmol) was added *via* the dropping funnel over 10 min. Stirring was continued for 3 h at −20 °C.

A 500 mL, three-necked, round bottom flask equipped with a 100 mL dropping funnel and connected to a vacuum/argon manifold was flame-dried. The flask was charged with molybdenum hexacarbonyl (6.50 g, 24.1 mmol) and diethyl ether (60 mL), and the resulting solution was cooled to 0 °C. The solution of the lithiated arene was cooled to −78 °C, transferred with argon overpressure into the dropping funnel *via* a low density polyethylene (LDPE) tube, and then rapidly added to the cold solution of the molybdenum hexacarbonyl. Once the addition was complete, the resulting yellow-orange mixture was stirred at ambient temperature for 4 h. The dropping funnel was replaced by a glass stopper and a solvent trap was connected to the vacuum/argon manifold. The solvents and all volatile materials were evaporated under reduced pressure (10^{-3} mbar). The resulting orange solid was suspended in dichloromethane (4 × 20 mL) and filtered through an argon filter frit packed with a Celite® pad mounted onto a flame dried, 250 mL, three-necked flask connected to a vacuum/argon manifold. The resulting filtrate was concentrated *in vacuo* (10^{-3} mbar) until ca. 10 mL of the solution remained.

A flame dried, 1 L, three-necked flask equipped with an argon manifold was charged with *n*-pentane (700 mL) and the concentrated solution was added dropwise with vigorous stirring. The product precipitated as a microcrystalline solid, which was collected by filtration using an argon filter frit mounted onto a flame dried, 2 L two-necked flask connected to a vacuum/argon manifold. The product was washed with *n*-pentane (3 × 20 mL) and dried under high vacuum (10^{-3} mbar) to give the desired complex as an diethyl ether adduct (2:1 ratio). Pale yellow microcrystalline solid material (8.76 g, 95%). **^{1}H NMR** (600 MHz, CD_2Cl_2): δ 6.99 – 6.95 (m, 1H), 6.91 (d, *J* = 7.6 Hz, 2H), 2.98 (q, *J* = 7.0 Hz, 3H), 2.23 (s, 6H), 0.69 (t, *J* = 7.0 Hz, 2H). **^{13}C NMR** (151 MHz, CD_2Cl_2): δ 353.1, 213.5, 208.8, 201.2, 159.6, 128.4, 126.5, 126.5, 65.9, 18.5, 13.4. **IR** (film): $\tilde{\nu}$ 3392, 1974, 1965, 1935, 1904, 1852, 1830, 1619, 1594, 1561, 1449,

1379, 1172, 860, 773, 699, 647, 588, 565, 508, 458. **HRMS** (ESI) calc. for $C_{14}H_9O_6MoLi$ [M − Et_2O − Li]$^-$: 370.94587; found: 370.94613.

The spectroscopic data was found to be in agreement with the data reported in the literature.[13]

[Mo(≡CAr)Br_3(dme)] (S2, Ar = 2,6-di(methyl)phenyl)

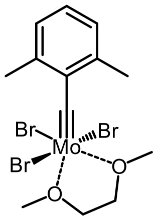

A 500 mL, three-necked, round bottom flask equipped with an internal thermometer and connected to a vacuum/argon manifold was flame dried. The flask was charged with [Li·$(Et_2O)_{1/2}$][Mo$(CO)_5$(COAr)] (**S1**) (4.37 g, 10.6 mmol) and dichloromethane (60 mL). The resulting orange mixture was cooled to −78 °C and stirred for 15 min before a solution of oxalyl bromide (1.09 mL, 11.6 mmol) in dichloromethane (5 mL) was added dropwise over 5 min, causing a color change to purple. Once the addition was complete, stirring was continued for 15 min at −78 °C. The mixture was then allowed to warm to −40 °C. Upon reaching this temperature a rapid color change to light brown was observed and the mixture was quickly cooled to −78 °C again. Stirring was discontinued to allow most of the precipitate to settle.

In parallel, a 1 L, three-necked, jacketed cooling flask equipped with a 25 mL pressure-equalizing dropping funnel and a jacketed cooling argon filter frit (diameter: 3 cm; height: 30 cm; porosity: 3) containing a pad of Celite® (15 cm) was connected to a vacuum/argon manifold and flame dried. The Celite® in the jacketed filter frit was slurried with dichloromethane and packed tightly before the frit was cooled to −78 °C using a cryostat. This flask was charged with 1, 2-dimethoxyethane (5.50 mL, 52.9 mmol), which was also cooled to −78 °C.

The cold light brown reaction mixture was transferred with argon overpressure *via* a LDPE tube in portions to the frit. [*Great care must be taken during this step in order to prevent the filter frit from clogging. Transferring the mixture onto the frit in small portions and regularly repacking the Celite® pad is recommended*]. Upon contact of the bright yellow filtrate with the 1,2-dimethoxyethane in the 1 L receiving flask, a color change to dark red was observed. The mixture was stirred for 10 min at –78 °C before a solution of bromine (0.51 mL, 10.0 mmol) in dichloromethane (5 mL) was added dropwise over a period of 15 min *via* the dropping funnel. Once the addition was complete, the mixture was stirred for 15 min at –78 °C. The resulting brown-orange mixture was allowed to reach ambient temperature over the course of 1 h, during which time the color intensifies. Stirring was continued for 1 h at ambient temperature before the dropping funnel and the filter frit were replaced by glass stoppers. The brown reaction mixture was filtered through an argon filter frit with a Celite® pad mounted onto a flame dried, 500 mL, two-necked flask connected to a vacuum/argon manifold. The frit was replaced by a stopper and the solvents were removed from the filtrate *in vacuo* to give the title complex as a rust colored solid (2.30 g, 41%). Impurities could be removed by washing the solid with diethyl ether. **^{1}H NMR** (400 MHz, CD_2Cl_2): δ 7.09 (d, J = 7.7 Hz, 2H), 6.79 (t, J = 7.6 Hz, 1H), 4.03 (s, 4H), 3.95 (s, 3H), 3.88 (s, 3H), 3.24 (s, 6H).

The spectroscopic data was found to be in agreement with the data reported in the literature.[14]

[Mo(≡CAr)(O*t*Bu)$_3$] (9, Ar = 2,6-di(methyl)phenyl)

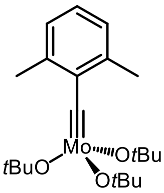

A 250 mL Schlenk flask was charged with [Mo(≡CAr)Br$_3$(dme)] (Ar = 2,6-di(methyl)phenyl) **S2** (2.05 g, 3.79 mmol,) and tetrahydrofuran (23 mL). A solution of sodium *tert*-butoxide (1.07 g, 11.2 mmol) in tetrahydrofuran (7 mL) was added dropwise *via* syringe at ambient temperature and stirring was continued overnight before the solvent was removed *in vacuo* to obtain a dark brown solid material. The residue was suspended in *n*-pentane (4 x 20 mL) and the suspension was filtered through a Celite® pad in an argon filter frit mounted onto a 250 mL, two-necked flask connected to a vacuum/argon manifold. The filter frit was replaced by a stopper and the filtrate was concentrated *in vacuo* (10^{-3} mbar) until a total volume of ca. 4 mL remained. The solution was cooled to −40 °C, causing crystallization of the title complex over a course of 4 h. Removal of the supernatant and drying of the residue under high vacuum (10^{-3} mbar) gave the desired complex as a brown powder (1.31 g, 3.04 mmol, 80%). **^{1}H NMR** (400 MHz, C_6D_6): δ 6.90 (dd, *J* = 7.3, 0.8 Hz, 2H), 6.80 (dd, *J* = 8.2, 6.8 Hz, 1H), 2.86 (s, 6H), 1.44 (s, 27H).

The spectroscopic data was found to be in agreement with the data reported in the literature.[15]

The Ligands

Compound S3

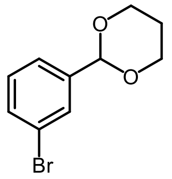

A 100 mL, two-necked round bottom flask equipped with a Dean-Stark apparatus (reflux condenser and distilling trap filled with toluene) and connected to a vacuum/argon manifold was charged with 5-bromobenzaldehyde (6.85 g, 37.0 mmol), toluene (50 mL) and propan-1,3-diol (2.94 mL, 40.7 mmol). *p*-Toluenesulfonic acid monohydrate (211 mg, 1.11 mmol) was added and the solution was stirred at reflux temperature overnight. After cooling to ambient temperature, the organic layer was washed with an aqueous solution of sodium bicarbonate (2 × 50 mL) and brine (1 × 50 mL). The organic phase was dried over magnesium sulfate and filtered, and the solvents were evaporated. The residue was purified by flash chromatography on silica (hexane/ethyl acetate, 19:1 to 9:1) to give the title compound as a yellow liquid (7.25 g, 81%). **^{1}H NMR** (400 MHz, $CDCl_3$): δ 7.67 (t, *J* = 1.8 Hz, 1H), 7.50 – 7.36 (m, 2H), 7.23 (t, *J* = 7.8 Hz, 1H), 5.46 (s, 1H), 4.26 (ddd, *J* = 12.0, 5.0, 1.5 Hz, 2H), 4.03 – 3.90 (m, 2H), 2.21 (dtt, *J* = 13.6, 12.4, 5.0 Hz, 1H), 1.45 (ddt, *J* = 13.6, 2.6, 1.3 Hz, 1H).

The spectroscopic data was found to be in agreement with the data reported in the literature.[16]

Compound 12a

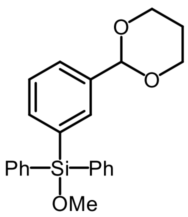

A 100 mL Schlenk flask was charged with compound **S3** (7.25 g, 29.8 mmol) and tetrahydrofuran (30 mL). The solution was cooled to 0 °C before a solution of isopropylmagesium chloride lithium chloride complex (1.25 M in tetrahydrofuran, 9.18 mL, 11.9 mmol) was added dropwise. After stirring for 5 min, a solution of *n*-butyllithium (1.6 M in hexane, 14.6 mL, 23.9 mmol) was added dropwise and the resulting grey suspension was stirred for 1 h at 0 °C.

A 250 mL, two-necked round bottom flask equipped with a pressure equilibrating dropping funnel and connected to a vacuum/argon manifold was charged with dimethoxydiphenylsilane (13.5 mL, 59.6 mmol) and tetrahydrofuran (25 mL). The resulting solution was cooled to 0 °C. The Grignard reagent was transferred into the dropping funnel with a LDPE tube and argon overpressure and then slowly added to the dimethoxydiphenylsilane solution at 0°C. Once the addition was complete, the mixture was allowed to reach ambient temperature and stirring was continued overnight. The reaction was carefully quenched by the addition of an saturated aqueous solution of ammonium chloride (100 mL). The layers were separated and the aqueous phase was extracted with ethyl acetate (3 × 75 mL). The combined organic layers were dried over magnesium sulfate and filtered, and the solvents were evaporated. The residue was purified by flash chromatography on silica (hexane/ethyl acetate, 9:1) to give the title compound as a colorless oil (9.58 g, 85%). **^{1}H NMR** (400 MHz, $CDCl_3$): δ 7.76 (ddt, *J* = 1.7, 1.1, 0.6 Hz, 1H), 7.67 – 7.61 (m, 6H), 7.49 – 7.36 (m, 7H), 5.50 (s, 1H), 4.26 (ddt, *J* = 10.5, 5.1, 1.5 Hz, 2H), 4.02 – 3.91 (m, 2H), 3.64 (s, 3H), 2.23 (dtt, *J* = 13.5, 12.4, 5.0 Hz, 1H), 1.43 (dtt, *J* = 13.5, 2.7, 1.4 Hz, 1H). **^{13}C NMR** (101 MHz, $CDCl_3$): δ 138.3, 136.1, 135.61, 135.56, 134.0, 133.9, 133.1, 130.2, 128.1, 128.01, 127.99, 127.9, 101.9, 67.5, 52.0, 25.9. **^{29}Si NMR** (60 MHz, $CDCl_3$): δ −11.3. **IR** (film): $\tilde{\nu}$ 2965, 1835, 1737, 1589, 1428, 1371, 1237, 1102, 1081, 995, 770, 738, 698, 504. **HRMS** (EI) calc. for $C_{23}H_{24}O_3SiNa$ $[M + Na]^+$: 399.13869; found: 399.13871.

Compound 13a

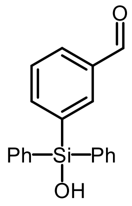

A 500 mL, one-neck round bottom flask equipped with a 100 mL dropping funnel was charged in air with compound **12a** (9.58 g, 24.53 mmol) and tetrahydrofuran (245 mL). The solution was cooled to 0 °C before hydrochloric acid (3 M in water, 245 mL) was added *via* the dropping funnel over 10 min. The mixture was warmed to ambient temperature and stirred for 1 h. The solution was carefully neutralized with an aqueous solution of sodium bicarbonate and extracted with dichloromethane (3 × 150 mL). The combined organic layers were dried over sodium sulfate and filtered, and the solvents were evaporated. The residue was purified by flash chromatography on silica (toluene/ethyl acetate, 10:1) to give the title compound as a colorless solid (6.88 g, 92%). **^{1}H NMR** (400 MHz, $CDCl_3$): δ 9.97 (s, 1H), 8.18 – 8.12 (m, 1H), 7.92 (ddt, *J* = 17.4, 7.3, 1.4 Hz, 2H), 7.66 – 7.60 (m, 4H), 7.58 – 7.52 (m, 1H), 7.51 – 7.44 (m, 2H), 7.44 – 7.37 (m, 4H), 2.83 (bs, 1H). **^{13}C NMR** (101 MHz, $CDCl_3$): δ 192.9, 141.1, 137.00, 136.96, 135.8, 135.1, 134.4, 130.9, 130.6, 128.7, 128.3. **^{29}Si NMR** (60 MHz, $CDCl_3$): δ −13.4. **IR** (film): $\tilde{\nu}$ 3414 (b), 3070, 3051, 1736, 1696, 1587, 1428, 1372, 1240, 1114, 1044, 862, 697, 645, 505, 457. **HRMS** (ESI) calc. for $C_{19}H_{16}O_2SiNa$ $[M + Na]^+$: 327.08118; found: 327.08071.

Ligand 14a

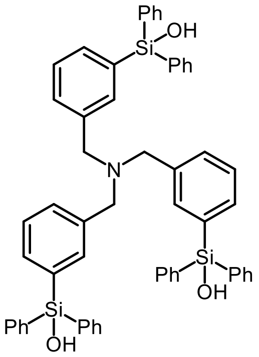

A 500 mL, two-necked round bottom flask connected to a vacuum/argon manifold was charged with compound **13a** (8.20 g, 26.9 mmol), tetrahydrofuran (270 mL) and ammonium acetate (642 mg, 8.33 mmol). Sodium trisacetoxyborohydride (9.10 g, 42.9 mmol) was added in portions and the resulting suspension was stirred for 24 h. The solution was neutralized with an aqueous solution of sodium bicarbonate and extracted with ethyl acetate (3 × 200 mL). The combined organic layers were dried over magnesium sulfate and filtered, and the solvents were evaporated. The residue was purified by flash chromatography on silica (dichloromethane/methanol, 99:1 to 98:2) to give a mixture of siloxane dimers/oligomers and ligand **14a** as a colorless powder.

A 500 mL, one-necked flask was charged in air with this crude material and tetrahydrofuran (400 mL). An aqueous solution of sodium hydroxide (2 M, 400 mL) was added and the mixture was stirred overnight. The layers were separated and the aqueous layer was extracted with ethyl acetate (3 × 300 mL). The combined organic phases were washed with brine (300 mL), dried over magnesium sulfate and filtered, and the solvents were evaporated to give the title compound as a colorless powder (4.75 g, 60%). **^{1}H NMR** (600 MHz, $CDCl_3$): δ 8.29 (t, *J* = 1.5 Hz, 3H), 7.51 – 7.45 (m, 12H), 7.39 – 7.32 (m, 9H), 7.29 (dt, *J* = 7.7, 1.6 Hz, 3H), 7.22 (dddd, *J* = 7.5, 6.5, 2.4, 0.7 Hz, 15H), 4.74 (s, 3H), 3.61 (s, 6H). **^{13}C NMR** (151 MHz, $CDCl_3$): δ 139.9, 135.4, 135.3, 135.1, 135.0, 133.5, 130.5, 130.0, 128.0, 127.6, 57.8. **^{29}Si NMR** (119 MHz, $CDCl_3$): δ −13.5. **IR** (film): $\tilde{\nu}$ 3290 (b), 3048, 1428, 1117, 828, 791, 740, 712, 699, 541, 509. **HRMS** (ESI) calc. for $C_{57}H_{50}NO_3Si_3$ $[M - H]^-$: 880.31041; found: 880.31091.

Compound S4

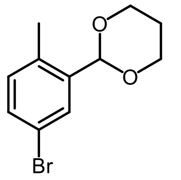

A 250 mL, two-necked round bottom flask equipped with a Dean-Stark apparatus (reflux condenser and distilling trap which was filled with toluene) and an argon manifold was charged with 5-bromo-2-methylbenzaldehyde (**11b**, 15.6 g, 78.4 mmol), toluene (100 mL), and propan-1,3-diol (6.23 mL, 86.2 mmol). *p*-Toluenesulfonic acid monohydrate (447 mg, 2.35 mmol) was added and the solution was stirred at reflux temperature overnight. After cooling to ambient temperature, the organic layer was washed with an aqueous solution of sodium bicarbonate (2 × 75 mL) and brine (1 × 100 mL). The organic layer was dried over magnesium sulfate and filtered, and the solvents were evaporated. The residue was purified by flash chromatography on silica (hexane/ethyl acetate, 9:1) to give the title compound as a yellow liquid (14.9 g, 72%). **^{1}H NMR** (400 MHz, $CDCl_3$): δ 7.74 (d, *J* = 2.2 Hz, 1H), 7.34 (dd, *J* = 8.1, 2.3 Hz, 1H), 7.01 (dd, *J* = 8.1, 0.8 Hz, 1H), 5.55 (s, 1H), 4.26 (ddt, *J* = 10.5, 5.1, 1.4 Hz, 2H), 4.03 – 3.92 (m, 2H), 2.33 (s, 3H), 2.29 – 2.15 (m, 1H), 1.45 (dtt, *J* = 13.6, 2.6, 1.4 Hz, 1H). **^{13}C NMR** (101 MHz, $CDCl_3$): δ 138.6, 134.7, 132.1, 131.6, 129.2, 119.7, 99.2, 67.6, 25.8, 18.4. **IR** (film): $\tilde{\nu}$ 2965, 2851, 1468, 1376, 1235, 1148, 1097, 1009, 989, 877, 855, 810, 615, 550, 476. **HRMS** (EI) calc. for $C_{11}H_{13}O_2Br$ $[M]^-$: 256.00954; found: 256.00935.

Compound 12b

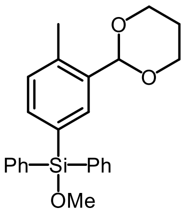

A 100 mL Schlenk flask was charged with compound **S4** (14.9 g, 56.7 mmol) and tetrahydrofuran (20 mL). The solution was cooled to 0 °C before a solution of isopropylmagesium chloride lithium chloride complex (1.25 M in tetrahydrofuran 18.5 mL, 23.1 mmol) was added dropwise. After stirring for 5 min, a solution of *n*-butyllithium (1.6 M in hexane, 28.4 mL, 46.3 mmol) was added dropwise and the resulting grey suspension was stirred for 1 h at 0 °C.

A 250 mL, two-necked flask equipped with a pressure equilibrating dropping funnel and connected to a vacuum/argon manifold was charged with dimethoxydiphenylsilane (26.2 mL, 116 mmol) and tetrahydrofuran (50 mL). The resulting solution was cooled to 0 °C. The Grignard reagent was transferred into the dropping funnel with a LDPE tube and argon overpressure and was then slowly added to the cooled dimethoxydiphenylsilane solution. Once the addition was complete, the mixture was allowed to warm to ambient temperature and stirring was continued overnight. The reaction was carefully quenched with a saturated aqueous solution of ammonium chloride (50 mL). The layers were separated and the aqueous phase was extracted with ethyl acetate (3 × 30 mL). The combined organic layers were dried over magnesium sulfate and filtered, and the solvents were evaporated. The residue was purified by flash chromatography on silica (hexane/ethyl acetate, 19:1) to give the title compound as a colorless oil (15.8 g, 70%). **^{1}H NMR** (400 MHz, $CDCl_3$): δ 7.88 (s, 1H), 7.70 – 7.59 (m, 4H), 7.50 – 7.36 (m, 7H), 7.21 (dt, *J* = 7.4, 0.7 Hz, 1H), 5.60 (s, 1H), 4.26 (ddt, *J* = 10.5, 5.1, 1.4 Hz, 2H), 4.03 – 3.92 (m, 2H),, 3.65 (s, 3H), 2.48 (s, 3H), 2.24 (dtt, *J* = 13.5, 12.5, 5.0 Hz, 1H), 1.42 (dtt, *J* = 13.4, 2.6, 1.4 Hz, 1H). **^{13}C NMR** (101 MHz, $CDCl_3$): δ 138.4, 136.2, 136.0, 135.5, 134.3, 133.2, 130.7, 130.4, 130.0, 127.9, 101.2, 67.6, 51.9, 25.8, 19.3. **^{29}Si NMR** (60 MHz, $CDCl_3$): δ −11.3. **IR** (film): $\tilde{\nu}$ 3068, 2963, 2934, 2836, 1602, 1428, 1377, 1236, 1149, 1107, 1088, 997, 768, 739, 700, 568, 501. **HRMS** (EI) calc. for $C_{24}H_{26}O_3Si$ $[M]^-$: 390.16457; found: 390.16510.

Compound 13b

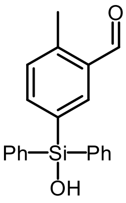

A 500 mL, one-neck flask equipped with a 100 mL dropping funnel was charged in air with compound **12b** (8.72 g, 22.3 mmol) and tetrahydrofuran (220 mL). The solution was cooled to 0 °C and hydrochloric acid (6.0 M in water, 57 mL, 0.34 mol) was added *via* the dropping funnel over 10 min. The mixture was warmed to ambient temperature and stirred for 1 h. The solution was carefully neutralized with a saturated aqueous solution of sodium bicarbonate and the aqueous layer was extracted with dichloromethane (3 × 100 mL). The combined organic phases were dried over sodium sulfate and filtered, and the solvents were evaporated. The residue was purified by flash chromatography on silica (toluene/ethyl acetate, 14:1) to give the title compound as a colorless liquid (6.68 g, 94%). **^{1}H NMR** (400 MHz, $CDCl_3$): δ 10.14 (s, 1H), 8.06 (d, *J* = 1.4 Hz, 1H), 7.72 (dd, *J* = 7.5, 1.4 Hz, 1H), 7.67 – 7.57 (m, 4H), 7.49 – 7.43 (m, 2H), 7.42 – 7.36 (m, 4H), 7.31 – 7.26 (m, 1H), 3.22 (bs, 1H), 2.67 (s, 3H). **^{13}C NMR** (101 MHz, $CDCl_3$): δ 193.7, 142.6, 140.2, 139.6, 135.1, 134.7, 133.8, 133.6, 131.6, 130.5, 128.2, 20.1. **^{29}Si NMR** (60 MHz, $CDCl_3$): δ −13.39.
IR (film): $\tilde{\nu}$ 3367 (b), 3049, 1696, 1680, 1592, 1551, 1428, 1201, 1114, 901, 848, 829, 740, 699, 636, 547, 503. **HRMS** (EI) calc. for $C_{20}H_{18}O_2SiNa$ $[M + Na]^+$: 341.09704; found: 341.09683.

Ligand 14b

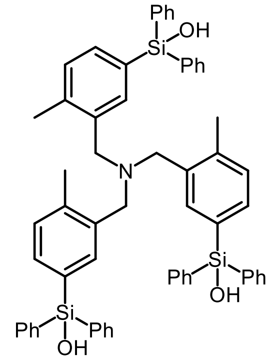

A 250 mL, two-necked round bottom flask connected to a vacuum/argon manifold was charged with compound **13b** (5.00 g, 15.7 mmol), tetrahydrofuran (160 mL) and ammonium acetate (390 mg, 5.07 mmol). Sodium trisacetoxyborohydride (4.99 g, 23.6 mmol) was added in portions and the resulting suspension was stirred for 24 h. The solution was neutralized with an aqueous solution of sodium bicarbonate and extracted with ethyl acetate (3 × 75 mL). The combined organic layers were dried over magnesium sulfate and filtered, and the solvents were evaporated. The residue was purified by flash chromatography on silica (dichloromethane/methanol, 50:1 to 5:1) to give a mixture of siloxane dimers/oligomers and ligand **14b** as a colorless powder.[1]
A 100 mL, one-necked flask was charged in air with this crude material and tetrahydrofuran (80 mL). An aqueous solution of sodium hydroxide (2 M, 80 mL) was added and the mixture was stirred overnight. The layers were separated and the aqueous phase was extracted with ethyl acetate (3 × 40 mL). The combined organic layers were washed with brine (1 × 50 mL), dried over magnesium sulfate and filtered, and the solvents were evaporated to give the title compound as a white powder (3.65 g, 65%). **^{1}H NMR** (600 MHz, $CDCl_3$): δ 8.44 (s, 3H), 7.46 – 7.42 (m, 12H), 7.34 – 7.30 (m, 6H), 7.19 (dd, *J* = 7.5, 1.3 Hz, 3H), 7.21 – 7.15 (m, 12H), 7.06 (d, *J* = 7.5 Hz, 3H), 4.74 (s, 3H), 3.66 (s, 6H), 2.25 (s, 9H). **^{13}C NMR** (151 MHz, $CDCl_3$): δ 138.3, 137.7, 135.5, 135.0, 135.0, 133.3, 132.4, 129.9, 129.6, 127.9, 55.1, 19.5. **^{29}Si NMR** (119 MHz, $CDCl_3$): δ −13.6. **^{15}N NMR** ([2D ^{1}H-^{15}N HMBC], 61 MHz, $CDCl_3$): δ −335. **IR** (film): $\tilde{\nu}$ 3303 (b), 3069, 3049, 2922, 1598, 1428, 1193, 1113, 906, 818, 728, 711, 696, 643, 560, 494. **HRMS** (ESI) calc. for $C_{60}H_{56}NO_3Si_3$ $[M - H]^-$: 922.35735; found: 922.35784.

[1] The amount of siloxane dimer formed varied between different batches.

Compound S5

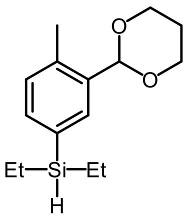

A 50 mL Schlenk flask was charged with compound **S4** (4.00 g, 15.6 mmol) and tetrahydrofuran (30 mL). The solution was cooled to 0 °C and a solution of isopropylmagesium chloride lithium chloride complex (1.25 M in tetrahydrofuran, 4.78 mL, 6.22 mmol) was added dropwise. After stirring for 5 min, a solution of *n*-butyllithium (1.6 M in hexane, 7.78 mL, 12.4 mmol) was added dropwise and the resulting grey suspension was stirred for 1 h at 0 °C.

A 250 mL, two necked round bottom flask equipped with a pressure equilibrating dropping funnel and connected to a vacuum/argon manifold was charged with Et_2SiH_2 (6.01 mL, 46.7 mmol) and tetrahydrofuran (20 mL). The resulting solution was cooled to 0 °C. The Grignard reagent was transferred into the dropping funnel with a LDPE tube and argon overpressure and then slowly added to the cooled diethylsilane solution. Once the addition was complete, the mixture was allowed to warm to ambient temperature and stirring was continued overnight. The reaction was carefully quenched with an aqueous, saturated solution of ammonium chloride (40 mL). The layers were separated and the aqueous phase was extracted with ethyl acetate (3 × 50 mL). The combined organic layers were dried over magnesium sulfate and filtered, and the solvents were evaporated. The residue was purified by flash chromatography on silica (hexane/ethyl acetate, 12:1) to give the title compound as a colorless oil (3.88 g, 94%). **^{1}H NMR** (400 MHz, $CDCl_3$): δ 7.73 (d, *J* = 1.3 Hz, 1H), 7.39 (dd, *J* = 7.4, 1.4 Hz, 1H), 7.15 (d, *J* = 7.4 Hz, 1H), 5.61 (s, 1H), 4.29 (ddt, *J* = 10.5, 5.0, 1.4 Hz, 2H), 4.20 (p, *J* = 3.4 Hz, 1H), 4.02 – 3.96 (m, 2H), 2.41 (s, 3H), 2.27 (qt, *J* = 13.5, 5.0 Hz, 1H), 1.45 (dtt, *J* = 13.4, 2.6, 1.4 Hz, 1H), 1.01 (t, *J* = 7.8 Hz, 6H), 0.84 (td, *J* = 8.3, 3.3 Hz, 4H). **^{13}C NMR** (101 MHz, $CDCl_3$): δ 137.1, 136.0, 135.2, 132.6, 132.3, 130.1, 100.7, 67.7, 25.9, 18.0, 8.3, 3.7. **^{29}Si NMR** (60 MHz, $CDCl_3$): δ −5.1. **IR** (film): $\tilde{\nu}$ 2954, 2872, 2102, 1377, 1235, 1149, 1106, 998, 967895, 801, 712, 687, 478. **HRMS** (EI) calc. for $C_{15}H_{24}O_2Si$ $[M]^+$: 264.15401; found: 264.15419.

Compound S6

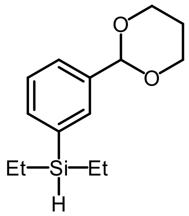

Prepared analogously from **S3** (4.00 g, 16.5 mmol) as a colorless liquid (2.89 g, 70%). **^{1}H NMR** (400 MHz, $CDCl_3$): δ 7.68 – 7.64 (m, 1H), 7.53 – 7.51 (m, 2H), 7.38 (td, *J* = 7.5, 0.6 Hz, 1H), 5.52 (s, 1H), 4.29 (ddt, *J* = 10.5, 5.0, 1.4 Hz, 2H), 4.24 (p, *J* = 3.3 Hz, 1H), 4.03 – 3.97 (m, 2H), 2.25 (dtt, *J* = 13.5, 12.4, 5.0 Hz, 1H), 1.45 (dtt, *J* = 13.5, 2.7, 1.4 Hz, 1H), 1.06 – 0.99 (m, 6H), 0.92 – 0.81 (m, 4H). **^{13}C NMR** (101 MHz, $CDCl_3$): δ 138.1, 135.7, 135.2, 132.3, 127.8, 127.1, 101.9, 67.5, 25.9, 8.2, 3.5. **^{29}Si NMR** (60 MHz, $CDCl_3$): δ −4.7. **IR** (film): $\tilde{\nu}$ 2955, 2913, 2873, 2851, 2106, 1373, 1237, 1147, 1106, 1016, 998, 969, 810, 787, 707. **HRMS** (ESI) calc. for $C_{14}H_{22}O_2SiNa$ $[M + Na]^+$: 273.12823; found: 273.12808.

Compound S7

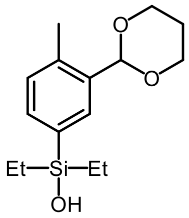

A 50 mL, one-necked round bottom flask was charged in air with compound **S5** (3.87 g, 14.7 mmol) and dichloromethane (30 mL). The solution was cooled to 0 °C before *m*-chloroperoxybenzoic acid (77% *w/w*, 3.62 g, 16.1 mmol) was added in portions. After stirring at ambient temperature for 5 h, the mixture was transferred into a separation funnel and was washed with a saturated aqueous solution of sodium bicarbonate (2 × 60 mL) and aqueous sodium hydroxide (1 M, 60 mL). The organic layer was dried over magnesium

sulfate and filtered, and the solvents were evaporated to give the title compound as a colorless liquid (3.84 g, 92%). **^{1}H NMR** (400 MHz, $CDCl_3$): δ 7.76 (d, *J* = 1.3 Hz, 1H), 7.42 (dd, *J* = 7.4, 1.4 Hz, 1H), 7.16 (dt, *J* = 7.4, 0.7 Hz, 1H), 5.61 (s, 1H), 4.28 (ddt, *J* = 10.4, 5.0, 1.4 Hz, 2H), 3.99 (dddd, *J* = 12.6, 10.5, 2.6, 1.5 Hz, 2H), 2.41 (d, *J* = 0.6 Hz, 3H), 2.36 – 2.17 (m, 1H), 1.74 (bs, 1H), 1.45 (dtt, *J* = 13.5, 2.6, 1.3 Hz, 1H), 1.03 – 0.94 (m, 6H), 0.91 – 0.76 (m, 4H). **^{13}C NMR** (101 MHz, $CDCl_3$): δ 137.5, 136.0, 134.4, 134.1, 131.1, 130.2, 100.7, 67.7, 25.9, 19.1, 6.7, 6.5. **IR** (film): $\tilde{\nu}$ 3433 (b), 2956, 2874, 1460, 1378, 1236, 1149, 1107, 1010, 897, 821, 719, 480. **HRMS** (EI) calc. for $C_{15}H_{24}O_3Si$ $[M]^+$: 280.14892; found: 280.14896.

Compound S8

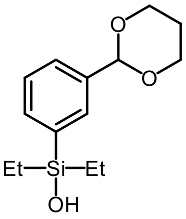

Prepared analogously from compound **S6** (2.89 g, 11.5 mmol) as a colorless liquid (2.84 g, 93%). **^{1}H NMR** (600 MHz, $CDCl_3$): δ 7.69 – 7.66 (m, 1H), 7.54 – 7.50 (m, 2H), 7.39 – 7.35 (m, 1H), 5.50 (s, 1H), 4.26 (ddt, *J* = 10.5, 5.0, 1.4 Hz, 2H), 4.01 – 3.94 (m, 2H), 2.29 – 2.14 (m, 2H), 1.48 – 1.40 (m, 1H), 1.01 – 0.93 (m, 6H), 0.85 – 0.80 (m, 4H). **^{13}C NMR** (151 MHz, $CDCl_3$): δ 138.0, 137.4, 134.2, 131.2, 127.8, 127.4, 102.0, 67.5, 25.9, 6.7, 6.5. **^{29}Si NMR** (60 MHz, $CDCl_3$): δ 8.2. **IR** (film): $\tilde{\nu}$ 3419 (b), 2955, 2146, 1413, 1237, 1146, 1118, 996, 837, 790, 720, 615, 472, 443. **HRMS** (EI) calc. for $C_{14}H_{22}O_3Si$ $[M]^+$: 266.13327; found: 266.13357.

Compound S9

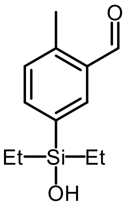

A 250 mL, one-neck round bottom flask equipped with a 50 mL dropping funnel was charged in air with compound **S7** (3.87 g, 13.8 mmol) and tetrahydrofuran (120 mL). The solution was cooled to 0 °C before hydrochloric acid (12 M in water, 17.0 mL, 207 mmol) was added *via* the dropping funnel over 10 min. After 20 min at 0 °C, the solution was carefully neutralized with a saturated aqueous solution of sodium bicarbonate and the aqueous phase was extracted with dichloromethane (3 × 100 mL). The combined organic layers were dried over sodium sulfate and filtered, and the solvents were evaporated to give the title compound as a colorless liquid (2.72 g, 89%). Compound **S9** is prone to siloxane formation and was immediately used in the subsequent step. **^{1}H NMR** (400 MHz, $CDCl_3$): δ 10.29 (s, 1H), 7.98 (d, *J* = 1.4 Hz, 1H), 7.68 (dd, *J* = 7.5, 1.4 Hz, 1H), 7.28 (d, *J* = 7.5 Hz, 1H), 2.68 (d, *J* = 0.7 Hz, 3H), 1.94 (s (br), 1H), 1.05 – 0.96 (m, 6H), 0.93 – 0.82 (m, 4H). **^{13}C NMR** (101 MHz, $CDCl_3$): δ 193.4, 142.1, 138.9, 137.8, 135.4, 133.8, 131.5, 19.9, 6.7, 6.6. **IR** (film): $\tilde{\nu}$ 3390, 2956, 2876, 1698, 1684, 1594, 1202, 1110, 1091, 1008, 899, 828, 725. **HRMS** (ESI) calc. for $C_{12}H_{18}O_2SiNa$ $[M + Na]^+$: 245.09683; found: 245.09693.

Compound S10

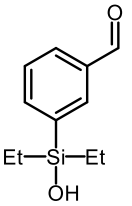

Prepared analogously from compound **S8** (500 mg, 1.88 mmol) as a colorless liquid (3.22 g, 82%). This compound is prone to siloxane formation and was immediately used in the next step of the ligand synthesis. **^{1}H NMR** (600 MHz, CD_2Cl_2): δ 10.02 (d, *J* = 0.4 Hz, 1H), 8.09 – 8.06 (m, 1H), 7.89 (ddd, *J* = 7.6, 1.8, 1.3 Hz, 1H), 7.85 (dt, *J* = 7.3, 1.3 Hz, 1H), 7.57 – 7.52 (m, 1H), 1.07 (s (br), 1 H), 1.01 – 0.97 (m, 6H), 0.91 – 0.85 (m, 4H). **^{13}C NMR** (151 MHz, CD_2Cl_2): δ 193.2, 140.0, 139.4, 136.1, 135.5, 130.7, 128.8, 6.8, 6.7. **^{29}Si NMR** (119 MHz, CD_2Cl_2): δ 7.5. **IR** (film): $\tilde{\nu}$ 3392, 2956, 2876, 1686, 2586, 2415, 1371, 1208, 1270, 1107, 1007, 961, 894, 836, 791, 720, 693, 652, 615, 436. **HRMS** (EI) calc. for $C_{11}H_{16}O_2Si$ $[M]^+$: 208.09141; found: 208.09143.

Ligand S11

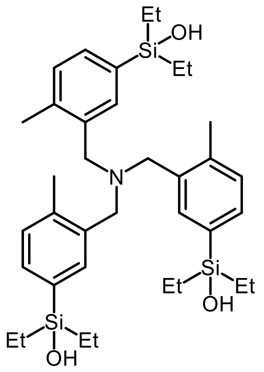

A 250 mL, two-necked round bottom flask connected to a vacuum/argon manifold was charged with compound **S9** (2.72 g, 12.2 mmol), tetrahydrofuran (100 mL) and ammonium acetate (304 mg, 3.95 mmol). Sodium trisacetoxyborohydride (3.81 g, 18.0 mmol) was added in portions and the resulting suspension was stirred for 24 h. Additional sodium triacetoxyborohydride (1.00 g, 4.72 mmol) was then added and stirring continued for an additional 48 h. For work up, the solution was cooled to 0 °C and carefully neutralized with an aqueous solution of sodium bicarbonate. The mixture was concentrated *via* rotary evaporation until 50 mL remained and was then extracted with *tert*-butyl methyl ether (3 × 50 mL). The combined organic layers were washed with an aqueous solution of sodium hydroxide (1 M, 40 mL), dried over magnesium sulfate and filtered, and the solvents were evaporated. The residue was purified by flash chromatography on silica (hexane/ethyl acetate, 5:1 to 4:1) to give the title compound as a colorless powder (873 mg, 35%). **^{1}H NMR** (600 MHz, $CDCl_3$): δ 8.31 (s, 3H), 7.20 (dd, *J* = 7.4, 1.0 Hz, 3H), 7.08 (d, *J* = 7.4 Hz, 3H), 4.28 (s, 3H), 3.64 (s, 6H), 2.25 (s, 9H), 0.87 (t, *J* = 7.9 Hz, 18H), 0.71 (m, 12H). **^{13}C NMR** (151 MHz, $CDCl_3$): δ 137.7, 137.3, 134.7, 134.1, 131.3, 129.4, 55.0, 19.4, 6.6, 6.7. **^{29}Si NMR** (119 MHz, $CDCl_3$): δ 7.5. ^{15}**N NMR** ([2D ^{1}H-^{15}N HMBC], 61 MHz, $CDCl_3$): δ −338. **IR** (film): $\tilde{\nu}$ 3312 (b), 2955, 2912, 2876, 2801, 1460, 1359, 1236, 1086, 1009, 967, 819, 727. **HRMS** (ESI) calc. for $C_{36}H_{56}NO_3Si_3$ $[M - H]^-$: 634.35736; found: 634.35773.

Ligand S12

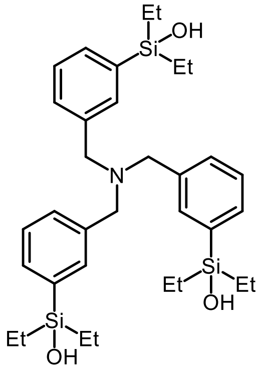

Prepared analogously from compound **S10** (3.47 g, 15.3 mmol) as a colorless oil (960 mg, 33%). **^{1}H NMR** (600 MHz, $CDCl_3$): δ 8.18 (d, *J* = 1.4 Hz, 3H), 7.31 – 7.27 (m, 3H), 7.24 – 7.22 (m, 6H), 4.33 (s(br), 3H), 3.57 (s, 6H), 0.96 (t, *J* = 7.9 Hz, 18H), 0.84 – 0.74 (m, 12H). **^{13}C NMR** (151 MHz, $CDCl_3$): δ 139.9, 137.9, 134.3, 131.6, 129.7, 127.3, 57.7, 6.80, 6.75. **^{29}Si NMR** (119 MHz, $CDCl_3$): δ 7.5. **IR** (film): $\tilde{\nu}$ 3292, 2955, 2876, 1459, 1410, 1236, 1114, 1008, 970, 826, 788, 724, 616, 443. **HRMS** (ESI) calc. for $C_{33}H_{52}NO_3Si_3$ $[M + H]^+$: 594.32496; found: 594.32459.

Complexes

Complex 10

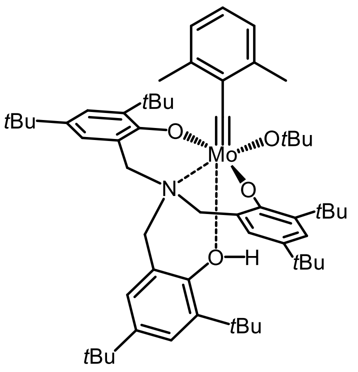

A 100 mL Schlenk flask was charged with ligand **7a** (310 mg, 0.46 mmol) which was azeotropically dried with benzene (2 × 3 mL) to remove residual water. Toluene (35 mL) was added and a solution of the alkylidyne complex **9** (200 mg, 0.46 mmol) in toluene (6 mL) was added dropwise to the solution. After stirring for 5 h, all volatile materials were removed *in vacuo* to give the title complex as a brown/red powder (420 mg, 94%). Red crystals suitable for single-crystal X-ray diffraction were grown from a concentrated solution in benzene. **^{1}H NMR** (600 MHz, C_6D_6): δ 7.48 (d, *J* = 2.4 Hz, 2H), 7.00 (d, *J* = 2.4 Hz, 1H), 6.97 (d, *J* = 2.5 Hz, 2H), 6.70 (d, *J* = 7.4 Hz, 2H), 6.64 (dd, *J* = 8.2, 6.7 Hz, 1H), 6.28 (s, 1H), 5.47 (s, 1H), 5.01 (d, *J* = 13.5 Hz, 2H), 3.31 (s, 2H), 3.17 (d, *J* = 13.6 Hz, 2H), 2.48 (s, 6H), 1.69 (s, 9H), 1.54 (s, 18H), 1.44 (s, 18H), 1.24 (s, 9H), 1.09 (s, 9H). **^{13}C NMR** (151 MHz, C_6D_6): δ 292.7, 160.2, 149.9, 143.6, 142.7, 142.5, 138.9, 137.9, 137.2, 133.0, 129.3, 128.6, 128.2, 128.1, 127.9, 125.7, 124.4, 124.1, 123.9, 123.7, 120.4, 81.5, 69.5, 58.4, 35.5, 34.3, 34.1, 33.9, 33.2, 32.2, 31.8, 31.0, 30.8, 21.4, 20.3. ^{15}**N NMR** ([2D ^{1}H-^{15}N HMBC], 61 MHz, C_6D_6): δ −324.[2] **^{95}Mo NMR** (26 MHz, C_6D_6): δ 349. **IR** (neat): $\tilde{\nu}$ 2951, 2900, 2867, 1477, 1444, 1414, 1359, 1286, 1264, 1238, 1204, 1169, 1149, 947, 915, 870, 841, 774, 751, 732, 695, 588, 574, 547, 527, 483, 466. **HRMS** (ESI) calc. for $C_{58}H_{85}MoNO_4Na$ [M + Na]$^+$: 980.54249; found: 980.54168.

Complex 15b

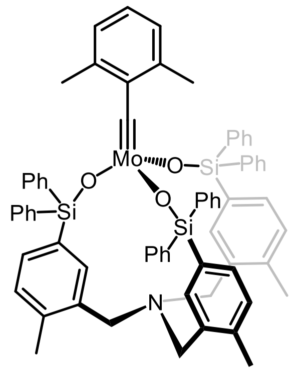

A 250 mL Schlenk flask was charged with ligand **14b** (855 mg, 0.93 mmol), which was azeotropically dried with benzene (2 × 4 mL) to remove any residual water. The compound was suspended in toluene (90 mL) and a solution of the alkylidyne complex **9** (400 mg, 0.93 mmol) in toluene (10 mL) was added dropwise. After stirring for 1.5 h, all volatile materials were removed *in vacuo* to give the title complex as a yellow powder (1.04 g, 98%). Orange crystals suitable for single-crystal X-ray diffraction were grown from a concentrated solution in benzene. **^{1}H NMR** (600 MHz, [D$_8$]-toluene): δ 8.84 (s, 3H), 7.69 (m, 12H), 7.34 (dd, J = 7.5, 1.0 Hz, 3H), 7.14 (tt, J = 7.1, 1.4 Hz, 6H), 7.04 (t, J = 7.6 Hz, 12H), 6.93 (d, J = 7.5 Hz, 3H), 6.55 (m, 3H), 3.45 (s, 6H), 2.13 (s, 6H), 2.06 (s, 9H). **^{13}C NMR** (151 MHz, [D$_8$]-toluene): δ 309.6, 145.6, 139.3, 138.3, 137.6, 136.5, 135.5, 134.6, 133.8, 133.2, 129.95, 129.89, 128.1, 127.9, 126.3, 54.4, 20.5, 19.4. **^{29}Si NMR** (119 MHz, [D$_8$]-toluene): δ −10.1. ^{15}**N NMR** ([2D ^{1}H-^{15}N HMBC], 61 MHz, [D$_8$]-toluene): δ −333. **^{95}Mo NMR** (26 MHz, [D$_8$]-toluene, 60 °C): δ 495. **IR** (neat): $\tilde{\nu}$ 1427, 1192, 1113, 1094, 1023, 926, 892, 816, 740, 730, 695, 648, 561, 495, 422. **HRMS** (ESI) calc. for $C_{69}H_{63}MoNO_3Si_3Na$ [M + Na]$^+$: 1158.30620; found: 1158.30562. **Elemental analysis** (%) calc. for $C_{69}H_{63}MoNO_3Si_3$: C 73.05, H 5.60, Mo 8.46, Si 7.43; found: C 72.85, H 5.54, Mo 8.28, Si 7.23.

---

[2] The corresponding ^{15}N NMR shift of the free ligand **7a** is −329 ppm

Complex 15a

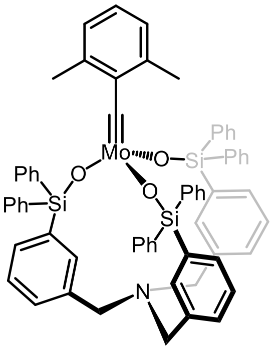

A 50 mL Schlenk flask was charged with ligand **14a** (204 mg, 0.23 mmol), which was azeotropically dried with benzene (3 × 1.5 mL) to remove residual water. The compound was suspended in toluene (30 mL) and a solution of the alkylidyne complex **9** (100 mg, 0.23 mmol) in toluene (8 mL) was added dropwise. After stirring for 3.5 h, all volatile materials were removed *in vacuo* to give the title complex as a yellow powder (238 mg, 94%). **^{1}H NMR** (600 MHz, [D$_8$]-toluene): δ 8.76 (s, 3H), 7.71 – 7.62 (m, 12H), 7.40 (dt, *J* = 7.4, 1.3 Hz, 3H), 7.20 – 7.12 (m, 6H), 7.07 (td, *J* = 7.4, 0.6 Hz, 3H), 7.05 – 6.99 (m, 15H), 6.58 – 6.54 (m, 2H), 6.54 – 6.50 (m, 1H), 3.39 (s, 6H), 2.09 (s, 6H). **^{13}C NMR** (151 MHz, [D$_8$]-toluene): δ 309.6, 145.7, 140.6, 139.4, 136.8, 136.4, 135.6, 135.0, 133.7, 130.6, 130.0, 128.1, 127.7, 126.4, 128.11, 58.1. 20.6. **^{29}Si NMR** (119 MHz, [D$_8$]-toluene): δ −10.3. **^{95}Mo NMR** (26 MHz, [D$_8$]-toluene, 60 °C): δ 499. **IR** (neat): $\tilde{\nu}$ 3045, 1428, 1406, 1114, 1020, 981, 915, 770, 741, 697, 540, 509, 488, 452. **HRMS** (ESI) calc. for $C_{66}H_{58}MoNO_3Si_3$ $[M + H]^+$: 1094.27731; found: 1094.27686.

Complex 17a

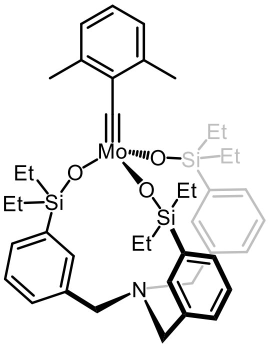

A 100 mL Schlenk flask was charged with ligand **S12** (339 mg, 0.52 mmol, 90% purity), which was azeotropically dried with benzene (2 × 2 mL) to remove residual water. The compound was dissolved in toluene (50 mL) and a solution of the akylidyne complex **9** (222 mg, 0.51 mmol) in toluene (6 mL) was added dropwise. After stirring for 1 h, all volatile materials were removed *in vacuo* to give a brown residue. This residue was dissolved in dichloromethane (3 mL) and an excess of pentane (50 mL) was added. The title complex precipitated from the solution after 3 d at –78 °C as a yellow powder and was collected by filtration (336 mg, 81%). **^{1}H NMR** (600 MHz, [D$_8$]-toluene): δ 8.45 (s, 3H), 7.26 (dt, *J* = 7.3, 1.3 Hz, 3H), 7.15 (td, *J* = 7.4, 0.6 Hz, 3H), 7.03 – 6.99 (m, 3H), 6.85 (d, *J* = 7.5 Hz, 2H), 6.72 (t, *J* = 7.5 Hz, 1H), 3.39 (s, 6H), 2.85 (s, 6H), 1.06 – 1.00 (m, 18H), 1.01 – 0.93 (m, 6H), 0.92 – 0.84 (m, 6H). **^{13}C NMR** (151 MHz, [D$_8$]-toluene): δ 305.7, 145.8, 140.4, 138.6, 138.0, 134.6, 131.7, 130.0, 127.6, 126.9, 127.8, 58.1, 20.8, 8.4, 7.1. **^{29}Si NMR** (119 MHz, [D$_8$]-toluene): δ 12.1. **^{95}Mo NMR** (26 MHz, [D$_8$]-toluene, 60 °C): δ 454. **IR** (neat): $\tilde{\nu}$ 2952, 2873, 1457, 1407, 1233, 1113, 981, 868, 768, 723, 665, 450, 420. **HRMS** (ESI) calc. for $C_{42}H_{58}MoNO_3Si_3$ $[M + H]^+$: 806.27731; found: 806.27657.

Complex 17b

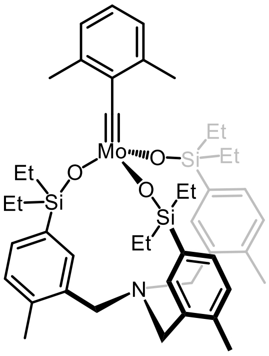

A 100 mL Schlenk flask was charged with ligand **S11** (300 mg, 0.47 mmol), which was azeotropically dried with benzene (2 × 2 mL) to remove residual water. The compound was suspended in toluene (45 mL) and a solution of the alkylidyne complex **9** (204 mg, 0.47 mmol) in toluene (8 mL) was added dropwise. After stirring for 3.5 h, all volatile materials were removed *in vacuo* to give a brown residue. The crude material was repeatedly triturated with pentane and then dried under high vacuum to give the toluene-free complex **17b** as a yellow powder (399 mg, 99%). **^{1}H NMR** (600 MHz, [D$_8$]-toluene): δ 8.60 (s, 3H), 7.22 (dd, *J* = 7.3, 1.1 Hz, 3H), 7.01 (d, *J* = 7.3 Hz, 3H), 6.88 – 6.84 (m, 2H), 6.72 (t, *J* = 7.5 Hz, 1H), 3.52

(s, 6H), 2.85 (s, 6H), 2.07 (s, 9H), 1.04 – 1.00 (m, 18H), 1.00 – 0.91 (m, 6H), 0.92 – 0.82 (m, 6H). **^{13}C NMR** (151 MHz, [D$_8$]-toluene): δ 305.7, 145.8, 138.5, 138.1, 137.0, 135.1, 134.1, 131.3, 129.8, 127.6, 126.9, 54.7, 20.8, 19.4, 8.3, 7.1. **^{29}Si NMR** (119 MHz, [D$_8$]-toluene): δ 12.2. **^{95}Mo NMR** (26 MHz, [D$_8$]-toluene, 60 °C): δ 451. **IR** (neat): $\tilde{\nu}$ 2951, 2910, 2873, 1457, 1374, 1233, 1193, 1094, 1013, 910, 892, 816, 765, 726, 705, 679, 550, 427. **HRMS** (ESI) calc. for $C_{45}H_{64}MoNO_3Si_3$ [M + H]$^+$: 848.32426; found: 848.32437. **Elemental analysis** (%) calc. for $C_{45}H_{63}MoNO_3Si_3$: C 63.87, H 7.50, N 1.66, Mo 11.34, Si 9.96; found: C 62.46, H 7.65, N 1.59, Mo 10.87, Si 9.11.

Adducts

Adduct 16a

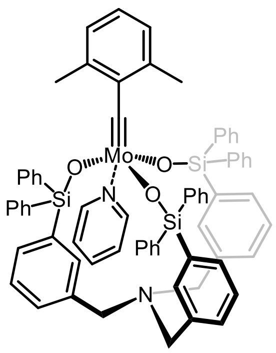

A 25 mL Schlenk flask was charged with complex **15a** (835 mg, 0.76 mol) and dichloromethane (15 mL). Pyridine (80 µL, 0.99 mmol) was added to the orange solution, causing an immediate color change to dark purple. After stirring for 1 h, all volatile materials were removed *in vacuo* to give a purple powder, which was washed with pentane (2 × 6 mL) and dried in vacuum to yield the title adduct (787 mg, 88%). Purple crystals suitable for single-crystal X-ray diffraction were grown from a dichloromethane/pentane solution by slow evaporation of the solvents. **^{1}H NMR** (600 MHz, [D$_8$]-toluene, 253 K): δ 9.68 (s, 1H), 9.02 (s, 2H), 7.99 (dd, *J* = 6.3, 1.4 Hz, 2H), 7.95 – 7.90 (m, 4H), 7.69 (d, *J* = 7.3 Hz, 1H), 7.55 – 7.51 (m, 4H), 7.48 (dt, *J* = 7.3, 1.3 Hz, 2H), 7.23 (t, *J* = 7.4 Hz, 1H), 7.20 – 7.13 (m, 8H), 7.10 – 7.05 (m, 5H), 6.99 (t, *J* = 7.8 Hz, 4H), 6.93 – 6.85 (m, 6H), 6.82 (d, *J* = 7.8 Hz, 2H), 6.75 – 6.70 (m, 5H), 6.04 (tt, *J* = 7.7, 1.6 Hz, 1H), 5.40 – 5.34 (m, 2H), 3.61 (d, *J* = 13.8 Hz, 2H), 3.53 (d, *J* = 13.8 Hz, 2H), 3.36 (s, 2H), 2.72 (s, 6H). **^{13}C NMR** (151 MHz, [D$_8$]-toluene, 253 K): δ 301.3, 150.8, 143.0, 142.8, 141.0, 140.0, 138.9, 138.6, 138.4, 137.4, 137.2, 137.0, 136.6, 135.6, 135.5, 134.4, 134.0, 133.8, 130.2, 129.3, 129.2, 129.0, 128.9, 128.5, 127.9, 127.7, 127.38, 127.36, 127.3, 124.4, 26.8, 58.2, 57.7, 21.2. **^{29}Si NMR** (119 MHz, [D$_8$]-toluene, 253 K): δ −18.2, −18.0. **^{15}N NMR** ([2D ^{1}H-^{15}N HMBC], 61 MHz, [D$_8$]-toluene, 253 K): δ −113.9 (N-pyridine); signal of benzylamine not detected. **IR** (film): $\tilde{\nu}$ 3044, 1605, 1446, 1427, 1404, 1361, 1108, 1033, 1024, 1011, 997, 922, 855, 773, 740, 696, 637, 540, 506, 486, 445, 426. **Elemental analysis** (%) calc. for $C_{71}H_{62}MoN_2O_3Si_3$: C 72.79, H 5.33, N 2.39; found: C 72.42, H 5.58, N 2.34.

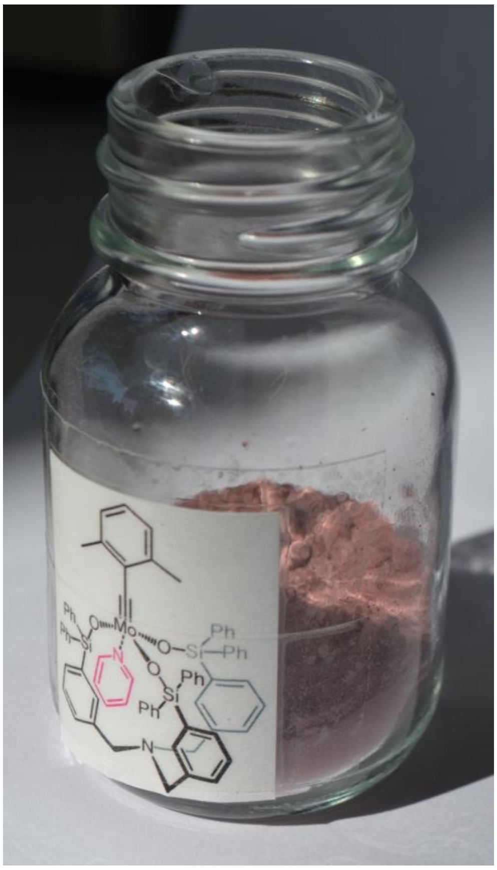

Figure S1. Photograph of a sample of the bench-stable adduct **16a** in powder form

Adduct 16b

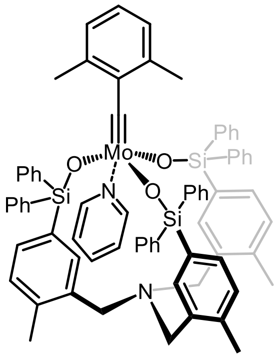

A 10 mL Schlenk flask was charged with complex **15b** (98 mg, 87 µmol) and dichloromethane (2 mL). Pyridine (11 µL, 0.13 mmol) was added to the orange solution, causing an immediate color change to dark purple. After stirring for 2 h, all volatile materials were removed *in vacuo* to give a purple powder which was washed with pentane (3 × 1.5 mL) and dried in vacuum to yield adduct **16b** (81 mg, 77%). Purple crystals suitable for single-crystal X-ray diffraction were grown from a dichloromethane/pentane solution by slow evaporation of the solvents. **^{1}H NMR** (600 MHz, [D_8]-toluene/CD_2Cl_2, 253 K): δ 9.61 (s, 1H), 8.93 (s, 2H), 7.92 – 7.81 (m, 2H), 7.78 – 7.63 (m, 4H), 7.45 (d, *J* = 7.4 Hz, 1H), 7.38 – 7.31 (m, 4H), 7.15 (d, *J* = 7.9 Hz, 2H), 7.09 (dt, *J* = 7.7, 1.6 Hz, 2H), 7.05 – 6.99 (m, 3H), 6.96 – 6.87 (m, 6H), 6.84 (t, *J* = 7.7 Hz, 6H), 6.73 – 6.68 (m, 6H), 6.65 (dd, *J* = 8.1, 7.0 Hz, 1H), 6.37 (tt, *J* = 7.7, 1.6 Hz, 1H), 5.78 – 5.62 (m, 2H), 3.68 (d, *J* = 14.9 Hz, 2H), 3.58 (d, *J* = 14.8 Hz, 2H), 3.47 (s, 2H), 2.47 (s, 6H), 2.11 (s, 6H), 2.10 (s, 3H). **^{13}C NMR** (151 MHz, [D_8]-toluene/CD_2Cl_2, 253 K): δ 301.1, 151.1, 142.72, 142.67, 138.7, 138.63, 138.60, 137.3, 137.21, 137.16, 137.1, 136.5, 135.7, 135.42, 135.43, 135.39, 135.38, 134.5, 133.7, 133.3, 132.7, 129.52, 129.48, 129.1, 128.8, 128.6, 128.5, 127.7, 127.5, 127.3, 126.6, 124.3, 20.9, 55.0, 54.7, 19.7, 19.2. **^{29}Si NMR** (119 MHz, [D_8]-toluene/CD_2Cl_2, 253 K): δ −18.8, −17.7. ^{15}**N NMR** ([2D ^{1}H-^{15}N HMBC], 61 MHz, [D_8]-toluene/CD_2Cl_2, 253 K): δ −113.2 (N-pyridine); signal of benzylamine not detected. **IR** (neat): $\tilde{\nu}$ 1427, 1110, 1024, 997, 926, 88, 817, 742, 694, 645, 560, 485, 421. **HRMS** (ESI) calc. for $C_{74}H_{68}MoN_2O_3Si_3$ $[M]^+$: 1214.35863; found: 1214.35929.

3-Bromopyridine Adduct 16c

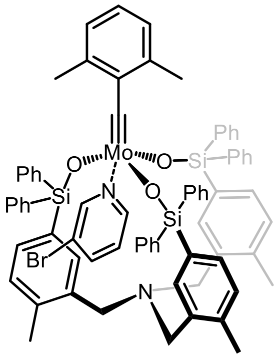

A 10 mL Schlenk flask was charged with complex **15b** (49 mg, 43 µmol) and dichloromethane (1.5 mL). 3-Bromopyridine (7 µL, 73 µmol) was added to the orange solution, causing an immediate color change to dark purple. After 15 min, pentane (7 mL) was added and the solution was cooled to −78 °C. The supernatant solution was separated from the precipitate by transfer into a new Schlenk flask using a filter-capped LDPE tube and argon overpressure. The solvents were removed in a constant stream of argon. The resulting purple solid was washed with pentane (3 × 1.5 mL) at 0 °C and dried in a gentle stream of argon to give the title adduct (48 mg, 85%). **^{1}H NMR** (600 MHz, [D_8]-toluene, 233 K): δ 9.76 (s, 1H), 9.11 (s, 2H), 8.09 (s, 1H), 8.01 (d, *J* = 5.4 Hz, 1H), 7.94 – 7.90 (m, 4H), 7.60 (d, *J* = 7.7 Hz, 1H), 7.59 (dd, *J* = 8.2, 1.4 Hz, 4H), 7.37 (d, *J* = 7.4 Hz, 2H), 7.27 – 7.22 (m, 4H), 7.19 – 7.10 (m, 4H), 7.09 (d, *J* = 7.5 Hz, 1H), 7.03 (t, *J* = 7.6 Hz, 4H), 6.99 – 6.93 (m, 8H), 6.84 (t, *J* = 7.6 Hz, 4H), 6.76 (d, *J* = 7.7 Hz, 2H), 6.71 – 6.66 (m, 1H), 6.13 (dt, *J* = 8.2, 2.3 Hz, 1H), 5.20 (dd, *J* = 8.2, 5.4 Hz, 1H), 3.70 (d, *J* = 15.0 Hz, 2H), 3.54 (d, *J* = 15.0 Hz, 2H), 3.42 (s, 2H), 2.59 (s, 6H), 2.09 (s, 6H), 2.08 (s, 3H). **^{13}C NMR** (151 MHz, [D_8]-toluene, 233 K): δ 301.9, 151.9, 150.0, 143.0, 142.3, 140.2, 139.7, 138.6, 138.5, 138.4, 137.2, 137.1, 136.4, 135.9, 135.53, 135.47, 135.4, 135.0, 134.6, 133.5, 133.4, 133.0, 129.7, 129.6, 129.3, 129.0, 128.7, 127.8, 127.8, 127.7, 127.3, 126.7, 125.2, 125.1, 124.9, 120.6, 54.83, 54.77, 21.0, 19.7, 19.3. **^{29}Si NMR** (119 MHz, [D_8]-toluene, 233 K): δ −17.3, −17.1. ^{15}**N NMR** ([2D ^{1}H-^{15}N HMBC], 61 MHz, [D_8]-toluene, 233 K): δ −108.0 (N-pyridine); signal of benzylamine not detected.

3,5-Dibromo-pyridine Adduct 16d

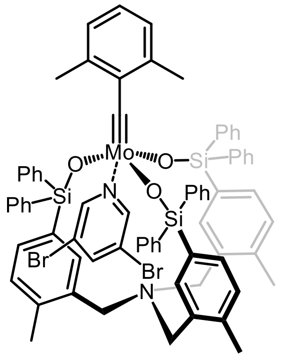

A 10 mL Schlenk flask was charged with complex **15b** (75 mg, 66 µmol) and dichloromethane (1.5 mL). 3,5-Dibromopyridine (23 mg, 99 µmol) was added to the orange solution, causing an immediate color change to dark red. After stirring for 1 h, pentane (5 mL) was added. The supernatant solution was separated from the precipitate by transfer into a new Schlenk flask using a filter-capped LDPE tube and argon overpressure. The solvents were removed in a constant stream of argon. The resulting purple solid was washed with pentane (3 × 2 mL) at 0 °C and dried in a gentle stream of argon to give the title adduct (75 mg, 83%). **^{1}H NMR** (600 MHz, [D$_8$]-toluene, 228 K): δ 9.70 (s, 1H), 9.08 (s, 2H), 8.20 (s, 2H), 7.91 (d, *J* = 6.9 Hz, 4H), 7.64 – 7.54 (m, 6H), 7.39 (d, *J* = 7.2 Hz, 2H), 7.34 (d, *J* = 6.8 Hz, 4H), 7.18 – 7.11 (m, 4H), 7.09 (d, *J* = 7.2 Hz, 1H), 7.05 – 7.01 (m, 4H), 7.99 – 6.96 (m, 7H), 6.92 (t, *J* = 7.0 Hz, 4H), 6.72 (d, *J* = 7.6 Hz, 2H), 6.67 – 6.58 (m, 1H), 6.29 (s, 1H), 3.69 (d, *J* = 14.8 Hz, 2H), 3.52 (d, *J* = 14.8 Hz, 2H), 3.40 (s, 2H), 2.54 (s, 6H), 2.10 (s, 6H), 2.08 (s, 3H). **^{13}C NMR** (151 MHz, [D$_8$]-toluene, 228 K): δ 302.7, 150.8, 143.1, 141.9, 141.8, 138.5, 138.21, 138.20, 137.5, 137.1, 136.9, 136.5, 135.9, 135.4, 135.3, 135.2, 134.5, 134.4, 133.363, 133.360, 133.4, 133.1, 129.8, 129.6, 129.3, 129.1, 129.0, 128.7, 127.9, 127.9, 127.7, 127.3, 126.7, 120.8, 54.8, 54.7, 20.8, 19.7, 19.4. **^{29}Si NMR** (119 MHz, [D$_8$]-toluene, 228 K): δ −16.6, −17.0.

4-Pyrrolidinopyridine Adduct 16e

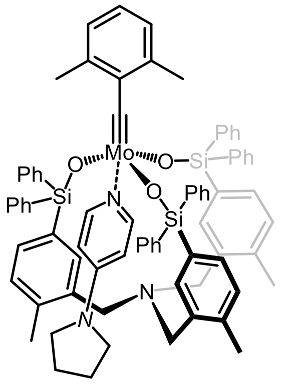

A 10 mL Schlenk flask was charged with complex **15b** (76 mg, 67 µmol) and dichloromethane (1.5 mL). 4-Pyrrolidino-pyridine (15 mg, 0.1 mmol) was added to the orange solution, causing an immediate color change to dark purple. After stirring for 3 h, all volatile materials were removed *in* vacuo. The purple powder was washed with pentane (2 × 2 mL) to give the title adduct (71 mg, 55 µmol, 83%) containing traces of free 4-pyrrolidino-pyridine and hydrolyzed ligand. The purity of the sample was only ca. 93% (NMR). **^{1}H NMR** (600 MHz, [D$_8$]-toluene, 273 K): δ 9.80 (s, 1H), 9.16 (s, 2H), 7.94 (dd, *J* = 8.1, 1.4 Hz, 4H), 7.66 (d, *J* = 6.8 Hz, 2H), 7.64 – 7.58 (m, 5H), 7.41 (dd, *J* = 7.2, 1.0 Hz, 2H), 7.35 (dd, *J* = 8.0, 1.4 Hz, 4H), 7.15 – 7.08 (m, 2H), 7.08 (d, *J* = 7.5 Hz, 3H), 7.03 (td, *J* = 7.1, 1.7 Hz, 6H), 7.01 – 6.96 (m, 4H), 6.84 – 6.80 (m, 4H), 6.78 – 6.74 (m, 4H), 6.74 – 6.71 (m, 1H), 4.74 – 4.69 (m, 2H), 3.74 (d, *J* = 14.5 Hz, 2H), 3.63 (d, *J* = 14.5 Hz, 2H), 3.51 (s, 2H), 2.72 (s, 6H), 2.21 – 2.18 (m, 4H), 2.15 (s, 6H), 2.12 (s, 3H), 1.26 – 1.23 (m, 4H). **^{13}C NMR** (151 MHz, [D$_8$]-toluene, 273 K): δ 298.8, 161.1, 150.7, 143.0, 142.9, 139.5, 138.7, 138.6, 137.2, 136.9, 136.4, 136.21, 136.17, 136.1, 136.0, 135.8, 135.2, 134.6, 133.7, 133.0, 129.53, 129.46, 129.0, 128.0, 127.9, 127.6, 126.9, 126.6, 128.3, 106.4, 55.1, 54.6, 46.4, 25.1, 21.2, 19.8, 19.3. **^{29}Si NMR** (119 MHz, [D$_8$]-toluene, 273 K): δ −19.4, −18.6. **^{15}N NMR** ([2D ^{1}H-^{15}N HMBC], 61 MHz, [D$_8$]-toluene, 273 K): δ −290.6 (N-pyrrolidinyl), −147.7 (N-pyridine); signal of benzylamine not detected.

Trimethylphosphine Adduct 16f

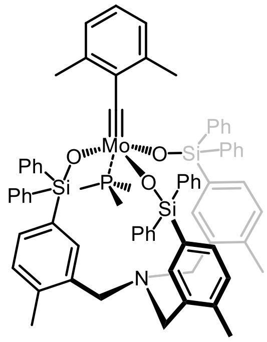

A 10 mL Schlenk flask was charged with complex **15b** (45 mg, 40 µmol) and dichloromethane (1.5 mL). Trimethylphosphine (9 µL, 80 µmol) was added, causing an immediate color change from orange to dark purple. After stirring for 1 h, all volatile materials were removed *in* vacuo. The purple powder was washed with pentane (3 × 1.5 mL) to give the title adduct (44 mg, 40 µmol, 92%). **^{1}H NMR** (600 MHz, [D$_8$]-toluene, 233 K): δ (s, 1H), 9.25 (s, 2H), 7.83 – 7.78 (m, 4H), 7.78 – 7.73 (m, 4H), 7.74 – 7.70 (m, 4H), 7.57 – 7.52 (m, 3H), 7.13 – 7.09 (m, 4H), 7.09 – 7.02 (m, 6H), 7.04 – 6.98 (m, 7H), 6.90 (t, *J* = 7.7 Hz, 4H), 6.76 – 6.71 (m, 3H), 3.78 (d, *J* = 15.9 Hz, 2H), 3.62 (d, *J* = 15.8 Hz, 2H), 3.45 (s, 2H), 2.50 (s, 6H), 2.08 (s, 3H), 1.97 (s, 6H), 0.42 (d, *J* = 8.6 Hz, 9H). **^{13}C NMR** (151 MHz, [D$_8$]-toluene, 233 K): δ 310.7 (d, *J* = 27.0 Hz), 143.0 (d, *J* = 2.3 Hz), 142.6 (d, *J* = 3.5 Hz), 139.4, 138.7, 138.6, 137.12, 137.09, 136.8, 136.5, 136.4, 136.1, 135.8, 135.6, 135.1 (d, *J* = 2.1 Hz), 133.5, 133.2, 132.3, 129.5, 129.4, 129.3, 129.0, 128.8, 128.9, 127.8, 127.3, 126.9, 55.1, 54.4, 20.9, 19.5, 19.0, 14.1 (d, *J* = 21.9 Hz). **^{29}Si NMR** (119 MHz, [D$_8$]-toluene, 233 K): δ −19.8 (d, *J* = 4.2 Hz), −16.0. **^{31}P NMR** (243 MHz, [D$_8$]-toluene, 233 K): δ −1.8.

Benchmarking Experiments: Catalyst Activity

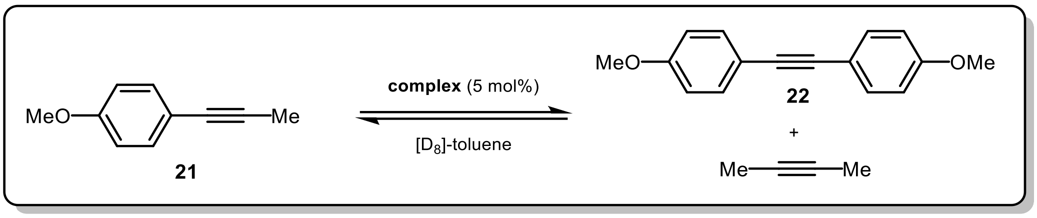

A flame-dried *J. Young* NMR tube filled with argon was charged with a stock solution of the respective complex (6.0 µM in [D8]-toluene, 0.5 mL, 3.0 µmol, 5 mol%) and a ^{1}H NMR spectrum recorded at 25 °C. A solution of 1-methoxy-4-(prop-1yn-1yl)benzene **21** (0.6 M in [D8]-toluene, 100 µL, 60.0 µmol) was then added and the conversion of the substrate to tolane **22** and 2-butyne was monitored by ^{1}H NMR spectroscopy at 25 °C (unless otherwise specified). The delay between the addition and the first NMR measurement amounts to 1 – 2 min due to sample loading and shimming. ^{1}H NMR spectra were acquired with a single scan every 60 sec. For measurements requiring lower temperatures the spectrometer was precooled, the sample prepared at −78 °C and directly transferred into the spectrometer.

Benchmarking Experiment for Adduct 16a (at 25 °C)

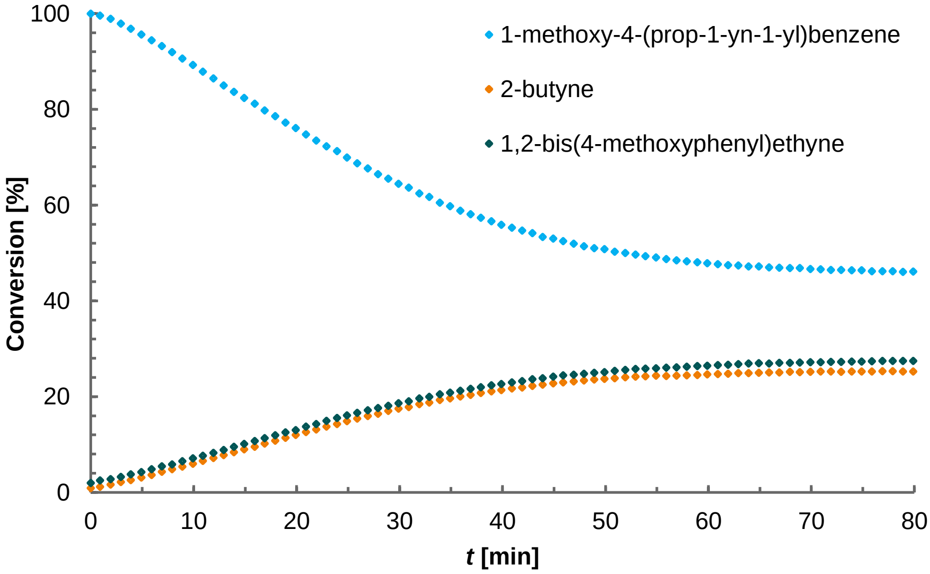

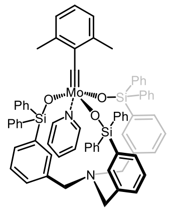

16a

Figure S2. Consumption of alkyne **21** and generation of product **22** and 2-butyne in [D8]-toluene in the benchmarking experiment using adduct **16a** as the catalyst (5 mol%), as monitored by ^{1}H NMR spectroscopy at 25 °C.

Comparison of Free Complex 15a to Pyridine Adduct 16a (at 25 °C)

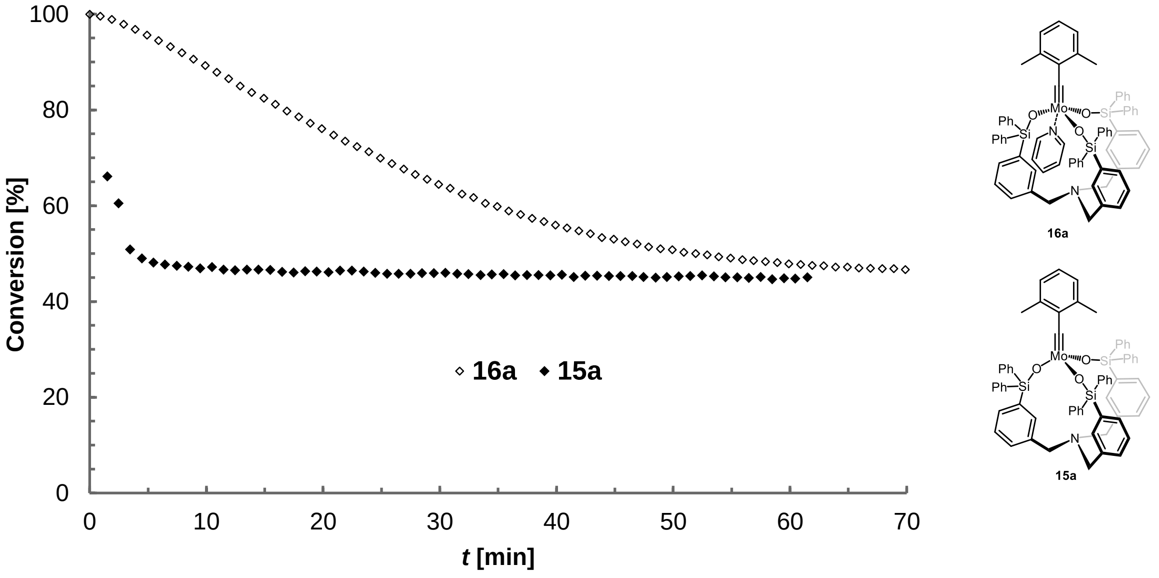

Figure S3. Consumption of alkyne **21** in benchmarking experiments using either the pyridine adduct **16a** or the free complex **15a** (5 mol%) as catalyst in [D8]-toluene, as monitored by ^{1}H NMR spectroscopy at 25 °C.

Comparison of Complex 15b to Complex 17b (at 0 °C)

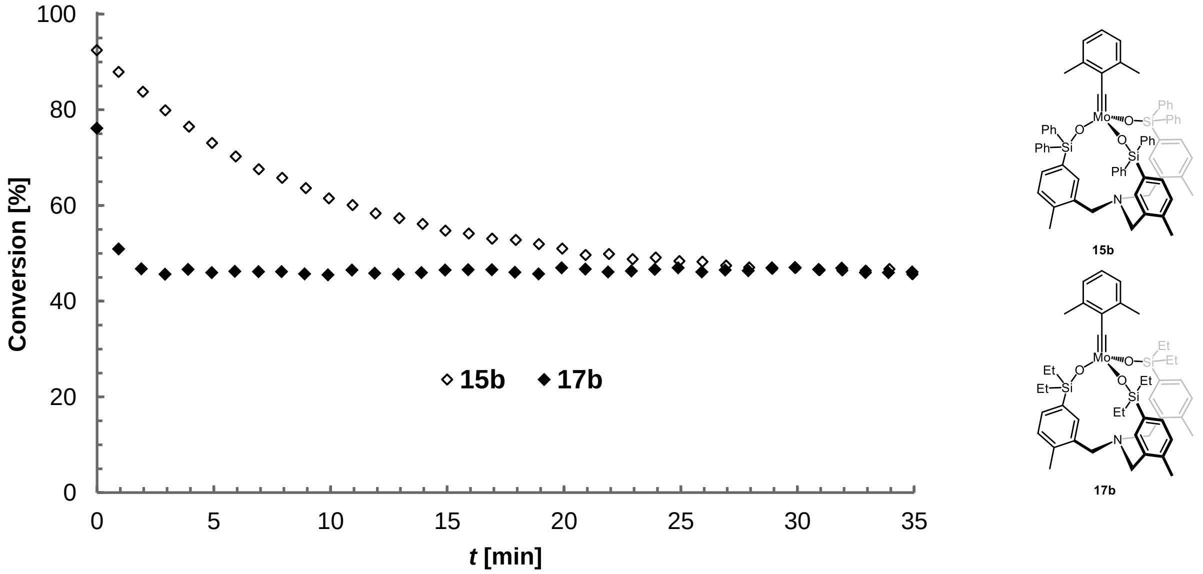

Figure S4. Consumption of alkyne **21** in benchmarking experiments using either complex **15b** or complex **17b** (5 mol%) as catalyst in [D8]-toluene, as monitored by ^{1}H NMR spectroscopy at 0 °C.

Comparison of Canopy Catalyst 2a to Complex 17b (at −20 °C)

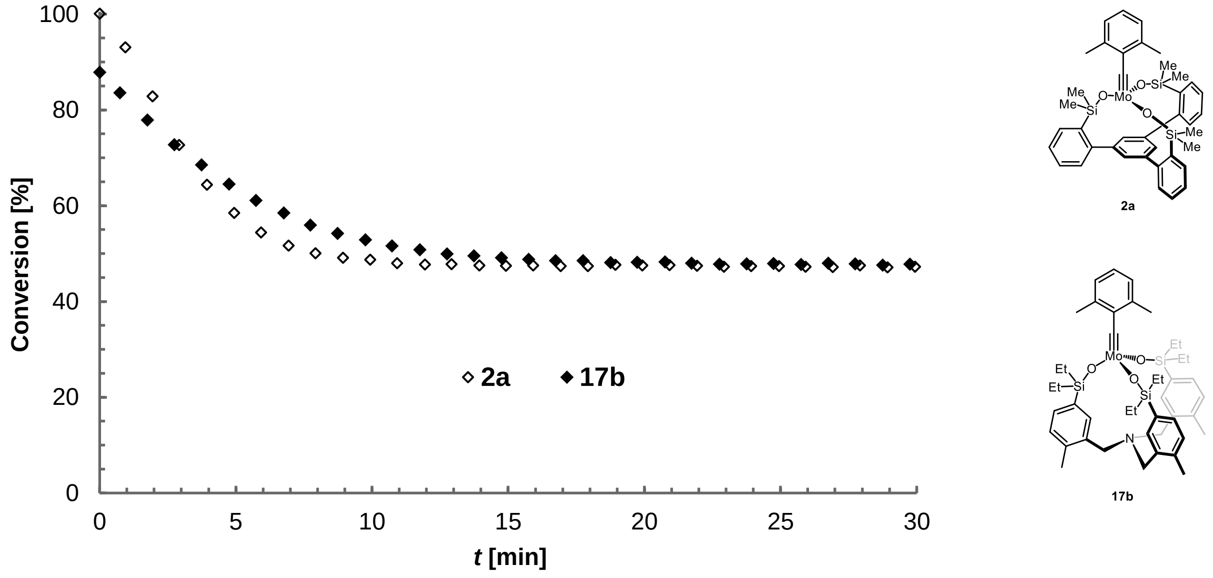

Figure S5. Consumption of alkyne **21** in benchmarking experiments using either canopy catalyst **2a** or complex **17b** (5 mol%) as catalyst in [D8]-toluene, as monitored by ^{1}H NMR spectroscopy at −20 °C.

Comparison of Adducts 16b, 16c, 16d, and 16e

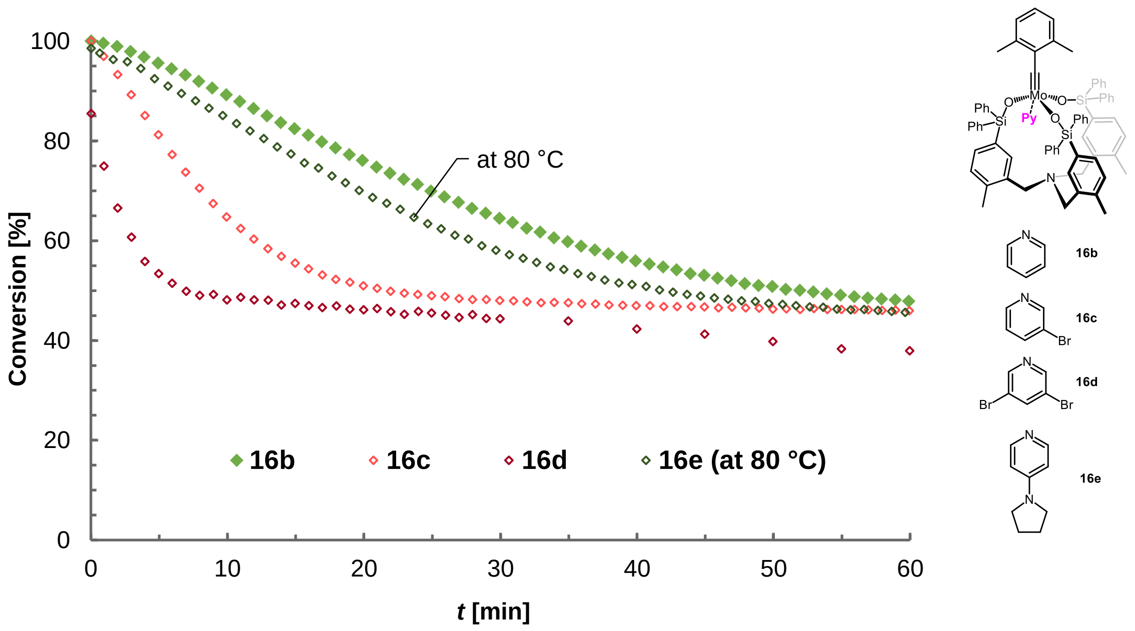

Figure S6. Consumption of alkyne **21** in benchmarking experiments using adducts **16b-e** (5 mol%) as catalysts in [D8]-toluene, as monitored by ^{1}H NMR spectroscopy; the reactions were performed at 25 °C except for the reaction catalyzed by **16e**, which was carried out at 80 °C.

Benchmarking Experiment for the Octahedral Tris-Phenolate Complex 10 (at 70 °C)

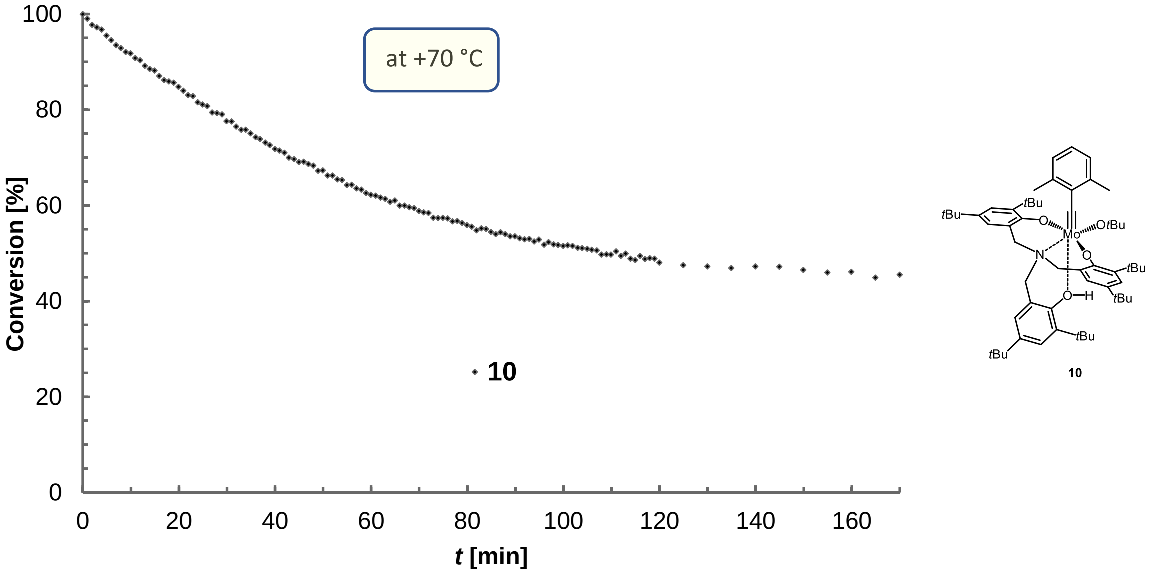

Figure S7. Consumption of alkyne **21** in a benchmarking experiment using complex **10** (5 mol%) as catalyst in [D8]-toluene, as monitored by ^{1}H NMR spectroscopy at +70 °C.

Benchmarking Experiment Using a Zhang-type Catalyst Prepared in situ from the Molybdenum Alkylidyne 6 and the Tris-Phenol Ligand 7 (Z = CH, R = *i*Pr)

A stock solution of the catalytically active mixture was prepared by premixing molybdenum alkylidyne **6** (6.0 mg, 9 µmol) and ligand **7** (Z = CH, R = *i*Pr; 4.2 mg, 9 µmol)[30] in $CDCl_3$ (1.5 mL) in a Schlenk flask. The solution was stirred for 30 min. A flame-dried *J. Young* NMR tube filled with argon was charged with an aliquot of the stock solution (6.0 µM in $CDCl_3$, 0.5 mL, 3.0 µmol, 5 mol%), which was then cooled to −50 °C. A solution of 1-methoxy-4-(prop-1yn-1yl)benzene **21** (0.6 M in [D8]-toluene, 100 µL, 60.0 µmol) was added and the tube was transferred into the precooled NMR spectrometer. The temperature of the sample was then raised from 253 K to 273 K and later to 298 K as no conversion was observed at the lower temperatures (see Figure S8).

The lack of reactivity at low temperature stands in marked contrast to the excellent performance of the new catalysts at 0°C and even –20°C, resulting in equilibration withing minutes (see Fig. S4 and S5).

Note: $CDCl_3$/[D8]-*toluene was chosen as the solvent system for this and the following benchmarking experiment. This medium is described in the literature as suitable, even though* CCl_4 *was recommended as optimal.*[30] *Not only is* CCl_4 *less adequate for the NMR experiments, but this chemical is hepatotoxic and potentially carcinogenic*. *Therefore* CCl_4 *is regulated under Regulation (EC) No 1005/2009 of the European Parliament and of the Council on substances that deplete the ozone layer (OJ L 286, 31.10.2009, p. 1) which prohibits its use except as an intermediate, industrial processing agent and laboratory agent.*

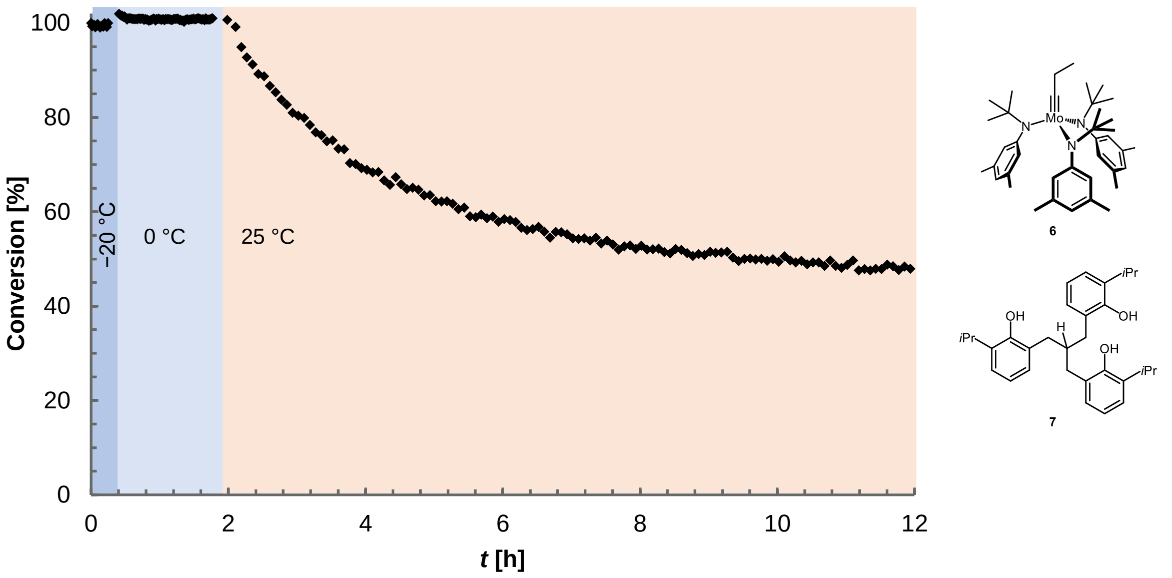

Figure S8. Consumption of alkyne **21** in a benchmarking experiment using a catalyst generated in situ from the molybdenum alkylidyne **6** and ligand **7** (5 mol% each) in a mixture of $CDCl_3$ and [D_8]-toluene, as monitored by ^{1}H NMR spectroscopy at 253 K (–20 °C), 273 K (0 °C) and 298 K (+25°C).

Side Reaction: Polymerization

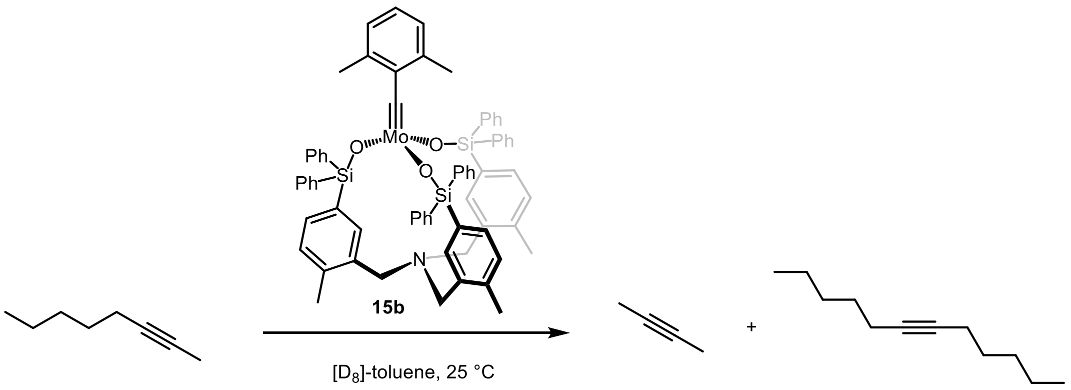

A flame-dried *J. Young* NMR tube filled with argon was charged with a stock solution of the complex **15b** (6.0 µM in [D8]-toluene, 0.5 mL, 3.0 µmol, 5 mol%) and a ^{1}H NMR spectrum recorded at 25 °C. A solution of 2-octyne (0.6 M in [D8]-toluene, 100 µL, 60.0 µmol) was then added and the conversion of this substrate to dodec-6-yne and 2-butyne was monitored by ^{1}H NMR spectroscopy at 25 °C. The initial equilibrium was reached upon the first scan and the sample was further monitored for 70 h. Polymerization of 2-butyne as well as 2-octyne and dodec-6-yne slowly lowered the concentration of these monomers in solution. After 70 h, the material in the NMR tube had converted into a gel.

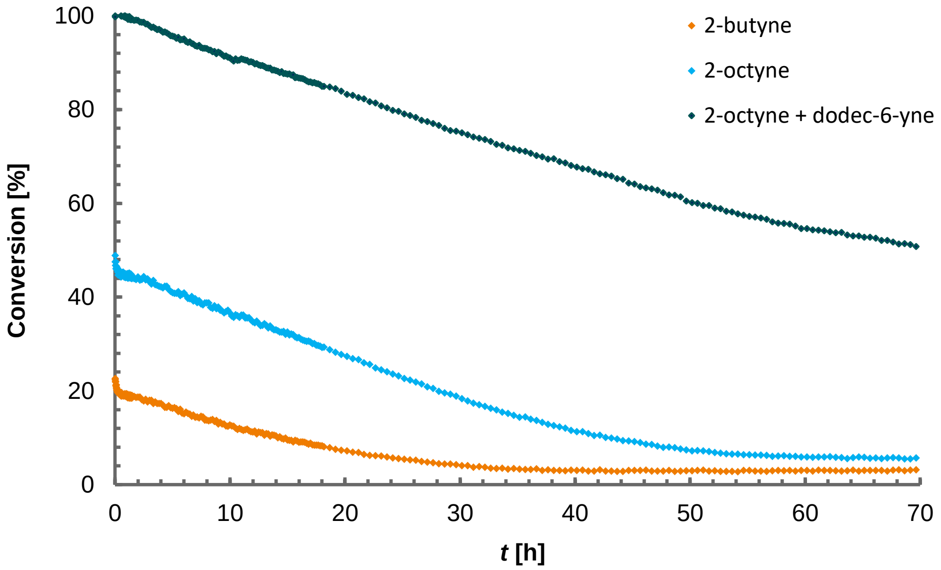

Figure S9. Consumption of 2-octyne in the presence of complex **15b** (5 mol%) in [D8]-toluene, as monitored by ^{1}H NMR spectroscopy at 25 °C.

Pyridine Scavenger Strategy

A flame-dried *J. Young* NMR tube filled with argon was charged with a stock solution of adduct **16a** (6.0 µM in [D8]-toluene, 0.5 mL, 3.0 µmol) and a ^{1}H NMR spectrum was recorded at 25 °C. A solution of triphenylborane (0.06 M in [D8]-toluene, 50 µL, 3.0 µmol) was then added, causing a color change from purple to orange (see inserted photographs), along with a characteristic change of the ^{1}H NMR spectrum recorded at 25 °C.

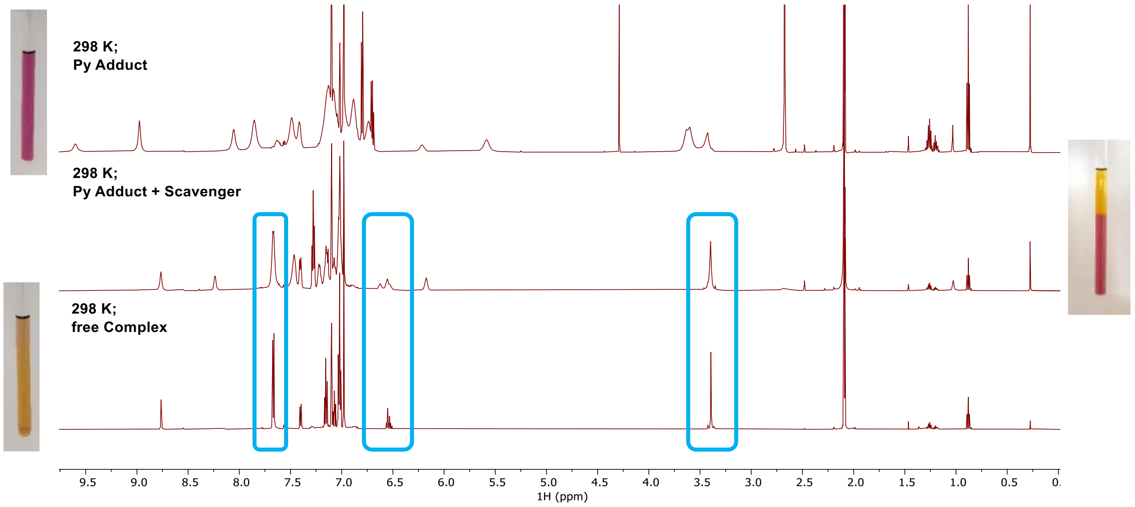

Benchmarking Experiment for Adduct 16a with/without Pyridine Scavenger (at 25 °C)

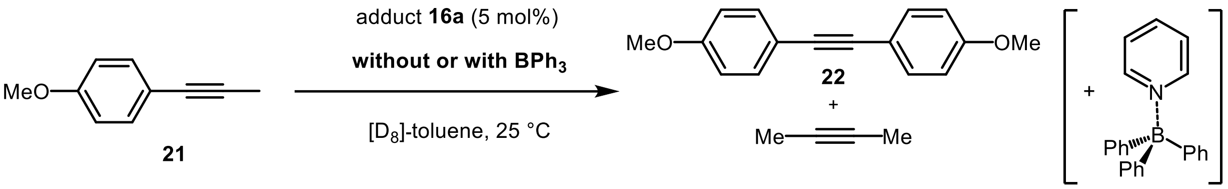

A flame-dried *J. Young* NMR tube filled with argon was charged with a stock solution of adduct **16a** (6.6 µM in [D8]-toluene, 0.45 mL, 3.0 µmol, 5 mol%). A solution of triphenylborane (0.06 M in [D8]-toluene, 50 µL, 3.0 µmol) was added, followed by a solution of alkyne **21** (0.6 M in [D8]-toluene, 100 µL, 60.0 µmol). The conversion of the substrate to tolane **22** was monitored by ^{1}H NMR spectroscopy at 25 °C.

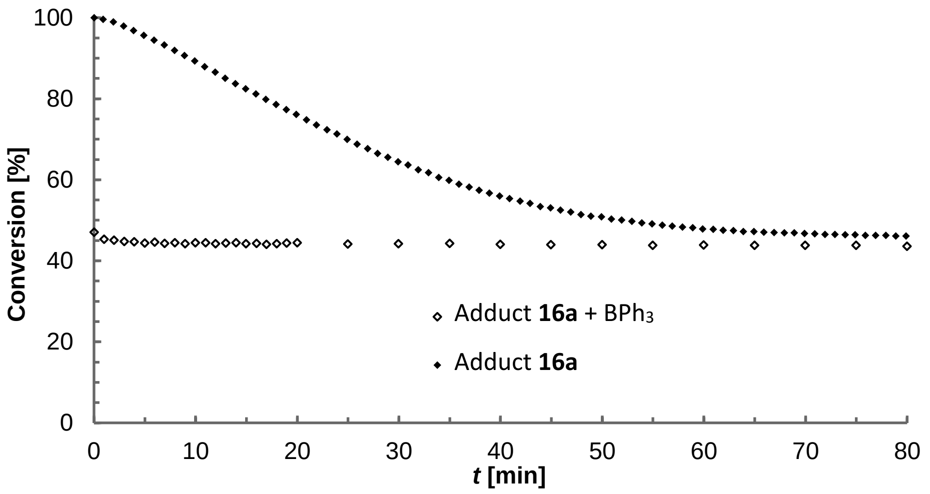

Figure S10. Consumption of alkyne **21** in a benchmarking experiment using adduct **16a** (5 mol%) as catalyst in [D8]-toluene with or without the addition of BPh_3 as a pyridine scavenger at 25 °C.

Reactive Intermediates

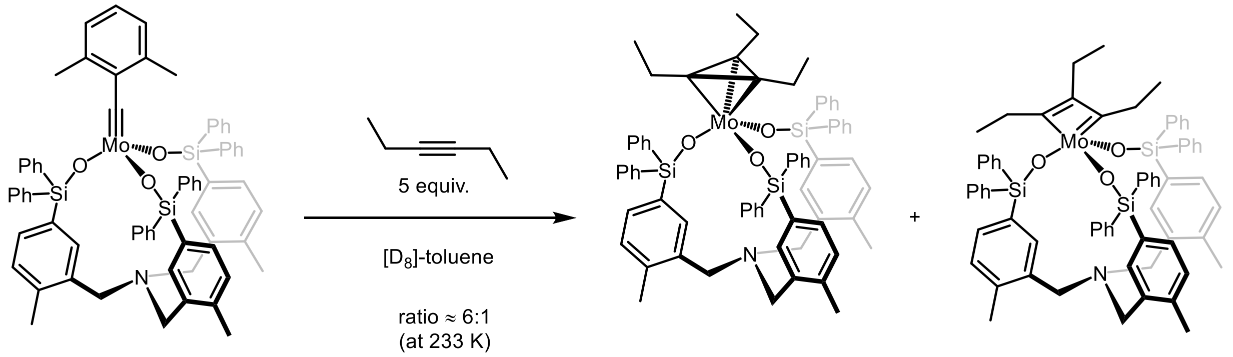

A flame-dried *J. Young* NMR tube filled with argon was charged with a stock solution of complex **15b** (20 µM in [D8]-toluene, 0.6 mL, 12 µmol) and a ^{1}H NMR spectrum recorded at 298 K. 3-Hexyne (7 µL, 61 µmol) was added and the *J. Young* NMR tube was inverted three times to ensure mixing, before a first ^{1}H NMR spectrum was recorded. A variable-temperature NMR study was conducted from 298 K to 233 K.

The major product could be characterized as metallatetrahedrane and all peaks were assigned. In contrast to what had been observed for the metallatetrahedrane derived from the canopy catalyst **2b**,[12] however, the protons of the methylene groups of the ethyl substituents at the metallatetrahedrane unit are not diastereotopic in this case. **^{1}H NMR** (600 MHz, [D8]-toluene, 233 K): δ 9.22 (s, 3H), 7.71 (d, *J* = 6.8 Hz, 12H), 7.34 (d, *J* = 7.4 Hz, 3H), 7.22 (t, *J* = 7.4 Hz, 6H), 7.16 (d, *J* = 7.5 Hz, 12H), 6.96 (d *J* = 7.4 Hz, 3H), 3.31 (s, 6H), 2.60 (q, *J* = 7.3 Hz, 6H), 2.01 (s, 9H), 0.58 (t, *J* = 7.5 Hz, 9H). **^{13}C NMR** (151 MHz, [D8]-toluene, 233 K): δ 138.4, 136.9, 137.6, 137.4, 135.6, 135.3, 135.1, 132.8, 129.5, 129.3, 82.6, 53.8, 20.3, 19.6, 14.9. **^{29}Si NMR** (119 MHz, [D8]-toluene, 233 K): δ −11.2.

The minor product showed broad signals at higher temperatures. Due to the low concentration and lower symmetry not all signals could be unambiguously assigned, but all characteristic signals support the structure assignment as metallacyclobutadiene complex.[12] This assignment is also consistent with the fact that this complex undergoes exchange with free 3-hexyne even at 233 K, whereas exchange for the metallatetrahedrane is only observed at 298 K.

^{1}H NMR of the reaction mixture, 600 MHz, [D8]-toluene, 233 K

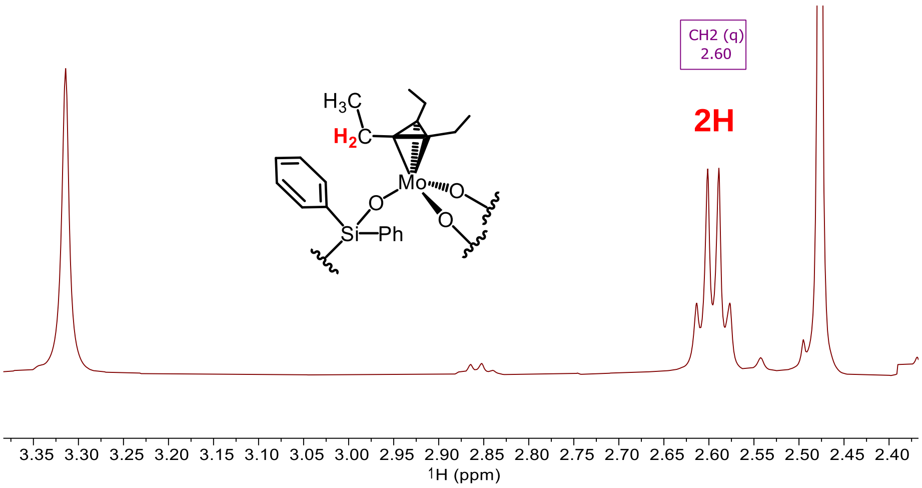

^{1}H NMR of the reaction mixture, 600 MHz, [D8]-toluene, 298-233 K

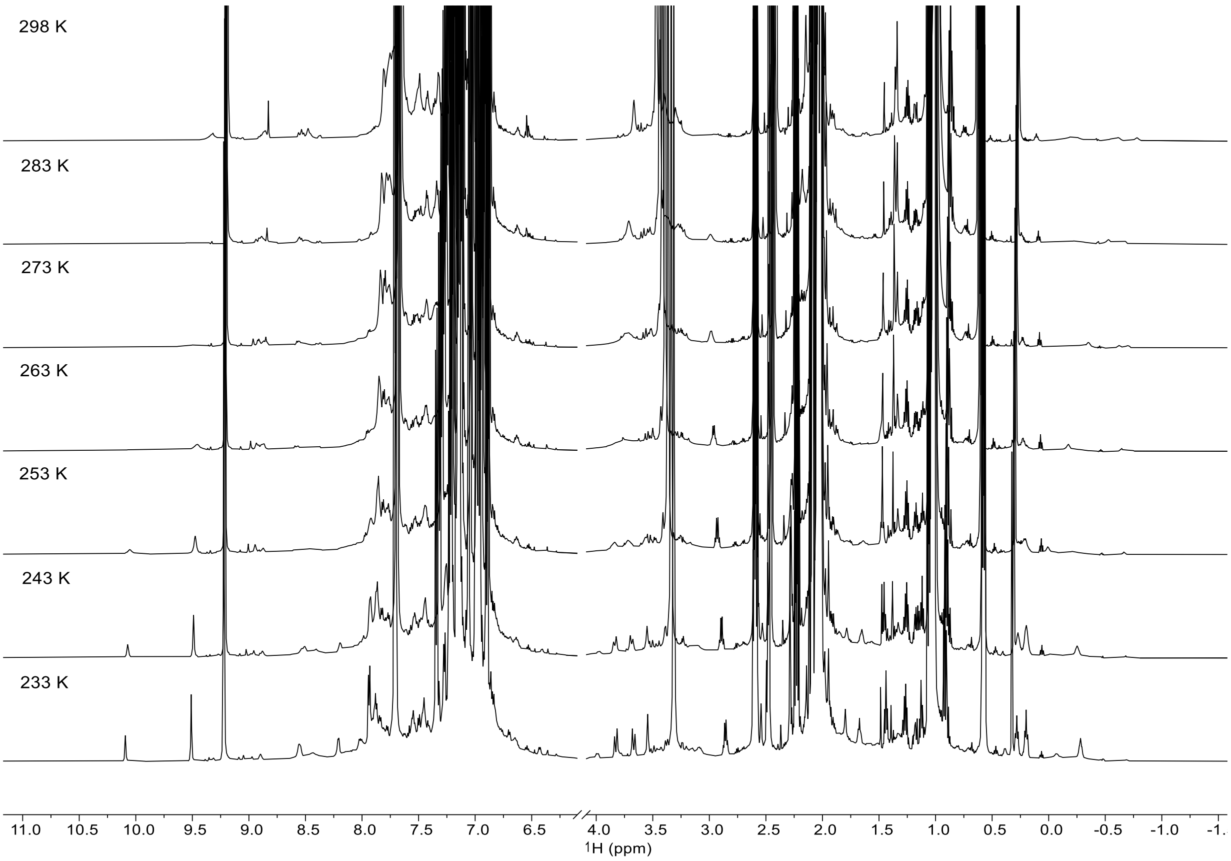

^{1}H -^{1}H ROESY of the reaction mixture, 600 MHz, [D8]-toluene, 233 K

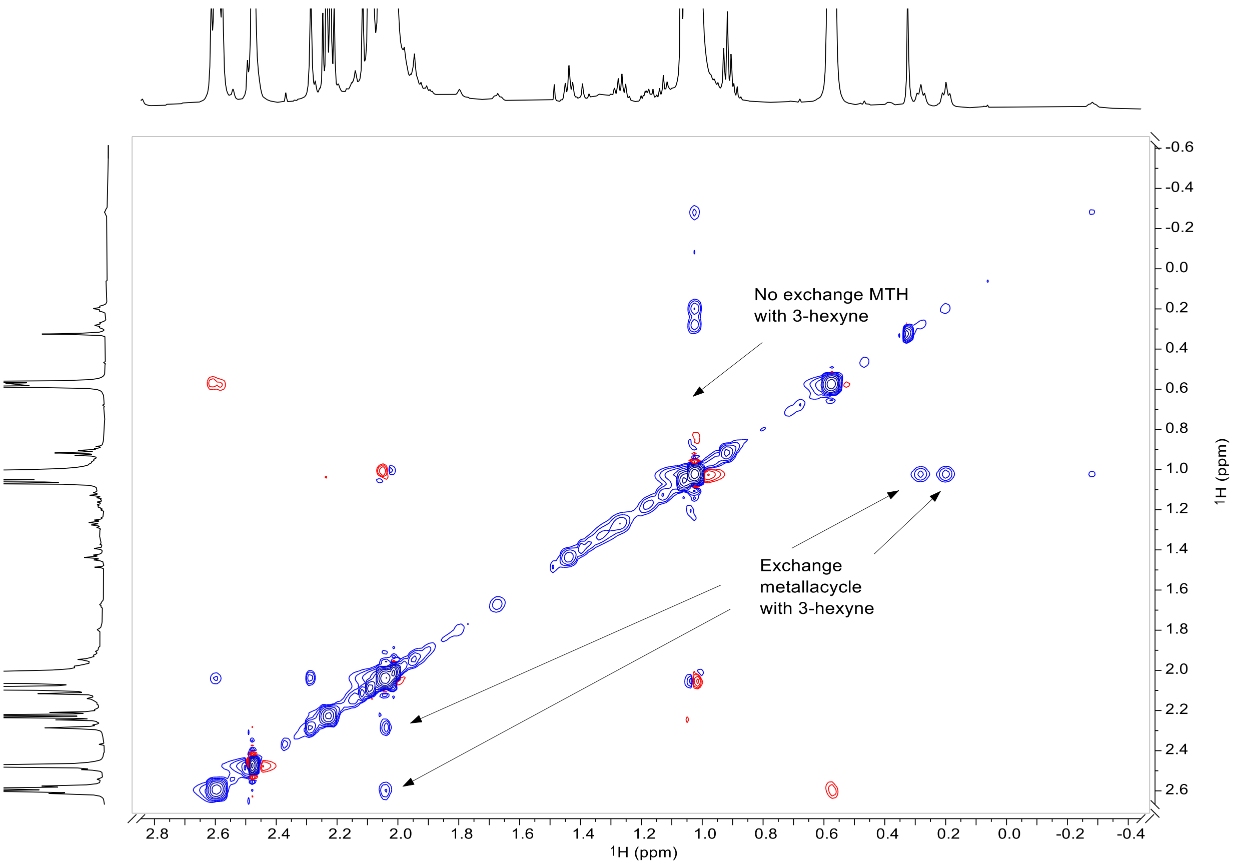

^{1}H -^{1}H ROESY of the reaction mixture, 600 MHz, [D8]-toluene, 298 K

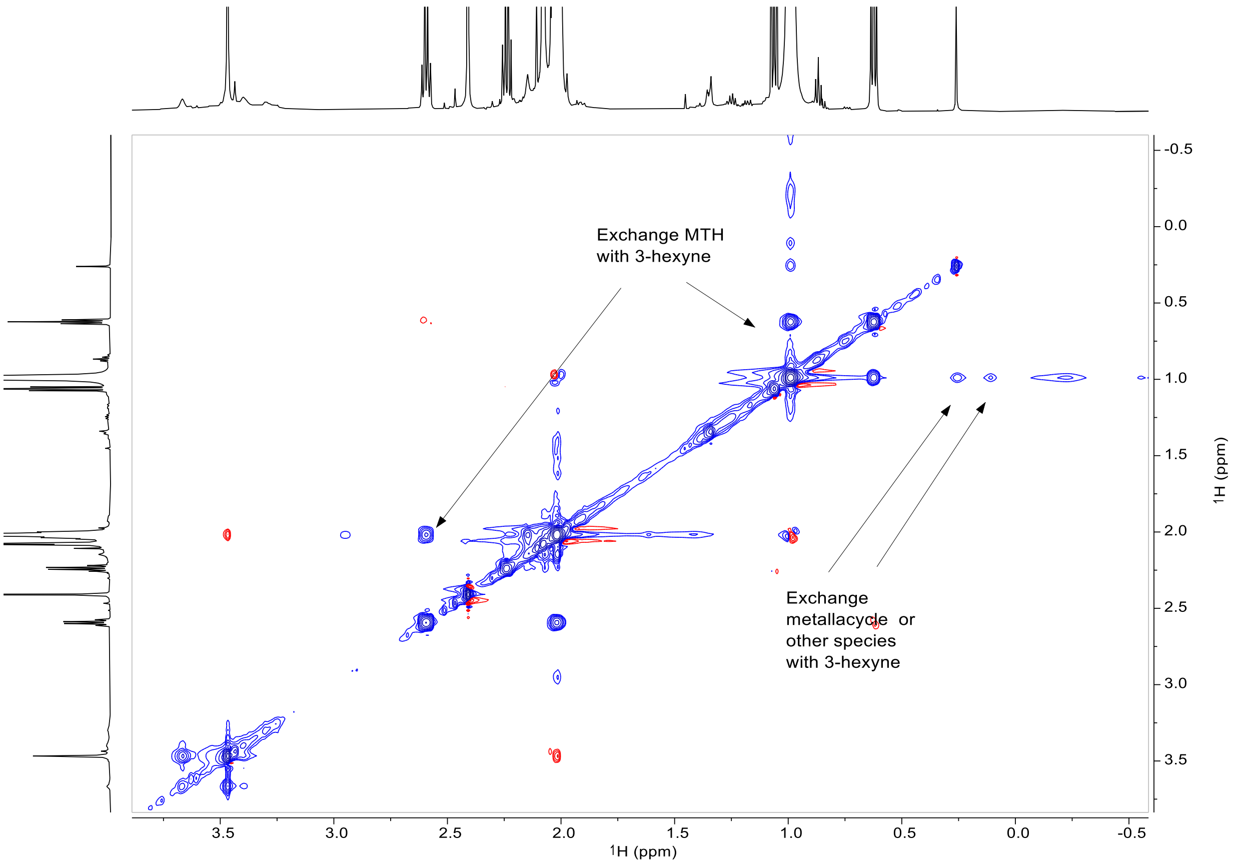

Stability

Stability in the Presence of 2-Butyne

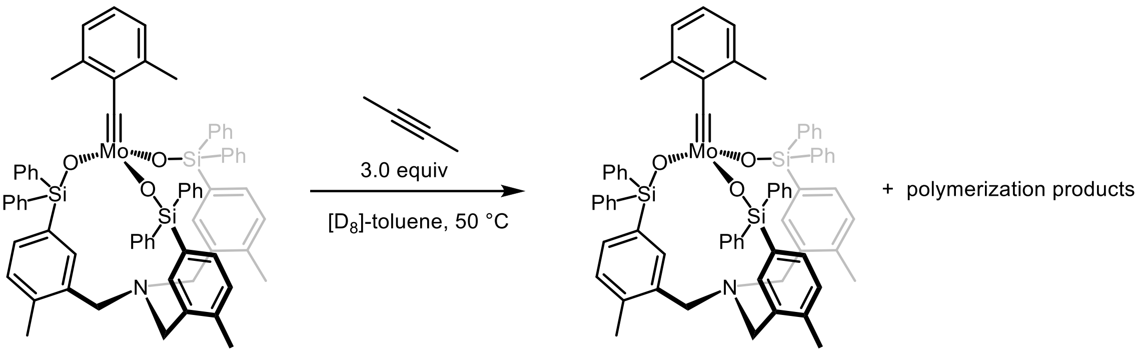

A flame-dried *J. Young* NMR tube filled with argon was charged with a stock solution of the complex **15b** (18 µM in [D8]-toluene, 0.5 mL, 9 µmol). An aliquot of a stock solution of 2-butyne (0.06 M in [D8]-toluene, 450 µL, 27 µmol) was added and the reactivity of complex **15b** monitored by ^{1}H NMR spectroscopy at 50 °C.

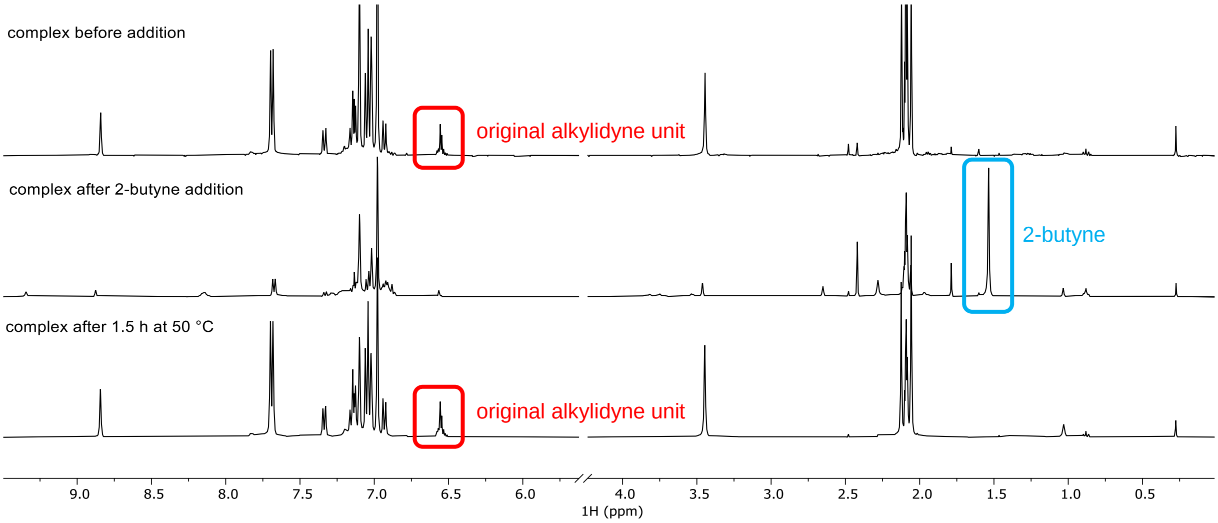

Upon addition of the 2-butyne, the signals of **15b** instantly disappear and excess free 2-butyne is detected. Subsequently, the signal of 2-butyne became broad likely due to polymerization. After 1.5 h, no signal for 2-butyne could be observed any longer and the catalyst had returned to its original state.

Stability of Powdered Pyridine-free Complex 15a in Air

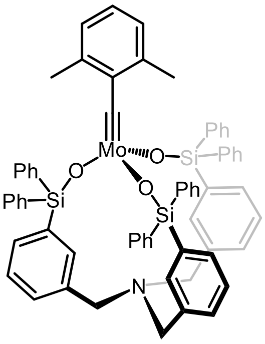

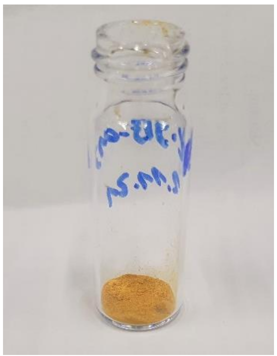

A sample of complex **15a** was placed in a vial open to air, which was kept on the bench. At the indicated time intervals, aliquots of this batch (5 mg) were dissolved in [D$_8$]-toluene (0.5 mL) in a Schlenk flask and transferred into a flame-dried *J. Young* NMR tube filled with argon. The degree of decomposition was assessed by ^{1}H NMR spectroscopy at 25 °C.

^{1}H NMR of complex 15a, 400 MHz, [D$_8$]-toluene, 25 °C

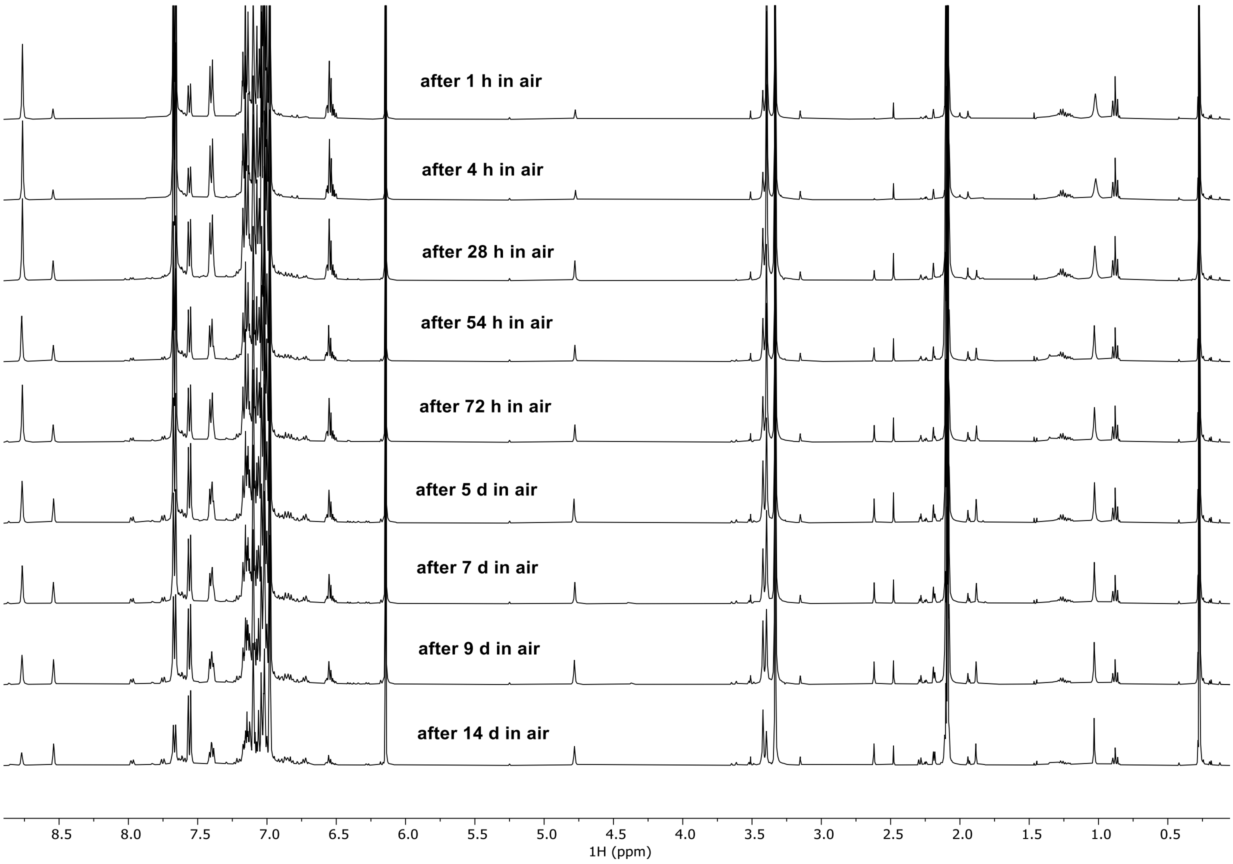

Stability of Adduct 16b in Crystalline Form in Air

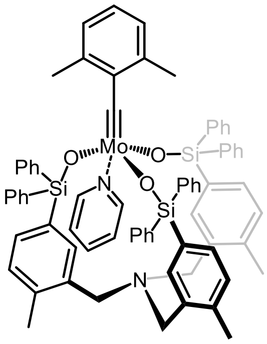

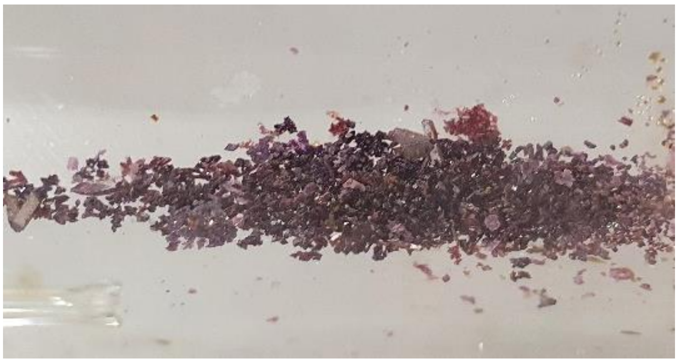

A batch of crystalline adduct **16b** was placed in a vial under air at ambient temperature and kept on the bench. At the indicated time intervals, aliquots of this batch (5 mg) were dissolved in [D_8]-toluene (0.5 mL) in a Schlenk flask and the solution was transferred into a flame-dried *J. Young* NMR tube filled with argon. The stability was assessed by ^{1}H NMR spectroscopy at 253 K.

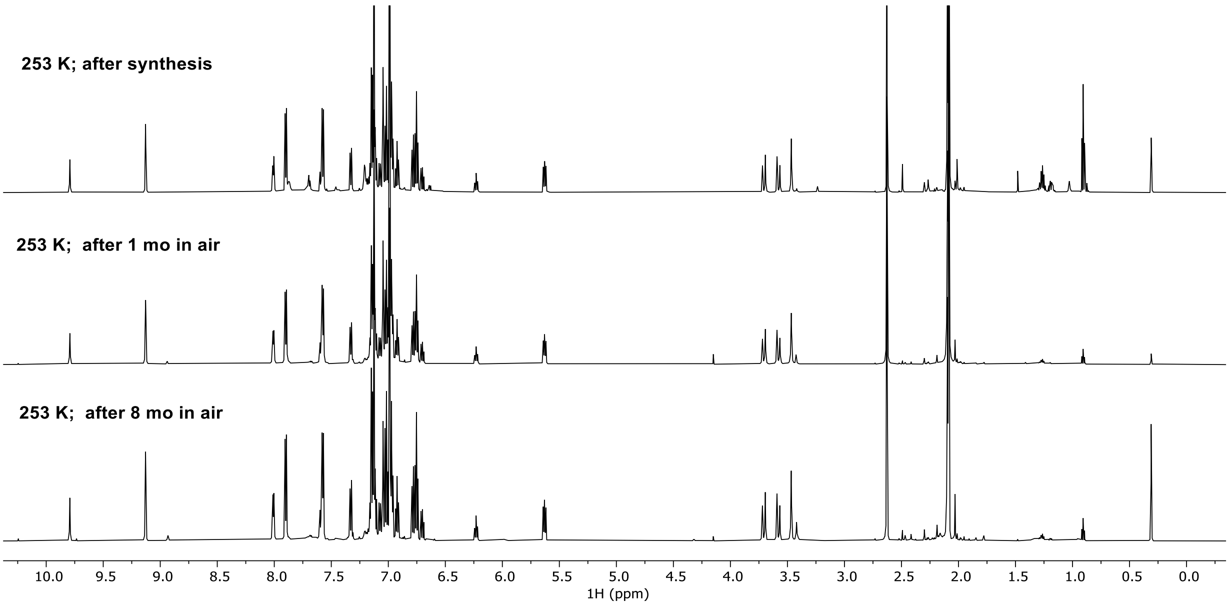

Additionally, the activity of the aged catalyst samples was tested in the standard benchmarking experiment described below and found unchanged even after 8 months of storage in air (Figure S11).

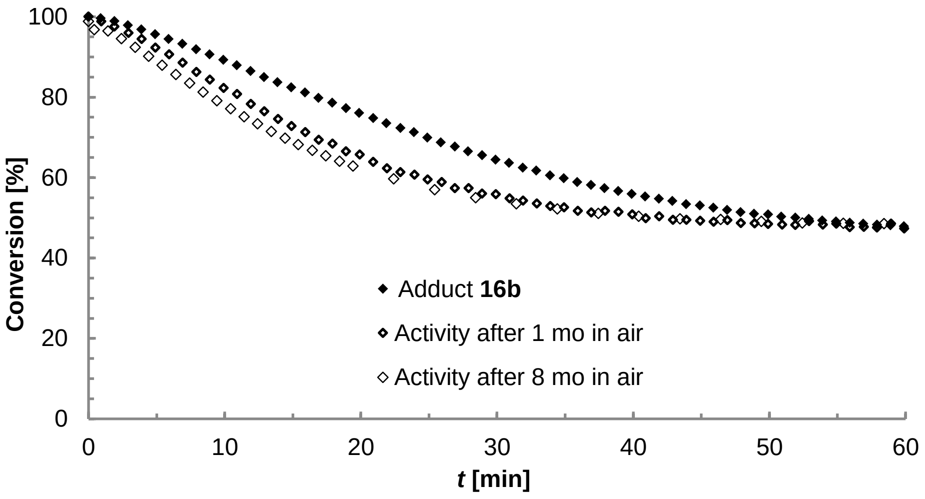

Figure S11. Consumption of alkyne **21** in benchmarking experiments using samples of crystalline adduct **16b** (5 mol%) in [D_8]-toluene as catalyst as monitored by ^{1}H NMR spectroscopy at 25 °C. The samples were either freshly prepared, or had been stored in air for 1 month or 8 months.

Stability of Adduct 16a as a Powder under Optimal Storage Conditions

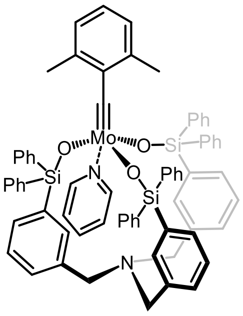

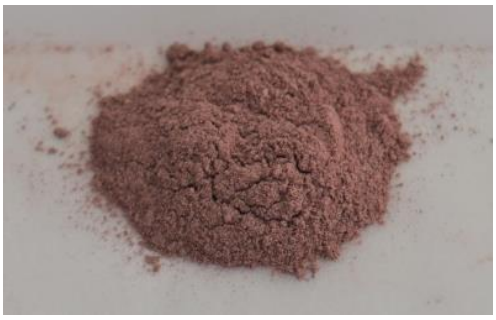

A batch of adduct **16a** in powder form was placed in an open vial which was stored at ambient temperature in an air-filled desiccator over silica orange gel (activated at 150 °C in the oven) as the drying agent. At the indicated time intervals, the vial was taken out of the desiccator and aliquots of the sample (5 mg) were dissolved in [D$_8$]-toluene (0.5 mL) and the solution transferred into a flame-dried *J. Young* NMR tube filled with argon. The stability was assessed by ^{1}H NMR spectroscopy at 253 K. The storage vial was again placed in the desiccator.

^{1}H NMR Spectra of Adduct 16a Stored in a Desiccator, 600 MHz, [D$_8$]-toluene, 253 K

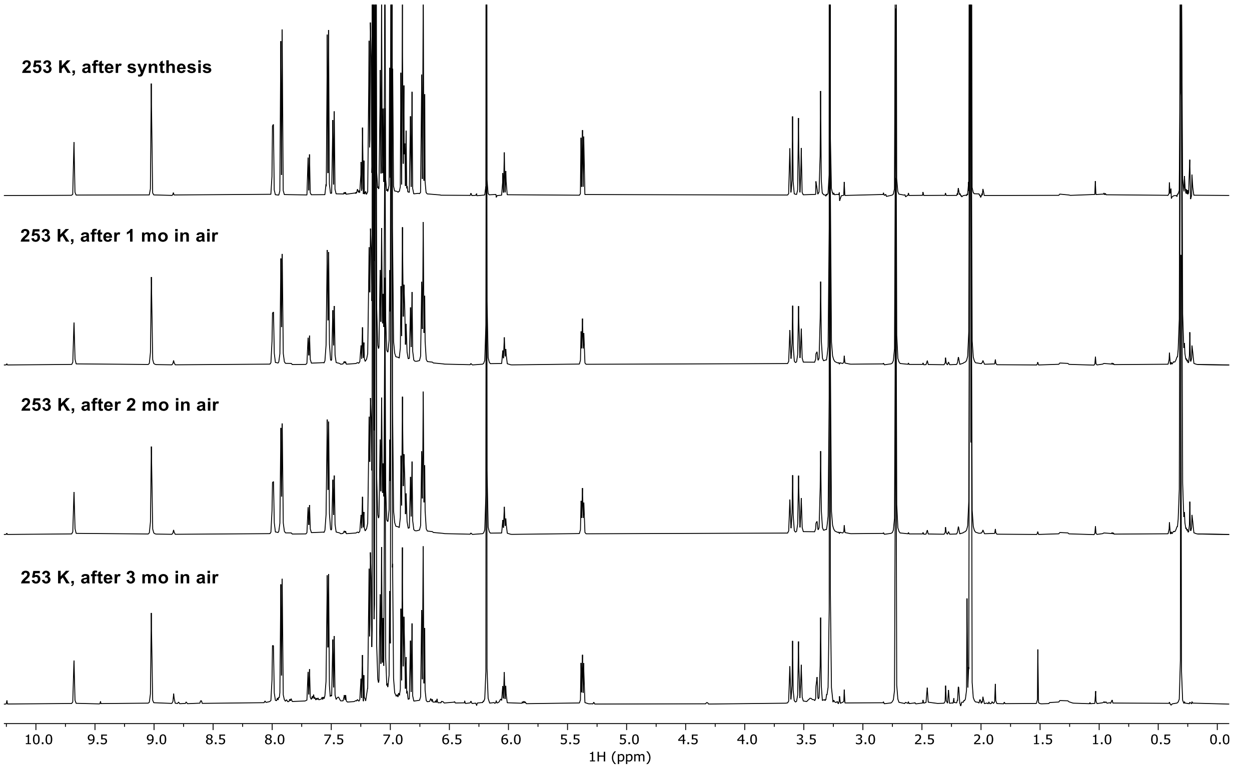

A second batch of adduct **16a** in powder form was placed in a vial which was briefly flushed with argon, closed with a non-air-tight screw cap, and stored at −20 °C. At the indicated time intervals the vial was removed from the freezer, allowed to reach ambient temperature and opened in air. Aliquots of the sample (5 mg) were dissolved in [D8]-toluene (0.5 mL) and the solution transferred into a flame-dried *J. Young* NMR tube filled with argon. The stability was assessed by ^{1}H NMR spectroscopy at 253 K. The storage vial was again flushed with argon, closed with the screw cap, and stored at −20 °C.

^{1}H NMR Spectra of Adduct 16a Stored in a Freezer, 600 MHz, [D8]-toluene, 253 K

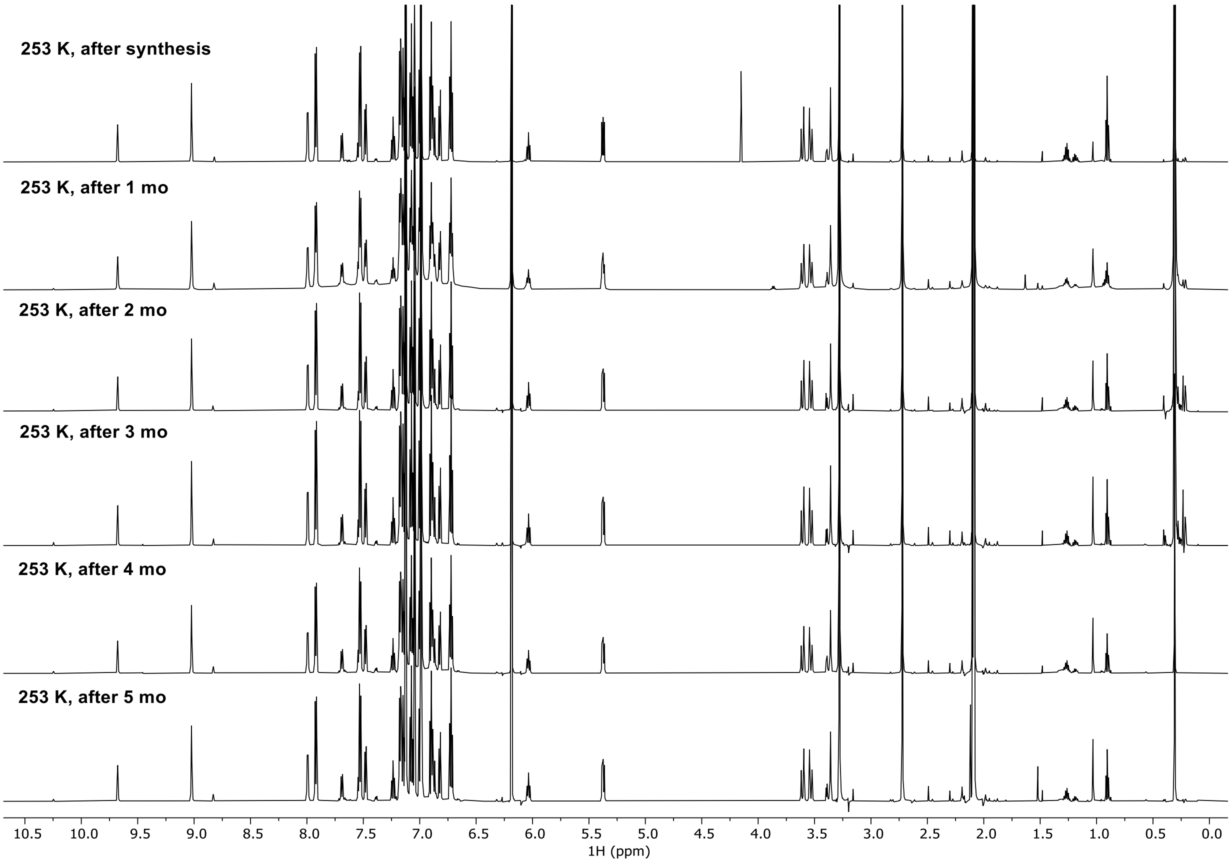

Combustion analysis of a sample taken from the catalyst batch after 4 months confirmed that the material was intact at this point in time; the following data were recorded: **Elemental analysis** (%) calc. for $C_{71}H_{62}MoN_2O_3Si_3$: C 72.79, H 5.33, N 2.39; found: C 72.42, H 5.51, N 2.31.

Benchmarking: Stability of Powdered Complex 2a in Air

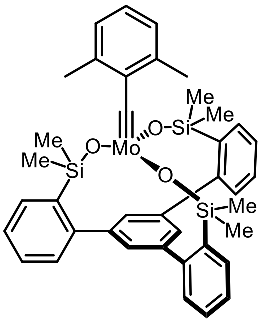

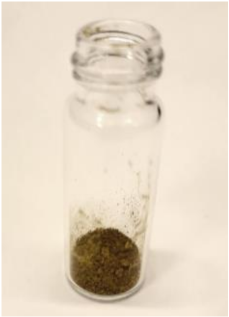

A sample of complex **2a** was placed in a vial open to air, which was kept on the bench. At the indicated time intervals, aliquots of this batch (7 mg) were dissolved in C_6D_6 (0.55 mL) in a Schlenk flask and the resulting solutions transferred into a flame-dried *J. Young* NMR tube filled with argon. The degree of decomposition was assessed by ^{1}H NMR spectroscopy at 25 °C.

Under these conditions, the compound decomposes within ca. 4 h.

^{1}H NMR of complex 2a, 400 MHz, C_6D_6, 25 °C

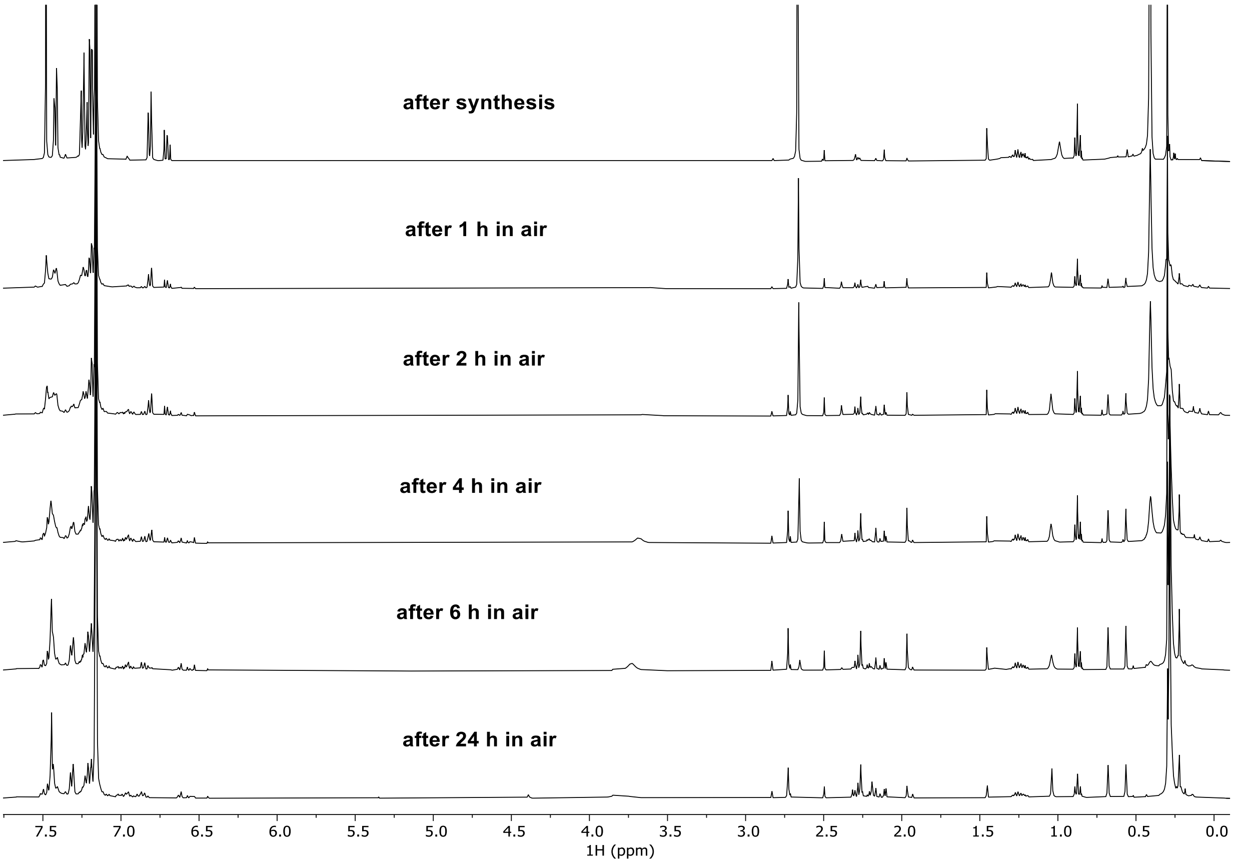

Benchmarking: Stability of a Prototype Zhang-Type Catalyst System in Air

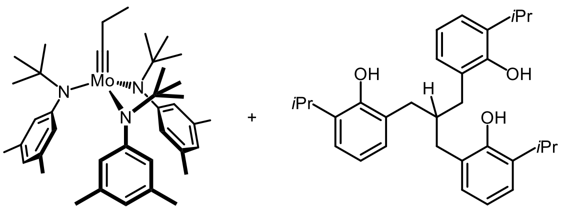

A 5 mL Schlenk flask was charged with the molybdenum alkylidyne **6** (10 mg, 15 µmol) and $CDCl_3$ (1.0 mL). Ligand **7** (6.9 mg, 15 µmol)[30] was added and the resulting solution was stirred for 30 min. One half of the solution (0.5 mL) was transferred into a flame-dried *J. Young* NMR tube filled with argon and analyzed by NMR spectroscopy. The ^{1}H NMR (see below) showed a mixture comprising (at least) two major components, the exact constitution of which is unclear. Importantly, the ^{1}H NMR and HMBC spectra were virtually identical to the corresponding spectra depicted in the Supporting Information of ref. [30]

The solvents were removed from the other half of the solution to give a brown viscous residue, which was exposed to air. After 12 h, the residue was dissolved in deuterated chloroform (0.5 mL) in a Schlenk flask and transferred into a flame-dried *J. Young* NMR tube filled with argon. No signals attributed to the mixture of active components were visible and only the resonances of the free ligand and unbound HN[(Ar)(*t*Bu)]$_3$ derived from **6** were detected by ^{1}H NMR spectroscopy, implying that the sample was fully composed at this point.

To confirm this conclusion, a small amount of alkyne **21** (ca. 3 mg) was added to the solution, but no metathesis reaction was observed by ^{1}H NMR, showing that all catalytic activity was lost.

^{1}H NMR of a catalyst formed in situ from molybdenum alkylidyne 6 of ligand 7, 600 and 400 MHz, $CDCl_3$, 25 °C

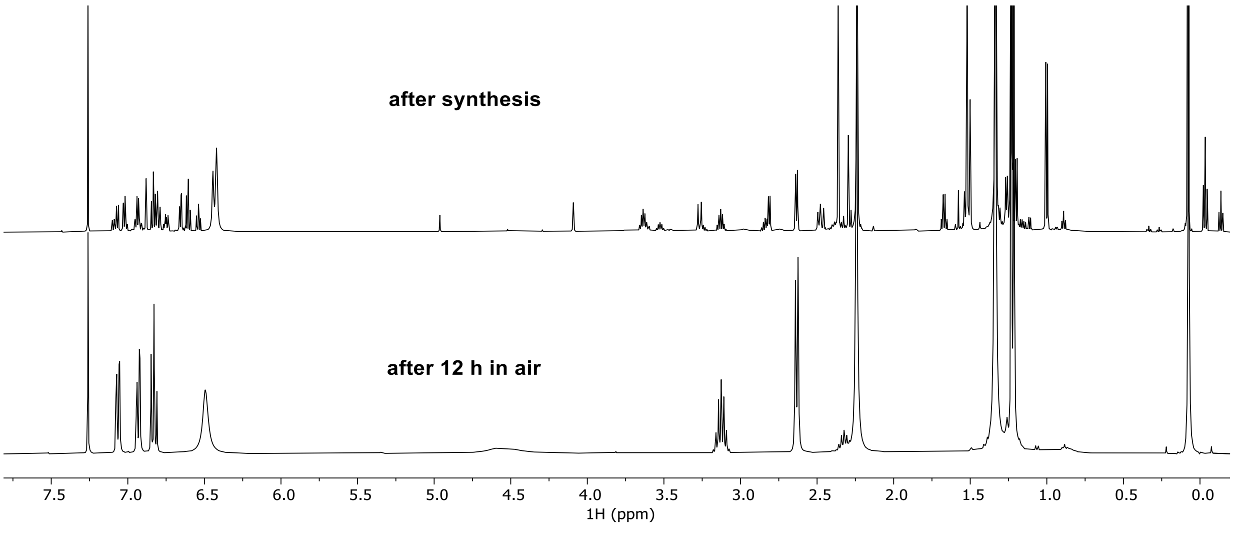

Alternative Set-Up: A sample of molybdenum alkylidyne **6** was placed in a vial open to air, which was kept on the bench. After 2 h, an aliquot of this "aged" batch (1.9 mg, 2.9 µmol) was dissolved in $CDCl_3$ (0.5 mL) in a Schlenk flask and ligand **7** (1.2 mg, 2.9 µmol) was added. After stirring for 30 min, the solution was transferred into a flame-dried *J. Young* NMR tube filled with argon. No signs of complex formation were observed but only signals of the free ligand and unbound HN[(Ar)(*t*Bu)]$_3$ derived from **6** were detected (see below), implying that alkylidyne **6** must have been fully decomposed before **7** was introduced.

To confirm this conclusion, a small amount of alkyne **21** (ca. 3 mg) was added to the solution, but no metathesis reaction was observed by ^{1}H NMR, showing that all catalytic activity was lost.

^{1}H NMR of a mixture of "aged" 6 followed by addition of ligand 7, 400 MHz, $CDCl_3$, 25 °C

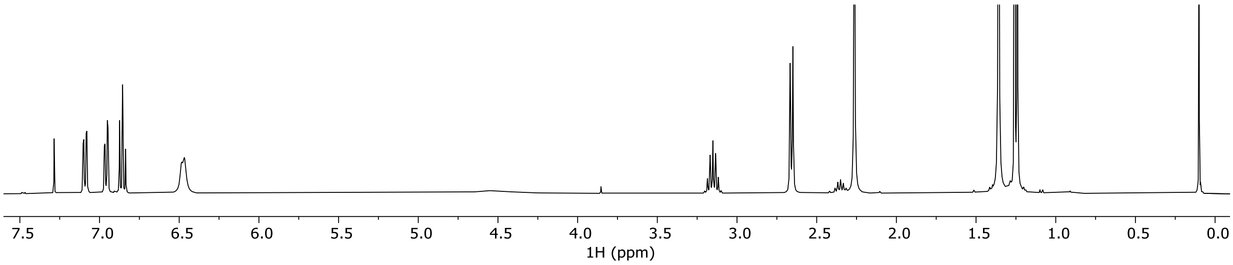

VT-NMR Studies of Different Adducts

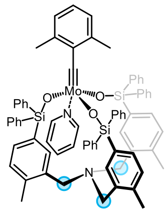

^{1}H NMR of Adduct 16b, 600 MHz, [D$_8$]-toluene/CD$_2$Cl$_2$, 323 - 233 K

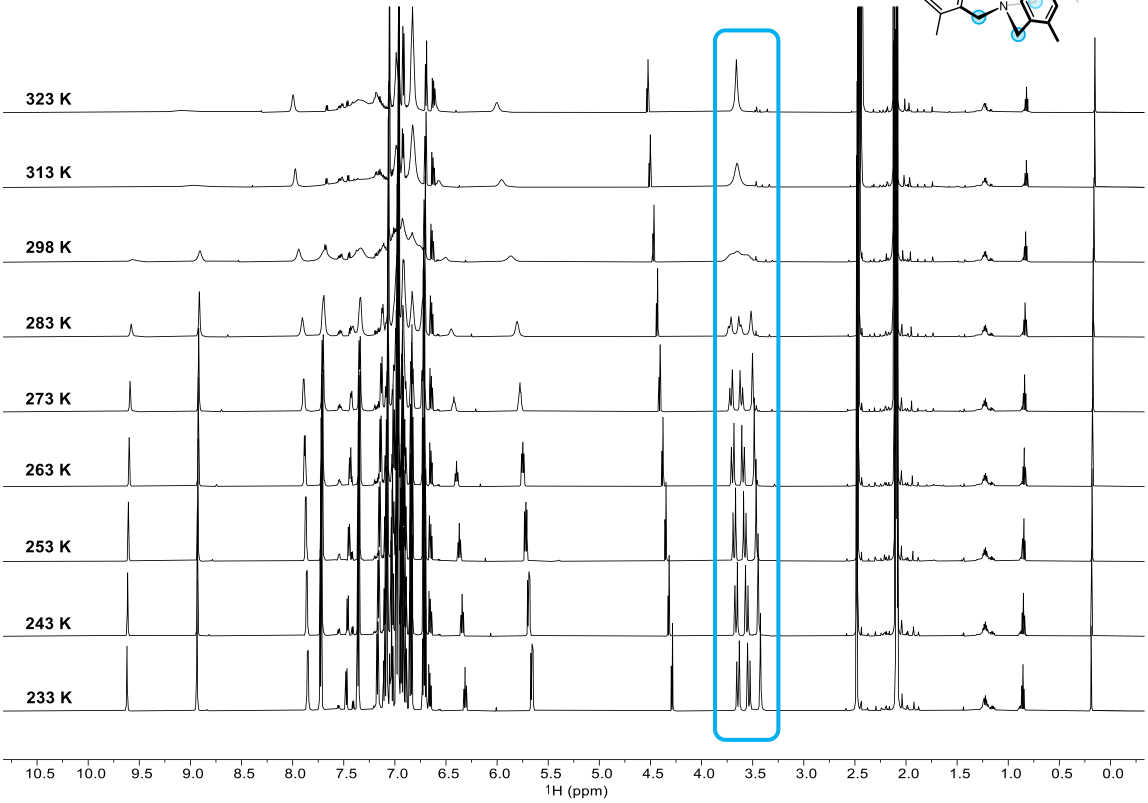

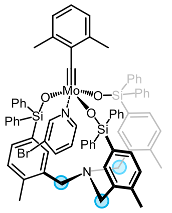

^{1}H NMR of Adduct 16c Comprising 3-Bromopyridine, 600 MHz, [D$_8$]-toluene, 323 - 233 K

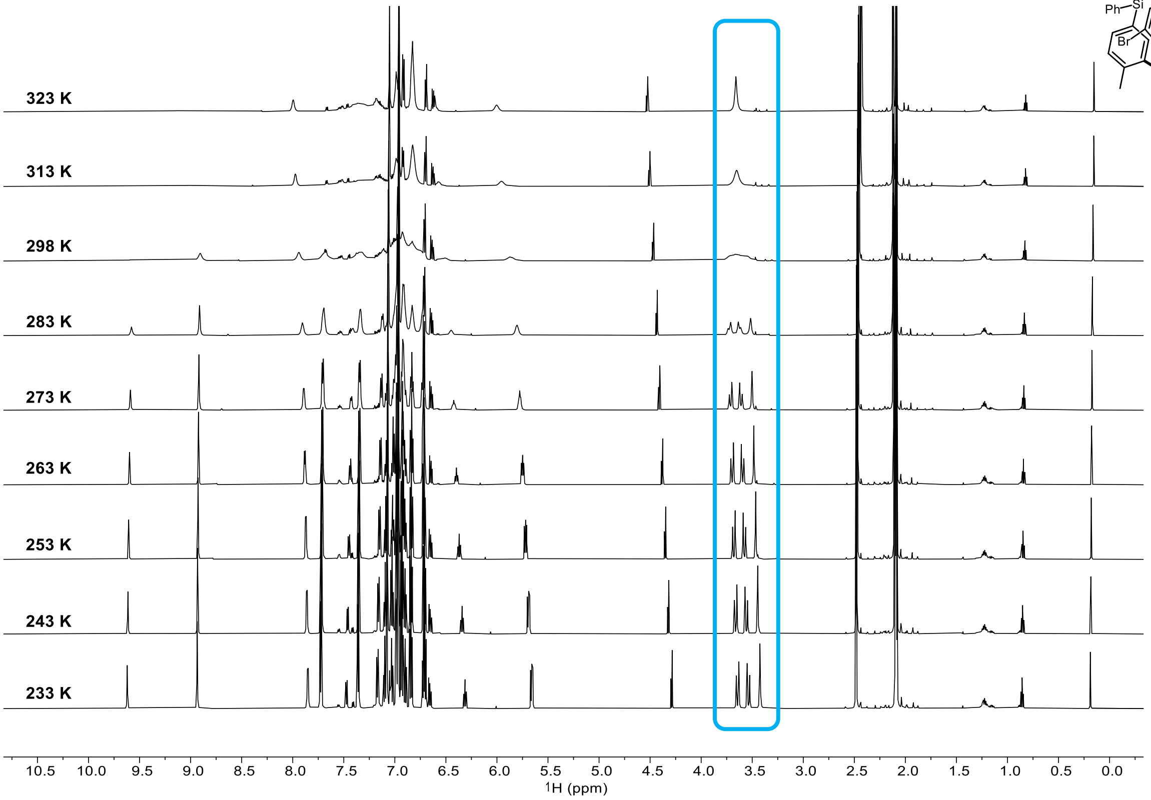

^{1}H NMR of Adduct 16d Comprising 3,5-Dibromopyridine, 600 MHz, [D$_8$]-toluene, 298 - 228 K

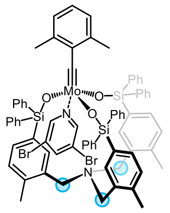

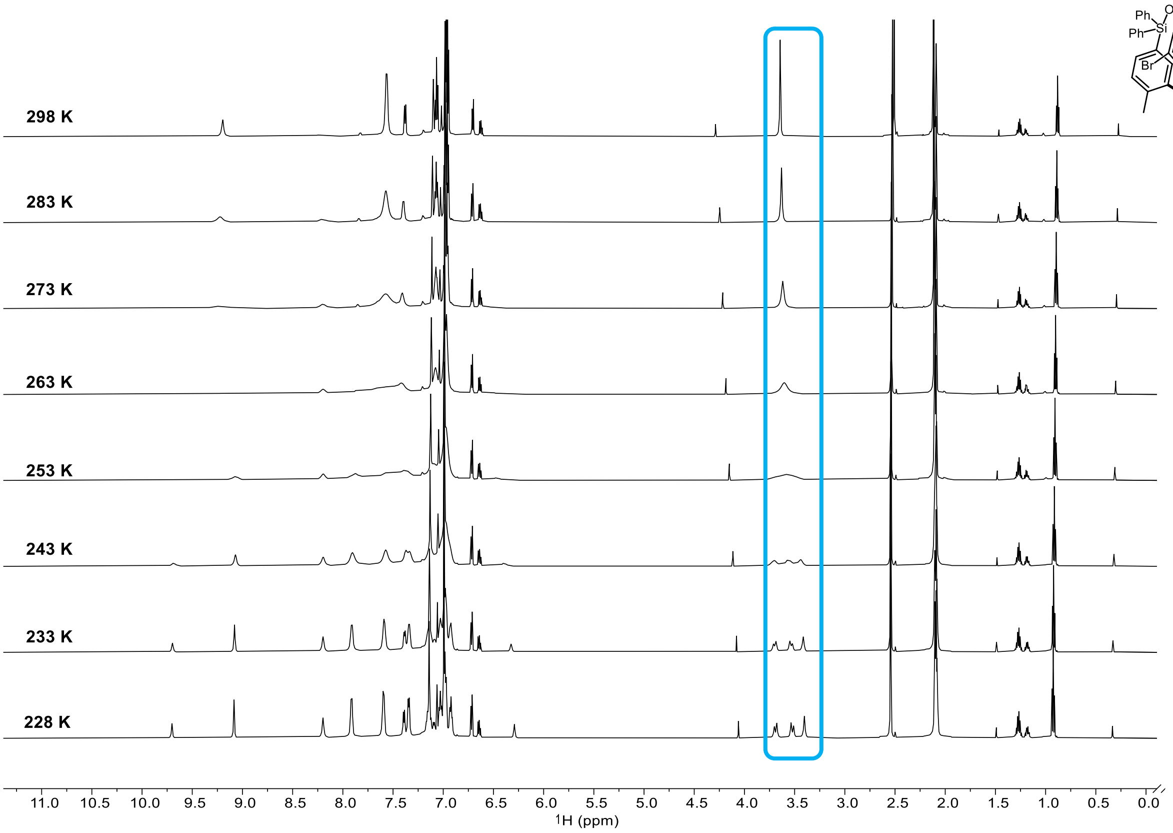

^{1}H NMR of Adduct 16e Comprising 4-Pyrrolidino-pyridine, 600 MHz, [D$_8$]-toluene, 353 - 233 K

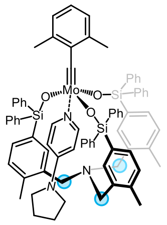

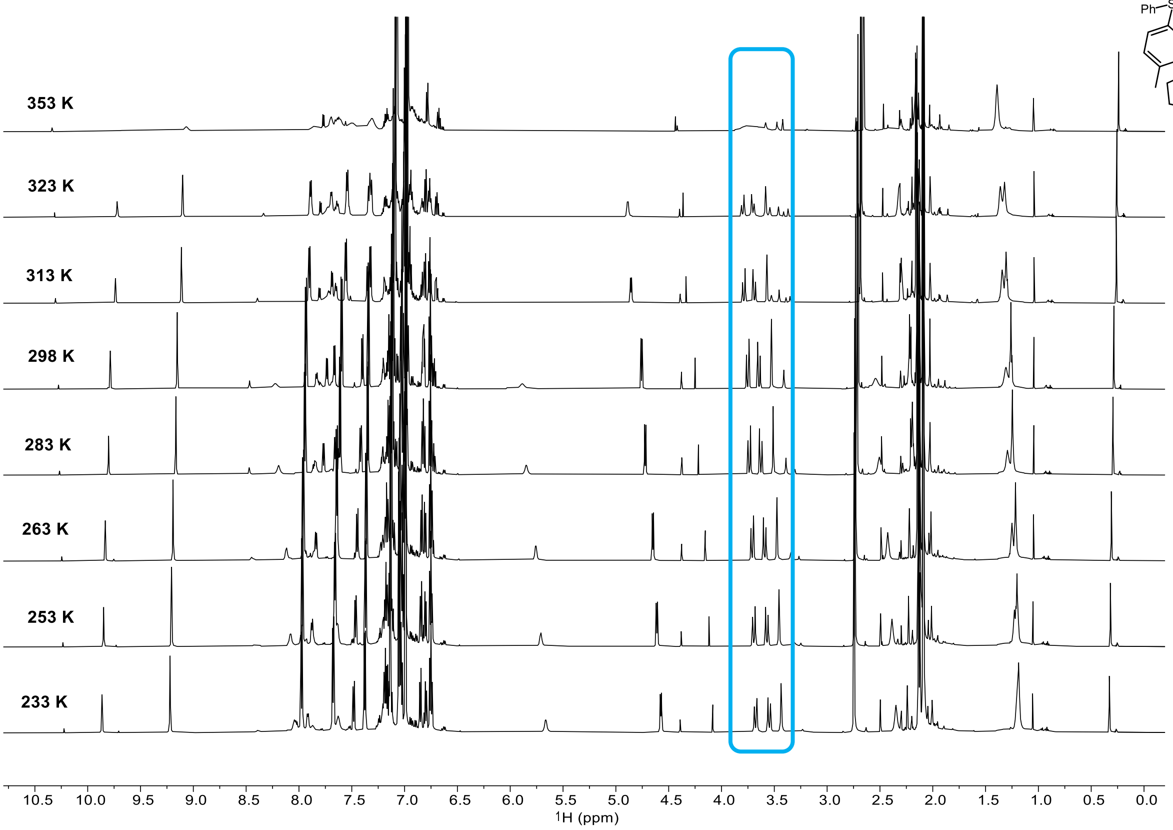

^{1}H NMR of Adduct 16f Comprising Trimethylphosphine, 600 MHz, [D8]-toluene, 333 - 233 K

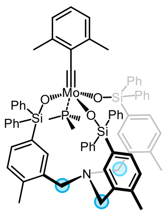

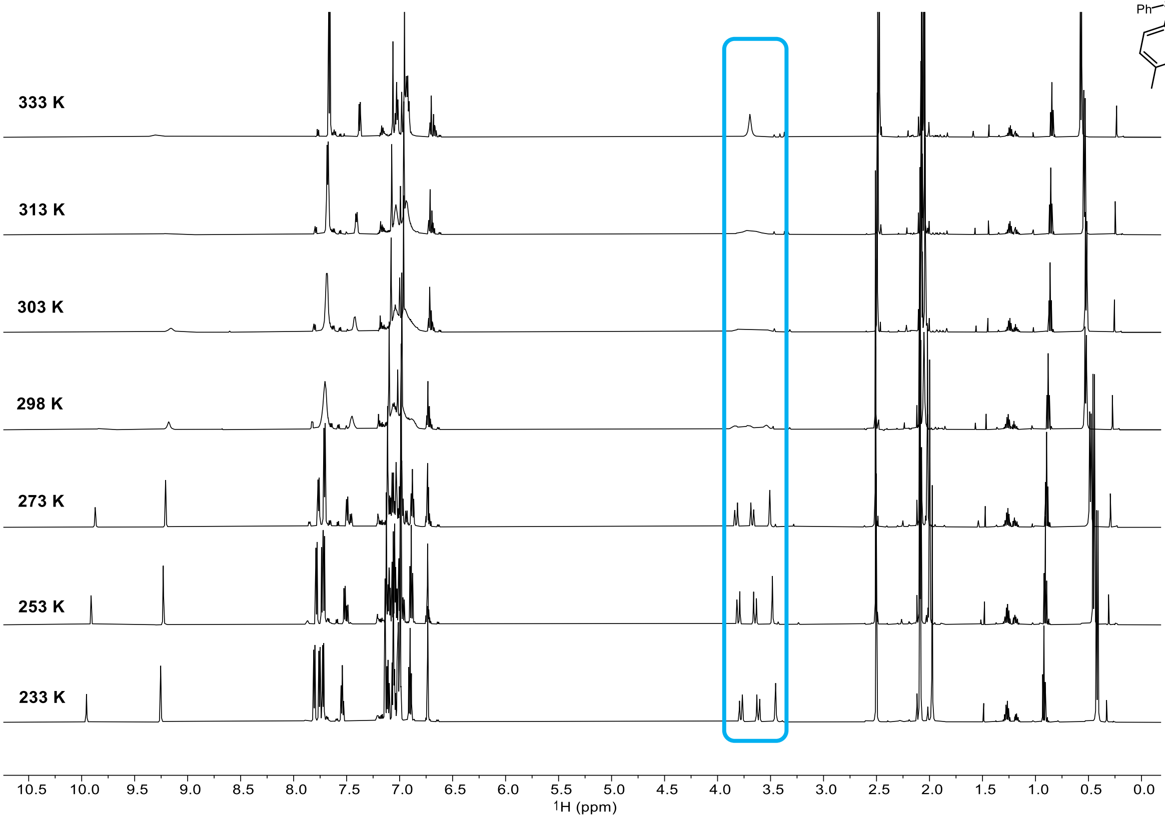

Expedited Ligand Removal

Advantage was taken of the heteroatom in the ligand backbone in order to separate products that proved to be copolar with catalyst debris formed upon work up. In a first example (compound **22f**) it sufficed to dissolve the material isolated after flash column chromatography in ethyl acetate and to wash this solution three times with aqueous HCl (3 M).

[1]**H-NMR**, 400 MHz, $CDCl_3$, 25 °C

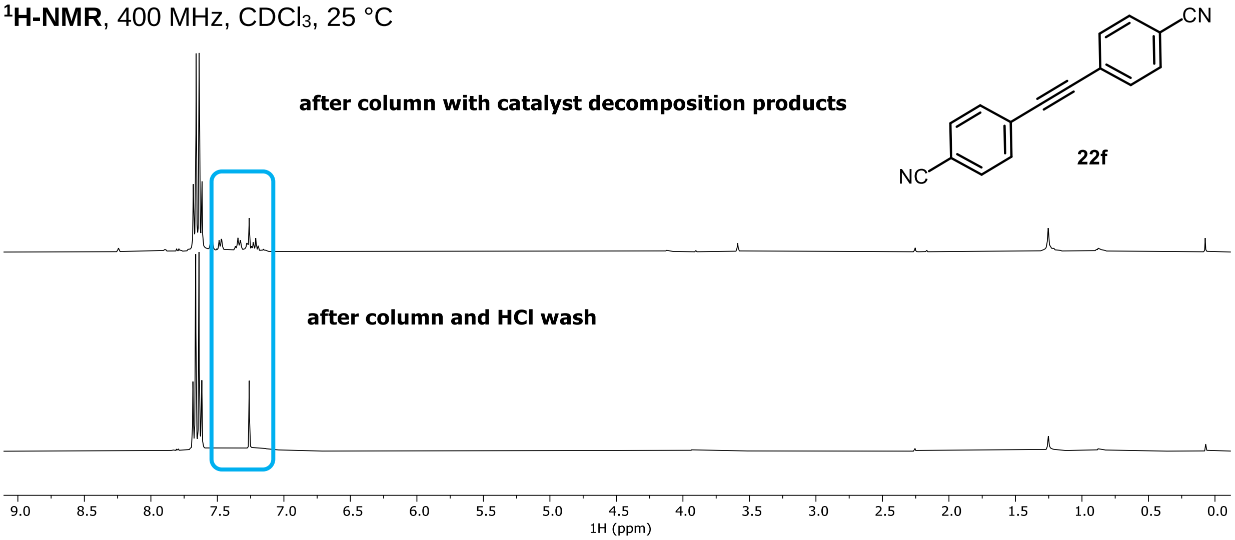

In a second example (compound **38**), the crude material was washed with aqueous HCl (3 M) prior to flash column chromatography in order to fully remove the catalyst decomposition products.

[1]**H-NMR**, 400 MHz, $CDCl_3$, 25 °C

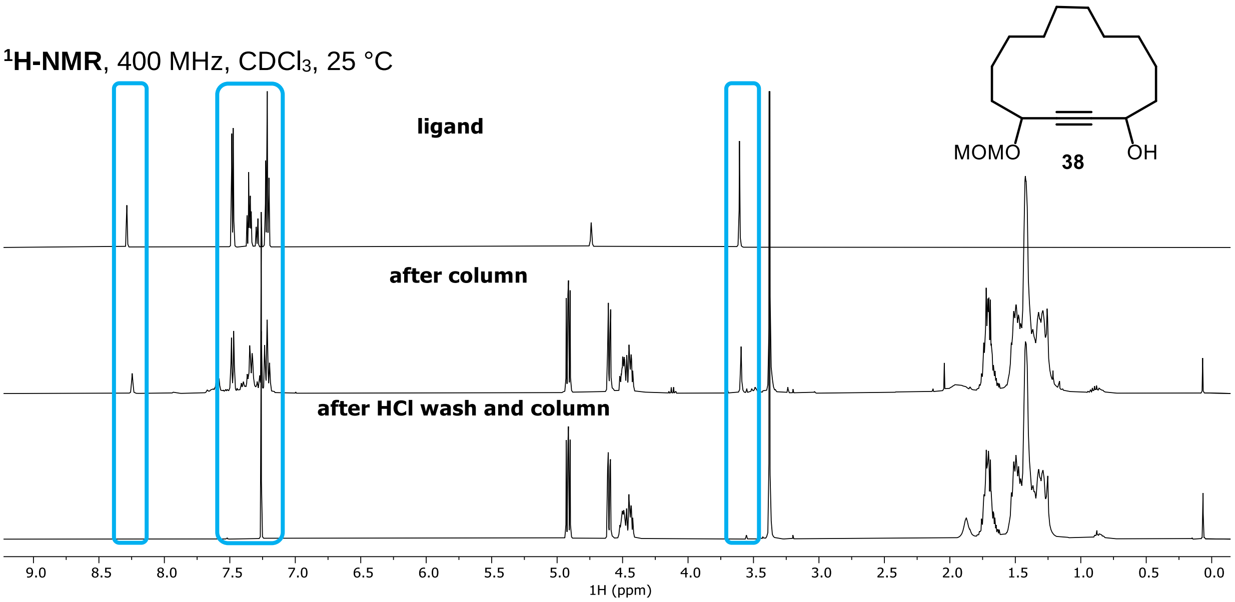

New Substrates

Diyne S13

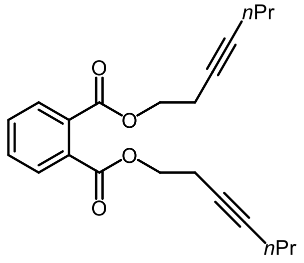

A 250 mL, one-necked flask was charged in air with phthalolyl chloride (2.00 mL, 13.8 mmol) and dichloromethane (70 mL). The solution was cooled to 0 °C before 3-hetyn-1-ol (3.36 mL, 27.6 mmol), pyridine (2.23 mL, 27.6 mmol) and 4-(dimethylamino)-pyridine (17 mg, 0.14 mmol) were added. The mixture was stirred at ambient temperature for 7 h. Water (50 mL) was added, the aqueous layer was extracted with dichloromethane (3 × 25 mL), the combined organic phases were washed with aqueous hydrogen chloride (1 M, 50 mL) and a saturated aqueous solution of the sodium bicarbonate (70 mL). The organic layer was dried over magnesium sulfate and filtered, and the solvents were evaporated. The residue was purified by flash chromatography on silica gel (hexane/ethyl acetate, 9:1) to give the title compound as a colorless oil (3.50 g, 72%). **^{1}H NMR** (400 MHz, $CDCl_3$): δ 7.74 (dd, *J* = 5.7, 3.3 Hz, 2H), 7.54 (dd, *J* = 5.7, 3.3 Hz, 2H), 4.38 (t, *J* = 7.2 Hz, 4H), 2.61 (tt, *J* = 7.2, 2.4 Hz, 4H), 2.13 (tt, *J* = 7.0, 2.4 Hz, 4H), 1.50 (h, *J* = 7.3 Hz, 4H), 0.96 (t, *J* = 7.4 Hz, 6H). **^{13}C NMR** (101 MHz, $CDCl_3$): δ 167.4, 132.1, 131.2, 129.1, 82.3, 75.5, 64.1, 22.4, 20.9, 19.3, 13.6. **IR** (film): $\tilde{\nu}$ 2962, 2933, 1724, 1456, 1382, 1340, 1268, 1120, 1079, 1061, 1039, 100, 970, 742, 704. **HRMS** (ESI) calc. for $C_{22}H_{26}O_4Na$ [M + Na]$^+$: 377.17233; found: 377.17237.

Diyne S14

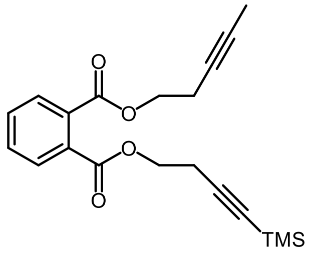

A 25 mL Schlenk flask was charged with diisopropylamine (0.12 mL, 0.84 mmol) and tetrahydrofuran (2 mL). The solution was cooled to −78 °C before a solution of *n*-butyllithium (1.6 M in hexane, 0.48 mL, 0.77 mL) was added. After 5 min, a solution of (but-3-yn-1-yl) (pent-3-yn-1-yl) phthalate (200 mg, 0.70 mmol) in tetrahydrofuran (2 mL) was added, causing a color change to yellow. After 15 min at −78 °C, a solution of trimethylchlorosilane (0.11 mL, 0.84 mL) in tetrahydrofuran (2 mL) was introduced and stirring was continued for 15 min at −78 °C. The mixture was allowed to warm to 0 °C and stirred at this temperature for 1 h. The reaction was quenched with a saturated solution of ammonium chloride (6 mL), the layers were separated, and the aqueous phase was extracted with ethyl acetate (3 × 10 mL). The combined organic layers were washed with brine (20 mL), dried over magnesium sulfate and filtered, and the solvents were evaporated. The residue was purified by flash chromatography on silica gel (hexane/ethyl acetate, 10:1) to give the title compound as a colorless oil (134 mg, 54%). **^{1}H NMR** (400 MHz, $CDCl_3$): δ 7.78 – 7.71 (m, 2H), 7.58 – 7.51 (m, 2H), 4.40 (t, *J* = 7.2 Hz, 2H), 4.37 (t, *J* = 7.0 Hz, 2H), 2.68 (t, *J* = 7.2 Hz, 2H), 2.58 (ddq, *J* = 9.6, 7.2, 2.6 Hz, 2H), 1.79 (t, *J* = 2.6 Hz, 3H), 0.15 (s, 9H). **^{13}C NMR** (101 MHz, $CDCl_3$): δ 167.3, 167.3, 132.1, 132.0, 131.3, 131.2, 129.2, 102.1, 86.9, 77.6, 74.7, 64.1, 63.4, 20.4, 19.3, 3.6, 0.1. **IR** (film): $\tilde{\nu}$ 2960, 2179, 1728, 1449, 1384, 1336, 1273, 1124, 1081, 1061, 1039, 971, 843, 760, 744, 703, 642. **HRMS** (ESI) calc. for $C_{20}H_{24}O_4SiNa$ [M + Na]$^+$: 379.13361; found: 379.13342.

Alkyne Metathesis Reactions

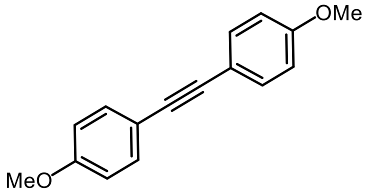

Representative Procedure for Alkyne Homo-Metathesis. Compound 22a. A 10 mL Schlenk flask was charged with 1-(4-methoxyphenyl)-1-propyne (**21**, 37 mg, 0.25 mmol), powdered molecular sieves (250 mg, 5Å) and toluene (1.25 mL). Adduct **16a** (6 mg, 5 µmol, 2 mol%) was weight out in air and added in one portion to the mixture. After stirring for 12 h at ambient temperature, the reaction was quenched with ethanol (1 mL) and the mixture was filtered through a short pad of Celite® which was carefully rinsed with ethyl acetate (20 mL) and dichloromethane (10 mL). The combined filtrates were evaporated and the residue was purified by flash chromatography on silica gel (hexane/ethyl acetate, 70:1 to 50:1) to give the title compound as a colorless solid (27 mg, 91%). **^{1}H NMR** (400 MHz, $CDCl_3$): δ 7.45 (d, *J* = 8.8 Hz, 4H), 6.87 (d, *J* = 8.8 Hz, 4H), 3.82 (s, 6H). **^{13}C NMR** (101 MHz, $CDCl_3$): δ 159.5, 133.0, 115.9, 114.1, 88.1, 55.4. The spectroscopic data was in agreement with the literature.[17]

Compound 22b

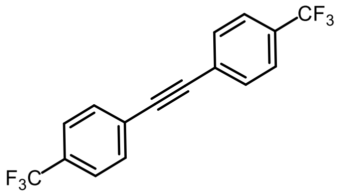

Prepared analogously with adduct **16a** as the catalyst (6 mg, 5 µmol, 2 mol%) at 50 °C. Purified by flash chromatography (hexane) to give the title compound as a colorless solid (35 mg, 89%). **^{1}H NMR** (400 MHz, $CDCl_3$): δ 7.70 – 7.59 (m, 8H). **^{13}C NMR** (101 MHz, $CDCl_3$): δ 132.1, 130.9, 126.6, 125.6 (q, *J* = 3.8 Hz), 122.2, 90.3. The spectroscopic data was in agreement with the literature.[18]

Compound 22c.

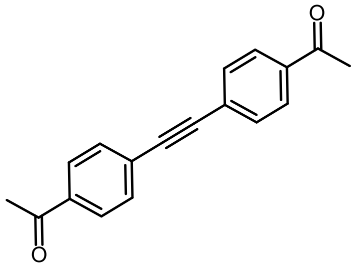

Prepared analogously with adduct **16a** as the catalyst (6 mg, 5 µmol, 2 mol%) at 50 °C. Purified by flash chromatography (hexane/ethyl acetate, 8:2 to 1:1) to give the title compound as a colorless solid (28 mg, 85%). **^{1}H NMR** (300 MHz, $CDCl_3$): δ 7.95 (d, *J* = 8.0 Hz, 4H), 7.63 (d, *J* = 8.1 Hz, 4H), 2.61 (s, 6H). **^{13}C NMR** (101 MHz, $CDCl_3$): δ 197.1, 136.6, 131.6, 128.1, 127.5. 91.5, 26.5. The spectroscopic data was in agreement with the literature.[19]

Compound 22d

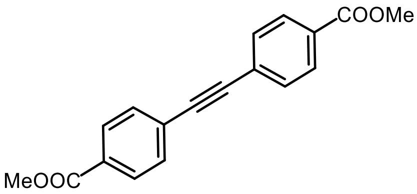

Prepared analogously with adduct **16a** as the catalyst (6 mg, 5 µmol, 2 mol%) at 50 °C. Purified by flash chromatography (hexane/dichloromethane, 1:1) to give the title compound as a colorless solid (36 mg, 99%).**^{1}H NMR** (400 MHz, $CDCl_3$): δ 8.04 (d, *J* = 8.5 Hz, 4H), 7.60 (d, *J* = 8.6 Hz, 4H), 3.93 (s, 6H). **^{13}C NMR** (101 MHz, $CDCl_3$): δ 166.6, 131.8, 130.1, 129.7, 127.5, 91.5, 52.4. The spectroscopic data was in agreement with the literature.[14]

Compound 22e

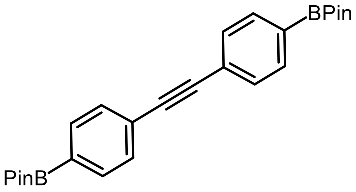

Prepared analogously with adduct **16a** as the catalyst (6 mg, 5 µmol, 2 mol%) at 50 °C. Purified by flash chromatography (hexane/dichloromethane, 10:1 to 8:2) to give the title compound as a colorless solid (38 mg, 71%). **^{1}H NMR** (400 MHz, $CDCl_3$): δ 7.79 (d, *J* = 8.2 Hz, 4H), 7.53 (d, *J* = 8.2 Hz, 4H), 1.35 (s, 24H). **^{13}C NMR** (101 MHz, $CDCl_3$): δ 134.7, 131.0, 126.0, 91.0, 84.1, 25.0 [Note: no signal was observed for the quaternary carbons directly bonded to boron]. **IR** (film): $\tilde{\nu}$ 2975, 1608, 1399, 1356, 1322, 1273, 1263, 1166, 1141, 1088, 1018, 962, 909, 859, 836, 739, 670, 652. **HRMS** (ESI) calc. for $C_{26}H_{32}B_2O_4Na$ $[M + Na]^+$: 453.23794; found: 453.23815. The spectroscopic data was in agreement with the literature.[20]

Compound 22f

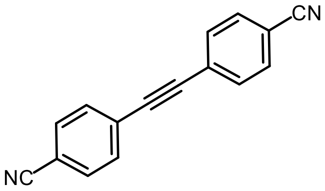

Prepared analogously with adduct **16a** as the catalyst (15 mg, 13 µmol, 5 mol%) at 50 °C. Purified by flash chromatography (hexane/ethyl acetate 12:1 to 3:2 to 1:1) to give crude product containing the co-polar ligand. To remove the impurity, the crude product was dissolved in ethyl acetate (30 mL) and the organic layer washed with aqueous hydrogen chloride (3 M, 3 × 10 mL). The organic layer was dried over magnesium sulfate and filtered, and the solvents were evaporated to give the title compound as a colorless solid (26 mg, 91%). **^{1}H NMR** (400 MHz, $CDCl_3$): δ 7.67 (d, *J* = 8.6 Hz, 4H), 7.63 (d, *J* = 8.7 Hz, 4H). **^{13}C NMR** (101 MHz, $CDCl_3$): δ 132.4, 132.3, 127.2, 118.4, 112.6, 91.7. The spectroscopic data was in agreement with the literature.[21]

Compound 22g

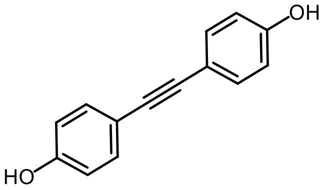

Prepared analogously with adduct **16a** as the catalyst (15 mg, 13 µmol, 5 mol%) at 50 °C. Purified by flash chromatography (hexane/ethyl acetate, 8:2 to 1:1) to give the title compound as a red solid (36 mg, 98%). **^{1}H NMR** (400 MHz, CD_3CN): δ 7.35 (d, *J* = 8.8 Hz, 4H), 7.27 (bs, 2H), 6.81 (d, *J* = 8.7 Hz, 4H). **^{13}C NMR** (101 MHz, CD_3CN): δ 157.8, 133.7, 116.3, 115.6, 88.3. The spectroscopic data was in agreement with the literature.[2]

Compound 22h

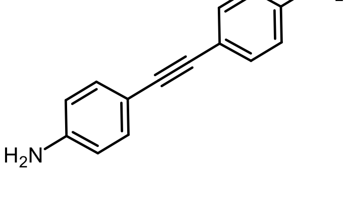

Prepared analogously with complex **17** as the catalyst (10 mg, 13 µmol, 5 mol%) at 110 °C. Purified by flash chromatography (hexane/ethyl acetate, 3:1 to 1:1) to give the title compound as an orange solid (24 mg, 71%). **^{1}H NMR** (400 MHz, $CDCl_3$): δ 7.30 (d, *J* = 8.6 Hz, 4H), 6.62 (d, *J* = 8.5 Hz, 4H), 3.77 (s, 4H). **^{13}C NMR** (101 MHz, $CDCl_3$): δ 146.3, 132.8, 114.9, 113.5, 87.9. The spectroscopic data was in agreement with the literature.[2]

Compound 22i

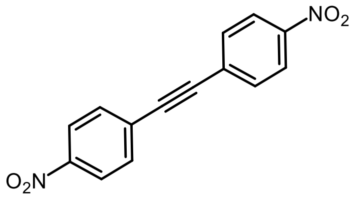

Prepared analogously with complex **17** as the catalyst (15 mg, 17 µmol, 7 mol%) at 110 °C. Purified by flash chromatography (hexane/ethyl acetate, 4:1) to give the title compound as a yellow solid (15 mg, 45%). **^{1}H NMR** (400 MHz, $CDCl_3$): δ 8.26 (d, *J* = 8.9 Hz, 4H), 7.72 (d, *J* = 8.8 Hz, 4H). **^{13}C NMR** (101 MHz, $CDCl_3$): δ 147.8, 132.8, 129.0, 123.9, 92.1. **HRMS** (EI) calc. for $C_{14}H_8N_2O_4$ $[M]^+$: 268.04786; found: 258.04825. The spectroscopic data was in agreement with the literature.[22]

Compound 23

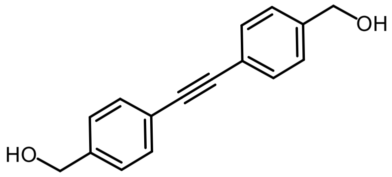

Prepared analogously with adduct **16a** as the catalyst (15 mg, 13 µmol, 5 mol%) at 50 °C. Purified by flash chromatography (dichloromethane/methanol 98:2) to give the title compound as a colorless solid (24 mg, 81%). **^{1}H NMR** (400 MHz, [D_6]-DMSO): δ 7.50 (d, *J* = 8.2 Hz, 4H), 7.36 (d, *J* = 8.1 Hz, 4H), 5.28 (t, *J* = 5.7 Hz, 2H), 4.53 (d, *J* = 5.7 Hz, 4H). **^{13}C NMR** (101 MHz, [D_6]-DMSO): δ 143.3, 131.1, 126.6, 120.5, 89.0, 62.5. The spectroscopic data was in agreement with the literature.[23]

Compound 24

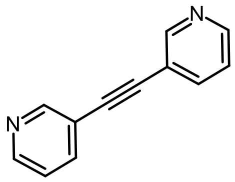

Prepared analogously with adduct **16a** as the catalyst (6 mg, 5 µmol, 2 mol%) at 90 °C. Purified by flash chromatography (hexane/ethyl acetate, 3:2 to 1:1) to give the title compound as a yellowish solid (20 mg, 89%). **^{1}H NMR** (400 MHz, $CDCl_3$): δ 8.78 (dd, *J* = 2.2, 0.9 Hz, 2H), 8.58 (dd, *J* = 4.9, 1.7 Hz, 2H), 7.83 (dt, *J* = 7.9, 1.9 Hz, 2H), 7.31 (ddd, *J* = 7.9, 4.9, 0.9 Hz, 2H). **^{13}C NMR** (101 MHz, $CDCl_3$): δ 152.5, 149.2, 138.7, 123.2, 119.9, 89.3. The spectroscopic data was in agreement with the literature.[19]

Compound 25

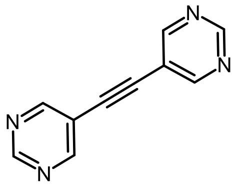

Prepared analogously with adduct **16a** as the catalyst (6 mg, 5 µmol, 2 mol%) at 100 °C. Purified by flash chromatography (dichloromethane/methanol, 95:5) to give the title compound as a colorless solid (10 mg, 44%). **^{1}H NMR** (400 MHz, $CDCl_3$): δ 9.21 (s, 2H), 8.91 (s, 4H). **^{13}C NMR** (101 MHz, $CDCl_3$): δ 159.0, 157.7, 118.7, 89.2. The spectroscopic data was in agreement with the literature.[24]

Compound 26

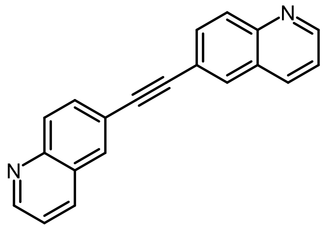

Prepared analogously with adduct **16a** as the catalyst (6 mg, 5 µmol, 2 mol%) at 50 °C. Purified by flash chromatography (dichloromethane/methanol, 99:1 to 98:2) to give the title compound as a colorless solid (28 mg, 80%). **^{1}H NMR** (400 MHz, $CDCl_3$): δ 8.97 – 8.91 (m, 2H), 8.16 (dd, *J* = 8.4, 1.7 Hz, 2H), 8.13 – 8.04 (m, 4H), 7.86 (dd, *J* = 8.7, 1.9 Hz, 2H), 7.44 (dd, *J* = 8.3, 4.2 Hz, 2H). **^{13}C NMR** (101 MHz, $CDCl_3$): δ 151.3, 147.9, 135.9, 132.3, 131.5, 129.9, 128.2, 122.0, 121.4, 90.4. **HRMS** (EI) calc. for $C_{20}H_{12}N_2$ $[M]^+$: 280.09950; found: 280.09974. The spectroscopic data was in agreement with the literature.[25]

Compound 27

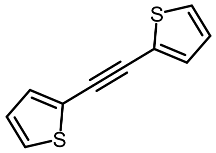

Prepared analogously with adduct **16a** as the catalyst (6 mg, 5 μmol, 2 mol%) at 50 °C. Purified by flash chromatography (hexane/dichloromethane, 100:1) to give the title compound as a colorless solid (21 mg, 88%). **^{1}H NMR** (400 MHz, $CDCl_3$): δ 7.31 (dd, *J* = 5.2, 1.2 Hz, 2H), 7.28 (dd, *J* = 3.7, 1.2 Hz, 2H), 7.01 (dd, *J* = 5.2, 3.6 Hz, 2H). **^{13}C NMR** (101 MHz, $CDCl_3$): δ 132.3, 127.8, 127.3, 123.1, 86.3. The spectroscopic data was in agreement with the literature.[19]

Compound 28

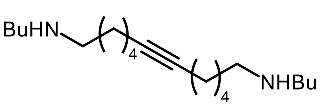

Prepared analogously with adduct **16a** as the catalyst (15 mg, 13 μmol, 5 mol%) at 50 °C. Purified by flash chromatography (dichloromethane/methanol, 99:1 to 95:5 with 2% triethylamine) to give the title compound as a brown oil (31 mg, 80%). **^{1}H NMR** (400 MHz, CD_2Cl_2): δ 2.64 – 2.47 (m, 8H), 2.20 – 2.06 (m, 4H), 1.71 (bs, 2H), 1.55 – 1.29 (m, 20H), 0.90 (t, *J* = 7.3 Hz, 6H). **^{13}C NMR** (101 MHz, CD_2Cl_2): δ 80.4, 50.3, 50.1, 32.7, 30.1, 29.5, 27.0, 20.9, 19.0, 14.2. **HRMS** (ESI) calc. for $C_{20}H_{41}N_2$ $[M + H]^+$: 309.32643; found: 309.32658. The spectroscopic data was in agreement with the literature.[18]

Compound 29

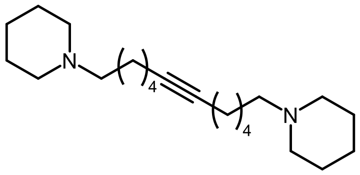

Prepared analogously with adduct **16a** as the catalyst (6 mg, 5 μmol, 2 mol%) at 50 °C. Purified by flash chromatography (hexane/ethyl acetate, 3:1 with trimethylamine 5%) to give the title compound as a yellow oil (35 mg, 80%). **^{1}H NMR** (400 MHz, $CDCl_3$): δ 2.37 (d, *J* = 14.1 Hz, 8H), 2.27 (dd, *J* = 9.2, 6.5 Hz, 4H), 2.18 – 2.09 (m, 4H), 1.57 (p, *J* = 5.6 Hz, 8H), 1.53 – 1.29 (m, 16H). **^{13}C NMR** (101 MHz, $CDCl_3$): δ 80.3, 59.6, 54.8, 29.2, 27.1, 26.6, 26.1, 24.6, 18.8. The spectroscopic data was in agreement with the literature.[2]

Compound 30

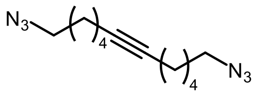

Prepared analogously with adduct **16a** as the catalyst (6 mg, 5 μmol, 2 mol%) at 50 °C. Purified by flash chromatography (hexane/ethyl acetate, 50:1) to give the title compound as a clear oil (24 mg, 77%). **^{1}H NMR** (400 MHz, $CDCl_3$): δ 3.27 (t, *J* = 6.9 Hz, 4H), 2.16 (td, *J* = 5.9, 2.0 Hz, 4H), 1.68 – 1.56 (m, 4H), 1.55 – 1.40 (m, 8H). **^{13}C NMR** (101 MHz, $CDCl_3$): δ 80.2, 51.5, 28.7, 28.5, 26.0, 18.7. The spectroscopic data was in agreement with the literature.[2]

Compound 32

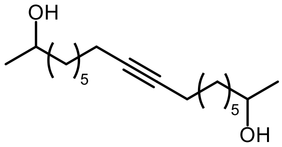

Prepared analogously with adduct **16a** as the catalyst (15 mg, 13 μmol, 5 mol%) at 50 °C. Purified by flash chromatography (dichloromethane/methanol, 98:2 to 90:10) to give the title compound as a yellow oil (29 mg, 82%). **^{1}H NMR** (400 MHz, $CDCl_3$): δ 3.82 – 3.72 (m, 2H), 2.17 – 2.08 (m, 4H), 1.62 (s(br), 2H), 1.52 – 1.24 (m, 20H), 1.17 (d, *J* = 6.2 Hz, 6H). **^{13}C NMR** (101 MHz,

CDCl3): δ 80.4, 68.2, 39.4, 29.3, 29.2, 28.9, 25.8, 23.6, 18.8. The spectroscopic data was in agreement with the literature.[2]

Compound 33a

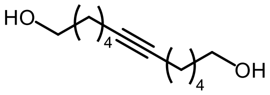

Prepared analogously with adduct **16a** as the catalyst (15 mg, 13 µmol, 5 mol%) at 50 °C using 4 Å molecular sieves (250 mg) instead of 5 Å molecular sieves. Purified by flash chromatography (hexane/ethyl acetate, 1:1) to give the title compound as a colorless solid (16 mg, 65%). **^{1}H NMR** (400 MHz, $CDCl_3$): δ 3.64 (t, *J* = 6.4 Hz, 4H), 2.23 – 2.11 (m, 4H), 1.69 (s, 2H), 1.63 – 1.39 (m, 12H). **^{13}C NMR** (101 MHz, $CDCl_3$): δ 80.3, 63.0, 32.4, 28.9, 25.1, 18.8. **HRMS** (ESI) calc. for $C_{12}H_{23}O_2$ [M + H]$^+$: 199.16962; found: 199.16956. The spectroscopic data was found to be in agreement with the data reported in the literature.[26]

Compound 33b

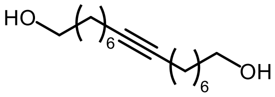

Prepared analogously with adduct **16a** as the catalyst (15 mg, 13 µmol, 5 mol%) at 50 °C. Purified by flash chromatography (hexane/ethyl acetate, 2:3) to give the title compound as a colorless solid (25 mg, 79%). **^{1}H NMR** (400 MHz, $CDCl_3$): δ 3.63 (t, *J* = 6.6 Hz, 4H), 2.18 – 2.09 (m, 4H), 1.62 – 1.28 (m, 22H). **^{13}C NMR** (101 MHz, $CDCl_3$): δ 80.4, 63.1, 32.9, 29.15, 29.09, 28.9, 25.8, 18.8. **HRMS** (ESI) calc. for $C_{16}H_{30}O_2Na$ [M + Na]$^+$: 277.21387; found: 277.21381. The spectroscopic data was in agreement with the literature.[2]

Compound 31

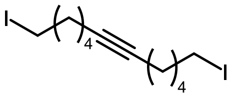

A 10 mL Schlenk flask was charged with 8-iodooct-2-yne (59 mg, 0.25 mmol), powdered molecular sieves (250 mg, 5Å) and toluene (1.15 mL). Adduct **16a** (6 mg, 5 µmol, 2 mol%) was weight out in air and added in one portion. A stock solution of triphenylborane (0.01 M in toluene, 0.5 mL, 5 µmol, 2 mol%) was added and the mixture was stirred for 1 h at ambient temperature. The reaction was quenched with ethanol (1 mL) and the mixture was filtered through a short pad of Celite® which was carefully rinsed with ethyl acetate (20 mL) and dichloromethane (10 mL). The solvents were evaporated and the residue was purified by flash chromatography on silica (hexane/ethyl acetate, 95:5) to give the title compound as a yellow oil (48 mg, 82%). **^{1}H NMR** (400 MHz, $CDCl_3$): δ 3.20 (t, *J* = 7.0 Hz, 4H), 2.16 (ddt, *J* = 5.1, 3.5, 2.0 Hz, 4H), 1.92 – 1.78 (m, 4H), 1.58 – 1.43 (m, 8H). **^{13}C NMR** (101 MHz, $CDCl_3$): δ 80.2, 33.2, 29.8, 28.1, 18.7, 7.0. The spectroscopic data was in agreement with the literature.[18]

Representative Procedure for Ring Closing Alkyne Metathesis. Compound 35. A 100 mL Schlenk

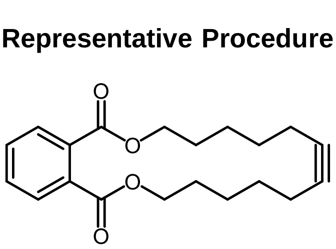

flask was charged with di(oct-6-yn-1-yl) phthalate (38 mg, 0.10 mmol), powdered molecular sieves (250 mg, 5Å) and toluene (50 mL). Adduct **16a** (6 mg, 5 µmol, 5 mol%) was weight out in air and added in one portion, and the mixture was stirred for 12 h at ambient temperature. The reaction was quenched with ethanol (5 mL) and the mixture was filtered through a short pad of Celite® which was carefully rinsed with ethyl acetate (20 mL) and dichloromethane (10 mL). The solvents were evaporated and the residue was purified by flash chromatography on silica gel (hexane/ethyl acetate, 10:1) to give the title compound as a yellow oil (30 mg, 91%). **^{1}H NMR** (400 MHz, $CDCl_3$): δ 7.73 (dd, *J* = 5.7, 3.3 Hz,

2H), 7.53 (dd, J = 5.7, 3.3 Hz, 2H), 4.35 (t, J = 6.0 Hz, 4H), 2.23 – 2.15 (m, 4H), 1.78 (dq, J = 9.2, 5.8 Hz, 4H), 1.63 – 1.44 (m, 8H). **^{13}C NMR** (101 MHz, $CDCl_3$): δ 168.0, 132.4, 131.1, 129.0, 80.7, 66.5, 28.5, 28.1, 26.0, 18.7. The spectroscopic data was in agreement with the literature.[19]

Compound 34

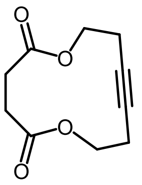

Prepared analogously either at ambient temperature with adduct **16a** as the catalyst (6 mg, 5 µmol, 5 mol%) or at 50 °C with adduct **16a** (2 mg, 2 µmol, 2 mol%). Purified by flash chromatography (hexane/ethyl acetate, 9:1) to give the title compound as a colorless oil (19 mg, 96% or 19 mg, 97%). **^{1}H NMR** (400 MHz, $CDCl_3$): δ 4.31 – 4.22 (m, 4H), 2.69 (s, 4H), 2.51 – 2.42 (m, 4H). **^{13}C NMR** (101 MHz, $CDCl_3$): δ 171.9, 78.9, 61.5, 30.1, 19.8. The spectroscopic data was in agreement with the literature.[19]

Compound 36

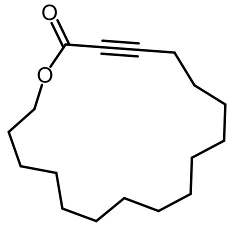

Prepared analogously with adduct **16a** as the catalyst (8.5 mg, 7 µmol, 10 mol%) at reflux over 2 h. Purified by flash chromatography (hexane/ethyl acetate, 30:1) to give the title compound as a colorless solid (26 mg, 98%). **^{1}H NMR** (400 MHz, $CDCl_3$): δ 4.27 – 4.18 (m, 2H), 2.41 (dd, J = 6.7, 4.8 Hz, 2H), 1.72 – 1.61 (m, 2H), 1.55 – 1.25 (m, 22H). **^{13}C NMR** (101 MHz, $CDCl_3$): δ 154.2, 89.1, 74.0, 65.7, 28.6, 28.3, 28.11, 28.09, 27.74, 27.72, 27.2, 27.1, 26.8, 26.7, 26.5, 18.6. The spectroscopic data was in agreement with the literature.[27]

Compound 37

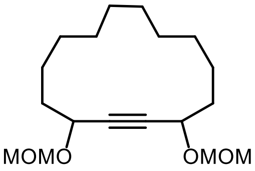

Prepared analogously with complex **17b** as the catalyst (17 mg, 20 µmol, 20 mol%) at reflux over 4 h. The complex was dissolved in a separate Schlenk flask in toluene (1 mL) and the solution added dropwise to the solution of the diyne at 110 °C. The crude product was purified by flash chromatography (hexane/ethyl acetate, 10:1 to 7:3) to give the title compound as a yellow oil (24 mg, 77%, mixture of two diastereomers). **^{1}H NMR** (400 MHz, $CDCl_3$): δ 4.91 (dd, J = 6.7, 3.3 Hz, 2H), 4.59 (dd, J = 6.8, 2.9 Hz, 2H), 4.48 – 4.40 (m, 2H), 3.37 (s, 6H), 1.79 – 1.65 (m, 4H), 1.59 – 1.22 (m, 16H). **^{13}C NMR** (101 MHz, $CDCl_3$): δ 94.33, 94.31, 84.8, 84.6, 66.3, 66.2, 55.7, 35.1, 35.0, 26.6, 26.5, 25.9, 25.8, 23.9, 23.8, 22.5, 22.4. **HRMS** (ESI) calc. for $C_{18}H_{32}O_4Na$ [M + Na]$^+$: 335.21928; found: 335.21918. The spectroscopic data was in agreement with the literature.[2]

Compound 38

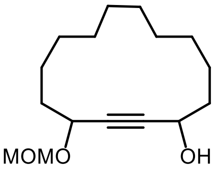

Prepared analogously with complex **16a** as the catalyst (23 mg, 20 µmol, 20 mol%) at reflux over 4 h. The catalyst was dissolved in a separate Schlenk flask in toluene (1 mL) and the solution added dropwise to the solution of the diyne at 110 °C. The crude product was washed with an aqueous HCl (3 M, 3 × 10 mL), the organic phase was dried over magnesium sulfate and filtered, and the solvents were evaporated. Purification of the residue by flash chromatography (toluene/*tert*-butyl methyl ether, 8:2) gave the title compound as a colorless solid (16 mg, 60%, mixture of two diastereomers). **^{1}H NMR** (400 MHz, $CDCl_3$): δ 4.92 (dd, J = 6.8, 5.1 Hz, 1H), 4.60 (dd, J = 6.8, 1.7 Hz, 1H), 4.54 – 4.41 (m, 2H), 3.38 (d, J = 0.7 Hz, 3H), 1.90 – 1.82

(m, 1H), 1.88 (bs, 1H), 1.77 – 1.63 (m, 4H), 1.57 – 1.17 (m, 14H). **^{13}C NMR** (101 MHz, $CDCl_3$): δ 94.3, 87.2, 87.1, 84.1, 83.8, 66.3, 66.2, 63.00, 62.96, 55.7, 37.2, 37.1, 35.0, 34.9, 26.6, 26.5, 25.9, 25.84, 25.79, 25.76, 23.93, 23.91, 23.8, 22.5, 22.4, 22.35, 22.33. **HRMS** (ESI) calc. for $C_{16}H_{28}NaO_3$ $[M + Na]^+$: 291.19307; found: 291.19290. The spectroscopic data was in agreement with the literature.[2]

Compound 39

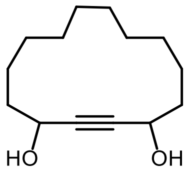

Prepared analogously with complex **16a** as the catalyst (23 mg, 20 µmol, 20 mol%) at reflux temperature. The complex was dissolved in a separate Schlenk flask in toluene (1 mL) and the solution was added dropwise to the solution of the diyne at 110 °C. The crude product was purified by flash chromatography (hexane/ethyl acetate 9:1) to give the title compound as a colorless solid (10 mg, 46%, mixture of two diastereomers). **^{1}H NMR** (400 MHz, $CDCl_3$): δ 4.54 – 4.45 (m, 2H), 1.81 – 1.59 (m, 4H), 1.57 – 1.17 (m, 16H). **^{13}C NMR** (101 MHz, $CDCl_3$): δ 86.5, 86.3, 63.1, 63.0, 37.2, 37.1, 26.54, 26.51, 25.79, 25.75, 23.9, 22.42, 22.37. **HRMS** (CI) calc. for $C_{14}H_{28}NO_2$ $[M + NH_4]^+$: 242.21145; found: 242.21154. The spectroscopic data was in agreement with the literature.[2]

Compound 48

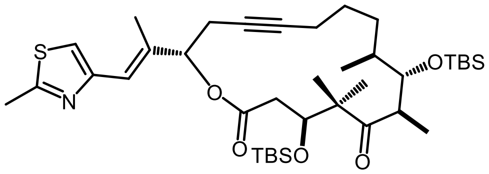

A 50 mL Schlenk flask was charged with diyne **47** (10 mg, 13 µmol), powdered molecular sieves (250 mg, 5Å) and toluene (7 mL). Adduct **16a** (1 mg, 0.5 µmol, 5 mol%) was weight out in air and dissolved in toluene (1 mL). The catalyst solution was added dropwise to the solution of the substrate at 60 °C and stirring was continued for 2 h at this temperature. The reaction was quenched with ethanol (5 mL) before the mixture was cooled to ambient temperature and filtered through a short pad of Celite® which was carefully rinsed with ethyl acetate (20 mL) and dichloromethane (10 mL). The solvents were evaporated and the residue was purified by flash chromatography on silica (pentane/diethyl ether, 10:1 to 7:3) to give the title compound as a colorless solid (8 mg, 81%). **^{1}H NMR** (400 MHz, $CDCl_3$): δ 6.97 (s, 1H), 6.56 (s, 1H), 5.34 (dd, J = 7.4, 3.4 Hz, 1H), 4.70 (dd, J = 6.4, 4.9 Hz, 1H), 3.93 (dd, J = 6.6, 1.9 Hz, 1H), 3.24 (p, J = 6.9 Hz, 1H), 2.84 – 2.74 (m, 1H), 2.72 (s, 3H), 2.70 – 2.54 (m, 3H), 2.17 (d, J = 1.3 Hz, 3H), 2.15 – 2.06 (m, 4H), 1.17 (s, 3H), 1.15 (s, 3H), 1.11 (s, 3H), 0.94 (s, 3H), 0.91 (s, 9H), 0.87 (s, 9H), 0.10 (s, 6H), 0.09 (s, 3H), 0.07 (s, 6H). **^{13}C NMR** (101 MHz, $CDCl_3$): δ 216.7, 170.2, 165.1, 152.4, 137.1, 120.5, 116.9, 82.4, 78.1, 77.4, 76.5, 72.8, 54.7, 44.6, 41.8, 39.1, 29.8, 26.4, 26.2, 26.1, 24.3, 21.2, 20.7, 19.4, 18.8, 18.7, 18.5, 17.1, 15.2, 1.2, –3.1, –3.6, –3.90, –3.92. **HRMS** (ESI) calc. for $C_{38}H_{65}NO_5Si_2SNa$ $[M + Na]^+$: 726.40142; found: 726.40156. The spectroscopic data was in agreement with the literature.[28]

Compound 50

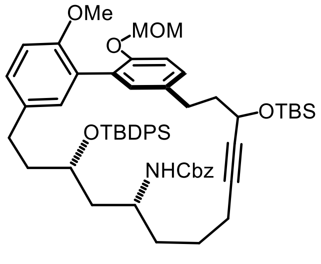

A 50 mL Schlenk flask was charged with diyne **49** (10 mg, 10 µmol), powdered molecular sieves (250 mg, 5Å) and toluene (5 mL). The mixture was heated to 50 °C. Adduct **16a** (0.5 mg, 0.5 µmol, 5 mol%) was weight out in air and added in one portion. After stirring for 1.5 h at 50 °C, the reaction was quenched with ethanol (5 mL) before the mixture was cooled to ambient temperature and filtered through a short pad of Celite® which was

carefully rinsed with ethyl acetate (20 mL) and dichloromethane (10 mL). The solvents were evaporated and the residue was purified by flash chromatography on silica (hexane/ethyl acetate, 6:1) to give the title compound as a colorless oil (6 mg, 65%). The two diastereomers of compound **50** exist in solution as two rotamers each. **^{1}H NMR** (600 MHz, [D$_8$]-toluene): δ 7.85 – 7.62 (m, 4H), 7.31 – 7.15 (m, 9H), 7.14 – 7.04 (m, 2H), 6.98 (d, *J* = 2.3 Hz, 4H), 6.91 – 6.74 (m, 1H), 6.65 – 6.49 (m, 1H), 5.07 – 4.92 (m, 2H), 4.92 – 4.82 (m, 2H), 4.50 – 4.35 (m, 1H), 4.06 – 3.75 (m, 3H), 3.43 – 3.32 (m, 3H), 3.18 – 3.12 (m, 3H), 2.81 – 2.61 (m, 3H), 2.60 – 2.30 (m, 2H), 2.14 – 1.93 (m, 5H), 1.87 – 1.78 (m,1H), 1.59 – 1.43 (m, 2H), 1.42 – 1.30 (m, 2H), 1.25 – 1.18 (m,10H), 0.98 – 0.96 (m, 9H), 0.20 – 0.08 (m, 6H). **^{13}C NMR** (151 MHz, [D$_8$]-toluene): δ 169.7, 155.99, 155.95, 155.87, 155.72, 155.7, 155.6, 154.43, 154.41, 154.37, 137.6, 137.5, 137.31, 137.25, 136.5, 136.44, 136.41, 136.38, 136.33, 136.30, 135.5, 135.3, 135.2, 134.9, 134.7, 134.3, 134.2, 133.7, 133.3, 132.5, 132.4, 132.0, 131.9, 130.2, 130.1, 130.0, 129.91, 129.85, 129.2, 128.55, 128.52, 128.44, 128.41, 128.23, 128.22, 128.01, 128.00, 127.9, 127.6, 115.8, 115.70, 115.66, 115.5, 111.4, 111.2, 111.0, 110.9, 95.50, 95.46, 85.4, 85.2, 85.0, 82.7, 82.5, 82.4, 81.8, 71.8, 69.7, 69.5, 66.4, 63.5, 63.1, 62.6, 60.0, 55.29, 55.26, 55.24, 55.19, 55.13, 55.08, 55.0, 49.2, 48.7, 48.4, 43.1, 43.0, 42.6, 42.1, 41.2, 40.8, 40.5, 39.0, 38.6, 33.4, 33.2, 31.5, 30.9, 30.3, 30.1, 27.4, 27.3, 26.11, 26.07, 25.7, 25.3, 24.0, 19.7, 19.6, 18.8, 18.5, 18.4, 18.3, 17.6, 17.5, 14.2, 1.4, –4.1, –4.2, –4.6, –4.7. **HRMS** (ESI) calc. for $C_{58}H_{75}O_7NSi_2Na$ [M + Na]$^+$: 976.49743; found: 976.49749. The spectroscopic data was in agreement with the literature.[9]

Compound 52

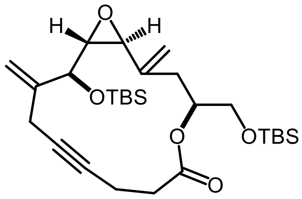

A 50 mL Schlenk flask was charged with diyne **51** (10 mg, 17 μmol), powdered molecular sieves (250 mg, 5Å) and toluene (9 mL). Adduct **16a** (2 mg, 2 μmol, 10 mol%) was weight out in air and dissolved in toluene (1 mL). The catalyst solution was added dropwise to the solution of the substrate at 60 °C and stirring was continued for 1.5 h at this temperature. The reaction was quenched with ethanol (5 mL) before the mixture was cooled to ambient temperature and filtered through a short pad of Celite® which was carefully rinsed with ethyl acetate (20 mL) and dichloromethane (10 mL). The solvents were evaporated and the residue was purified by flash chromatography on silica (hexane/ethyl acetate, 22:1) to give the title compound as a colorless solid (9 mg, 94%). **^{1}H NMR** (400 MHz, $CDCl_3$): δ 5.31 (s, 1H), 5.18 – 5.15 (m, 1H), 5.14 (s, 1H), 5.08 (d, *J* = 1.1 Hz, 1H), 5.07 – 4.99 (m, 1H), 4.25 (d, *J* = 2.9 Hz, 1H), 3.74 – 3.58 (m, 2H), 3.48 (d, *J* = 2.0 Hz, 1H), 3.26 (dd, *J* = 3.0, 2.1 Hz, 1H), 3.11 – 3.02 (m, 1H), 2.83 – 2.72 (m, 1H), 2.52 – 2.30 (m, 5H), 2.18 (dd, *J* = 15.5, 9.9 Hz, 1H), 0.88 (s, 9H), 0.87 (s, 9H), 0.05 (s, 3H), 0.04 (s, 3H), 0.04 (s, 3H), 0.01 (s, 3H). **^{13}C NMR** (101 MHz, $CDCl_3$): δ 171.6, 144.8, 141.2, 116.4, 115.6, 79.5, 79.2, 74.4, 73.1, 64.3, 59.9, 56.9, 34.6, 31.7, 26.0, 25.9, 25.8, 21.4, 18.4, 18.3, 15.5, –4.8, –5.0, –5.2, –5.3. **HRMS** (ESI) calc. for $C_{28}H_{48}O_5Si_2Na$ [M + Na]$^+$: 543.29325; found: 543.29340. The spectroscopic data was in agreement with the literature.[8]

Compound 54

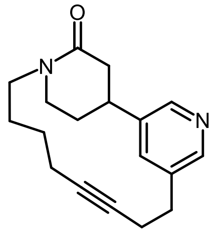

A 50 mL Schlenk flask was charged with diyne **53** (20 mg, 59 μmol), powdered molecular sieves (500 mg, 5Å) and toluene (30 mL). Adduct **16a** (21 mg, 18 μmol, 30 mol%) was weight out in air and dissolved in toluene (1 mL) in a separate Schlenk flask. The catalyst solution was added dropwise to the solution of the substrate stirred at reflux temperature. After 1.5 h, the reaction was quenched by the addition of ethanol (5 mL), the mixture was cooled to ambient temperature and filtered through a short pad of Celite® which was carefully rinsed with ethyl acetate (20 mL) and dichloromethane (10 mL). The solvents were evaporated and the residue was purified by flash chromatography on silica gel (dichloromethane/methanol, 95:5) to give the title compound as a colorless solid (12 mg, 71%). **^{1}H NMR** (400 MHz, CD_2Cl_2): δ 8.40 (bs, 1H), 8.30 (bs, 1H), 7.40 (t, *J* = 2.3 Hz, 1H), 4.38 (ddd, *J* = 13.7, 7.2, 4.0 Hz, 1H), 3.49 (t, *J* = 4.2 Hz, 1H), 3.21 (td, *J* = 12.1, 4.4 Hz, 1H), 3.09 (ddd, *J* = 12.5, 5.6, 2.7 Hz, 1H), 2.91 (ddd, *J* = 13.7, 5.6, 3.2 Hz, 1H), 2.75 – 2.70 (m, 2H), 2.69 – 2.63 (m, 1H), 2.63 – 2.57 (m, 1H), 2.42 – 2.33 (m, 2H), 2.33 – 2.22 (m, 1H), 2.11 – 2.02 (m, 2H), 1.68 – 1.55 (m, 2H), 1.52 – 1.42 (m, 1H), 1.41 – 1.33 (m, 2H). **^{13}C NMR** (101 MHz, CD_2Cl_2): δ 168.6, 148.4, 148.3, 138.0, 136.7, 134.6, 82.0, 79.5, 47.3, 45.1, 35.7, 34.0, 32.8, 29.9, 28.4, 28.0, 20.9, 18.5. **HRMS** (EI) calc. for $C_{18}H_{22}N_2O$ $[M]^+$: 282.17266; found: 282.17293. The spectroscopic data was in agreement with the literature.[6]

Compound 56

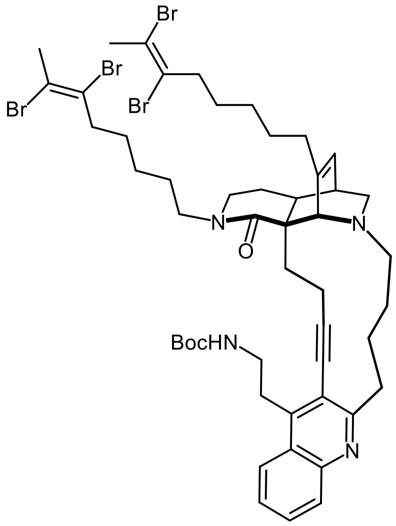

A 25 mL Schlenk flask was charged with diyne **55** (10 mg, 8.7 μmol), powdered molecular sieves (400 mg, 5Å) and toluene (5 mL). Adduct **16a** (3 mg, 2.6 μmol, 30 mol%) was weight out in air and dissolved in toluene (1 mL). The catalyst solution was added dropwise to the solution of the substrate stirred at reflux temperature. After stirring for 2 h, the reaction was quenched with ethanol (5 mL) before the mixture was cooled to ambient temperature and filtered through a short pad of Celite® which was carefully rinsed with ethyl acetate (20 mL) and dichloromethane (10 mL). The solvents were evaporated and the residue was purified by flash chromatography on silica (hexane/ethyl acetate, 4:1 with trimethylamine 5%) to give the title compound as a pale brown oil (8 mg, 81%). **^{1}H NMR** (400 MHz, $CDCl_3$): δ 8.06 (d, *J* = 8.4 Hz, 1H), 7.99 (dd, *J* = 8.4, 1.3 Hz, 1H), 7.63 (ddd, *J* = 8.3, 6.8, 1.3 Hz, 1H), 7.54 – 7.45 (m, 1H), 5.90 – 5.79 (m, 1H), 4.81 (s, 1H), 3.78 – 3.70 (m, 1H), 3.66 – 3.36 (m, 7H), 3.34 – 3.25 (m, 1H), 3.12 – 2.98 (m, 3H), 2.93 (dd, *J* = 9.3, 2.1 Hz, 1H), 2.86 – 2.77 (m, 1H), 2.75 – 2.58 (m, 6H), 2.46 (d, *J* = 34.6 Hz, 1H), 2.44 (d, *J* = 1.0 Hz, 3H), 2.41 (t, *J* = 0.9 Hz, 3H), 2.34 – 2.19 (m, 4H), 2.17 – 2.06 (m, 1H), 2.03 – 1.91 (m, 1H), 1.90 – 1.81 (m, 1H), 1.73 (dd, *J* = 9.3, 2.5 Hz, 3H), 1.66 – 1.58 (m, 7H), 1.57 – 1.47 (m, 2H), 1.43 (s, 9H), 1.34 (d, *J* = 7.2 Hz, 2H), 1.30 – 1.20 (m, 3H). **^{13}C NMR** (101 MHz, $CDCl_3$): δ 172.1, 164.3, 156.0, 146.2, 145.7, 129.3, 126.4, 123.9, 122.4, 122.1, 115.7, 115.3, 102.4, 79.1, 76.3, 61.0, 55.5, 54.5, 52.7, 47.8, 47.1, 45.5, 40.8, 40.5, 40.4, 37.6, 37.2, 35.1, 30.6, 28.9, 28.62, 28.57, 28.0, 27.8, 27.6, 27.3, 26.8, 25.6, 25.3, 13.6. **HRMS** (ESI) calc. for $C_{50}H_{67}O_3N_4Br_4$ $[M + H]^+$: 1087.19412; found: 1087.19569. The spectroscopic data was in agreement with the literature.[10]

Compound 58. A flame-dried two-necked round bottom flask equipped with a magnetic stir bar and a

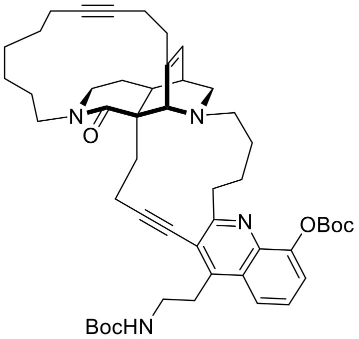

reflux condenser was charged with flame-dried 5 Å molecular sieves (powder, 200 mg) under Ar. Toluene (6 mL) was added and the resulting suspension was degassed by bubbling a gentle stream of Ar through it for 30 min. The suspension was then heated to 110 °C (oil bath) for 30 min before tetra-yne **57** (10 mg, 11 µmol) was added at this temperature as a solution in toluene (1 mL). Next, a solution of catalyst **17b** (1.9 mg, 2.2 µmol) in toluene (0.5 mL) was added dropwise over the course of 10 min. The resulting yellow suspension was stirred for 1 h at 110 °C before it as allowed to come to room temperature. The suspension was filtered through a pad of silica, which was carefully washed with EtOAc (3 x 5 mL). The combined filtrates were concentrated under reduced pressure and the residue was purified by flash chromatography on silica gel (pentane/*tert*-butyl methyl ether, 8:2 to 4:6) to afford the title compound as a white film (7.5 mg, 85%). $[\alpha]_D^{20}$ = 20.1 (c = 0.45, CH_2Cl_2); **^{1}H NMR** (400 MHz, CD_2Cl_2): δ = 7.98 (d, *J* = 8.5 Hz, 1H), 7.47 (t, *J* = 8.0 Hz, 1H), 7.39 (dd, *J* = 7.5, 1.3 Hz, 1H), 6.06 (dd, *J* = 6.8, 2.2 Hz, 1H), 5.14 (s, 1H), 4.17 (ddd, *J* = 13.6, 7.5, 5.8 Hz, 1H), 3.73 (m, 1H), 3.54 – 3.32 (m, 5H), 3.17 (td, *J* = 12.5, 1.9 Hz, 1H), 3.08 (dt, *J* = 12.7, 3.6 Hz, 1H), 3.03 – 2.89 (m, 2H), 2.82 – 2.67 (m, 2H), 2.63 – 2.47 (m, 4H), 2.42 – 2.24 (m, 4H), 2.23 – 2.01 (m, 4H), 1.95 (ddd, *J* = 14.4, 10.0, 5.0 Hz, 1H), 1.91 – 1.81 (m, 1H), 1.72 (dd, *J* = 9.5, 2.6 Hz, 1H), 1.66 – 1.54 (m, 13H), 1.53 – 1.44 (m, 4H), 1.43 (s, 9H), 1.38 – 1.23 (m, 5H) ppm; **^{13}C NMR** (101 MHz, CD_2Cl_2): δ = 172.3, 164.6, 156.3, 152.2, 148.0, 147.1, 143.2, 139.2, 127.5, 125.8, 122.3, 121.8, 120.7, 119.1, 103.5, 83.4, 80.9, 80.1, 79.2, 76.3, 61.0, 55.6, 54.4, 52.6, 47.9, 46.3, 43.6, 41.0, 39.1, 37.4, 37.3, 34.3, 31.5, 30.7, 29.2, 28.6 (3C), 28.0, 28.0, 27.78 (3C), 27.76, 25.5, 25.4, 18.1, 15.1, 13.9 ppm; **IR** (film) $\tilde{\nu}$ = 3338, 2926, 2853, 1760, 1705, 1630, 1490, 1367, 1275, 1247, 1150, 753 cm^{-1}; **HRMS** (ESI): *m/z*: calcd. for $C_{49}H_{65}N_4O_6$ $[M+H]^+$: 805.48986, found: 805.48971.

RCAM of Diynes Carrying Different End Caps

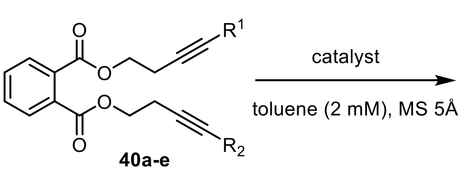

40a-e **41**

R^1 = R^2 = *n*Pr

A 100 mL Schlenk flask was charged with substrate **40b** (35 mg, 0.10 mmol), powdered molecular sieves (5Å, 250 mg) and toluene (50 mL). The mixture was heated to 50 °C before adduct **16a** (6 mg, 5 µmol, 5 mol%) was weight out in air and added in one portion. After stirring for 4 h at 50 °C, the reaction was quenched with ethanol (5 mL) before the mixture was cooled to ambient temperature and filtered through a short pad of Celite® which was carefully rinsed with ethyl acetate (20 mL). The solvents were evaporated and the residue was purified by flash chromatography on silica gel (hexane/ethyl acetate, 4:1) to give the title compound as a colorless solid (22 mg, 90%).

R^1 = H, R^2 = Me

A 100 mL Schlenk flask was charged with but-3-yn-1-yl pent-3-yn-1-yl phthalate (**40d**, 28 mg, 0.10 mmol), powdered molecular sieves (1.00 g, 5Å), powdered molecular sieves (1.00 g, 4Å) and toluene (50 mL). Adduct **16a** (12 mg, 10 μmol, 10 mol%) was weight out in air and added to the solution in one portion. A stock solution of triphenylborane (0.01 M in toluene, 1.0 mL, 10 μmol, 5 mol%) was added and the resulting mixture stirred for 3 h at ambient temperature. The reaction was quenched by the addition of ethanol (5 mL) and the mixture filtered through a short pad of Celite® which was carefully rinsed with ethyl acetate (20 mL) and dichloromethane (10 mL). The solvents were evaporated and the residue was purified by flash chromatography on silica (hexane/ethyl acetate, 4:1) to give the title compound as a colorless solid (22 mg, 92%). **^{1}H NMR** (400 MHz, $CDCl_3$): δ 7.69 (dd, *J* = 5.7, 3.3 Hz, 2H), 7.53 (dd, *J* = 5.7, 3.3 Hz, 2H), 4.45 (td, *J* = 4.7, 1.5 Hz, 4H), 2.58 – 2.49 (m, 4H). **^{13}C NMR** (101 MHz, $CDCl_3$): δ 167.9, 133.3, 131.1, 128.5, 79.1, 62.8, 19.9. **HRMS** (EI) calc. for $C_{14}H_{12}O_4$ $[M]^+$: 244.07301; found: 244.07312. The spectroscopic data was in agreement with the literature.[29]

R^1 = TMS, R^2 = Me

A 100 mL Schlenk flask was charged with substrate **40c** (36 mg, 0.10 mmol) and toluene (50 mL). Adduct **16a** (6 mg, 5 μmol, 5 mol%) was weight out in air and added in one portion to the mixture stirred at 100 °C. After 7 h at 100 °C, the reaction was quenched with ethanol (5 mL) before the mixture was cooled to ambient temperature and filtered through a short pad of Celite® which was carefully rinsed with ethyl acetate (20 mL). The solvents were evaporated and the residue was purified by flash chromatography on silica (hexane/ethyl acetate, 4:1) to give the title compound as a colorless solid (21 mg, 86%). A second fraction was collected as a clear oil that consisted of the bis-silylated diyne **S15** (3 mg, 7%), which analyzed as follows:

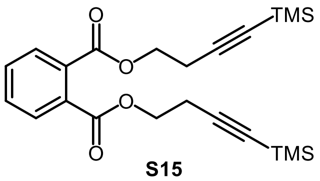

S15

^{1}H NMR (400 MHz, $CDCl_3$): δ 7.78 – 7.73 (m, 2H), 7.58 – 7.52 (m, 2H), 4.40 (t, *J* = 7.2 Hz, 4H), 2.68 (t, *J* = 7.2 Hz, 4H), 0.15 (s, 18H). **^{13}C NMR** (101 MHz, $CDCl_3$): δ 167.2, 132.0, 131.3, 129.2, 102.1, 86.9, 63.4, 20.4, 0.1. **IR** (film): $\tilde{\nu}$ 2961, 2179, 1733, 1273, 1251, 1125, 1081, 1059, 843, 790, 744, 697, 671, 639. **HRMS** (ESI) calc. for $C_{22}H_{30}O_4Si_2Na$ $[M + Na]^+$: 437.15749; found: 437.15786.

Alkyne Homo-Metathesis Reaction at Low Temperature

At 0 °C: A 10 mL Schlenk flask was charged with 1-(4-methoxyphenyl)-1-propyne (**21**, 37 mg, 0.25 mmol), powdered molecular sieves (250 mg, 5Å) and toluene (1.25 mL). The mixture was cooled to 0 °C. Adduct **16a** (15 mg, 13 μmol, 5 mol%) was weight out in air and added in one portion to the mixture, followed by triphenylborane (3 mg, 13 μmol, 5 mol%). After stirring for 24 h at 0 °C, the reaction was quenched with ethanol (1 mL) and the mixture allowed to reach ambient temperature. Work-up and purification were conducted as described above. Product **22a** was obtained as a colorless solid (27 mg, 91%), which was identical in all regard to the sample described above.

At −20 °C: A 10 mL Schlenk flask was charged with 1-(4-methoxyphenyl)-1-propyne (**21**, 37 mg, 0.25 mmol), powdered molecular sieves (250 mg, 5Å) and toluene (1.25 mL). The mixture was cooled to −20 °C. Complex **17** (10 mg, 13 μmol, 5 mol%) was weight out in air and added in one portion to the

mixture. After stirring for 24 h at −20 °C, the reaction was quenched with ethanol (1 mL) and the mixture allowed to warm to ambient temperature. Work-up and purification were conducted as described to give product **22a** as a colorless solid (25 mg, 84%), which was again identical in all regard to the sample described above.

2 References

1. Kol, M.; Shamis, M.; Goldberg, I.; Goldschmidt, Z.; Alfi, S.; Hayut-Salant, E., Titanium(IV) complexes of trianionic amine triphenolate ligands. *Inorg.c Chem. Commun.* **2001,** *4* (4), 177-179.
2. Schaubach, S.; Gebauer, K.; Ungeheuer, F.; Hoffmeister, L.; Ilg, M. K.; Wirtz, C.; Fürstner, A., A Two-Component Alkyne Metathesis Catalyst System with an Improved Substrate Scope and Functional Group Tolerance: Development and Applications to Natural Product Synthesis. *Chem. Eur. J.* **2016,** *22* (25), 8494-8507.
3. Schaubach, S. Catalysts with Multivalent Siloxy Ligands & Formal Total Synthesis of (+)-Aspicilin & Stabilization of α-Helical Peptide Structures. Doctoral Dissertation, TU Dortmund, Mülheim an der Ruhr, 2016.
4. Heppekausen, J. Effiziente Silyloxy-basierte Katalysatoren für die Alkin-Metathese. Doctoral Dissertation, TU Dortmund, Mülheim an der Ruhr, 2012.
5. Fürstner, A.; Seidel, G., Palladium-catalyzed arylation of polar organometallics mediated by 9-methoxy-9-borabicyclo[3.3.1]nonane: Suzuki reactions of extended scope. *Tetrahedron* **1995,** *51* (41), 11165-11176.
6. Dalling, A. G.; Späth, G.; Fürstner, A., Total Synthesis of the Tetracyclic Pyridinium Alkaloid *epi*-Tetradehydrohalicyclamine B. *Angew. Chem. Int. Ed.* **2022,** *61* (41), e202209651.
7. Fürstner, A.; Mathes, C.; Grela, K., Concise total syntheses of epothilone A and C based on alkyne metathesis. *Chem. Commun. (London)* **2001,** (12), 1057-1059.
8. Fürstner, A.; Flügge, S.; Larionov, O.; Takahashi, Y.; Kubota, T.; Kobayashi, J. I., Total Synthesis and Biological Evaluation of Amphidinolide V and Analogues. *Chem. Eur. J* **2009,** *15* (16), 4011-4029.
9. Gebauer, K.; Fürstner, A., Total Synthesis of the Biphenyl Alkaloid (−)-Lythranidine. *Angew. Chem., Int. Ed.* **2014,** *53* (25), 6393-6396.
10. Meng, Z.; Spohr, S. M.; Tobegen, S.; Farès, C.; Fürstner, A., A Unified Approach to Polycyclic Alkaloids of the Ingenamine Estate: Total Syntheses of Keramaphidin B, Ingenamine, and Nominal Njaoamine I. *J. Am. Chem. Soc.* **2021,** *143* (35), 14402-14414.
11. Fulmer, G. R.; Miller, A. J. M.; Sherden, N. H.; Gottlieb, H. E.; Nudelman, A.; Stoltz, B. M.; Bercaw, J. E.; Goldberg, K. I., NMR Chemical Shifts of Trace Impurities: Common Laboratory Solvents, Organics, and Gases in Deuterated Solvents Relevant to the Organometallic Chemist. *Organometallics* **2010,** *29* (9), 2176-2179.
12. Cheatham, S.; Gierth, P.; Bermel, W.; Kupče, Ē., HCNMBC – A pulse sequence for H–(C)–N Multiple Bond Correlations at natural isotopic abundance. *Journal of Magnetic Resonance* **2014,** *247*, 38-41.
13. Hillenbrand, J. Molybdän- und Wolfram-Katalysatoren mit Tripodalen Liganden für die Alkin-Metathese & Isolierung eines homoleptischen Mo(V) Alkoxid Komplexes. Doctoral Dissertation, TU Dortmund, Mülheim an der Ruhr, 2021.
14. Heppekausen, J.; Stade, R.; Kondoh, A.; Seidel, G.; Goddard, R.; Fürstner, A., Optimized Synthesis, Structural Investigations, Ligand Tuning and Synthetic Evaluation of Silyloxy-Based Alkyne Metathesis Catalysts. *Chem. Eur. J* **2012,** *18* (33), 10281-10299.
15. Hillenbrand, J.; Leutzsch, M.; Fürstner, A., Molybdenum Alkylidyne Complexes with Tripodal Silanolate Ligands: The Next Generation of Alkyne Metathesis Catalysts. *Angew. Chem., Int. Ed.* **2019,** *58* (44), 15690-15696.
16. Hentschel, F.; Raimer, B.; Kelter, G.; Fiebig, H.-H.; Sasse, F.; Lindel, T., Synthesis and Cytotoxicity of a Diazirine-Based Photopsammaplin. **2014,** *2014* (10), 2120-2127.

17. Jin, G.; Zhang, X.; Cao, S., Transition-Metal-Free Sonogashira-Type Cross-Coupling of Alkynes with Fluoroarenes. *Org. Lett.* **2013,** *15* (12), 3114-3117.
18. Hillenbrand, J.; Leutzsch, M.; Yiannakas, E.; Gordon, C. P.; Wille, C.; Nöthling, N.; Copéret, C.; Fürstner, A., "Canopy Catalysts" for Alkyne Metathesis: Molybdenum Alkylidyne Complexes with a Tripodal Ligand Framework. *J. Am. Chem. Soc.* **2020,** *142* (25), 11279-11294.
19. Heppekausen, J.; Stade, R.; Goddard, R.; Fürstner, A., Practical New Silyloxy-Based Alkyne Metathesis Catalysts with Optimized Activity and Selectivity Profiles. *J. Am. Chem. Soc.* **2010,** *132* (32), 11045-11057.
20. Takase, M.; Nakajima, A.; Takeuchi, T., Synthesis of an extended hexagonal molecule as a highly symmetrical ligand. *Tetrahedron Lett.* **2005,** *46* (10), 1739-1742.
21. Pschirer, N. G.; Bunz, U. H. F., Alkyne metathesis with simple catalyst systems: High yield dimerization of propynylated aromatics; scope and limitations. *Tetrahedron Lett.* **1999,** *40* (13), 2481-2484.
22. Sattler, L. E.; Hilt, G., Iodonium Cation-Pool Electrolysis for the Three-Component Synthesis of 1,3-Oxazoles. *Chem. Eur. J* **2021,** *27* (2), 605-608.
23. Son, J.-W.; Kim, S.-K.; Kim, S.-N.; Kim, K.-M.; Synthesis of Functionalized Bisarylacetylene Derivatives from Acetylene Gas Over Nano-sized Carbon Ball Supported Palladium Catalyst. *Bull. Korean Chem. Soc.* **2010,** *31* (7), 2097-2099.
24. Draper, S. M.; Gregg, D. J.; Madathil, R., Heterosuperbenzenes: A New Family of Nitrogen-Functionalized, Graphitic Molecules. *J. Am. Chem. Soc.* **2002,** *124* (14), 3486-3487.
25. Qiu, S.; Zhang, C.; Qiu, R.; Yin, G.; Huang, J., One-Pot Domino Synthesis of Diarylalkynes/1,4-Diaryl-1,3-diynes by [9,9-Dimethyl-4,5-bis(diphenylphosphino)-xanthene] (Xantphos)–Copper(I) Iodide–Palladium(II) Acetate-Catalyzed Double Sonogashira-Type Reaction. *Adv. Synth. Catal.* **2018,** *360* (2), 313-321.
26. Obando, D.; Koda, Y.; Pantarat, N.; Lev, S.; Zuo, X.; Bijosono Oei, J.; Widmer, F.; Djordjevic, J. T.; Sorrell, T. C.; Jolliffe, K. A., Synthesis and Evaluation of a Series of Bis(pentylpyridinium) Compounds as Antifungal Agents. *ChemMedChem* **2018,** *13* (14), 1421-1436.
27. Persich, P.; Llaveria, J.; Lhermet, R.; de Haro, T.; Stade, R.; Kondoh, A.; Fürstner, A., Increasing the Structural Span of Alkyne Metathesis. *Chem. Eur. J.* **2013,** *19* (39), 13047-13058.
28. Fürstner, A.; Mathes, C.; Lehmann, C. W., Alkyne Metathesis: Development of a Novel Molybdenum-Based Catalyst System and Its Application to the Total Synthesis of Epothilone A and C. *Chem. Eur. J* **2001,** *7* (24), 5299-5317.
29. Haberlag, B.; Freytag, M.; Daniliuc, C. G.; Jones, P. G.; Tamm, M., Efficient Metathesis of Terminal Alkynes. *Angew. Chem. Int. Ed.* **2012,** *51* (52), 13019-13022.
30. Du, Y.; Yang, H.; Zhu, C.; Ortiz, M.; Okochi, K. D.; Shoemaker, R.; Jin, Y.; Zhang, W., *Chem. - Eur. J.* **2016,** *22*, 7959-7963.

3 NMR Spectra of New Compounds

Ligands

^{1}H NMR of 12a, 400 MHz, $CDCl_3$, 25 °C

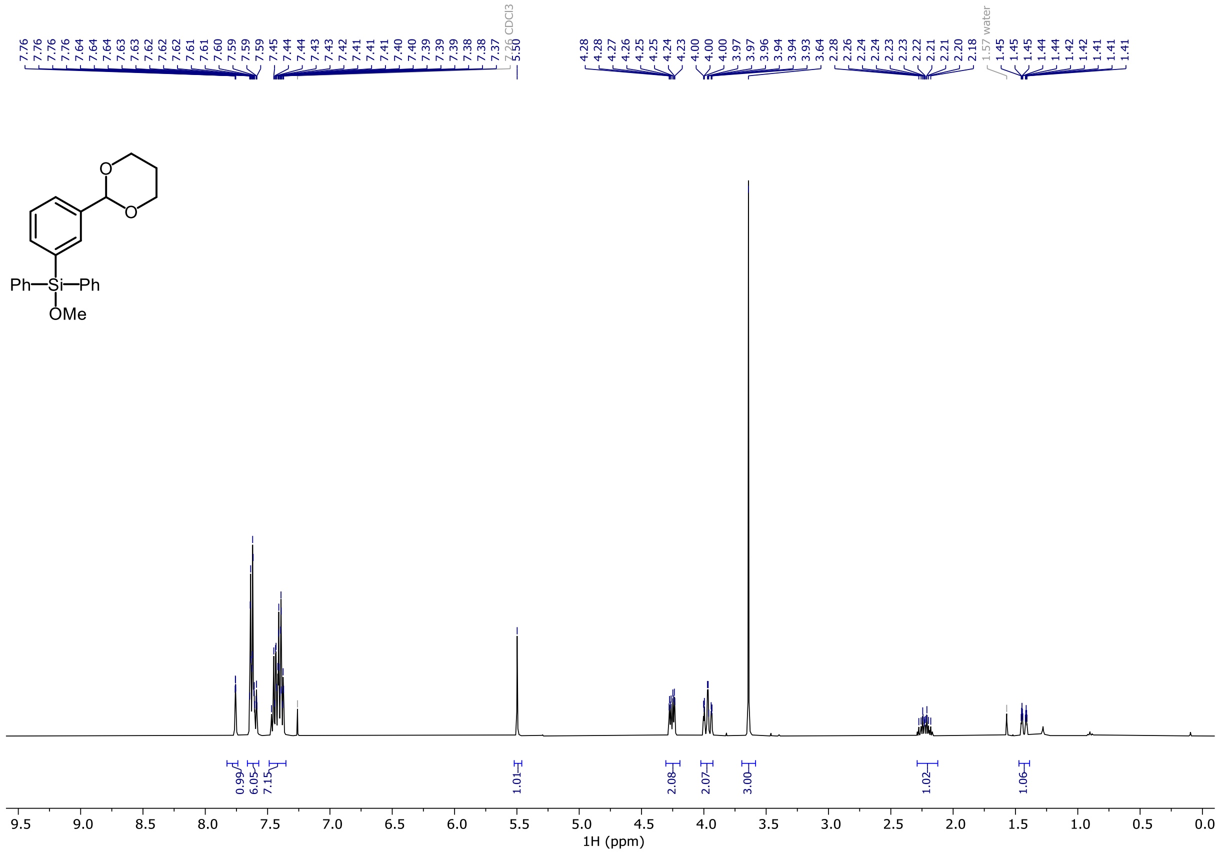

^{13}C NMR of 12a, 101 MHz, $CDCl_3$, 25 °C

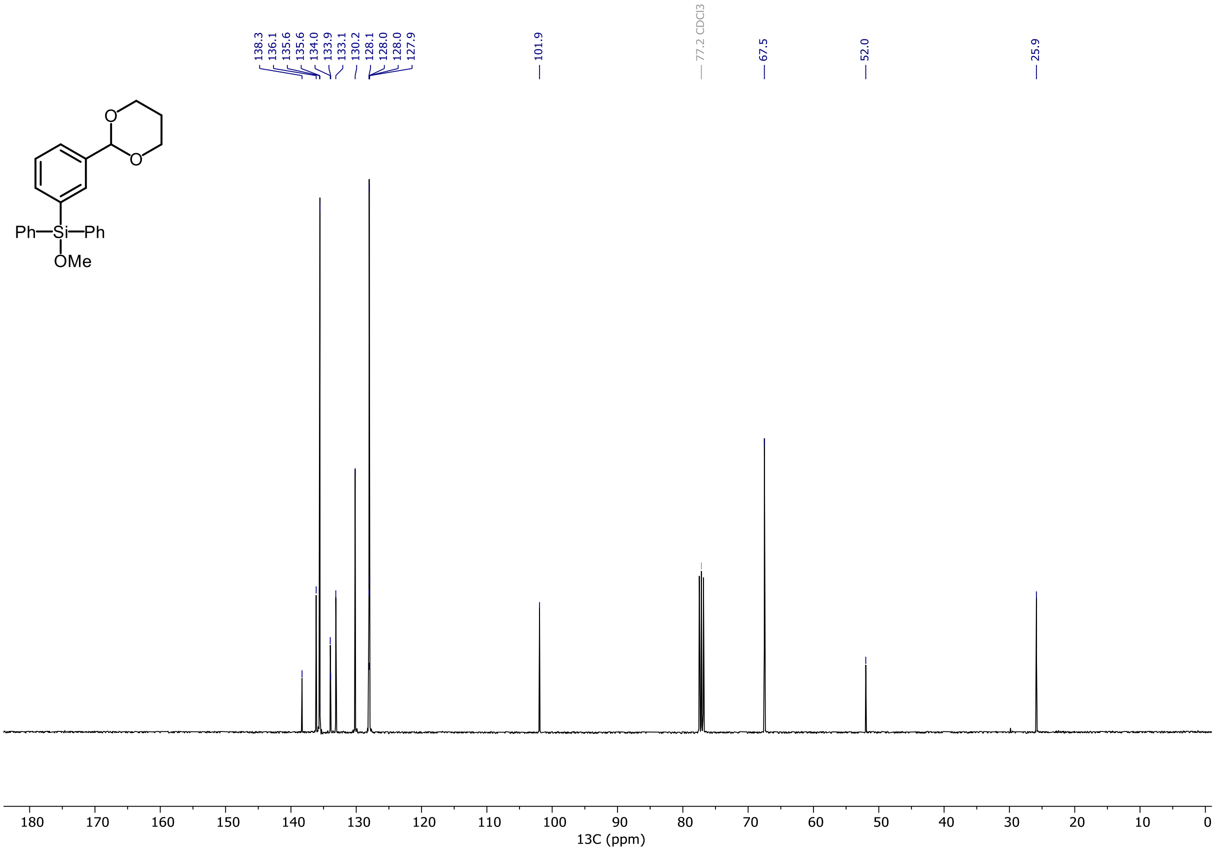

^{29}Si NMR of 12a, 60 MHz, $CDCl_3$, 25 °C

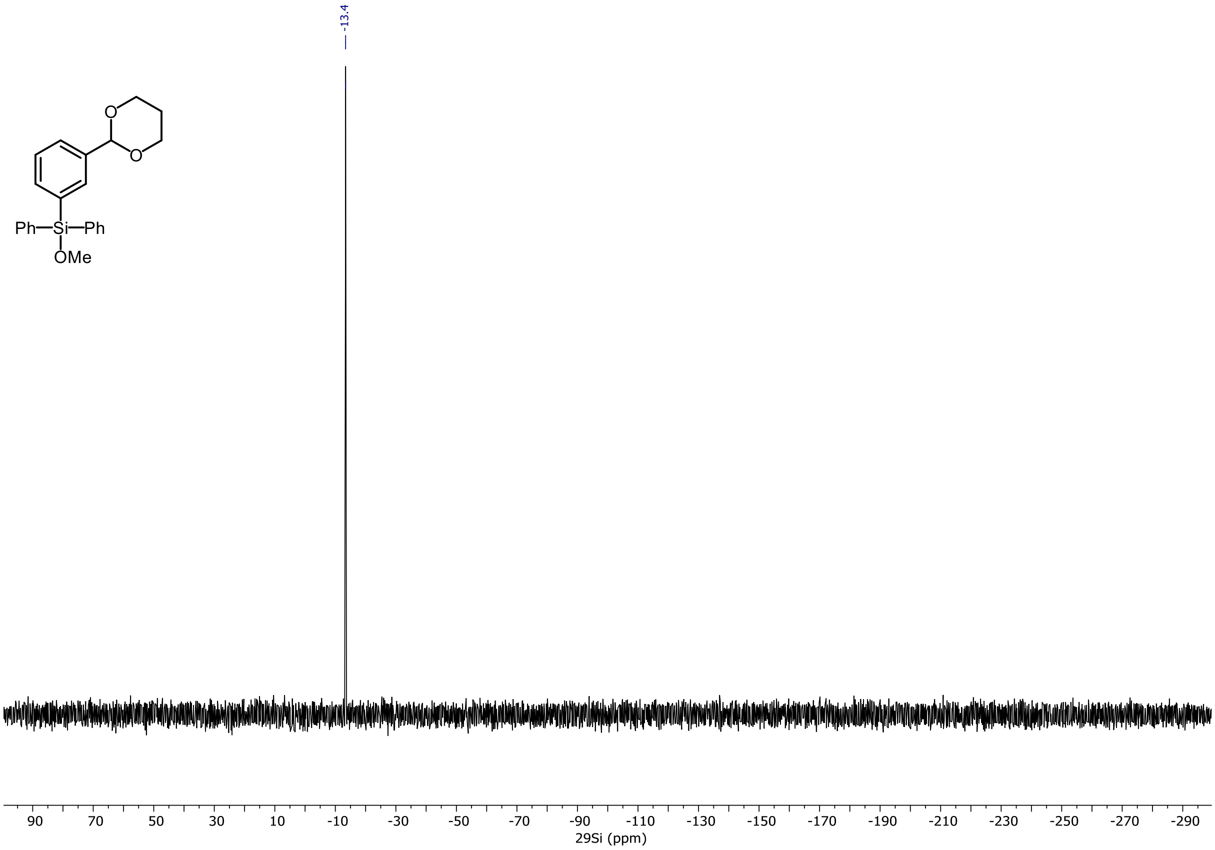

^{1}H NMR of 13a, 400 MHz, $CDCl_3$, 25 °C

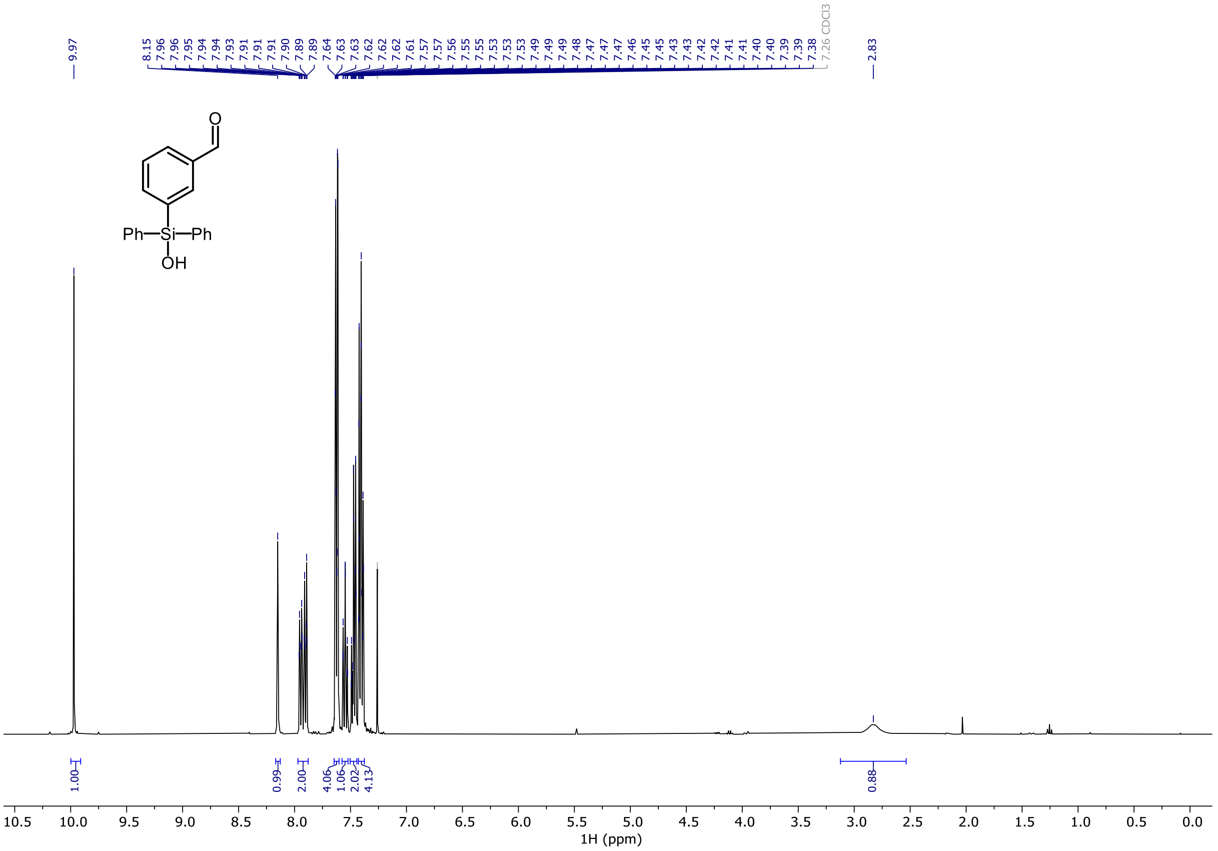

^{13}C NMR of 13a, 101 MHz, $CDCl_3$, 25 °C

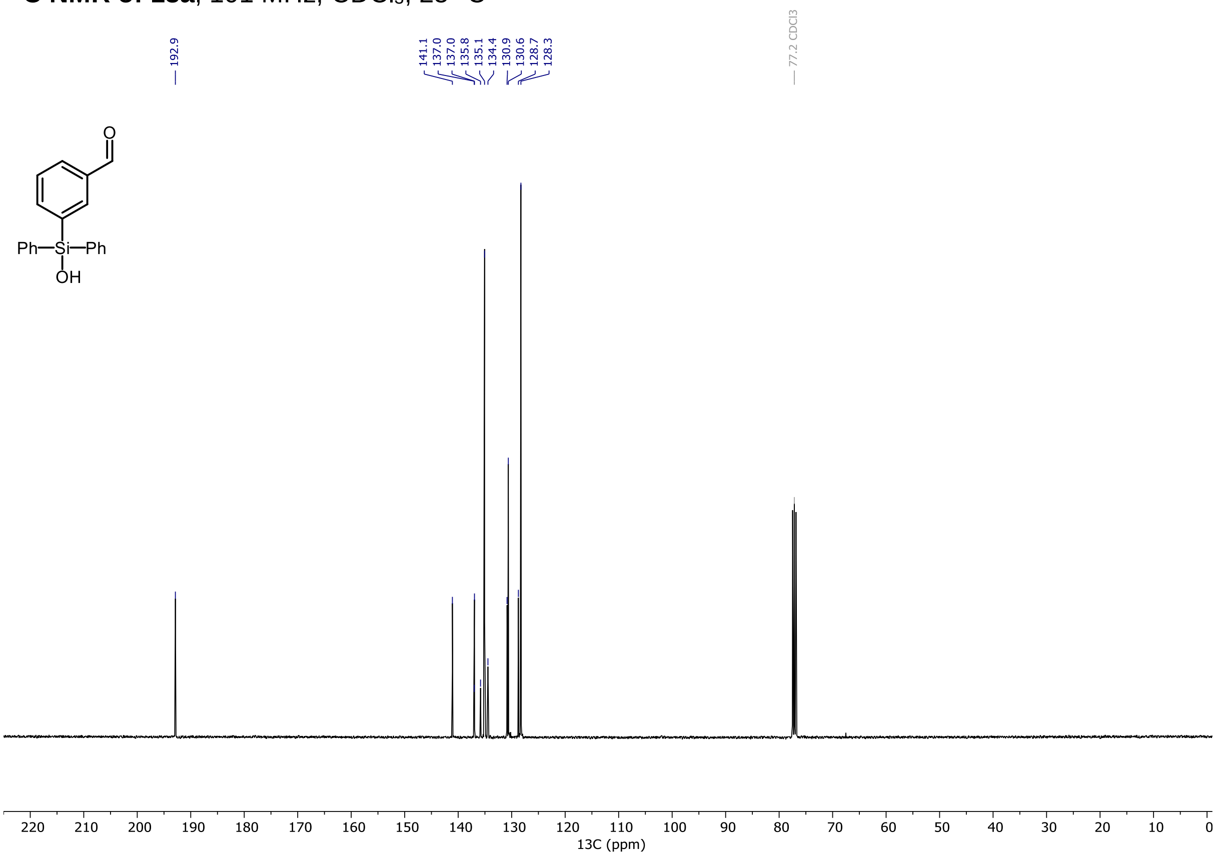

^{29}Si NMR of 13a, 60 MHz, $CDCl_3$, 25 °C

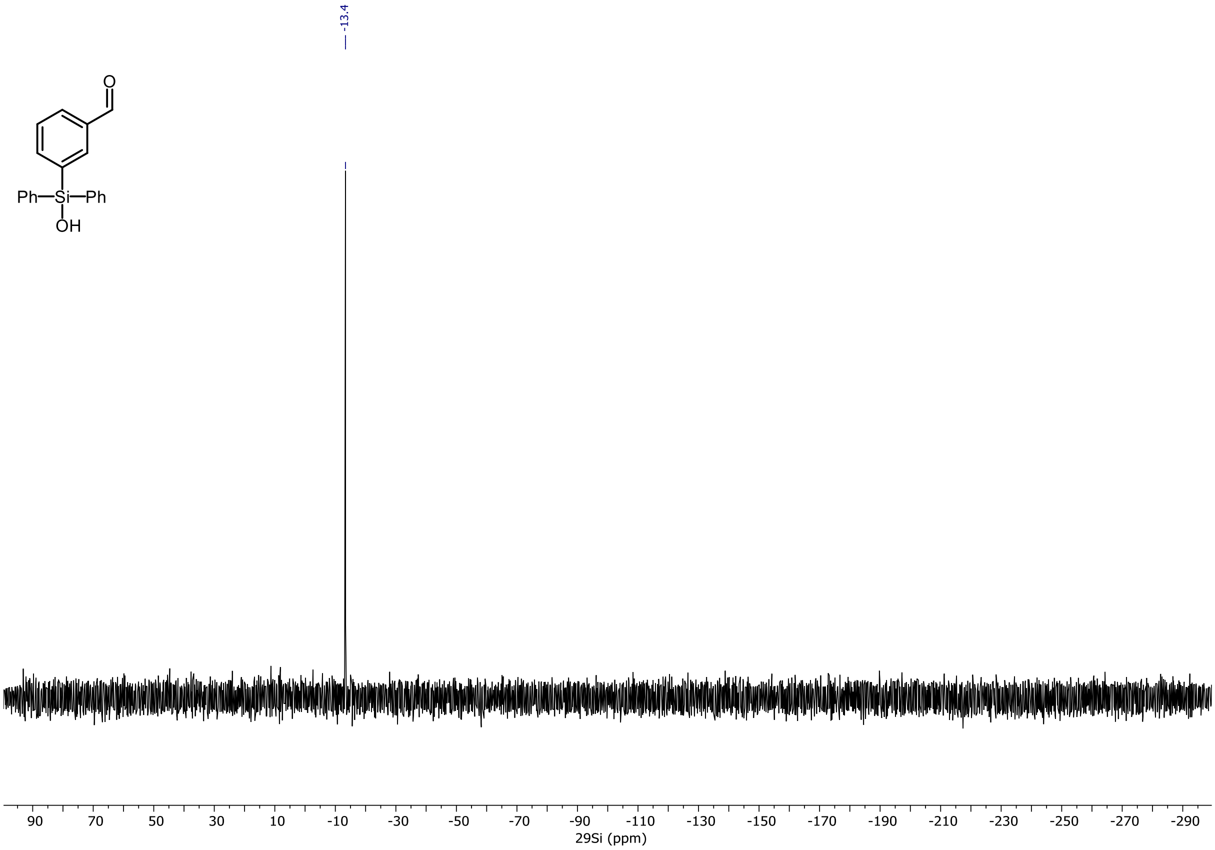

^{1}H NMR of ligand 14a, 600 MHz, $CDCl_3$, 25 °C

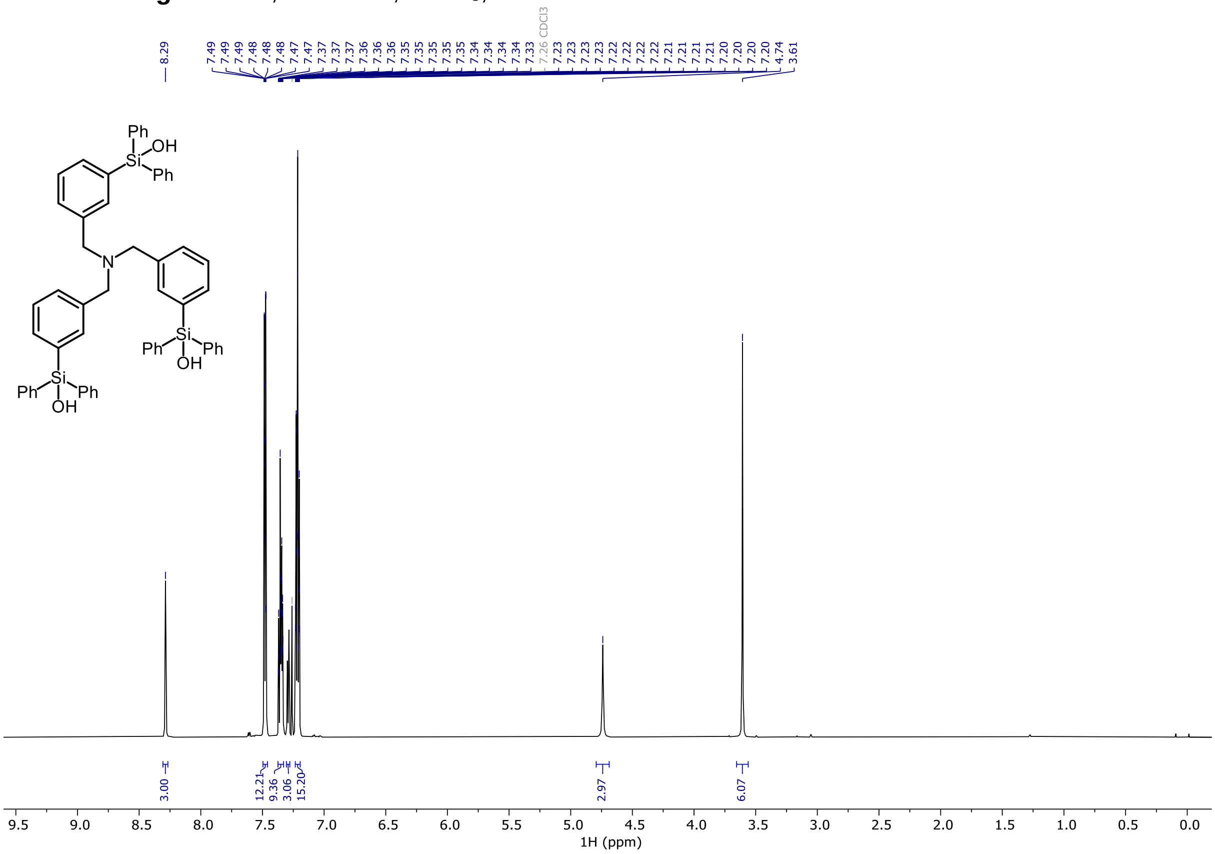

^{13}C NMR of ligand 14a, 151 MHz, $CDCl_3$, 25 °C

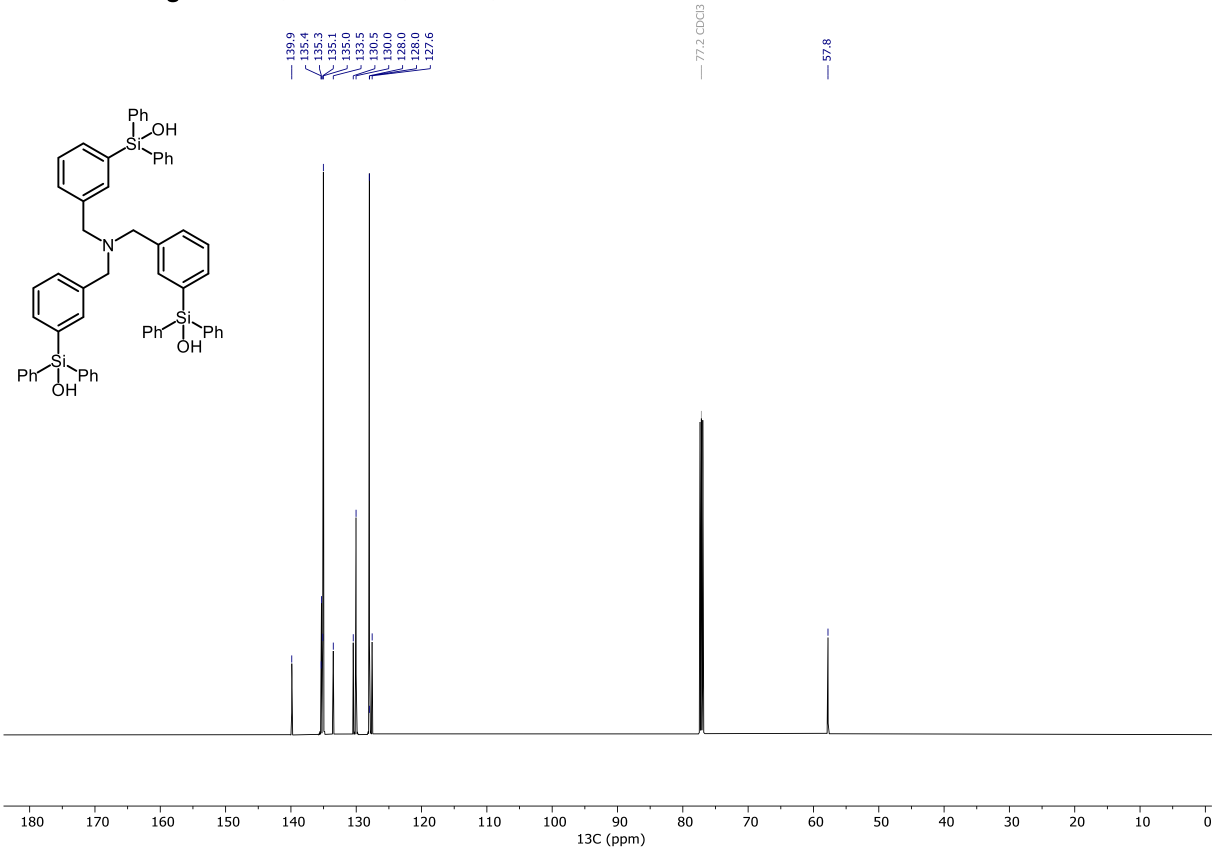

^{29}Si NMR of ligand 14a, 119 MHz, $CDCl_3$, 25 °C

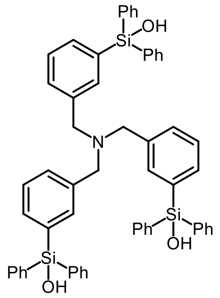

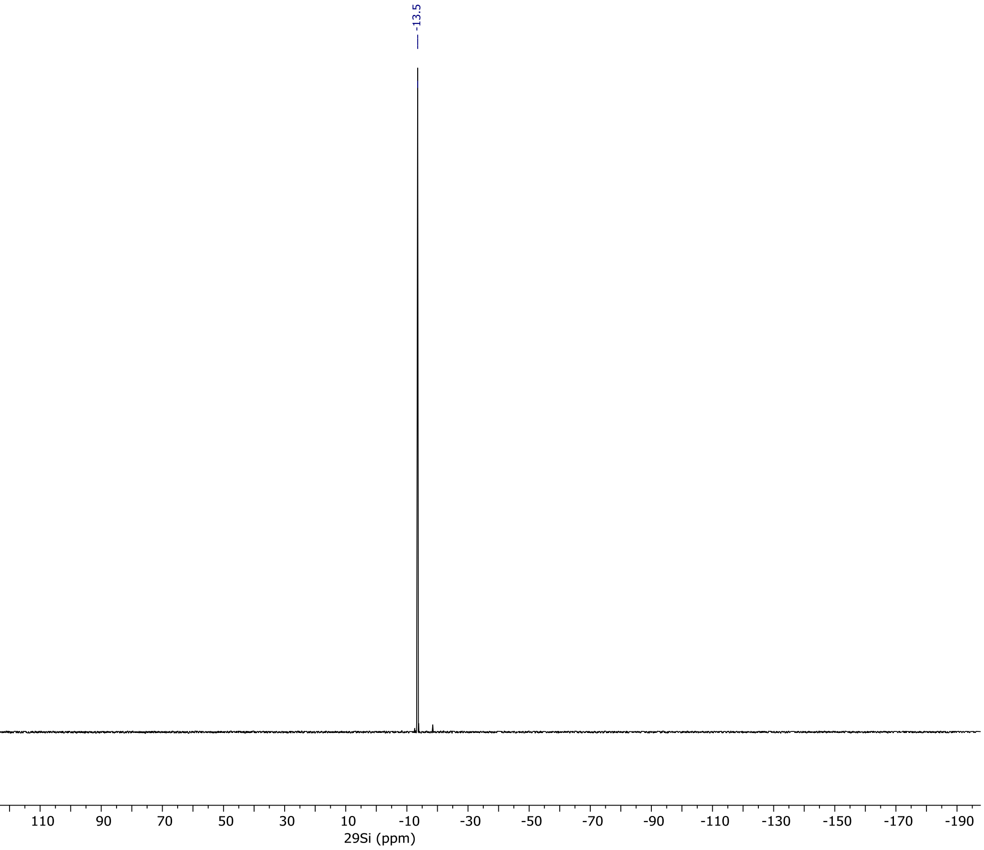

^{1}H NMR of S4, 400 MHz, $CDCl_3$, 25 °C

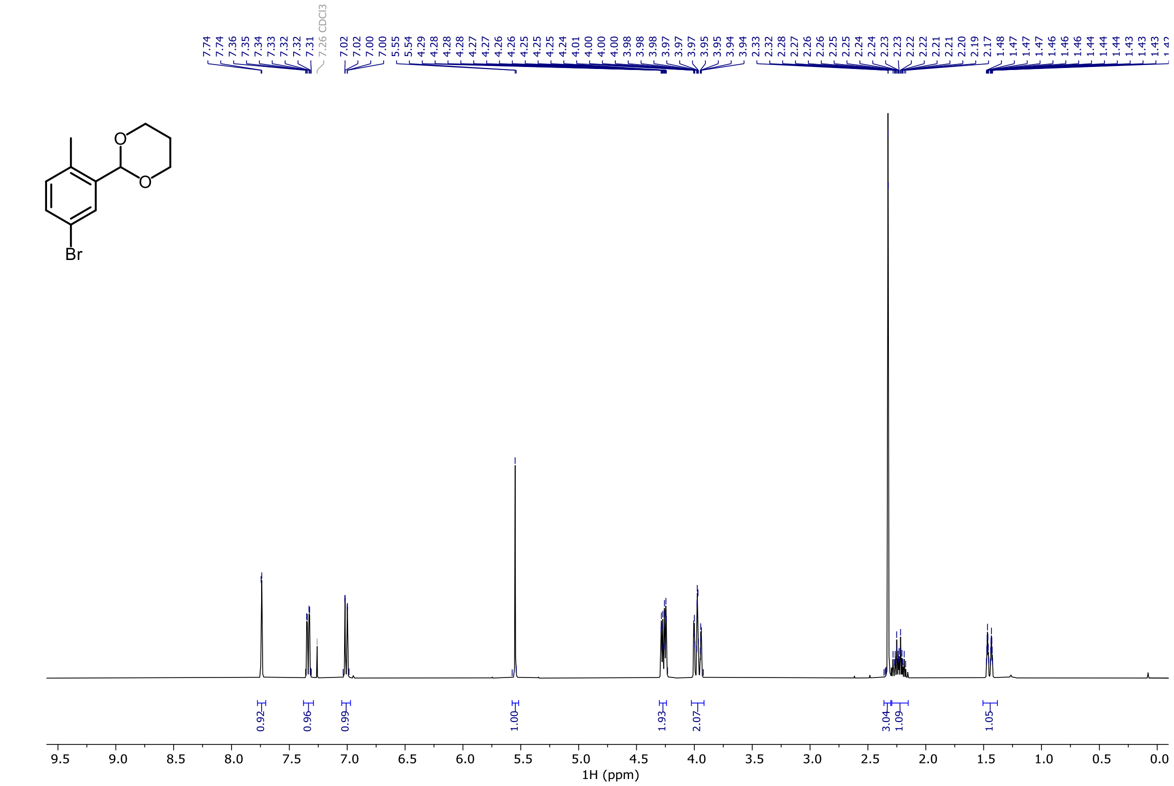

^{13}C NMR of S4, 101 MHz, $CDCl_3$, 25 °C

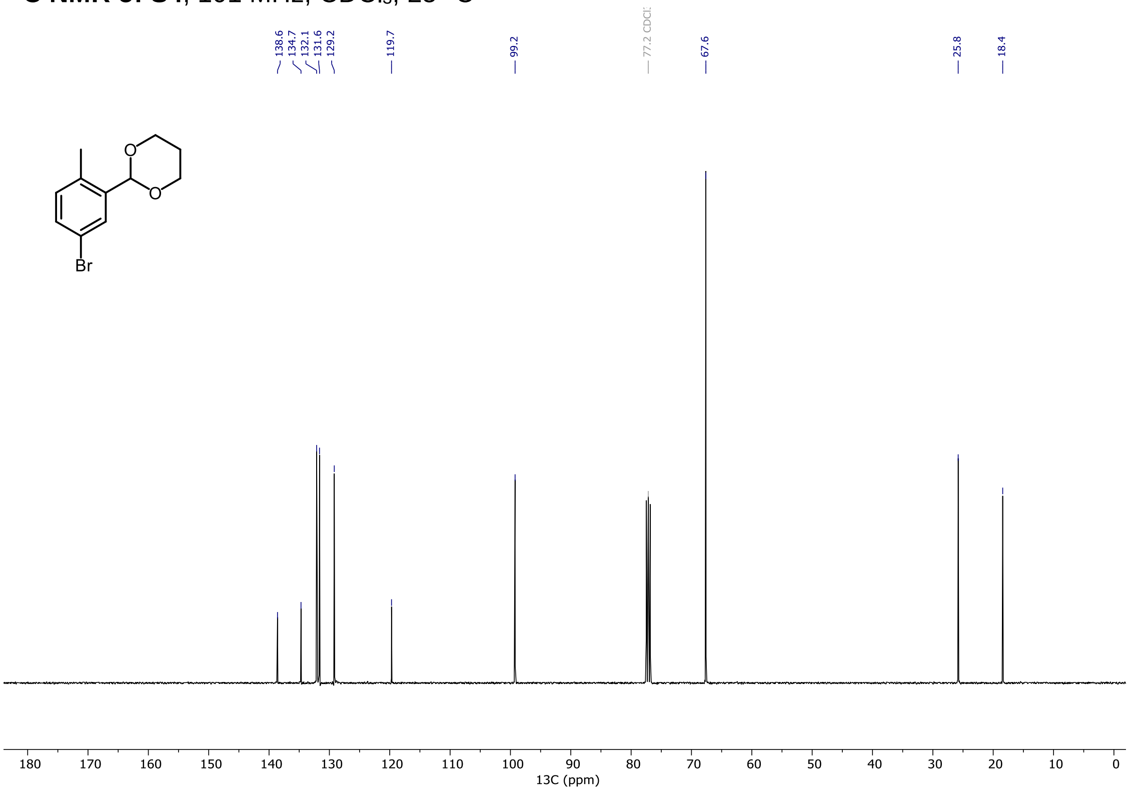

^{1}H NMR of 12b, 400 MHz, $CDCl_3$, 25 °C

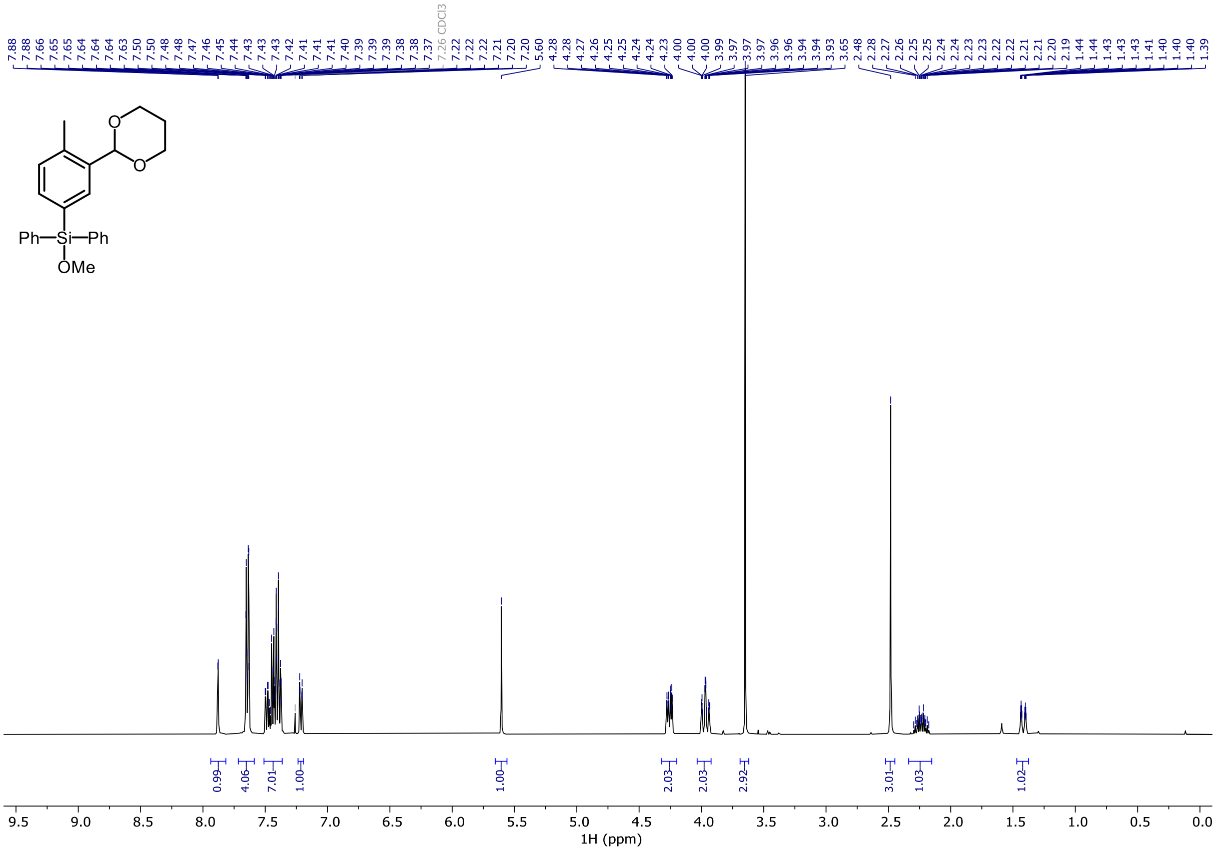

^{13}C NMR of 12b, 101 MHz, $CDCl_3$, 25 °C

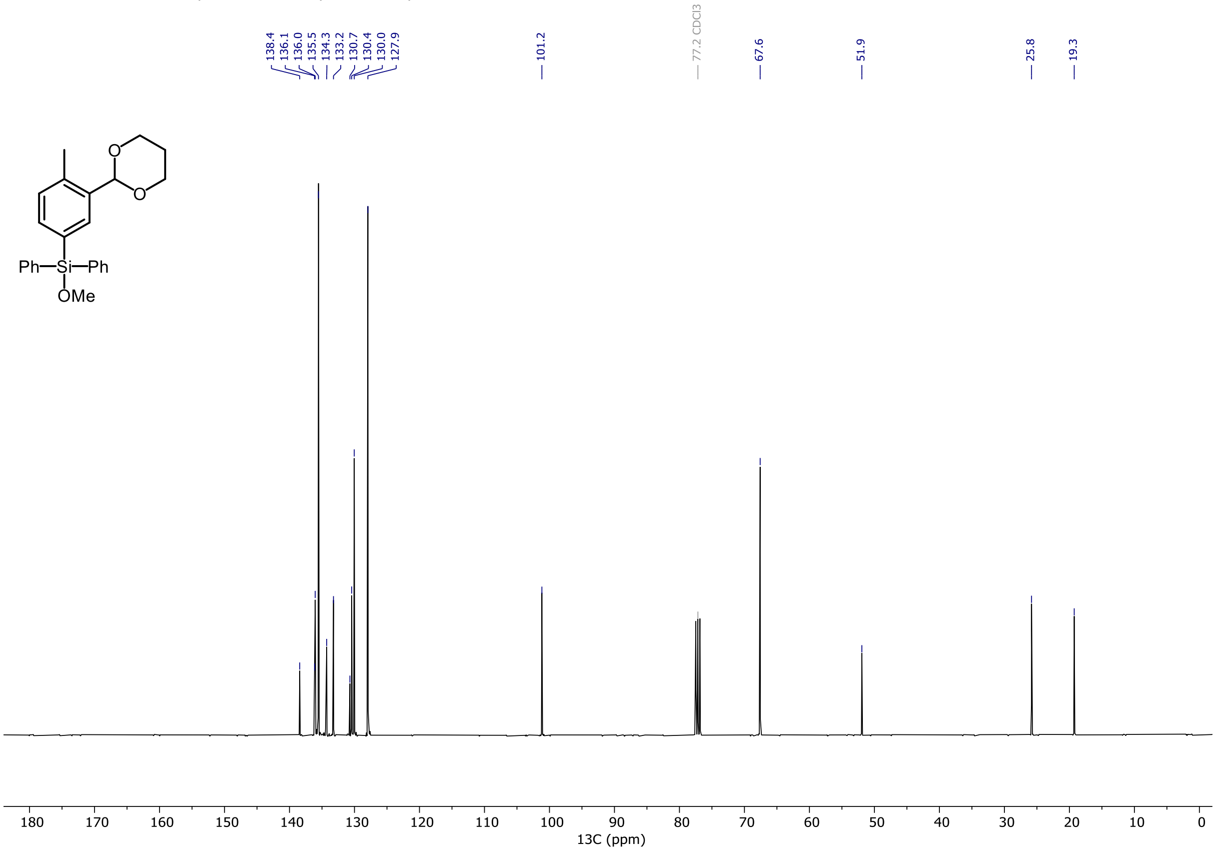

^{29}Si NMR of 12b, 60 MHz, $CDCl_3$, 25 °C

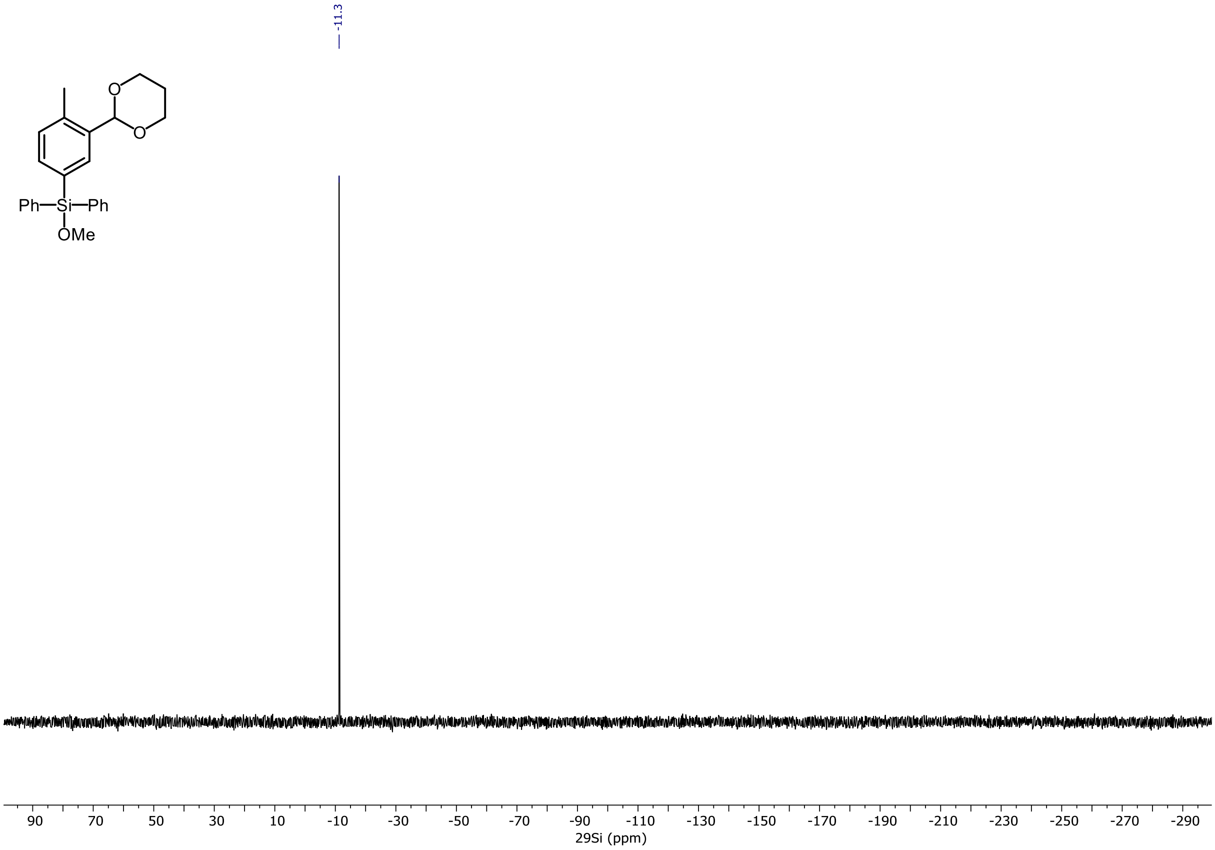

^{1}H NMR of 13b, 400 MHz, $CDCl_3$, 25 °C

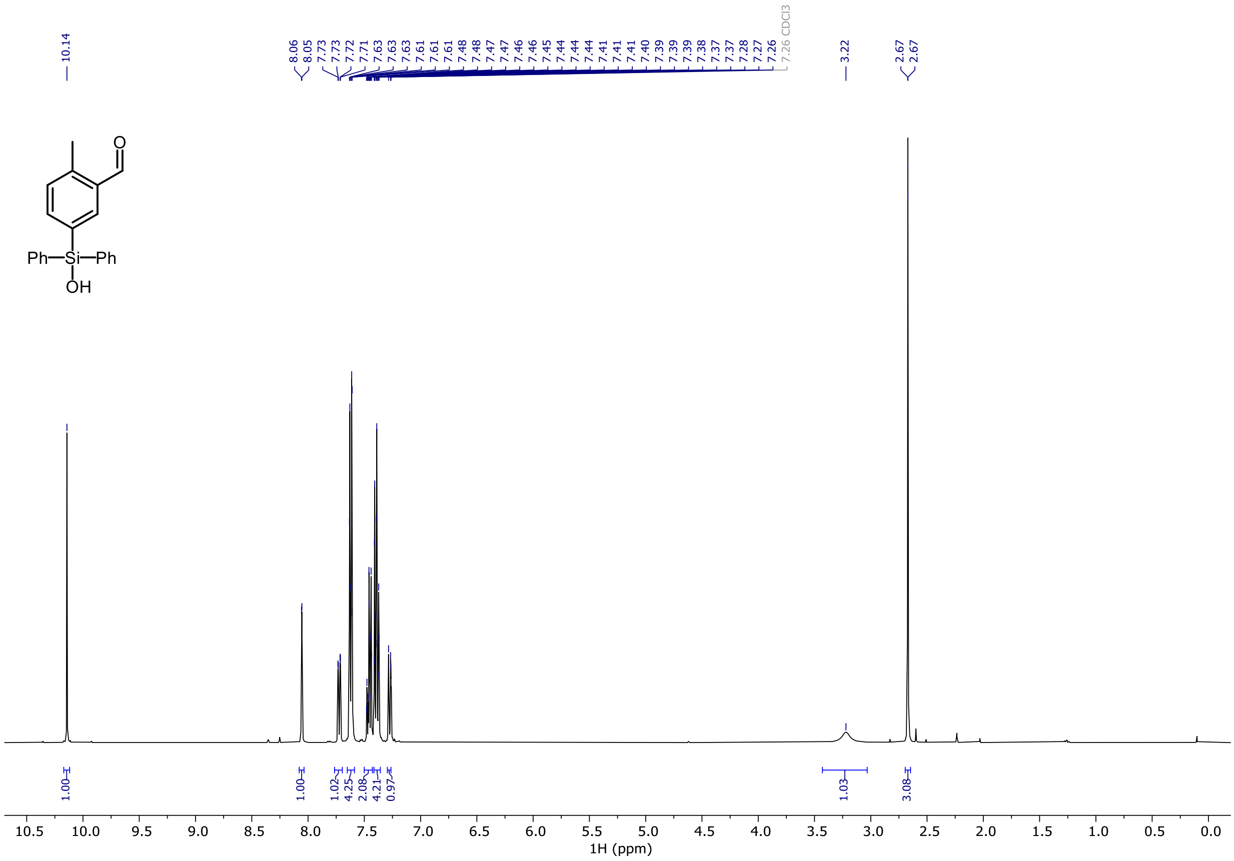

^{13}C NMR of 13b, 101 MHz, $CDCl_3$, 25 °C

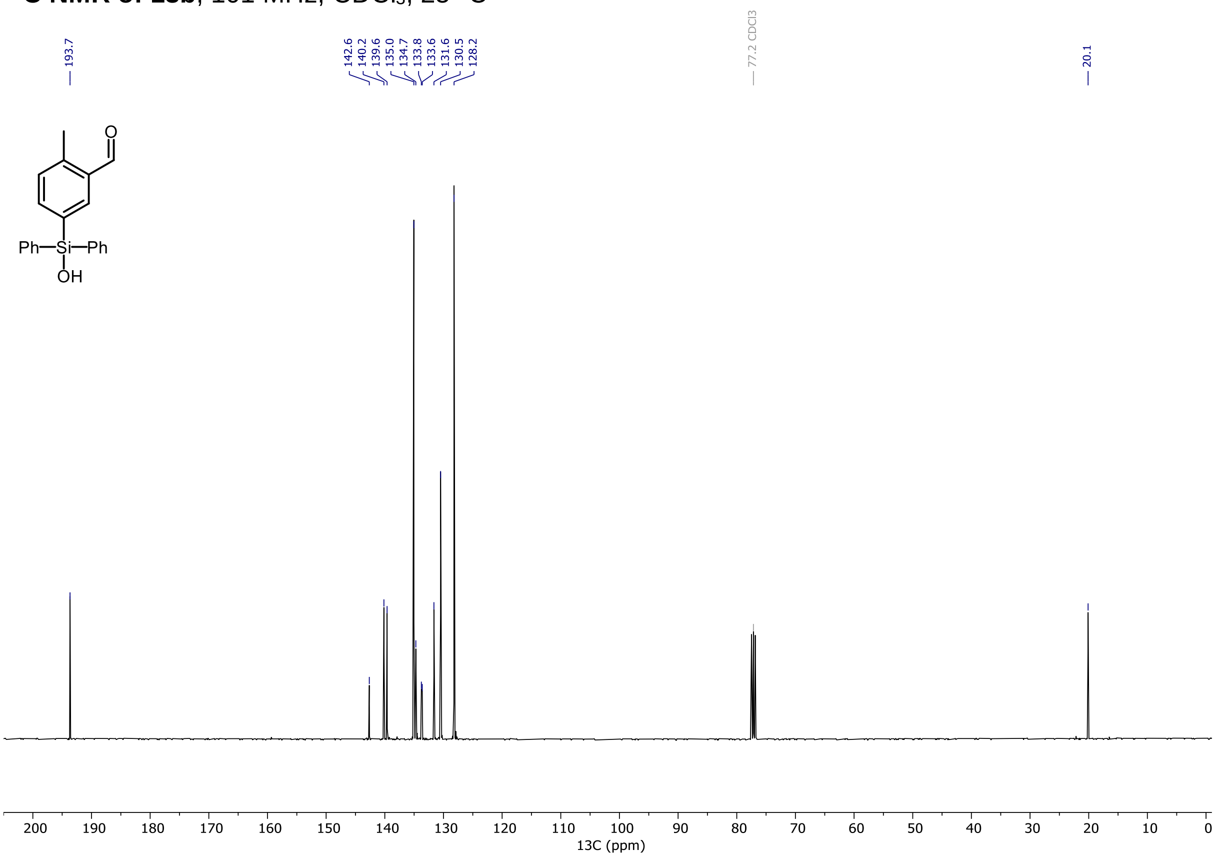

^{29}Si NMR of 13b, 60 MHz, $CDCl_3$, 25 °C

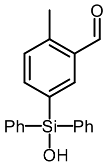

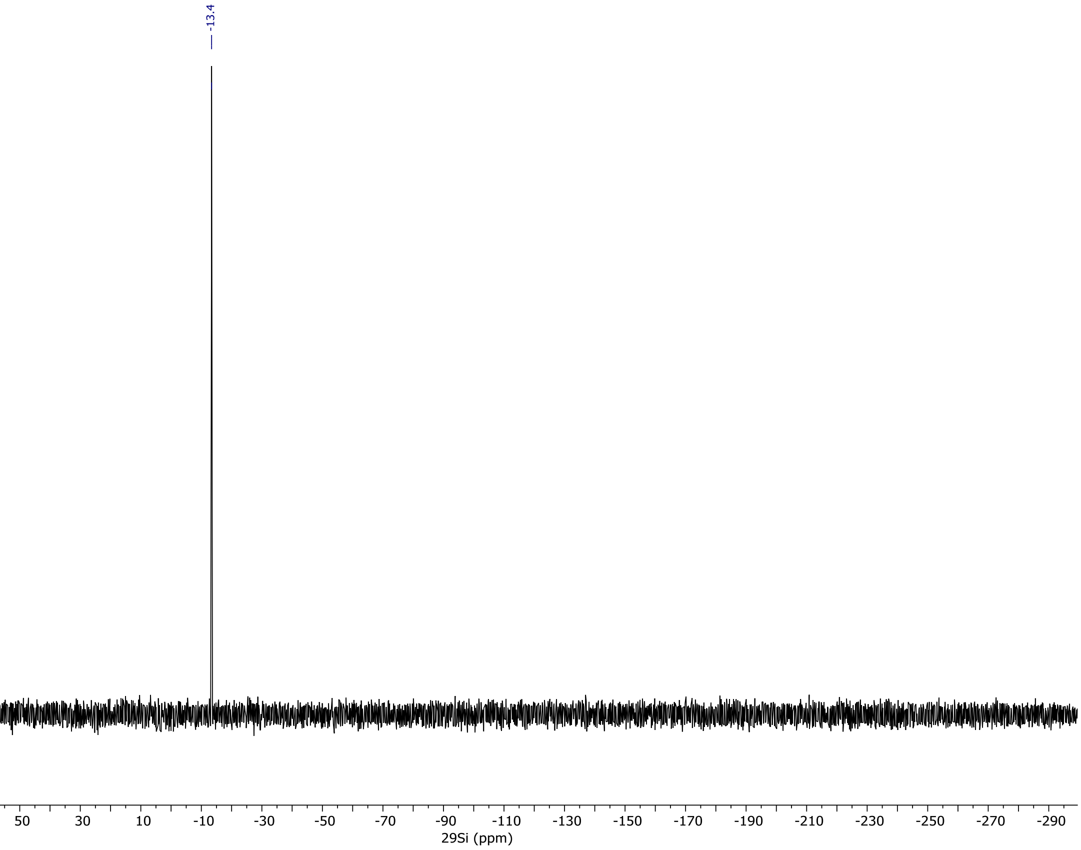

^{1}H NMR of ligand 14b, 600 MHz, $CDCl_3$, 25 °C

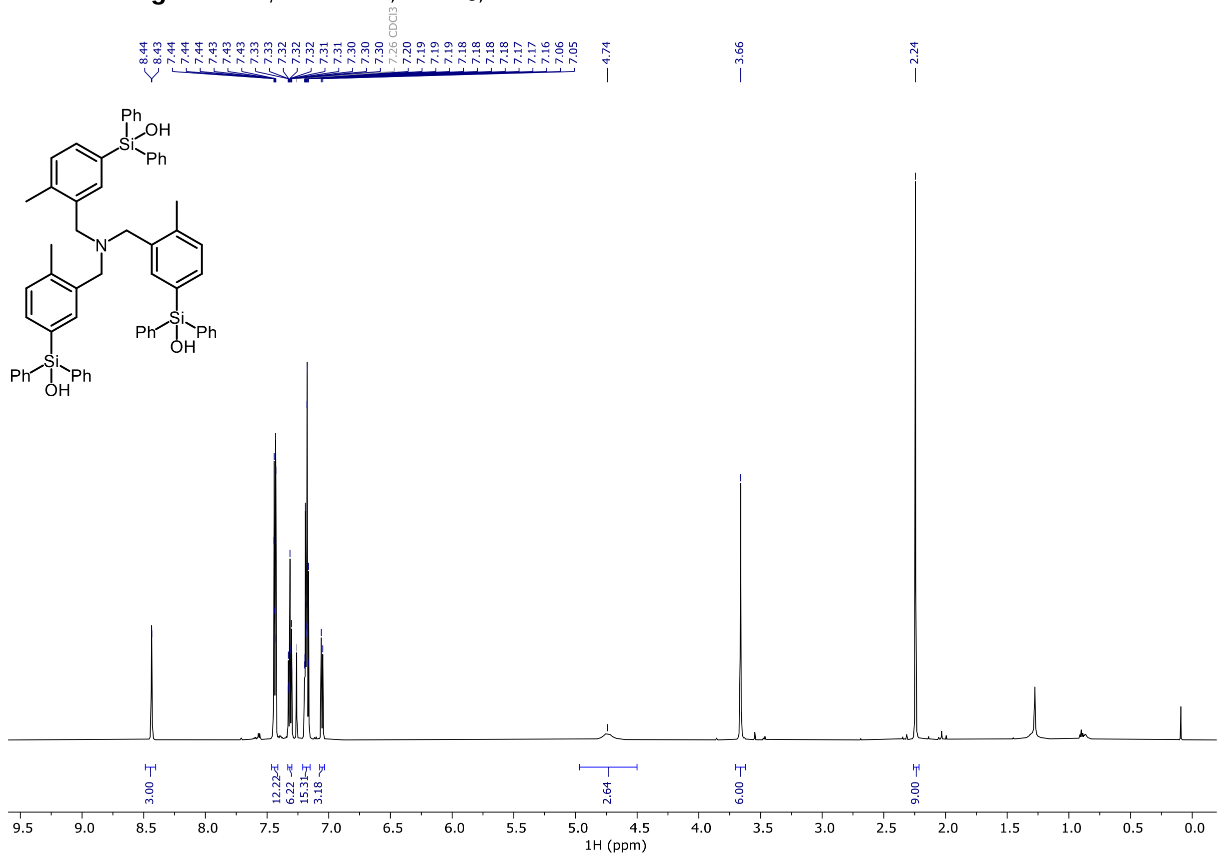

^{13}C NMR of ligand 14b, 151 MHz, $CDCl_3$, 25 °C

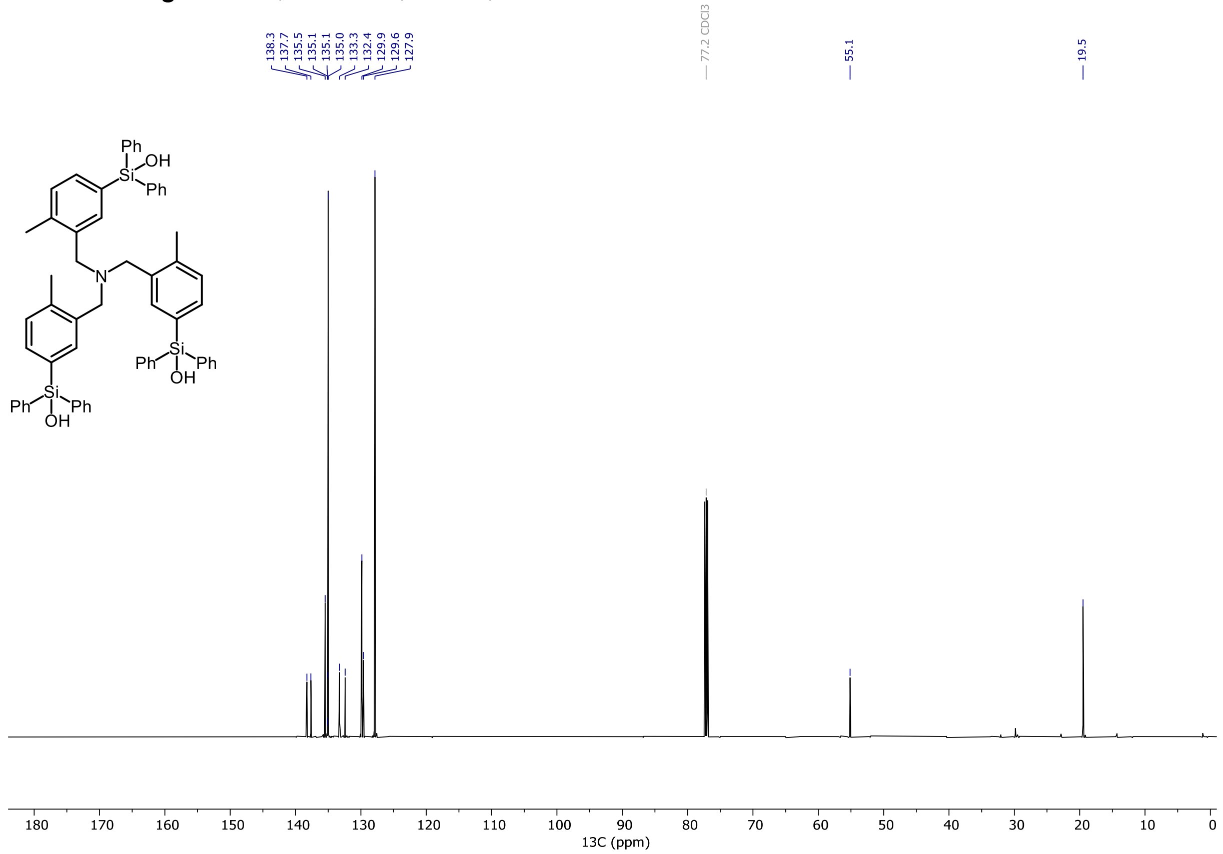

^{29}Si NMR of ligand 14b, 119 MHz, $CDCl_3$, 25 °C

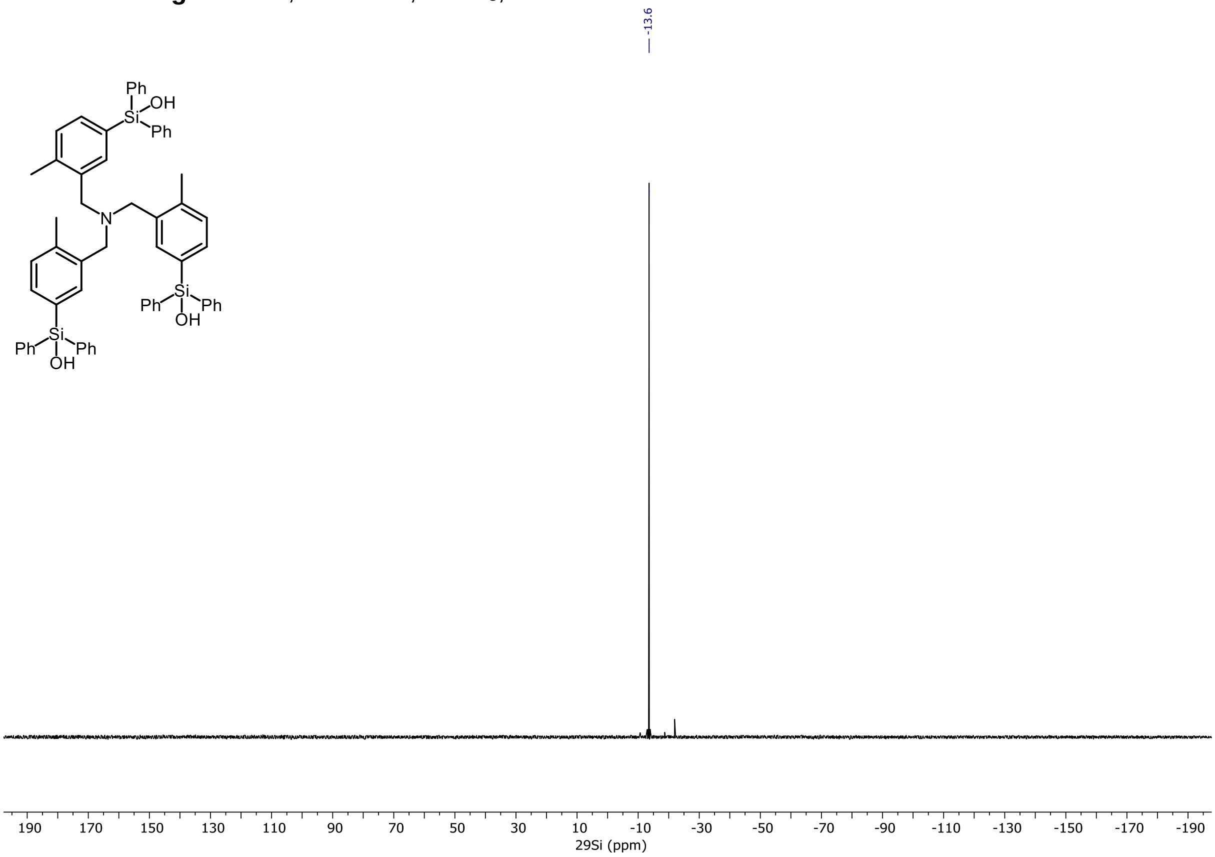

^{1}H -^{15}N HMBC of ligand 14b, 61 MHz, $CDCl_3$, 25 °C

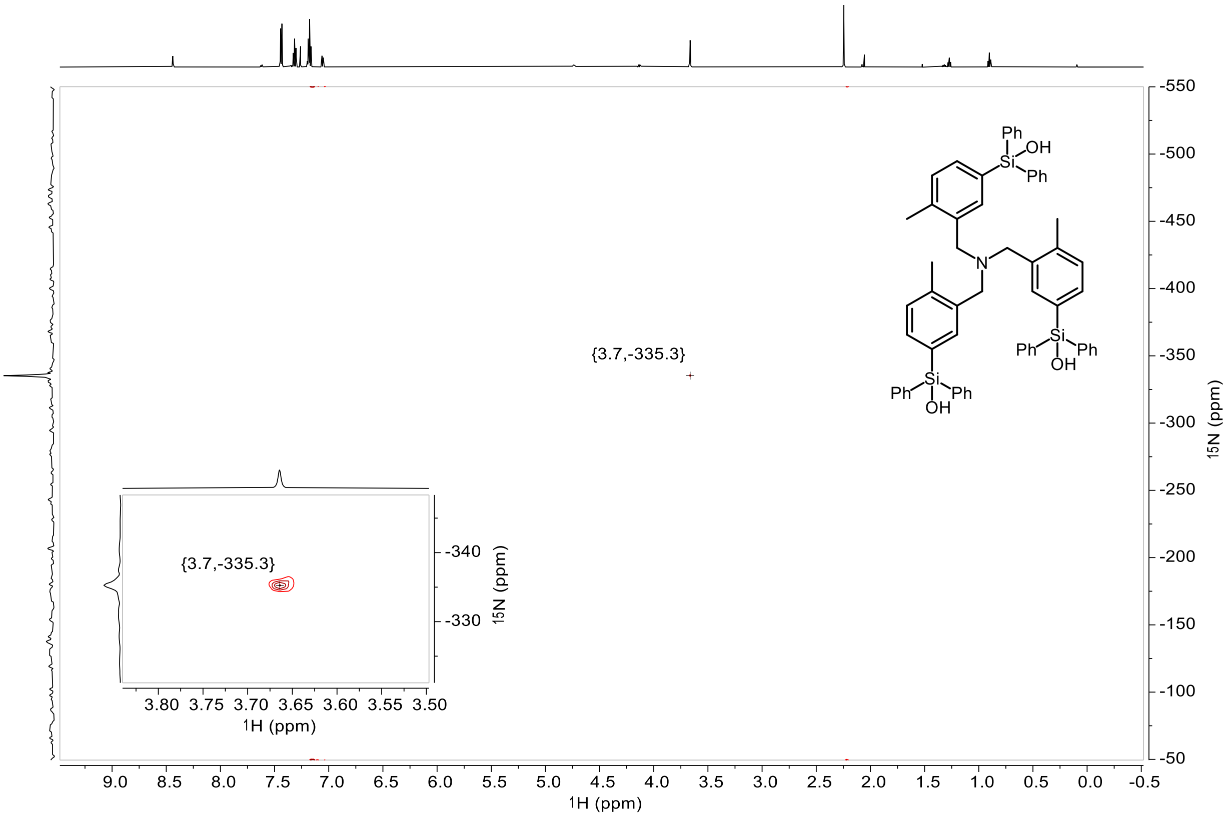

¹H NMR of S5, 600 MHz, $CDCl_3$, 25 °C

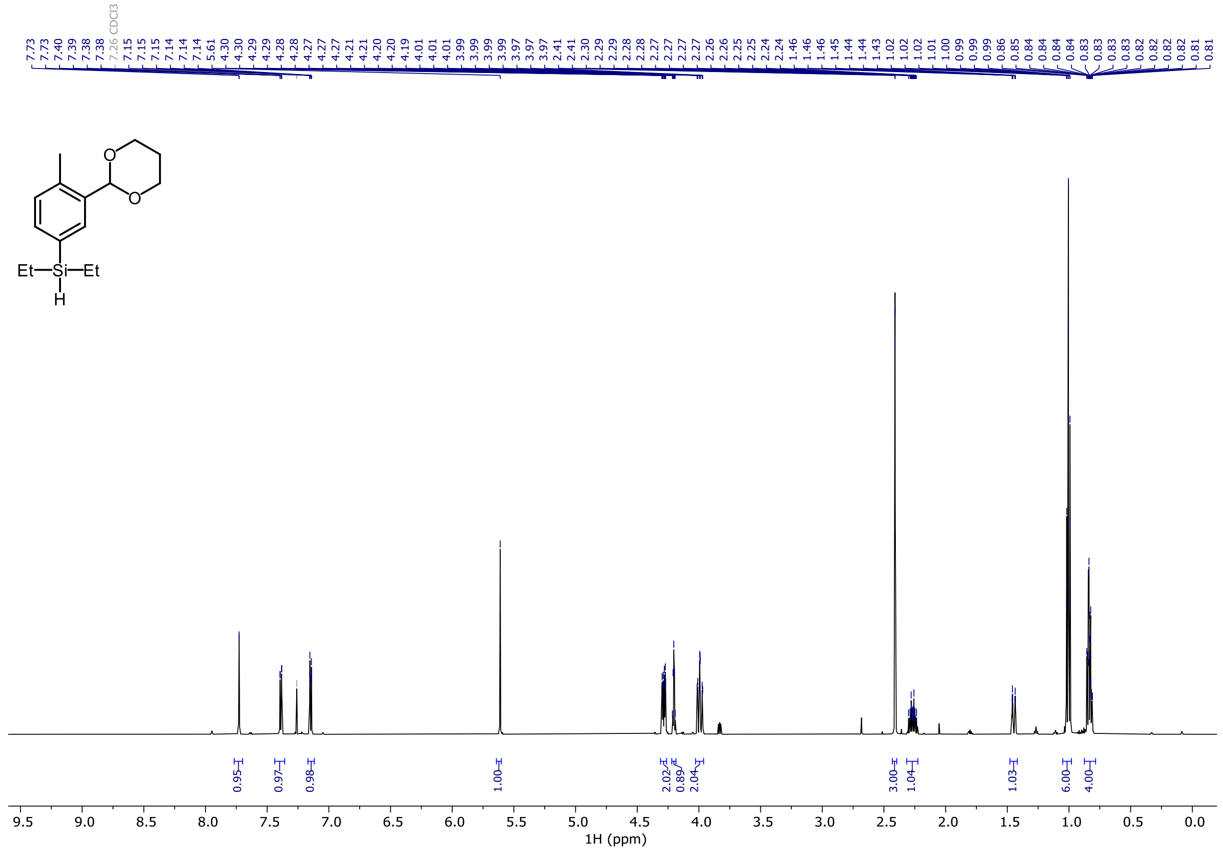

¹³C NMR of S5, 151 MHz, $CDCl_3$, 25 °C

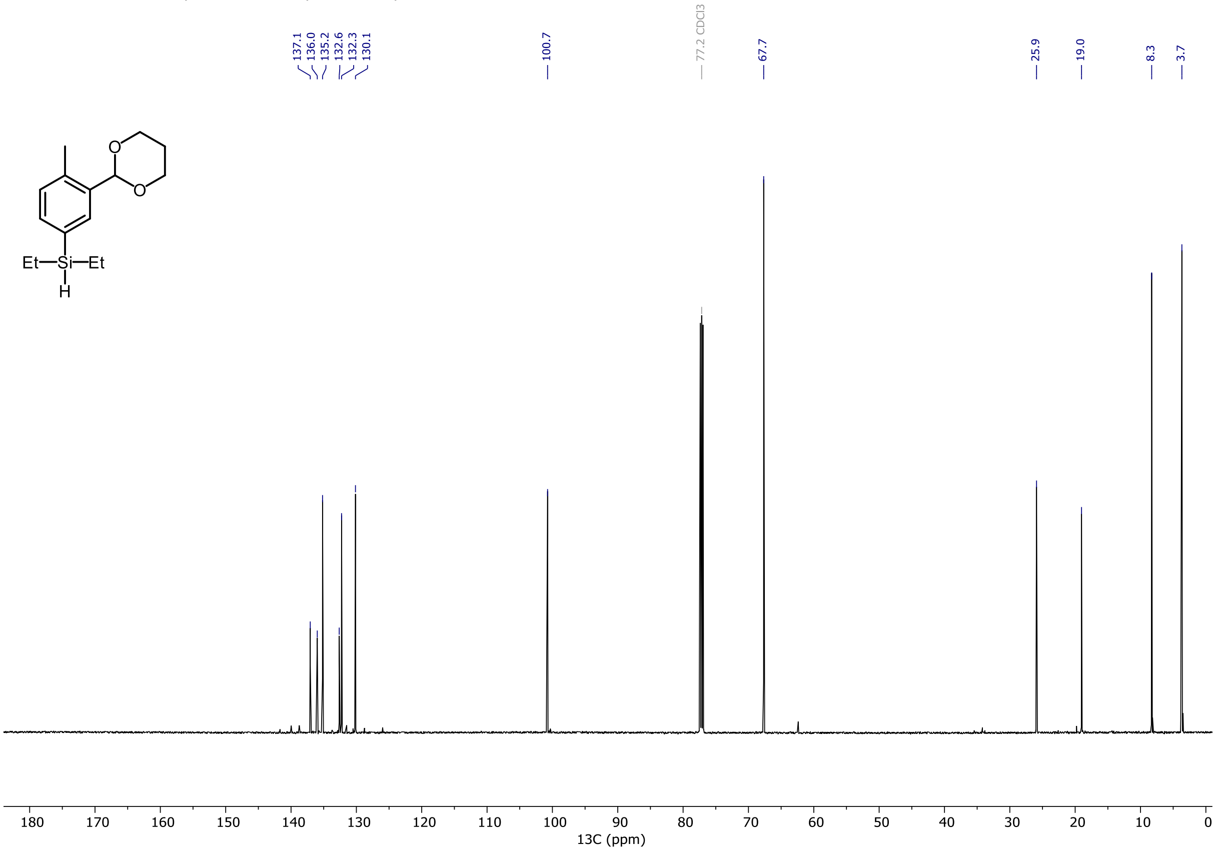

^{29}Si NMR of S5, 119 MHz, $CDCl_3$, 25 °C

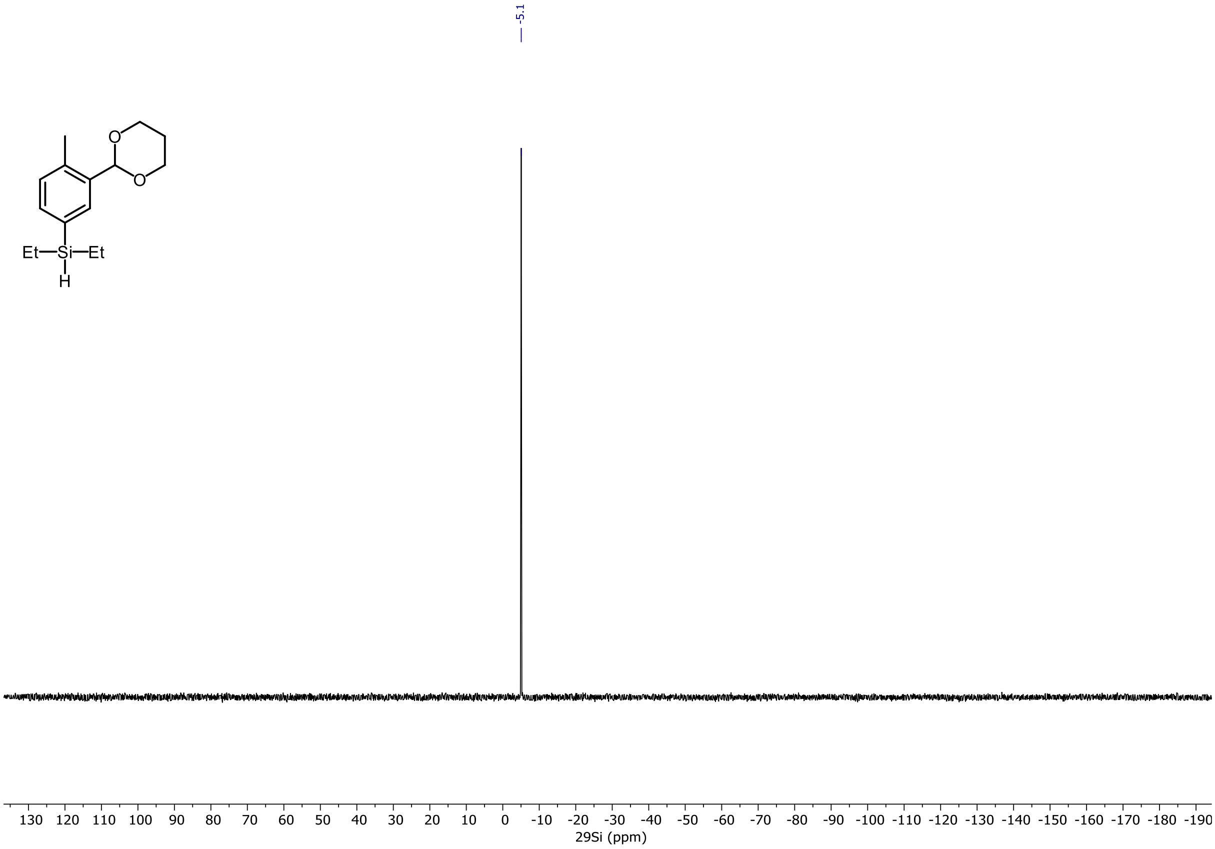

^{1}H NMR of S6, 600 MHz, $CDCl_3$, 25 °C

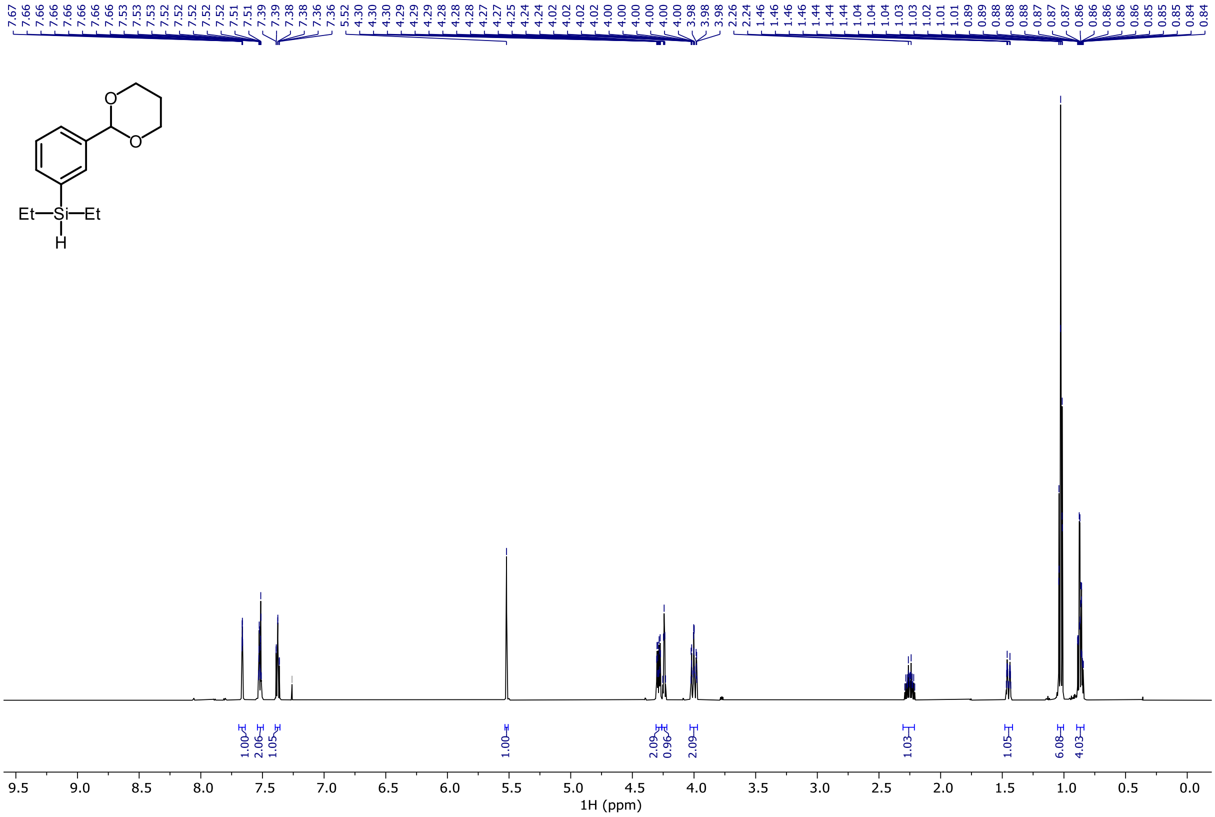

^{13}C NMR of S6, 151 MHz, $CDCl_3$, 25 °C

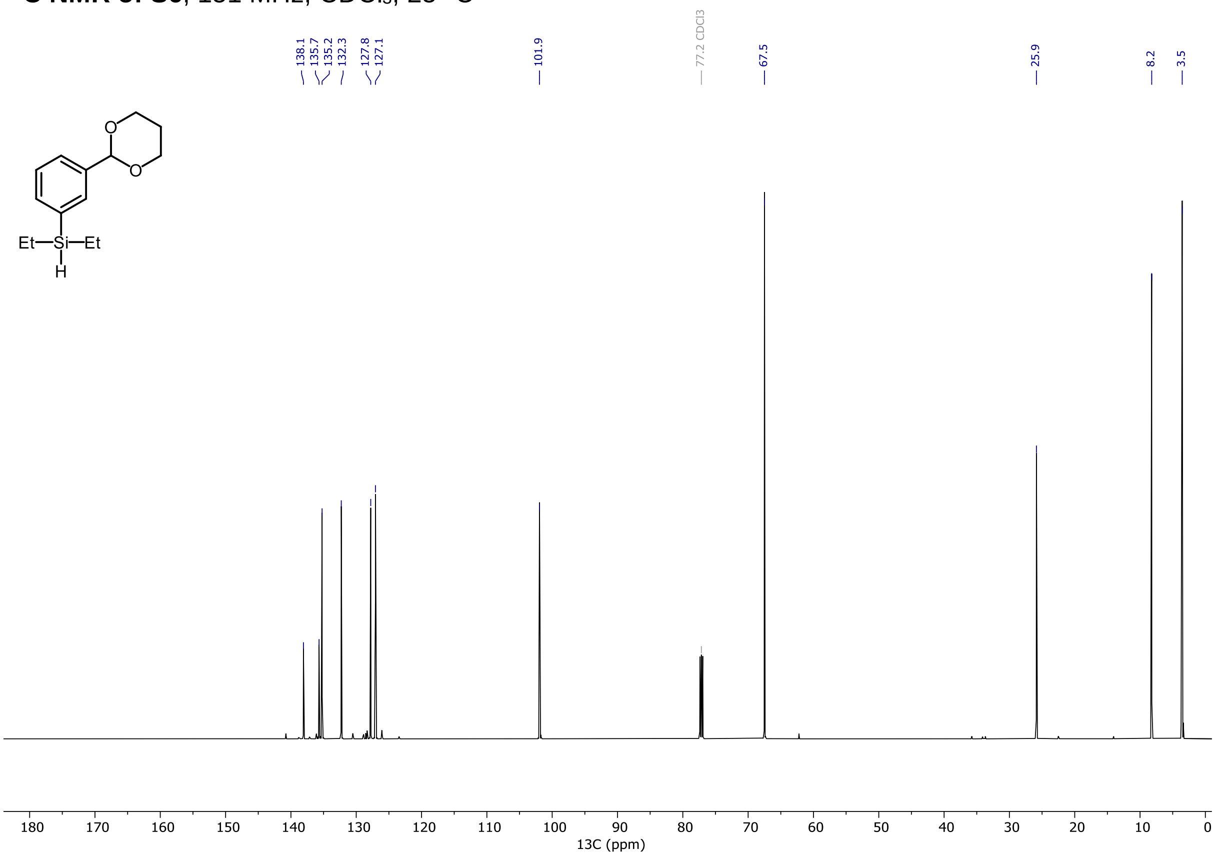

^{29}Si NMR of S6, 119 MHz, $CDCl_3$, 25 °C

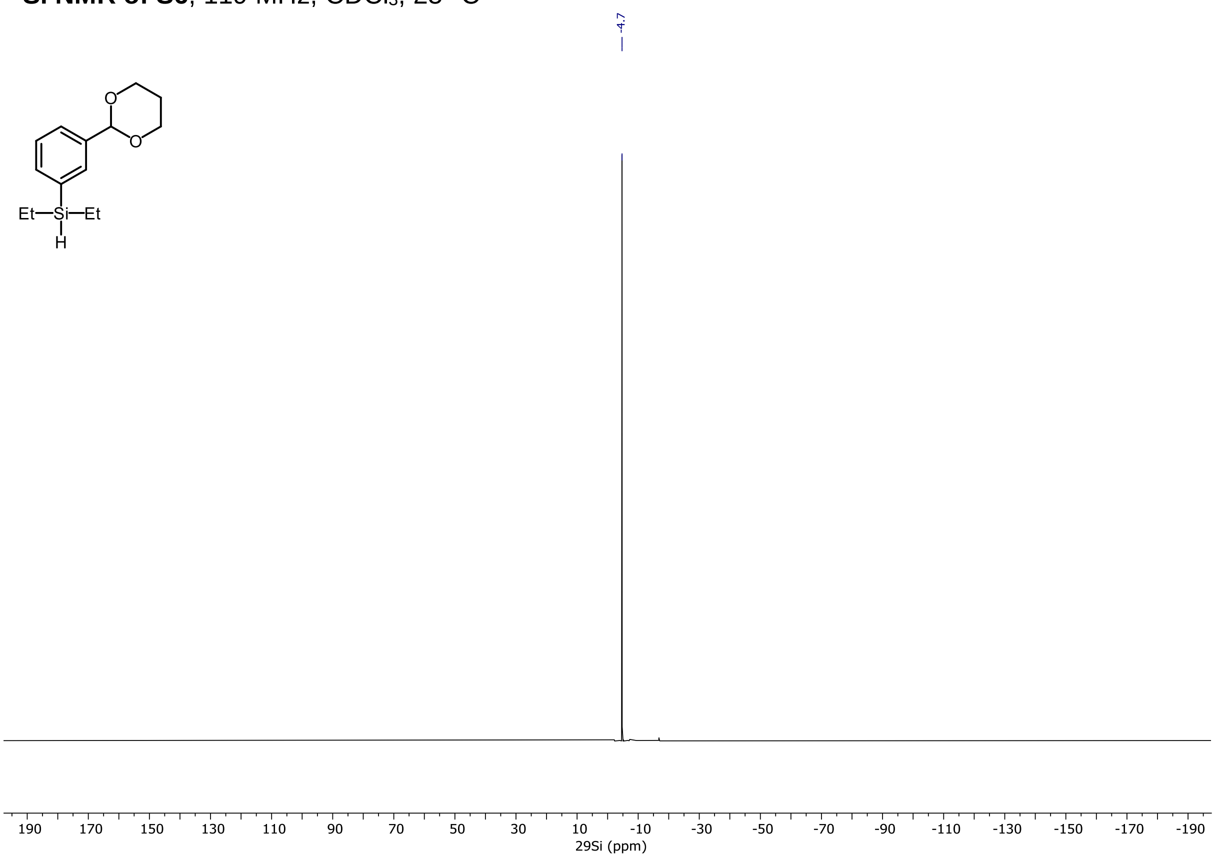

^{1}H NMR of S7, 400 MHz, $CDCl_3$, 25 °C

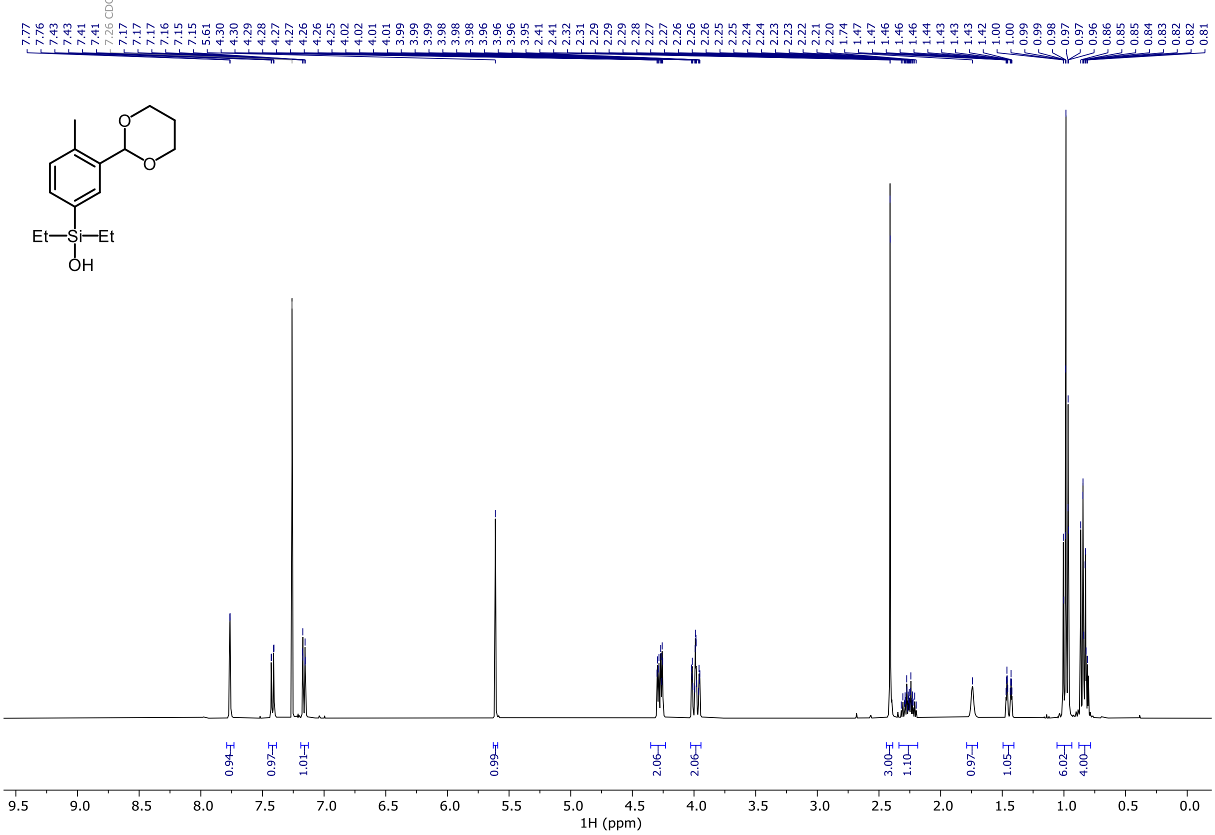

^{13}C NMR of S7, 101 MHz, $CDCl_3$, 25 °C

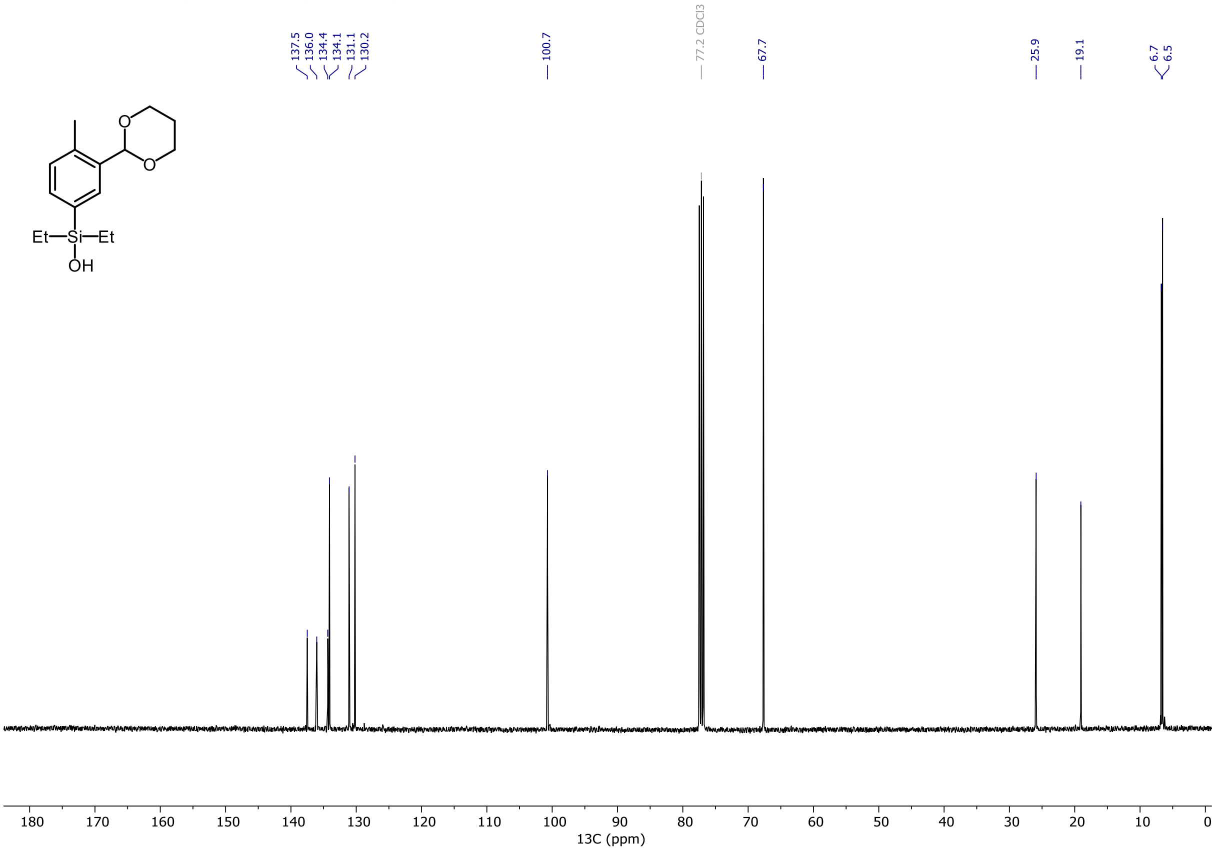

^{1}H NMR of S8, 600 MHz, $CDCl_3$, 25 °C

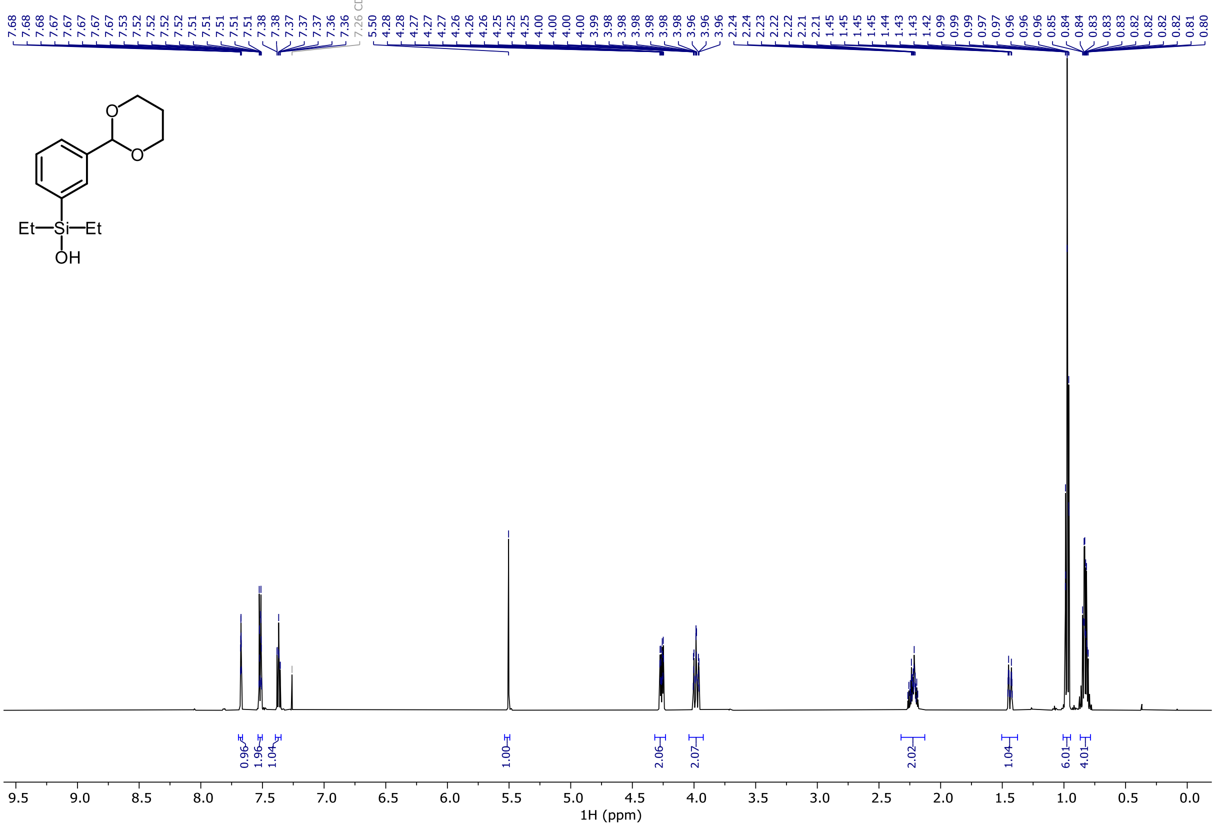

^{13}C NMR of S8, 151 MHz, $CDCl_3$, 25 °C

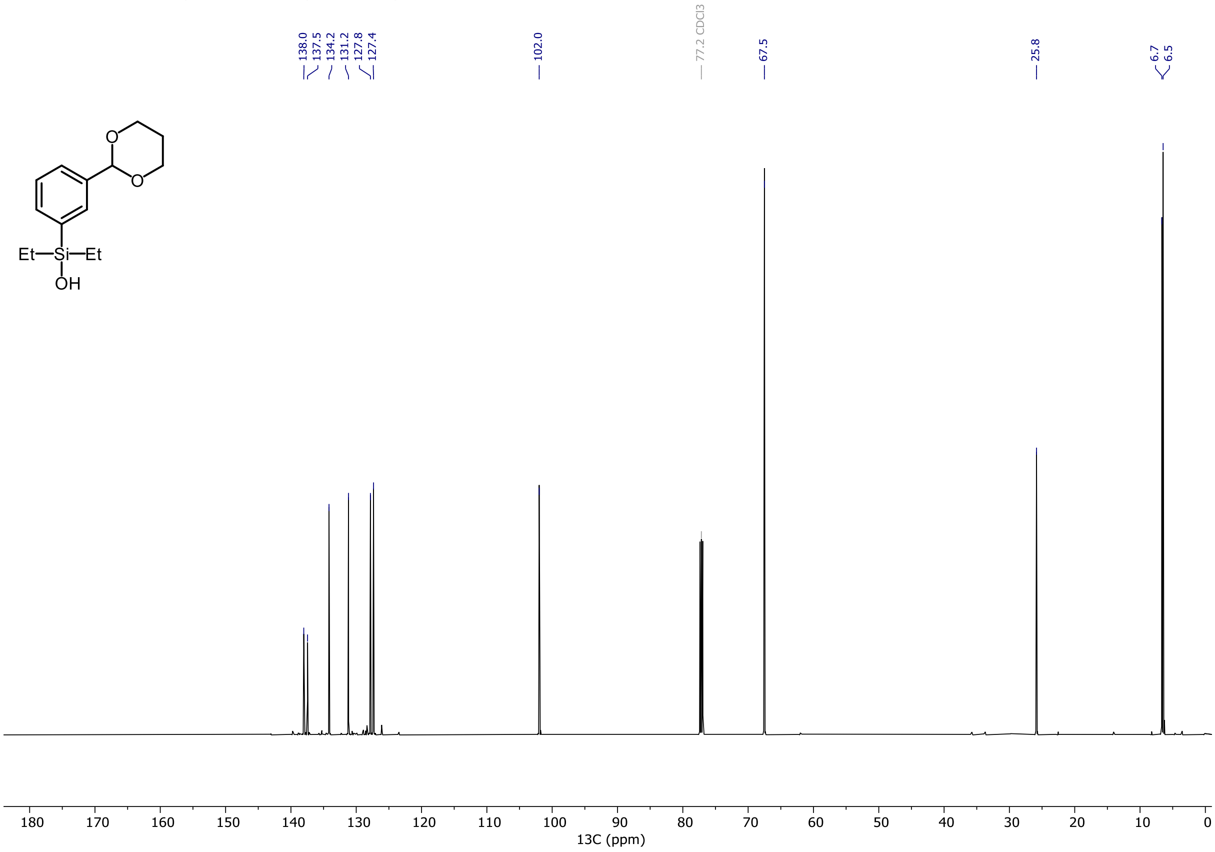

^{29}Si NMR of S8, 119 MHz, $CDCl_3$, 25 °C

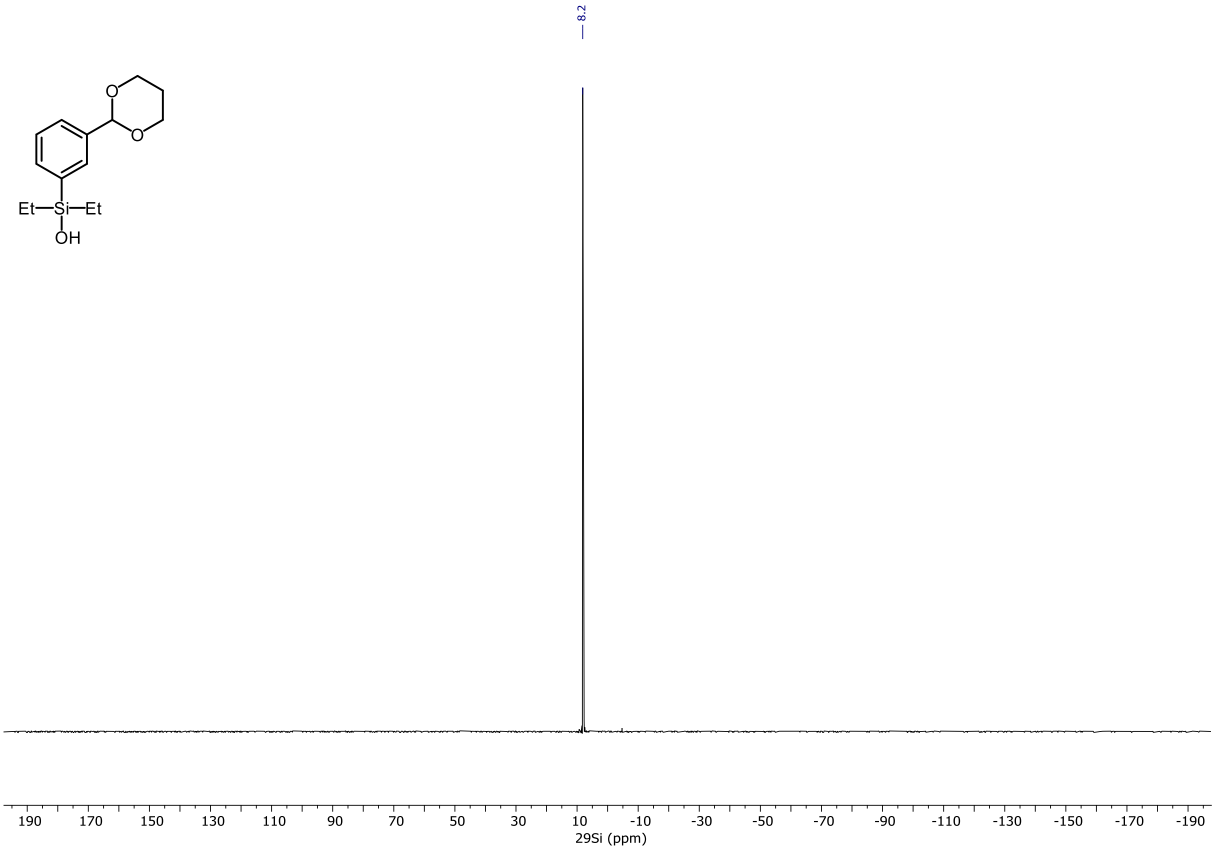

^{1}H NMR of S9, 400 MHz, $CDCl_3$, 25 °C

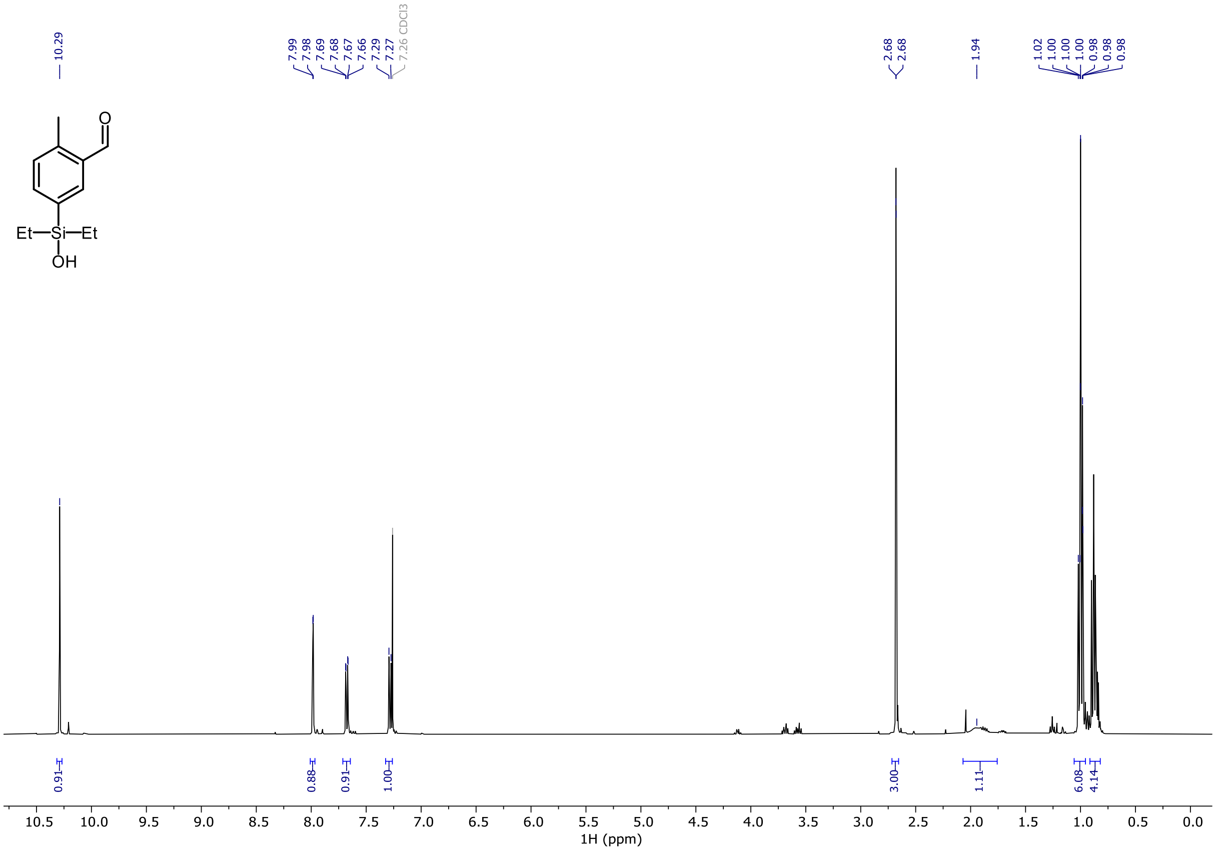

^{13}C NMR of S9, 101 MHz, $CDCl_3$, 25 °C

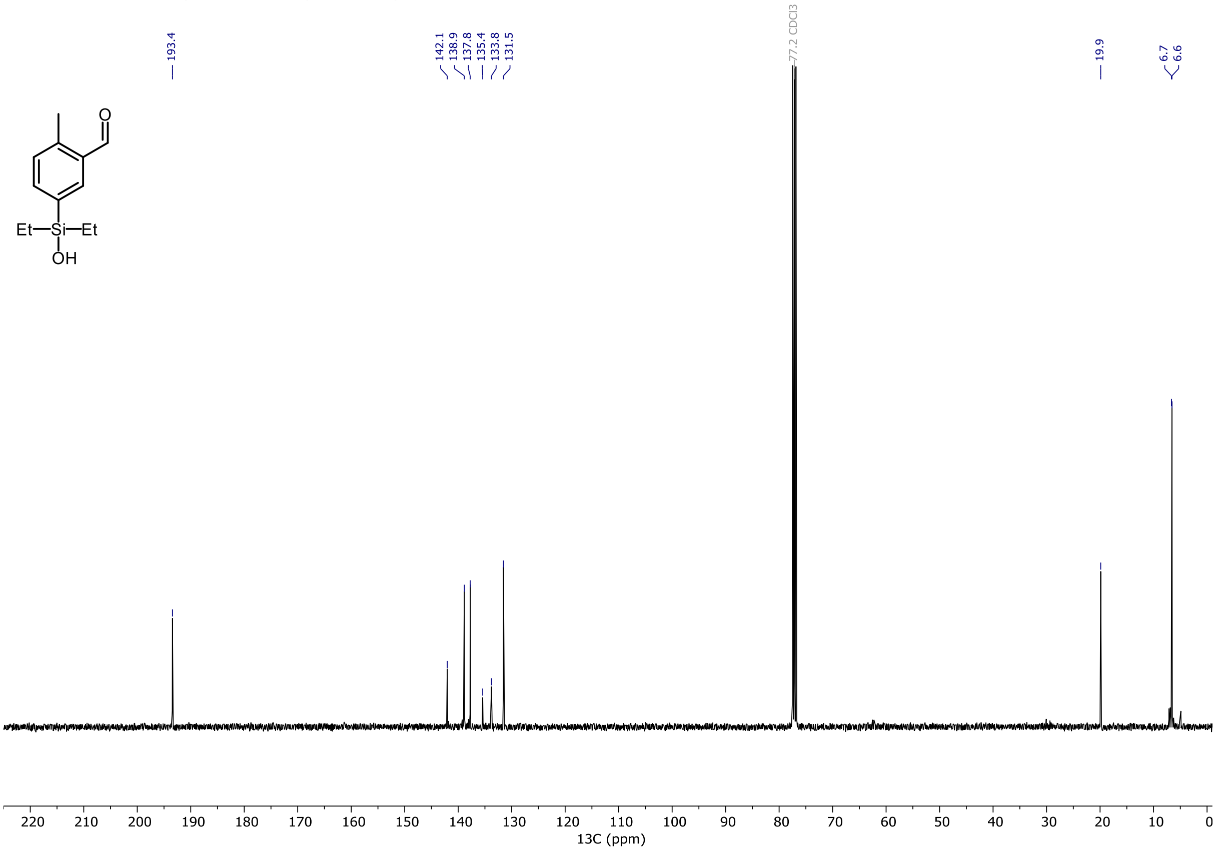

^{1}H NMR of S10, 600 MHz, CD_2Cl_2, 25 °C

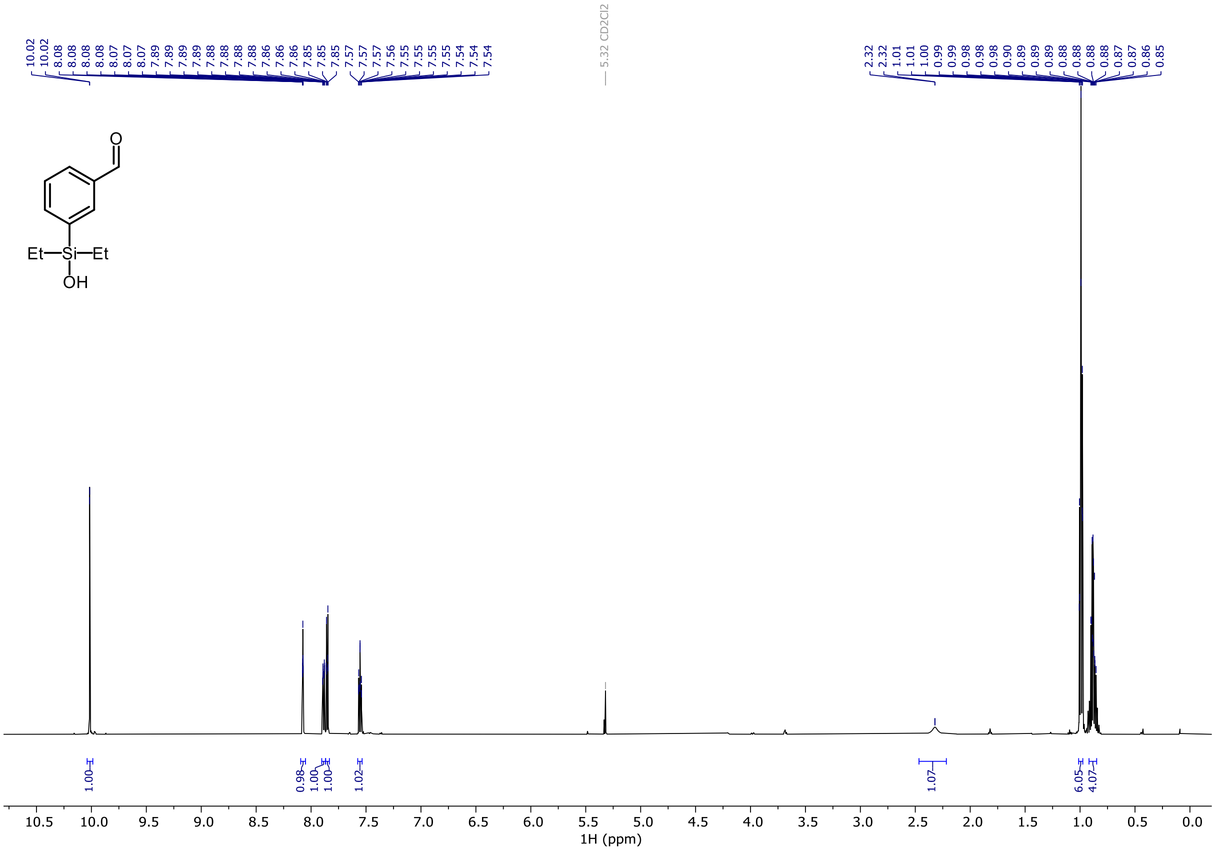

^{13}C NMR of S10, 151 MHz, CD_2Cl_2, 25 °C

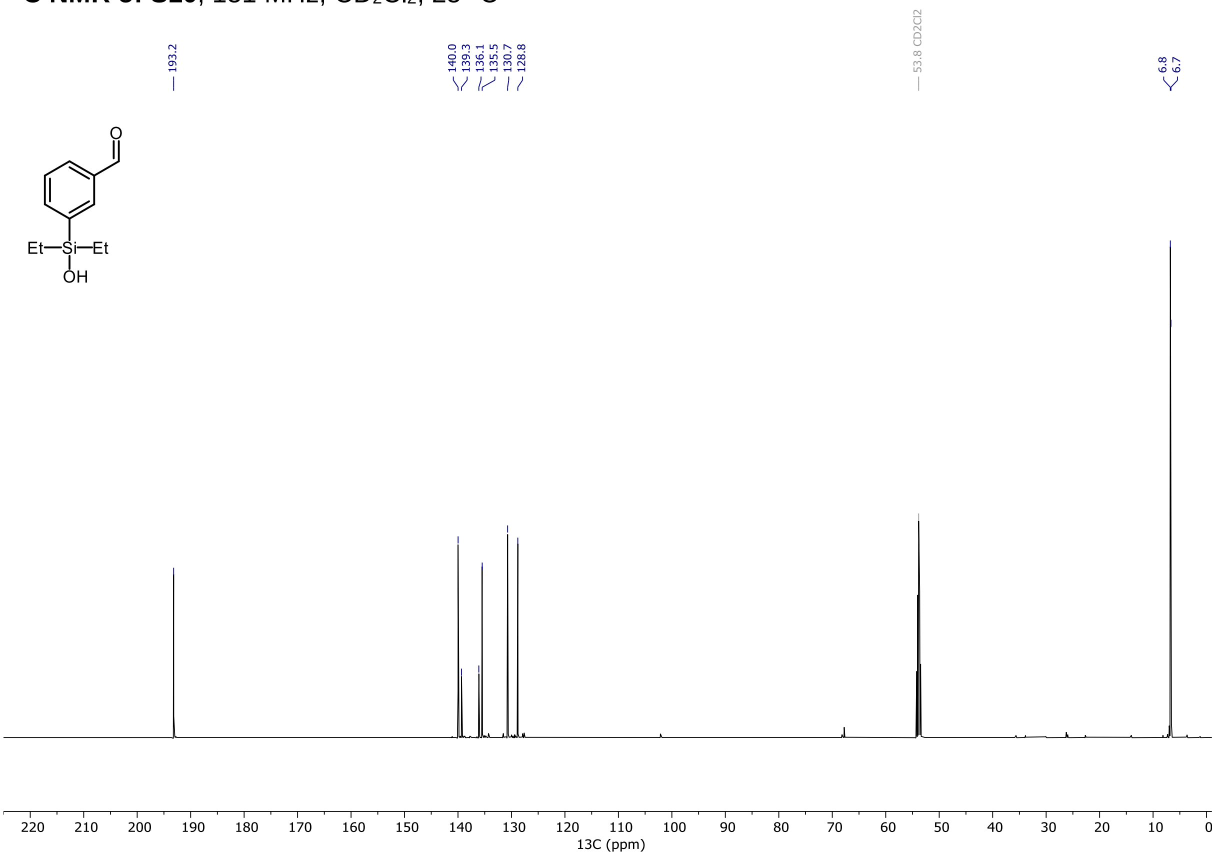

^{29}Si NMR of S10, 119 MHz, CD_2Cl_2, 25 °C

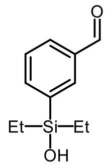

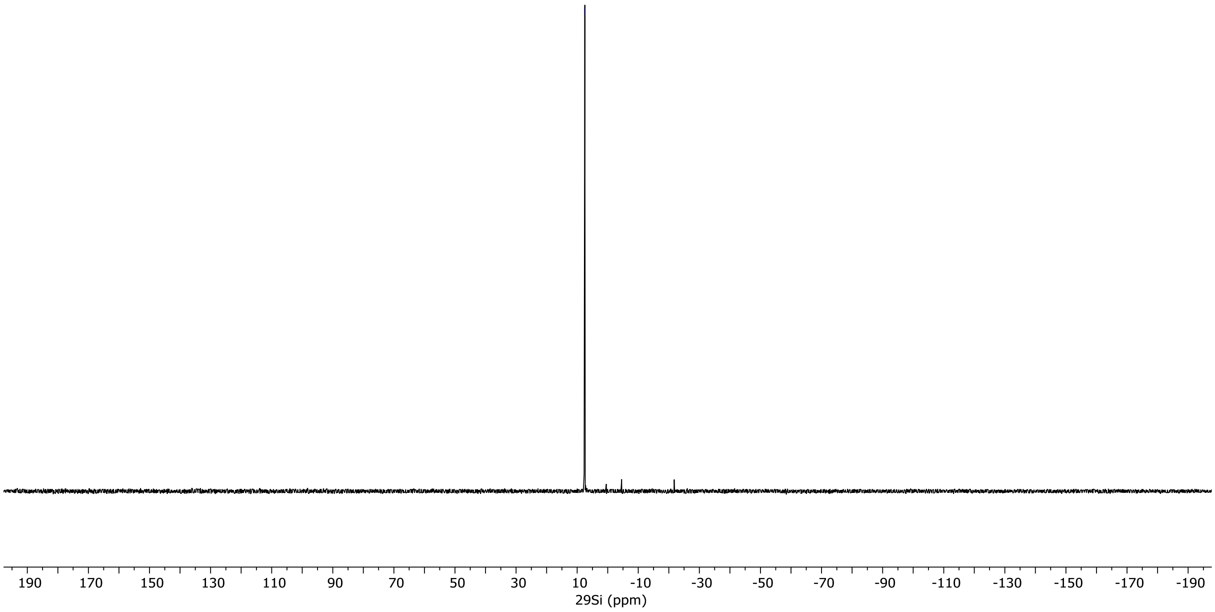

^{1}H NMR of ligand S11, 600 MHz, $CDCl_3$, 25 °C

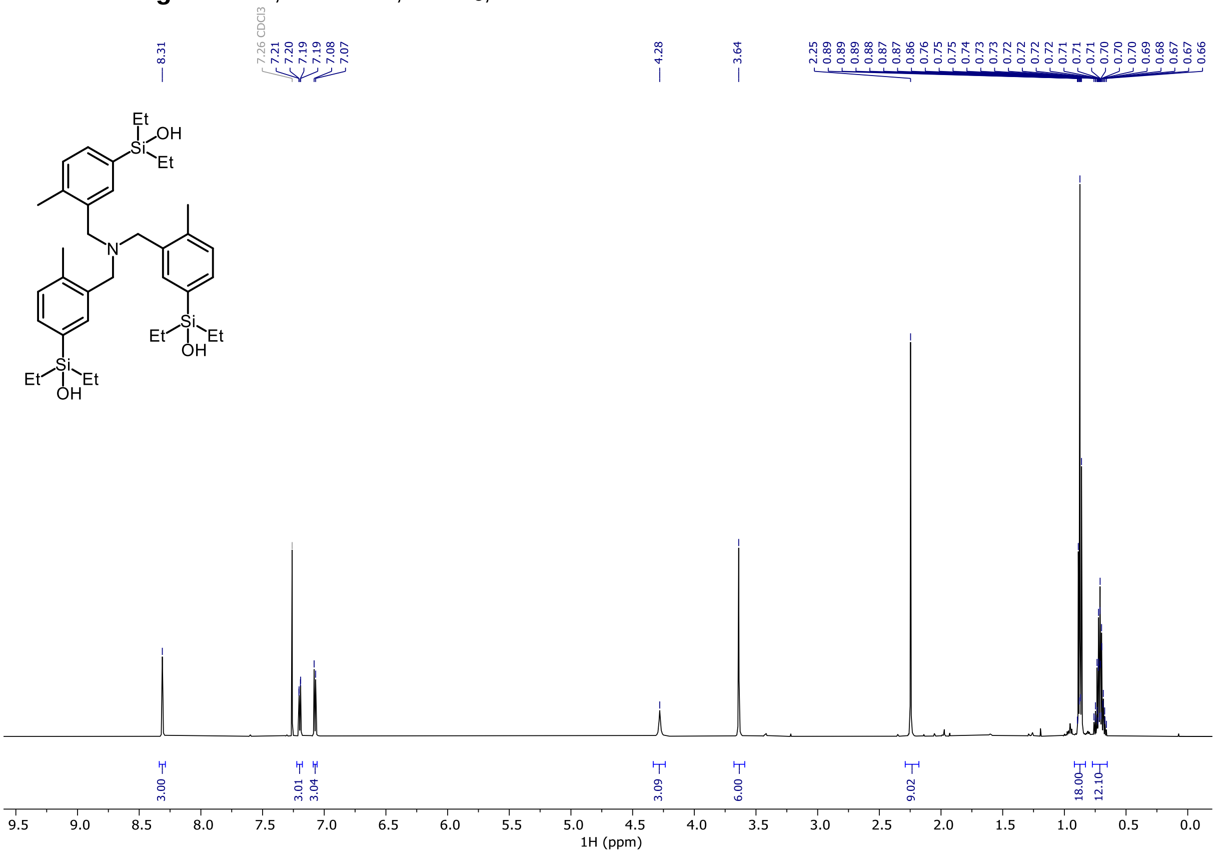

^{13}C NMR of ligand S11, 151 MHz, $CDCl_3$, 25 °C

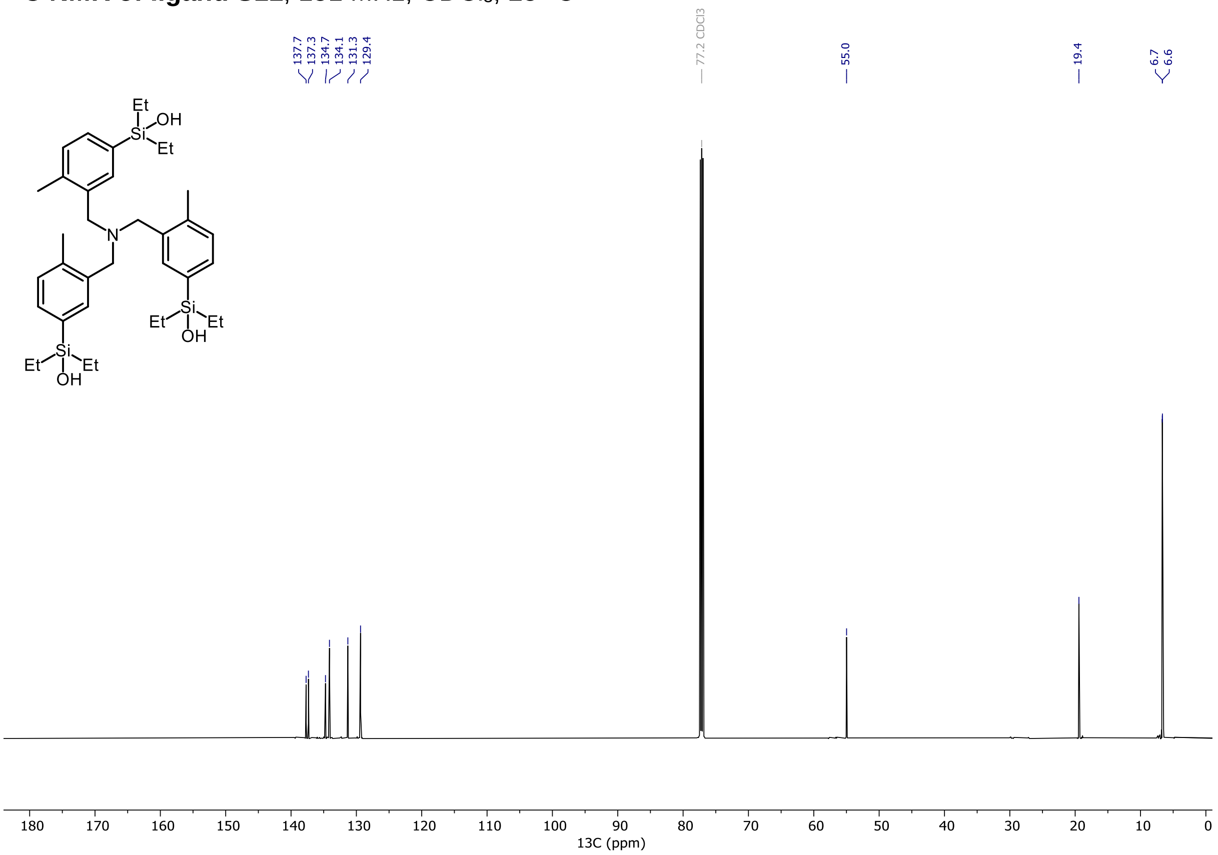

^{29}Si NMR of ligand S11, 119 MHz, $CDCl_3$, 25 °C

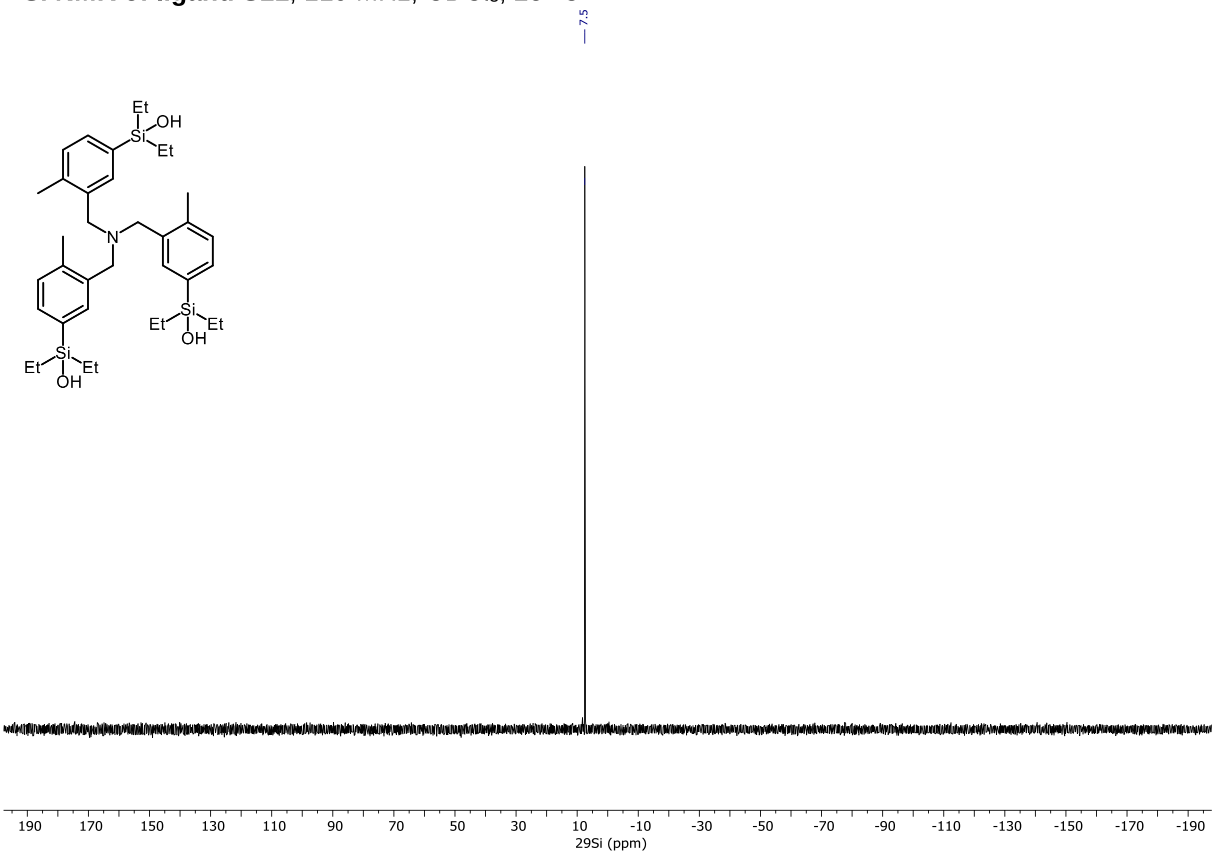

^{1}H NMR of ligand S12, 600 MHz, $CDCl_3$, 25 °C

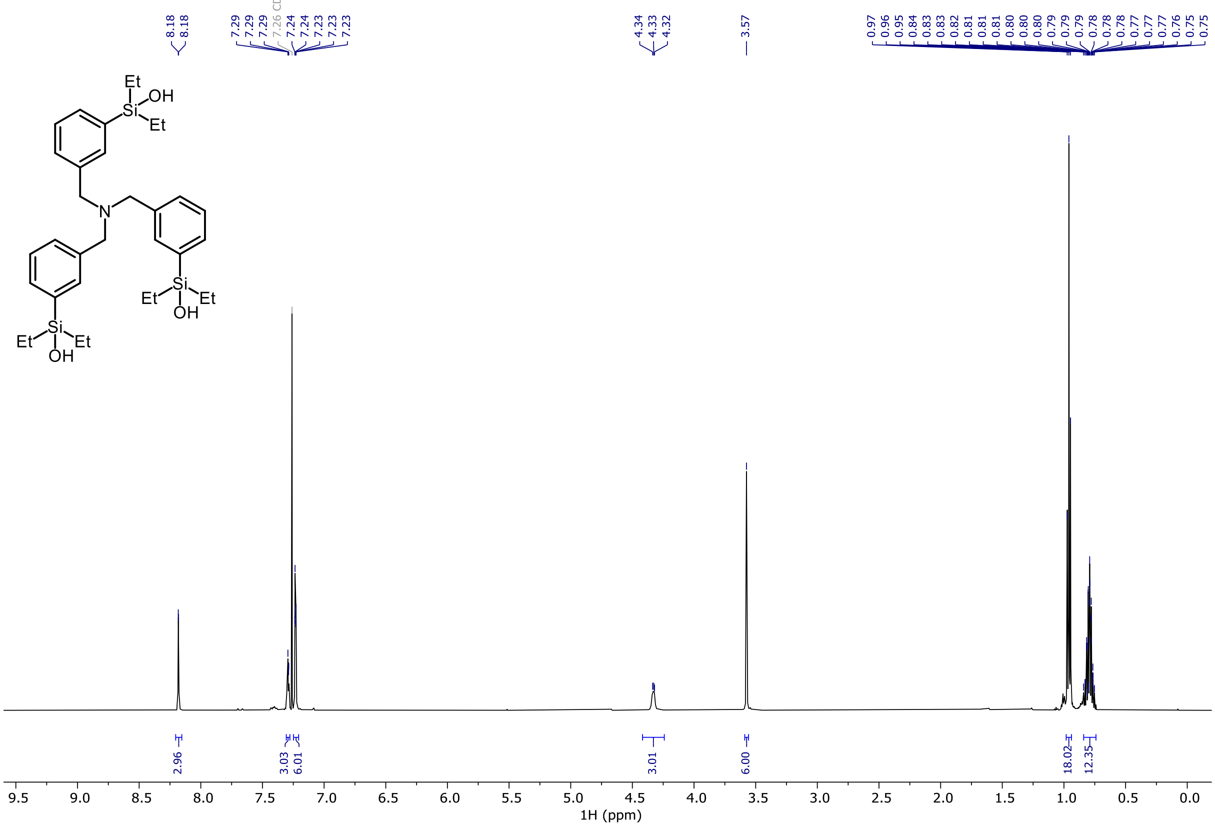

^{13}C NMR of S12, 151 MHz, $CDCl_3$, 25 °C

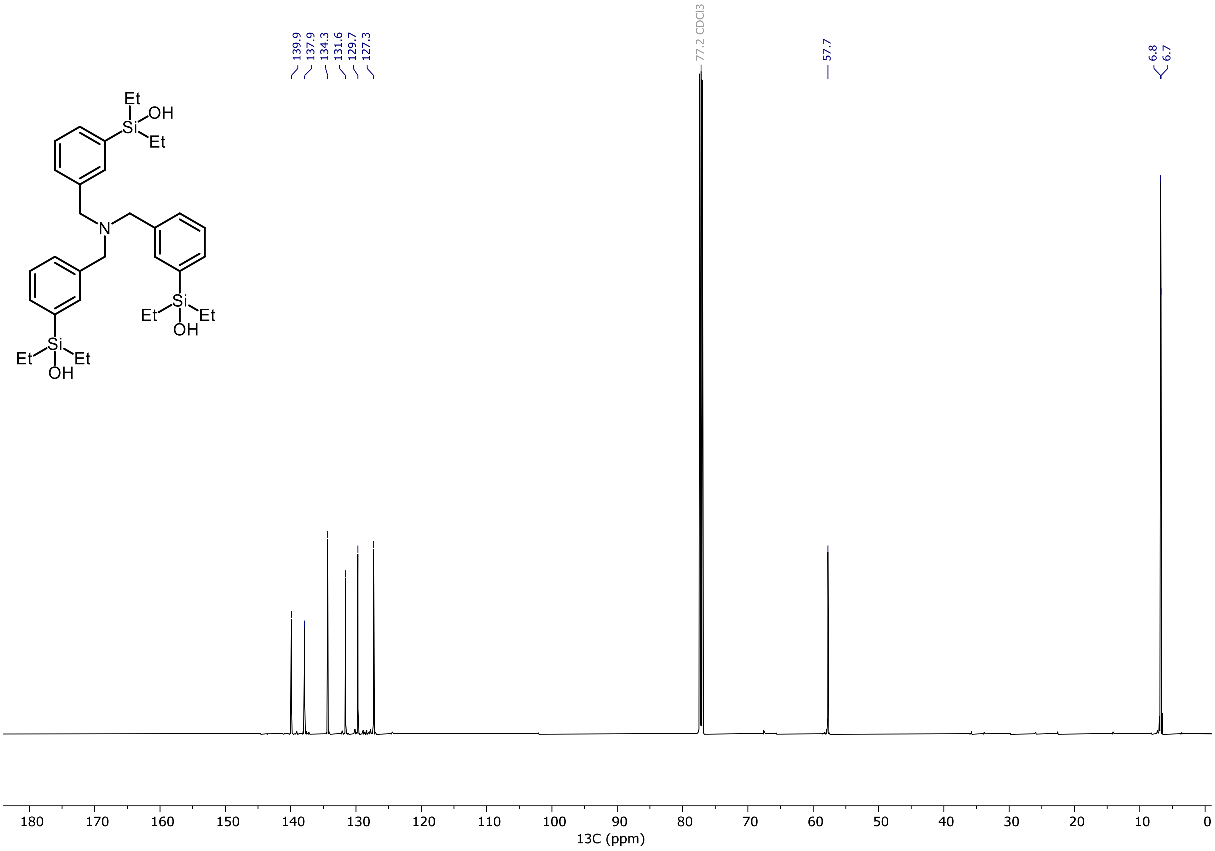

^{29}Si NMR of S12, 119 MHz, $CDCl_3$, 25 °C

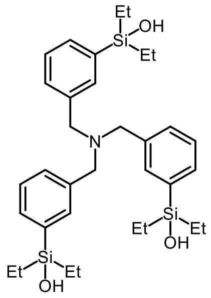

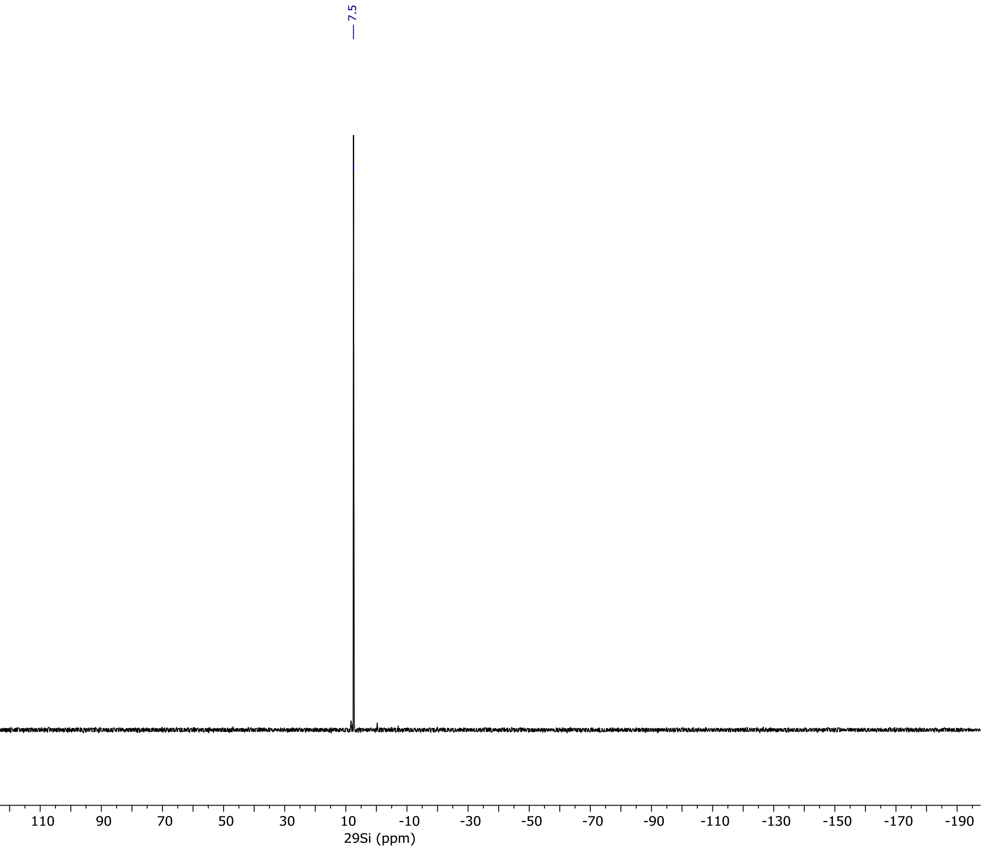

Complexes

^{1}H NMR of 10, 600 MHz, C_6D_6, 25 °C

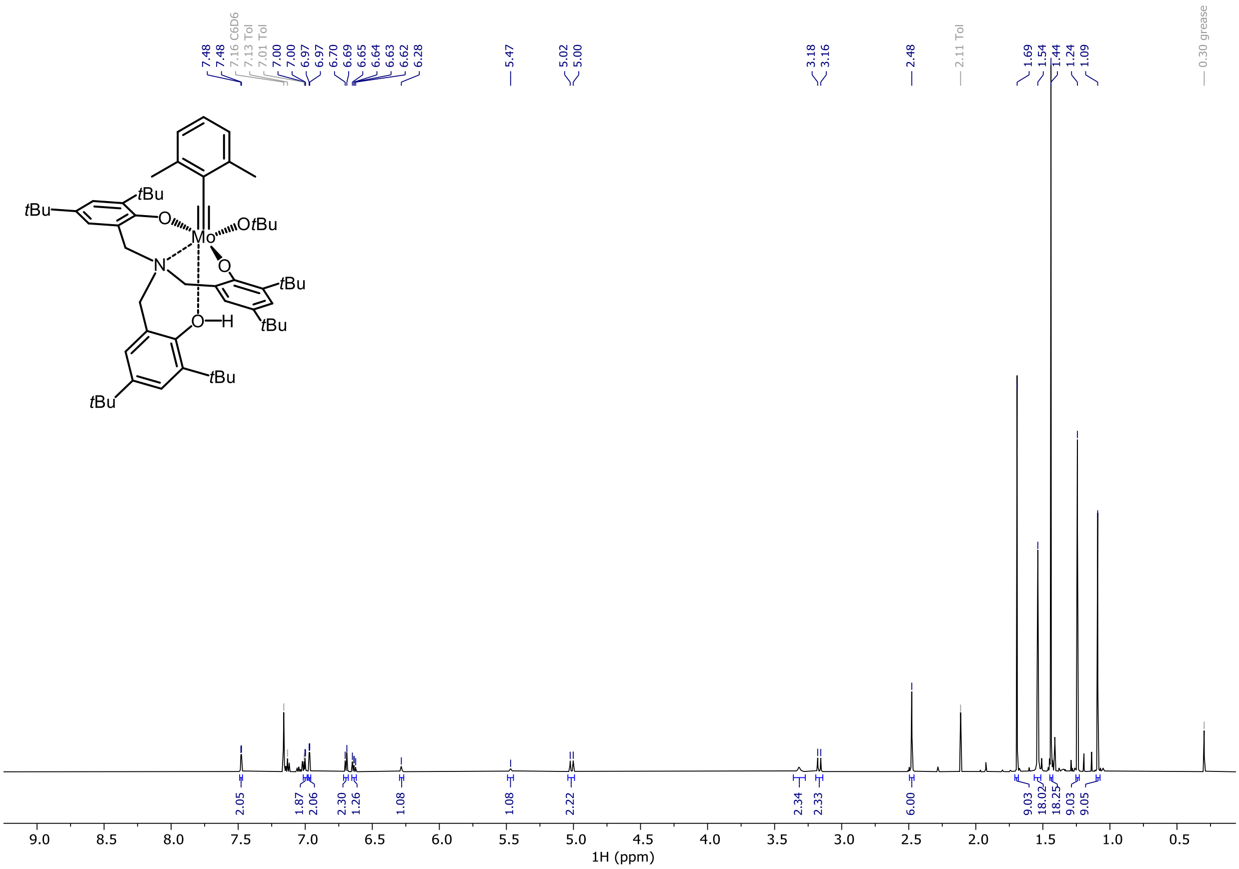

^{13}C NMR of 10, 151 MHz, C_6D_6, 25 °C

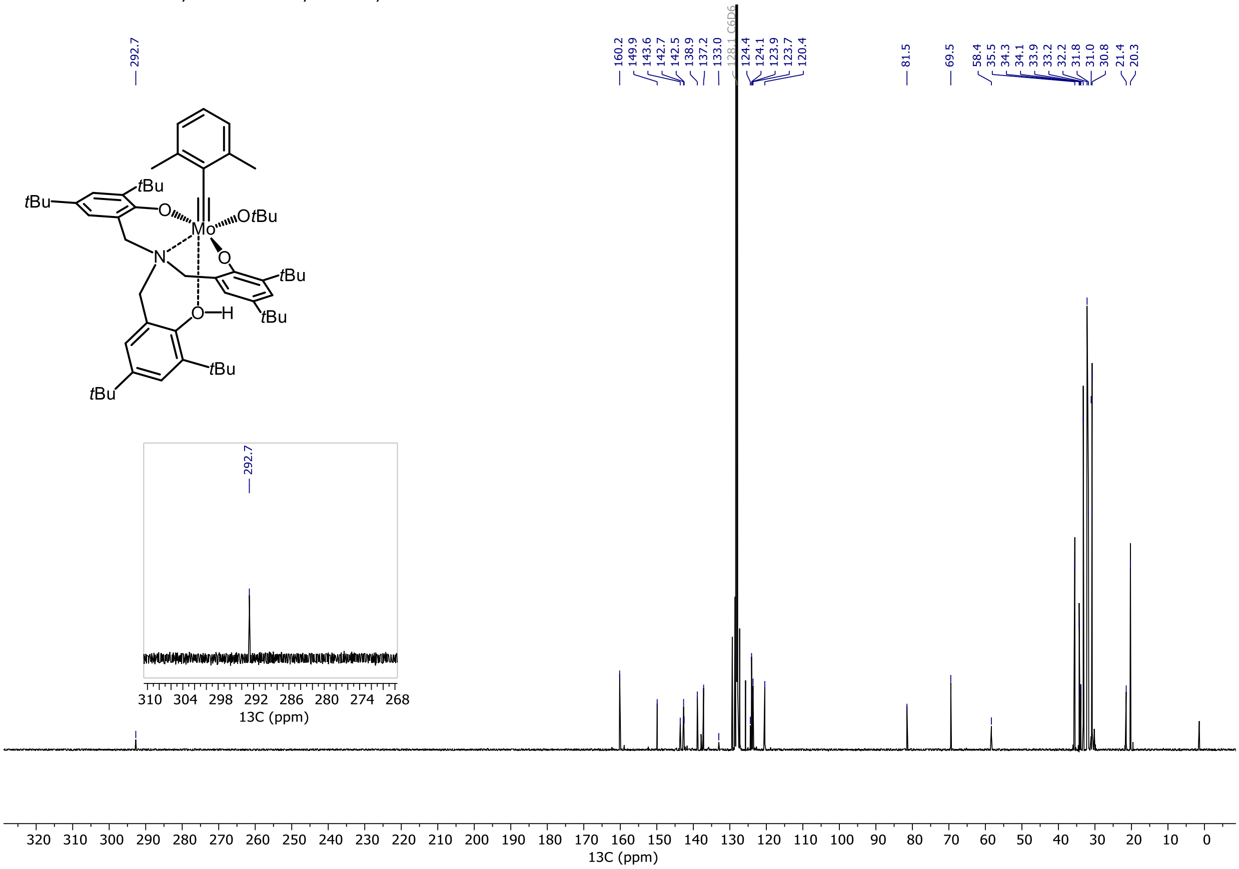

^{95}Mo NMR of 10, 26 MHz, C_6D_6, 25 °C

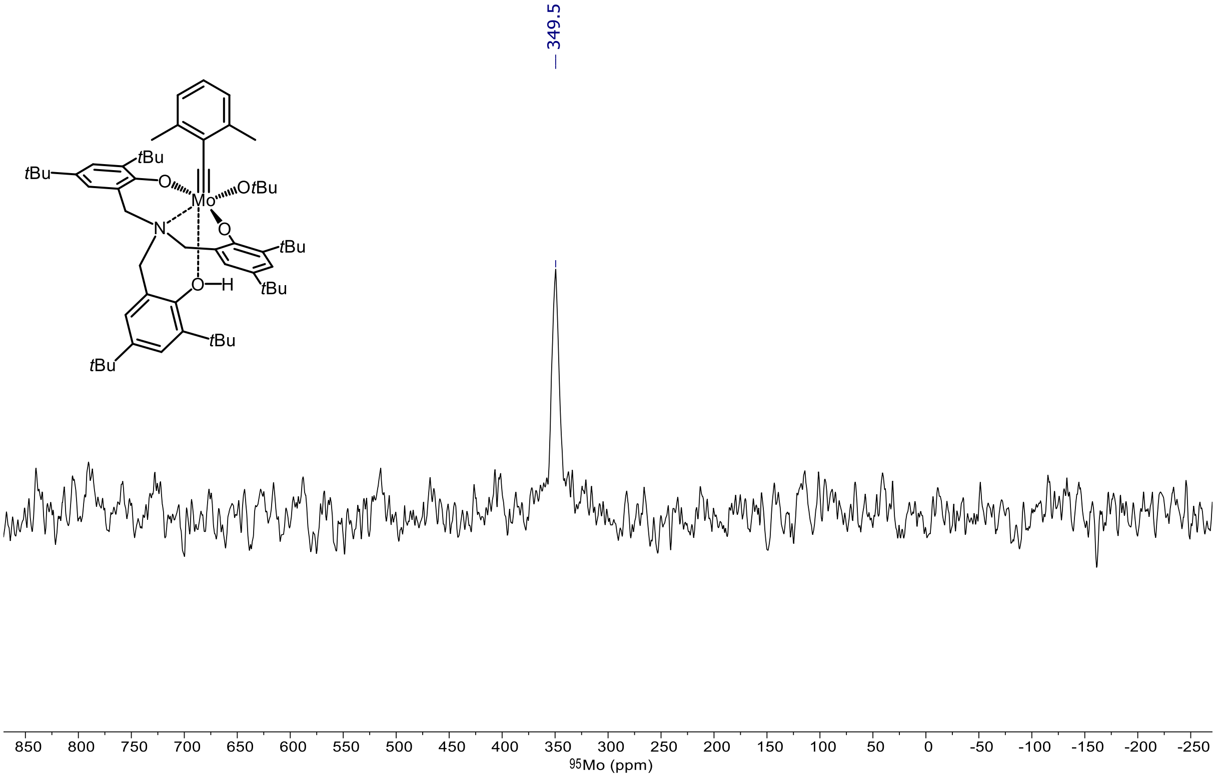

^{1}H -^{15}N HMBC of 10, 61 MHz, C_6D_6, 25 °C

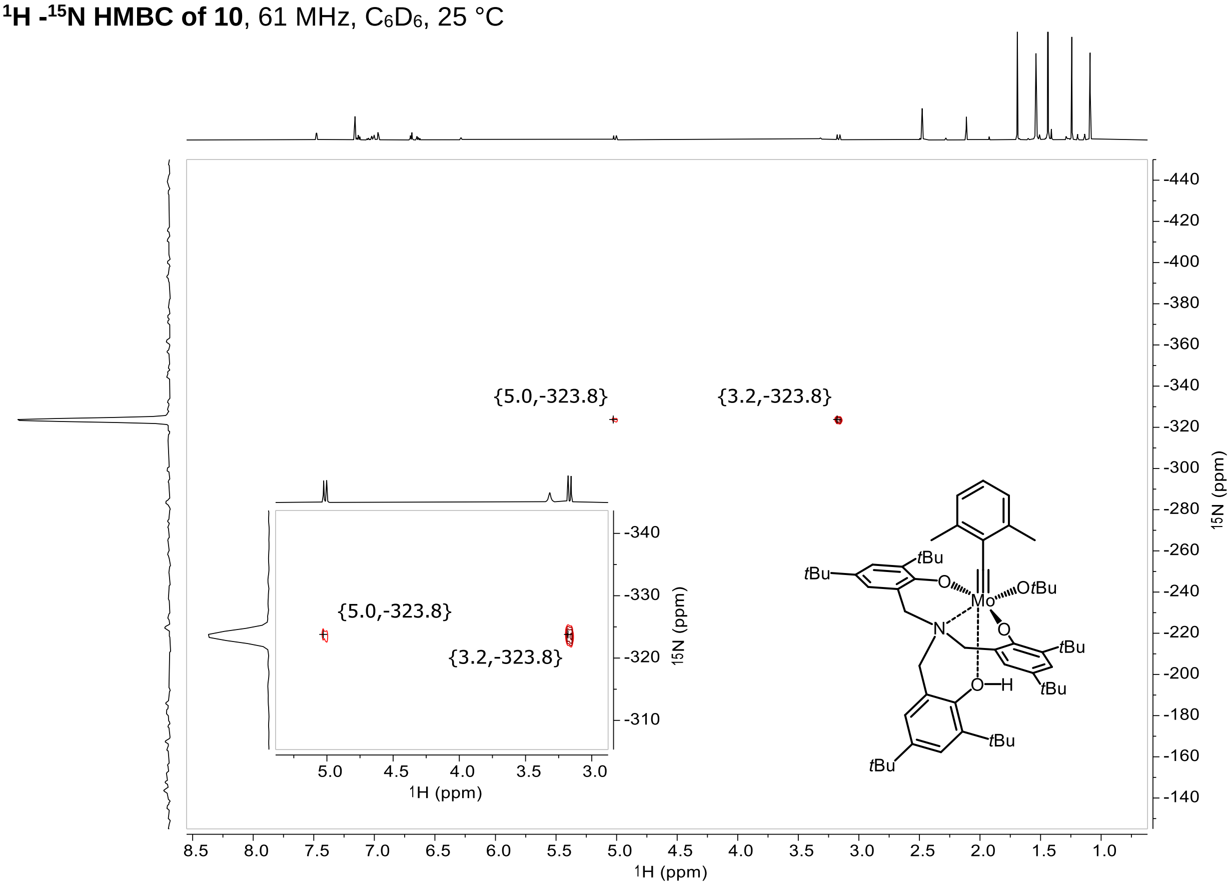

^{1}H NMR of 15a, 400 MHz, [D$_8$]-toluene, 25 °C

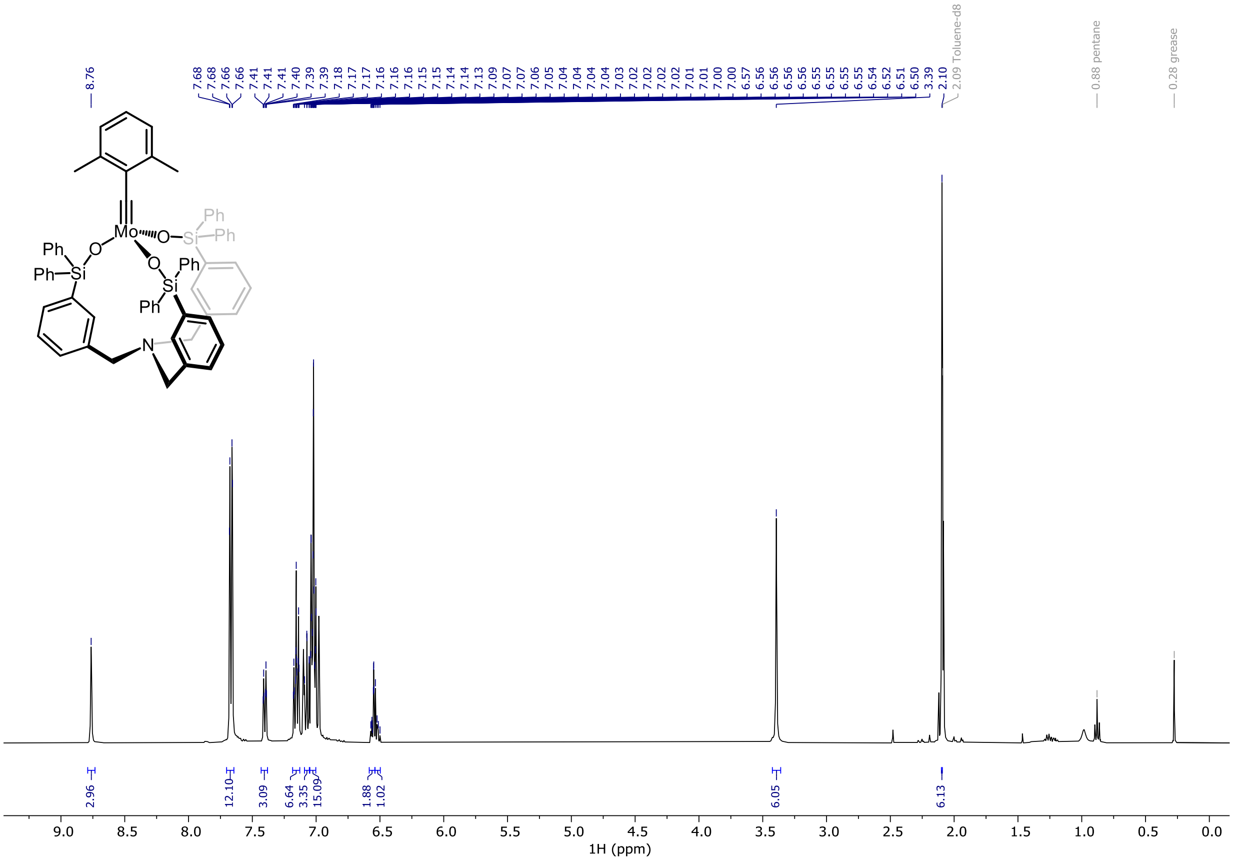

^{13}C NMR of 15a, 151 MHz, [D$_8$]-toluene, 25 °C

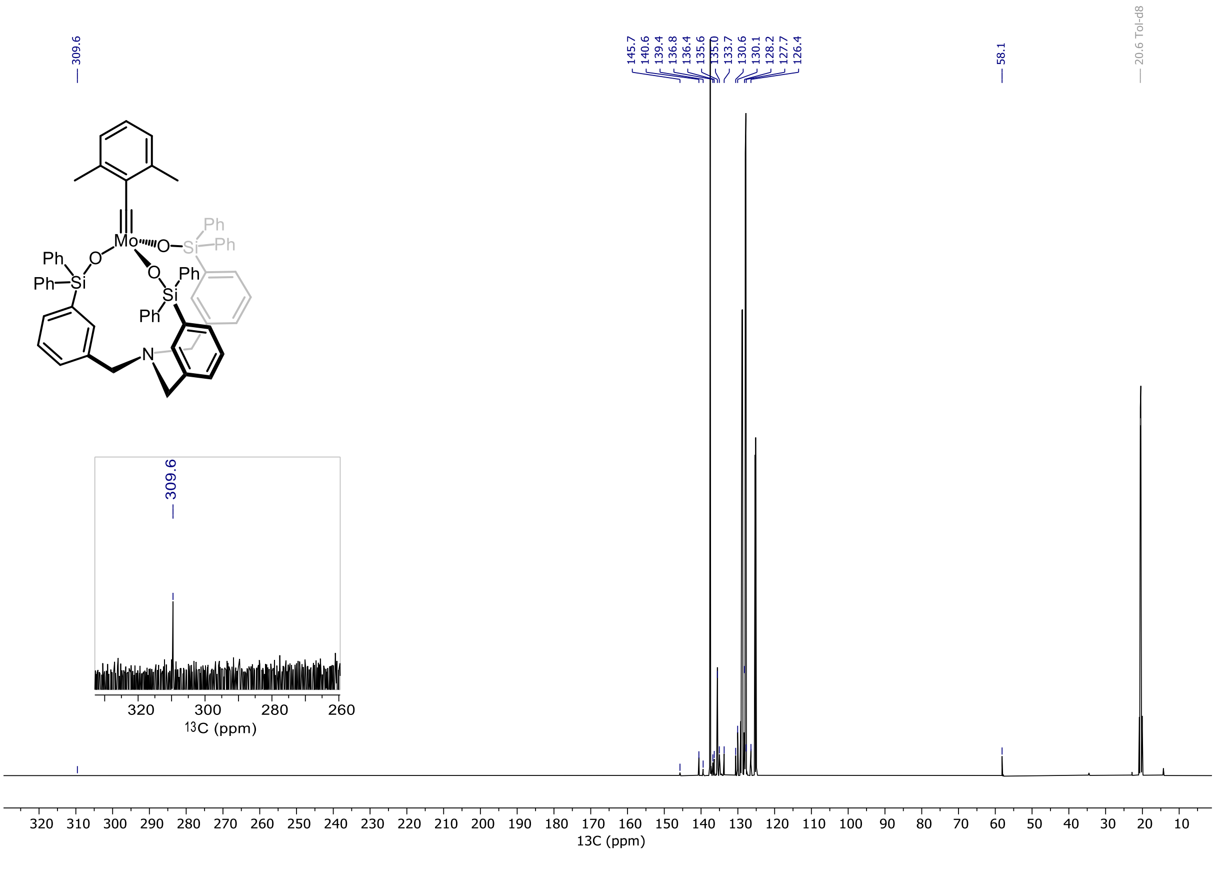

^{29}Si NMR of 15a, 60 MHz, [D8]-toluene, 25 °C

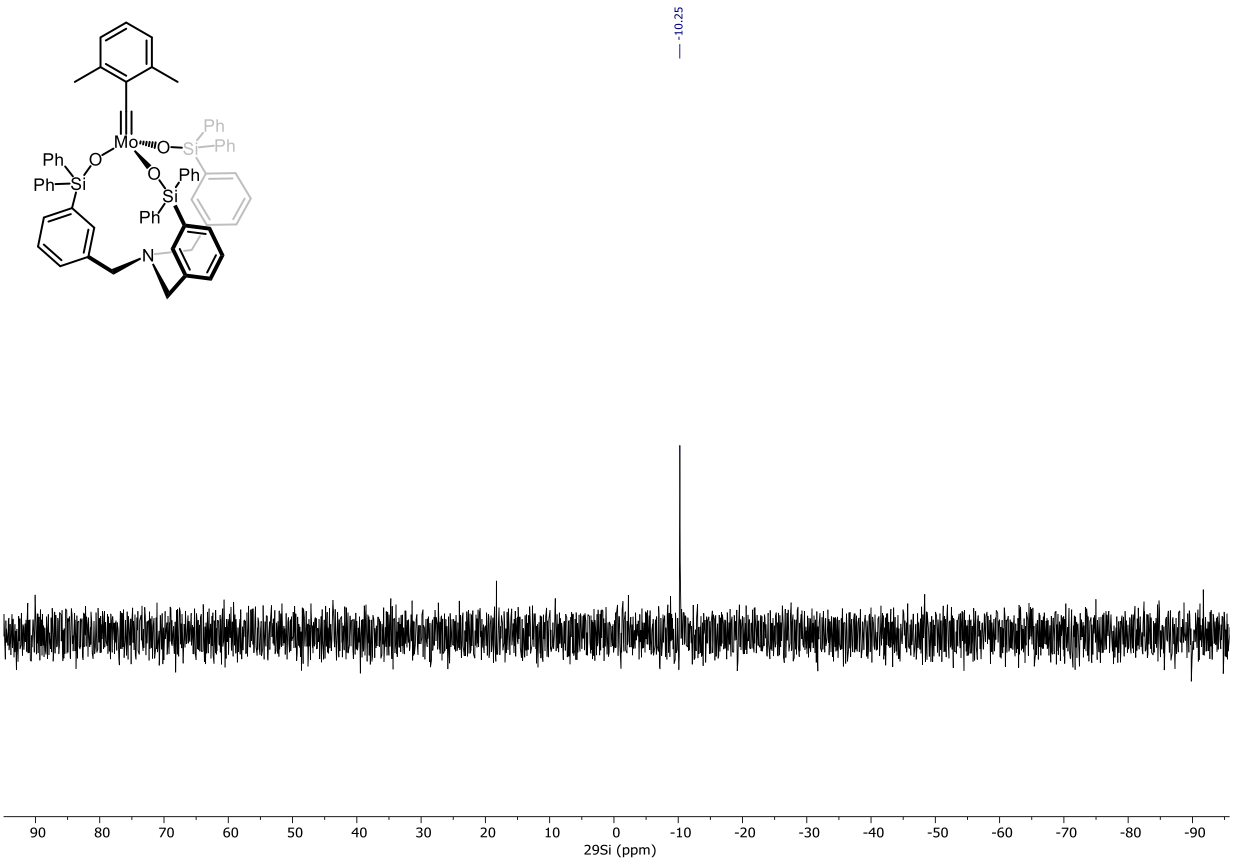

^{95}Mo NMR of 15a, 26 MHz, [D8]-toluene, 60 °C

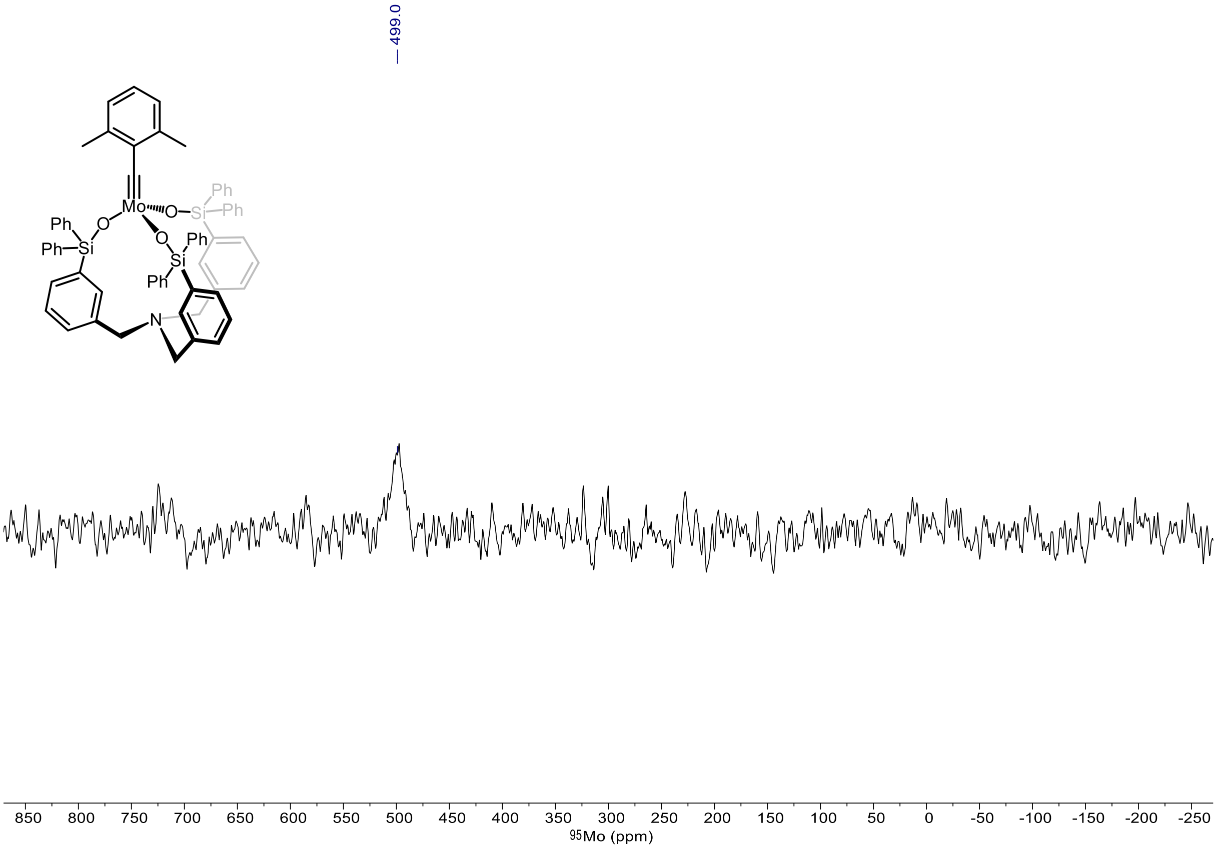

^{1}H NMR of 15b, 600 MHz, [D$_8$]-toluene, 25 °C

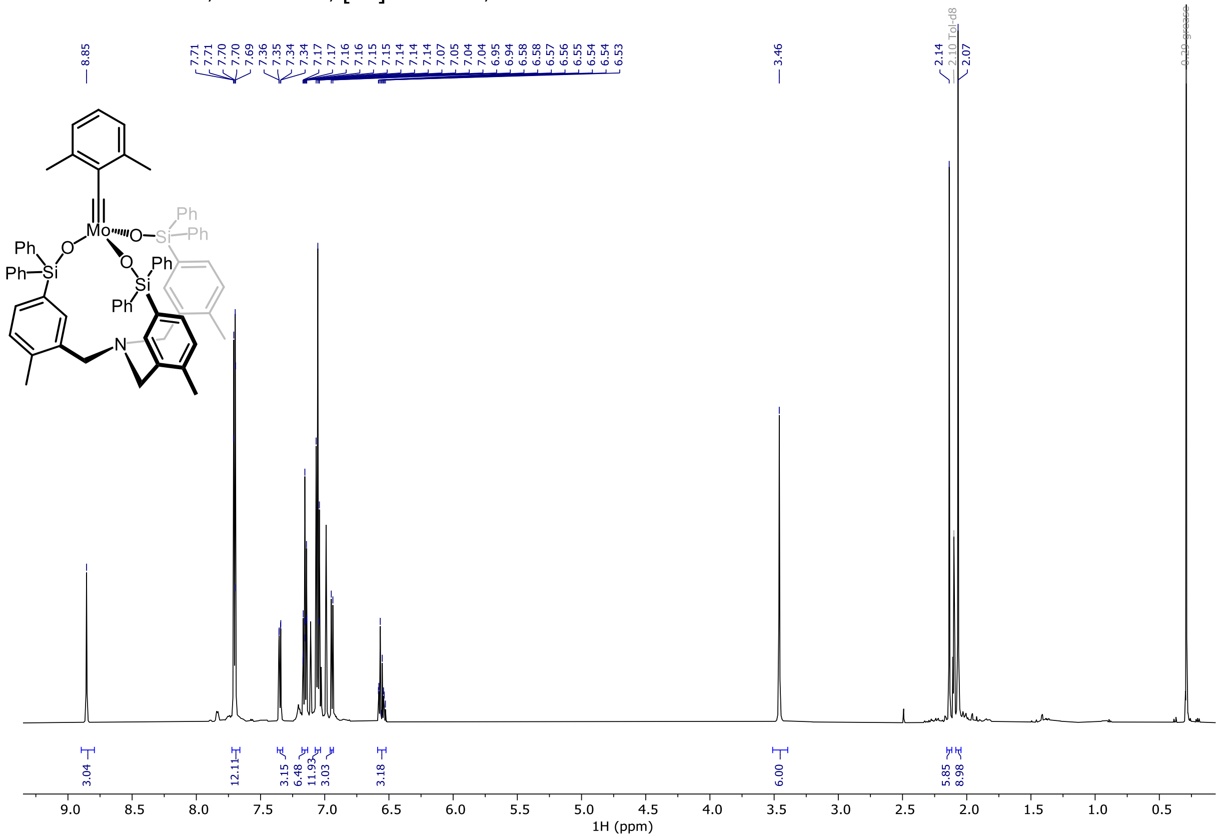

^{13}C NMR of 15b, 151 MHz, [D$_8$]-toluene, 25 °C

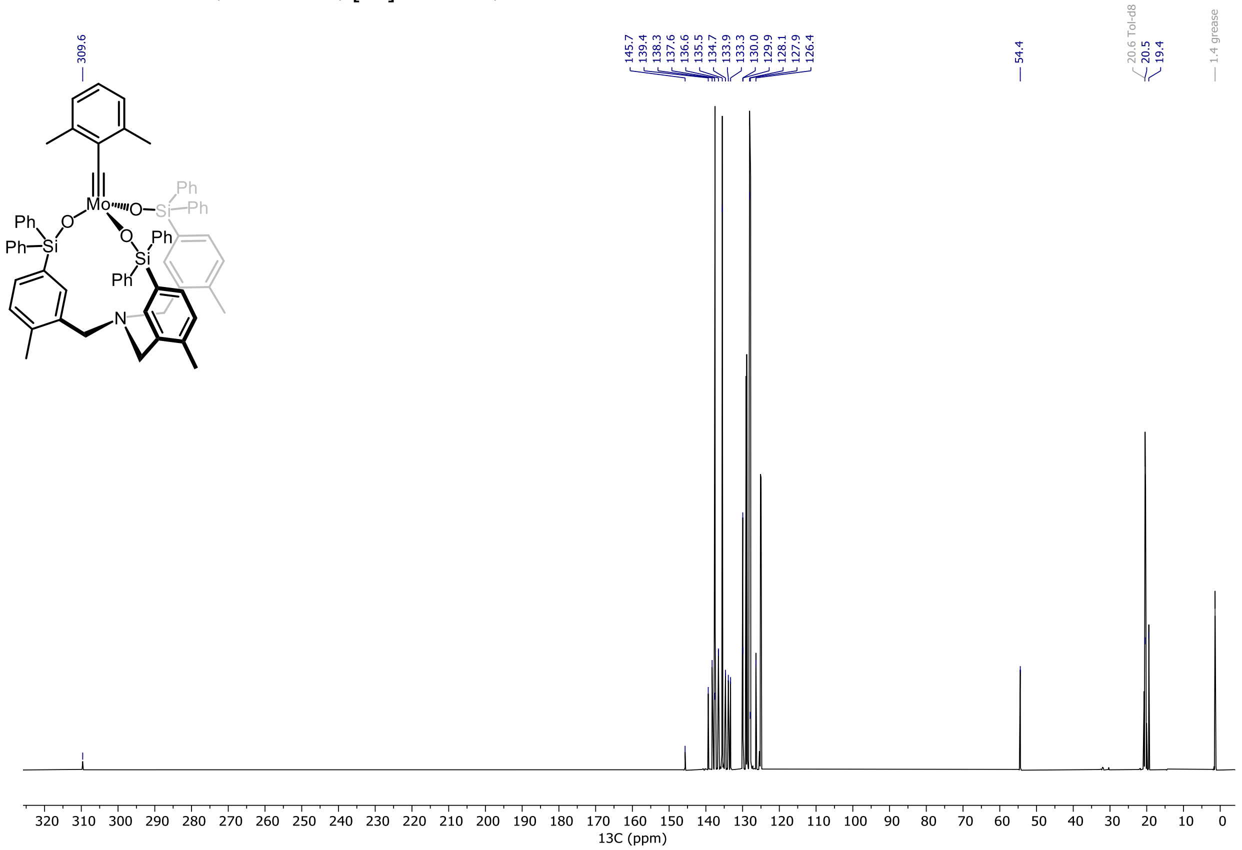

^{29}Si NMR of 15b, 60 MHz, [D8]-toluene, 25 °C

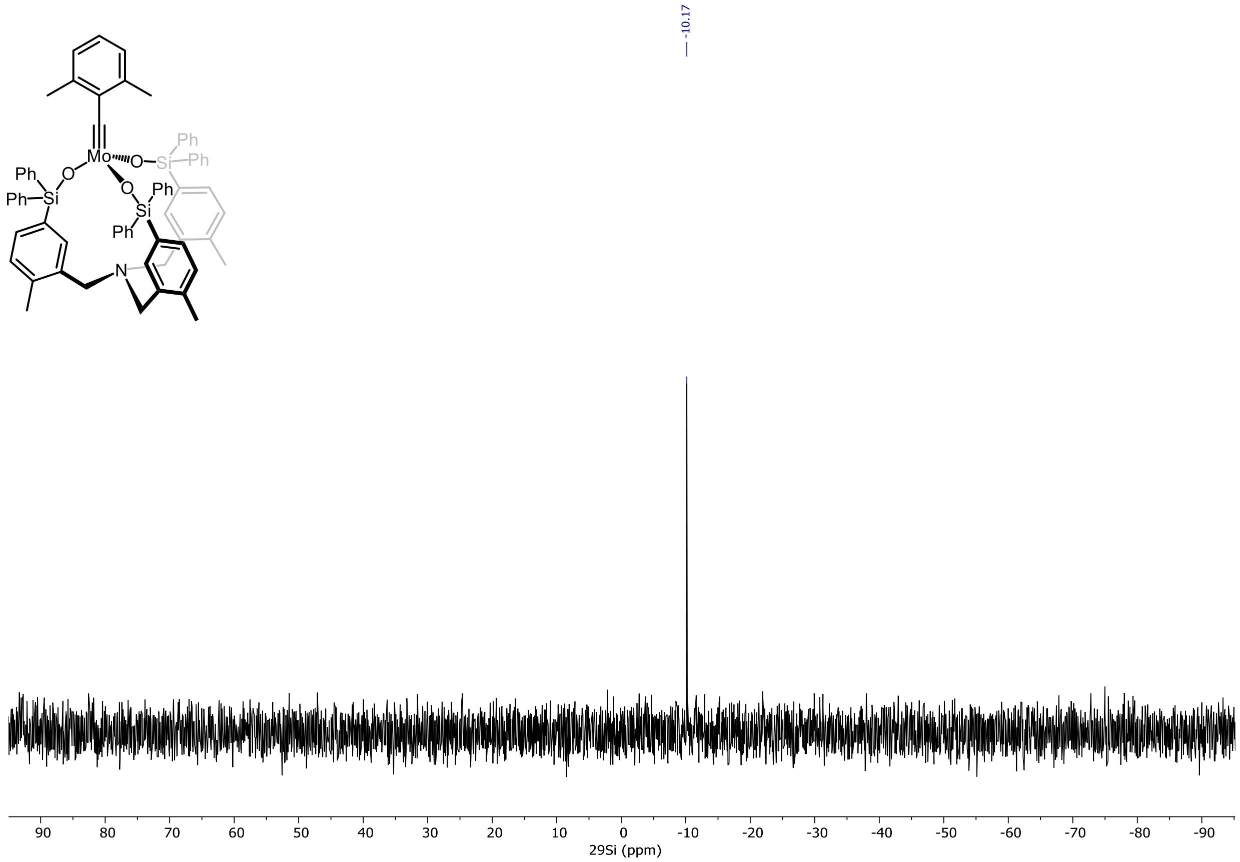

[95]Mo NMR of 15b, 26 MHz, [D8]-toluene, 60 °C

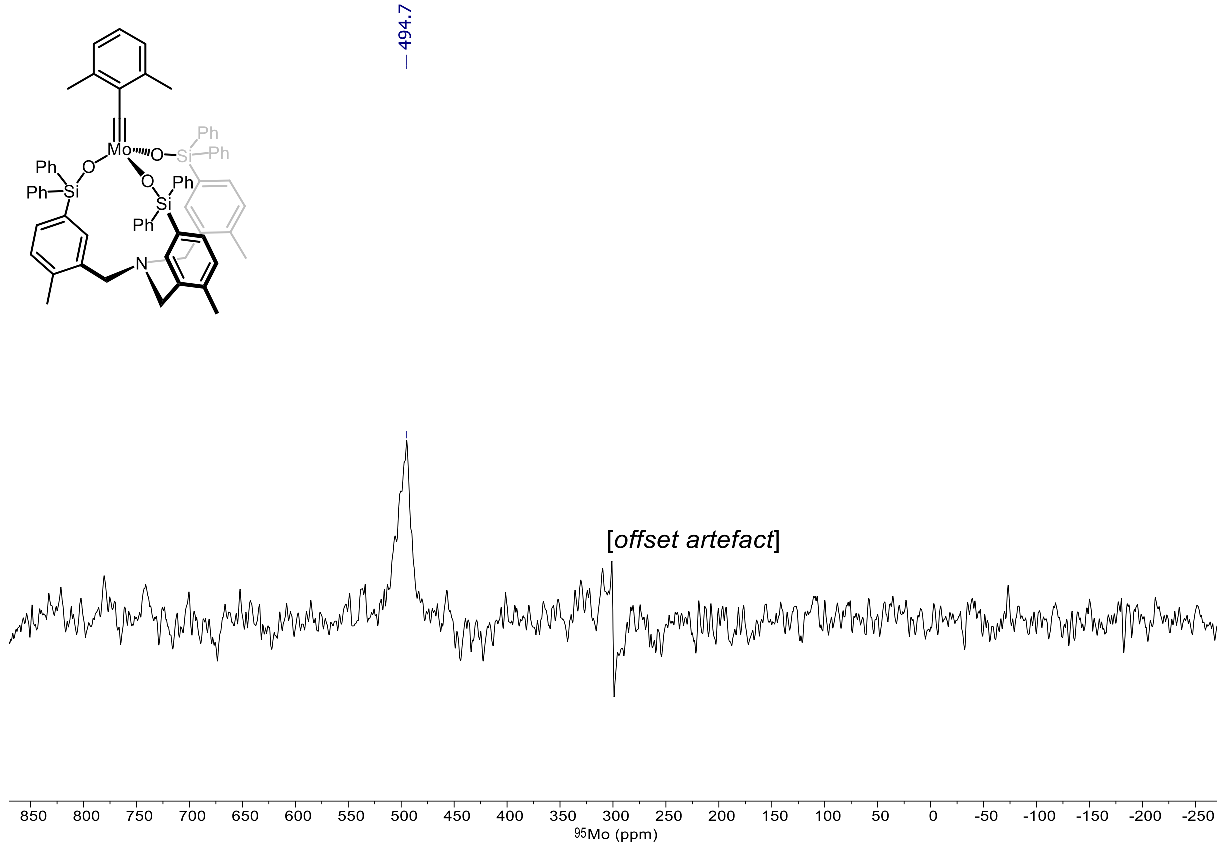

^{1}H -^{15}N HMBC of 15b, 61 MHz, [D8]-toluene, 25 °C

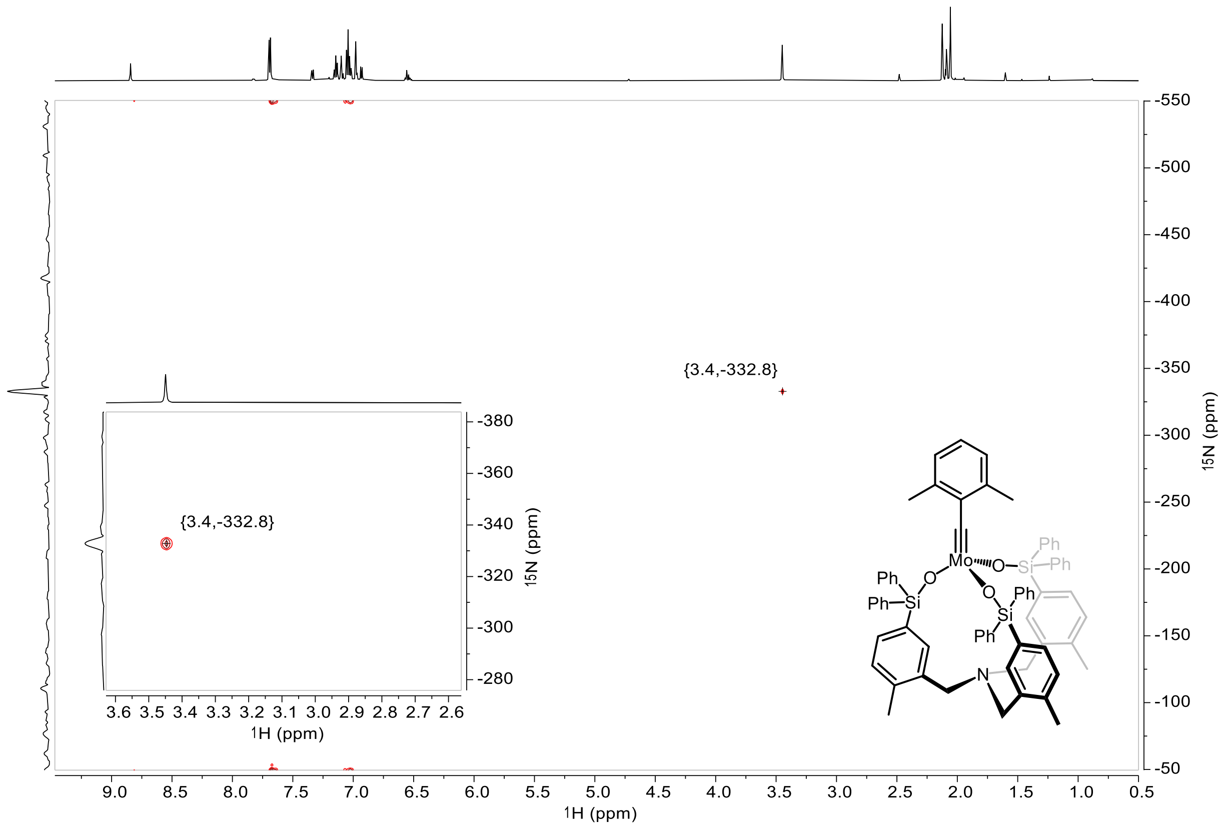

^{1}H NMR of 17a, 600 MHz, [D$_8$]-toluene, 25 °C

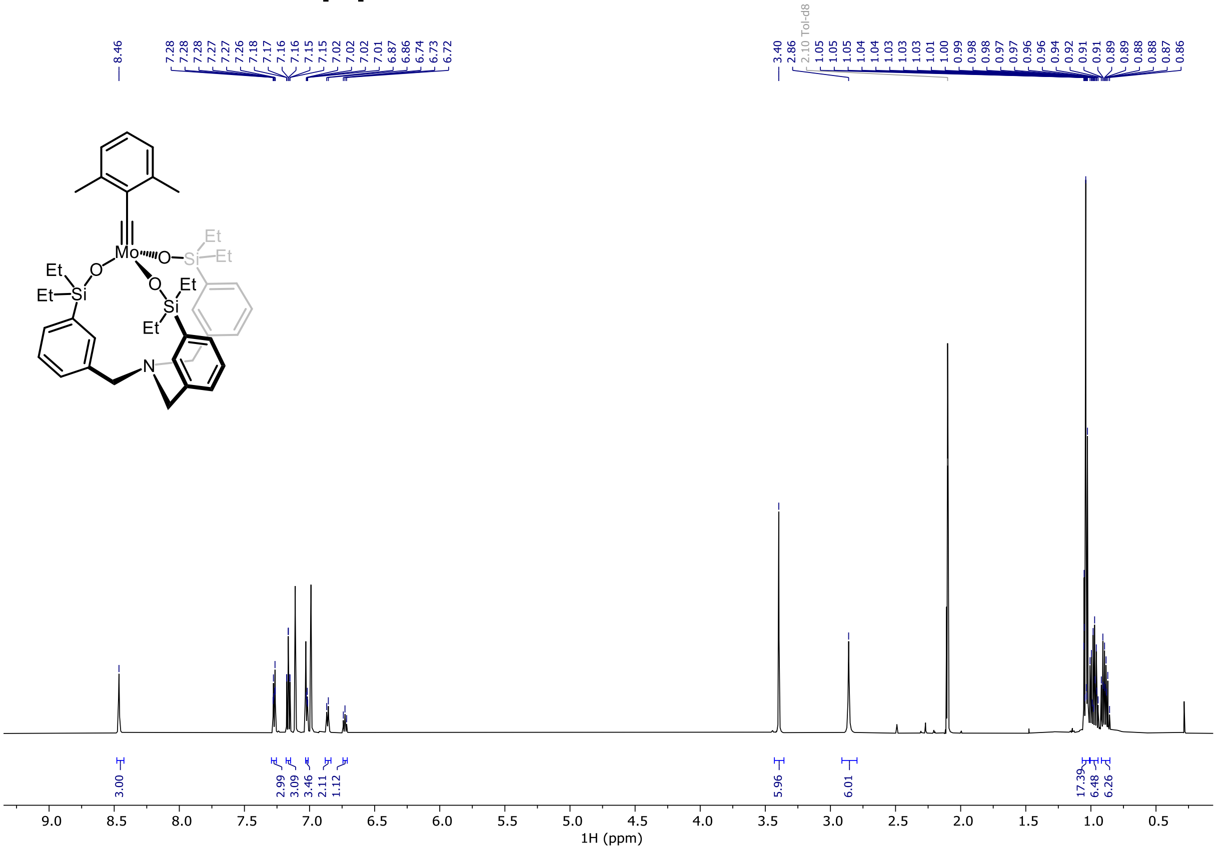

^{13}C NMR of 17a, 151 MHz, [D$_8$]-toluene, 25 °C

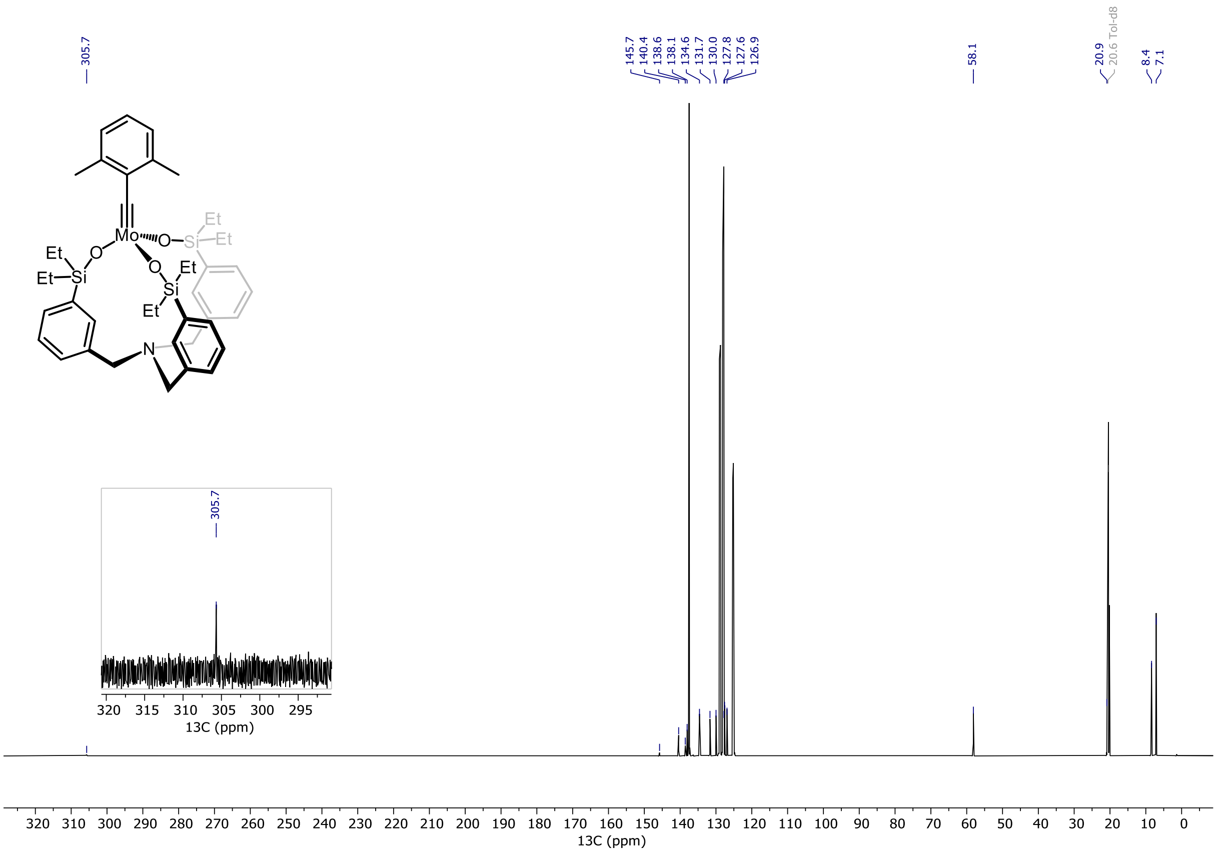

^{29}Si NMR of 17a, 119 MHz, [D$_8$]-toluene, 25 °C

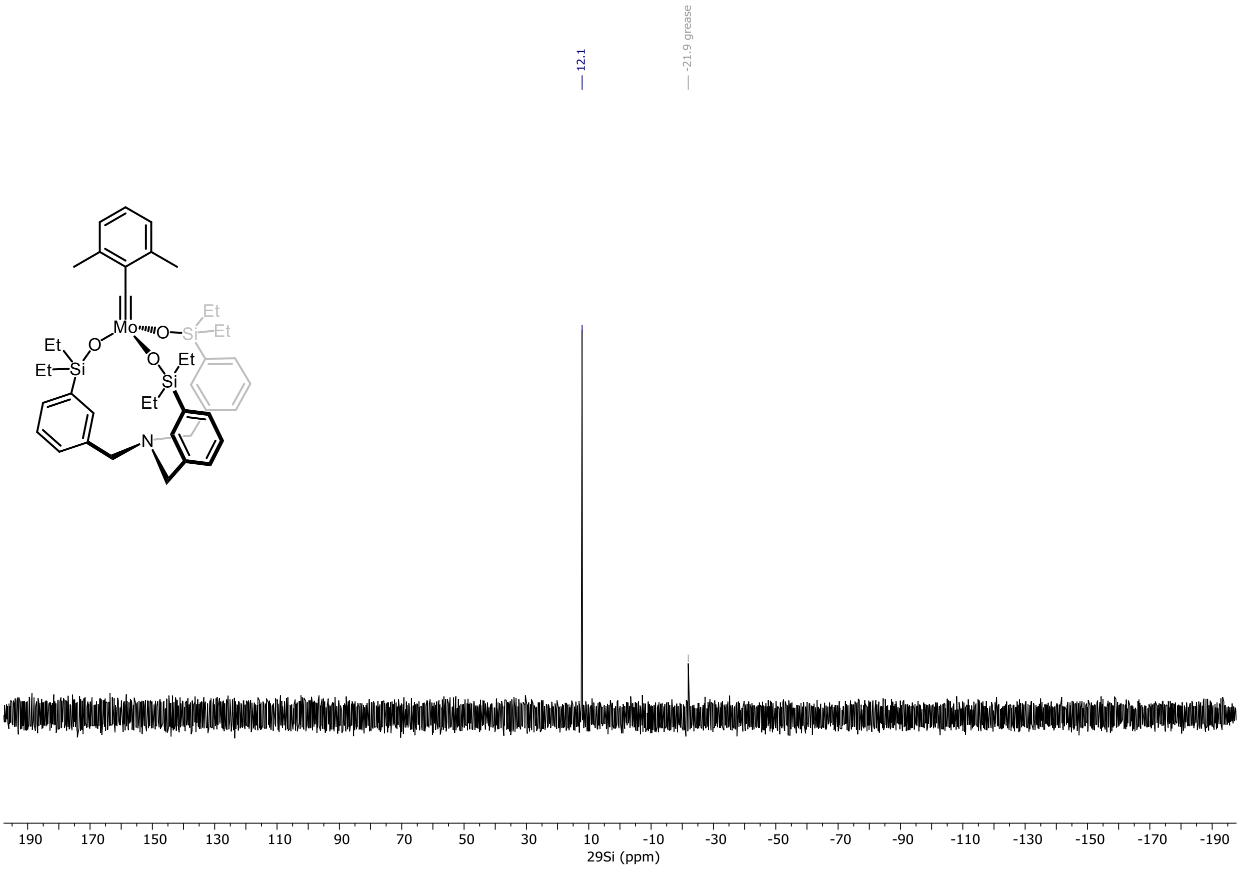

^{95}Mo NMR of 17a, 26 MHz, [D$_8$]-toluene, 60 °C

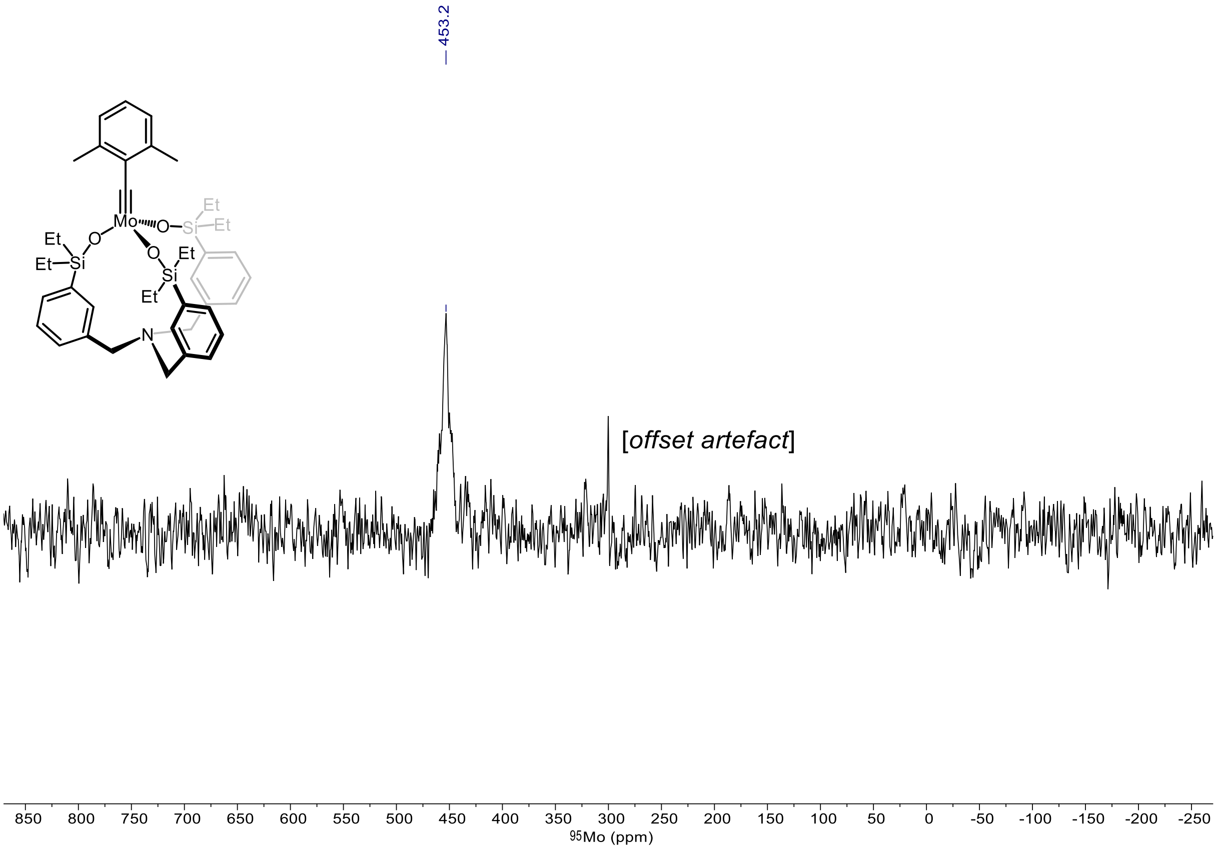

^{1}H NMR of 17b, 600 MHz, [D$_8$]-toluene, 25 °C

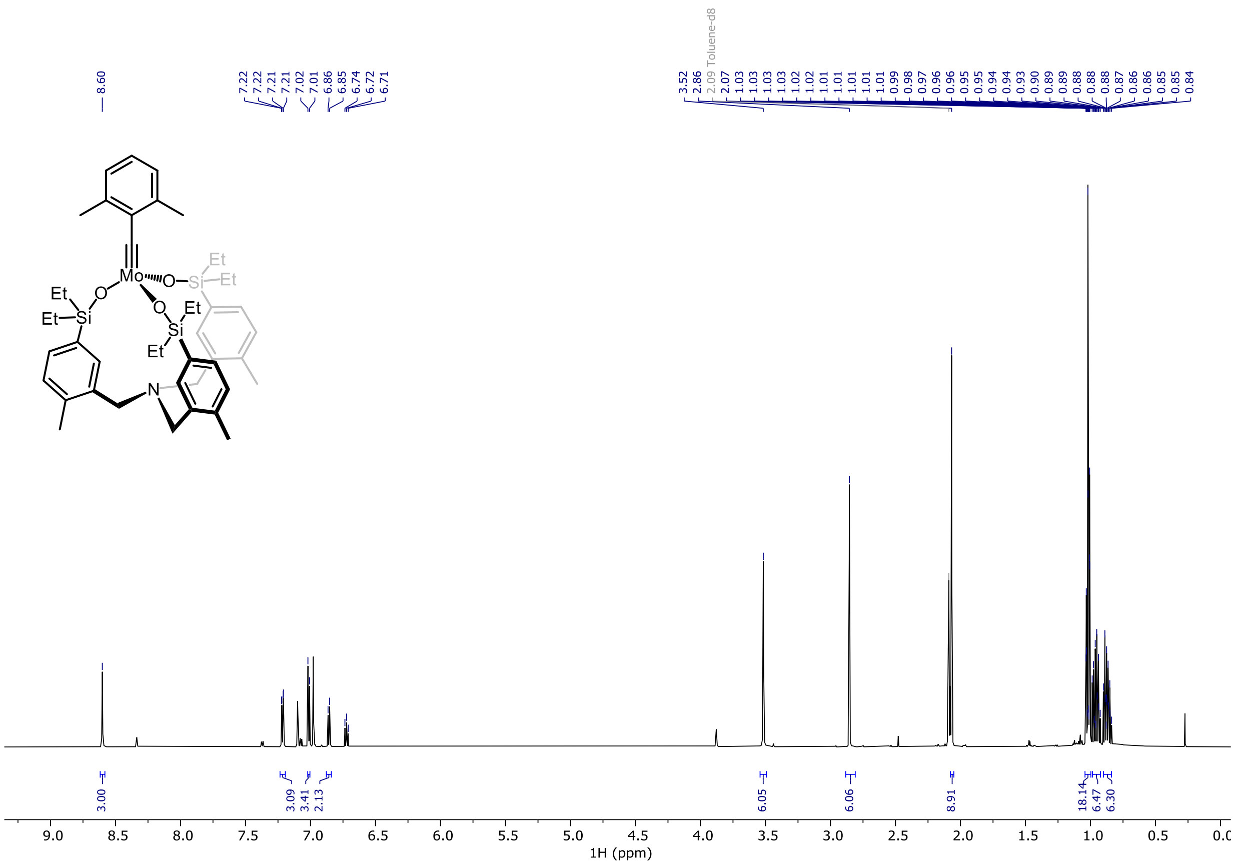

^{13}C NMR of 17b, 151 MHz, [D$_8$]-toluene, 25 °C

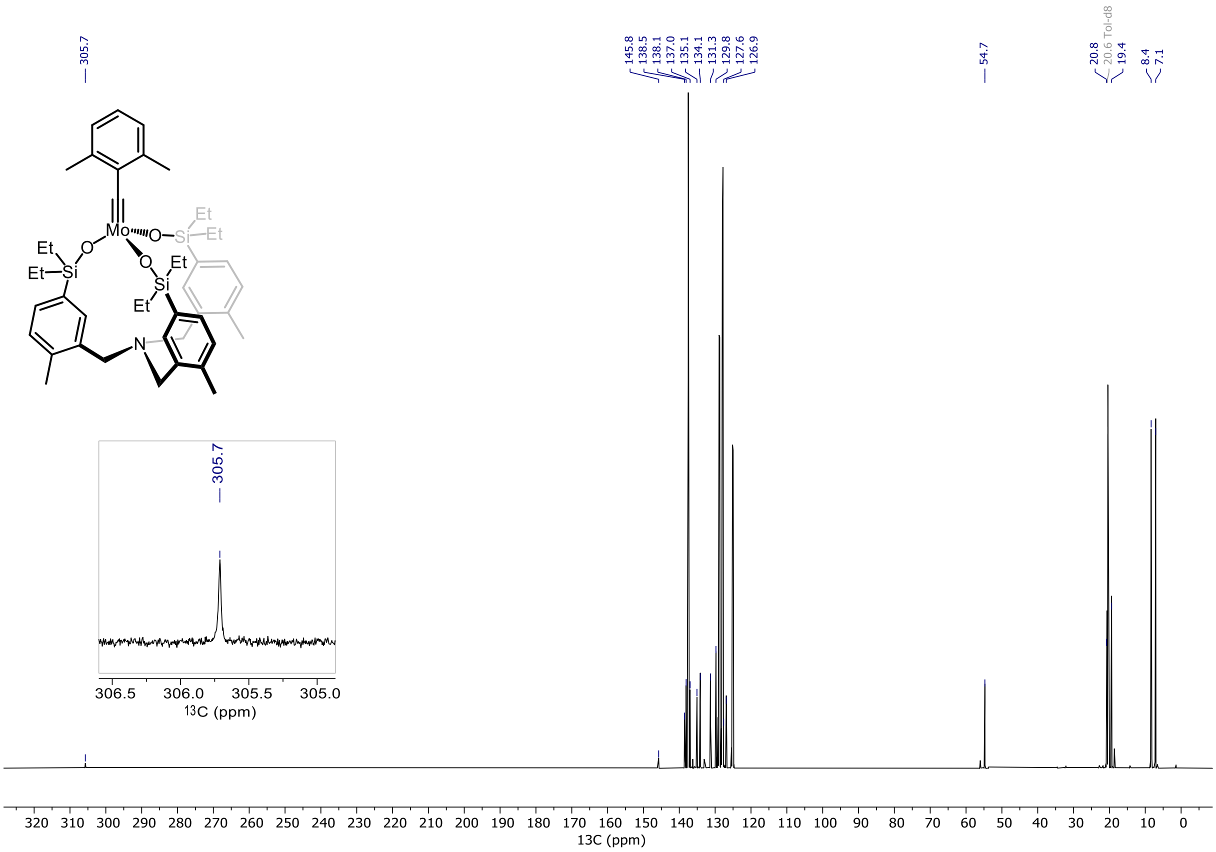

^{29}Si NMR of 17b, 119 MHz, [D8]-toluene, 25 °C

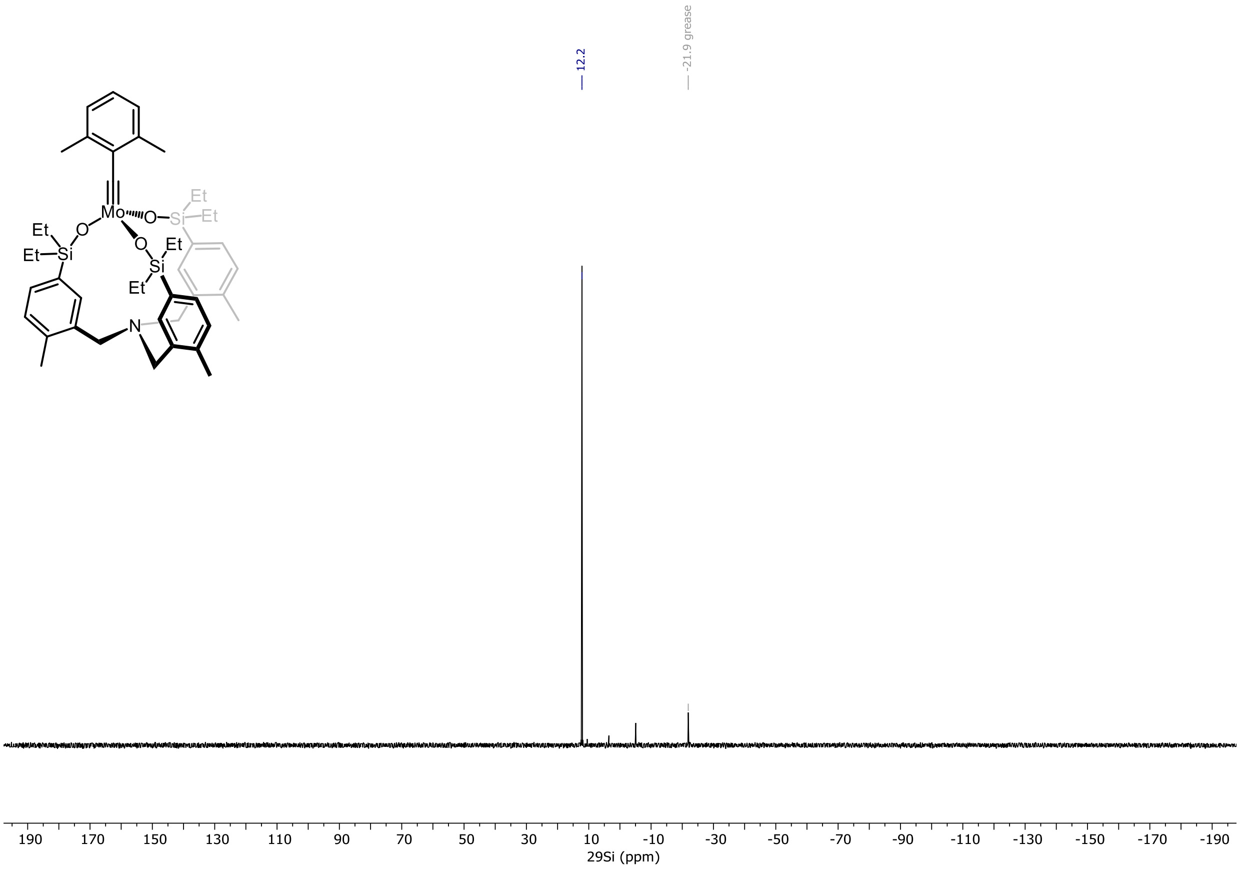

^{95}Mo NMR of 17b, 26 MHz, [D8]-toluene, 60 °C

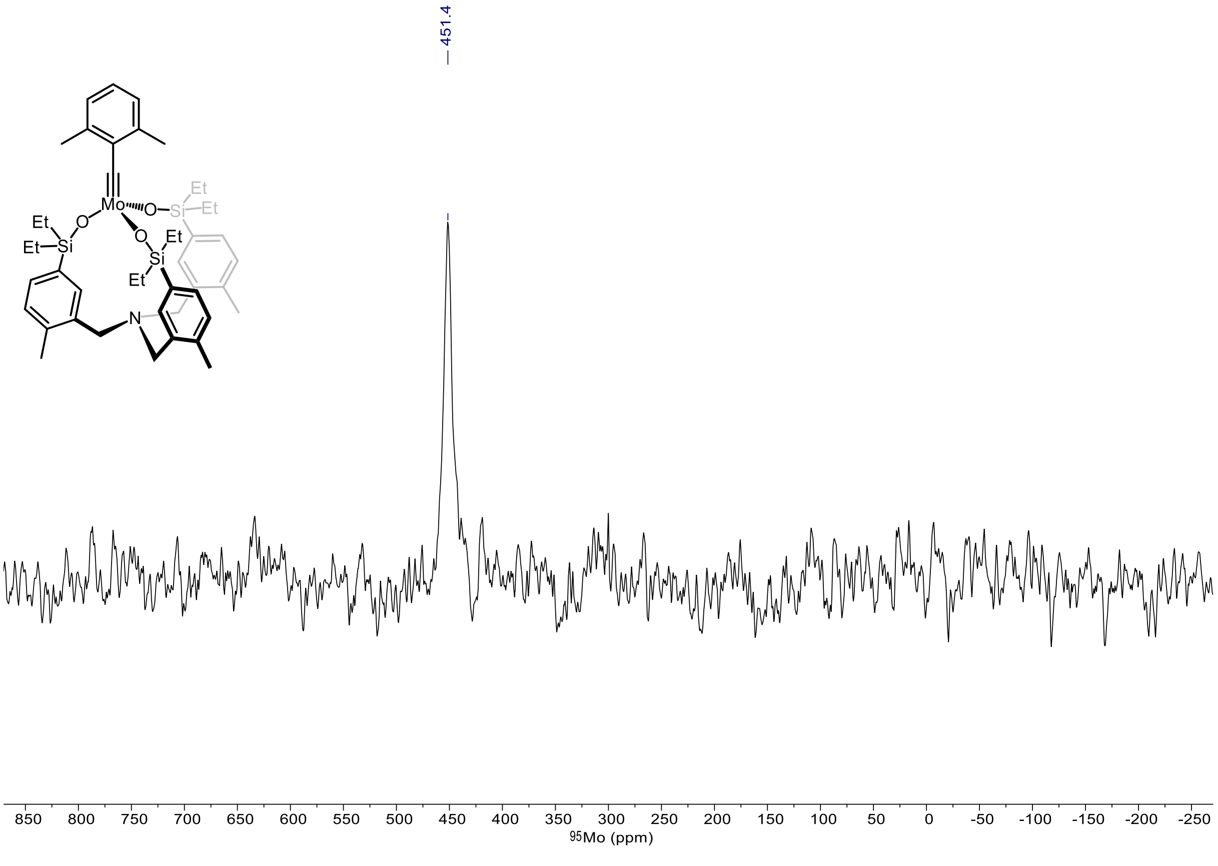

Adducts

^{1}H NMR of 16a, 600 MHz, [D$_8$]-toluene, 253 K

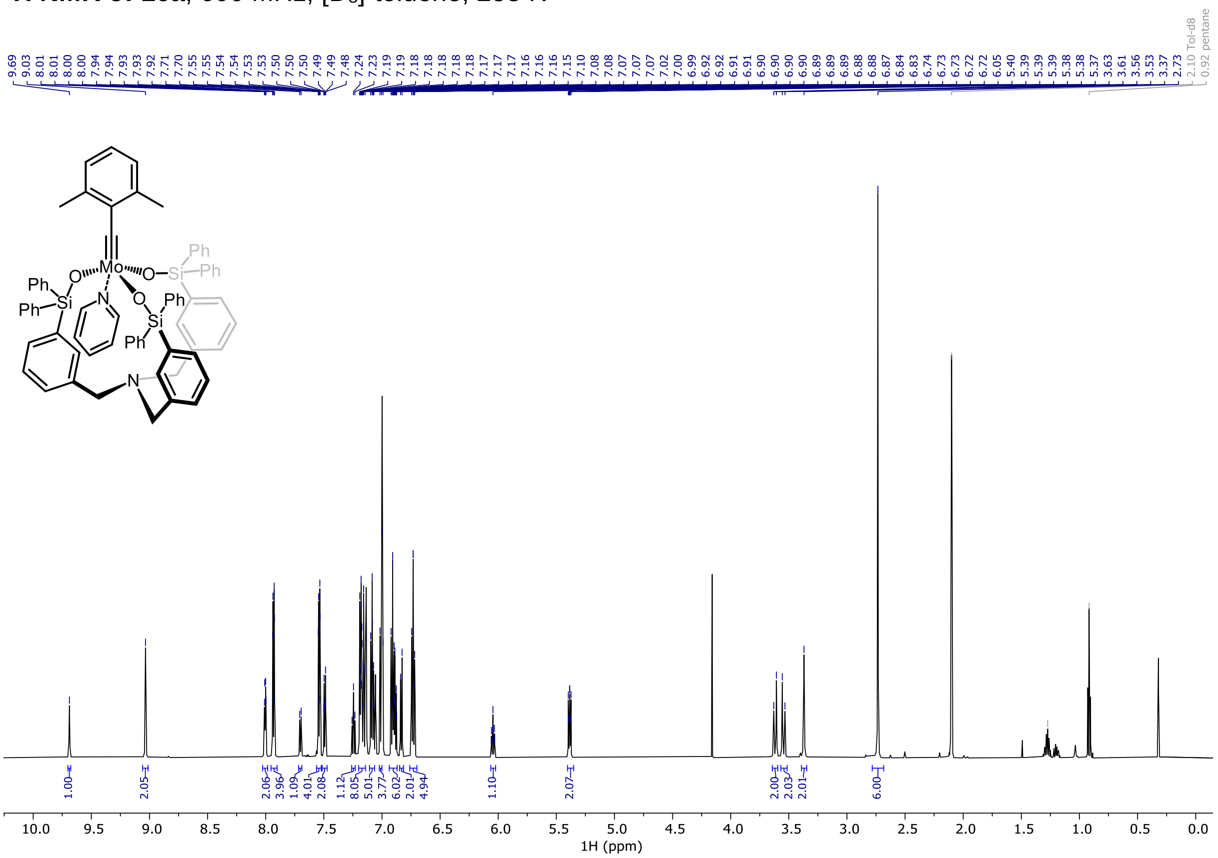

^{13}C NMR of 16a, 151 MHz, [D$_8$]-toluene, 253 K

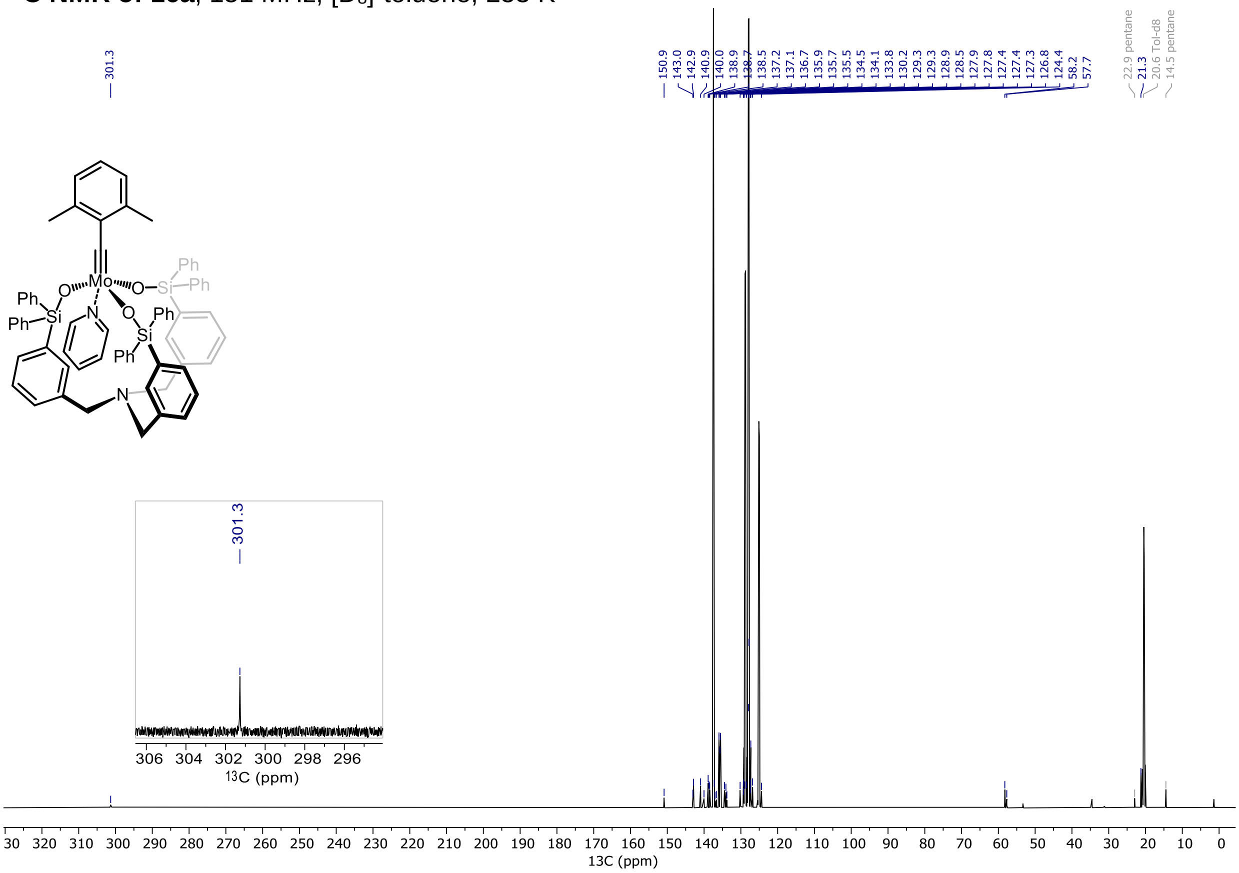

^{29}Si NMR of 16a, 119 MHz, [D8]-toluene, 253 K

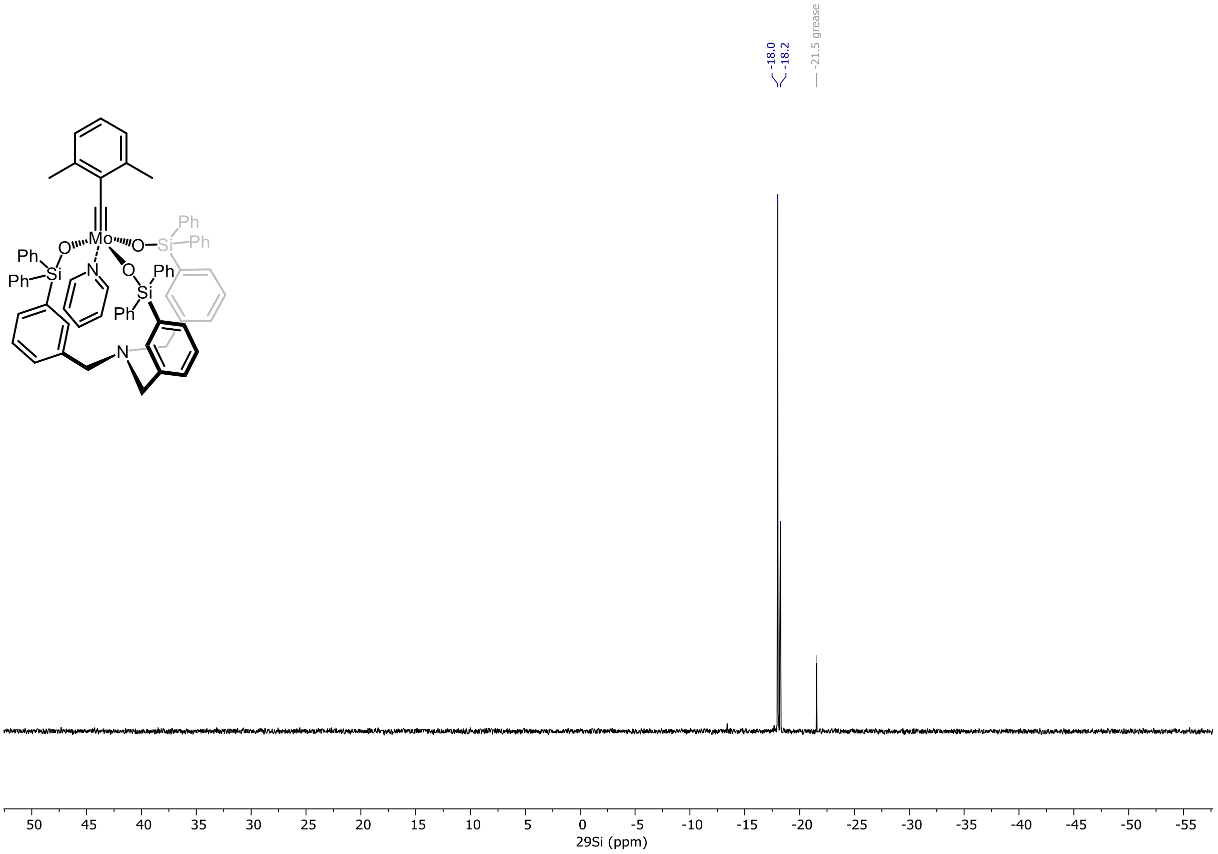

^{1}H -^{15}N HMBC of 16a, 61 MHz, [D8]-toluene, 253 °K

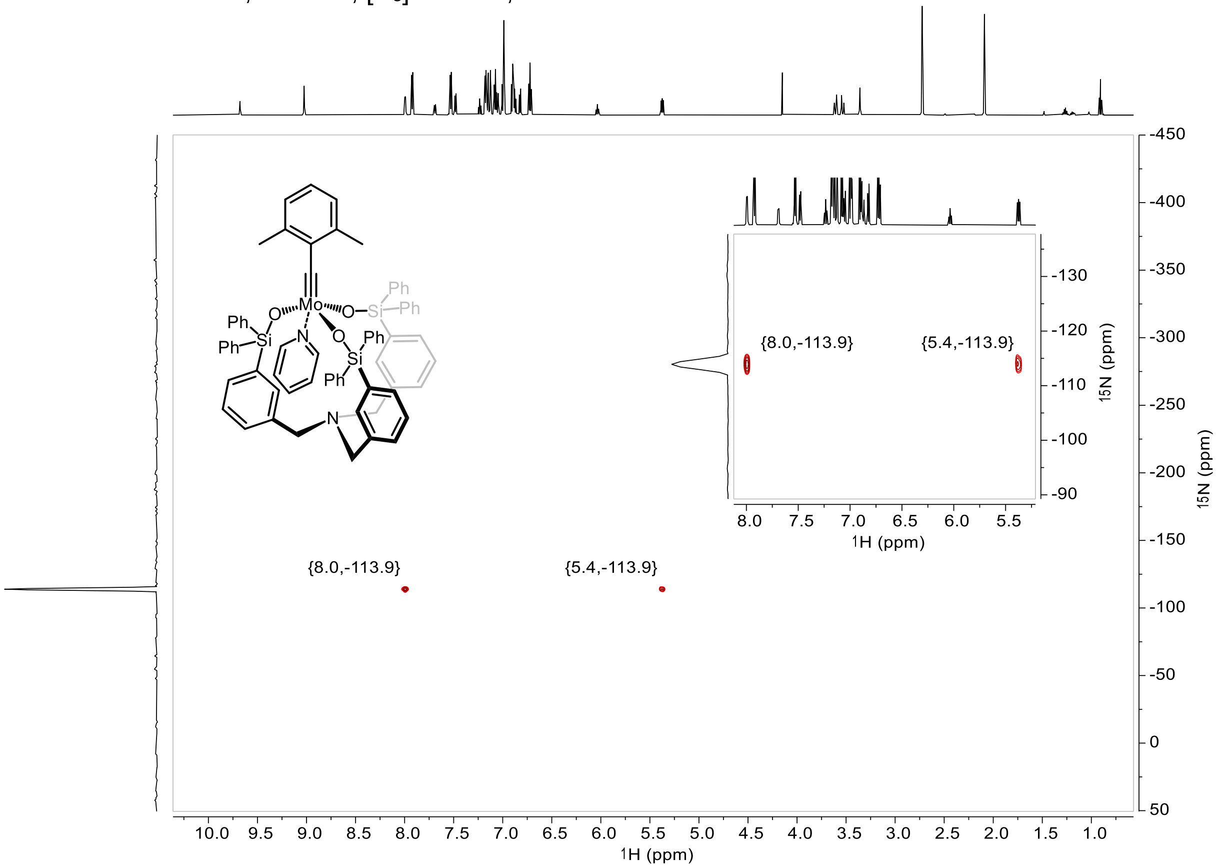

^{1}H NMR of 16b, 600 MHz, [D$_8$]-toluene/CD_2Cl_2, 253 K

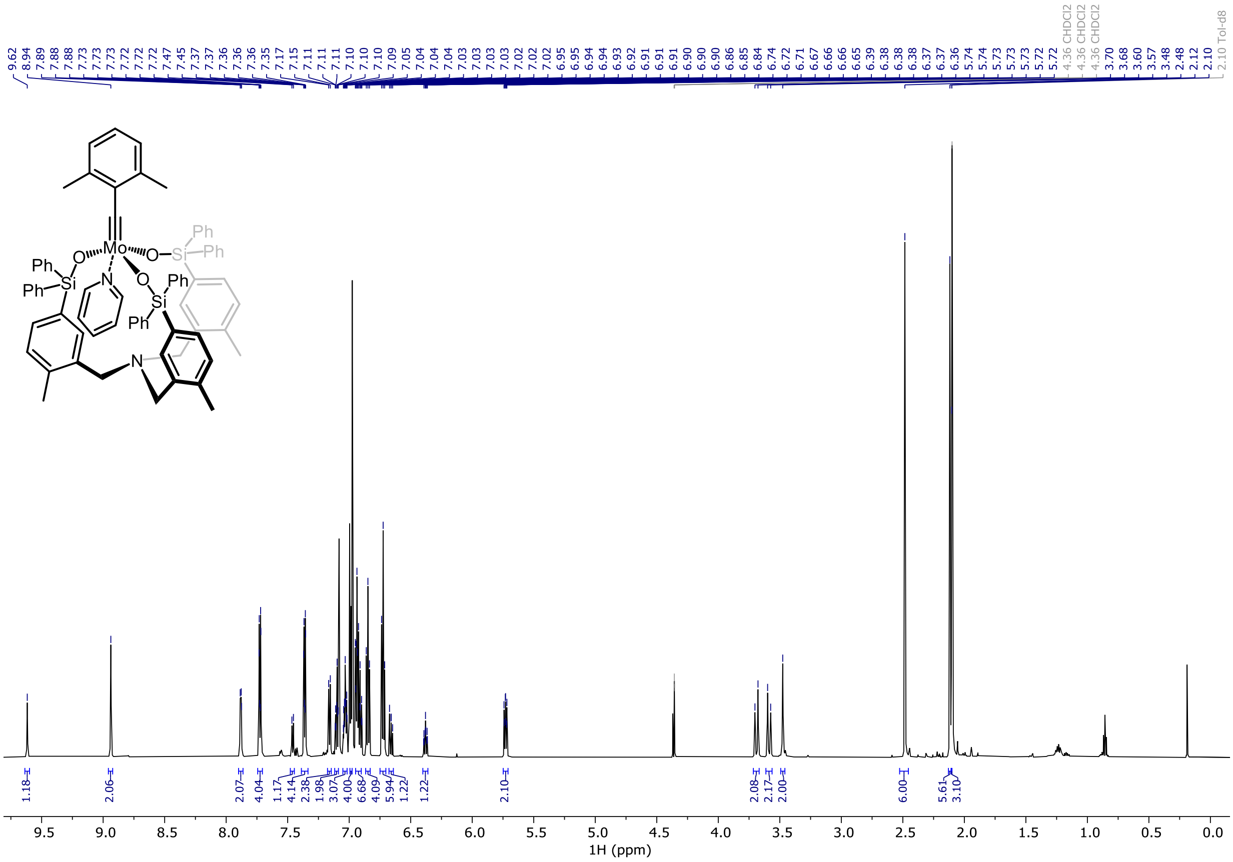

^{13}C NMR of 16b, 151 MHz, [D$_8$]-toluene/CD_2Cl_2, 253 K

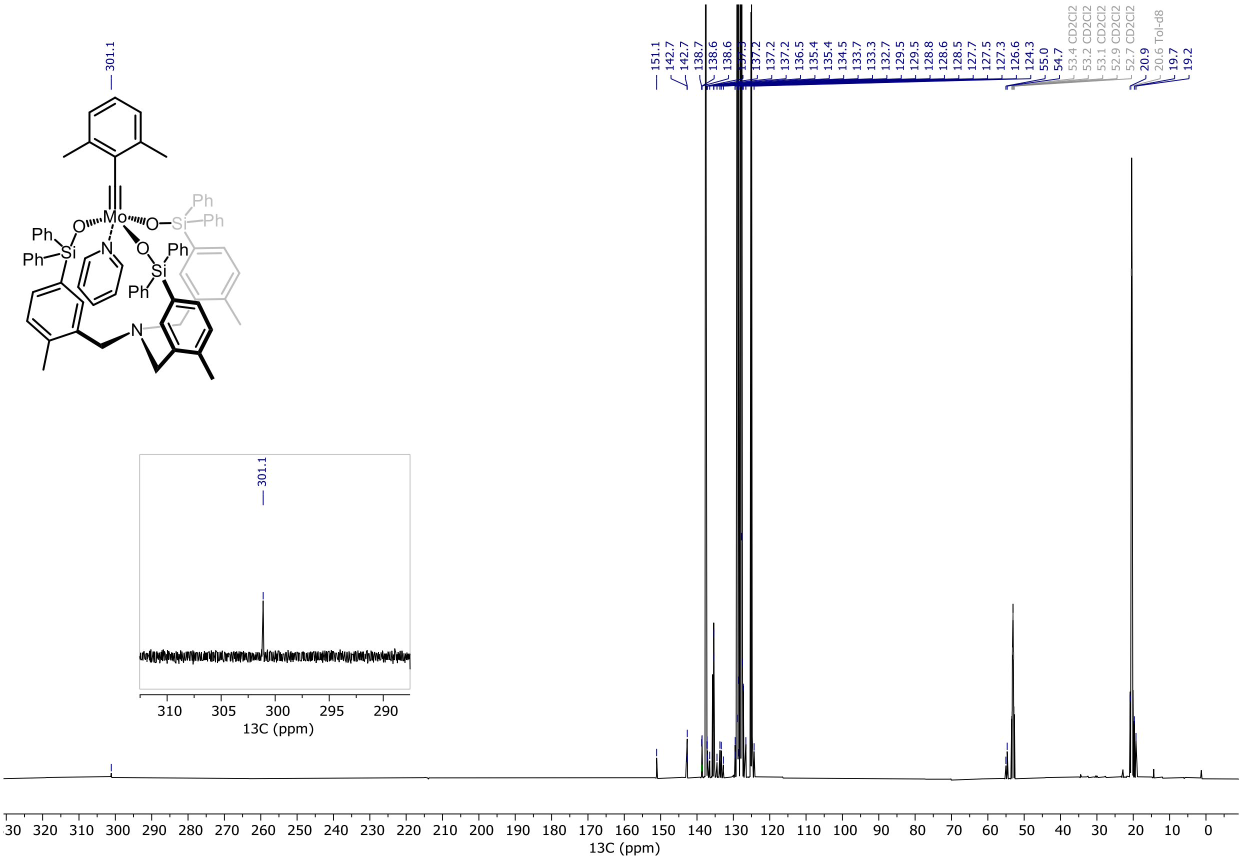

^{29}Si NMR of 16b, 119 MHz, [D8]-toluene/CD_2Cl_2, 253 K

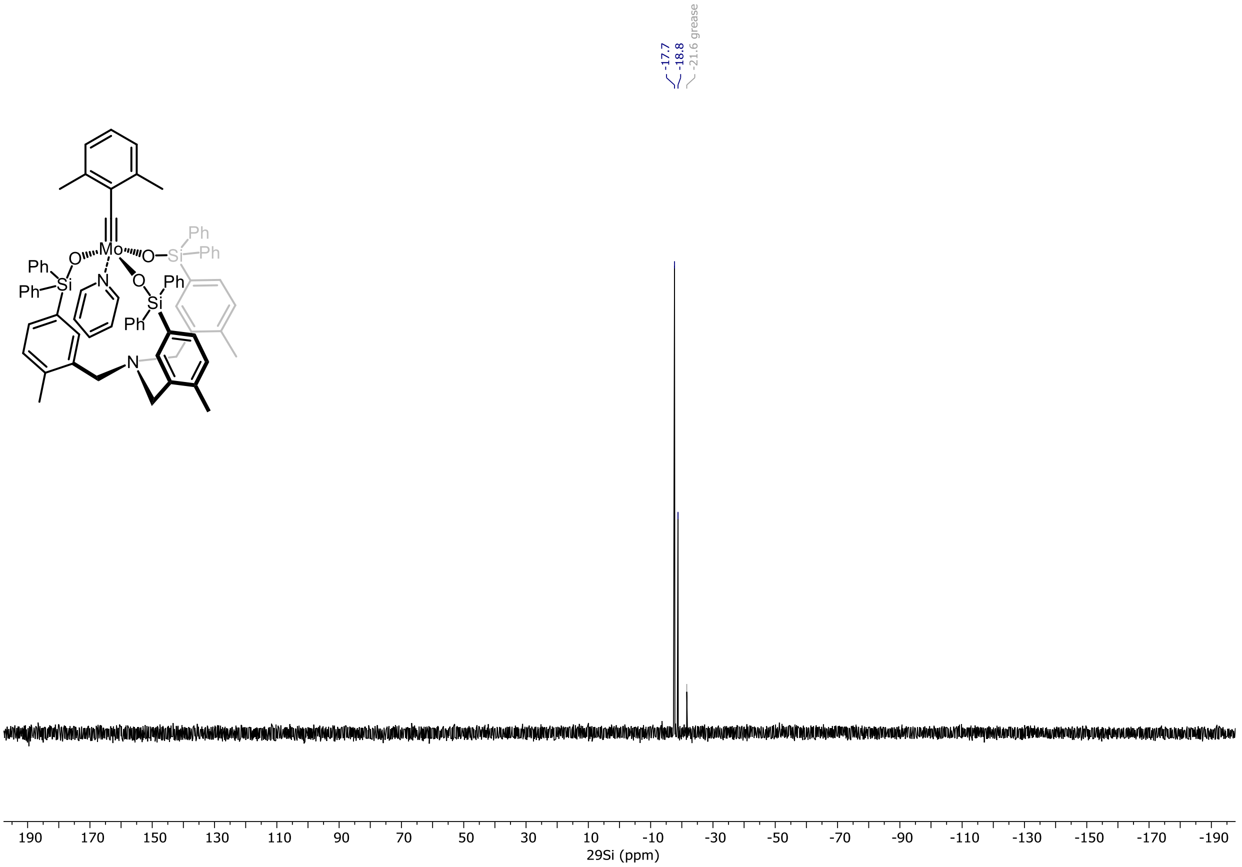

^{1}H -^{15}N HMBC of 16b, 61 MHz, [D8]-toluene/CD_2Cl_2, 253 °K

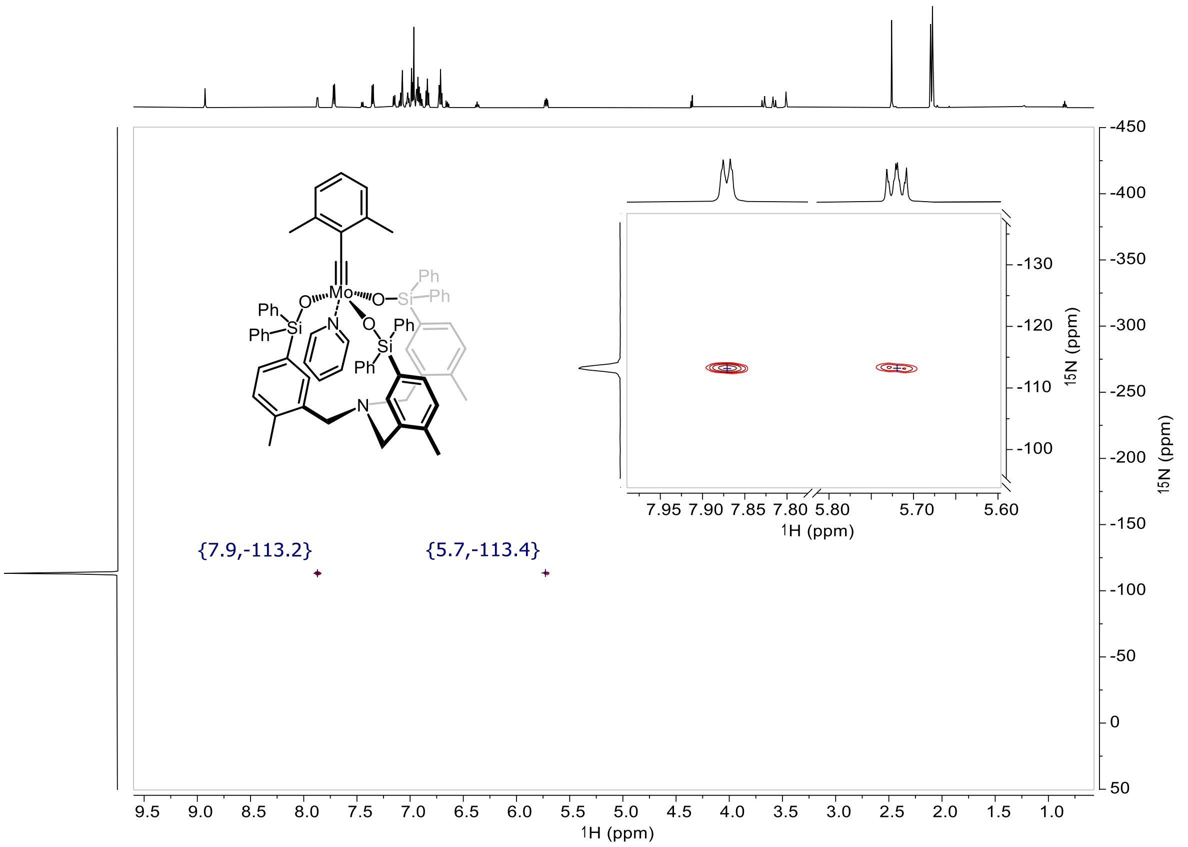

^{1}H NMR of 16c, 600 MHz, [D$_8$]-toluene, 233 K

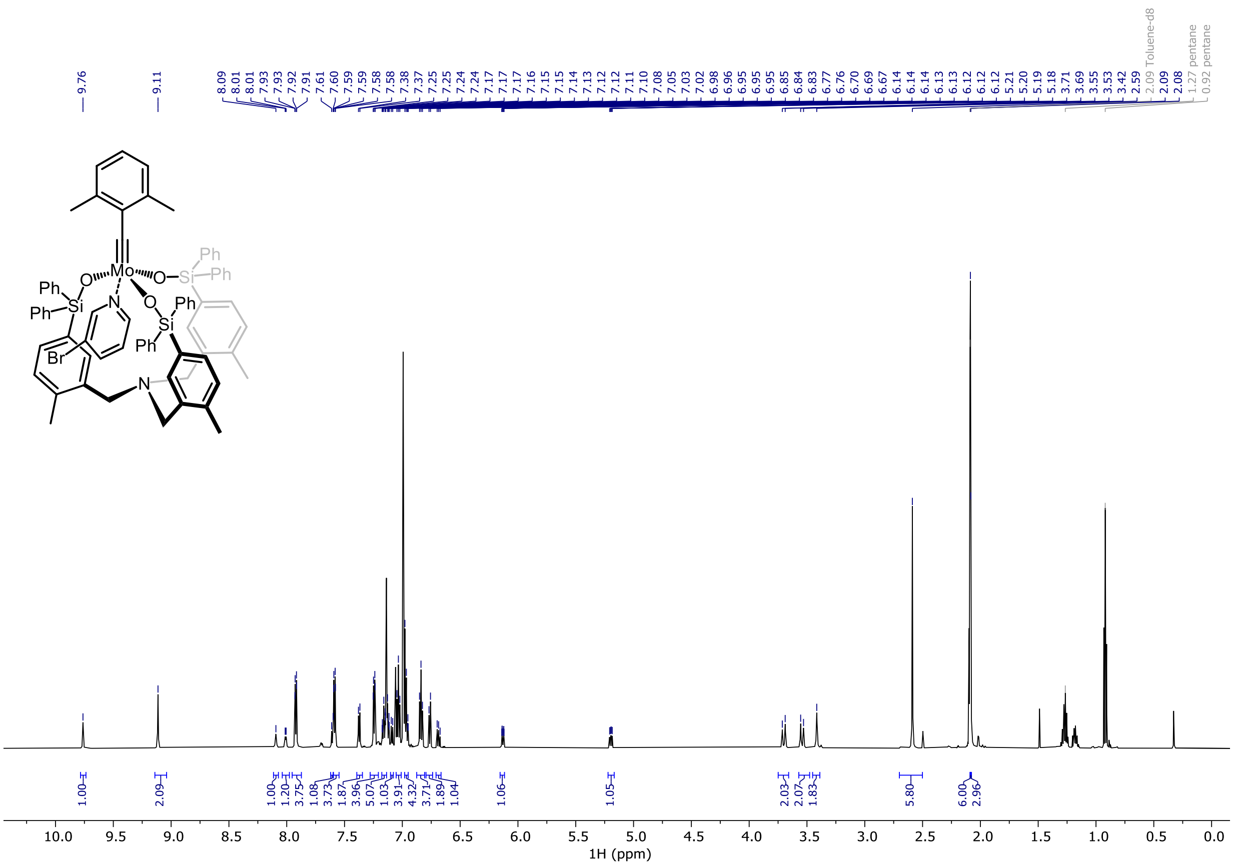

^{13}C NMR of 16c, 151 MHz, [D$_8$]-toluene, 233 K

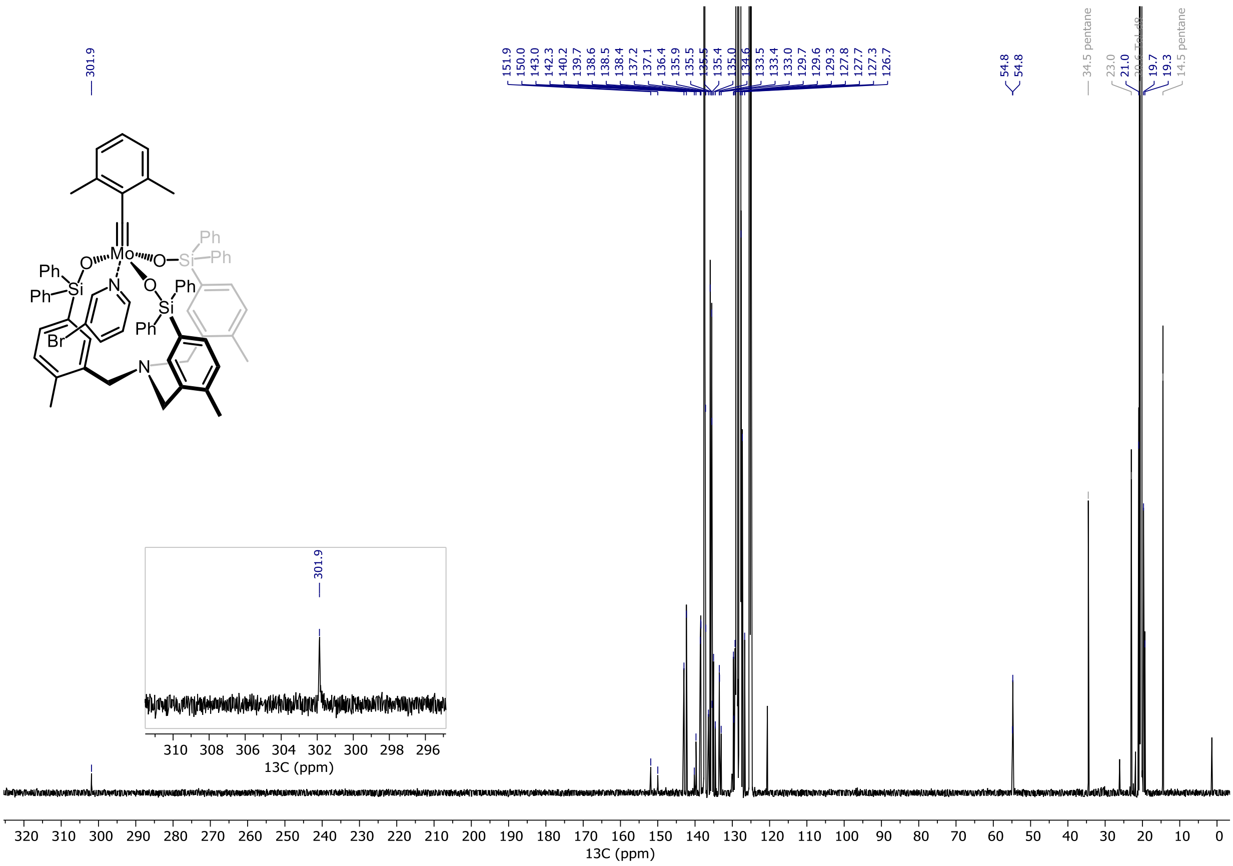

^{29}Si NMR of 16c, 119 MHz, [D8]-toluene, 233 K

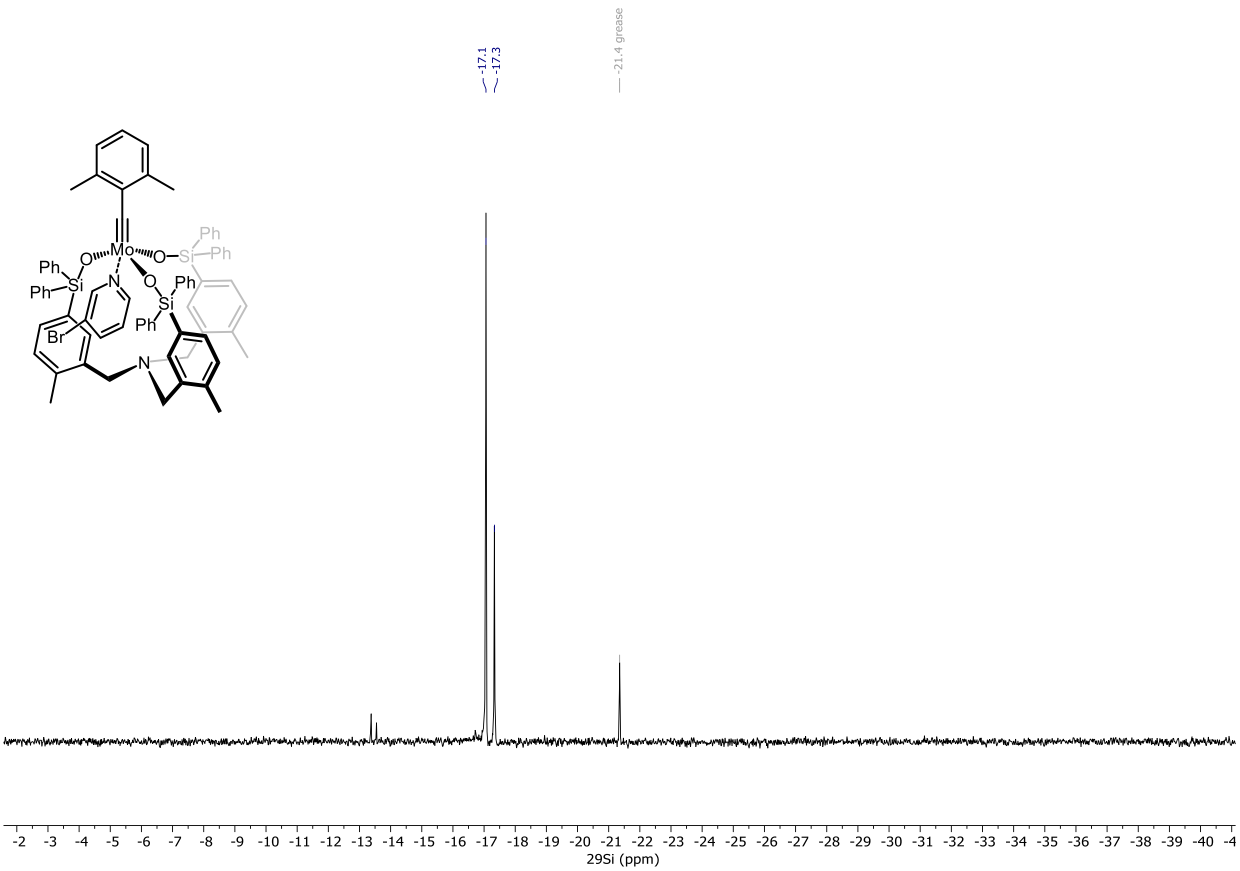

^{1}H -^{15}N HMBC of 16c, 61 MHz, [D8]-toluene, 233 °K

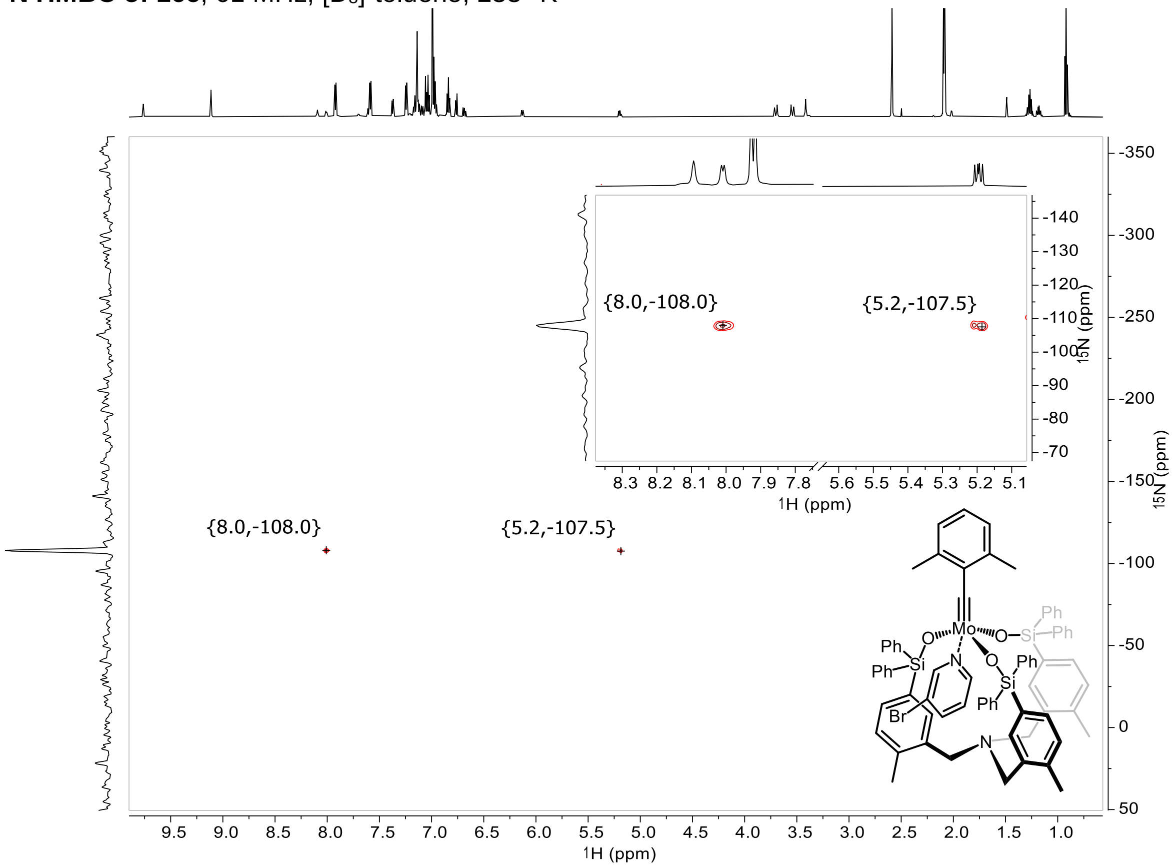

^{1}H NMR of 16d, 600 MHz, [D$_8$]-toluene, 228 K

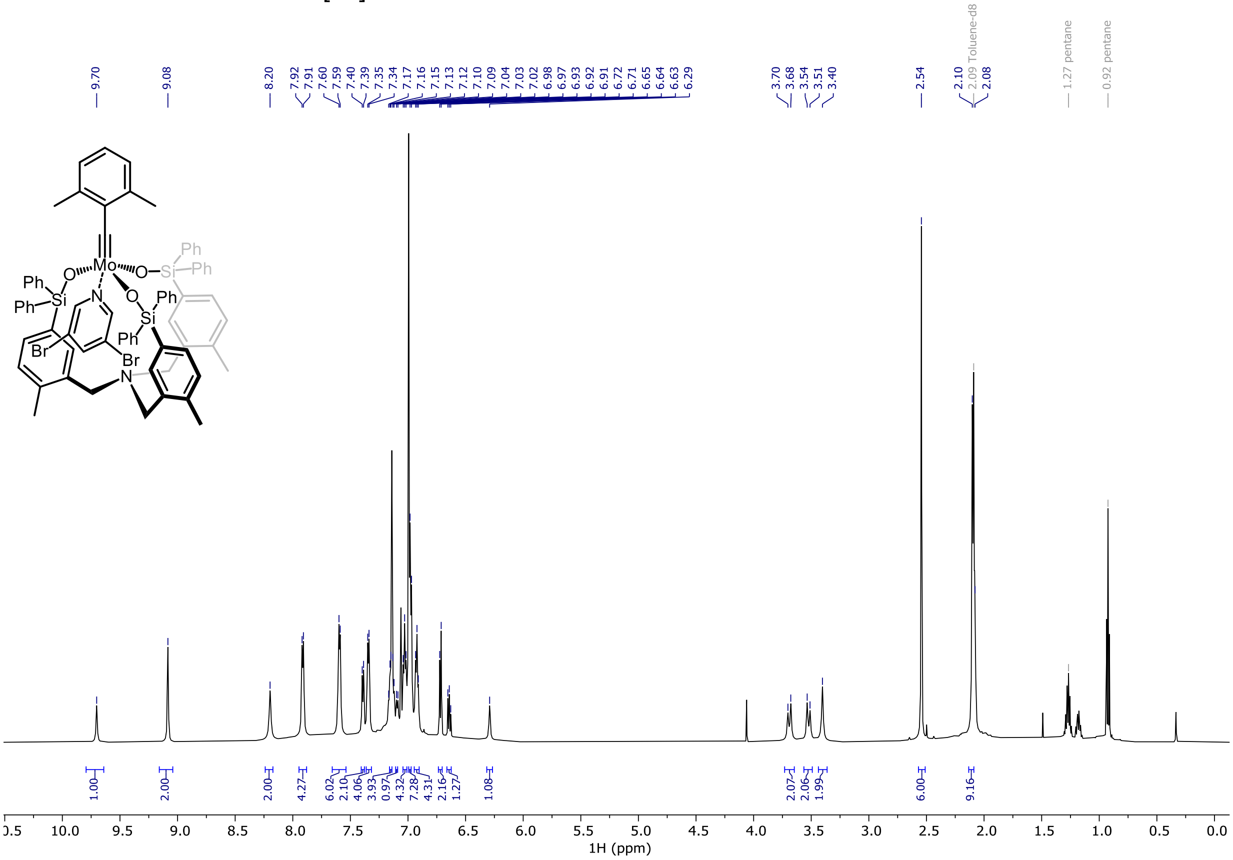

^{13}C NMR of 16d, 151 MHz, [D$_8$]-toluene, 228 K

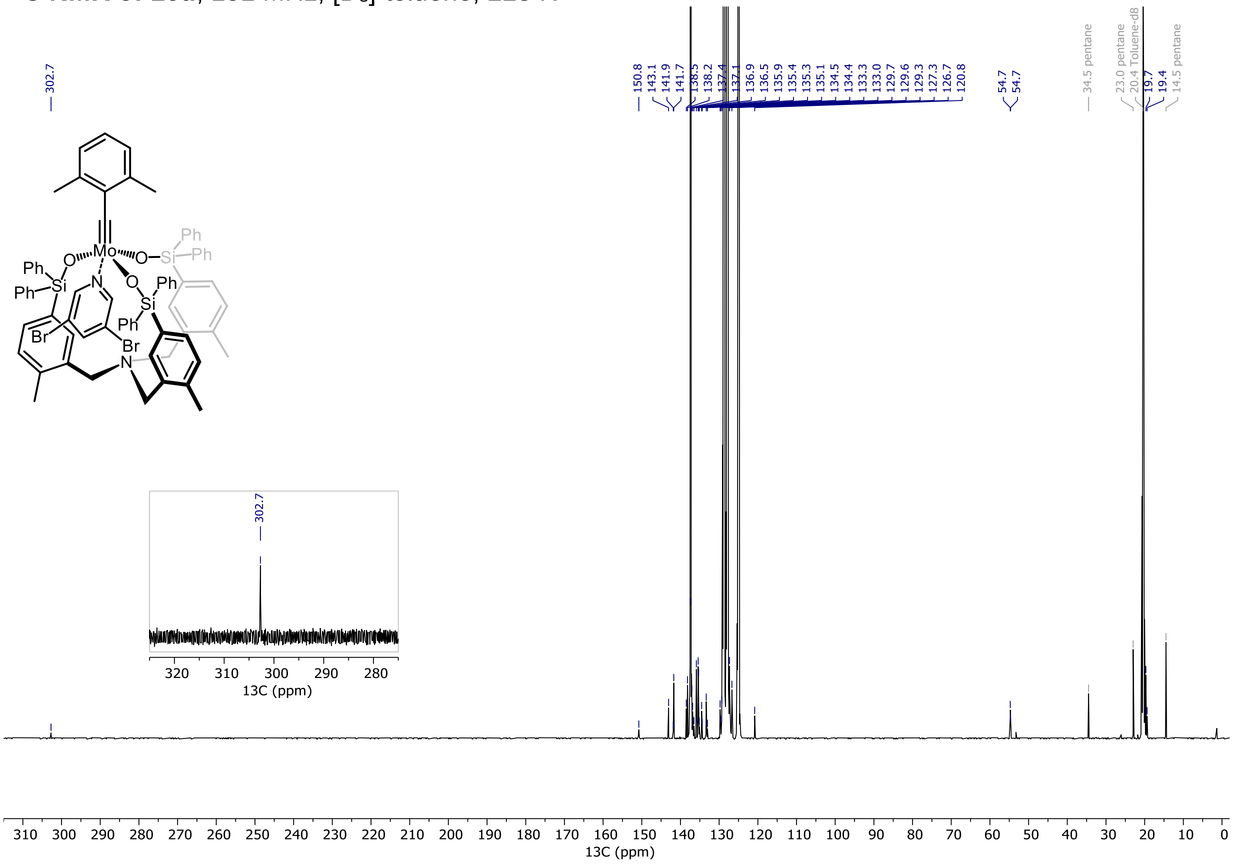

^{29}Si NMR of 16d, 119 MHz, [D8]-toluene, 228 K

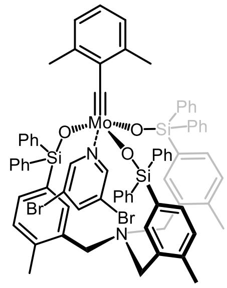

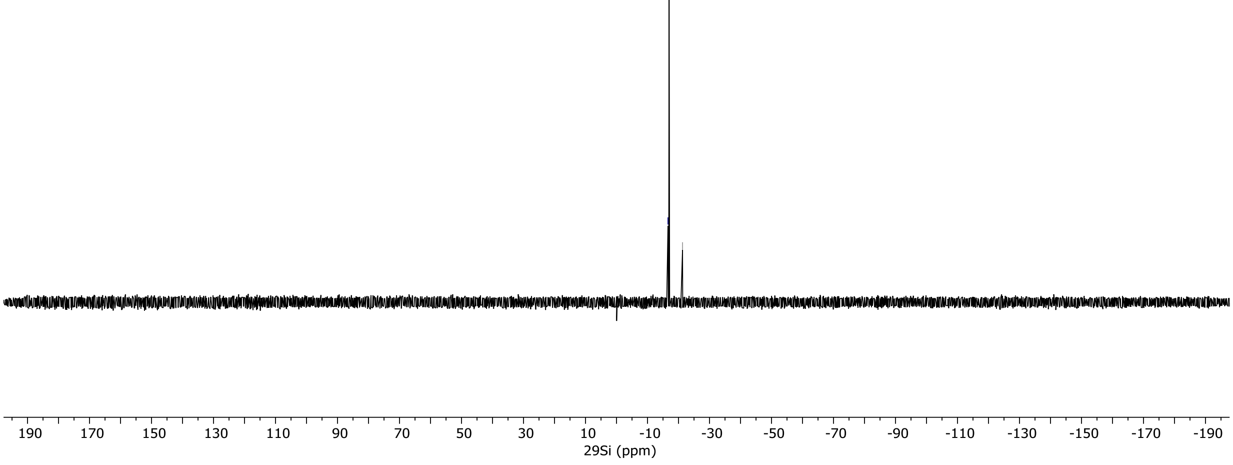

^{1}H NMR of 16e (ca. 93% purity), 600 MHz, [D$_8$]-toluene, 273 K

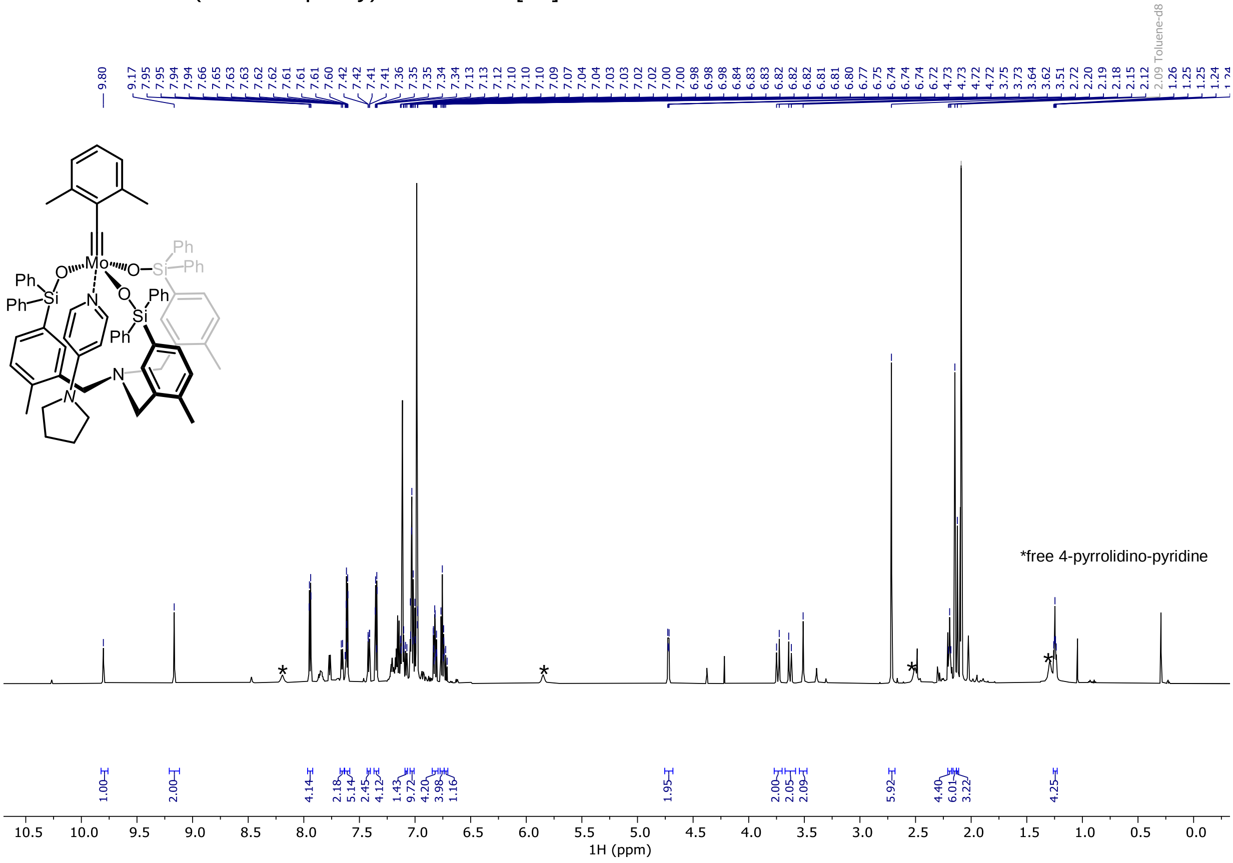

^{13}C NMR of 16e (ca. 93% purity), 151 MHz, [D$_8$]-toluene, 273 K

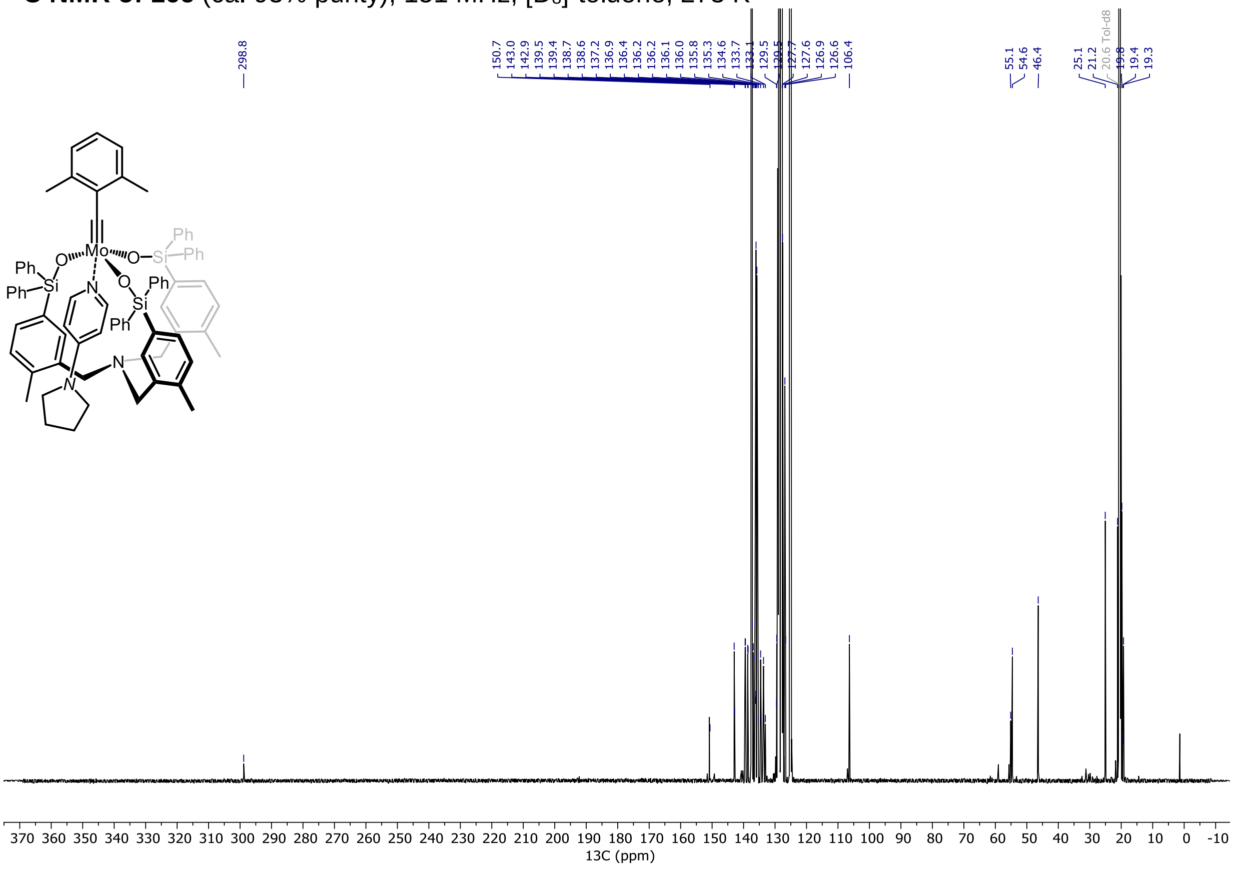

^{29}Si NMR of 16e (ca. 93% purity), 119 MHz, [D$_8$]-toluene, 273 K

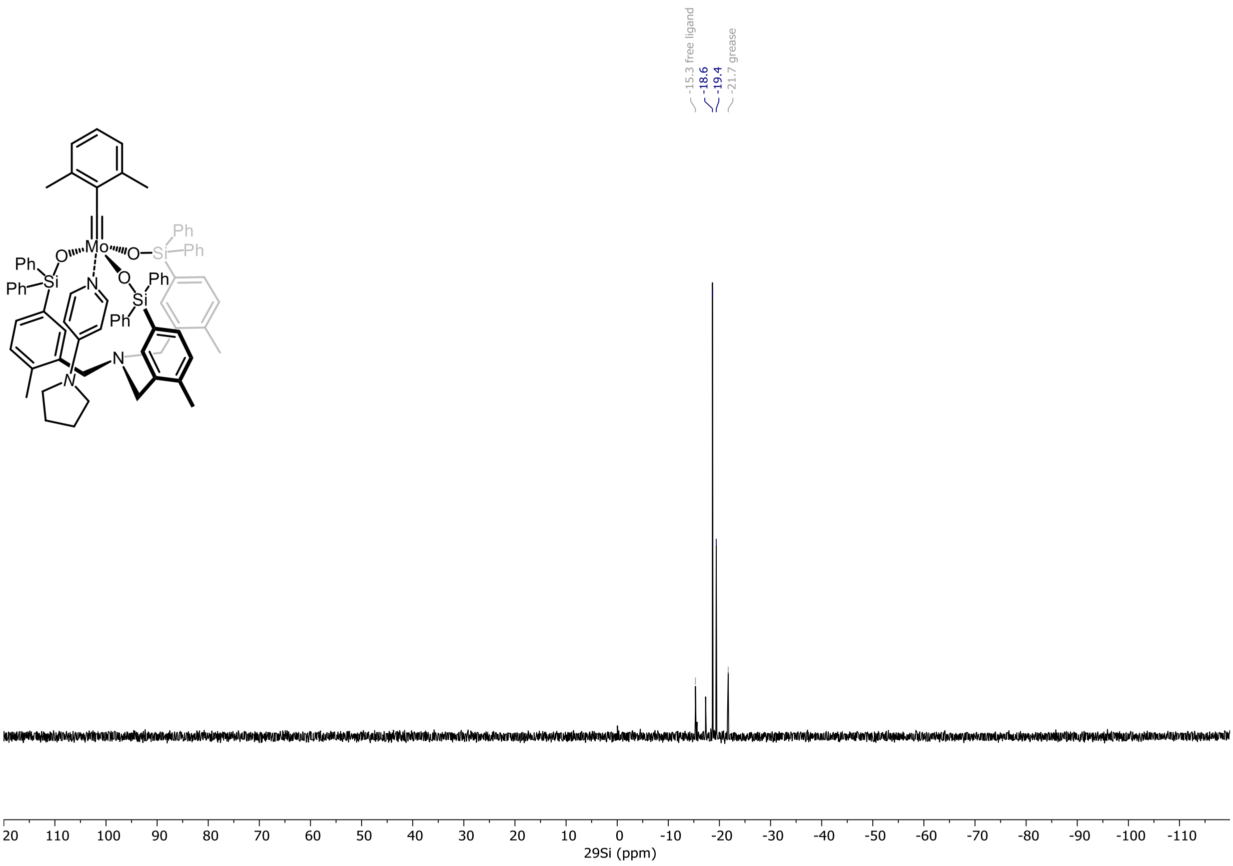

^{1}H -^{15}N HMBC of 16e, 61 MHz, [D$_8$]-toluene, 273 °K

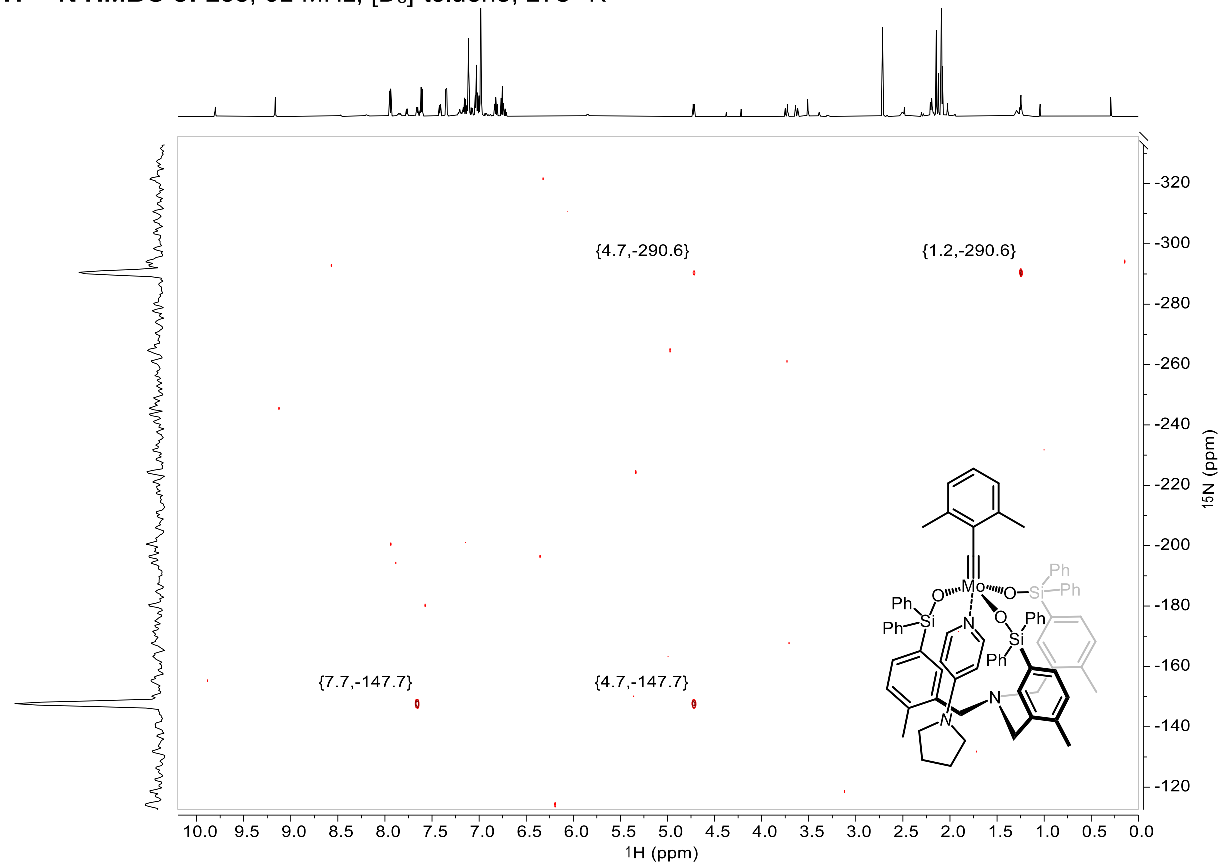

^{1}H NMR of 16f, 600 MHz, [D_8]-toluene, 233 K

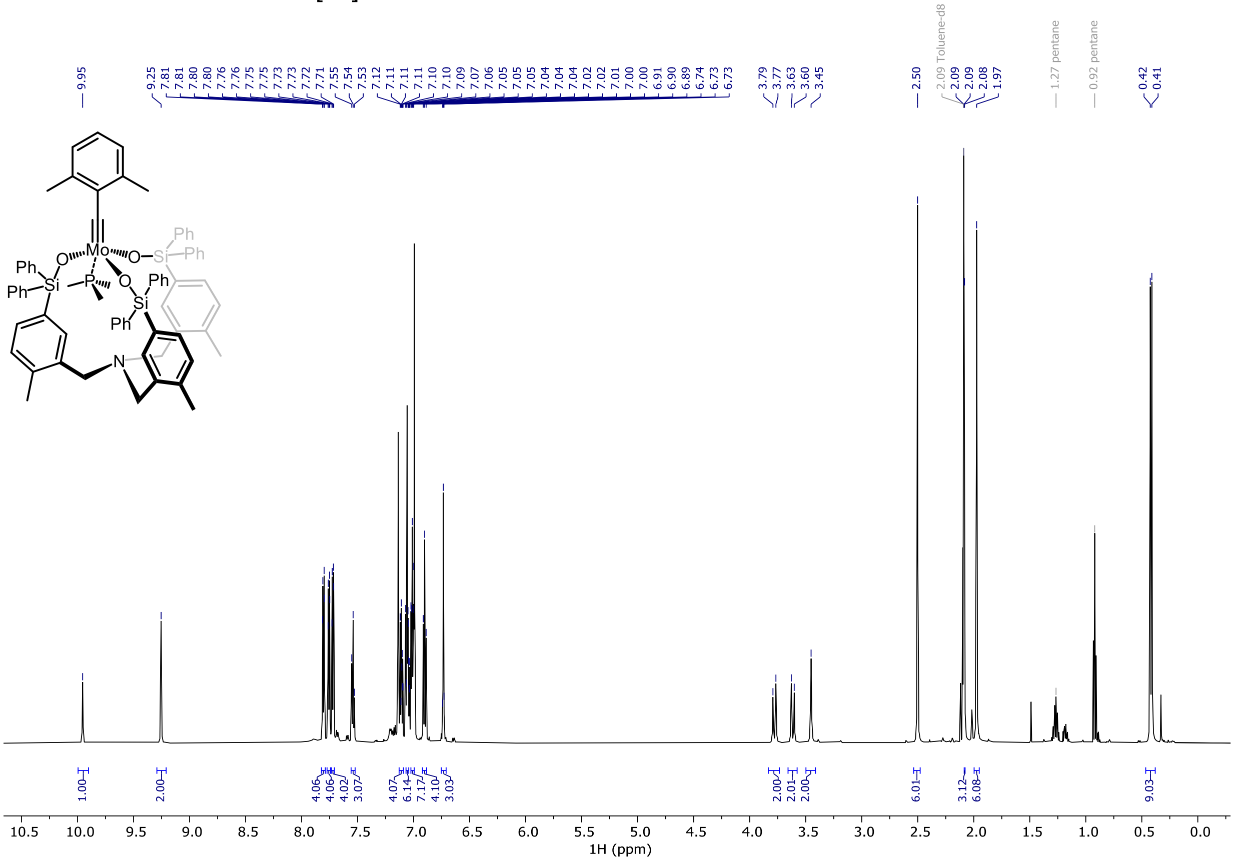

^{13}C NMR of 16f, 151 MHz, [D_8]-toluene, 233 K

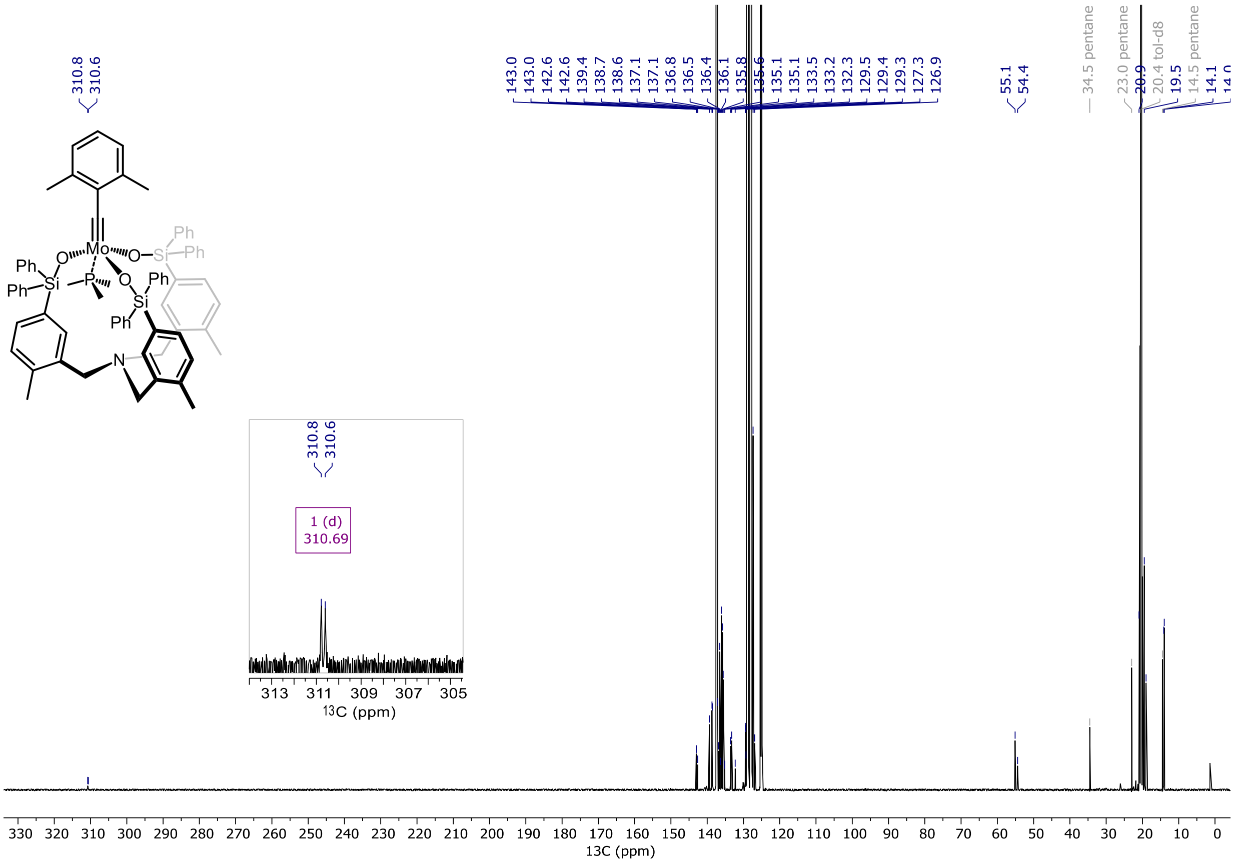

^{29}Si NMR of 16f, 119 MHz, [D$_8$]-toluene, 233 K

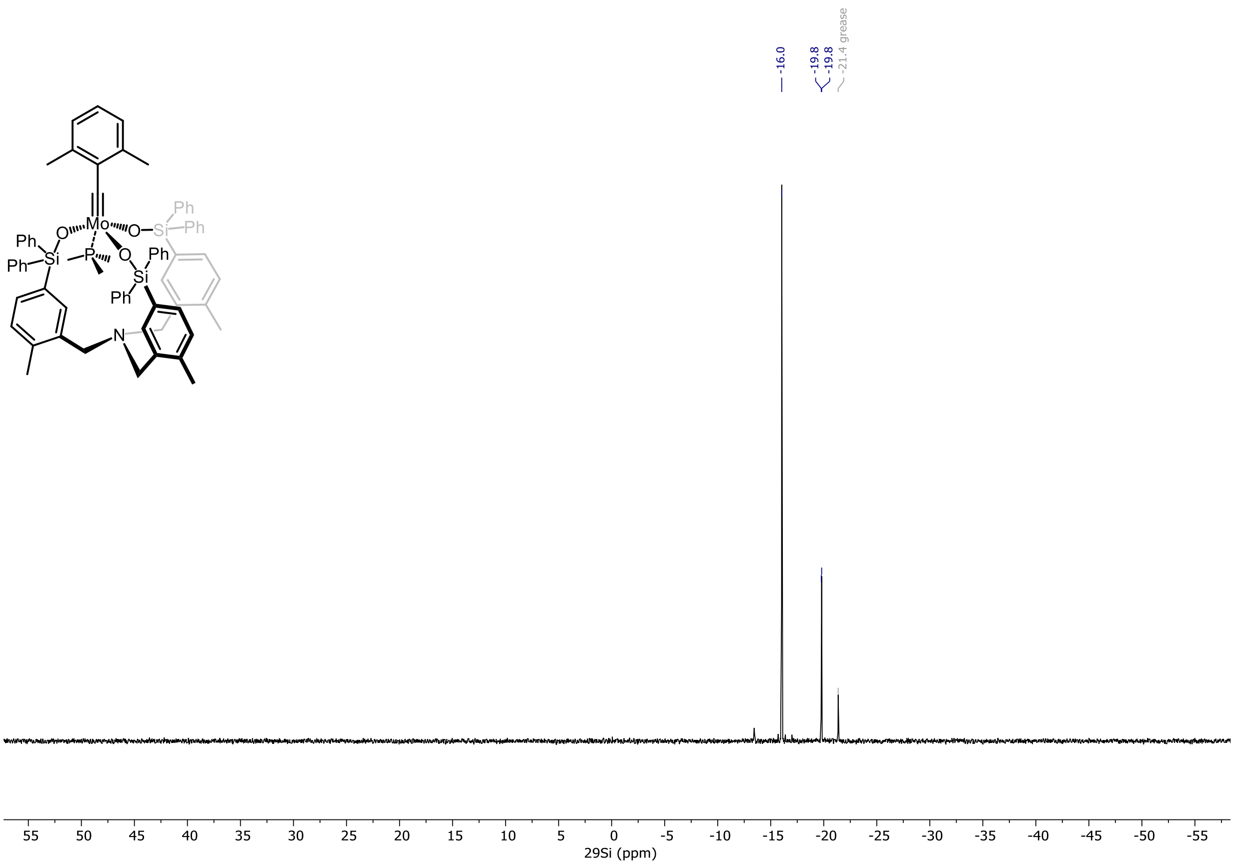

^{31}P NMR of 16f, 243 MHz, [D$_8$]-toluene, 233 K

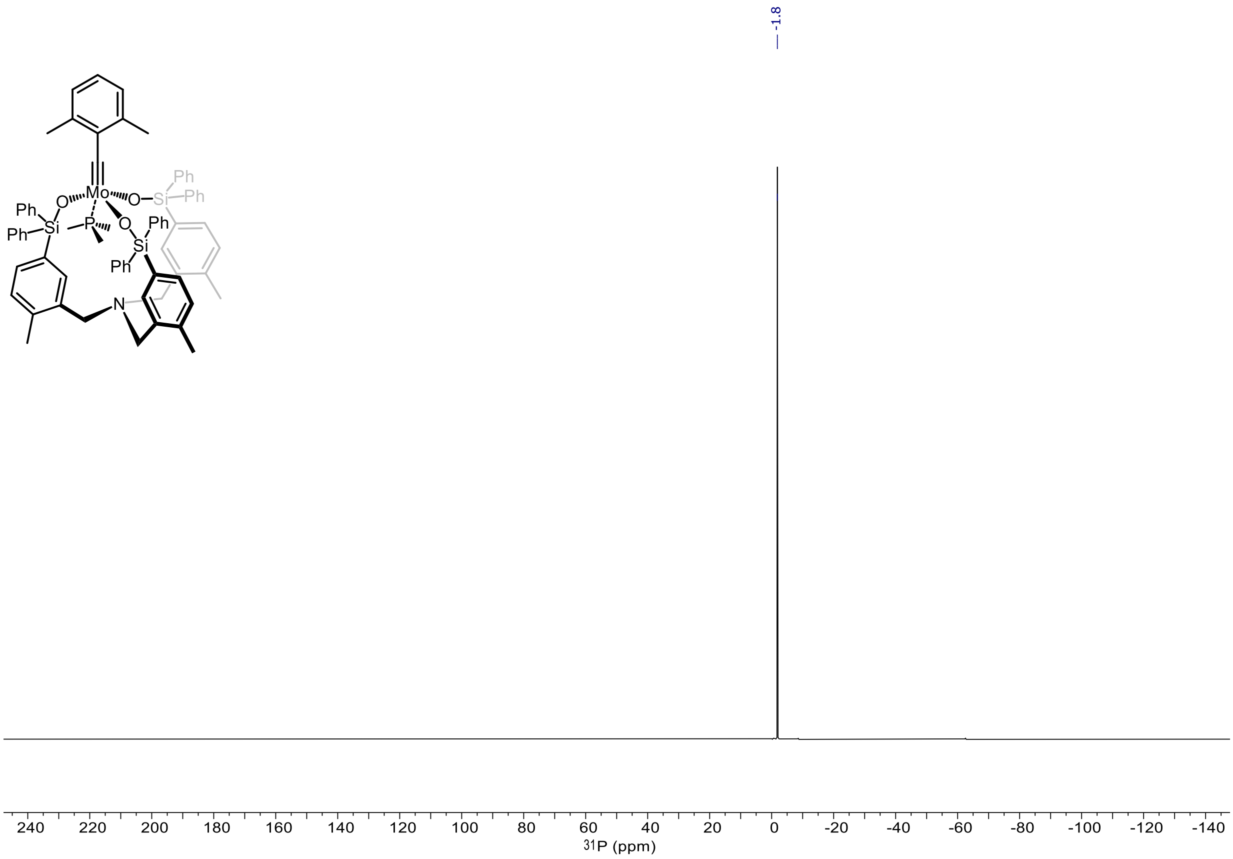

New Substrates

^{1}H NMR of S13, 400 MHz, $CDCl_3$, 25 °C

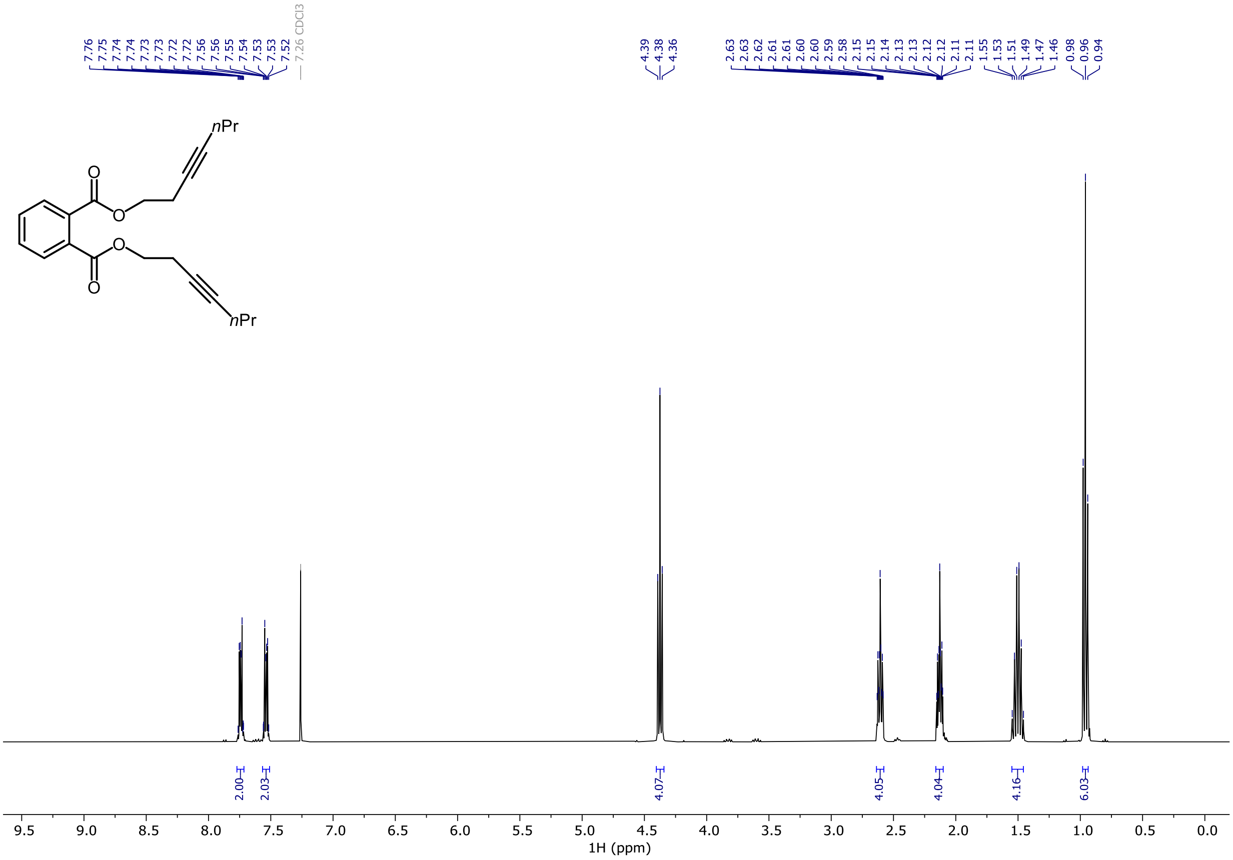

^{13}C NMR of S13, 101 MHz, $CDCl_3$, 25 °C

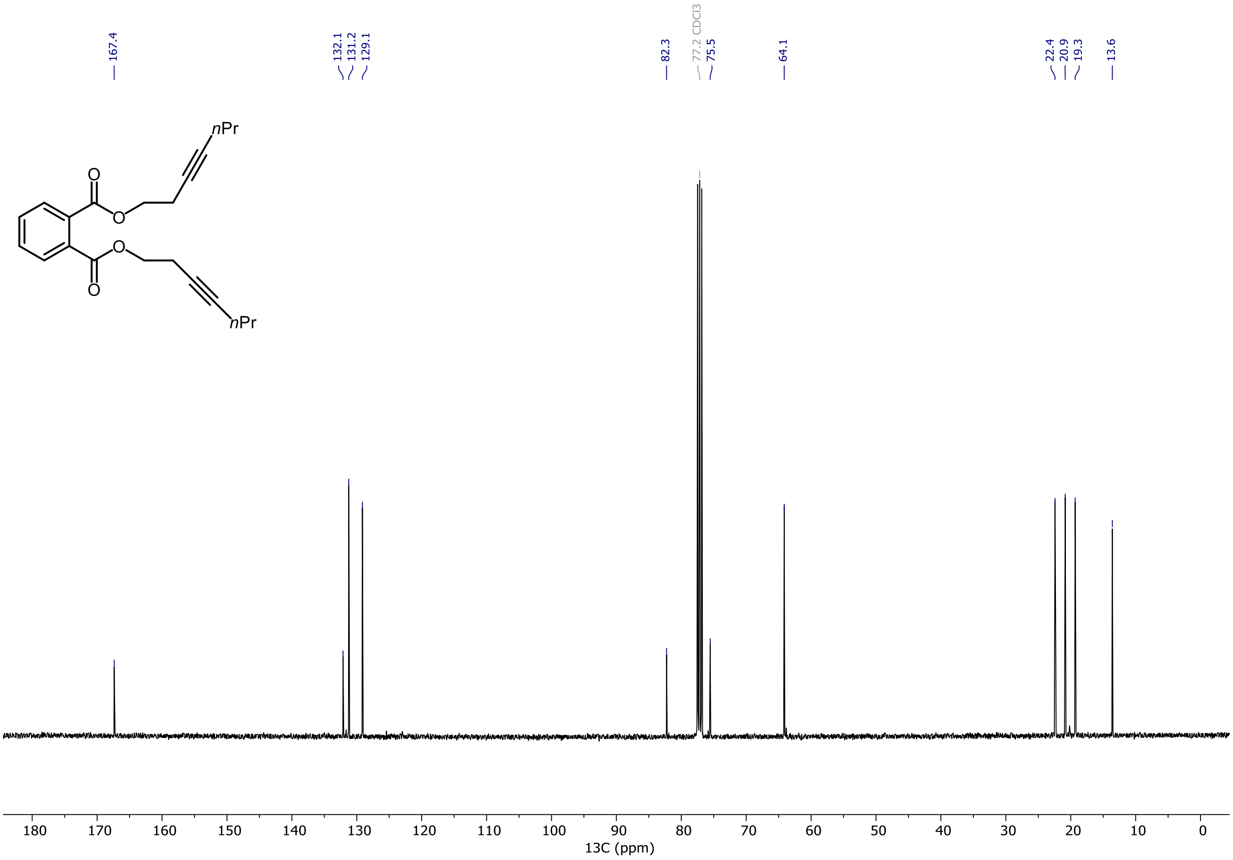

^{1}H NMR of S14, 400 MHz, $CDCl_3$, 25 °C

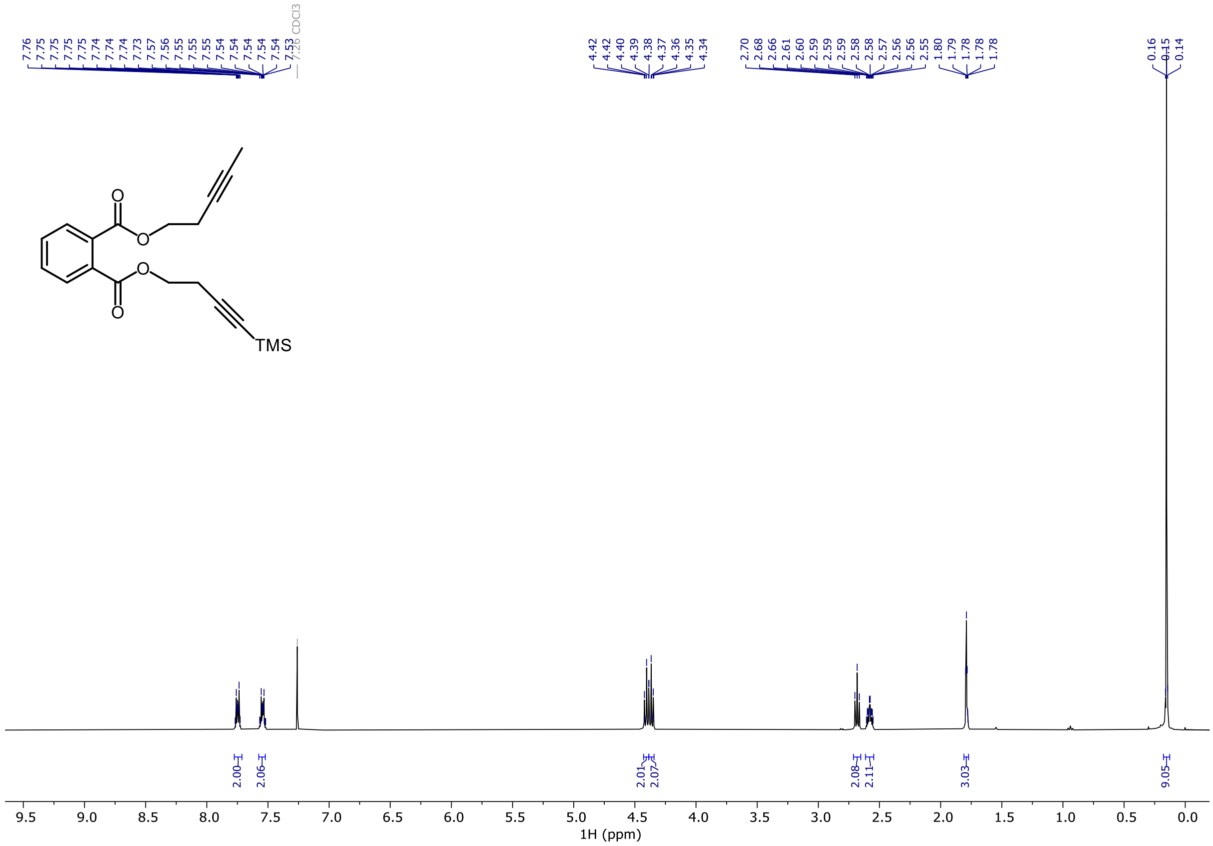

^{13}C NMR of S14, 101 MHz, $CDCl_3$, 25 °C

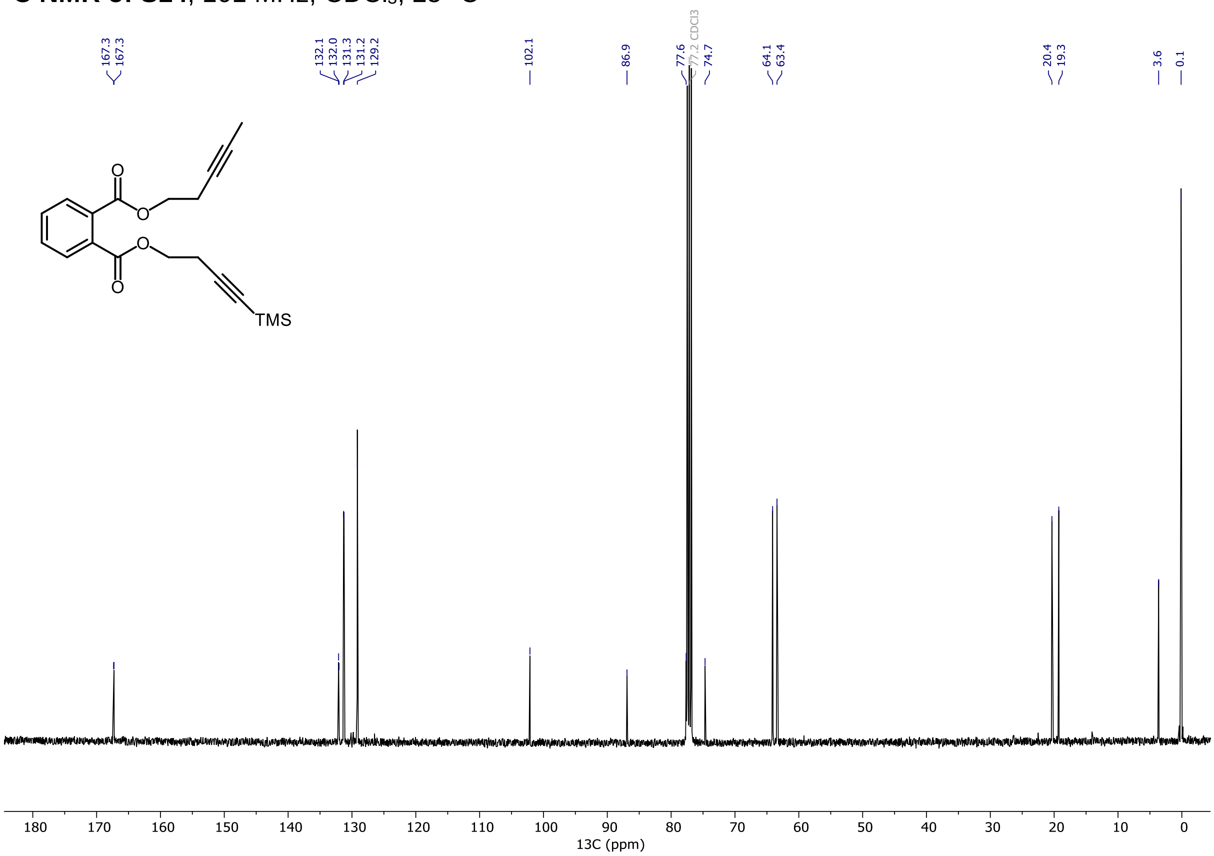

Byproduct

^{1}H NMR of S15, 400 MHz, $CDCl_3$, 25 °C

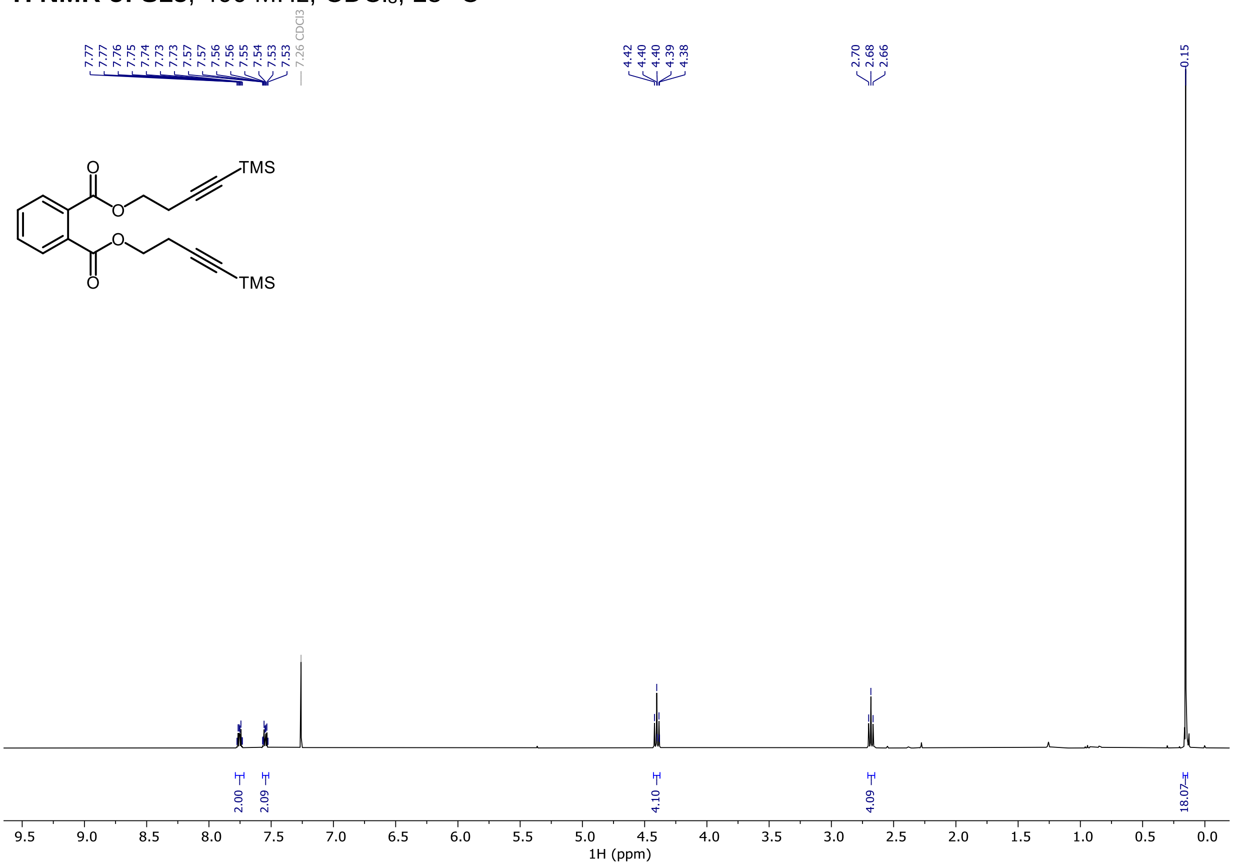

^{13}C NMR of S15, 101 MHz, $CDCl_3$, 25 °C

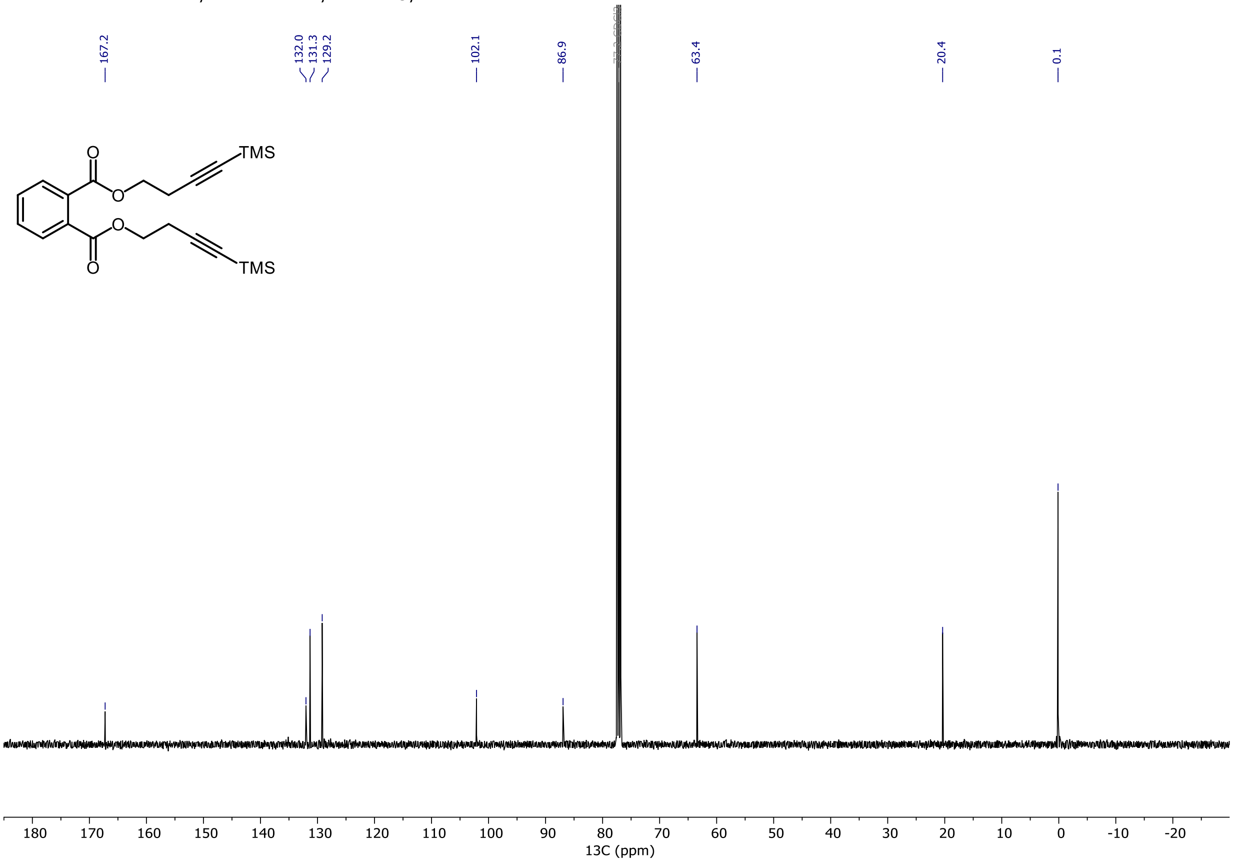

Supplement: Supplementary file 1 — ja3c10430_si_001.pdf [file ja3c10430_si_001.pdf]
