# Supplementary material for: From the Glovebox to the Benchtop: Air-Stable High Performance Molybdenum Alkylidyne Catalysts for Alkyne Metathesis
Source: J Am Chem Soc. 2023 Nov 30;145(49):26993–7009. doi: 10.1021/jacs.3c10430 (PMC10722517; doi:10.1021/jacs.3c10430)
Supplement: Supplementary file 2 — ja3c10430_si_002.pdf [file ja3c10430_si_002.pdf]

SUPPORTING CRYSTALLOGRAPHIC INFORMATION

From the Glovebox to the Benchtop: Air-Stable High Performance Molybdenum Alkylidyne Catalysts for Alkyne Metathesis

J. Nepomuk Korber, Christian Wille, Markus Leutzsch, and Alois Fürstner*

Max-Planck-Institut für Kohlenforschung, 45470 Mülheim an der Ruhr, Germany

*E-Mail: fuerstner@mpi-muelheim.mpg.de

Table of Contents

Single crystal structure analysis of 9 .... S2

Single crystal structure analysis of 10 · benzene solvate S8

Single crystal structure analysis of 14b · cyclohexane solvate S20

Single crystal structure analysis of 15a · dichloromethane solvate S27

Single crystal structure analysis of 15b · benzene solvate S39

Single crystal structure analysis of 16b S58

Single crystal structure analysis of 9

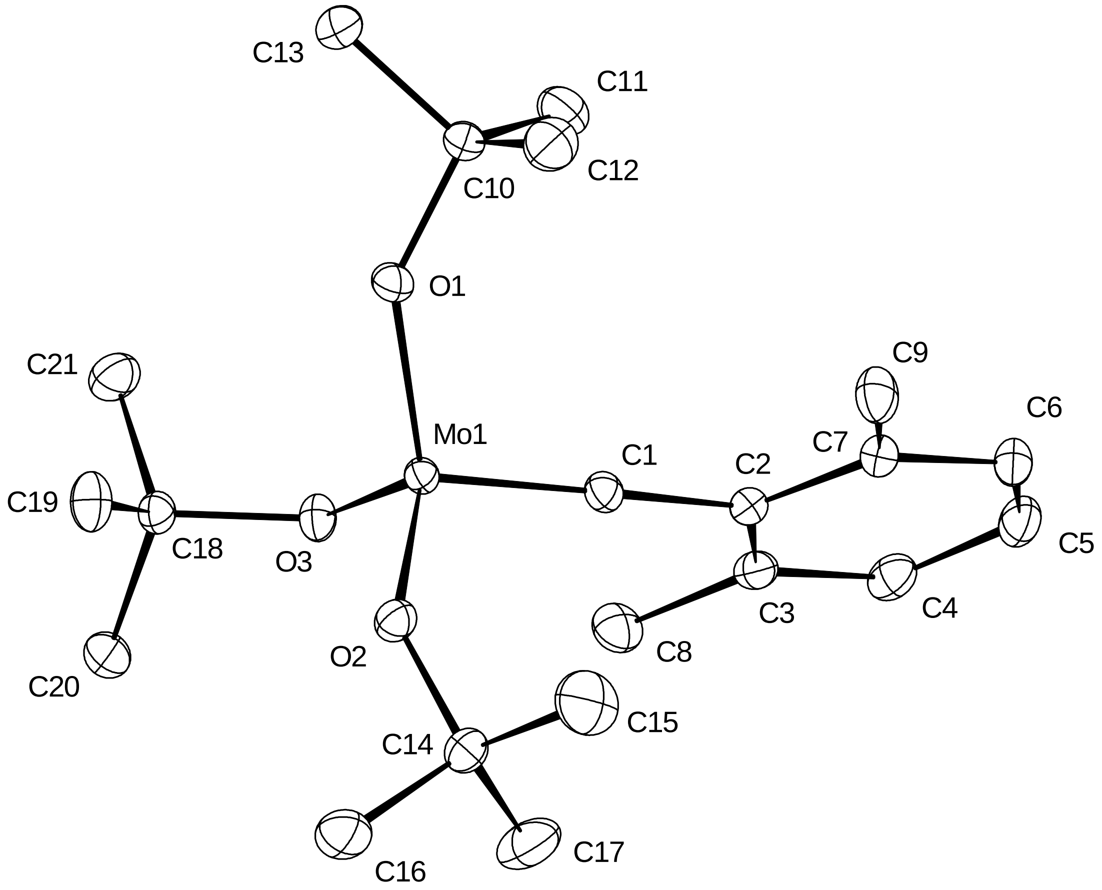

Figure S1. The molecular structure of **9**. H atoms have been removed for clarity.

X-ray Crystal Structure Analysis of 9:
$C_{21} H_{36} Mo O_3$, M_r = 432.44 g mol^{-1}, yellow prism, crystal size 0.335 x 0.324 x 0.12 mm^3, triclinic, space group *P*-1 [2], *a* = 9.695(2) Å, b = 10.945(3) Å, *c* = 11.647(3) Å, α = 98.330(10)°, β = 102.841(9)°, γ = 105.806(10)°, *V* = 1131.3(4) Å^3, *T* = 100(2) K, *Z* = 2, D_{calc} = 1.270 g·cm^3, λ = 0.71073 Å, $\mu(Mo\text{-}K_\alpha)$ = 0.594 mm^{-1}, numerical absorption correction ($T_{\min}$ = 0.8630, $T_{\max}$ = 0.9569), Bruker AXS D8-Venture diffractometer with IμS Diamond Mo-anode X-ray source and PHOTON III detector, 1.982 < θ < 33.345°, 198517 measured reflections, 8706 independent reflections, 8284 reflections with $I > 2\sigma(I)$, R_{int} = 0.0421. The structure was solved by *SHELXT* and refined by full-matrix least-squares (*SHELXL*) against F^2 to R_1 = 0.0163 [$I > 2\sigma(I)$], wR_2 = 0.0428 [all data], 237 parameters and 0 restraints.

Full .cif data for the compound are available under the CCDC number **CCDC 2293662**

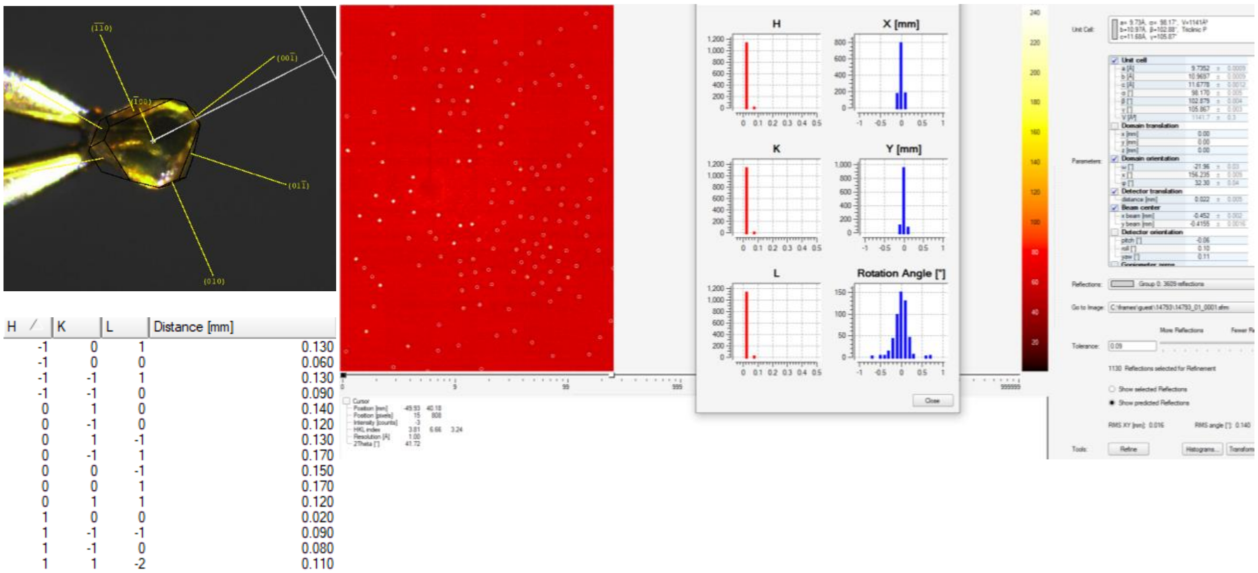

Figure S2. Crystal faces and unit cell determination/refinement of compound **9**.

INTENSITY STATISTICS FOR DATASET

Resolution	#Data	#Theory	%Complete	Redundancy	Mean I	Mean I/s	Rmerge	Rsigma
Inf - 2.56	131	142	92.3	32.64	200.75	145.61	0.0277	0.0088
2.56 - 1.74	311	311	100.0	40.10	137.28	136.75	0.0268	0.0058
1.74 - 1.38	445	445	100.0	38.74	104.38	128.57	0.0287	0.0064
1.38 - 1.21	431	431	100.0	31.47	81.89	103.65	0.0319	0.0076
1.21 - 1.10	448	448	100.0	27.66	57.79	86.84	0.0376	0.0091
1.10 - 1.02	460	460	100.0	26.25	47.69	76.43	0.0428	0.0101
1.02 - 0.96	440	440	100.0	25.25	44.57	72.25	0.0463	0.0110
0.96 - 0.91	463	463	100.0	24.37	38.97	65.38	0.0483	0.0120
0.91 - 0.87	472	472	100.0	23.42	34.18	60.30	0.0536	0.0132
0.87 - 0.84	383	383	100.0	22.96	31.71	55.67	0.0561	0.0141
0.84 - 0.81	464	464	100.0	21.47	28.03	49.62	0.0619	0.0161
0.81 - 0.79	352	352	100.0	21.03	26.27	46.99	0.0672	0.0170
0.79 - 0.76	600	600	100.0	19.67	23.90	41.98	0.0669	0.0190
0.76 - 0.74	435	435	100.0	17.71	23.22	37.77	0.0717	0.0213
0.74 - 0.72	509	509	100.0	17.61	19.61	33.83	0.0805	0.0238
0.72 - 0.71	275	275	100.0	17.57	18.43	31.69	0.0820	0.0252
0.71 - 0.69	565	565	100.0	17.21	17.07	29.06	0.0881	0.0274
0.69 - 0.68	343	343	100.0	16.88	15.34	26.36	0.0983	0.0308
0.68 - 0.67	334	334	100.0	16.75	14.56	24.80	0.1017	0.0325
0.67 - 0.65	846	888	95.3	13.67	13.43	20.69	0.1111	0.0487
0.75 - 0.65	3113	3155	98.7	16.25	16.44	27.47	0.0913	0.0316
Inf - 0.65	8707	8760	99.4	22.66	41.04	58.32	0.0420	0.0131

One reflection was shadowed by the beamstop and omitted (OMIT 0 0 1) from data set before the final refinement cycles due to high I/σI (> 10).

Table S1. Crystal data and structure refinement of compound **9**.

Identification code	14793	
Empirical formula	$C_{21} H_{36} Mo O_3$	
Color	yellow	
Formula weight	432.44 g·mol^{-1}	
Temperature	100(2) K	
Wavelength	0.71073 Å	
Crystal system	Triclinic	
Space group	*P*-1, (no. 2)	
Unit cell dimensions	a = 9.695(2) Å	α= 98.330(10)°.
	b = 10.945(3) Å	β= 102.841(9)°.
	c = 11.647(3) Å	γ = 105.806(10)°.
Volume	1131.3(4) Å^3	
Z	2	
Density (calculated)	1.270 Mg·m^{-3}	
Absorption coefficient	0.594 mm^{-1}	
F(000)	456 e	
Crystal size	0.335 x 0.324 x 0.12 mm^3	
θ range for data collection	1.982 to 33.345°.	
Index ranges	$-14 \leq h \leq 14$, $-16 \leq k \leq 16$, $-17 \leq l \leq 17$	
Reflections collected	198517	
Independent reflections	8706 [R_{int} = 0.0421]	
Reflections with I>2σ(I)	8284	
Completeness to θ = 25.242°	99.7 %	
Absorption correction	Numerical	
Max. and min. transmission	0.9569 and 0.8630	
Refinement method	Full-matrix least-squares on F^2	
Data / restraints / parameters	8706 / 0 / 237	
Goodness-of-fit on F^2	1.078	
Final R indices [I>2σ(I)]	R_1 = 0.0163	wR^2 = 0.0423
R indices (all data)	R_1 = 0.0179	wR^2 = 0.0428
Extinction coefficient	n/a	
Largest diff. peak and hole	0.474 and -0.419 e·Å^{-3}	

Table S2. Bond lengths [Å] and angles [°] of compound **9**.

Mo(1)-O(1)	1.8753(7)	Mo(1)-O(2)	1.8728(7)
Mo(1)-O(3)	1.9039(7)	Mo(1)-C(1)	1.7567(8)
O(1)-C(10)	1.4392(10)	O(2)-C(14)	1.4409(10)
O(3)-C(18)	1.4518(10)	C(1)-C(2)	1.4511(11)
C(2)-C(3)	1.4160(11)	C(2)-C(7)	1.4106(11)
C(3)-C(4)	1.3935(12)	C(3)-C(8)	1.5020(12)
C(4)-H(4)	0.9500	C(4)-C(5)	1.3899(13)
C(5)-H(5)	0.9500	C(5)-C(6)	1.3864(13)
C(6)-H(6)	0.9500	C(6)-C(7)	1.3989(12)
C(7)-C(9)	1.5060(12)	C(8)-H(8A)	0.9800
C(8)-H(8B)	0.9800	C(8)-H(8C)	0.9800
C(9)-H(9A)	0.9800	C(9)-H(9B)	0.9800
C(9)-H(9C)	0.9800	C(10)-C(11)	1.5204(13)
C(10)-C(12)	1.5210(13)	C(10)-C(13)	1.5269(12)
C(11)-H(11A)	0.9800	C(11)-H(11B)	0.9800
C(11)-H(11C)	0.9800	C(12)-H(12A)	0.9800
C(12)-H(12B)	0.9800	C(12)-H(12C)	0.9800
C(13)-H(13A)	0.9800	C(13)-H(13B)	0.9800
C(13)-H(13C)	0.9800	C(14)-C(15)	1.5122(14)
C(14)-C(16)	1.5240(13)	C(14)-C(17)	1.5252(14)
C(15)-H(15A)	0.9800	C(15)-H(15B)	0.9800
C(15)-H(15C)	0.9800	C(16)-H(16A)	0.9800
C(16)-H(16B)	0.9800	C(16)-H(16C)	0.9800
C(17)-H(17A)	0.9800	C(17)-H(17B)	0.9800
C(17)-H(17C)	0.9800	C(18)-C(19)	1.5280(12)
C(18)-C(20)	1.5210(12)	C(18)-C(21)	1.5212(12)
C(19)-H(19A)	0.9800	C(19)-H(19B)	0.9800
C(19)-H(19C)	0.9800	C(20)-H(20A)	0.9800
C(20)-H(20B)	0.9800	C(20)-H(20C)	0.9800
C(21)-H(21A)	0.9800	C(21)-H(21B)	0.9800
C(21)-H(21C)	0.9800		
O(1)-Mo(1)-O(3)	113.56(3)	O(2)-Mo(1)-O(1)	113.51(3)
O(2)-Mo(1)-O(3)	113.17(3)	C(1)-Mo(1)-O(1)	108.83(3)

C(1)-Mo(1)-O(2)	107.37(4)	C(1)-Mo(1)-O(3)	99.14(3)
C(10)-O(1)-Mo(1)	143.03(5)	C(14)-O(2)-Mo(1)	140.98(5)
C(18)-O(3)-Mo(1)	120.05(5)	C(2)-C(1)-Mo(1)	174.71(6)
C(3)-C(2)-C(1)	117.64(7)	C(7)-C(2)-C(1)	122.34(7)
C(7)-C(2)-C(3)	119.98(7)	C(2)-C(3)-C(8)	119.85(7)
C(4)-C(3)-C(2)	119.12(8)	C(4)-C(3)-C(8)	121.02(8)
C(3)-C(4)-H(4)	119.5	C(5)-C(4)-C(3)	120.96(8)
C(5)-C(4)-H(4)	119.5	C(4)-C(5)-H(5)	120.1
C(6)-C(5)-C(4)	119.82(8)	C(6)-C(5)-H(5)	120.1
C(5)-C(6)-H(6)	119.5	C(5)-C(6)-C(7)	121.07(8)
C(7)-C(6)-H(6)	119.5	C(2)-C(7)-C(9)	121.35(7)
C(6)-C(7)-C(2)	118.96(8)	C(6)-C(7)-C(9)	119.69(7)
C(3)-C(8)-H(8A)	109.5	C(3)-C(8)-H(8B)	109.5
C(3)-C(8)-H(8C)	109.5	H(8A)-C(8)-H(8B)	109.5
H(8A)-C(8)-H(8C)	109.5	H(8B)-C(8)-H(8C)	109.5
C(7)-C(9)-H(9A)	109.5	C(7)-C(9)-H(9B)	109.5
C(7)-C(9)-H(9C)	109.5	H(9A)-C(9)-H(9B)	109.5
H(9A)-C(9)-H(9C)	109.5	H(9B)-C(9)-H(9C)	109.5
O(1)-C(10)-C(11)	109.91(7)	O(1)-C(10)-C(12)	107.66(7)
O(1)-C(10)-C(13)	105.66(7)	C(11)-C(10)-C(12)	112.06(8)
C(11)-C(10)-C(13)	110.55(8)	C(12)-C(10)-C(13)	110.74(8)
C(10)-C(11)-H(11A)	109.5	C(10)-C(11)-H(11B)	109.5
C(10)-C(11)-H(11C)	109.5	H(11A)-C(11)-H(11B)	109.5
H(11A)-C(11)-H(11C)	109.5	H(11B)-C(11)-H(11C)	109.5
C(10)-C(12)-H(12A)	109.5	C(10)-C(12)-H(12B)	109.5
C(10)-C(12)-H(12C)	109.5	H(12A)-C(12)-H(12B)	109.5
H(12A)-C(12)-H(12C)	109.5	H(12B)-C(12)-H(12C)	109.5
C(10)-C(13)-H(13A)	109.5	C(10)-C(13)-H(13B)	109.5
C(10)-C(13)-H(13C)	109.5	H(13A)-C(13)-H(13B)	109.5
H(13A)-C(13)-H(13C)	109.5	H(13B)-C(13)-H(13C)	109.5
O(2)-C(14)-C(15)	108.87(8)	O(2)-C(14)-C(16)	105.10(7)
O(2)-C(14)-C(17)	109.55(7)	C(15)-C(14)-C(16)	111.45(9)
C(15)-C(14)-C(17)	111.24(9)	C(16)-C(14)-C(17)	110.43(8)
C(14)-C(15)-H(15A)	109.5	C(14)-C(15)-H(15B)	109.5
C(14)-C(15)-H(15C)	109.5	H(15A)-C(15)-H(15B)	109.5
H(15A)-C(15)-H(15C)	109.5	H(15B)-C(15)-H(15C)	109.5

C(14)-C(16)-H(16A)	109.5	C(14)-C(16)-H(16B)	109.5
C(14)-C(16)-H(16C)	109.5	H(16A)-C(16)-H(16B)	109.5
H(16A)-C(16)-H(16C)	109.5	H(16B)-C(16)-H(16C)	109.5
C(14)-C(17)-H(17A)	109.5	C(14)-C(17)-H(17B)	109.5
C(14)-C(17)-H(17C)	109.5	H(17A)-C(17)-H(17B)	109.5
H(17A)-C(17)-H(17C)	109.5	H(17B)-C(17)-H(17C)	109.5
O(3)-C(18)-C(19)	112.00(6)	O(3)-C(18)-C(20)	105.95(6)
O(3)-C(18)-C(21)	106.66(7)	C(20)-C(18)-C(19)	110.52(7)
C(20)-C(18)-C(21)	111.71(7)	C(21)-C(18)-C(19)	109.89(7)
C(18)-C(19)-H(19A)	109.5	C(18)-C(19)-H(19B)	109.5
C(18)-C(19)-H(19C)	109.5	H(19A)-C(19)-H(19B)	109.5
H(19A)-C(19)-H(19C)	109.5	H(19B)-C(19)-H(19C)	109.5
C(18)-C(20)-H(20A)	109.5	C(18)-C(20)-H(20B)	109.5
C(18)-C(20)-H(20C)	109.5	H(20A)-C(20)-H(20B)	109.5
H(20A)-C(20)-H(20C)	109.5	H(20B)-C(20)-H(20C)	109.5
C(18)-C(21)-H(21A)	109.5	C(18)-C(21)-H(21B)	109.5
C(18)-C(21)-H(21C)	109.5	H(21A)-C(21)-H(21B)	109.5
H(21A)-C(21)-H(21C)	109.5	H(21B)-C(21)-H(21C)	109.5

Single crystal structure analysis of 10 · benzene solvate

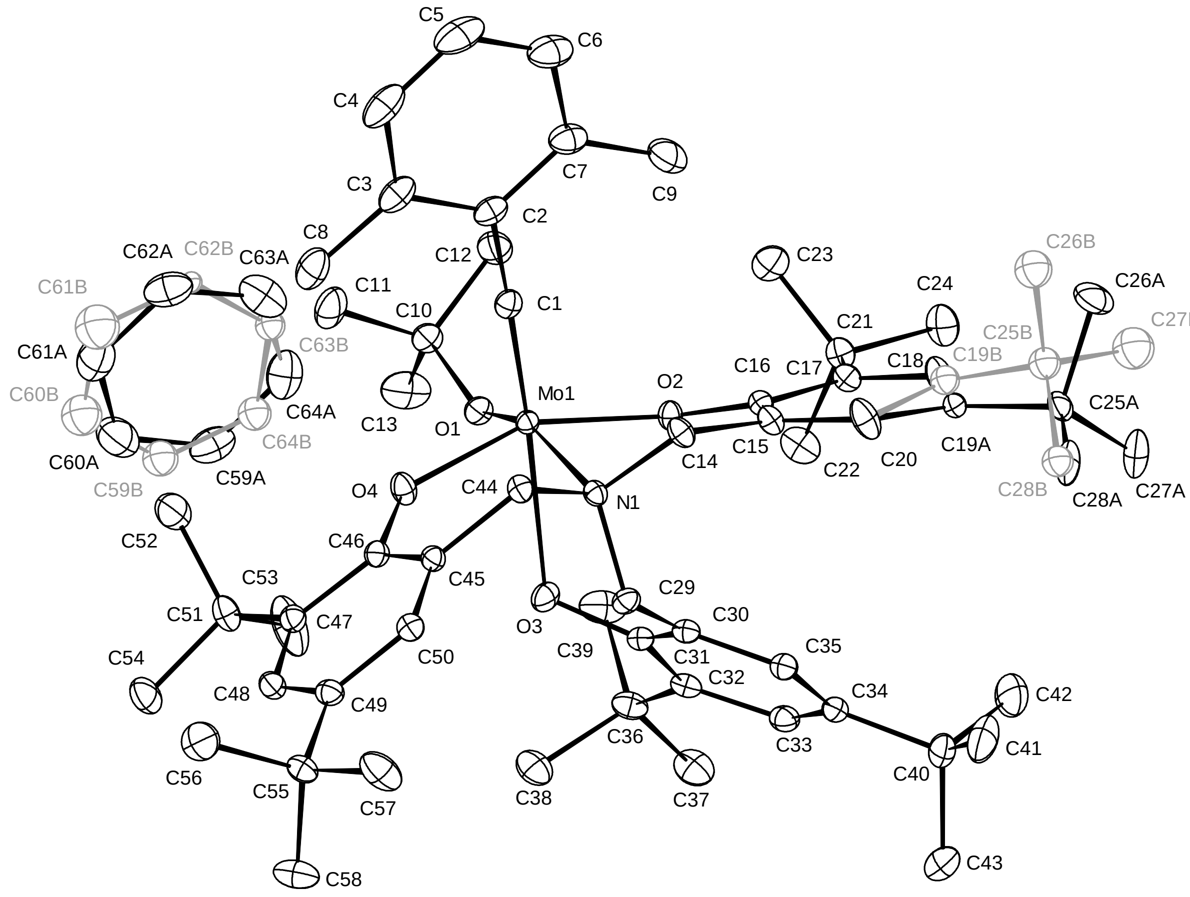

Figure S3. The molecular structure of **10 · benzene solvate**. H atoms have been removed for clarity. Main structure shown in black and disordered parts shown in grey.

X-ray Crystal Structure Analysis of 10 · benzene solvate:
C_{64} H_{91} Mo N O_4, M_r = 1034.31 g mol^{-1}, orange block, crystal size 0.14 x 0.081 x 0.081 mm^3, monoclinic, space group $P2_1/n$ [14], a = 14.2159(8) Å, b = 18.0559(10) Å, c = 22.4512(13) Å, β = 90.396(2)°, V = 5762.7(6) Å^3, T = 100(2) K, Z = 4, D_{calc} = 1.192 g·cm^3, λ = 0.71073 Å, $\mu(Mo\text{-}K_\alpha)$ = 0.274 mm^{-1}, Gaussian absorption correction ($T_{\min}$ = 0.8992, $T_{\max}$ = 1.0000), Bruker-AXS Kappa Mach3 with APEX-II detector and IµS microfocus Mo-anode X-ray source, 1.447 < θ < 30.998°, 192900 measured reflections, 18380 independent reflections, 14663 reflections with $I > 2\sigma(I)$, R_{int} = 0.0696. The structure was solved by *SHELXT* and refined by full-matrix least-squares (*SHELXL*) against F^2 to R_1 = 0.0327 [$I > 2\sigma(I)$], wR_2 = 0.0798 [all data], 693 parameters and 0 restraints.

Full .cif data for the compound are available under the CCDC number **CCDC 2293663**

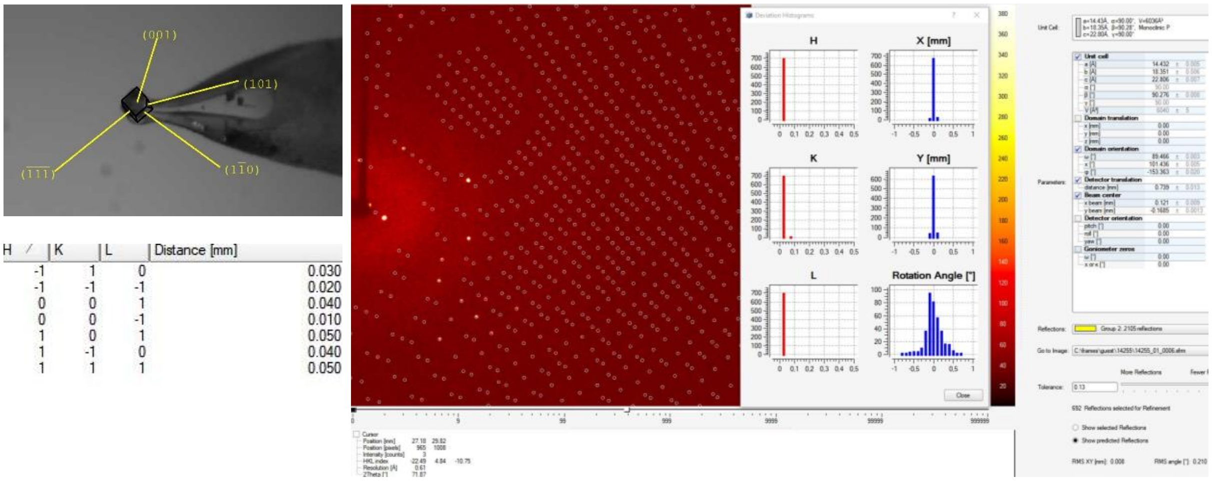

Figure S4. Crystal faces and unit cell determination/refinement of compound **10 · benzene solvate**.

INTENSITY STATISTICS FOR DATASET

Resolution	#Data	#Theory	%Complete	Redundancy	Mean I	Mean I/s	Rmerge	Rsigma
Inf - 2.78	316	316	100.0	16.75	99.90	76.14	0.0244	0.0092
2.78 - 1.84	738	738	100.0	18.09	50.35	64.50	0.0315	0.0106
1.84 - 1.45	1049	1049	100.0	18.29	31.44	54.83	0.0414	0.0127
1.45 - 1.26	1081	1081	100.0	18.16	23.95	46.28	0.0519	0.0152
1.26 - 1.14	1095	1095	100.0	17.54	20.16	40.05	0.0632	0.0176
1.14 - 1.06	1020	1020	100.0	14.89	16.52	30.72	0.0756	0.0230
1.06 - 1.00	993	993	100.0	11.74	14.10	24.88	0.0869	0.0299
1.00 - 0.95	1017	1017	100.0	10.15	12.65	20.98	0.0980	0.0354
0.95 - 0.91	1025	1025	100.0	8.91	10.84	17.74	0.1120	0.0427
0.91 - 0.87	1184	1184	100.0	7.99	9.81	15.40	0.1244	0.0497
0.87 - 0.84	1029	1029	100.0	7.69	8.74	13.56	0.1357	0.0573
0.84 - 0.81	1223	1223	100.0	7.34	7.74	11.67	0.1561	0.0660
0.81 - 0.79	870	870	100.0	7.17	8.08	11.65	0.1575	0.0676
0.79 - 0.77	1031	1031	100.0	6.88	7.27	10.42	0.1765	0.0759
0.77 - 0.75	1079	1079	100.0	6.76	6.44	9.03	0.1983	0.0873
0.75 - 0.73	1260	1260	100.0	6.47	6.68	9.10	0.2000	0.0893
0.73 - 0.72	643	643	100.0	6.39	5.71	7.89	0.2261	0.1059
0.72 - 0.70	1453	1453	100.0	6.16	5.15	6.98	0.2562	0.1202
0.70 - 0.69	815	816	99.9	5.97	5.01	6.67	0.2697	0.1284
0.69 - 0.67	1652	1700	97.2	4.98	4.48	5.35	0.3024	0.1779
0.67 - 0.66	250	562	44.5	0.81	3.07	1.77	0.4402	0.6185
0.76 - 0.66	6625	6986	94.8	5.54	5.31	6.97	0.2440	0.1326
Inf - 0.66	20823	21184	98.3	9.67	13.83	20.99	0.0692	0.0394

A resolution cut off (SHEL 999 0.69) was applied to exclude poorly determined intensities at high diffraction angles. A ^{t}Bu substituted aromatic subgroup shows a slight twofold positional disorder. This has been described with fixed occupancies of 65:35% respectively. Another twofold positional disorder can be found in the solute benzene molecule. This was described with a fixed occupancy of 90:10% and AFIX was used to fit the minor part into a hexagonal shape. Isotropic atomic displacement parameters were used to describe the minor moieties. In addition, the H position in the vicinity of the O3 atom could be located in the residual electron density map and its position

was freely refined. An additional indication of the position of a H atom bonded to O3 is given by the difference in bond length of Mo(1)-O(3): 2.4717(10) Å compared to the Mo(1)-O(2): 1.9628(9) Å and Mo(1)-O(4): 2.0000(9) Å bonds.

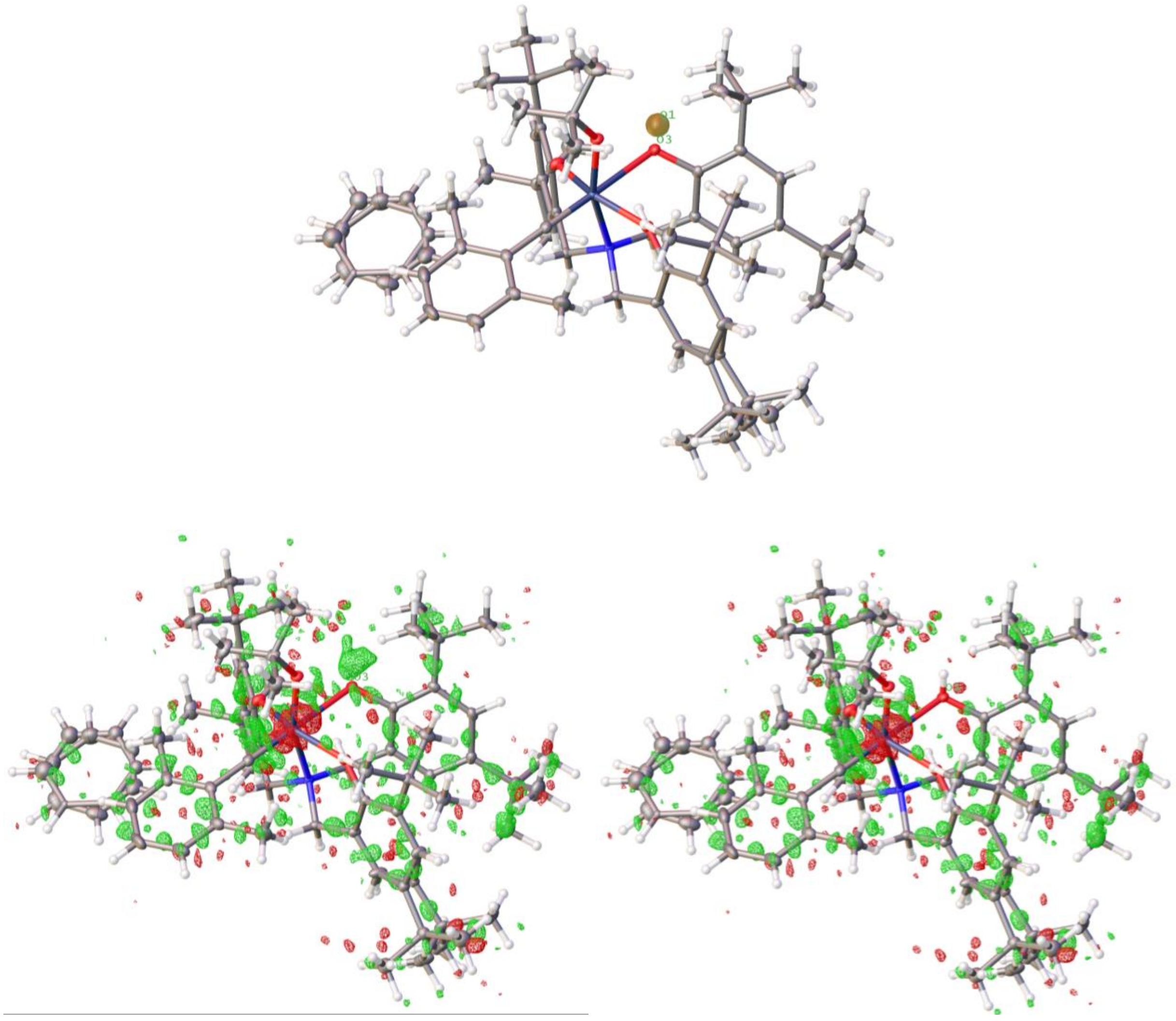

Figure S5. Structure (top) and residual electron density peak (brown sphere) of 0.78 e/A^{-3} in the vicinity of O3. Difference density distribution (bottom) of compound **10** before (left) and after (right) adding a H atom to O3 (source: Olex2, diff-map, level 0.3 $e/Å^{3}$, 0.1 Å resolution).

Table S3. Crystal data and structure refinement of compound **10 · benzene solvate**.

Identification code	14255	
Empirical formula	$C_{64} H_{91} Mo N O_4$	
Color	orange	
Formula weight	1034.31 $g \cdot mol^{-1}$	
Temperature	100(2) K	
Wavelength	0.71073 Å	
Crystal system	Monoclinic	
Space group	$P2_1/n$, (no. 14)	
Unit cell dimensions	a = 14.2159(8) Å	α= 90°.
	b = 18.0559(10) Å	β= 90.396(2)°.
	c = 22.4512(13) Å	γ = 90°.
Volume	5762.7(6) $Å^3$	
Z	4	
Density (calculated)	1.192 $Mg \cdot m^{-3}$	
Absorption coefficient	0.274 mm^{-1}	
F(000)	2224 e	
Crystal size	0.14 x 0.081 x 0.081 mm^3	
θ range for data collection	1.447 to 30.998°.	
Index ranges	$-20 \le h \le 20$, $-26 \le k \le 26$, $-32 \le l \le 32$	
Reflections collected	192900	
Independent reflections	18380 [R_{int} = 0.0696]	
Reflections with $I>2\sigma(I)$	14663	
Completeness to θ = 25.242°	100.0 %	
Absorption correction	Gaussian	
Max. and min. transmission	1.0000 and 0.8992	
Refinement method	Full-matrix least-squares on F^2	
Data / restraints / parameters	18380 / 0 / 693	
Goodness-of-fit on F^2	1.028	
Final R indices [$I>2\sigma(I)$]	R_1 = 0.0327	wR^2 = 0.0728
R indices (all data)	R_1 = 0.0502	wR^2 = 0.0798
Extinction coefficient	n/a	
Largest diff. peak and hole	0.467 and -0.536 $e \cdot Å^{-3}$	

Table S4. Bond lengths [Å] and angles [°] of compound **10 · benzene solvate**.

Mo(1)-O(1)	1.9029(9)	Mo(1)-O(2)	1.9628(9)
Mo(1)-O(3)	2.4717(10)	Mo(1)-O(4)	2.0000(9)
Mo(1)-N(1)	2.2945(11)	Mo(1)-C(1)	1.7559(14)
O(1)-C(10)	1.4417(16)	O(2)-C(16)	1.3489(15)
O(3)-H(3)	0.77(2)	O(3)-C(31)	1.3878(16)
O(4)-C(46)	1.3360(16)	N(1)-C(14)	1.4918(16)
N(1)-C(29)	1.5033(17)	N(1)-C(44)	1.5092(17)
C(1)-C(2)	1.4515(19)	C(2)-C(3)	1.410(2)
C(2)-C(7)	1.412(2)	C(3)-C(4)	1.394(2)
C(3)-C(8)	1.503(2)	C(4)-H(4)	0.9500
C(4)-C(5)	1.379(3)	C(5)-H(5)	0.9500
C(5)-C(6)	1.377(3)	C(6)-H(6)	0.9500
C(6)-C(7)	1.393(2)	C(7)-C(9)	1.497(2)
C(8)-H(8A)	0.9800	C(8)-H(8B)	0.9800
C(8)-H(8C)	0.9800	C(9)-H(9A)	0.9800
C(9)-H(9B)	0.9800	C(9)-H(9C)	0.9800
C(10)-C(11)	1.511(2)	C(10)-C(12)	1.519(2)
C(10)-C(13)	1.526(2)	C(11)-H(11A)	0.9800
C(11)-H(11B)	0.9800	C(11)-H(11C)	0.9800
C(12)-H(12A)	0.9800	C(12)-H(12B)	0.9800
C(12)-H(12C)	0.9800	C(13)-H(13A)	0.9800
C(13)-H(13B)	0.9800	C(13)-H(13C)	0.9800
C(14)-H(14A)	0.9900	C(14)-H(14B)	0.9900
C(14)-C(15)	1.4996(18)	C(15)-C(16)	1.3989(19)
C(15)-C(20)	1.3940(18)	C(16)-C(17)	1.4170(18)
C(17)-C(18)	1.3939(18)	C(17)-C(21)	1.5286(19)
C(18)-H(18)	0.9500	C(18)-H(18A)	0.9500
C(18)-C(19A)	1.393(3)	C(18)-C(19B)	1.422(7)
C(19A)-C(20)	1.421(3)	C(19A)-C(25A)	1.539(3)
C(19B)-C(20)	1.348(7)	C(19B)-C(25B)	1.528(8)
C(20)-H(20)	0.9500	C(20)-H(20A)	0.9500
C(21)-C(22)	1.530(2)	C(21)-C(23)	1.533(2)
C(21)-C(24)	1.533(2)	C(22)-H(22A)	0.9800
C(22)-H(22B)	0.9800	C(22)-H(22C)	0.9800

C(23)-H(23A)	0.9800	C(23)-H(23B)	0.9800
C(23)-H(23C)	0.9800	C(24)-H(24A)	0.9800
C(24)-H(24B)	0.9800	C(24)-H(24C)	0.9800
C(25A)-C(26A)	1.528(3)	C(25A)-C(27A)	1.543(4)
C(25A)-C(28A)	1.379(6)	C(25B)-C(26B)	1.532(7)
C(25B)-C(27B)	1.527(7)	C(25B)-C(28B)	1.773(12)
C(26A)-H(26A)	0.9800	C(26A)-H(26B)	0.9800
C(26A)-H(26C)	0.9800	C(26B)-H(26D)	0.9800
C(26B)-H(26E)	0.9800	C(26B)-H(26F)	0.9800
C(27A)-H(27A)	0.9800	C(27A)-H(27B)	0.9800
C(27A)-H(27C)	0.9800	C(27B)-H(27D)	0.9800
C(27B)-H(27E)	0.9800	C(27B)-H(27F)	0.9800
C(28A)-H(28A)	0.9800	C(28A)-H(28B)	0.9800
C(28A)-H(28C)	0.9800	C(28B)-H(28D)	0.9800
C(28B)-H(28E)	0.9800	C(28B)-H(28F)	0.9800
C(29)-H(29A)	0.9900	C(29)-H(29B)	0.9900
C(29)-C(30)	1.5084(19)	C(30)-C(31)	1.3906(18)
C(30)-C(35)	1.3914(18)	C(31)-C(32)	1.3994(19)
C(32)-C(33)	1.3983(19)	C(32)-C(36)	1.5463(19)
C(33)-H(33)	0.9500	C(33)-C(34)	1.3938(19)
C(34)-C(35)	1.3868(19)	C(34)-C(40)	1.5300(19)
C(35)-H(35)	0.9500	C(36)-C(37)	1.533(2)
C(36)-C(38)	1.543(2)	C(36)-C(39)	1.541(2)
C(37)-H(37A)	0.9800	C(37)-H(37B)	0.9800
C(37)-H(37C)	0.9800	C(38)-H(38A)	0.9800
C(38)-H(38B)	0.9800	C(38)-H(38C)	0.9800
C(39)-H(39A)	0.9800	C(39)-H(39B)	0.9800
C(39)-H(39C)	0.9800	C(40)-C(41)	1.525(2)
C(40)-C(42)	1.524(2)	C(40)-C(43)	1.540(2)
C(41)-H(41A)	0.9800	C(41)-H(41B)	0.9800
C(41)-H(41C)	0.9800	C(42)-H(42A)	0.9800
C(42)-H(42B)	0.9800	C(42)-H(42C)	0.9800
C(43)-H(43A)	0.9800	C(43)-H(43B)	0.9800
C(43)-H(43C)	0.9800	C(44)-H(44A)	0.9900
C(44)-H(44B)	0.9900	C(44)-C(45)	1.4946(17)
C(45)-C(46)	1.3978(18)	C(45)-C(50)	1.3914(18)

C(46)-C(47)	1.4187(18)	C(47)-C(48)	1.3947(19)
C(47)-C(51)	1.5292(19)	C(48)-H(48)	0.9500
C(48)-C(49)	1.4026(19)	C(49)-C(50)	1.3857(18)
C(49)-C(55)	1.5334(19)	C(50)-H(50)	0.9500
C(51)-C(52)	1.534(2)	C(51)-C(53)	1.537(2)
C(51)-C(54)	1.534(2)	C(52)-H(52A)	0.9800
C(52)-H(52B)	0.9800	C(52)-H(52C)	0.9800
C(53)-H(53A)	0.9800	C(53)-H(53B)	0.9800
C(53)-H(53C)	0.9800	C(54)-H(54A)	0.9800
C(54)-H(54B)	0.9800	C(54)-H(54C)	0.9800
C(55)-C(56)	1.536(2)	C(55)-C(57)	1.530(2)
C(55)-C(58)	1.526(2)	C(56)-H(56A)	0.9800
C(56)-H(56B)	0.9800	C(56)-H(56C)	0.9800
C(57)-H(57A)	0.9800	C(57)-H(57B)	0.9800
C(57)-H(57C)	0.9800	C(58)-H(58A)	0.9800
C(58)-H(58B)	0.9800	C(58)-H(58C)	0.9800
C(59A)-H(59A)	0.9500	C(59A)-C(60A)	1.381(3)
C(59A)-C(64A)	1.375(3)	C(60A)-H(60A)	0.9500
C(60A)-C(61A)	1.387(4)	C(61A)-H(61A)	0.9500
C(61A)-C(62A)	1.368(4)	C(62A)-H(62A)	0.9500
C(62A)-C(63A)	1.384(4)	C(63A)-H(63A)	0.9500
C(63A)-C(64A)	1.384(3)	C(64A)-H(64A)	0.9500
C(64B)-H(64B)	0.9500	C(64B)-C(59B)	1.3900
C(64B)-C(63B)	1.3900	C(59B)-H(59B)	0.9500
C(59B)-C(60B)	1.3900	C(60B)-H(60B)	0.9500
C(60B)-C(61B)	1.3900	C(61B)-H(61B)	0.9500
C(61B)-C(62B)	1.3900	C(62B)-H(62B)	0.9500
C(62B)-C(63B)	1.3900	C(63B)-H(63B)	0.9500
O(1)-Mo(1)-O(2)	96.15(4)	O(1)-Mo(1)-O(3)	79.10(4)
O(1)-Mo(1)-O(4)	95.03(4)	O(1)-Mo(1)-N(1)	159.34(4)
O(2)-Mo(1)-O(3)	82.78(4)	O(2)-Mo(1)-O(4)	154.44(4)
O(2)-Mo(1)-N(1)	79.93(4)	O(4)-Mo(1)-O(3)	76.88(4)
O(4)-Mo(1)-N(1)	81.51(4)	N(1)-Mo(1)-O(3)	80.28(4)
C(1)-Mo(1)-O(1)	105.90(5)	C(1)-Mo(1)-O(2)	101.34(5)
C(1)-Mo(1)-O(3)	172.98(5)	C(1)-Mo(1)-O(4)	97.56(5)

C(1)-Mo(1)-N(1)	94.75(5)	C(10)-O(1)-Mo(1)	138.53(9)
C(16)-O(2)-Mo(1)	140.59(9)	Mo(1)-O(3)-H(3)	104.0(17)
C(31)-O(3)-Mo(1)	117.27(8)	C(31)-O(3)-H(3)	114.6(17)
C(46)-O(4)-Mo(1)	139.50(8)	C(14)-N(1)-Mo(1)	110.98(8)
C(14)-N(1)-C(29)	110.42(10)	C(14)-N(1)-C(44)	103.52(10)
C(29)-N(1)-Mo(1)	114.54(8)	C(29)-N(1)-C(44)	107.37(10)
C(44)-N(1)-Mo(1)	109.36(7)	C(2)-C(1)-Mo(1)	174.94(11)
C(3)-C(2)-C(1)	119.76(13)	C(3)-C(2)-C(7)	120.47(14)
C(7)-C(2)-C(1)	119.74(13)	C(2)-C(3)-C(8)	121.12(14)
C(4)-C(3)-C(2)	118.35(15)	C(4)-C(3)-C(8)	120.51(15)
C(3)-C(4)-H(4)	119.3	C(5)-C(4)-C(3)	121.33(16)
C(5)-C(4)-H(4)	119.3	C(4)-C(5)-H(5)	119.9
C(6)-C(5)-C(4)	120.13(15)	C(6)-C(5)-H(5)	119.9
C(5)-C(6)-H(6)	119.5	C(5)-C(6)-C(7)	121.04(16)
C(7)-C(6)-H(6)	119.5	C(2)-C(7)-C(9)	120.87(13)
C(6)-C(7)-C(2)	118.68(15)	C(6)-C(7)-C(9)	120.45(15)
C(3)-C(8)-H(8A)	109.5	C(3)-C(8)-H(8B)	109.5
C(3)-C(8)-H(8C)	109.5	H(8A)-C(8)-H(8B)	109.5
H(8A)-C(8)-H(8C)	109.5	H(8B)-C(8)-H(8C)	109.5
C(7)-C(9)-H(9A)	109.5	C(7)-C(9)-H(9B)	109.5
C(7)-C(9)-H(9C)	109.5	H(9A)-C(9)-H(9B)	109.5
H(9A)-C(9)-H(9C)	109.5	H(9B)-C(9)-H(9C)	109.5
O(1)-C(10)-C(11)	108.93(11)	O(1)-C(10)-C(12)	109.65(11)
O(1)-C(10)-C(13)	106.31(12)	C(11)-C(10)-C(12)	111.16(13)
C(11)-C(10)-C(13)	110.85(13)	C(12)-C(10)-C(13)	109.81(13)
C(10)-C(11)-H(11A)	109.5	C(10)-C(11)-H(11B)	109.5
C(10)-C(11)-H(11C)	109.5	H(11A)-C(11)-H(11B)	109.5
H(11A)-C(11)-H(11C)	109.5	H(11B)-C(11)-H(11C)	109.5
C(10)-C(12)-H(12A)	109.5	C(10)-C(12)-H(12B)	109.5
C(10)-C(12)-H(12C)	109.5	H(12A)-C(12)-H(12B)	109.5
H(12A)-C(12)-H(12C)	109.5	H(12B)-C(12)-H(12C)	109.5
C(10)-C(13)-H(13A)	109.5	C(10)-C(13)-H(13B)	109.5
C(10)-C(13)-H(13C)	109.5	H(13A)-C(13)-H(13B)	109.5
H(13A)-C(13)-H(13C)	109.5	H(13B)-C(13)-H(13C)	109.5
N(1)-C(14)-H(14A)	107.9	N(1)-C(14)-H(14B)	107.9
N(1)-C(14)-C(15)	117.70(11)	H(14A)-C(14)-H(14B)	107.2

C(15)-C(14)-H(14A)	107.9	C(15)-C(14)-H(14B)	107.9
C(16)-C(15)-C(14)	121.76(12)	C(20)-C(15)-C(14)	117.50(12)
C(20)-C(15)-C(16)	120.57(12)	O(2)-C(16)-C(15)	119.30(11)
O(2)-C(16)-C(17)	121.35(12)	C(15)-C(16)-C(17)	119.28(12)
C(16)-C(17)-C(21)	121.94(11)	C(18)-C(17)-C(16)	117.49(12)
C(18)-C(17)-C(21)	120.57(12)	C(17)-C(18)-H(18)	117.5
C(17)-C(18)-H(18A)	119.3	C(17)-C(18)-C(19B)	121.4(3)
C(19A)-C(18)-C(17)	125.04(16)	C(19A)-C(18)-H(18)	117.5
C(19B)-C(18)-H(18A)	119.3	C(18)-C(19A)-C(20)	114.5(2)
C(18)-C(19A)-C(25A)	121.6(2)	C(20)-C(19A)-C(25A)	123.1(2)
C(18)-C(19B)-C(25B)	121.0(5)	C(20)-C(19B)-C(18)	117.4(5)
C(20)-C(19B)-C(25B)	120.4(5)	C(15)-C(20)-C(19A)	121.74(17)
C(15)-C(20)-H(20)	119.1	C(15)-C(20)-H(20A)	119.9
C(19A)-C(20)-H(20)	119.1	C(19B)-C(20)-C(15)	120.2(3)
C(19B)-C(20)-H(20A)	119.9	C(17)-C(21)-C(22)	109.01(11)
C(17)-C(21)-C(23)	109.38(12)	C(17)-C(21)-C(24)	112.58(11)
C(22)-C(21)-C(23)	110.44(12)	C(22)-C(21)-C(24)	108.26(12)
C(23)-C(21)-C(24)	107.16(12)	C(21)-C(22)-H(22A)	109.5
C(21)-C(22)-H(22B)	109.5	C(21)-C(22)-H(22C)	109.5
H(22A)-C(22)-H(22B)	109.5	H(22A)-C(22)-H(22C)	109.5
H(22B)-C(22)-H(22C)	109.5	C(21)-C(23)-H(23A)	109.5
C(21)-C(23)-H(23B)	109.5	C(21)-C(23)-H(23C)	109.5
H(23A)-C(23)-H(23B)	109.5	H(23A)-C(23)-H(23C)	109.5
H(23B)-C(23)-H(23C)	109.5	C(21)-C(24)-H(24A)	109.5
C(21)-C(24)-H(24B)	109.5	C(21)-C(24)-H(24C)	109.5
H(24A)-C(24)-H(24B)	109.5	H(24A)-C(24)-H(24C)	109.5
H(24B)-C(24)-H(24C)	109.5	C(19A)-C(25A)-C(27A)	109.9(2)
C(26A)-C(25A)-C(19A)	109.4(2)	C(26A)-C(25A)-C(27A)	108.3(2)
C(28A)-C(25A)-C(19A)	114.2(3)	C(28A)-C(25A)-C(26A)	110.1(3)
C(28A)-C(25A)-C(27A)	104.7(3)	C(19B)-C(25B)-C(26B)	107.5(4)
C(19B)-C(25B)-C(28B)	107.2(5)	C(26B)-C(25B)-C(28B)	103.5(5)
C(27B)-C(25B)-C(19B)	113.3(4)	C(27B)-C(25B)-C(26B)	108.6(4)
C(27B)-C(25B)-C(28B)	116.0(5)	C(25A)-C(26A)-H(26A)	109.5
C(25A)-C(26A)-H(26B)	109.5	C(25A)-C(26A)-H(26C)	109.5
H(26A)-C(26A)-H(26B)	109.5	H(26A)-C(26A)-H(26C)	109.5
H(26B)-C(26A)-H(26C)	109.5	C(25B)-C(26B)-H(26D)	109.5

C(25B)-C(26B)-H(26E)	109.5	C(25B)-C(26B)-H(26F)	109.5
H(26D)-C(26B)-H(26E)	109.5	H(26D)-C(26B)-H(26F)	109.5
H(26E)-C(26B)-H(26F)	109.5	C(25A)-C(27A)-H(27A)	109.5
C(25A)-C(27A)-H(27B)	109.5	C(25A)-C(27A)-H(27C)	109.5
H(27A)-C(27A)-H(27B)	109.5	H(27A)-C(27A)-H(27C)	109.5
H(27B)-C(27A)-H(27C)	109.5	C(25B)-C(27B)-H(27D)	109.5
C(25B)-C(27B)-H(27E)	109.5	C(25B)-C(27B)-H(27F)	109.5
H(27D)-C(27B)-H(27E)	109.5	H(27D)-C(27B)-H(27F)	109.5
H(27E)-C(27B)-H(27F)	109.5	C(25A)-C(28A)-H(28A)	109.5
C(25A)-C(28A)-H(28B)	109.5	C(25A)-C(28A)-H(28C)	109.5
H(28A)-C(28A)-H(28B)	109.5	H(28A)-C(28A)-H(28C)	109.5
H(28B)-C(28A)-H(28C)	109.5	C(25B)-C(28B)-H(28D)	109.5
C(25B)-C(28B)-H(28E)	109.5	C(25B)-C(28B)-H(28F)	109.5
H(28D)-C(28B)-H(28E)	109.5	H(28D)-C(28B)-H(28F)	109.5
H(28E)-C(28B)-H(28F)	109.5	N(1)-C(29)-H(29A)	107.5
N(1)-C(29)-H(29B)	107.5	N(1)-C(29)-C(30)	119.16(11)
H(29A)-C(29)-H(29B)	107.0	C(30)-C(29)-H(29A)	107.5
C(30)-C(29)-H(29B)	107.5	C(31)-C(30)-C(29)	120.74(12)
C(31)-C(30)-C(35)	118.57(13)	C(35)-C(30)-C(29)	120.01(12)
O(3)-C(31)-C(30)	113.36(12)	O(3)-C(31)-C(32)	123.59(12)
C(30)-C(31)-C(32)	123.04(12)	C(31)-C(32)-C(36)	122.68(12)
C(33)-C(32)-C(31)	115.37(12)	C(33)-C(32)-C(36)	121.85(12)
C(32)-C(33)-H(33)	118.1	C(34)-C(33)-C(32)	123.79(13)
C(34)-C(33)-H(33)	118.1	C(33)-C(34)-C(40)	119.83(13)
C(35)-C(34)-C(33)	117.88(12)	C(35)-C(34)-C(40)	122.24(13)
C(30)-C(35)-H(35)	119.4	C(34)-C(35)-C(30)	121.20(13)
C(34)-C(35)-H(35)	119.4	C(37)-C(36)-C(32)	111.46(12)
C(37)-C(36)-C(38)	106.10(12)	C(37)-C(36)-C(39)	106.91(13)
C(38)-C(36)-C(32)	110.30(12)	C(39)-C(36)-C(32)	111.76(11)
C(39)-C(36)-C(38)	110.11(13)	C(36)-C(37)-H(37A)	109.5
C(36)-C(37)-H(37B)	109.5	C(36)-C(37)-H(37C)	109.5
H(37A)-C(37)-H(37B)	109.5	H(37A)-C(37)-H(37C)	109.5
H(37B)-C(37)-H(37C)	109.5	C(36)-C(38)-H(38A)	109.5
C(36)-C(38)-H(38B)	109.5	C(36)-C(38)-H(38C)	109.5
H(38A)-C(38)-H(38B)	109.5	H(38A)-C(38)-H(38C)	109.5
H(38B)-C(38)-H(38C)	109.5	C(36)-C(39)-H(39A)	109.5

C(36)-C(39)-H(39B)	109.5	C(36)-C(39)-H(39C)	109.5
H(39A)-C(39)-H(39B)	109.5	H(39A)-C(39)-H(39C)	109.5
H(39B)-C(39)-H(39C)	109.5	C(34)-C(40)-C(43)	109.27(12)
C(41)-C(40)-C(34)	112.50(13)	C(41)-C(40)-C(43)	107.07(14)
C(42)-C(40)-C(34)	109.55(13)	C(42)-C(40)-C(41)	108.75(14)
C(42)-C(40)-C(43)	109.66(14)	C(40)-C(41)-H(41A)	109.5
C(40)-C(41)-H(41B)	109.5	C(40)-C(41)-H(41C)	109.5
H(41A)-C(41)-H(41B)	109.5	H(41A)-C(41)-H(41C)	109.5
H(41B)-C(41)-H(41C)	109.5	C(40)-C(42)-H(42A)	109.5
C(40)-C(42)-H(42B)	109.5	C(40)-C(42)-H(42C)	109.5
H(42A)-C(42)-H(42B)	109.5	H(42A)-C(42)-H(42C)	109.5
H(42B)-C(42)-H(42C)	109.5	C(40)-C(43)-H(43A)	109.5
C(40)-C(43)-H(43B)	109.5	C(40)-C(43)-H(43C)	109.5
H(43A)-C(43)-H(43B)	109.5	H(43A)-C(43)-H(43C)	109.5
H(43B)-C(43)-H(43C)	109.5	N(1)-C(44)-H(44A)	108.7
N(1)-C(44)-H(44B)	108.7	H(44A)-C(44)-H(44B)	107.6
C(45)-C(44)-N(1)	114.12(10)	C(45)-C(44)-H(44A)	108.7
C(45)-C(44)-H(44B)	108.7	C(46)-C(45)-C(44)	118.93(12)
C(50)-C(45)-C(44)	119.77(12)	C(50)-C(45)-C(46)	121.29(12)
O(4)-C(46)-C(45)	119.01(11)	O(4)-C(46)-C(47)	122.01(12)
C(45)-C(46)-C(47)	118.97(12)	C(46)-C(47)-C(51)	120.89(12)
C(48)-C(47)-C(46)	117.50(12)	C(48)-C(47)-C(51)	121.59(12)
C(47)-C(48)-H(48)	117.9	C(47)-C(48)-C(49)	124.17(12)
C(49)-C(48)-H(48)	117.9	C(48)-C(49)-C(55)	120.73(12)
C(50)-C(49)-C(48)	116.64(12)	C(50)-C(49)-C(55)	122.64(12)
C(45)-C(50)-H(50)	119.3	C(49)-C(50)-C(45)	121.39(12)
C(49)-C(50)-H(50)	119.3	C(47)-C(51)-C(52)	108.69(12)
C(47)-C(51)-C(53)	110.80(12)	C(47)-C(51)-C(54)	112.56(12)
C(52)-C(51)-C(53)	109.79(15)	C(52)-C(51)-C(54)	107.91(12)
C(54)-C(51)-C(53)	107.03(13)	C(51)-C(52)-H(52A)	109.5
C(51)-C(52)-H(52B)	109.5	C(51)-C(52)-H(52C)	109.5
H(52A)-C(52)-H(52B)	109.5	H(52A)-C(52)-H(52C)	109.5
H(52B)-C(52)-H(52C)	109.5	C(51)-C(53)-H(53A)	109.5
C(51)-C(53)-H(53B)	109.5	C(51)-C(53)-H(53C)	109.5
H(53A)-C(53)-H(53B)	109.5	H(53A)-C(53)-H(53C)	109.5
H(53B)-C(53)-H(53C)	109.5	C(51)-C(54)-H(54A)	109.5

C(51)-C(54)-H(54B)	109.5	C(51)-C(54)-H(54C)	109.5
H(54A)-C(54)-H(54B)	109.5	H(54A)-C(54)-H(54C)	109.5
H(54B)-C(54)-H(54C)	109.5	C(49)-C(55)-C(56)	109.61(12)
C(57)-C(55)-C(49)	111.84(11)	C(57)-C(55)-C(56)	108.00(13)
C(58)-C(55)-C(49)	110.10(12)	C(58)-C(55)-C(56)	109.35(12)
C(58)-C(55)-C(57)	107.87(13)	C(55)-C(56)-H(56A)	109.5
C(55)-C(56)-H(56B)	109.5	C(55)-C(56)-H(56C)	109.5
H(56A)-C(56)-H(56B)	109.5	H(56A)-C(56)-H(56C)	109.5
H(56B)-C(56)-H(56C)	109.5	C(55)-C(57)-H(57A)	109.5
C(55)-C(57)-H(57B)	109.5	C(55)-C(57)-H(57C)	109.5
H(57A)-C(57)-H(57B)	109.5	H(57A)-C(57)-H(57C)	109.5
H(57B)-C(57)-H(57C)	109.5	C(55)-C(58)-H(58A)	109.5
C(55)-C(58)-H(58B)	109.5	C(55)-C(58)-H(58C)	109.5
H(58A)-C(58)-H(58B)	109.5	H(58A)-C(58)-H(58C)	109.5
H(58B)-C(58)-H(58C)	109.5	C(60A)-C(59A)-H(59A)	120.1
C(64A)-C(59A)-H(59A)	120.1	C(64A)-C(59A)-C(60A)	119.82(19)
C(59A)-C(60A)-H(60A)	120.0	C(59A)-C(60A)-C(61A)	120.1(2)
C(61A)-C(60A)-H(60A)	120.0	C(60A)-C(61A)-H(61A)	120.1
C(62A)-C(61A)-C(60A)	119.9(2)	C(62A)-C(61A)-H(61A)	120.1
C(61A)-C(62A)-H(62A)	119.8	C(61A)-C(62A)-C(63A)	120.3(2)
C(63A)-C(62A)-H(62A)	119.8	C(62A)-C(63A)-H(63A)	120.2
C(64A)-C(63A)-C(62A)	119.6(2)	C(64A)-C(63A)-H(63A)	120.2
C(59A)-C(64A)-C(63A)	120.25(19)	C(59A)-C(64A)-H(64A)	119.9
C(63A)-C(64A)-H(64A)	119.9	C(59B)-C(64B)-H(64B)	120.0
C(59B)-C(64B)-C(63B)	120.0	C(63B)-C(64B)-H(64B)	120.0
C(64B)-C(59B)-H(59B)	120.0	C(64B)-C(59B)-C(60B)	120.0
C(60B)-C(59B)-H(59B)	120.0	C(59B)-C(60B)-H(60B)	120.0
C(61B)-C(60B)-C(59B)	120.0	C(61B)-C(60B)-H(60B)	120.0
C(60B)-C(61B)-H(61B)	120.0	C(60B)-C(61B)-C(62B)	120.0
C(62B)-C(61B)-H(61B)	120.0	C(61B)-C(62B)-H(62B)	120.0
C(63B)-C(62B)-C(61B)	120.0	C(63B)-C(62B)-H(62B)	120.0
C(64B)-C(63B)-H(63B)	120.0	C(62B)-C(63B)-C(64B)	120.0
C(62B)-C(63B)-H(63B)	120.0		

Single crystal structure analysis of 14b · cyclohexane solvate

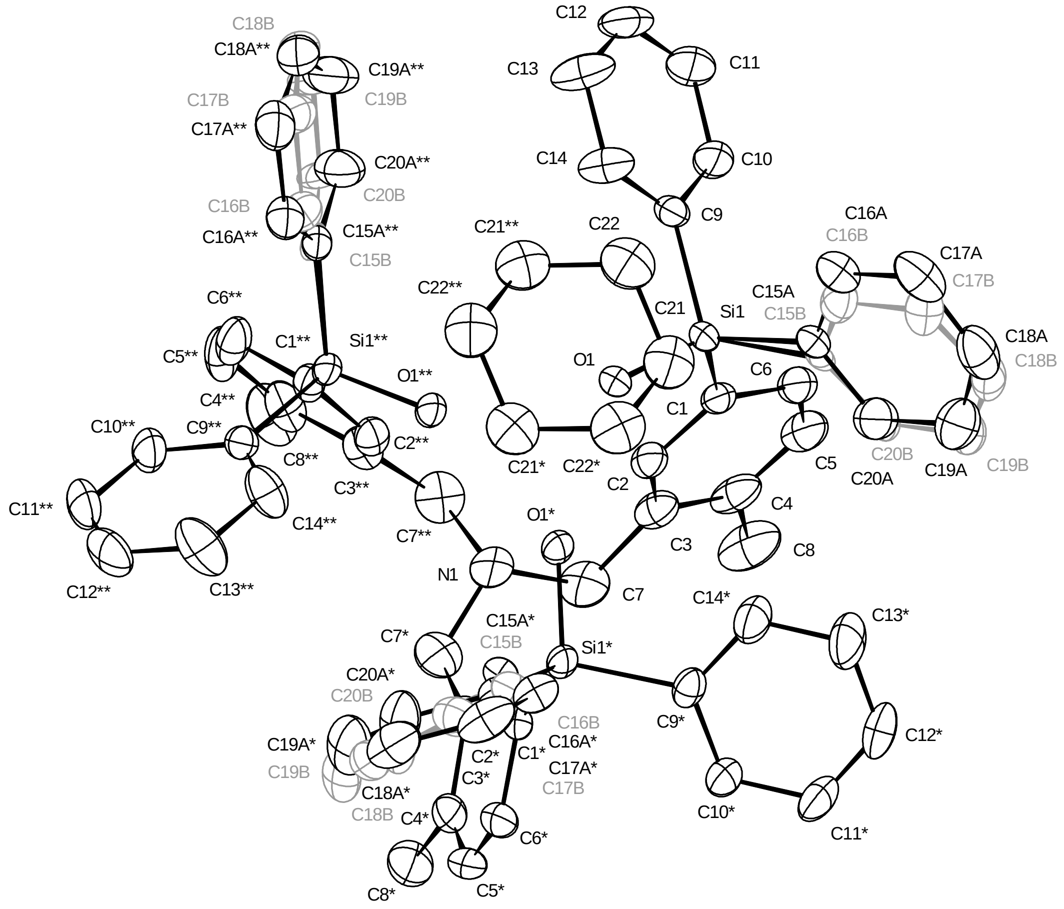

Figure S6. The molecular structure of **14b · cyclohexane solvate**. H atoms have been removed for clarity. Main structure shown in black and disordered parts shown in grey.

X-ray Crystal Structure Analysis of 14b · cyclohexane solvate:

C_{66} H_{69} N O_3 Si_3, M_r = 1008.49 g mol^{-1}, colourless plate, crystal size 0.17 x 0.12 x 0.05 mm^3, trigonal, space group R-3 [148], a = 14.2662(11) Å, c = 49.894(2) Å, V = 8794.1(14) Å^3, T = 100(2) K, Z = 6, D_{calc} = 1.143 g·cm^3, λ = 0.71073 Å, $\mu(Mo\text{-}K_\alpha)$ = 0.436 mm^{-1}, Gaussian absorption correction ($T_{\min}$ = 0.95533, $T_{\max}$ = 0.99219), Bruker AXS Enraf-Nonius KappaCCD diffractometer with a FR591 rotating Mo-anode X-ray source, 3.300 < θ < 30.503°, 67174 measured reflections, 5982 independent reflections, 4120 reflections with $I > 2\sigma(I)$, R_{int} = 0.0744. The structure was solved by *SHELXS* and refined by full-matrix least-squares (*SHELXL*) against F^2 to R_1 = 0. 0.0587 [$I > 2\sigma(I)$], wR_2 = 0.1356 [all data], 277 parameters and 72 restraints.

Full .cif data for the compound are available under the CCDC number **CCDC 2293659**

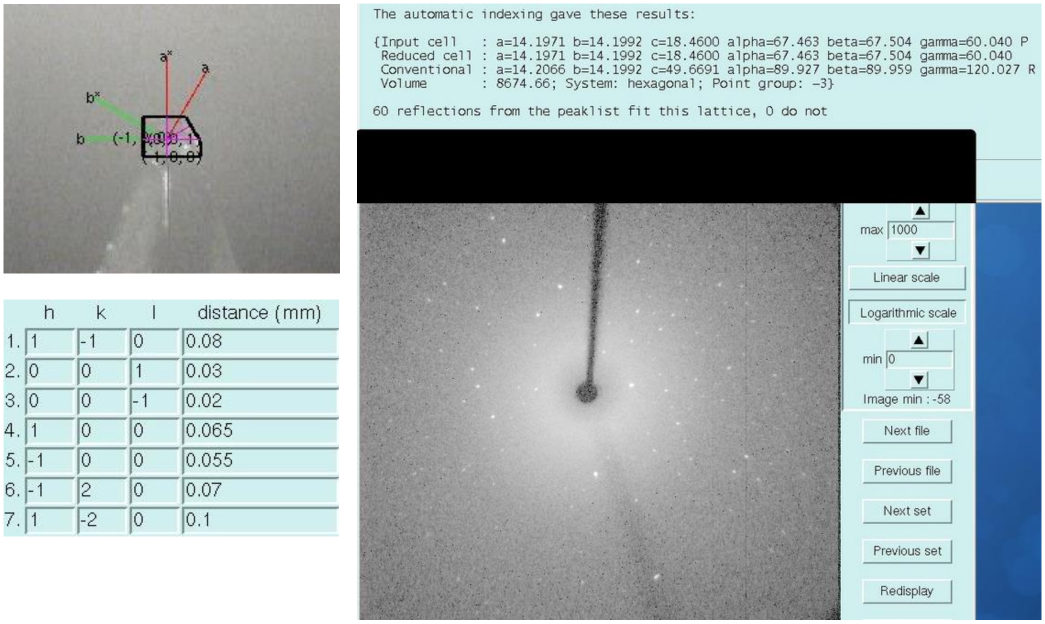

Figure S7. Crystal faces and unit cell determination/refinement of compound **14b · cyclohexane solvate**.

INTENSITY STATISTICS FOR DATASET

Resolution	#Data	#Theory	%Complete	Redundancy	Mean I	Mean I/s	Rmerge	Rsigma
Inf - 2.69	102	110	92.7	17.08	103.14	100.46	0.0299	0.0079
2.69 - 1.80	241	241	100.0	19.36	32.34	72.92	0.0324	0.0090
1.80 - 1.44	338	338	100.0	18.68	12.95	48.12	0.0457	0.0140
1.44 - 1.26	344	344	100.0	16.72	11.97	38.68	0.0487	0.0174
1.26 - 1.14	360	360	100.0	15.32	9.83	30.96	0.0606	0.0223
1.14 - 1.06	333	333	100.0	14.14	5.65	20.41	0.0906	0.0353
1.06 - 1.00	342	342	100.0	13.16	3.82	14.37	0.1285	0.0516
1.00 - 0.95	326	326	100.0	12.33	4.04	13.84	0.1312	0.0547
0.95 - 0.91	340	340	100.0	11.80	3.02	10.76	0.1663	0.0731
0.91 - 0.87	392	392	100.0	11.13	2.82	9.74	0.1923	0.0847
0.87 - 0.84	328	328	100.0	10.74	2.17	7.47	0.2449	0.1121
0.84 - 0.82	283	283	100.0	9.51	1.78	5.84	0.3133	0.1489
0.82 - 0.79	428	428	100.0	7.97	1.78	4.97	0.3046	0.1685
0.79 - 0.77	341	341	100.0	6.90	1.63	4.13	0.3257	0.2031
0.77 - 0.75	363	363	100.0	6.75	1.18	3.01	0.4489	0.2897
0.75 - 0.73	407	407	100.0	6.39	1.10	2.65	0.4601	0.3342
0.73 - 0.72	223	223	100.0	6.44	0.85	2.04	0.5499	0.4384
0.72 - 0.70	494	494	100.0	6.12	0.82	1.90	0.5931	0.4861
0.70 - 0.69	260	260	100.0	6.01	0.78	1.70	0.6305	0.5442
0.69 - 0.68	265	265	100.0	5.88	0.82	1.75	0.6201	0.5357
0.68 - 0.67	275	276	99.6	5.50	0.73	1.43	0.6814	0.6534
0.77 - 0.67	2287	2288	100.0	6.18	0.91	2.13	0.5459	0.4358
Inf - 0.67	6785	6794	99.9	10.57	6.13	15.44	0.0726	0.0492

A resolution cut off (SHEL 999 0.7) was applied to exclude poorly determined reflections at high diffraction angles. Three reflections (OMIT 0 1 5; OMIT -2 2 2; OMIT 0 1 8) with high I\σ(I) were excluded before the final refinement cycles. One terminal phenyl ring shows a disorder over two positions. It was refined using a free variable (FVAR) and occupancies of 65.747:34.253% were

determined. The ISOR (0.01 0.02) instruction was used equalise the thermal ellipsoids of the disordered parts. In addition, the structure contains a second cyclohexane molecule that is located on a crystallographic special position. Due to the disorder over several positions, a proper refinement was not suitable. Therefore, a solvent mask (SQUEEZE routine in Olex2) was applied to exclude the remaining electron density. This procedure resulted in a structure model with a void volume of 447.58 Å³, corresponding to 5.1% of the unit cell volume (calculated with Mercury 2021.2.0 program, probe radius of 1.2 Å and an approximate grid spacing of 0.7 Å).

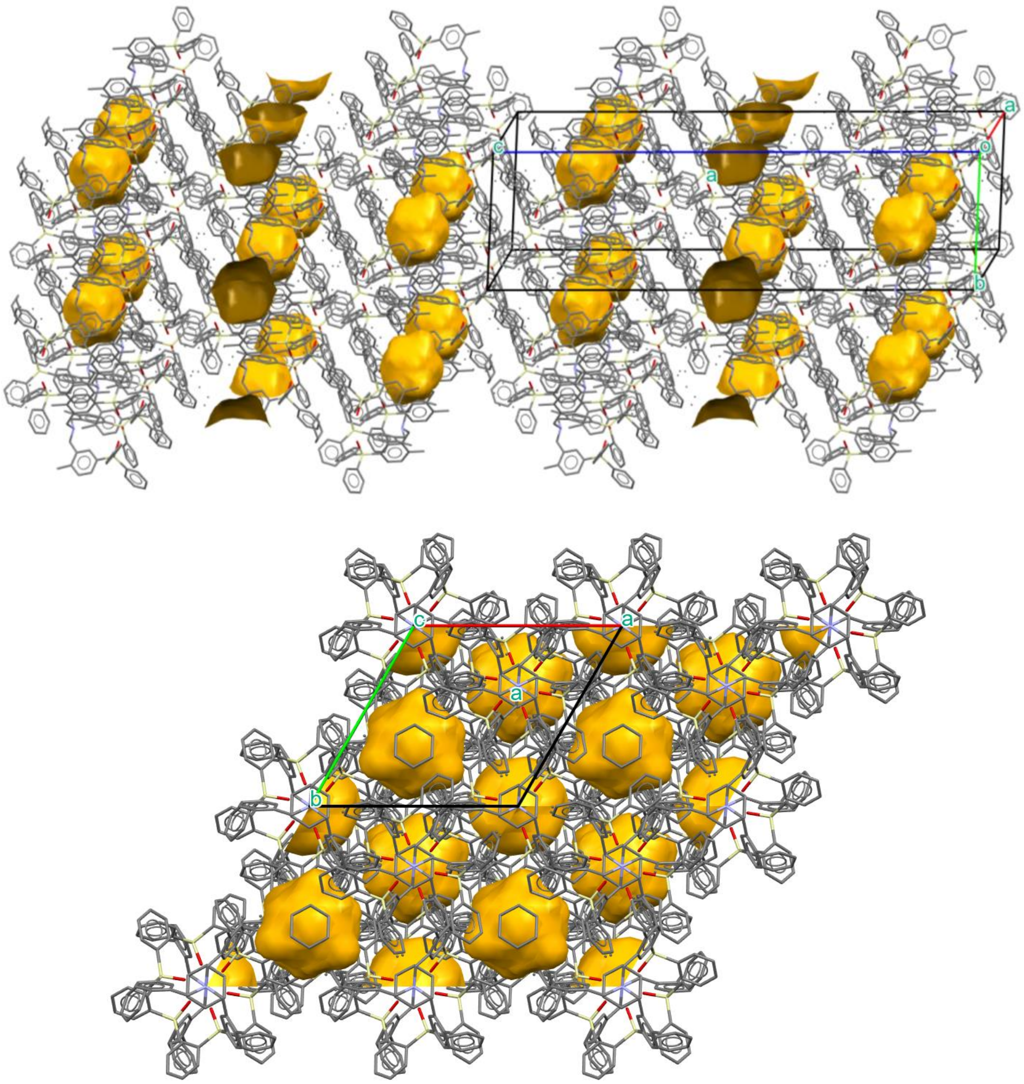

Figure S8. Solvent accessible voids (orange brown surface) in the structure of **14b · cyclohexane solvate** in a random orientation (top) and along the crystallographic c axis (bottom).

Table S5. Crystal data and structure refinement of compound **14b · cyclohexane solvate**.

Identification code	10535	
Empirical formula	$C_{66} H_{69} N O_3 Si_3$	
Color	colorless	
Formula weight	1008.49 g·mol^{-1}	
Temperature	100(2) K	
Wavelength	0.71073 Å	
Crystal system	Trigonal	
Space group	*R*-3, (no. 148)	
Unit cell dimensions	a = 14.2662(11) Å	α= 90°.
	b = 14.2662(11) Å	β= 90°.
	c = 49.894(2) Å	γ = 120°.
Volume	8794.1(14) Å^3	
Z	6	
Density (calculated)	1.143 Mg·m^{-3}	
Absorption coefficient	0.126 mm^{-1}	
F(000)	3228 e	
Crystal size	0.17 x 0.12 x 0.05 mm^3	
θ range for data collection	3.300 to 30.503°.	
Index ranges	$-19 \le h \le 20$, $-20\le k \le 20$, $-71 \le l \le 71$	
Reflections collected	67174	
Independent reflections	5982 [R_{int} = 0.0744]	
Reflections with I>2σ(I)	4120	
Completeness to θ = 25.242°	99.7 %	
Absorption correction	Gaussian	
Max. and min. transmission	0.99412 and 0.98250	
Refinement method	Full-matrix least-squares on F^2	
Data / restraints / parameters	5982 / 72 / 277	
Goodness-of-fit on F^2	1.035	
Final R indices [I>2σ(I)]	R_1 = 0.0587	wR^2 = 0.1200
R indices (all data)	R_1 = 0.0954	wR^2 = 0.1356
Extinction coefficient	n/a	
Largest diff. peak and hole	0.352 and -0.378 e·Å^{-3}	

Table S6. Bond lengths [Å] and angles [°] of compound **14b · cyclohexane solvate**.

Si(1)-O(1)	1.6388(12)	Si(1)-C(1)	1.8547(19)
Si(1)-C(9)	1.8571(17)	Si(1)-C(15A)	1.896(8)
Si(1)-C(15B)	1.836(18)	O(1)-H(1)	0.8400
N(1)-C(7)#1	1.462(2)	N(1)-C(7)	1.462(2)
N(1)-C(7)#2	1.462(2)	C(1)-C(2)	1.396(2)
C(1)-C(6)	1.398(2)	C(2)-H(2)	0.9500
C(2)-C(3)	1.393(2)	C(3)-C(4)	1.405(3)
C(3)-C(7)	1.514(3)	C(4)-C(5)	1.392(3)
C(4)-C(8)	1.509(3)	C(5)-H(5)	0.9500
C(5)-C(6)	1.378(3)	C(6)-H(6)	0.9500
C(7)-H(7A)	0.9900	C(7)-H(7B)	0.9900
C(8)-H(8A)	0.9800	C(8)-H(8B)	0.9800
C(8)-H(8C)	0.9800	C(9)-C(10)	1.384(3)
C(9)-C(14)	1.384(3)	C(10)-H(10)	0.9500
C(10)-C(11)	1.384(3)	C(11)-H(11)	0.9500
C(11)-C(12)	1.364(3)	C(12)-H(12)	0.9500
C(12)-C(13)	1.369(3)	C(13)-H(13)	0.9500
C(13)-C(14)	1.389(3)	C(14)-H(14)	0.9500
C(15A)-C(16A)	1.378(8)	C(15A)-C(20A)	1.410(10)
C(15B)-C(16B)	1.387(15)	C(15B)-C(20B)	1.345(17)
C(16A)-H(16A)	0.9500	C(16A)-C(17A)	1.373(12)
C(16B)-H(16B)	0.9500	C(16B)-C(17B)	1.43(2)
C(17A)-H(17A)	0.9500	C(17A)-C(18A)	1.361(9)
C(17B)-H(17B)	0.9500	C(17B)-C(18B)	1.385(15)
C(18A)-H(18A)	0.9500	C(18A)-C(19A)	1.362(9)
C(18B)-H(18B)	0.9500	C(18B)-C(19B)	1.377(15)
C(19A)-H(19A)	0.9500	C(19A)-C(20A)	1.385(9)
C(19B)-H(19B)	0.9500	C(19B)-C(20B)	1.399(15)
C(20A)-H(20A)	0.9500	C(20B)-H(20B)	0.9500
C(21)-H(21A)	0.9900	C(21)-H(21B)	0.9900
C(21)-C(22)	1.521(3)	C(21)-C(22)#2	1.518(3)
C(22)-H(22A)	0.9900	C(22)-H(22B)	0.9900
O(1)-Si(1)-C(1)	110.13(7)	O(1)-Si(1)-C(9)	104.74(7)

O(1)-Si(1)-C(15A)	108.7(3)	O(1)-Si(1)-C(15B)	114.7(5)
C(1)-Si(1)-C(9)	110.68(8)	C(1)-Si(1)-C(15A)	113.5(3)
C(9)-Si(1)-C(15A)	108.7(3)	C(15B)-Si(1)-C(1)	101.1(4)
C(15B)-Si(1)-C(9)	115.6(5)	Si(1)-O(1)-H(1)	109.5
C(7)#1-N(1)-C(7)	110.82(12)	C(7)#2-N(1)-C(7)	110.82(12)
C(7)#1-N(1)-C(7)#2	110.82(12)	C(2)-C(1)-Si(1)	120.41(12)
C(2)-C(1)-C(6)	117.15(17)	C(6)-C(1)-Si(1)	122.44(15)
C(1)-C(2)-H(2)	118.4	C(3)-C(2)-C(1)	123.11(16)
C(3)-C(2)-H(2)	118.4	C(2)-C(3)-C(4)	118.47(18)
C(2)-C(3)-C(7)	119.88(16)	C(4)-C(3)-C(7)	121.47(17)
C(3)-C(4)-C(8)	121.5(2)	C(5)-C(4)-C(3)	118.67(18)
C(5)-C(4)-C(8)	119.77(19)	C(4)-C(5)-H(5)	119.0
C(6)-C(5)-C(4)	121.94(17)	C(6)-C(5)-H(5)	119.0
C(1)-C(6)-H(6)	119.7	C(5)-C(6)-C(1)	120.61(19)
C(5)-C(6)-H(6)	119.7	N(1)-C(7)-C(3)	113.95(16)
N(1)-C(7)-H(7A)	108.8	N(1)-C(7)-H(7B)	108.8
C(3)-C(7)-H(7A)	108.8	C(3)-C(7)-H(7B)	108.8
H(7A)-C(7)-H(7B)	107.7	C(4)-C(8)-H(8A)	109.5
C(4)-C(8)-H(8B)	109.5	C(4)-C(8)-H(8C)	109.5
H(8A)-C(8)-H(8B)	109.5	H(8A)-C(8)-H(8C)	109.5
H(8B)-C(8)-H(8C)	109.5	C(10)-C(9)-Si(1)	120.51(14)
C(14)-C(9)-Si(1)	122.39(14)	C(14)-C(9)-C(10)	117.10(16)
C(9)-C(10)-H(10)	119.1	C(11)-C(10)-C(9)	121.8(2)
C(11)-C(10)-H(10)	119.1	C(10)-C(11)-H(11)	119.9
C(12)-C(11)-C(10)	120.2(2)	C(12)-C(11)-H(11)	119.9
C(11)-C(12)-H(12)	120.3	C(11)-C(12)-C(13)	119.39(18)
C(13)-C(12)-H(12)	120.3	C(12)-C(13)-H(13)	119.8
C(12)-C(13)-C(14)	120.5(2)	C(14)-C(13)-H(13)	119.8
C(9)-C(14)-C(13)	121.06(19)	C(9)-C(14)-H(14)	119.5
C(13)-C(14)-H(14)	119.5	C(16A)-C(15A)-Si(1)	123.8(6)
C(16A)-C(15A)-C(20A)	116.1(7)	C(20A)-C(15A)-Si(1)	120.0(5)
C(16B)-C(15B)-Si(1)	114.5(11)	C(20B)-C(15B)-Si(1)	127.9(10)
C(20B)-C(15B)-C(16B)	117.4(15)	C(15A)-C(16A)-H(16A)	119.0
C(17A)-C(16A)-C(15A)	122.0(7)	C(17A)-C(16A)-H(16A)	119.0
C(15B)-C(16B)-H(16B)	118.8	C(15B)-C(16B)-C(17B)	122.4(13)
C(17B)-C(16B)-H(16B)	118.8	C(16A)-C(17A)-H(17A)	120.0

C(18A)-C(17A)-C(16A)	120.0(6)	C(18A)-C(17A)-H(17A)	120.0
C(16B)-C(17B)-H(17B)	121.1	C(18B)-C(17B)-C(16B)	117.8(12)
C(18B)-C(17B)-H(17B)	121.1	C(17A)-C(18A)-H(18A)	119.4
C(17A)-C(18A)-C(19A)	121.1(7)	C(19A)-C(18A)-H(18A)	119.4
C(17B)-C(18B)-H(18B)	120.4	C(19B)-C(18B)-C(17B)	119.2(13)
C(19B)-C(18B)-H(18B)	120.4	C(18A)-C(19A)-H(19A)	120.7
C(18A)-C(19A)-C(20A)	118.7(7)	C(20A)-C(19A)-H(19A)	120.7
C(18B)-C(19B)-H(19B)	119.4	C(18B)-C(19B)-C(20B)	121.2(11)
C(20B)-C(19B)-H(19B)	119.4	C(15A)-C(20A)-H(20A)	119.0
C(19A)-C(20A)-C(15A)	122.0(6)	C(19A)-C(20A)-H(20A)	119.0
C(15B)-C(20B)-C(19B)	122.0(11)	C(15B)-C(20B)-H(20B)	119.0
C(19B)-C(20B)-H(20B)	119.0	H(21A)-C(21)-H(21B)	108.0
C(22)#2-C(21)-H(21A)	109.3	C(22)-C(21)-H(21A)	109.3
C(22)-C(21)-H(21B)	109.3	C(22)#2-C(21)-H(21B)	109.3
C(22)#2-C(21)-C(22)	111.6(2)	C(21)#1-C(22)-C(21)	111.0(2)
C(21)#1-C(22)-H(22A)	109.4	C(21)-C(22)-H(22A)	109.4
C(21)-C(22)-H(22B)	109.4	C(21)#1-C(22)-H(22B)	109.4
H(22A)-C(22)-H(22B)	108.0		

Symmetry transformations used to generate equivalent atoms:
#1 -y+1,x-y,z   #2 -x+y+1,-x+1,z

Single crystal structure analysis of 15a · dichloromethane solvate

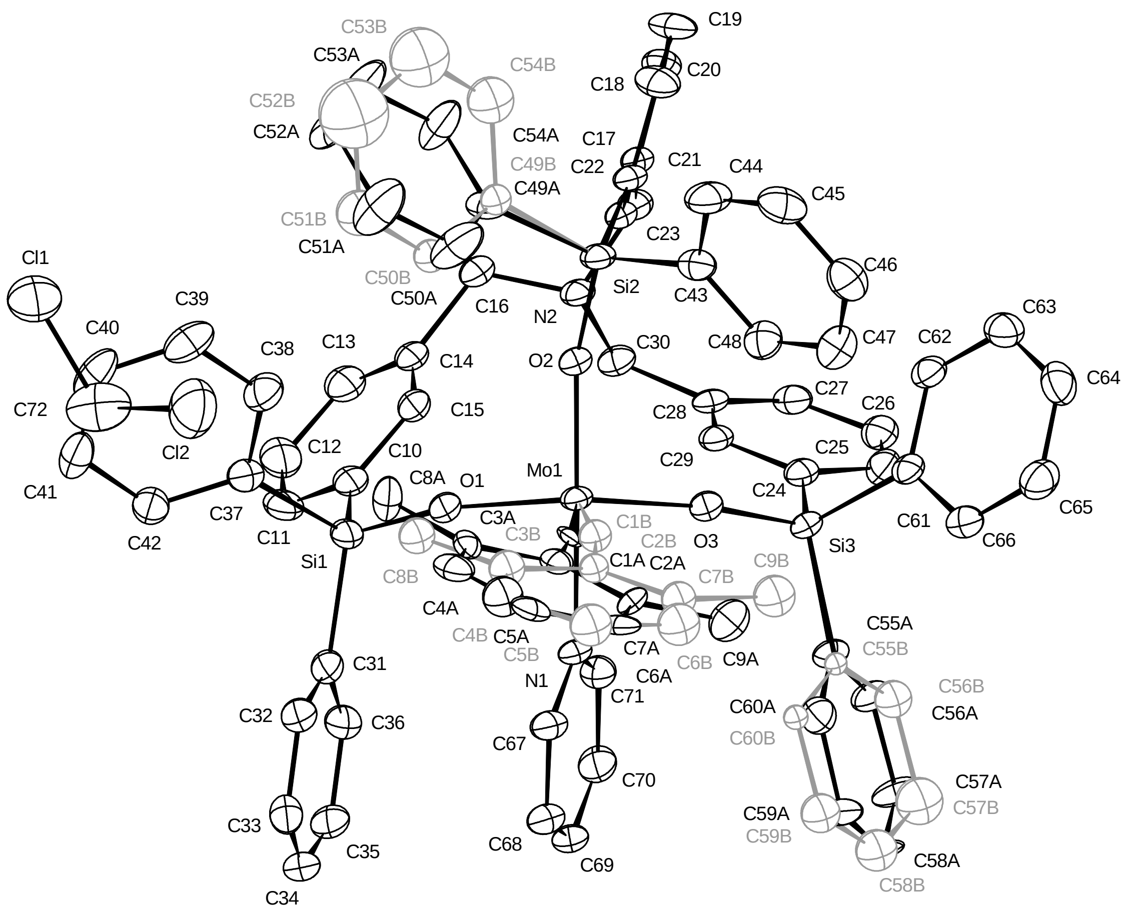

Figure S9. The molecular structure of **15a · dichloromethane solvate**. H atoms have been removed for clarity. Main structure shown in black and disordered parts shown in grey.

X-ray Crystal Structure Analysis of 15a · dichloromethane solvate:
C_{72} H_{64} Cl_2 Mo N_2 O_3 Si_3, M_r = 1256.36 g mol^{-1}, violet plate, crystal size 0.153 x 0.05 x 0.007 mm^3, monoclinic, space group $P2_1/c$ [14], a = 20.887(3) Å, b = 12.7056(18) Å, c = 26.768(4) Å, β = 107.864(3)°, V = 6761.2(16) Å^3, T = 100(2) K, Z = 4, D_{calc} = 1.234 g·cm^3, λ = 0.71073 Å, μ(Mo-K_α) = 0.372 mm^{-1}, Gaussian absorption correction ($T_{\min}$ = 0.97410, $T_{\max}$ = 0.99858), Bruker-AXS Kappa Mach3 with APEX-II detector and IµS microfocus Mo-anode X-ray source, 1.599 < θ < 26.732°, 143560 measured reflections, 14302 independent reflections, 9833 reflections with $I > 2\sigma(I)$, R_{int} = 0.0972. The structure was solved by *SHELXT* and refined by full-matrix least-squares (*SHELXL*) against F^2 to R_1 = 0.0493 [$I > 2\sigma(I)$], wR_2 = 0.1216 [all data], 812 parameters and 36 restraints.

Full .cif data for the compound are available under the CCDC number **CCDC 2293661**

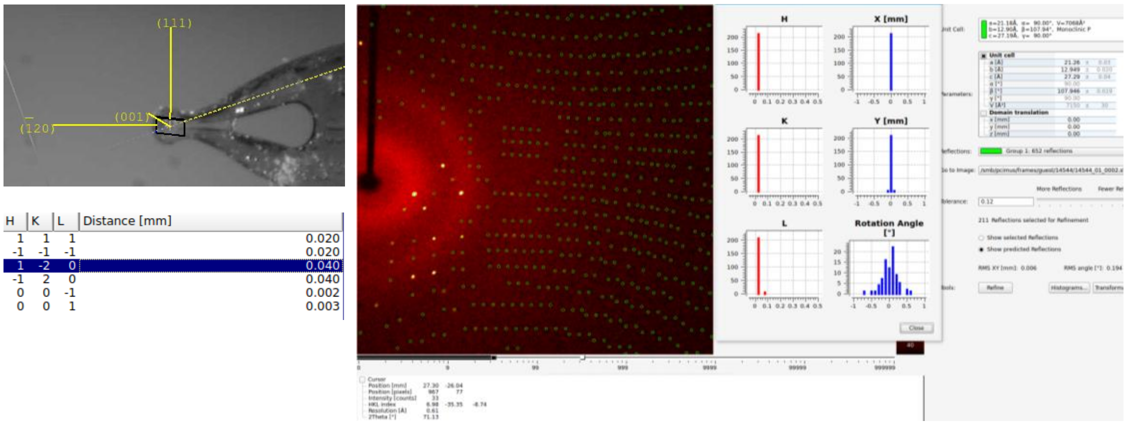

Figure S10. Crystal faces and unit cell determination/refinement of compound **15a · dichloromethane solvate**.

INTENSITY STATISTICS FOR DATASET

Resolution	#Data	#Theory	%Complete	Redundancy	Mean I	Mean I/s	Rmerge	Rsigma
Inf - 3.31	232	233	99.6	13.52	74.18	47.72	0.0252	0.0142
3.31 - 2.17	554	554	100.0	14.98	33.88	40.71	0.0348	0.0164
2.17 - 1.72	754	754	100.0	15.30	24.54	36.42	0.0484	0.0193
1.72 - 1.49	787	787	100.0	15.37	17.90	31.19	0.0600	0.0225
1.49 - 1.35	786	786	100.0	15.39	13.70	26.79	0.0762	0.0273
1.35 - 1.25	780	780	100.0	15.20	13.08	24.25	0.0861	0.0299
1.25 - 1.18	728	728	100.0	14.88	10.22	19.49	0.1069	0.0373
1.18 - 1.11	908	908	100.0	14.23	8.40	16.68	0.1256	0.0458
1.11 - 1.06	766	766	100.0	11.32	8.14	13.58	0.1390	0.0595
1.06 - 1.02	784	784	100.0	9.50	6.24	9.70	0.1777	0.0891
1.02 - 0.99	641	641	100.0	8.46	5.56	8.08	0.2062	0.1082
0.99 - 0.96	750	750	100.0	7.71	5.34	7.28	0.2136	0.1218
0.96 - 0.93	831	831	100.0	6.96	4.55	6.04	0.2446	0.1522
0.93 - 0.90	920	920	100.0	6.28	4.36	5.19	0.2713	0.1728
0.90 - 0.88	719	719	100.0	5.82	3.64	4.19	0.3111	0.2164
0.88 - 0.86	770	770	100.0	5.62	3.51	3.97	0.3286	0.2318
0.86 - 0.84	823	823	100.0	5.54	3.26	3.62	0.3582	0.2561
0.84 - 0.82	926	926	100.0	5.33	3.08	3.28	0.3756	0.2843
0.82 - 0.81	506	506	100.0	5.37	2.81	2.93	0.4152	0.3108
0.81 - 0.79	1017	1060	95.9	4.64	2.11	2.13	0.4837	0.4491
0.79 - 0.78	422	677	62.3	1.45	2.28	1.48	0.4529	0.6476
0.88 - 0.78	4464	4762	93.7	4.71	2.86	2.99	0.3867	0.3253
Inf - 0.78	15404	15703	98.1	9.44	9.51	13.62	0.0916	0.0691

The crystal investigated showed only moderate scattering intensities even with higher exposure times per frame. A resolution cut off (SHEL 999 0.79) was applied to exclude poorly determined intensities at higher diffraction angles. Eight reflections (OMIT 1 0 0; 22 7 2; 1 0 4; -1 0 4; -1 0 6; 3 1 3; -4 2 3; -4 2 10) with high I\σ(I) were excluded before the final refinement cycles. The structure shows some disordered parts. Two of the phenyl rings connected to Si2 and Si3 are disordered over two positions. Both aryl rings were described with fixed occupancies of 70:30%. An AFIX instruction was applied to force one of the aryl rings into a hexagonal shape. Furthermore,

the alkylidyne ligand attached to the Mo central atom shows a twofold positional disorder. This was described with fixed atomic occupancies of 60:40%. Isotropic displacement parameters were applied for minor parts of the disordered subunits. The ISOR (0.008 0.02) instruction was used equalise the thermal ellipsoids of six carbon atoms in the disordered parts.

In addition, the structure contains a second solute molecule (mixture of DCM and pentane) which is located on a crystallographic special position. Due to the disorder over several positions, a proper refinement was not suitable. Therefore, a solvent mask (SQUEEZE routine in Olex2) was applied to exclude the remaining electron density. This procedure resulted in a structure model with a void volume of 719.01 Å³, corresponding to 10.6% of the unit cell volume (calculated with Mercury 2021.2.0 program, probe radius of 1.2 Å and an approximate grid spacing of 0.7 Å).

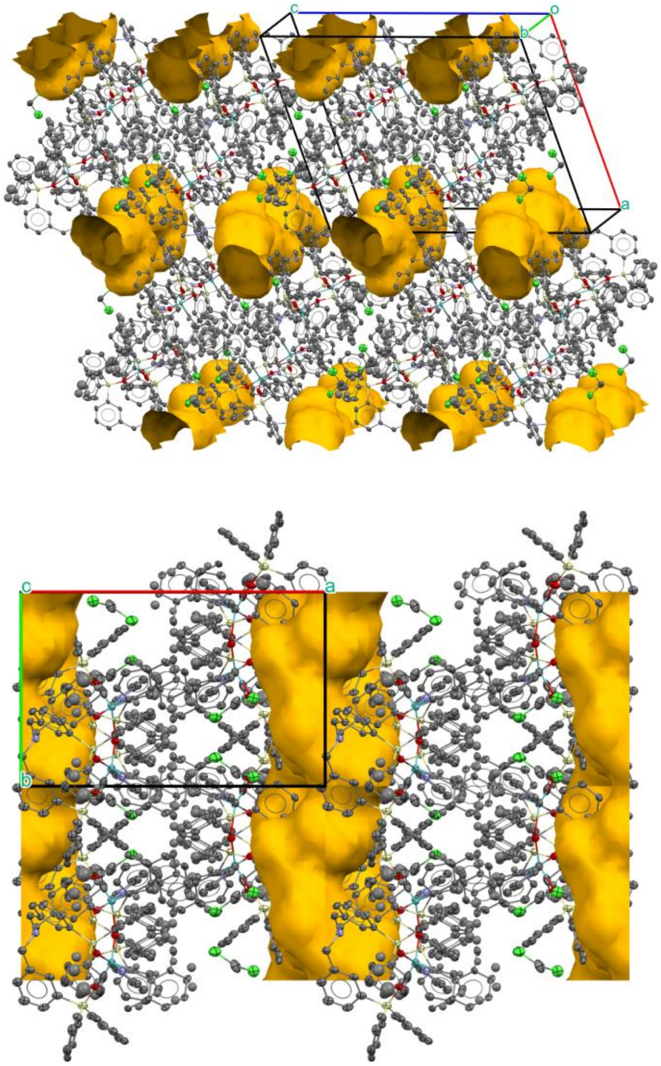

Figure S11. Solvent accessible voids (orange brown surface) in the structure of **15a · dichloromethane solvate** in a random orientation (top) and along the crystallographic c axis (bottom).

Table S7. Crystal data and structure refinement of compound **15a · dichloromethane solvate**.

Identification code	14544	
Empirical formula	C_{72} H_{64} Cl_2 Mo N_2 O_3 Si_3	
Color	violet	
Formula weight	1256.36 $g·mol^{-1}$	
Temperature	100(2) K	
Wavelength	0.71073 Å	
Crystal system	Monoclinic	
Space group	$P2_1/c$, (no. 14)	
Unit cell dimensions	a = 20.887(3) Å	α= 90°.
	b = 12.7056(18) Å	β= 107.864(3)°.
	c = 26.768(4) Å	γ = 90°.
Volume	6761.2(16) $Å^3$	
Z	4	
Density (calculated)	1.234 $Mg·m^{-3}$	
Absorption coefficient	0.372 mm^{-1}	
F(000)	2608 e	
Crystal size	0.153 x 0.05 x 0.007 mm^3	
θ range for data collection	1.599 to 26.732°.	
Index ranges	$-25 \le h \le 26$, $-16\le k \le 16$, $-33 \le l \le 33$	
Reflections collected	143560	
Independent reflections	14302 [R_{int} = 0.0972]	
Reflections with $I>2\sigma(I)$	9833	
Completeness to θ = 25.242°	99.9 %	
Absorption correction	Gaussian	
Max. and min. transmission	0.99858 and 0.97410	
Refinement method	Full-matrix least-squares on F^2	
Data / restraints / parameters	14302 / 36 / 812	
Goodness-of-fit on F^2	1.021	
Final R indices [$I>2\sigma(I)$]	R_1 = 0.0493	wR^2 = 0.1069
R indices (all data)	R_1 = 0.0874	wR^2 = 0.1216
Extinction coefficient	n/a	
Largest diff. peak and hole	0.453 and -1.027 $e·Å^{-3}$	

Table S8. Bond lengths [Å] and angles [°] of compound **15a · dichloromethane solvate**.

Mo(1)-O(1)	1.916(2)	Mo(1)-O(2)	1.923(2)
Mo(1)-O(3)	1.929(2)	Mo(1)-N(1)	2.265(2)
Mo(1)-C(1A)	1.705(10)	Mo(1)-C(1B)	1.766(17)
Si(1)-O(1)	1.617(2)	Si(1)-C(10)	1.872(4)
Si(1)-C(31)	1.876(3)	Si(1)-C(37)	1.870(3)
Si(2)-O(2)	1.618(2)	Si(2)-C(17)	1.867(3)
Si(2)-C(43)	1.876(3)	Si(2)-C(49B)	1.874(7)
Si(2)-C(49A)	1.913(3)	Si(3)-O(3)	1.615(2)
Si(3)-C(24)	1.877(3)	Si(3)-C(55A)	1.833(10)
Si(3)-C(55B)	1.98(3)	Si(3)-C(61)	1.876(3)
N(1)-C(67)	1.343(4)	N(1)-C(71)	1.341(4)
N(2)-C(16)	1.459(4)	N(2)-C(23)	1.467(4)
N(2)-C(30)	1.462(4)	C(1A)-C(2A)	1.438(12)
C(1B)-C(2B)	1.51(2)	C(2A)-C(3A)	1.399(10)
C(2A)-C(7A)	1.407(9)	C(2B)-C(3B)	1.415(14)
C(2B)-C(7B)	1.398(14)	C(3A)-C(4A)	1.393(8)
C(3A)-C(8A)	1.507(8)	C(3B)-C(4B)	1.377(16)
C(3B)-C(8B)	1.490(14)	C(4A)-H(4A)	0.9500
C(4A)-C(5A)	1.403(10)	C(4B)-H(4B)	0.9500
C(4B)-C(5B)	1.419(17)	C(5A)-H(5A)	0.9500
C(5A)-C(6A)	1.375(10)	C(5B)-H(5B)	0.9500
C(5B)-C(6B)	1.405(15)	C(6A)-H(6A)	0.9500
C(6A)-C(7A)	1.387(9)	C(6B)-H(6B)	0.9500
C(6B)-C(7B)	1.374(14)	C(7A)-C(9A)	1.513(9)
C(7B)-C(9B)	1.520(15)	C(8A)-H(8AA)	0.9800
C(8A)-H(8AB)	0.9800	C(8A)-H(8AC)	0.9800
C(8B)-H(8BA)	0.9800	C(8B)-H(8BB)	0.9800
C(8B)-H(8BC)	0.9800	C(9A)-H(9AA)	0.9800
C(9A)-H(9AB)	0.9800	C(9A)-H(9AC)	0.9800
C(9B)-H(9BA)	0.9800	C(9B)-H(9BB)	0.9800
C(9B)-H(9BC)	0.9800	C(10)-C(11)	1.400(5)
C(10)-C(15)	1.407(4)	C(11)-H(11)	0.9500
C(11)-C(12)	1.378(5)	C(12)-H(12)	0.9500
C(12)-C(13)	1.391(5)	C(13)-H(13)	0.9500

C(13)-C(14)	1.385(5)	C(14)-C(15)	1.388(5)
C(14)-C(16)	1.511(4)	C(15)-H(15)	0.9500
C(16)-H(16A)	0.9900	C(16)-H(16B)	0.9900
C(17)-C(18)	1.399(4)	C(17)-C(22)	1.398(4)
C(18)-H(18)	0.9500	C(18)-C(19)	1.376(5)
C(19)-H(19)	0.9500	C(19)-C(20)	1.390(5)
C(20)-H(20)	0.9500	C(20)-C(21)	1.387(4)
C(21)-C(22)	1.387(4)	C(21)-C(23)	1.511(4)
C(22)-H(22)	0.9500	C(23)-H(23A)	0.9900
C(23)-H(23B)	0.9900	C(24)-C(25)	1.399(4)
C(24)-C(29)	1.393(4)	C(25)-H(25)	0.9500
C(25)-C(26)	1.375(5)	C(26)-H(26)	0.9500
C(26)-C(27)	1.398(5)	C(27)-H(27)	0.9500
C(27)-C(28)	1.384(4)	C(28)-C(29)	1.394(4)
C(28)-C(30)	1.509(4)	C(29)-H(29)	0.9500
C(30)-H(30A)	0.9900	C(30)-H(30B)	0.9900
C(31)-C(32)	1.394(5)	C(31)-C(36)	1.398(5)
C(32)-H(32)	0.9500	C(32)-C(33)	1.397(5)
C(33)-H(33)	0.9500	C(33)-C(34)	1.382(5)
C(34)-H(34)	0.9500	C(34)-C(35)	1.370(5)
C(35)-H(35)	0.9500	C(35)-C(36)	1.400(5)
C(36)-H(36)	0.9500	C(37)-C(38)	1.406(5)
C(37)-C(42)	1.392(5)	C(38)-H(38)	0.9500
C(38)-C(39)	1.389(5)	C(39)-H(39)	0.9500
C(39)-C(40)	1.375(6)	C(40)-H(40)	0.9500
C(40)-C(41)	1.379(5)	C(41)-H(41)	0.9500
C(41)-C(42)	1.386(5)	C(42)-H(42)	0.9500
C(43)-C(44)	1.398(5)	C(43)-C(48)	1.396(4)
C(44)-H(44)	0.9500	C(44)-C(45)	1.380(5)
C(45)-H(45)	0.9500	C(45)-C(46)	1.381(5)
C(46)-H(46)	0.9500	C(46)-C(47)	1.378(5)
C(47)-H(47)	0.9500	C(47)-C(48)	1.382(5)
C(48)-H(48)	0.9500	C(53B)-H(53B)	0.9500
C(53B)-C(54B)	1.3900	C(53B)-C(52B)	1.3900
C(54B)-H(54B)	0.9500	C(54B)-C(49B)	1.3900
C(49B)-C(50B)	1.3900	C(50B)-H(50B)	0.9500

C(50B)-C(51B)	1.3900	C(51B)-H(51B)	0.9500
C(51B)-C(52B)	1.3900	C(52B)-H(52B)	0.9500
C(54A)-H(54A)	0.9500	C(54A)-C(49A)	1.3900
C(54A)-C(53A)	1.3900	C(49A)-C(50A)	1.3900
C(50A)-H(50A)	0.9500	C(50A)-C(51A)	1.3900
C(51A)-H(51A)	0.9500	C(51A)-C(52A)	1.3900
C(52A)-H(52A)	0.9500	C(52A)-C(53A)	1.3900
C(53A)-H(53A)	0.9500	C(55A)-C(56A)	1.393(13)
C(55A)-C(60A)	1.423(14)	C(55B)-C(56B)	1.37(3)
C(55B)-C(60B)	1.33(3)	C(56A)-H(56A)	0.9500
C(56A)-C(57A)	1.381(7)	C(56B)-H(56B)	0.9500
C(56B)-C(57B)	1.48(3)	C(57A)-H(57A)	0.9500
C(57A)-C(58A)	1.365(9)	C(57B)-H(57B)	0.9500
C(57B)-C(58B)	1.37(2)	C(58A)-H(58A)	0.9500
C(58A)-C(59A)	1.387(11)	C(58B)-H(58B)	0.9500
C(58B)-C(59B)	1.28(3)	C(59A)-H(59A)	0.9500
C(59A)-C(60A)	1.383(13)	C(59B)-H(59B)	0.9500
C(59B)-C(60B)	1.39(4)	C(60A)-H(60A)	0.9500
C(60B)-H(60B)	0.9500	C(61)-C(62)	1.392(4)
C(61)-C(66)	1.398(4)	C(62)-H(62)	0.9500
C(62)-C(63)	1.379(5)	C(63)-H(63)	0.9500
C(63)-C(64)	1.374(5)	C(64)-H(64)	0.9500
C(64)-C(65)	1.381(5)	C(65)-H(65)	0.9500
C(65)-C(66)	1.387(5)	C(66)-H(66)	0.9500
C(67)-H(67)	0.9500	C(67)-C(68)	1.381(4)
C(68)-H(68)	0.9500	C(68)-C(69)	1.368(5)
C(69)-H(69)	0.9500	C(69)-C(70)	1.379(5)
C(70)-H(70)	0.9500	C(70)-C(71)	1.382(4)
C(71)-H(71)	0.9500	Cl(1)-C(72)	1.757(4)
Cl(2)-C(72)	1.756(5)	C(72)-H(72A)	0.9900
C(72)-H(72B)	0.9900		

O(1)-Mo(1)-O(2)	94.84(9)	O(1)-Mo(1)-O(3)	142.97(10)
O(1)-Mo(1)-N(1)	80.57(9)	O(2)-Mo(1)-O(3)	95.42(9)
O(2)-Mo(1)-N(1)	166.20(9)	O(3)-Mo(1)-N(1)	81.08(9)
C(1A)-Mo(1)-O(1)	101.1(3)	C(1A)-Mo(1)-O(2)	103.2(3)

C(1A)-Mo(1)-O(3)	110.9(3)	C(1A)-Mo(1)-N(1)	90.5(3)
C(1B)-Mo(1)-O(1)	112.8(4)	C(1B)-Mo(1)-O(2)	102.2(6)
C(1B)-Mo(1)-O(3)	99.6(4)	C(1B)-Mo(1)-N(1)	91.6(6)
O(1)-Si(1)-C(10)	109.18(14)	O(1)-Si(1)-C(31)	111.26(14)
O(1)-Si(1)-C(37)	107.59(14)	C(10)-Si(1)-C(31)	111.50(15)
C(37)-Si(1)-C(10)	107.45(15)	C(37)-Si(1)-C(31)	109.70(14)
O(2)-Si(2)-C(17)	106.35(13)	O(2)-Si(2)-C(43)	110.21(13)
O(2)-Si(2)-C(49B)	114.9(3)	O(2)-Si(2)-C(49A)	111.00(18)
C(17)-Si(2)-C(43)	112.45(14)	C(17)-Si(2)-C(49B)	108.1(3)
C(17)-Si(2)-C(49A)	108.76(15)	C(43)-Si(2)-C(49A)	108.10(18)
C(49B)-Si(2)-C(43)	104.9(3)	O(3)-Si(3)-C(24)	110.03(13)
O(3)-Si(3)-C(55A)	111.4(4)	O(3)-Si(3)-C(55B)	108.4(8)
O(3)-Si(3)-C(61)	110.14(12)	C(24)-Si(3)-C(55B)	115.6(7)
C(55A)-Si(3)-C(24)	107.6(3)	C(55A)-Si(3)-C(61)	108.5(3)
C(61)-Si(3)-C(24)	109.16(14)	C(61)-Si(3)-C(55B)	103.3(7)
Si(1)-O(1)-Mo(1)	163.95(15)	Si(2)-O(2)-Mo(1)	158.75(14)
Si(3)-O(3)-Mo(1)	169.26(14)	C(67)-N(1)-Mo(1)	122.3(2)
C(71)-N(1)-Mo(1)	120.2(2)	C(71)-N(1)-C(67)	117.5(3)
C(16)-N(2)-C(23)	111.4(2)	C(16)-N(2)-C(30)	112.1(2)
C(30)-N(2)-C(23)	111.7(2)	C(2A)-C(1A)-Mo(1)	173.8(7)
C(2B)-C(1B)-Mo(1)	170.9(10)	C(3A)-C(2A)-C(1A)	119.1(7)
C(3A)-C(2A)-C(7A)	120.7(6)	C(7A)-C(2A)-C(1A)	120.3(7)
C(3B)-C(2B)-C(1B)	119.6(11)	C(7B)-C(2B)-C(1B)	120.2(10)
C(7B)-C(2B)-C(3B)	120.1(12)	C(2A)-C(3A)-C(8A)	120.3(5)
C(4A)-C(3A)-C(2A)	119.4(7)	C(4A)-C(3A)-C(8A)	120.3(6)
C(2B)-C(3B)-C(8B)	119.8(11)	C(4B)-C(3B)-C(2B)	121.4(11)
C(4B)-C(3B)-C(8B)	118.8(11)	C(3A)-C(4A)-H(4A)	120.7
C(3A)-C(4A)-C(5A)	118.6(6)	C(5A)-C(4A)-H(4A)	120.7
C(3B)-C(4B)-H(4B)	121.8	C(3B)-C(4B)-C(5B)	116.4(12)
C(5B)-C(4B)-H(4B)	121.8	C(4A)-C(5A)-H(5A)	118.7
C(6A)-C(5A)-C(4A)	122.7(7)	C(6A)-C(5A)-H(5A)	118.7
C(4B)-C(5B)-H(5B)	118.3	C(6B)-C(5B)-C(4B)	123.4(12)
C(6B)-C(5B)-H(5B)	118.3	C(5A)-C(6A)-H(6A)	120.6
C(5A)-C(6A)-C(7A)	118.7(7)	C(7A)-C(6A)-H(6A)	120.6
C(5B)-C(6B)-H(6B)	120.9	C(7B)-C(6B)-C(5B)	118.1(11)
C(7B)-C(6B)-H(6B)	120.9	C(2A)-C(7A)-C(9A)	121.5(6)

C(6A)-C(7A)-C(2A)	119.9(6)	C(6A)-C(7A)-C(9A)	118.6(7)
C(2B)-C(7B)-C(9B)	121.3(9)	C(6B)-C(7B)-C(2B)	120.4(12)
C(6B)-C(7B)-C(9B)	118.0(11)	C(3A)-C(8A)-H(8AA)	109.5
C(3A)-C(8A)-H(8AB)	109.5	C(3A)-C(8A)-H(8AC)	109.5
H(8AA)-C(8A)-H(8AB)	109.5	H(8AA)-C(8A)-H(8AC)	109.5
H(8AB)-C(8A)-H(8AC)	109.5	C(3B)-C(8B)-H(8BA)	109.5
C(3B)-C(8B)-H(8BB)	109.5	C(3B)-C(8B)-H(8BC)	109.5
H(8BA)-C(8B)-H(8BB)	109.5	H(8BA)-C(8B)-H(8BC)	109.5
H(8BB)-C(8B)-H(8BC)	109.5	C(7A)-C(9A)-H(9AA)	109.5
C(7A)-C(9A)-H(9AB)	109.5	C(7A)-C(9A)-H(9AC)	109.5
H(9AA)-C(9A)-H(9AB)	109.5	H(9AA)-C(9A)-H(9AC)	109.5
H(9AB)-C(9A)-H(9AC)	109.5	C(7B)-C(9B)-H(9BA)	109.5
C(7B)-C(9B)-H(9BB)	109.5	C(7B)-C(9B)-H(9BC)	109.5
H(9BA)-C(9B)-H(9BB)	109.5	H(9BA)-C(9B)-H(9BC)	109.5
H(9BB)-C(9B)-H(9BC)	109.5	C(11)-C(10)-Si(1)	122.2(3)
C(11)-C(10)-C(15)	116.9(3)	C(15)-C(10)-Si(1)	120.4(3)
C(10)-C(11)-H(11)	119.3	C(12)-C(11)-C(10)	121.4(3)
C(12)-C(11)-H(11)	119.3	C(11)-C(12)-H(12)	120.0
C(11)-C(12)-C(13)	119.9(3)	C(13)-C(12)-H(12)	120.0
C(12)-C(13)-H(13)	119.6	C(14)-C(13)-C(12)	120.8(3)
C(14)-C(13)-H(13)	119.6	C(13)-C(14)-C(15)	118.3(3)
C(13)-C(14)-C(16)	120.7(3)	C(15)-C(14)-C(16)	120.9(3)
C(10)-C(15)-H(15)	118.7	C(14)-C(15)-C(10)	122.5(3)
C(14)-C(15)-H(15)	118.7	N(2)-C(16)-C(14)	113.1(3)
N(2)-C(16)-H(16A)	109.0	N(2)-C(16)-H(16B)	109.0
C(14)-C(16)-H(16A)	109.0	C(14)-C(16)-H(16B)	109.0
H(16A)-C(16)-H(16B)	107.8	C(18)-C(17)-Si(2)	124.5(2)
C(22)-C(17)-Si(2)	118.3(2)	C(22)-C(17)-C(18)	117.0(3)
C(17)-C(18)-H(18)	119.4	C(19)-C(18)-C(17)	121.2(3)
C(19)-C(18)-H(18)	119.4	C(18)-C(19)-H(19)	119.7
C(18)-C(19)-C(20)	120.6(3)	C(20)-C(19)-H(19)	119.7
C(19)-C(20)-H(20)	120.1	C(21)-C(20)-C(19)	119.9(3)
C(21)-C(20)-H(20)	120.1	C(20)-C(21)-C(22)	118.8(3)
C(20)-C(21)-C(23)	119.9(3)	C(22)-C(21)-C(23)	121.2(3)
C(17)-C(22)-H(22)	118.7	C(21)-C(22)-C(17)	122.6(3)
C(21)-C(22)-H(22)	118.7	N(2)-C(23)-C(21)	113.3(2)

N(2)-C(23)-H(23A)	108.9	N(2)-C(23)-H(23B)	108.9
C(21)-C(23)-H(23A)	108.9	C(21)-C(23)-H(23B)	108.9
H(23A)-C(23)-H(23B)	107.7	C(25)-C(24)-Si(3)	121.4(2)
C(29)-C(24)-Si(3)	121.7(2)	C(29)-C(24)-C(25)	116.9(3)
C(24)-C(25)-H(25)	119.0	C(26)-C(25)-C(24)	122.0(3)
C(26)-C(25)-H(25)	119.0	C(25)-C(26)-H(26)	120.2
C(25)-C(26)-C(27)	119.5(3)	C(27)-C(26)-H(26)	120.2
C(26)-C(27)-H(27)	119.8	C(28)-C(27)-C(26)	120.4(3)
C(28)-C(27)-H(27)	119.8	C(27)-C(28)-C(29)	118.6(3)
C(27)-C(28)-C(30)	121.6(3)	C(29)-C(28)-C(30)	119.8(3)
C(24)-C(29)-C(28)	122.6(3)	C(24)-C(29)-H(29)	118.7
C(28)-C(29)-H(29)	118.7	N(2)-C(30)-C(28)	110.1(3)
N(2)-C(30)-H(30A)	109.6	N(2)-C(30)-H(30B)	109.6
C(28)-C(30)-H(30A)	109.6	C(28)-C(30)-H(30B)	109.6
H(30A)-C(30)-H(30B)	108.1	C(32)-C(31)-Si(1)	119.6(2)
C(32)-C(31)-C(36)	117.2(3)	C(36)-C(31)-Si(1)	123.2(3)
C(31)-C(32)-H(32)	119.1	C(31)-C(32)-C(33)	121.9(3)
C(33)-C(32)-H(32)	119.1	C(32)-C(33)-H(33)	120.3
C(34)-C(33)-C(32)	119.3(3)	C(34)-C(33)-H(33)	120.3
C(33)-C(34)-H(34)	119.7	C(35)-C(34)-C(33)	120.5(3)
C(35)-C(34)-H(34)	119.7	C(34)-C(35)-H(35)	120.0
C(34)-C(35)-C(36)	119.9(3)	C(36)-C(35)-H(35)	120.0
C(31)-C(36)-C(35)	121.2(3)	C(31)-C(36)-H(36)	119.4
C(35)-C(36)-H(36)	119.4	C(38)-C(37)-Si(1)	119.3(2)
C(42)-C(37)-Si(1)	122.8(3)	C(42)-C(37)-C(38)	117.7(3)
C(37)-C(38)-H(38)	119.6	C(39)-C(38)-C(37)	120.7(3)
C(39)-C(38)-H(38)	119.6	C(38)-C(39)-H(39)	119.9
C(40)-C(39)-C(38)	120.2(3)	C(40)-C(39)-H(39)	119.9
C(39)-C(40)-H(40)	120.0	C(39)-C(40)-C(41)	120.0(3)
C(41)-C(40)-H(40)	120.0	C(40)-C(41)-H(41)	119.9
C(40)-C(41)-C(42)	120.1(3)	C(42)-C(41)-H(41)	119.9
C(37)-C(42)-H(42)	119.4	C(41)-C(42)-C(37)	121.2(3)
C(41)-C(42)-H(42)	119.4	C(44)-C(43)-Si(2)	122.3(3)
C(48)-C(43)-Si(2)	120.6(2)	C(48)-C(43)-C(44)	117.1(3)
C(43)-C(44)-H(44)	119.4	C(45)-C(44)-C(43)	121.1(3)
C(45)-C(44)-H(44)	119.4	C(44)-C(45)-H(45)	119.5

C(44)-C(45)-C(46)	120.9(3)	C(46)-C(45)-H(45)	119.5
C(45)-C(46)-H(46)	120.6	C(47)-C(46)-C(45)	118.8(3)
C(47)-C(46)-H(46)	120.6	C(46)-C(47)-H(47)	119.7
C(46)-C(47)-C(48)	120.6(3)	C(48)-C(47)-H(47)	119.7
C(43)-C(48)-H(48)	119.3	C(47)-C(48)-C(43)	121.5(3)
C(47)-C(48)-H(48)	119.3	C(54B)-C(53B)-H(53B)	120.0
C(54B)-C(53B)-C(52B)	120.0	C(52B)-C(53B)-H(53B)	120.0
C(53B)-C(54B)-H(54B)	120.0	C(53B)-C(54B)-C(49B)	120.0
C(49B)-C(54B)-H(54B)	120.0	C(54B)-C(49B)-Si(2)	119.4(6)
C(50B)-C(49B)-Si(2)	120.3(6)	C(50B)-C(49B)-C(54B)	120.0
C(49B)-C(50B)-H(50B)	120.0	C(51B)-C(50B)-C(49B)	120.0
C(51B)-C(50B)-H(50B)	120.0	C(50B)-C(51B)-H(51B)	120.0
C(50B)-C(51B)-C(52B)	120.0	C(52B)-C(51B)-H(51B)	120.0
C(53B)-C(52B)-H(52B)	120.0	C(51B)-C(52B)-C(53B)	120.0
C(51B)-C(52B)-H(52B)	120.0	C(49A)-C(54A)-H(54A)	120.0
C(49A)-C(54A)-C(53A)	120.0	C(53A)-C(54A)-H(54A)	120.0
C(54A)-C(49A)-Si(2)	121.2(2)	C(50A)-C(49A)-Si(2)	118.7(2)
C(50A)-C(49A)-C(54A)	120.0	C(49A)-C(50A)-H(50A)	120.0
C(49A)-C(50A)-C(51A)	120.0	C(51A)-C(50A)-H(50A)	120.0
C(50A)-C(51A)-H(51A)	120.0	C(52A)-C(51A)-C(50A)	120.0
C(52A)-C(51A)-H(51A)	120.0	C(51A)-C(52A)-H(52A)	120.0
C(51A)-C(52A)-C(53A)	120.0	C(53A)-C(52A)-H(52A)	120.0
C(54A)-C(53A)-H(53A)	120.0	C(52A)-C(53A)-C(54A)	120.0
C(52A)-C(53A)-H(53A)	120.0	C(56A)-C(55A)-Si(3)	124.8(7)
C(56A)-C(55A)-C(60A)	115.1(9)	C(60A)-C(55A)-Si(3)	119.8(8)
C(56B)-C(55B)-Si(3)	116.1(17)	C(60B)-C(55B)-Si(3)	120(2)
C(60B)-C(55B)-C(56B)	124(2)	C(55A)-C(56A)-H(56A)	119.1
C(57A)-C(56A)-C(55A)	121.9(7)	C(57A)-C(56A)-H(56A)	119.1
C(55B)-C(56B)-H(56B)	123.1	C(55B)-C(56B)-C(57B)	113.9(17)
C(57B)-C(56B)-H(56B)	123.1	C(56A)-C(57A)-H(57A)	119.0
C(58A)-C(57A)-C(56A)	122.0(5)	C(58A)-C(57A)-H(57A)	119.0
C(56B)-C(57B)-H(57B)	121.2	C(58B)-C(57B)-C(56B)	117.6(17)
C(58B)-C(57B)-H(57B)	121.2	C(57A)-C(58A)-H(58A)	120.8
C(57A)-C(58A)-C(59A)	118.5(5)	C(59A)-C(58A)-H(58A)	120.8
C(57B)-C(58B)-H(58B)	117.5	C(59B)-C(58B)-C(57B)	125(2)
C(59B)-C(58B)-H(58B)	117.5	C(58A)-C(59A)-H(59A)	120.1

C(60A)-C(59A)-C(58A)	119.9(8)	C(60A)-C(59A)-H(59A)	120.1
C(58B)-C(59B)-H(59B)	120.8	C(58B)-C(59B)-C(60B)	118(2)
C(60B)-C(59B)-H(59B)	120.8	C(55A)-C(60A)-H(60A)	118.7
C(59A)-C(60A)-C(55A)	122.5(10)	C(59A)-C(60A)-H(60A)	118.7
C(55B)-C(60B)-C(59B)	120(2)	C(55B)-C(60B)-H(60B)	119.9
C(59B)-C(60B)-H(60B)	119.9	C(62)-C(61)-Si(3)	120.7(2)
C(62)-C(61)-C(66)	117.7(3)	C(66)-C(61)-Si(3)	121.7(2)
C(61)-C(62)-H(62)	119.4	C(63)-C(62)-C(61)	121.3(3)
C(63)-C(62)-H(62)	119.4	C(62)-C(63)-H(63)	119.9
C(64)-C(63)-C(62)	120.2(3)	C(64)-C(63)-H(63)	119.9
C(63)-C(64)-H(64)	119.9	C(63)-C(64)-C(65)	120.1(3)
C(65)-C(64)-H(64)	119.9	C(64)-C(65)-H(65)	120.2
C(64)-C(65)-C(66)	119.7(3)	C(66)-C(65)-H(65)	120.2
C(61)-C(66)-H(66)	119.5	C(65)-C(66)-C(61)	121.1(3)
C(65)-C(66)-H(66)	119.5	N(1)-C(67)-H(67)	118.7
N(1)-C(67)-C(68)	122.7(3)	C(68)-C(67)-H(67)	118.7
C(67)-C(68)-H(68)	120.4	C(69)-C(68)-C(67)	119.1(3)
C(69)-C(68)-H(68)	120.4	C(68)-C(69)-H(69)	120.4
C(68)-C(69)-C(70)	119.1(3)	C(70)-C(69)-H(69)	120.4
C(69)-C(70)-H(70)	120.6	C(69)-C(70)-C(71)	118.7(3)
C(71)-C(70)-H(70)	120.6	N(1)-C(71)-C(70)	122.8(3)
N(1)-C(71)-H(71)	118.6	C(70)-C(71)-H(71)	118.6
Cl(1)-C(72)-H(72A)	109.1	Cl(1)-C(72)-H(72B)	109.1
Cl(2)-C(72)-Cl(1)	112.3(2)	Cl(2)-C(72)-H(72A)	109.1
Cl(2)-C(72)-H(72B)	109.1	H(72A)-C(72)-H(72B)	107.9

Single crystal structure analysis of 15b · benzene solvate

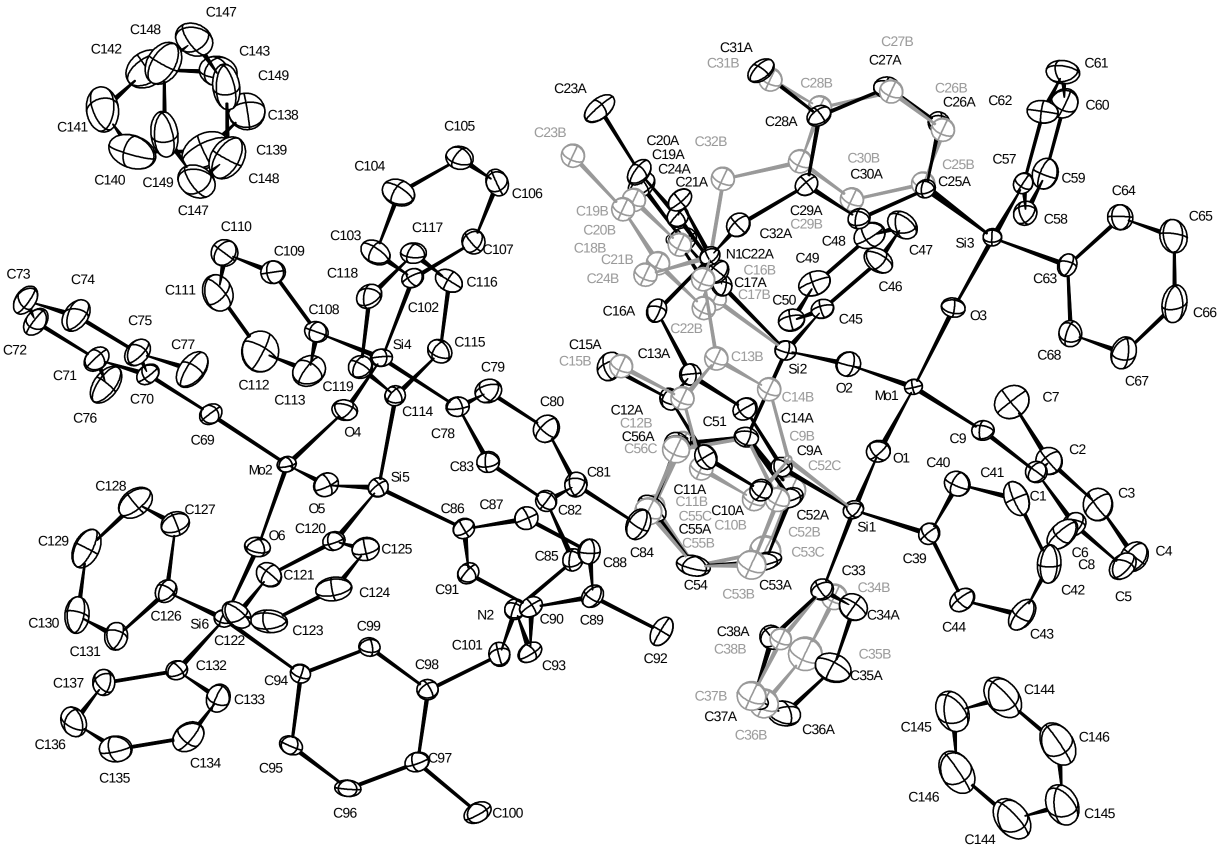

Figure S12. The molecular structure of **15b · benzene solvate**. H atoms have been removed for clarity. Main structure shown in black and disordered parts shown in grey.

X-ray Crystal Structure Analysis of 15b · benzene solvate:

$C_{75}H_{69}MoNO_3Si_3$, M_r = 1212.52 g mol^{-1}, yellow prism, crystal size 0.185 x 0.141 x 0.061 mm^3, triclinic, space group P-1 [2], a = 14.0701(9) Å, b = 20.5423(13) Å, c = 22.8551(15), α = 82.019(3)°, β = 78.595(3)°, γ = 75.507(2)°, V = 6241.5(7) Å^3, T = 100(2) K, Z = 4, D_{calc} = 1.290 g·cm^3, λ = 0.71073 Å, $\mu(Mo\text{-}K_\alpha)$ = 0.317 mm^{-1}, Gaussian absorption correction ($T_{\min}$ = 0.95849, $T_{\max}$ = 0.98591), Bruker-AXS Kappa Mach3 with APEX-II detector and IμS microfocus Mo-anode X-ray source, 0.913 < θ < 33.142°, 420731 measured reflections, 47608 independent reflections, 38271 reflections with $I > 2\sigma(I)$, R_{int} = 0.0508. The structure was solved by *SHELXT* and refined by full-matrix least-squares (*SHELXL*) against F^2 to R_1 = 0.0334 [$I > 2\sigma(I)$], wR_2 = 0.0851 [all data], 1584 parameters and 0 restraints.

Full .cif data for the compound are available under the CCDC number **CCDC 2293664**

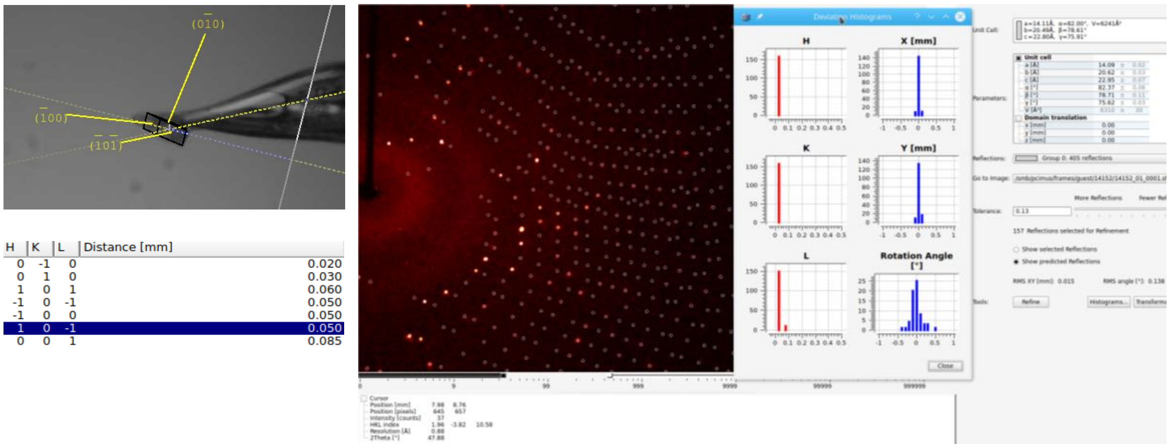

Figure S13. Crystal faces and unit cell determination/refinement of compound **15b · benzene solvate**.

INTENSITY STATISTICS FOR DATASET

Resolution	#Data	#Theory	%Complete	Redundancy	Mean I	Mean I/s	Rmerge	Rsigma
Inf - 2.57	774	774	100.0	12.76	31.08	97.77	0.0210	0.0074
2.57 - 1.72	1793	1793	100.0	14.60	13.29	82.07	0.0240	0.0085
1.72 - 1.36	2626	2626	100.0	14.74	8.99	68.07	0.0276	0.0101
1.36 - 1.19	2573	2573	100.0	14.50	7.30	56.57	0.0327	0.0120
1.19 - 1.08	2591	2591	100.0	13.29	5.16	44.40	0.0413	0.0160
1.08 - 1.00	2715	2715	100.0	10.79	4.37	34.94	0.0475	0.0209
1.00 - 0.94	2647	2647	100.0	9.45	3.42	26.82	0.0578	0.0270
0.94 - 0.89	2827	2827	100.0	8.51	3.02	23.02	0.0661	0.0323
0.89 - 0.86	2021	2021	100.0	8.04	2.94	21.27	0.0718	0.0352
0.86 - 0.82	3142	3142	100.0	7.72	2.74	19.36	0.0796	0.0394
0.82 - 0.79	2769	2769	100.0	7.40	2.44	16.89	0.0922	0.0458
0.79 - 0.77	2178	2178	100.0	7.15	2.09	14.57	0.1080	0.0547
0.77 - 0.75	2334	2334	100.0	6.92	1.78	12.28	0.1271	0.0640
0.75 - 0.73	2594	2594	100.0	6.73	1.70	11.53	0.1404	0.0702
0.73 - 0.71	2927	2927	100.0	6.51	1.62	10.51	0.1531	0.0778
0.71 - 0.69	3285	3286	100.0	6.28	1.43	9.29	0.1780	0.0910
0.69 - 0.68	1780	1782	99.9	6.07	1.32	8.46	0.1953	0.1022
0.68 - 0.66	3885	3894	99.8	5.88	1.26	7.70	0.2193	0.1118
0.66 - 0.65	2148	2157	99.6	5.69	1.11	6.68	0.2519	0.1297
0.65 - 0.64	2196	2213	99.2	5.25	1.06	6.32	0.2694	0.1445
0.64 - 0.63	1489	2518	59.1	1.57	0.83	3.33	0.3205	0.2931
0.73 - 0.63	17710	18777	94.3	5.39	1.28	7.87	0.2021	0.1145
Inf - 0.63	51294	52361	98.0	8.33	3.71	24.83	0.0505	0.0321

A resolution cut off (SHEL 999 0.7) was applied to exclude poorly determined reflections at high diffraction angles. The structure contains some disordered subunits (phenyl rings and tripodal ligand of one molecule of the asymmetric unit). These have been described as best as possible with different parts and occupancies. Isotropic atomic displacement parameters were partially applied to minor parts of the disorder. AFIX instruction was applied to force one of the aryl rings into a hexagonal shape. The correctness of the structure was also confirmed by spectroscopic methods.

Table S9. Crystal data and structure refinement of compound **15b · benzene solvate**.

Identification code	14152	
Empirical formula	$C_{75} H_{69} Mo N O_3 Si_3$	
Color	yellow	
Formula weight	1212.52 g·mol^{-1}	
Temperature	100(2) K	
Wavelength	0.71073 Å	
Crystal system	Triclinic	
Space group	*P*-1, (no. 2)	
Unit cell dimensions	a = 14.0701(9) Å	α= 82.019(3)°.
	b = 20.5423(13) Å	β= 78.595(3)°.
	c = 22.8551(15) Å	γ = 75.507(2)°.
Volume	6241.5(7) Å^3	
Z	4	
Density (calculated)	1.290 Mg·m^{-3}	
Absorption coefficient	0.317 mm^{-1}	
F(000)	2536 e	
Crystal size	0.185 x 0.141 x 0.061 mm^3	
θ range for data collection	0.913 to 33.142°.	
Index ranges	-21 ≤ h ≤ 21, -31≤ k ≤ 31, -35 ≤ l ≤ 35	
Reflections collected	420731	
Independent reflections	47608 [R_{int} = 0.0508]	
Reflections with I>2σ(I)	38271	
Completeness to θ = 25.242°	100.0 %	
Absorption correction	Gaussian	
Max. and min. transmission	0.98591 and 0.95849	
Refinement method	Full-matrix least-squares on F^2	
Data / restraints / parameters	47608 / 0 / 1584	
Goodness-of-fit on F^2	1.016	
Final R indices [I>2σ(I)]	R_1 = 0.0334	wR^2 = 0.0786
R indices (all data)	R_1 = 0.0493	wR^2 = 0.0851
Extinction coefficient	n/a	
Largest diff. peak and hole	0.960 and -0.920 e·Å^{-3}	

Table S10. Bond lengths [Å] and angles [°] of compound **15b · benzene solvate**.

Mo(1)-O(1)	1.8688(9)	Mo(1)-O(2)	1.8774(9)
Mo(1)-O(3)	1.8748(9)	Mo(1)-C(9)	1.7435(12)
Si(1)-O(1)	1.6361(9)	Si(1)-C(9A)	1.8537(18)
Si(1)-C(9B)	1.930(12)	Si(1)-C(33)	1.8624(13)
Si(1)-C(39)	1.8626(12)	Si(2)-O(2)	1.6340(9)
Si(2)-C(17A)	1.8705(15)	Si(2)-C(17B)	1.791(11)
Si(2)-C(45)	1.8661(12)	Si(2)-C(51)	1.8628(13)
Si(3)-O(3)	1.6285(9)	Si(3)-C(25A)	1.8693(17)
Si(3)-C(25B)	1.860(16)	Si(3)-C(57)	1.8702(13)
Si(3)-C(63)	1.8617(12)	N(1)-C(16A)	1.4709(17)
N(1)-C(16B)	1.460(16)	N(1)-C(24A)	1.4602(16)
N(1)-C(24B)	1.489(16)	N(1)-C(32A)	1.4662(17)
N(1)-C(32B)	1.478(17)	C(1)-C(2)	1.4091(18)
C(1)-C(6)	1.4093(19)	C(1)-C(9)	1.4420(17)
C(2)-C(3)	1.393(2)	C(2)-C(7)	1.503(2)
C(3)-H(3)	0.9500	C(3)-C(4)	1.383(3)
C(4)-H(4)	0.9500	C(4)-C(5)	1.382(2)
C(5)-H(5)	0.9500	C(5)-C(6)	1.394(2)
C(6)-C(8)	1.495(2)	C(7)-H(7A)	0.9800
C(7)-H(7B)	0.9800	C(7)-H(7C)	0.9800
C(8)-H(8A)	0.9800	C(8)-H(8B)	0.9800
C(8)-H(8C)	0.9800	C(9A)-C(10A)	1.4020(19)
C(9A)-C(14A)	1.401(2)	C(10A)-H(10A)	0.9500
C(10A)-C(11A)	1.387(2)	C(11A)-H(11A)	0.9500
C(11A)-C(12A)	1.396(2)	C(12A)-C(13A)	1.412(2)
C(12A)-C(15A)	1.508(2)	C(13A)-C(14A)	1.3936(19)
C(13A)-C(16A)	1.5149(19)	C(13B)-C(14B)	1.3900
C(13B)-C(12B)	1.3900	C(13B)-C(16B)	1.527(19)
C(14B)-H(14B)	0.9500	C(14B)-C(9B)	1.3900
C(9B)-C(10B)	1.3900	C(10B)-H(10B)	0.9500
C(10B)-C(11B)	1.3900	C(11B)-H(11B)	0.9500
C(11B)-C(12B)	1.3900	C(12B)-C(15B)	1.57(3)
C(14A)-H(14A)	0.9500	C(15A)-H(15A)	0.9800
C(15A)-H(15B)	0.9800	C(15A)-H(15C)	0.9800

C(15B)-H(15D)	0.9800	C(15B)-H(15E)	0.9800
C(15B)-H(15F)	0.9800	C(16A)-H(16A)	0.9900
C(16A)-H(16B)	0.9900	C(16B)-H(16C)	0.9900
C(16B)-H(16D)	0.9900	C(17A)-C(18A)	1.3992(19)
C(17A)-C(22A)	1.4076(19)	C(18A)-H(18A)	0.9500
C(18A)-C(19A)	1.391(2)	C(19A)-H(19A)	0.9500
C(19A)-C(20A)	1.383(2)	C(20A)-C(21A)	1.4016(19)
C(20A)-C(23A)	1.514(2)	C(21A)-C(22A)	1.3934(19)
C(21A)-C(24A)	1.5109(19)	C(21B)-C(22B)	1.3900
C(21B)-C(20B)	1.3900	C(21B)-C(24B)	1.474(19)
C(22B)-H(22B)	0.9500	C(22B)-C(17B)	1.3900
C(17B)-C(18B)	1.3900	C(18B)-H(18B)	0.9500
C(18B)-C(19B)	1.3900	C(19B)-H(19B)	0.9500
C(19B)-C(20B)	1.3900	C(20B)-C(23B)	1.509(19)
C(22A)-H(22A)	0.9500	C(23A)-H(23A)	0.9800
C(23A)-H(23B)	0.9800	C(23A)-H(23C)	0.9800
C(23B)-H(23D)	0.9800	C(23B)-H(23E)	0.9800
C(23B)-H(23F)	0.9800	C(24A)-H(24A)	0.9900
C(24A)-H(24B)	0.9900	C(24B)-H(24C)	0.9900
C(24B)-H(24D)	0.9900	C(25A)-C(26A)	1.4012(19)
C(25A)-C(30A)	1.398(2)	C(26A)-H(26A)	0.9500
C(26A)-C(27A)	1.389(2)	C(27A)-H(27A)	0.9500
C(27A)-C(28A)	1.390(2)	C(28A)-C(29A)	1.402(2)
C(28A)-C(31A)	1.521(2)	C(29A)-C(30A)	1.3989(19)
C(29A)-C(32A)	1.5170(19)	C(29B)-C(30B)	1.3900
C(29B)-C(28B)	1.3900	C(29B)-C(32B)	1.53(2)
C(30B)-H(30B)	0.9500	C(30B)-C(25B)	1.3900
C(25B)-C(26B)	1.3900	C(26B)-H(26B)	0.9500
C(26B)-C(27B)	1.3900	C(27B)-H(27B)	0.9500
C(27B)-C(28B)	1.3900	C(28B)-C(31B)	1.34(4)
C(30A)-H(30A)	0.9500	C(31A)-H(31A)	0.9800
C(31A)-H(31B)	0.9800	C(31A)-H(31C)	0.9800
C(31B)-H(31D)	0.9800	C(31B)-H(31E)	0.9800
C(31B)-H(31F)	0.9800	C(32A)-H(32A)	0.9900
C(32A)-H(32B)	0.9900	C(32B)-H(32C)	0.9900
C(32B)-H(32D)	0.9900	C(34A)-H(34A)	0.9500

C(34A)-C(33)	1.420(3)	C(34A)-C(35A)	1.395(3)
C(34B)-H(34B)	0.9500	C(34B)-C(33)	1.3900
C(34B)-C(35B)	1.3900	C(33)-C(38B)	1.3900
C(33)-C(38A)	1.403(3)	C(38B)-H(38B)	0.9500
C(38B)-C(37B)	1.3900	C(37B)-H(37B)	0.9500
C(37B)-C(36B)	1.3900	C(36B)-H(36B)	0.9500
C(36B)-C(35B)	1.3900	C(35B)-H(35B)	0.9500
C(35A)-H(35A)	0.9500	C(35A)-C(36A)	1.366(4)
C(36A)-H(36A)	0.9500	C(36A)-C(37A)	1.389(5)
C(37A)-H(37A)	0.9500	C(37A)-C(38A)	1.390(4)
C(38A)-H(38A)	0.9500	C(39)-C(40)	1.3964(19)
C(39)-C(44)	1.3983(18)	C(40)-H(40)	0.9500
C(40)-C(41)	1.387(2)	C(41)-H(41)	0.9500
C(41)-C(42)	1.383(3)	C(42)-H(42)	0.9500
C(42)-C(43)	1.381(2)	C(43)-H(43)	0.9500
C(43)-C(44)	1.3923(19)	C(44)-H(44)	0.9500
C(45)-C(46)	1.3974(18)	C(45)-C(50)	1.3975(17)
C(46)-H(46)	0.9500	C(46)-C(47)	1.388(2)
C(47)-H(47)	0.9500	C(47)-C(48)	1.382(2)
C(48)-H(48)	0.9500	C(48)-C(49)	1.381(2)
C(49)-H(49)	0.9500	C(49)-C(50)	1.3906(18)
C(50)-H(50)	0.9500	C(51)-C(52A)	1.412(4)
C(51)-C(52B)	1.354(10)	C(51)-C(52C)	1.415(10)
C(51)-C(56A)	1.403(6)	C(51)-C(56B)	1.410(15)
C(51)-C(56C)	1.345(9)	C(52A)-H(52A)	0.9500
C(52A)-C(53A)	1.390(5)	C(52B)-H(52B)	0.9500
C(52B)-C(53B)	1.391(15)	C(52C)-H(52C)	0.9500
C(52C)-C(53C)	1.376(14)	C(53A)-H(53A)	0.9500
C(53A)-C(54)	1.420(5)	C(53B)-H(53B)	0.9500
C(53B)-C(54)	1.426(11)	C(53C)-H(53C)	0.9500
C(53C)-C(54)	1.383(11)	C(54)-H(54)	0.9500
C(54)-H(54A)	0.9500	C(54)-H(54B)	0.9500
C(54)-C(55A)	1.331(7)	C(54)-C(55B)	1.370(13)
C(54)-C(55C)	1.412(9)	C(55A)-H(55A)	0.9500
C(55A)-C(56A)	1.396(8)	C(55B)-H(55B)	0.9500
C(55B)-C(56B)	1.40(2)	C(55C)-H(55C)	0.9500

C(55C)-C(56C)	1.378(13)	C(56A)-H(56A)	0.9500
C(56B)-H(56B)	0.9500	C(56C)-H(56C)	0.9500
C(57)-C(58)	1.3950(19)	C(57)-C(62)	1.4001(19)
C(58)-H(58)	0.9500	C(58)-C(59)	1.390(2)
C(59)-H(59)	0.9500	C(59)-C(60)	1.381(2)
C(60)-H(60)	0.9500	C(60)-C(61)	1.383(2)
C(61)-H(61)	0.9500	C(61)-C(62)	1.390(2)
C(62)-H(62)	0.9500	C(63)-C(64)	1.3953(18)
C(63)-C(68)	1.3976(18)	C(64)-H(64)	0.9500
C(64)-C(65)	1.390(2)	C(65)-H(65)	0.9500
C(65)-C(66)	1.386(3)	C(66)-H(66)	0.9500
C(66)-C(67)	1.384(2)	C(67)-H(67)	0.9500
C(67)-C(68)	1.391(2)	C(68)-H(68)	0.9500
Mo(2)-O(4)	1.8698(9)	Mo(2)-O(5)	1.8784(9)
Mo(2)-O(6)	1.8679(9)	Mo(2)-C(69)	1.7468(13)
Si(4)-O(4)	1.6368(10)	Si(4)-C(78)	1.8614(14)
Si(4)-C(102)	1.8631(13)	Si(4)-C(108)	1.8592(13)
Si(5)-O(5)	1.6321(9)	Si(5)-C(86)	1.8640(13)
Si(5)-C(114)	1.8622(13)	Si(5)-C(120)	1.8619(13)
Si(6)-O(6)	1.6286(9)	Si(6)-C(94)	1.8663(13)
Si(6)-C(126)	1.8638(13)	Si(6)-C(132)	1.8637(13)
N(2)-C(85)	1.4700(16)	N(2)-C(93)	1.4664(16)
N(2)-C(101)	1.4611(16)	C(69)-C(70)	1.4445(18)
C(70)-C(71)	1.4087(18)	C(70)-C(75)	1.409(2)
C(71)-C(72)	1.393(2)	C(71)-C(76)	1.501(2)
C(72)-H(72)	0.9500	C(72)-C(73)	1.382(2)
C(73)-H(73)	0.9500	C(73)-C(74)	1.383(2)
C(74)-H(74)	0.9500	C(74)-C(75)	1.390(2)
C(75)-C(77)	1.502(2)	C(76)-H(76A)	0.9800
C(76)-H(76B)	0.9800	C(76)-H(76C)	0.9800
C(77)-H(77A)	0.9800	C(77)-H(77B)	0.9800
C(77)-H(77C)	0.9800	C(78)-C(79)	1.4016(19)
C(78)-C(83)	1.3991(18)	C(79)-H(79)	0.9500
C(79)-C(80)	1.388(2)	C(80)-H(80)	0.9500
C(80)-C(81)	1.4003(19)	C(81)-C(82)	1.3986(19)
C(81)-C(84)	1.5092(19)	C(82)-C(83)	1.3947(18)

C(82)-C(85)	1.5163(18)	C(83)-H(83)	0.9500
C(84)-H(84A)	0.9800	C(84)-H(84B)	0.9800
C(84)-H(84C)	0.9800	C(85)-H(85A)	0.9900
C(85)-H(85B)	0.9900	C(86)-C(87)	1.4025(17)
C(86)-C(91)	1.3976(17)	C(87)-H(87)	0.9500
C(87)-C(88)	1.3867(18)	C(88)-H(88)	0.9500
C(88)-C(89)	1.3964(19)	C(89)-C(90)	1.3988(17)
C(89)-C(92)	1.5089(18)	C(90)-C(91)	1.3970(17)
C(90)-C(93)	1.5105(18)	C(91)-H(91)	0.9500
C(92)-H(92A)	0.9800	C(92)-H(92B)	0.9800
C(92)-H(92C)	0.9800	C(93)-H(93A)	0.9900
C(93)-H(93B)	0.9900	C(94)-C(95)	1.3988(17)
C(94)-C(99)	1.3974(16)	C(95)-H(95)	0.9500
C(95)-C(96)	1.3824(19)	C(96)-H(96)	0.9500
C(96)-C(97)	1.3970(18)	C(97)-C(98)	1.4012(17)
C(97)-C(100)	1.5126(18)	C(98)-C(99)	1.3985(17)
C(98)-C(101)	1.5131(17)	C(99)-H(99)	0.9500
C(100)-H(10C)	0.9800	C(100)-H(10D)	0.9800
C(100)-H(10E)	0.9800	C(101)-H(10F)	0.9900
C(101)-H(10G)	0.9900	C(102)-C(103)	1.396(2)
C(102)-C(107)	1.3951(19)	C(103)-H(103)	0.9500
C(103)-C(104)	1.388(2)	C(104)-H(104)	0.9500
C(104)-C(105)	1.384(2)	C(105)-H(105)	0.9500
C(105)-C(106)	1.384(2)	C(106)-H(106)	0.9500
C(106)-C(107)	1.387(2)	C(107)-H(107)	0.9500
C(108)-C(109)	1.3989(19)	C(108)-C(113)	1.390(2)
C(109)-H(109)	0.9500	C(109)-C(110)	1.385(2)
C(110)-H(110)	0.9500	C(110)-C(111)	1.376(3)
C(111)-H(111)	0.9500	C(111)-C(112)	1.378(3)
C(112)-H(112)	0.9500	C(112)-C(113)	1.386(2)
C(113)-H(113)	0.9500	C(114)-C(115)	1.3985(17)
C(114)-C(119)	1.3953(18)	C(115)-H(115)	0.9500
C(115)-C(116)	1.3865(19)	C(116)-H(116)	0.9500
C(116)-C(117)	1.387(2)	C(117)-H(117)	0.9500
C(117)-C(118)	1.385(2)	C(118)-H(118)	0.9500
C(118)-C(119)	1.3912(19)	C(119)-H(119)	0.9500

C(120)-C(121)	1.3965(18)	C(120)-C(125)	1.4012(18)
C(121)-H(121)	0.9500	C(121)-C(122)	1.397(2)
C(122)-H(122)	0.9500	C(122)-C(123)	1.381(3)
C(123)-H(123)	0.9500	C(123)-C(124)	1.381(2)
C(124)-H(124)	0.9500	C(124)-C(125)	1.388(2)
C(125)-H(125)	0.9500	C(126)-C(127)	1.4021(19)
C(126)-C(131)	1.3975(18)	C(127)-H(127)	0.9500
C(127)-C(128)	1.389(2)	C(128)-H(128)	0.9500
C(128)-C(129)	1.381(3)	C(129)-H(129)	0.9500
C(129)-C(130)	1.377(3)	C(130)-H(130)	0.9500
C(130)-C(131)	1.393(2)	C(131)-H(131)	0.9500
C(132)-C(133)	1.4001(18)	C(132)-C(137)	1.3956(18)
C(133)-H(133)	0.9500	C(133)-C(134)	1.389(2)
C(134)-H(134)	0.9500	C(134)-C(135)	1.386(2)
C(135)-H(135)	0.9500	C(135)-C(136)	1.381(2)
C(136)-H(136)	0.9500	C(136)-C(137)	1.393(2)
C(137)-H(137)	0.9500	C(138)-H(138)	0.9500
C(138)-C(139)	1.347(3)	C(138)-C(143)	1.344(3)
C(139)-H(139)	0.9500	C(139)-C(140)	1.386(4)
C(140)-H(140)	0.9500	C(140)-C(141)	1.409(4)
C(141)-H(141)	0.9500	C(141)-C(142)	1.382(4)
C(142)-H(142)	0.9500	C(142)-C(143)	1.359(3)
C(143)-H(143)	0.9500	C(144)-H(144)	0.9500
C(144)-C(145)	1.377(3)	C(144)-C(146)#1	1.375(4)
C(145)-H(145)	0.9500	C(145)-C(146)	1.383(4)
C(146)-H(146)	0.9500	C(147)-H(147)	0.9500
C(147)-C(148)	1.369(3)	C(147)-C(149)#2	1.376(4)
C(148)-H(148)	0.9500	C(148)-C(149)	1.371(3)
C(149)-H(149)	0.9500		
O(1)-Mo(1)-O(2)	113.62(4)	O(1)-Mo(1)-O(3)	111.01(4)
O(3)-Mo(1)-O(2)	113.83(4)	C(9)-Mo(1)-O(1)	105.30(5)
C(9)-Mo(1)-O(2)	105.63(5)	C(9)-Mo(1)-O(3)	106.66(5)
O(1)-Si(1)-C(9A)	106.12(7)	O(1)-Si(1)-C(9B)	104.8(5)
O(1)-Si(1)-C(33)	107.29(5)	O(1)-Si(1)-C(39)	110.23(5)
C(9A)-Si(1)-C(33)	112.39(6)	C(9A)-Si(1)-C(39)	110.71(7)

C(33)-Si(1)-C(9B)	119.4(5)	C(33)-Si(1)-C(39)	109.97(6)
C(39)-Si(1)-C(9B)	105.0(5)	O(2)-Si(2)-C(17A)	107.75(6)
O(2)-Si(2)-C(17B)	111.5(6)	O(2)-Si(2)-C(45)	110.68(5)
O(2)-Si(2)-C(51)	108.65(5)	C(17B)-Si(2)-C(45)	111.6(5)
C(17B)-Si(2)-C(51)	106.5(7)	C(45)-Si(2)-C(17A)	106.78(6)
C(51)-Si(2)-C(17A)	115.16(6)	C(51)-Si(2)-C(45)	107.81(6)
O(3)-Si(3)-C(25A)	107.15(7)	O(3)-Si(3)-C(25B)	107.0(6)
O(3)-Si(3)-C(57)	107.61(5)	O(3)-Si(3)-C(63)	109.08(5)
C(25A)-Si(3)-C(57)	111.95(7)	C(25B)-Si(3)-C(57)	106.2(9)
C(25B)-Si(3)-C(63)	114.4(8)	C(63)-Si(3)-C(25A)	108.78(6)
C(63)-Si(3)-C(57)	112.10(5)	Si(1)-O(1)-Mo(1)	176.02(6)
Si(2)-O(2)-Mo(1)	173.68(7)	Si(3)-O(3)-Mo(1)	173.96(7)
C(16B)-N(1)-C(24B)	111.1(9)	C(16B)-N(1)-C(32B)	110.5(9)
C(24A)-N(1)-C(16A)	111.08(10)	C(24A)-N(1)-C(32A)	109.12(10)
C(32A)-N(1)-C(16A)	111.02(10)	C(32B)-N(1)-C(24B)	110.0(9)
C(2)-C(1)-C(6)	120.71(12)	C(2)-C(1)-C(9)	119.62(12)
C(6)-C(1)-C(9)	119.66(12)	C(1)-C(2)-C(7)	119.93(13)
C(3)-C(2)-C(1)	118.44(14)	C(3)-C(2)-C(7)	121.61(14)
C(2)-C(3)-H(3)	119.4	C(4)-C(3)-C(2)	121.12(15)
C(4)-C(3)-H(3)	119.4	C(3)-C(4)-H(4)	119.9
C(5)-C(4)-C(3)	120.16(14)	C(5)-C(4)-H(4)	119.9
C(4)-C(5)-H(5)	119.5	C(4)-C(5)-C(6)	120.93(15)
C(6)-C(5)-H(5)	119.5	C(1)-C(6)-C(8)	120.70(12)
C(5)-C(6)-C(1)	118.61(14)	C(5)-C(6)-C(8)	120.68(13)
C(2)-C(7)-H(7A)	109.5	C(2)-C(7)-H(7B)	109.5
C(2)-C(7)-H(7C)	109.5	H(7A)-C(7)-H(7B)	109.5
H(7A)-C(7)-H(7C)	109.5	H(7B)-C(7)-H(7C)	109.5
C(6)-C(8)-H(8A)	109.5	C(6)-C(8)-H(8B)	109.5
C(6)-C(8)-H(8C)	109.5	H(8A)-C(8)-H(8B)	109.5
H(8A)-C(8)-H(8C)	109.5	H(8B)-C(8)-H(8C)	109.5
C(1)-C(9)-Mo(1)	175.03(10)	C(10A)-C(9A)-Si(1)	123.14(12)
C(14A)-C(9A)-Si(1)	119.82(13)	C(14A)-C(9A)-C(10A)	117.04(13)
C(9A)-C(10A)-H(10A)	119.7	C(11A)-C(10A)-C(9A)	120.62(12)
C(11A)-C(10A)-H(10A)	119.7	C(10A)-C(11A)-H(11A)	119.0
C(10A)-C(11A)-C(12A)	122.01(13)	C(12A)-C(11A)-H(11A)	119.0
C(11A)-C(12A)-C(13A)	118.32(13)	C(11A)-C(12A)-C(15A)	119.42(14)

C(13A)-C(12A)-C(15A)	122.24(14)	C(12A)-C(13A)-C(16A)	123.09(13)
C(14A)-C(13A)-C(12A)	118.83(13)	C(14A)-C(13A)-C(16A)	118.07(12)
C(14B)-C(13B)-C(12B)	120.0	C(14B)-C(13B)-C(16B)	117.2(10)
C(12B)-C(13B)-C(16B)	122.6(10)	C(13B)-C(14B)-H(14B)	120.0
C(9B)-C(14B)-C(13B)	120.0	C(9B)-C(14B)-H(14B)	120.0
C(14B)-C(9B)-Si(1)	123.3(9)	C(14B)-C(9B)-C(10B)	120.0
C(10B)-C(9B)-Si(1)	116.4(9)	C(9B)-C(10B)-H(10B)	120.0
C(11B)-C(10B)-C(9B)	120.0	C(11B)-C(10B)-H(10B)	120.0
C(10B)-C(11B)-H(11B)	120.0	C(10B)-C(11B)-C(12B)	120.0
C(12B)-C(11B)-H(11B)	120.0	C(13B)-C(12B)-C(15B)	121.5(13)
C(11B)-C(12B)-C(13B)	120.0	C(11B)-C(12B)-C(15B)	118.5(13)
C(9A)-C(14A)-H(14A)	118.4	C(13A)-C(14A)-C(9A)	123.16(14)
C(13A)-C(14A)-H(14A)	118.4	C(12A)-C(15A)-H(15A)	109.5
C(12A)-C(15A)-H(15B)	109.5	C(12A)-C(15A)-H(15C)	109.5
H(15A)-C(15A)-H(15B)	109.5	H(15A)-C(15A)-H(15C)	109.5
H(15B)-C(15A)-H(15C)	109.5	C(12B)-C(15B)-H(15D)	109.5
C(12B)-C(15B)-H(15E)	109.5	C(12B)-C(15B)-H(15F)	109.5
H(15D)-C(15B)-H(15E)	109.5	H(15D)-C(15B)-H(15F)	109.5
H(15E)-C(15B)-H(15F)	109.5	N(1)-C(16A)-C(13A)	111.07(11)
N(1)-C(16A)-H(16A)	109.4	N(1)-C(16A)-H(16B)	109.4
C(13A)-C(16A)-H(16A)	109.4	C(13A)-C(16A)-H(16B)	109.4
H(16A)-C(16A)-H(16B)	108.0	N(1)-C(16B)-C(13B)	113.1(12)
N(1)-C(16B)-H(16C)	109.0	N(1)-C(16B)-H(16D)	109.0
C(13B)-C(16B)-H(16C)	109.0	C(13B)-C(16B)-H(16D)	109.0
H(16C)-C(16B)-H(16D)	107.8	C(18A)-C(17A)-Si(2)	120.72(12)
C(18A)-C(17A)-C(22A)	116.14(13)	C(22A)-C(17A)-Si(2)	122.53(11)
C(17A)-C(18A)-H(18A)	119.3	C(19A)-C(18A)-C(17A)	121.33(14)
C(19A)-C(18A)-H(18A)	119.3	C(18A)-C(19A)-H(19A)	119.1
C(20A)-C(19A)-C(18A)	121.76(13)	C(20A)-C(19A)-H(19A)	119.1
C(19A)-C(20A)-C(21A)	118.44(14)	C(19A)-C(20A)-C(23A)	120.28(13)
C(21A)-C(20A)-C(23A)	121.19(13)	C(20A)-C(21A)-C(24A)	119.04(12)
C(22A)-C(21A)-C(20A)	119.36(13)	C(22A)-C(21A)-C(24A)	121.37(12)
C(22B)-C(21B)-C(20B)	120.0	C(22B)-C(21B)-C(24B)	119.6(10)
C(20B)-C(21B)-C(24B)	120.4(10)	C(21B)-C(22B)-H(22B)	120.0
C(21B)-C(22B)-C(17B)	120.0	C(17B)-C(22B)-H(22B)	120.0
C(22B)-C(17B)-Si(2)	114.8(9)	C(18B)-C(17B)-Si(2)	124.5(9)

C(18B)-C(17B)-C(22B)	120.0	C(17B)-C(18B)-H(18B)	120.0
C(17B)-C(18B)-C(19B)	120.0	C(19B)-C(18B)-H(18B)	120.0
C(18B)-C(19B)-H(19B)	120.0	C(20B)-C(19B)-C(18B)	120.0
C(20B)-C(19B)-H(19B)	120.0	C(21B)-C(20B)-C(23B)	126.5(10)
C(19B)-C(20B)-C(21B)	120.0	C(19B)-C(20B)-C(23B)	113.5(10)
C(17A)-C(22A)-H(22A)	118.5	C(21A)-C(22A)-C(17A)	122.95(12)
C(21A)-C(22A)-H(22A)	118.5	C(20A)-C(23A)-H(23A)	109.5
C(20A)-C(23A)-H(23B)	109.5	C(20A)-C(23A)-H(23C)	109.5
H(23A)-C(23A)-H(23B)	109.5	H(23A)-C(23A)-H(23C)	109.5
H(23B)-C(23A)-H(23C)	109.5	C(20B)-C(23B)-H(23D)	109.5
C(20B)-C(23B)-H(23E)	109.5	C(20B)-C(23B)-H(23F)	109.5
H(23D)-C(23B)-H(23E)	109.5	H(23D)-C(23B)-H(23F)	109.5
H(23E)-C(23B)-H(23F)	109.5	N(1)-C(24A)-C(21A)	114.49(11)
N(1)-C(24A)-H(24A)	108.6	N(1)-C(24A)-H(24B)	108.6
C(21A)-C(24A)-H(24A)	108.6	C(21A)-C(24A)-H(24B)	108.6
H(24A)-C(24A)-H(24B)	107.6	N(1)-C(24B)-H(24C)	109.5
N(1)-C(24B)-H(24D)	109.5	C(21B)-C(24B)-N(1)	110.6(11)
C(21B)-C(24B)-H(24C)	109.5	C(21B)-C(24B)-H(24D)	109.5
H(24C)-C(24B)-H(24D)	108.1	C(26A)-C(25A)-Si(3)	123.45(13)
C(30A)-C(25A)-Si(3)	120.27(13)	C(30A)-C(25A)-C(26A)	116.24(13)
C(25A)-C(26A)-H(26A)	119.4	C(27A)-C(26A)-C(25A)	121.26(13)
C(27A)-C(26A)-H(26A)	119.4	C(26A)-C(27A)-H(27A)	119.2
C(26A)-C(27A)-C(28A)	121.60(13)	C(28A)-C(27A)-H(27A)	119.2
C(27A)-C(28A)-C(29A)	118.17(13)	C(27A)-C(28A)-C(31A)	119.30(17)
C(29A)-C(28A)-C(31A)	122.50(17)	C(28A)-C(29A)-C(32A)	123.01(13)
C(30A)-C(29A)-C(28A)	119.14(13)	C(30A)-C(29A)-C(32A)	117.85(12)
C(30B)-C(29B)-C(28B)	120.0	C(30B)-C(29B)-C(32B)	116.5(11)
C(28B)-C(29B)-C(32B)	123.4(11)	C(29B)-C(30B)-H(30B)	120.0
C(25B)-C(30B)-C(29B)	120.0	C(25B)-C(30B)-H(30B)	120.0
C(30B)-C(25B)-Si(3)	119.9(12)	C(30B)-C(25B)-C(26B)	120.0
C(26B)-C(25B)-Si(3)	120.0(12)	C(25B)-C(26B)-H(26B)	120.0
C(27B)-C(26B)-C(25B)	120.0	C(27B)-C(26B)-H(26B)	120.0
C(26B)-C(27B)-H(27B)	120.0	C(26B)-C(27B)-C(28B)	120.0
C(28B)-C(27B)-H(27B)	120.0	C(27B)-C(28B)-C(29B)	120.0
C(31B)-C(28B)-C(29B)	119(2)	C(31B)-C(28B)-C(27B)	120(2)
C(25A)-C(30A)-C(29A)	123.01(13)	C(25A)-C(30A)-H(30A)	118.5

C(29A)-C(30A)-H(30A)	118.5	C(28A)-C(31A)-H(31A)	109.5
C(28A)-C(31A)-H(31B)	109.5	C(28A)-C(31A)-H(31C)	109.5
H(31A)-C(31A)-H(31B)	109.5	H(31A)-C(31A)-H(31C)	109.5
H(31B)-C(31A)-H(31C)	109.5	C(28B)-C(31B)-H(31D)	109.5
C(28B)-C(31B)-H(31E)	109.5	C(28B)-C(31B)-H(31F)	109.5
H(31D)-C(31B)-H(31E)	109.5	H(31D)-C(31B)-H(31F)	109.5
H(31E)-C(31B)-H(31F)	109.5	N(1)-C(32A)-C(29A)	110.95(11)
N(1)-C(32A)-H(32A)	109.4	N(1)-C(32A)-H(32B)	109.4
C(29A)-C(32A)-H(32A)	109.4	C(29A)-C(32A)-H(32B)	109.4
H(32A)-C(32A)-H(32B)	108.0	N(1)-C(32B)-C(29B)	111.1(12)
N(1)-C(32B)-H(32C)	109.4	N(1)-C(32B)-H(32D)	109.4
C(29B)-C(32B)-H(32C)	109.4	C(29B)-C(32B)-H(32D)	109.4
H(32C)-C(32B)-H(32D)	108.0	C(33)-C(34A)-H(34A)	119.4
C(35A)-C(34A)-H(34A)	119.4	C(35A)-C(34A)-C(33)	121.3(2)
C(33)-C(34B)-H(34B)	120.0	C(33)-C(34B)-C(35B)	120.0
C(35B)-C(34B)-H(34B)	120.0	C(34A)-C(33)-Si(1)	119.69(12)
C(34B)-C(33)-Si(1)	121.13(14)	C(34B)-C(33)-C(38B)	120.0
C(38B)-C(33)-Si(1)	118.65(14)	C(38A)-C(33)-Si(1)	123.76(15)
C(38A)-C(33)-C(34A)	116.40(18)	C(33)-C(38B)-H(38B)	120.0
C(33)-C(38B)-C(37B)	120.0	C(37B)-C(38B)-H(38B)	120.0
C(38B)-C(37B)-H(37B)	120.0	C(36B)-C(37B)-C(38B)	120.0
C(36B)-C(37B)-H(37B)	120.0	C(37B)-C(36B)-H(36B)	120.0
C(37B)-C(36B)-C(35B)	120.0	C(35B)-C(36B)-H(36B)	120.0
C(34B)-C(35B)-H(35B)	120.0	C(36B)-C(35B)-C(34B)	120.0
C(36B)-C(35B)-H(35B)	120.0	C(34A)-C(35A)-H(35A)	119.7
C(36A)-C(35A)-C(34A)	120.6(2)	C(36A)-C(35A)-H(35A)	119.7
C(35A)-C(36A)-H(36A)	120.1	C(35A)-C(36A)-C(37A)	119.8(2)
C(37A)-C(36A)-H(36A)	120.1	C(36A)-C(37A)-H(37A)	119.8
C(36A)-C(37A)-C(38A)	120.3(2)	C(38A)-C(37A)-H(37A)	119.8
C(33)-C(38A)-H(38A)	119.2	C(37A)-C(38A)-C(33)	121.7(2)
C(37A)-C(38A)-H(38A)	119.2	C(40)-C(39)-Si(1)	120.02(10)
C(40)-C(39)-C(44)	117.83(12)	C(44)-C(39)-Si(1)	122.07(10)
C(39)-C(40)-H(40)	119.3	C(41)-C(40)-C(39)	121.34(14)
C(41)-C(40)-H(40)	119.3	C(40)-C(41)-H(41)	120.1
C(42)-C(41)-C(40)	119.87(14)	C(42)-C(41)-H(41)	120.1
C(41)-C(42)-H(42)	120.0	C(43)-C(42)-C(41)	120.00(13)

C(43)-C(42)-H(42)	120.0	C(42)-C(43)-H(43)	120.0
C(42)-C(43)-C(44)	120.10(14)	C(44)-C(43)-H(43)	120.0
C(39)-C(44)-H(44)	119.6	C(43)-C(44)-C(39)	120.85(14)
C(43)-C(44)-H(44)	119.6	C(46)-C(45)-Si(2)	120.94(10)
C(46)-C(45)-C(50)	117.34(12)	C(50)-C(45)-Si(2)	121.50(9)
C(45)-C(46)-H(46)	119.3	C(47)-C(46)-C(45)	121.41(13)
C(47)-C(46)-H(46)	119.3	C(46)-C(47)-H(47)	119.9
C(48)-C(47)-C(46)	120.12(14)	C(48)-C(47)-H(47)	119.9
C(47)-C(48)-H(48)	120.2	C(49)-C(48)-C(47)	119.65(13)
C(49)-C(48)-H(48)	120.2	C(48)-C(49)-H(49)	119.9
C(48)-C(49)-C(50)	120.18(13)	C(50)-C(49)-H(49)	119.9
C(45)-C(50)-H(50)	119.4	C(49)-C(50)-C(45)	121.26(13)
C(49)-C(50)-H(50)	119.4	C(52A)-C(51)-Si(2)	119.42(18)
C(52B)-C(51)-Si(2)	122.1(4)	C(52B)-C(51)-C(56B)	116.3(8)
C(52C)-C(51)-Si(2)	118.6(4)	C(56A)-C(51)-Si(2)	122.5(3)
C(56A)-C(51)-C(52A)	117.6(3)	C(56B)-C(51)-Si(2)	121.6(6)
C(56C)-C(51)-Si(2)	123.1(4)	C(56C)-C(51)-C(52C)	117.7(6)
C(51)-C(52A)-H(52A)	119.7	C(53A)-C(52A)-C(51)	120.7(3)
C(53A)-C(52A)-H(52A)	119.7	C(51)-C(52B)-H(52B)	118.6
C(51)-C(52B)-C(53B)	122.8(9)	C(53B)-C(52B)-H(52B)	118.6
C(51)-C(52C)-H(52C)	119.1	C(53C)-C(52C)-C(51)	121.9(8)
C(53C)-C(52C)-H(52C)	119.1	C(52A)-C(53A)-H(53A)	120.3
C(52A)-C(53A)-C(54)	119.5(3)	C(54)-C(53A)-H(53A)	120.3
C(52B)-C(53B)-H(53B)	120.8	C(52B)-C(53B)-C(54)	118.5(9)
C(54)-C(53B)-H(53B)	120.8	C(52C)-C(53C)-H(53C)	120.2
C(52C)-C(53C)-C(54)	119.5(9)	C(54)-C(53C)-H(53C)	120.2
C(53A)-C(54)-H(54)	120.0	C(53B)-C(54)-H(54A)	120.1
C(53C)-C(54)-H(54B)	121.1	C(53C)-C(54)-C(55C)	117.8(6)
C(55A)-C(54)-C(53A)	120.0(3)	C(55A)-C(54)-H(54)	120.0
C(55B)-C(54)-C(53B)	119.9(8)	C(55B)-C(54)-H(54A)	120.1
C(55C)-C(54)-H(54B)	121.1	C(54)-C(55A)-H(55A)	119.3
C(54)-C(55A)-C(56A)	121.4(5)	C(56A)-C(55A)-H(55A)	119.3
C(54)-C(55B)-H(55B)	121.4	C(54)-C(55B)-C(56B)	117.2(12)
C(56B)-C(55B)-H(55B)	121.4	C(54)-C(55C)-H(55C)	119.6
C(56C)-C(55C)-C(54)	120.8(8)	C(56C)-C(55C)-H(55C)	119.6
C(51)-C(56A)-H(56A)	119.6	C(55A)-C(56A)-C(51)	120.8(5)

C(55A)-C(56A)-H(56A)	119.6	C(51)-C(56B)-H(56B)	118.1
C(55B)-C(56B)-C(51)	123.8(12)	C(55B)-C(56B)-H(56B)	118.1
C(51)-C(56C)-C(55C)	121.6(8)	C(51)-C(56C)-H(56C)	119.2
C(55C)-C(56C)-H(56C)	119.2	C(58)-C(57)-Si(3)	119.85(10)
C(58)-C(57)-C(62)	117.26(12)	C(62)-C(57)-Si(3)	122.85(10)
C(57)-C(58)-H(58)	119.4	C(59)-C(58)-C(57)	121.28(13)
C(59)-C(58)-H(58)	119.4	C(58)-C(59)-H(59)	119.9
C(60)-C(59)-C(58)	120.27(14)	C(60)-C(59)-H(59)	119.9
C(59)-C(60)-H(60)	120.1	C(59)-C(60)-C(61)	119.80(14)
C(61)-C(60)-H(60)	120.1	C(60)-C(61)-H(61)	120.1
C(60)-C(61)-C(62)	119.74(15)	C(62)-C(61)-H(61)	120.1
C(57)-C(62)-H(62)	119.2	C(61)-C(62)-C(57)	121.65(14)
C(61)-C(62)-H(62)	119.2	C(64)-C(63)-Si(3)	120.84(10)
C(64)-C(63)-C(68)	118.04(12)	C(68)-C(63)-Si(3)	121.06(10)
C(63)-C(64)-H(64)	119.4	C(65)-C(64)-C(63)	121.20(14)
C(65)-C(64)-H(64)	119.4	C(64)-C(65)-H(65)	120.1
C(66)-C(65)-C(64)	119.81(15)	C(66)-C(65)-H(65)	120.1
C(65)-C(66)-H(66)	120.0	C(67)-C(66)-C(65)	119.97(14)
C(67)-C(66)-H(66)	120.0	C(66)-C(67)-H(67)	120.0
C(66)-C(67)-C(68)	120.02(14)	C(68)-C(67)-H(67)	120.0
C(63)-C(68)-H(68)	119.6	C(67)-C(68)-C(63)	120.89(14)
C(67)-C(68)-H(68)	119.6	O(4)-Mo(2)-O(5)	114.64(4)
O(6)-Mo(2)-O(4)	110.59(4)	O(6)-Mo(2)-O(5)	111.19(4)
C(69)-Mo(2)-O(4)	106.97(5)	C(69)-Mo(2)-O(5)	105.65(5)
C(69)-Mo(2)-O(6)	107.34(5)	O(4)-Si(4)-C(78)	106.97(6)
O(4)-Si(4)-C(102)	106.48(6)	O(4)-Si(4)-C(108)	110.94(6)
C(78)-Si(4)-C(102)	110.78(6)	C(108)-Si(4)-C(78)	111.12(6)
C(108)-Si(4)-C(102)	110.40(6)	O(5)-Si(5)-C(86)	108.23(5)
O(5)-Si(5)-C(114)	109.38(5)	O(5)-Si(5)-C(120)	108.10(5)
C(114)-Si(5)-C(86)	109.23(6)	C(120)-Si(5)-C(86)	111.12(5)
C(120)-Si(5)-C(114)	110.72(6)	O(6)-Si(6)-C(94)	106.46(5)
O(6)-Si(6)-C(126)	107.91(5)	O(6)-Si(6)-C(132)	109.42(5)
C(126)-Si(6)-C(94)	112.02(6)	C(126)-Si(6)-C(132)	113.93(6)
C(132)-Si(6)-C(94)	106.83(5)	Si(4)-O(4)-Mo(2)	169.14(7)
Si(5)-O(5)-Mo(2)	163.36(6)	Si(6)-O(6)-Mo(2)	173.59(7)
C(93)-N(2)-C(85)	112.55(10)	C(101)-N(2)-C(85)	110.27(10)

C(101)-N(2)-C(93)	110.92(10)	C(70)-C(69)-Mo(2)	176.91(11)
C(71)-C(70)-C(69)	120.45(12)	C(71)-C(70)-C(75)	120.37(12)
C(75)-C(70)-C(69)	119.16(12)	C(70)-C(71)-C(76)	120.53(13)
C(72)-C(71)-C(70)	118.69(14)	C(72)-C(71)-C(76)	120.76(13)
C(71)-C(72)-H(72)	119.4	C(73)-C(72)-C(71)	121.15(14)
C(73)-C(72)-H(72)	119.4	C(72)-C(73)-H(73)	120.1
C(72)-C(73)-C(74)	119.85(14)	C(74)-C(73)-H(73)	120.1
C(73)-C(74)-H(74)	119.4	C(73)-C(74)-C(75)	121.14(15)
C(75)-C(74)-H(74)	119.4	C(70)-C(75)-C(77)	120.05(13)
C(74)-C(75)-C(70)	118.81(13)	C(74)-C(75)-C(77)	121.14(14)
C(71)-C(76)-H(76A)	109.5	C(71)-C(76)-H(76B)	109.5
C(71)-C(76)-H(76C)	109.5	H(76A)-C(76)-H(76B)	109.5
H(76A)-C(76)-H(76C)	109.5	H(76B)-C(76)-H(76C)	109.5
C(75)-C(77)-H(77A)	109.5	C(75)-C(77)-H(77B)	109.5
C(75)-C(77)-H(77C)	109.5	H(77A)-C(77)-H(77B)	109.5
H(77A)-C(77)-H(77C)	109.5	H(77B)-C(77)-H(77C)	109.5
C(79)-C(78)-Si(4)	124.28(10)	C(83)-C(78)-Si(4)	118.80(10)
C(83)-C(78)-C(79)	116.91(12)	C(78)-C(79)-H(79)	119.6
C(80)-C(79)-C(78)	120.79(13)	C(80)-C(79)-H(79)	119.6
C(79)-C(80)-H(80)	119.2	C(79)-C(80)-C(81)	121.69(13)
C(81)-C(80)-H(80)	119.2	C(80)-C(81)-C(84)	119.23(13)
C(82)-C(81)-C(80)	118.33(12)	C(82)-C(81)-C(84)	122.44(12)
C(81)-C(82)-C(85)	123.87(12)	C(83)-C(82)-C(81)	119.30(12)
C(83)-C(82)-C(85)	116.82(11)	C(78)-C(83)-H(83)	118.5
C(82)-C(83)-C(78)	122.97(12)	C(82)-C(83)-H(83)	118.5
C(81)-C(84)-H(84A)	109.5	C(81)-C(84)-H(84B)	109.5
C(81)-C(84)-H(84C)	109.5	H(84A)-C(84)-H(84B)	109.5
H(84A)-C(84)-H(84C)	109.5	H(84B)-C(84)-H(84C)	109.5
N(2)-C(85)-C(82)	110.34(10)	N(2)-C(85)-H(85A)	109.6
N(2)-C(85)-H(85B)	109.6	C(82)-C(85)-H(85A)	109.6
C(82)-C(85)-H(85B)	109.6	H(85A)-C(85)-H(85B)	108.1
C(87)-C(86)-Si(5)	121.59(9)	C(91)-C(86)-Si(5)	121.49(9)
C(91)-C(86)-C(87)	116.87(11)	C(86)-C(87)-H(87)	119.5
C(88)-C(87)-C(86)	121.03(12)	C(88)-C(87)-H(87)	119.5
C(87)-C(88)-H(88)	119.3	C(87)-C(88)-C(89)	121.45(11)
C(89)-C(88)-H(88)	119.3	C(88)-C(89)-C(90)	118.57(11)

C(88)-C(89)-C(92)	119.55(11)	C(90)-C(89)-C(92)	121.88(12)
C(89)-C(90)-C(93)	122.56(11)	C(91)-C(90)-C(89)	119.27(12)
C(91)-C(90)-C(93)	118.14(11)	C(86)-C(91)-H(91)	118.6
C(90)-C(91)-C(86)	122.80(11)	C(90)-C(91)-H(91)	118.6
C(89)-C(92)-H(92A)	109.5	C(89)-C(92)-H(92B)	109.5
C(89)-C(92)-H(92C)	109.5	H(92A)-C(92)-H(92B)	109.5
H(92A)-C(92)-H(92C)	109.5	H(92B)-C(92)-H(92C)	109.5
N(2)-C(93)-C(90)	111.04(10)	N(2)-C(93)-H(93A)	109.4
N(2)-C(93)-H(93B)	109.4	C(90)-C(93)-H(93A)	109.4
C(90)-C(93)-H(93B)	109.4	H(93A)-C(93)-H(93B)	108.0
C(95)-C(94)-Si(6)	120.52(9)	C(99)-C(94)-Si(6)	122.54(9)
C(99)-C(94)-C(95)	116.89(11)	C(94)-C(95)-H(95)	119.3
C(96)-C(95)-C(94)	121.35(11)	C(96)-C(95)-H(95)	119.3
C(95)-C(96)-H(96)	119.2	C(95)-C(96)-C(97)	121.51(12)
C(97)-C(96)-H(96)	119.2	C(96)-C(97)-C(98)	118.14(12)
C(96)-C(97)-C(100)	119.01(12)	C(98)-C(97)-C(100)	122.83(12)
C(97)-C(98)-C(101)	122.75(11)	C(99)-C(98)-C(97)	119.58(11)
C(99)-C(98)-C(101)	117.64(11)	C(94)-C(99)-C(98)	122.45(11)
C(94)-C(99)-H(99)	118.8	C(98)-C(99)-H(99)	118.8
C(97)-C(100)-H(10C)	109.5	C(97)-C(100)-H(10D)	109.5
C(97)-C(100)-H(10E)	109.5	H(10C)-C(100)-H(10D)	109.5
H(10C)-C(100)-H(10E)	109.5	H(10D)-C(100)-H(10E)	109.5
N(2)-C(101)-C(98)	112.32(10)	N(2)-C(101)-H(10F)	109.1
N(2)-C(101)-H(10G)	109.1	C(98)-C(101)-H(10F)	109.1
C(98)-C(101)-H(10G)	109.1	H(10F)-C(101)-H(10G)	107.9
C(103)-C(102)-Si(4)	121.19(10)	C(107)-C(102)-Si(4)	120.96(10)
C(107)-C(102)-C(103)	117.85(12)	C(102)-C(103)-H(103)	119.5
C(104)-C(103)-C(102)	121.03(13)	C(104)-C(103)-H(103)	119.5
C(103)-C(104)-H(104)	119.9	C(105)-C(104)-C(103)	120.22(14)
C(105)-C(104)-H(104)	119.9	C(104)-C(105)-H(105)	120.2
C(104)-C(105)-C(106)	119.55(13)	C(106)-C(105)-H(105)	120.2
C(105)-C(106)-H(106)	119.9	C(105)-C(106)-C(107)	120.18(14)
C(107)-C(106)-H(106)	119.9	C(102)-C(107)-H(107)	119.4
C(106)-C(107)-C(102)	121.15(14)	C(106)-C(107)-H(107)	119.4
C(109)-C(108)-Si(4)	121.07(10)	C(113)-C(108)-Si(4)	121.32(11)
C(113)-C(108)-C(109)	117.61(13)	C(108)-C(109)-H(109)	119.4

C(110)-C(109)-C(108)	121.12(14)	C(110)-C(109)-H(109)	119.4
C(109)-C(110)-H(110)	119.9	C(111)-C(110)-C(109)	120.19(16)
C(111)-C(110)-H(110)	119.9	C(110)-C(111)-H(111)	120.2
C(110)-C(111)-C(112)	119.62(15)	C(112)-C(111)-H(111)	120.2
C(111)-C(112)-H(112)	119.8	C(111)-C(112)-C(113)	120.40(17)
C(113)-C(112)-H(112)	119.8	C(108)-C(113)-H(113)	119.5
C(112)-C(113)-C(108)	121.05(17)	C(112)-C(113)-H(113)	119.5
C(115)-C(114)-Si(5)	119.46(10)	C(119)-C(114)-Si(5)	122.53(9)
C(119)-C(114)-C(115)	117.81(12)	C(114)-C(115)-H(115)	119.5
C(116)-C(115)-C(114)	120.98(13)	C(116)-C(115)-H(115)	119.5
C(115)-C(116)-H(116)	119.9	C(115)-C(116)-C(117)	120.27(13)
C(117)-C(116)-H(116)	119.9	C(116)-C(117)-H(117)	120.1
C(118)-C(117)-C(116)	119.78(13)	C(118)-C(117)-H(117)	120.1
C(117)-C(118)-H(118)	120.1	C(117)-C(118)-C(119)	119.73(13)
C(119)-C(118)-H(118)	120.1	C(114)-C(119)-H(119)	119.3
C(118)-C(119)-C(114)	121.42(12)	C(118)-C(119)-H(119)	119.3
C(121)-C(120)-Si(5)	122.02(10)	C(121)-C(120)-C(125)	117.83(12)
C(125)-C(120)-Si(5)	120.15(10)	C(120)-C(121)-H(121)	119.6
C(120)-C(121)-C(122)	120.81(14)	C(122)-C(121)-H(121)	119.6
C(121)-C(122)-H(122)	119.9	C(123)-C(122)-C(121)	120.13(15)
C(123)-C(122)-H(122)	119.9	C(122)-C(123)-H(123)	120.0
C(122)-C(123)-C(124)	119.97(14)	C(124)-C(123)-H(123)	120.0
C(123)-C(124)-H(124)	120.0	C(123)-C(124)-C(125)	120.08(14)
C(125)-C(124)-H(124)	120.0	C(120)-C(125)-H(125)	119.4
C(124)-C(125)-C(120)	121.18(13)	C(124)-C(125)-H(125)	119.4
C(127)-C(126)-Si(6)	119.50(10)	C(131)-C(126)-Si(6)	122.96(10)
C(131)-C(126)-C(127)	117.43(12)	C(126)-C(127)-H(127)	119.3
C(128)-C(127)-C(126)	121.35(14)	C(128)-C(127)-H(127)	119.3
C(127)-C(128)-H(128)	120.0	C(129)-C(128)-C(127)	119.96(15)
C(129)-C(128)-H(128)	120.0	C(128)-C(129)-H(129)	120.1
C(130)-C(129)-C(128)	119.89(14)	C(130)-C(129)-H(129)	120.1
C(129)-C(130)-H(130)	119.8	C(129)-C(130)-C(131)	120.36(15)
C(131)-C(130)-H(130)	119.8	C(126)-C(131)-H(131)	119.5
C(130)-C(131)-C(126)	120.99(14)	C(130)-C(131)-H(131)	119.5
C(133)-C(132)-Si(6)	116.96(10)	C(137)-C(132)-Si(6)	125.51(10)
C(137)-C(132)-C(133)	117.52(12)	C(132)-C(133)-H(133)	119.2

C(134)-C(133)-C(132)	121.59(13)	C(134)-C(133)-H(133)	119.2
C(133)-C(134)-H(134)	120.2	C(135)-C(134)-C(133)	119.67(15)
C(135)-C(134)-H(134)	120.2	C(134)-C(135)-H(135)	120.0
C(136)-C(135)-C(134)	119.92(14)	C(136)-C(135)-H(135)	120.0
C(135)-C(136)-H(136)	119.9	C(135)-C(136)-C(137)	120.19(14)
C(137)-C(136)-H(136)	119.9	C(132)-C(137)-H(137)	119.5
C(136)-C(137)-C(132)	121.10(14)	C(136)-C(137)-H(137)	119.5
C(139)-C(138)-H(138)	119.6	C(143)-C(138)-H(138)	119.6
C(143)-C(138)-C(139)	120.9(2)	C(138)-C(139)-H(139)	119.9
C(138)-C(139)-C(140)	120.3(2)	C(140)-C(139)-H(139)	119.9
C(139)-C(140)-H(140)	120.6	C(139)-C(140)-C(141)	118.7(2)
C(141)-C(140)-H(140)	120.6	C(140)-C(141)-H(141)	120.5
C(142)-C(141)-C(140)	119.0(2)	C(142)-C(141)-H(141)	120.5
C(141)-C(142)-H(142)	120.2	C(143)-C(142)-C(141)	119.6(2)
C(143)-C(142)-H(142)	120.2	C(138)-C(143)-C(142)	121.5(2)
C(138)-C(143)-H(143)	119.3	C(142)-C(143)-H(143)	119.3
C(145)-C(144)-H(144)	119.9	C(146)#1-C(144)-H(144)	119.9
C(146)#1-C(144)-C(145)	120.2(3)	C(144)-C(145)-H(145)	119.8
C(144)-C(145)-C(146)	120.3(2)	C(146)-C(145)-H(145)	119.8
C(144)#1-C(146)-C(145)	119.4(2)	C(144)#1-C(146)-H(146)	120.3
C(145)-C(146)-H(146)	120.3	C(148)-C(147)-H(147)	120.0
C(148)-C(147)-C(149)#2	120.1(2)	C(149)#2-C(147)-H(147)	120.0
C(147)-C(148)-H(148)	120.1	C(147)-C(148)-C(149)	119.7(2)
C(149)-C(148)-H(148)	120.1	C(147)#2-C(149)-H(149)	119.9
C(148)-C(149)-C(147)#2	120.2(2)	C(148)-C(149)-H(149)	119.9 _

Symmetry transformations used to generate equivalent atoms:

#1 -x+1,-y+2,-z+1   #2 -x,-y,-z+2

Single crystal structure analysis of 16b

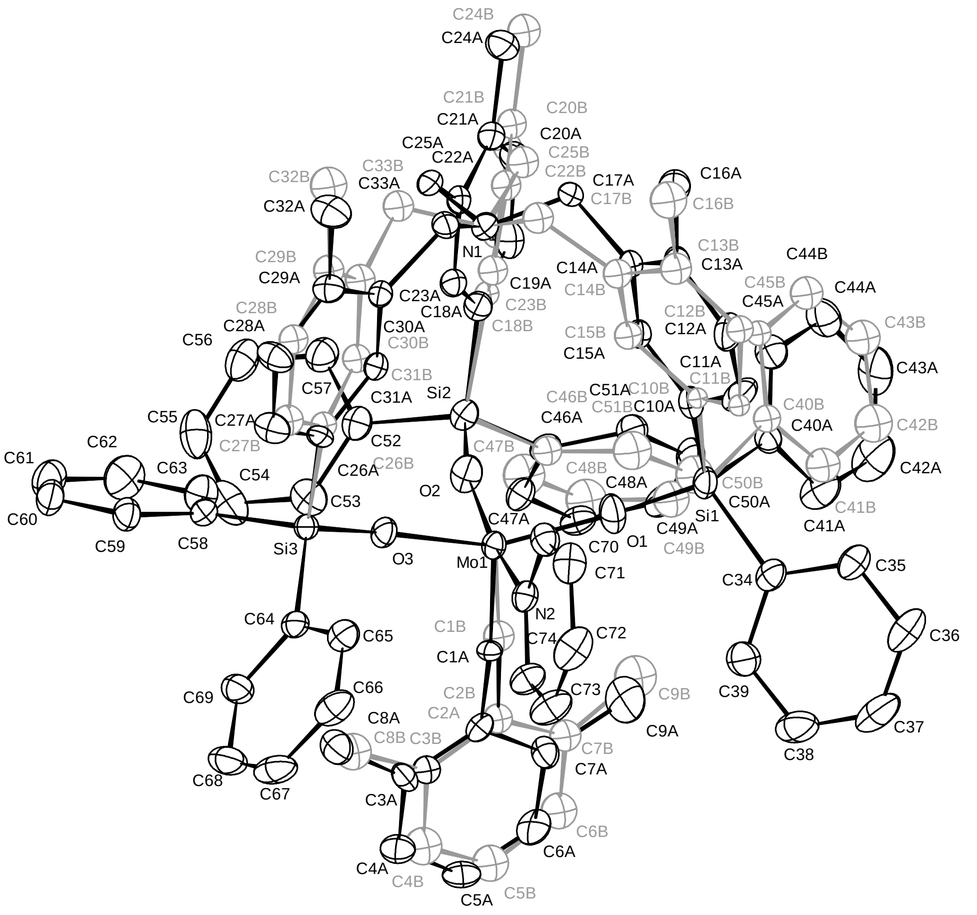

Figure S14. The molecular structure of **16b**. H atoms have been removed for clarity. Main structure shown in black and disordered parts shown in grey.

X-ray Crystal Structure Analysis of 16b:

C_{74} H_{68} Mo N_2 O_3 Si_3, M_r = 1213.51 g mol^{-1}, purple prism, crystal size 0.19 x 0.18 x 0.08 mm^3, triclinic, space group *P*-1 [2], a = 12.196(2) Å, b = 14.638(2) Å, c = 19.3970(9) Å, α = 83.076(7)°, β = 76.990(6)°, γ = 67.589(12)°, V = 3116.8(8) Å^3, T = 100(2) K, Z = 2, D_{calc} = 1.293 g·cm^3, λ = 0.71073 Å, $\mu(Mo\text{-}K_{\alpha})$ = 0.318 mm^{-1}, Gaussian absorption correction ($T_{\min}$ = 0.92274, $T_{\max}$ = 0.97586), Bruker AXS Enraf-Nonius KappaCCD diffractometer with a FR591 rotating Mo-anode X-ray source, 2.678 < θ < 31.506°, 106119 measured reflections, 20734 independent reflections, 16516 reflections with $I > 2\sigma(I)$, R_{int} = 0.0461. The structure was solved by *SHELXS* and refined by full-matrix least-squares (*SHELXL*) against F^2 to R_1 = 0.0337 [$I > 2\sigma(I)$], wR_2 = 0.0840 [all data], 926 parameters and 0 restraints.

Full .cif data for the compound are available under the CCDC number **CCDC 2293660**

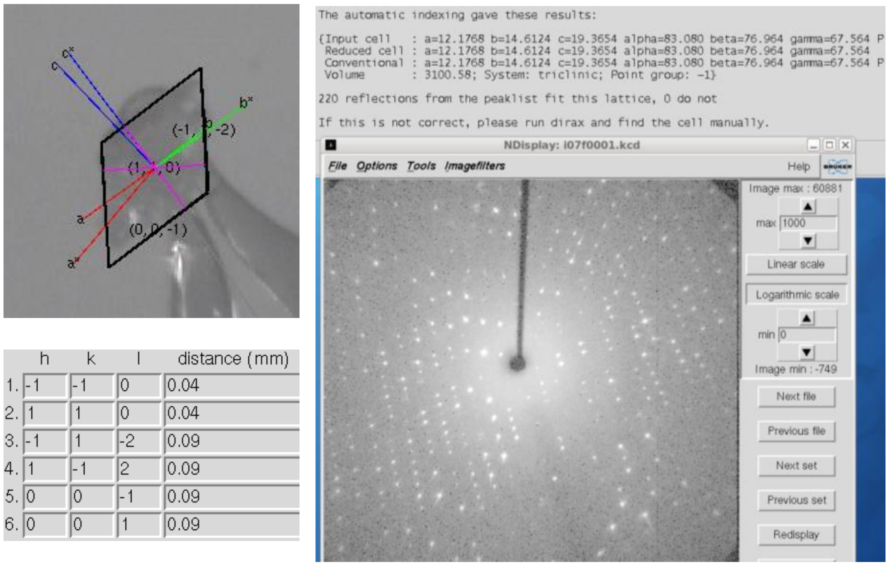

Figure S15. Crystal faces and unit cell determination/refinement of compound **16b**.

INTENSITY STATISTICS FOR DATASET

Resolution	#Data	#Theory	%Complete	Redundancy	Mean I	Mean I/s	Rmerge	Rsigma
Inf - 2.61	357	372	96.0	10.78	95.68	72.54	0.0318	0.0125
2.61 - 1.76	828	828	100.0	7.66	43.59	53.25	0.0294	0.0152
1.76 - 1.40	1182	1182	100.0	6.80	28.59	45.42	0.0308	0.0173
1.40 - 1.22	1204	1204	100.0	6.40	21.95	39.17	0.0333	0.0189
1.22 - 1.11	1188	1188	100.0	6.07	15.05	33.18	0.0371	0.0219
1.11 - 1.03	1205	1205	100.0	5.88	13.32	29.99	0.0403	0.0241
1.03 - 0.97	1174	1174	100.0	5.58	10.40	26.25	0.0472	0.0282
0.97 - 0.92	1229	1229	100.0	5.32	8.62	23.06	0.0547	0.0331
0.92 - 0.88	1193	1193	100.0	5.08	7.55	19.43	0.0623	0.0384
0.88 - 0.85	1047	1047	100.0	4.85	7.46	19.17	0.0669	0.0415
0.85 - 0.82	1212	1212	100.0	4.67	6.45	16.29	0.0749	0.0497
0.82 - 0.79	1403	1403	100.0	4.53	5.44	13.54	0.0924	0.0627
0.79 - 0.77	1049	1049	100.0	4.28	4.75	11.48	0.1026	0.0774
0.77 - 0.75	1176	1176	100.0	4.19	3.87	8.98	0.1300	0.1034
0.75 - 0.73	1322	1322	100.0	4.01	3.70	7.85	0.1416	0.1234
0.73 - 0.71	1468	1468	100.0	3.87	3.20	6.15	0.1633	0.1629
0.71 - 0.70	775	775	100.0	3.78	2.86	4.97	0.1872	0.2039
0.70 - 0.68	1724	1724	100.0	3.59	2.81	4.36	0.1932	0.2371
0.68 - 0.67	961	961	100.0	3.47	2.31	3.12	0.2357	0.3289
0.67 - 0.66	976	978	99.8	3.43	2.35	2.91	0.2446	0.3585
0.66 - 0.65	901	911	98.9	3.23	2.12	2.43	0.2744	0.4287
0.75 - 0.65	8127	8139	99.9	3.65	2.84	4.77	0.1888	0.2314
Inf - 0.65	23574	23601	99.9	4.91	10.69	19.04	0.0478	0.0447

A resolution cut off (SHEL 999 0.68) was applied to exclude poorly determined intensities at high diffraction angles. The missing low angle reflections were presumably shadowed by the primary beam stop. Two reflections (OMIT -3 6 3; 0 0 6) with high I\σ(I) were excluded before the final refinement cycles. The structure contains some disordered subunits (alkylidyne ligand, phenyl rings and tripodal ligand). These have been described as best as possible with different parts and

occupancies. Isotropic atomic displacement parameters were partially applied to minor parts of the disorder. AFIX instruction was applied to force one of the aryl rings into a hexagonal shape.

Table S11. Crystal data and structure refinement of compound **16b**.

Identification code	14313	
Empirical formula	$C_{74} H_{68} Mo N_2 O_3 Si_3$	
Color	purple	
Formula weight	1213.51 g·mol^{-1}	
Temperature	100(2) K	
Wavelength	0.71073 Å	
Crystal system	Triclinic	
Space group	*P*-1, (no. 2)	
Unit cell dimensions	a = 12.196(2) Å	α= 83.076(7)°.
	b = 14.638(2) Å	β= 76.990(6)°.
	c = 19.3970(9) Å	γ = 67.589(12)°.
Volume	3116.8(8) Å^3	
Z	2	
Density (calculated)	1.293 Mg·m^{-3}	
Absorption coefficient	0.318 mm^{-1}	
F(000)	1268 e	
Crystal size	0.19 x 0.18 x 0.08 mm^3	
θ range for data collection	2.678 to 31.506°.	
Index ranges	$-17 \le h \le 17$, $-21 \le k \le 21$, $-28 \le l \le 28$	
Reflections collected	106119	
Independent reflections	20734 [R_{int} = 0.0461]	
Reflections with I>2σ(I)	16516	
Completeness to θ = 25.242°	99.8 %	
Absorption correction	Gaussian	
Max. and min. transmission	0.97586 and 0.92274	
Refinement method	Full-matrix least-squares on F^2	
Data / restraints / parameters	20734 / 0 / 926	
Goodness-of-fit on F^2	1.024	
Final R indices [I>2σ(I)]	R_1 = 0.0337	wR^2 = 0.0779
R indices (all data)	R_1 = 0.0510	wR^2 = 0.0840
Extinction coefficient	n/a	
Largest diff. peak and hole	0.477 and -0.828 e·Å^{-3}	

Table S12. Bond lengths [Å] and angles [°] of compound **16b**.

Mo(1)-O(1)	1.9370(10)	Mo(1)-O(2)	1.9067(10)
Mo(1)-O(3)	1.9284(10)	Mo(1)-N(2)	2.2545(12)
Mo(1)-C(1A)	1.810(5)	Mo(1)-C(1B)	1.605(8)
Si(1)-O(1)	1.6172(11)	Si(1)-C(10A)	1.800(4)
Si(1)-C(10B)	2.037(8)	Si(1)-C(34)	1.8735(15)
Si(1)-C(40A)	1.881(4)	Si(1)-C(40B)	1.916(10)
Si(2)-O(2)	1.6130(11)	Si(2)-C(18A)	1.818(4)
Si(2)-C(18B)	1.987(10)	Si(2)-C(46A)	1.869(3)
Si(2)-C(46B)	1.891(6)	Si(2)-C(52)	1.8766(15)
Si(3)-O(3)	1.6160(10)	Si(3)-C(26A)	1.850(5)
Si(3)-C(26B)	1.930(14)	Si(3)-C(58)	1.8692(14)
Si(3)-C(64)	1.8772(14)	N(1)-C(17A)	1.442(2)
N(1)-C(17B)	1.425(5)	N(1)-C(25A)	1.450(2)
N(1)-C(25B)	1.596(5)	N(1)-C(33A)	1.505(2)
N(1)-C(33B)	1.408(5)	N(2)-C(70)	1.3474(18)
N(2)-C(74)	1.3391(19)	C(1A)-C(2A)	1.424(6)
C(1B)-C(2B)	1.530(11)	C(2A)-C(3A)	1.420(6)
C(2A)-C(7A)	1.428(5)	C(2B)-C(3B)	1.384(7)
C(2B)-C(7B)	1.394(9)	C(3A)-C(4A)	1.365(4)
C(3A)-C(8A)	1.498(6)	C(3B)-C(4B)	1.460(9)
C(3B)-C(8B)	1.496(9)	C(4A)-H(4A)	0.9500
C(4A)-C(5A)	1.391(5)	C(4B)-H(4B)	0.9500
C(4B)-C(5B)	1.371(8)	C(5A)-H(5A)	0.9500
C(5A)-C(6A)	1.367(5)	C(5B)-H(5B)	0.9500
C(5B)-C(6B)	1.362(7)	C(6A)-H(6A)	0.9500
C(6A)-C(7A)	1.403(4)	C(6B)-H(6B)	0.9500
C(6B)-C(7B)	1.404(8)	C(7A)-C(9A)	1.515(6)
C(7B)-C(9B)	1.447(9)	C(8A)-H(8AA)	0.9800
C(8A)-H(8AB)	0.9800	C(8A)-H(8AC)	0.9800
C(8B)-H(8BA)	0.9800	C(8B)-H(8BB)	0.9800
C(8B)-H(8BC)	0.9800	C(9A)-H(9AA)	0.9800
C(9A)-H(9AB)	0.9800	C(9A)-H(9AC)	0.9800
C(9B)-H(9BA)	0.9800	C(9B)-H(9BB)	0.9800
C(9B)-H(9BC)	0.9800	C(10A)-C(11A)	1.466(7)

C(10A)-C(15A)	1.377(5)	C(10B)-C(11B)	1.242(14)
C(10B)-C(15B)	1.468(11)	C(11A)-H(11A)	0.9500
C(11A)-C(12A)	1.314(8)	C(11B)-H(11B)	0.9500
C(11B)-C(12B)	1.559(16)	C(12A)-H(12A)	0.9500
C(12A)-C(13A)	1.403(5)	C(12B)-H(12B)	0.9500
C(12B)-C(13B)	1.389(13)	C(13A)-C(14A)	1.399(4)
C(13A)-C(16A)	1.516(4)	C(13B)-C(14B)	1.375(9)
C(13B)-C(16B)	1.510(10)	C(14A)-C(15A)	1.401(3)
C(14A)-C(17A)	1.514(3)	C(14B)-C(15B)	1.386(7)
C(14B)-C(17B)	1.528(7)	C(15A)-H(15A)	0.9500
C(15B)-H(15B)	0.9500	C(16A)-H(16A)	0.9800
C(16A)-H(16B)	0.9800	C(16A)-H(16C)	0.9800
C(16B)-H(16D)	0.9800	C(16B)-H(16E)	0.9800
C(16B)-H(16F)	0.9800	C(17A)-H(17A)	0.9900
C(17A)-H(17B)	0.9900	C(17B)-H(17C)	0.9900
C(17B)-H(17D)	0.9900	C(18A)-C(19A)	1.420(5)
C(18A)-C(23A)	1.390(5)	C(18B)-C(19B)	1.324(12)
C(18B)-C(23B)	1.417(11)	C(19A)-H(19A)	0.9500
C(19A)-C(20A)	1.379(5)	C(19B)-H(19B)	0.9500
C(19B)-C(20B)	1.437(12)	C(20A)-H(20A)	0.9500
C(20A)-C(21A)	1.398(4)	C(20B)-H(20B)	0.9500
C(20B)-C(21B)	1.374(9)	C(21A)-C(22A)	1.398(3)
C(21A)-C(24A)	1.508(3)	C(21B)-C(22B)	1.372(7)
C(21B)-C(24B)	1.518(8)	C(22A)-C(23A)	1.398(3)
C(22A)-C(25A)	1.513(3)	C(22B)-C(23B)	1.405(7)
C(22B)-C(25B)	1.525(7)	C(23A)-H(23A)	0.9500
C(23B)-H(23B)	0.9500	C(24A)-H(24A)	0.9800
C(24A)-H(24B)	0.9800	C(24A)-H(24C)	0.9800
C(24B)-H(24D)	0.9800	C(24B)-H(24E)	0.9800
C(24B)-H(24F)	0.9800	C(25A)-H(25A)	0.9900
C(25A)-H(25B)	0.9900	C(25B)-H(25C)	0.9900
C(25B)-H(25D)	0.9900	C(26A)-C(27A)	1.403(7)
C(26A)-C(31A)	1.435(5)	C(26B)-C(27B)	1.358(17)
C(26B)-C(31B)	1.332(12)	C(27A)-H(27A)	0.9500
C(27A)-C(28A)	1.371(5)	C(27B)-H(27B)	0.9500
C(27B)-C(28B)	1.446(14)	C(28A)-H(28A)	0.9500

C(28A)-C(29A)	1.400(3)	C(28B)-H(28B)	0.9500
C(28B)-C(29B)	1.378(8)	C(29A)-C(30A)	1.406(3)
C(29A)-C(32A)	1.509(3)	C(29B)-C(30B)	1.412(8)
C(29B)-C(32B)	1.520(8)	C(30A)-C(31A)	1.393(3)
C(30A)-C(33A)	1.515(3)	C(30B)-C(31B)	1.378(7)
C(30B)-C(33B)	1.526(7)	C(31A)-H(31A)	0.9500
C(31B)-H(31B)	0.9500	C(32A)-H(32A)	0.9800
C(32A)-H(32B)	0.9800	C(32A)-H(32C)	0.9800
C(32B)-H(32D)	0.9800	C(32B)-H(32E)	0.9800
C(32B)-H(32F)	0.9800	C(33A)-H(33A)	0.9900
C(33A)-H(33B)	0.9900	C(33B)-H(33C)	0.9900
C(33B)-H(33D)	0.9900	C(34)-C(35)	1.399(2)
C(34)-C(39)	1.398(2)	C(35)-H(35)	0.9500
C(35)-C(36)	1.392(2)	C(36)-H(36)	0.9500
C(36)-C(37)	1.384(3)	C(37)-H(37)	0.9500
C(37)-C(38)	1.380(3)	C(38)-H(38)	0.9500
C(38)-C(39)	1.390(2)	C(39)-H(39)	0.9500
C(40A)-C(41A)	1.393(5)	C(40A)-C(45A)	1.396(5)
C(40B)-C(41B)	1.371(11)	C(40B)-C(45B)	1.365(10)
C(41A)-H(41A)	0.9500	C(41A)-C(42A)	1.381(4)
C(41B)-H(41B)	0.9500	C(41B)-C(42B)	1.440(13)
C(42A)-H(42A)	0.9500	C(42A)-C(43A)	1.387(5)
C(42B)-H(42B)	0.9500	C(42B)-C(43B)	1.365(10)
C(43A)-H(43A)	0.9500	C(43A)-C(44A)	1.367(5)
C(43B)-H(43B)	0.9500	C(43B)-C(44B)	1.350(9)
C(44A)-H(44A)	0.9500	C(44A)-C(45A)	1.395(4)
C(44B)-H(44B)	0.9500	C(44B)-C(45B)	1.377(10)
C(45A)-H(45A)	0.9500	C(45B)-H(45B)	0.9500
C(46A)-C(47A)	1.413(4)	C(46A)-C(51A)	1.398(4)
C(46B)-C(51B)	1.3900	C(46B)-C(47B)	1.3900
C(51B)-H(51B)	0.9500	C(51B)-C(50B)	1.3900
C(50B)-H(50B)	0.9500	C(50B)-C(49B)	1.3900
C(49B)-H(49B)	0.9500	C(49B)-C(48B)	1.3900
C(48B)-H(48B)	0.9500	C(48B)-C(47B)	1.3900
C(47B)-H(47B)	0.9500	C(47A)-H(47A)	0.9500
C(47A)-C(48A)	1.388(3)	C(48A)-H(48A)	0.9500

C(48A)-C(49A)	1.377(5)	C(49A)-H(49A)	0.9500
C(49A)-C(50A)	1.388(5)	C(50A)-H(50A)	0.9500
C(50A)-C(51A)	1.399(3)	C(51A)-H(51A)	0.9500
C(52)-C(53)	1.396(2)	C(52)-C(57)	1.401(2)
C(53)-H(53)	0.9500	C(53)-C(54)	1.390(2)
C(54)-H(54)	0.9500	C(54)-C(55)	1.378(3)
C(55)-H(55)	0.9500	C(55)-C(56)	1.381(3)
C(56)-H(56)	0.9500	C(56)-C(57)	1.388(2)
C(57)-H(57)	0.9500	C(58)-C(59)	1.3997(19)
C(58)-C(63)	1.392(2)	C(59)-H(59)	0.9500
C(59)-C(60)	1.390(2)	C(60)-H(60)	0.9500
C(60)-C(61)	1.379(3)	C(61)-H(61)	0.9500
C(61)-C(62)	1.376(3)	C(62)-H(62)	0.9500
C(62)-C(63)	1.393(2)	C(63)-H(63)	0.9500
C(64)-C(65)	1.397(2)	C(64)-C(69)	1.4013(19)
C(65)-H(65)	0.9500	C(65)-C(66)	1.392(2)
C(66)-H(66)	0.9500	C(66)-C(67)	1.386(3)
C(67)-H(67)	0.9500	C(67)-C(68)	1.383(3)
C(68)-H(68)	0.9500	C(68)-C(69)	1.388(2)
C(69)-H(69)	0.9500	C(70)-H(70)	0.9500
C(70)-C(71)	1.381(2)	C(71)-H(71)	0.9500
C(71)-C(72)	1.379(2)	C(72)-H(72)	0.9500
C(72)-C(73)	1.381(2)	C(73)-H(73)	0.9500
C(73)-C(74)	1.385(2)	C(74)-H(74)	0.9500

O(1)-Mo(1)-N(2)	80.85(4)	O(2)-Mo(1)-O(1)	95.76(5)
O(2)-Mo(1)-O(3)	94.00(4)	O(2)-Mo(1)-N(2)	164.52(5)
O(3)-Mo(1)-O(1)	145.04(5)	O(3)-Mo(1)-N(2)	80.88(4)
C(1A)-Mo(1)-O(1)	106.74(15)	C(1A)-Mo(1)-O(2)	102.83(14)
C(1A)-Mo(1)-O(3)	103.63(15)	C(1A)-Mo(1)-N(2)	92.59(14)
C(1B)-Mo(1)-O(1)	101.7(3)	C(1B)-Mo(1)-O(2)	100.6(3)
C(1B)-Mo(1)-O(3)	109.3(3)	C(1B)-Mo(1)-N(2)	94.9(3)
O(1)-Si(1)-C(10A)	108.91(13)	O(1)-Si(1)-C(10B)	112.4(3)
O(1)-Si(1)-C(34)	111.18(7)	O(1)-Si(1)-C(40A)	110.07(12)
O(1)-Si(1)-C(40B)	113.6(3)	C(10A)-Si(1)-C(34)	113.60(13)
C(10A)-Si(1)-C(40A)	109.71(18)	C(34)-Si(1)-C(10B)	108.2(3)

C(34)-Si(1)-C(40A)	103.25(12)	C(34)-Si(1)-C(40B)	109.6(3)
C(40B)-Si(1)-C(10B)	101.2(3)	O(2)-Si(2)-C(18A)	106.07(14)
O(2)-Si(2)-C(18B)	111.4(3)	O(2)-Si(2)-C(46A)	112.24(17)
O(2)-Si(2)-C(46B)	110.6(3)	O(2)-Si(2)-C(52)	109.30(6)
C(18A)-Si(2)-C(46A)	113.4(2)	C(18A)-Si(2)-C(52)	108.24(15)
C(46A)-Si(2)-C(52)	107.53(13)	C(46B)-Si(2)-C(18B)	108.3(4)
C(52)-Si(2)-C(18B)	109.0(3)	C(52)-Si(2)-C(46B)	108.2(2)
O(3)-Si(3)-C(26A)	111.25(16)	O(3)-Si(3)-C(26B)	106.7(4)
O(3)-Si(3)-C(58)	109.51(6)	O(3)-Si(3)-C(64)	113.58(6)
C(26A)-Si(3)-C(58)	108.23(17)	C(26A)-Si(3)-C(64)	106.57(16)
C(58)-Si(3)-C(26B)	106.3(4)	C(58)-Si(3)-C(64)	107.50(6)
C(64)-Si(3)-C(26B)	113.0(4)	Si(1)-O(1)-Mo(1)	163.63(7)
Si(2)-O(2)-Mo(1)	167.91(7)	Si(3)-O(3)-Mo(1)	164.50(6)
C(17A)-N(1)-C(25A)	115.40(14)	C(17A)-N(1)-C(33A)	109.77(13)
C(17B)-N(1)-C(25B)	105.4(3)	C(25A)-N(1)-C(33A)	109.71(13)
C(33B)-N(1)-C(17B)	117.6(3)	C(33B)-N(1)-C(25B)	106.9(3)
C(70)-N(2)-Mo(1)	117.49(9)	C(74)-N(2)-Mo(1)	124.80(9)
C(74)-N(2)-C(70)	117.68(12)	C(2A)-C(1A)-Mo(1)	171.0(3)
C(2B)-C(1B)-Mo(1)	173.8(8)	C(1A)-C(2A)-C(7A)	118.8(4)
C(3A)-C(2A)-C(1A)	120.9(4)	C(3A)-C(2A)-C(7A)	120.2(3)
C(3B)-C(2B)-C(1B)	117.0(6)	C(3B)-C(2B)-C(7B)	124.0(7)
C(7B)-C(2B)-C(1B)	118.9(6)	C(2A)-C(3A)-C(8A)	118.2(3)
C(4A)-C(3A)-C(2A)	118.3(3)	C(4A)-C(3A)-C(8A)	123.5(4)
C(2B)-C(3B)-C(4B)	117.3(6)	C(2B)-C(3B)-C(8B)	124.6(7)
C(4B)-C(3B)-C(8B)	118.1(6)	C(3A)-C(4A)-H(4A)	118.9
C(3A)-C(4A)-C(5A)	122.2(3)	C(5A)-C(4A)-H(4A)	118.9
C(3B)-C(4B)-H(4B)	120.8	C(5B)-C(4B)-C(3B)	118.5(6)
C(5B)-C(4B)-H(4B)	120.8	C(4A)-C(5A)-H(5A)	119.8
C(6A)-C(5A)-C(4A)	120.3(3)	C(6A)-C(5A)-H(5A)	119.8
C(4B)-C(5B)-H(5B)	119.2	C(6B)-C(5B)-C(4B)	121.6(6)
C(6B)-C(5B)-H(5B)	119.2	C(5A)-C(6A)-H(6A)	119.6
C(5A)-C(6A)-C(7A)	120.7(3)	C(7A)-C(6A)-H(6A)	119.6
C(5B)-C(6B)-H(6B)	118.7	C(5B)-C(6B)-C(7B)	122.7(6)
C(7B)-C(6B)-H(6B)	118.7	C(2A)-C(7A)-C(9A)	121.0(3)
C(6A)-C(7A)-C(2A)	118.3(3)	C(6A)-C(7A)-C(9A)	120.7(3)
C(2B)-C(7B)-C(6B)	115.9(5)	C(2B)-C(7B)-C(9B)	121.5(6)

C(6B)-C(7B)-C(9B)	122.6(6)	C(3A)-C(8A)-H(8AA)	109.5
C(3A)-C(8A)-H(8AB)	109.5	C(3A)-C(8A)-H(8AC)	109.5
H(8AA)-C(8A)-H(8AB)	109.5	H(8AA)-C(8A)-H(8AC)	109.5
H(8AB)-C(8A)-H(8AC)	109.5	C(3B)-C(8B)-H(8BA)	109.5
C(3B)-C(8B)-H(8BB)	109.5	C(3B)-C(8B)-H(8BC)	109.5
H(8BA)-C(8B)-H(8BB)	109.5	H(8BA)-C(8B)-H(8BC)	109.5
H(8BB)-C(8B)-H(8BC)	109.5	C(7A)-C(9A)-H(9AA)	109.5
C(7A)-C(9A)-H(9AB)	109.5	C(7A)-C(9A)-H(9AC)	109.5
H(9AA)-C(9A)-H(9AB)	109.5	H(9AA)-C(9A)-H(9AC)	109.5
H(9AB)-C(9A)-H(9AC)	109.5	C(7B)-C(9B)-H(9BA)	109.5
C(7B)-C(9B)-H(9BB)	109.5	C(7B)-C(9B)-H(9BC)	109.5
H(9BA)-C(9B)-H(9BB)	109.5	H(9BA)-C(9B)-H(9BC)	109.5
H(9BB)-C(9B)-H(9BC)	109.5	C(11A)-C(10A)-Si(1)	126.1(4)
C(15A)-C(10A)-Si(1)	119.5(3)	C(15A)-C(10A)-C(11A)	113.8(4)
C(11B)-C(10B)-Si(1)	122.9(9)	C(11B)-C(10B)-C(15B)	118.9(9)
C(15B)-C(10B)-Si(1)	117.2(5)	C(10A)-C(11A)-H(11A)	118.7
C(12A)-C(11A)-C(10A)	122.6(6)	C(12A)-C(11A)-H(11A)	118.7
C(10B)-C(11B)-H(11B)	120.8	C(10B)-C(11B)-C(12B)	118.4(12)
C(12B)-C(11B)-H(11B)	120.8	C(11A)-C(12A)-H(12A)	118.8
C(11A)-C(12A)-C(13A)	122.4(4)	C(13A)-C(12A)-H(12A)	118.8
C(11B)-C(12B)-H(12B)	120.9	C(13B)-C(12B)-C(11B)	118.1(8)
C(13B)-C(12B)-H(12B)	120.9	C(12A)-C(13A)-C(16A)	119.5(3)
C(14A)-C(13A)-C(12A)	117.2(3)	C(14A)-C(13A)-C(16A)	123.2(2)
C(12B)-C(13B)-C(16B)	119.6(7)	C(14B)-C(13B)-C(12B)	119.9(7)
C(14B)-C(13B)-C(16B)	120.4(7)	C(13A)-C(14A)-C(15A)	119.7(2)
C(13A)-C(14A)-C(17A)	124.01(19)	C(15A)-C(14A)-C(17A)	116.27(19)
C(13B)-C(14B)-C(15B)	119.0(6)	C(13B)-C(14B)-C(17B)	120.0(5)
C(15B)-C(14B)-C(17B)	120.7(5)	C(10A)-C(15A)-C(14A)	123.8(3)
C(10A)-C(15A)-H(15A)	118.1	C(14A)-C(15A)-H(15A)	118.1
C(10B)-C(15B)-H(15B)	118.9	C(14B)-C(15B)-C(10B)	122.1(5)
C(14B)-C(15B)-H(15B)	118.9	C(13A)-C(16A)-H(16A)	109.5
C(13A)-C(16A)-H(16B)	109.5	C(13A)-C(16A)-H(16C)	109.5
H(16A)-C(16A)-H(16B)	109.5	H(16A)-C(16A)-H(16C)	109.5
H(16B)-C(16A)-H(16C)	109.5	C(13B)-C(16B)-H(16D)	109.5
C(13B)-C(16B)-H(16E)	109.5	C(13B)-C(16B)-H(16F)	109.5
H(16D)-C(16B)-H(16E)	109.5	H(16D)-C(16B)-H(16F)	109.5

H(16E)-C(16B)-H(16F)	109.5	N(1)-C(17A)-C(14A)	110.44(15)
N(1)-C(17A)-H(17A)	109.6	N(1)-C(17A)-H(17B)	109.6
C(14A)-C(17A)-H(17A)	109.6	C(14A)-C(17A)-H(17B)	109.6
H(17A)-C(17A)-H(17B)	108.1	N(1)-C(17B)-C(14B)	114.5(4)
N(1)-C(17B)-H(17C)	108.6	N(1)-C(17B)-H(17D)	108.6
C(14B)-C(17B)-H(17C)	108.6	C(14B)-C(17B)-H(17D)	108.6
H(17C)-C(17B)-H(17D)	107.6	C(19A)-C(18A)-Si(2)	123.1(3)
C(23A)-C(18A)-Si(2)	120.8(3)	C(23A)-C(18A)-C(19A)	116.1(4)
C(19B)-C(18B)-Si(2)	122.2(8)	C(19B)-C(18B)-C(23B)	119.1(9)
C(23B)-C(18B)-Si(2)	118.7(6)	C(18A)-C(19A)-H(19A)	119.5
C(20A)-C(19A)-C(18A)	121.0(4)	C(20A)-C(19A)-H(19A)	119.5
C(18B)-C(19B)-H(19B)	119.9	C(18B)-C(19B)-C(20B)	120.2(8)
C(20B)-C(19B)-H(19B)	119.9	C(19A)-C(20A)-H(20A)	119.1
C(19A)-C(20A)-C(21A)	121.7(3)	C(21A)-C(20A)-H(20A)	119.1
C(19B)-C(20B)-H(20B)	119.6	C(21B)-C(20B)-C(19B)	120.9(7)
C(21B)-C(20B)-H(20B)	119.6	C(20A)-C(21A)-C(22A)	118.6(2)
C(20A)-C(21A)-C(24A)	118.4(2)	C(22A)-C(21A)-C(24A)	123.1(2)
C(20B)-C(21B)-C(24B)	118.8(6)	C(22B)-C(21B)-C(20B)	119.3(6)
C(22B)-C(21B)-C(24B)	121.9(5)	C(21A)-C(22A)-C(25A)	122.55(18)
C(23A)-C(22A)-C(21A)	118.87(19)	C(23A)-C(22A)-C(25A)	118.58(17)
C(21B)-C(22B)-C(23B)	119.4(5)	C(21B)-C(22B)-C(25B)	119.7(5)
C(23B)-C(22B)-C(25B)	120.6(4)	C(18A)-C(23A)-C(22A)	123.8(2)
C(18A)-C(23A)-H(23A)	118.1	C(22A)-C(23A)-H(23A)	118.1
C(18B)-C(23B)-H(23B)	119.5	C(22B)-C(23B)-C(18B)	121.0(6)
C(22B)-C(23B)-H(23B)	119.5	C(21A)-C(24A)-H(24A)	109.5
C(21A)-C(24A)-H(24B)	109.5	C(21A)-C(24A)-H(24C)	109.5
H(24A)-C(24A)-H(24B)	109.5	H(24A)-C(24A)-H(24C)	109.5
H(24B)-C(24A)-H(24C)	109.5	C(21B)-C(24B)-H(24D)	109.5
C(21B)-C(24B)-H(24E)	109.5	C(21B)-C(24B)-H(24F)	109.5
H(24D)-C(24B)-H(24E)	109.5	H(24D)-C(24B)-H(24F)	109.5
H(24E)-C(24B)-H(24F)	109.5	N(1)-C(25A)-C(22A)	110.29(14)
N(1)-C(25A)-H(25A)	109.6	N(1)-C(25A)-H(25B)	109.6
C(22A)-C(25A)-H(25A)	109.6	C(22A)-C(25A)-H(25B)	109.6
H(25A)-C(25A)-H(25B)	108.1	N(1)-C(25B)-H(25C)	108.4
N(1)-C(25B)-H(25D)	108.4	C(22B)-C(25B)-N(1)	115.3(4)
C(22B)-C(25B)-H(25C)	108.4	C(22B)-C(25B)-H(25D)	108.4

H(25C)-C(25B)-H(25D)	107.5	C(27A)-C(26A)-Si(3)	122.4(3)
C(27A)-C(26A)-C(31A)	114.5(4)	C(31A)-C(26A)-Si(3)	123.1(3)
C(27B)-C(26B)-Si(3)	118.1(8)	C(31B)-C(26B)-Si(3)	117.0(9)
C(31B)-C(26B)-C(27B)	123.8(12)	C(26A)-C(27A)-H(27A)	118.4
C(28A)-C(27A)-C(26A)	123.1(4)	C(28A)-C(27A)-H(27A)	118.4
C(26B)-C(27B)-H(27B)	121.7	C(26B)-C(27B)-C(28B)	116.7(9)
C(28B)-C(27B)-H(27B)	121.7	C(27A)-C(28A)-H(28A)	119.3
C(27A)-C(28A)-C(29A)	121.4(3)	C(29A)-C(28A)-H(28A)	119.3
C(27B)-C(28B)-H(28B)	120.0	C(29B)-C(28B)-C(27B)	120.0(7)
C(29B)-C(28B)-H(28B)	120.0	C(28A)-C(29A)-C(30A)	118.41(19)
C(28A)-C(29A)-C(32A)	120.3(2)	C(30A)-C(29A)-C(32A)	121.33(19)
C(28B)-C(29B)-C(30B)	119.5(5)	C(28B)-C(29B)-C(32B)	117.9(6)
C(30B)-C(29B)-C(32B)	122.5(5)	C(29A)-C(30A)-C(33A)	119.55(16)
C(31A)-C(30A)-C(29A)	119.16(17)	C(31A)-C(30A)-C(33A)	121.08(16)
C(29B)-C(30B)-C(33B)	123.0(4)	C(31B)-C(30B)-C(29B)	118.9(5)
C(31B)-C(30B)-C(33B)	118.1(4)	C(26A)-C(31A)-H(31A)	118.3
C(30A)-C(31A)-C(26A)	123.4(3)	C(30A)-C(31A)-H(31A)	118.3
C(26B)-C(31B)-C(30B)	120.9(8)	C(26B)-C(31B)-H(31B)	119.5
C(30B)-C(31B)-H(31B)	119.5	C(29A)-C(32A)-H(32A)	109.5
C(29A)-C(32A)-H(32B)	109.5	C(29A)-C(32A)-H(32C)	109.5
H(32A)-C(32A)-H(32B)	109.5	H(32A)-C(32A)-H(32C)	109.5
H(32B)-C(32A)-H(32C)	109.5	C(29B)-C(32B)-H(32D)	109.5
C(29B)-C(32B)-H(32E)	109.5	C(29B)-C(32B)-H(32F)	109.5
H(32D)-C(32B)-H(32E)	109.5	H(32D)-C(32B)-H(32F)	109.5
H(32E)-C(32B)-H(32F)	109.5	N(1)-C(33A)-C(30A)	115.78(14)
N(1)-C(33A)-H(33A)	108.3	N(1)-C(33A)-H(33B)	108.3
C(30A)-C(33A)-H(33A)	108.3	C(30A)-C(33A)-H(33B)	108.3
H(33A)-C(33A)-H(33B)	107.4	N(1)-C(33B)-C(30B)	105.4(4)
N(1)-C(33B)-H(33C)	110.7	N(1)-C(33B)-H(33D)	110.7
C(30B)-C(33B)-H(33C)	110.7	C(30B)-C(33B)-H(33D)	110.7
H(33C)-C(33B)-H(33D)	108.8	C(35)-C(34)-Si(1)	120.40(11)
C(39)-C(34)-Si(1)	121.60(12)	C(39)-C(34)-C(35)	117.59(14)
C(34)-C(35)-H(35)	119.2	C(36)-C(35)-C(34)	121.53(16)
C(36)-C(35)-H(35)	119.2	C(35)-C(36)-H(36)	120.2
C(37)-C(36)-C(35)	119.55(17)	C(37)-C(36)-H(36)	120.2
C(36)-C(37)-H(37)	120.0	C(38)-C(37)-C(36)	119.98(16)

C(38)-C(37)-H(37)	120.0	C(37)-C(38)-H(38)	119.8
C(37)-C(38)-C(39)	120.38(17)	C(39)-C(38)-H(38)	119.8
C(34)-C(39)-H(39)	119.6	C(38)-C(39)-C(34)	120.89(17)
C(38)-C(39)-H(39)	119.6	C(41A)-C(40A)-Si(1)	121.3(3)
C(41A)-C(40A)-C(45A)	115.6(3)	C(45A)-C(40A)-Si(1)	122.9(3)
C(41B)-C(40B)-Si(1)	114.6(6)	C(45B)-C(40B)-Si(1)	124.6(6)
C(45B)-C(40B)-C(41B)	120.8(9)	C(40A)-C(41A)-H(41A)	119.0
C(42A)-C(41A)-C(40A)	121.9(3)	C(42A)-C(41A)-H(41A)	119.0
C(40B)-C(41B)-H(41B)	120.3	C(40B)-C(41B)-C(42B)	119.4(8)
C(42B)-C(41B)-H(41B)	120.3	C(41A)-C(42A)-H(42A)	119.8
C(41A)-C(42A)-C(43A)	120.5(3)	C(43A)-C(42A)-H(42A)	119.8
C(41B)-C(42B)-H(42B)	121.4	C(43B)-C(42B)-C(41B)	117.2(8)
C(43B)-C(42B)-H(42B)	121.4	C(42A)-C(43A)-H(43A)	120.2
C(44A)-C(43A)-C(42A)	119.7(2)	C(44A)-C(43A)-H(43A)	120.2
C(42B)-C(43B)-H(43B)	118.8	C(44B)-C(43B)-C(42B)	122.4(8)
C(44B)-C(43B)-H(43B)	118.8	C(43A)-C(44A)-H(44A)	120.5
C(43A)-C(44A)-C(45A)	119.0(3)	C(45A)-C(44A)-H(44A)	120.5
C(43B)-C(44B)-H(44B)	119.8	C(43B)-C(44B)-C(45B)	120.3(7)
C(45B)-C(44B)-H(44B)	119.8	C(40A)-C(45A)-H(45A)	118.4
C(44A)-C(45A)-C(40A)	123.2(3)	C(44A)-C(45A)-H(45A)	118.4
C(40B)-C(45B)-C(44B)	119.8(7)	C(40B)-C(45B)-H(45B)	120.1
C(44B)-C(45B)-H(45B)	120.1	C(47A)-C(46A)-Si(2)	120.0(2)
C(51A)-C(46A)-Si(2)	123.5(3)	C(51A)-C(46A)-C(47A)	116.4(2)
C(51B)-C(46B)-Si(2)	119.2(4)	C(51B)-C(46B)-C(47B)	120.0
C(47B)-C(46B)-Si(2)	120.8(4)	C(46B)-C(51B)-H(51B)	120.0
C(46B)-C(51B)-C(50B)	120.0	C(50B)-C(51B)-H(51B)	120.0
C(51B)-C(50B)-H(50B)	120.0	C(51B)-C(50B)-C(49B)	120.0
C(49B)-C(50B)-H(50B)	120.0	C(50B)-C(49B)-H(49B)	120.0
C(48B)-C(49B)-C(50B)	120.0	C(48B)-C(49B)-H(49B)	120.0
C(49B)-C(48B)-H(48B)	120.0	C(49B)-C(48B)-C(47B)	120.0
C(47B)-C(48B)-H(48B)	120.0	C(46B)-C(47B)-H(47B)	120.0
C(48B)-C(47B)-C(46B)	120.0	C(48B)-C(47B)-H(47B)	120.0
C(46A)-C(47A)-H(47A)	118.8	C(48A)-C(47A)-C(46A)	122.4(2)
C(48A)-C(47A)-H(47A)	118.8	C(47A)-C(48A)-H(48A)	120.2
C(49A)-C(48A)-C(47A)	119.5(2)	C(49A)-C(48A)-H(48A)	120.2
C(48A)-C(49A)-H(49A)	120.0	C(48A)-C(49A)-C(50A)	120.1(2)

C(50A)-C(49A)-H(49A)	120.0	C(49A)-C(50A)-H(50A)	120.0
C(49A)-C(50A)-C(51A)	120.1(2)	C(51A)-C(50A)-H(50A)	120.0
C(46A)-C(51A)-C(50A)	121.4(2)	C(46A)-C(51A)-H(51A)	119.3
C(50A)-C(51A)-H(51A)	119.3	C(53)-C(52)-Si(2)	121.78(12)
C(53)-C(52)-C(57)	117.12(14)	C(57)-C(52)-Si(2)	121.11(11)
C(52)-C(53)-H(53)	119.3	C(54)-C(53)-C(52)	121.35(16)
C(54)-C(53)-H(53)	119.3	C(53)-C(54)-H(54)	119.9
C(55)-C(54)-C(53)	120.19(16)	C(55)-C(54)-H(54)	119.9
C(54)-C(55)-H(55)	120.0	C(54)-C(55)-C(56)	119.92(15)
C(56)-C(55)-H(55)	120.0	C(55)-C(56)-H(56)	120.1
C(55)-C(56)-C(57)	119.83(16)	C(57)-C(56)-H(56)	120.1
C(52)-C(57)-H(57)	119.2	C(56)-C(57)-C(52)	121.60(15)
C(56)-C(57)-H(57)	119.2	C(59)-C(58)-Si(3)	120.03(11)
C(63)-C(58)-Si(3)	122.24(11)	C(63)-C(58)-C(59)	117.59(13)
C(58)-C(59)-H(59)	119.4	C(60)-C(59)-C(58)	121.15(15)
C(60)-C(59)-H(59)	119.4	C(59)-C(60)-H(60)	120.0
C(61)-C(60)-C(59)	120.06(15)	C(61)-C(60)-H(60)	120.0
C(60)-C(61)-H(61)	120.1	C(62)-C(61)-C(60)	119.87(15)
C(62)-C(61)-H(61)	120.1	C(61)-C(62)-H(62)	119.9
C(61)-C(62)-C(63)	120.23(16)	C(63)-C(62)-H(62)	119.9
C(58)-C(63)-C(62)	121.10(15)	C(58)-C(63)-H(63)	119.5
C(62)-C(63)-H(63)	119.5	C(65)-C(64)-Si(3)	121.69(10)
C(65)-C(64)-C(69)	117.53(13)	C(69)-C(64)-Si(3)	120.78(11)
C(64)-C(65)-H(65)	119.3	C(66)-C(65)-C(64)	121.37(14)
C(66)-C(65)-H(65)	119.3	C(65)-C(66)-H(66)	120.1
C(67)-C(66)-C(65)	119.76(15)	C(67)-C(66)-H(66)	120.1
C(66)-C(67)-H(67)	120.0	C(68)-C(67)-C(66)	120.05(14)
C(68)-C(67)-H(67)	120.0	C(67)-C(68)-H(68)	120.0
C(67)-C(68)-C(69)	119.92(15)	C(69)-C(68)-H(68)	120.0
C(64)-C(69)-H(69)	119.3	C(68)-C(69)-C(64)	121.34(15)
C(68)-C(69)-H(69)	119.3	N(2)-C(70)-H(70)	118.6
N(2)-C(70)-C(71)	122.84(14)	C(71)-C(70)-H(70)	118.6
C(70)-C(71)-H(71)	120.6	C(72)-C(71)-C(70)	118.90(14)
C(72)-C(71)-H(71)	120.6	C(71)-C(72)-H(72)	120.6
C(71)-C(72)-C(73)	118.85(15)	C(73)-C(72)-H(72)	120.6
C(72)-C(73)-H(73)	120.5	C(72)-C(73)-C(74)	119.06(15)

C(74)-C(73)-H(73)	120.5	N(2)-C(74)-C(73)	122.65(14)
N(2)-C(74)-H(74)	118.7	C(73)-C(74)-H(74)	118.7
